# BacEffluxPred: A two-tier system to predict and categorize bacterial efflux mediated antibiotic resistance proteins

**DOI:** 10.1038/s41598-020-65981-3

**Published:** 2020-06-09

**Authors:** Deeksha Pandey, Bandana Kumari, Neelja Singhal, Manish Kumar

**Affiliations:** Department of Biophysics, University of Delhi South Campus, New Delhi, 110021 Delhi India

**Keywords:** Computational biology and bioinformatics, Microbiology, Medical research, Pathogenesis

## Abstract

Efflux proteins are transport proteins, which are involved in transporting different substrates from the cell to the external environment, including antibiotics. The efflux mechanism and efflux pumps are a major reason underlying emerging rampant antibiotic resistance (AR) in microbes. To reduce the resources required and time of identification, characterization and classification of bacterial efflux proteins, we have developed a fast and accurate support vector machine based two-tier prediction system, BacEffluxPred, which can predict bacterial efflux proteins responsible for AR and identify their corresponding families. A leave-one-out cross-validation also called jackknife procedure was used for performance evaluation. The accuracy to discriminate bacterial AR efflux from non-AR efflux was obtained as 85.81% (at tier-I) while accuracies for prediction of efflux pump families like ABC, MFS, RND and MATE family were found 92.13%, 85.39%, 91.01% and 99.44%, respectively (at tier-II). Benchmarking on an independent dataset also showed that BacEffluxPred had comparable accuracy for prediction of bacterial AR efflux pumps and their families. This is the first *in-silico* tool for predicting bacterial AR efflux proteins and their families and is freely available as both web-server and standalone versions at http://proteininformatics.org/mkumar/baceffluxpred/.

## Introduction

Antibiotics are considered as one of the most important discoveries of the nineteenth century. However, with the passage of time, the efficacy of antibiotics has been gradually compromised by the emergence of antibiotic-resistant pathogens^1,2^. Due to the emergence of antibiotic-resistant microbial pathogens, diseases, which were earlier easy to treat, have become difficult to cure. In bacteria several mechanisms contribute to development of antibiotic resistance (AR) for example, (a) evolving mutations in the antibiotic targets, (b) modifications in the bacterial cell surface which prevents antibiotics from penetrating inside the cell, (c) efflux pumps which pump out the antibiotics from the cell even before they reach their target, and (d) producing enzymes which inactivate the antibiotics.

Efflux proteins are ubiquitous in nature and are present in eukaryotic as well as prokaryotic (both Gram-positive and Gram-negative bacteria) organisms. The normal function of bacterial efflux pumps is to prevent intracellular accumulation of toxic compounds by an energy-dependent system. The molecules that are effluxed out of the cell do not undergo any modification or degradation. Estimation on the basis of genomic analyses indicated efflux protein pumps constitute between 6–18% of all the transporters present in any bacterial species^3^. Efflux pumps might be specific for one substrate or may transport a range of structurally dissimilar compounds (including antibiotics of multiple classes). Several studies reported that efflux pumps were associated with multiple drug resistance (MDR) in bacteria^4,5^.

On the basis of sequence similarity pattern, specificity towards different substrates, number of components, number of trans-membrane spanning regions, energy sources and structural features, efflux pumps can be divided in two major families (i) primary transporters, which use ATP as the energy source, hence also called ATP-binding cassette (ABC) transporter^6^, and (ii) secondary transporters, which employ proton (or sodium) gradient as a source of energy. On the basis of sequence conservation and functional similarities, secondary transporters are further divided into four families namely, the major facilitator superfamily (MFS)^7^, the resistance-nodulation and cell division (RND) family^8^, the small multidrug resistance (SMR) family^9^ and the multidrug and toxic compound extrusion (MATE) family^10^. It is well established that efflux pumps have played a key role in the emergence of antibiotic resistance in several bacterial pathogens^2,11–14^.

In the past, several attempts were made to use machine learning tools for prediction of antimicrobial resistance (AMR) genes/proteins in the whole genome as well as at genes/proteins level. A support vector machine (SVM) and pseudo-amino acid composition based two-tier prediction method was developed in our laboratory to predict and classify β-lactamases into four Ambler classes^15^. Later, it was extended to further classify the Metallo-β-lactamases (class B) into three subclasses^16^. An artificial neural network based classifier, DeepARG was developed to identify novel antimicrobial resistance genes in the metagenomic data^17^. Pesesky *et al*.^18^ compared the rules-based and machine-learning predictions with standard phenotypic diagnostic test for twelve antibiotic agents of six major antibiotic classes and, found that the rules-based prediction showed an agreement of 89%, while the machine-learning predictions showed 90.3% agreement with the standard phenotypic tests. Recently, Chowdhury *et al*.^19^ used game theory to reduce the number of features from the bacterial protein sequences and used these features as an input in SVM to identify putative AMR genes encoding, acetyltransferases, β-lactamases, and dihydrofolate reductase in several genera of Gram-negative bacteria like, *Acinetobacter*, *Klebsiella*, *Campylobacter*, *Salmonella* and *Escherichia*. Their method showed 93–99% accuracy in prediction. Recently Kim *et al*.^20^ have utilized the antibiotic resistant bacterial genomic sequences to characterize the genetic features that might be associated with AMR. They have also developed a pipeline, named as VAMPr, to discover variant-level genetic features and its correlation with phenotypic AMR data.

Besides, there are other antibiotic resistance databases that were built on the basis of known antibiotic resistance genes. The most popular resources are ResFinder^21^, the Comprehensive Antibiotic Resistance Database (CARD)^22^, and Resfams^23^. Our laboratory has also developed a database of β–lactamases named as CBMAR^24^. However, we could not find any *in-silico* tool that can discriminate bacterial antibiotic resistance efflux (ARE) proteins from efflux proteins which do not efflux out antibiotics (non-ARE), and/or can predict the family to which an ARE protein might belong.

In the present manuscript, we have described a systematic attempt to build a machine-learning based two-tier *in-silico* tool, named BacEffluxPred which discriminates bacterial ARE proteins from non-ARE and also predicts its respective family. BacEffluxPred completes a prediction cycle in two different tiers. In tier-I, discrimination between ARE and non-ARE proteins is done while in tier-II, family of the ARE protein(s) is predicted. BacEffluxPred has also been evaluated on an independent dataset and a web-server was developed which is freely available for the scientific community. We expect that BacEffluxPred would be helpful to the scientific community in the prediction and annotation of bacterial efflux proteins that confer AR.

## Results

### Tier-I prediction

At tier-I, we achieved 85.81% accuracy with MCC 0.57. The corresponding values of sensitivity and specificity were 80.23% and 86.84%, respectively (Table 1).

**Table 1:** Performance of SVM models at training and independent testing dataset during LOOCV at tier-I and II.

Threshold	Tier		Training Dataset					Independent Testing Dataset				
			AC (%)	SEN (%)	SPE (%)	MCC	AUC	AC (%)	SEN (%)	SPE (%)	MCC	AUC
−0.4	**Tier-I**		85.81	80.23	86.84	0.57	0.87	94.24	86.84	95.61	0.79	0.95
−0.4	**T** **i** **e** **r** **-** **II**	**ABC**	92.13	88.24	93.06	0.77	0.96	93.75	100.00	92.00	0.85	0.96
−0.3		**MFS**	85.39	87.50	83.67	0.71	0.92	93.75	93.33	94.12	0.87	0.97
−0.4		**RND**	91.01	90.00	91.30	0.76	0.94	93.75	100.00	92.00	0.85	1.00
0.3		**MATE**	99.44	95.00	100.00	0.97	0.99	100.00	100.00	100.00	1.00	1.00

The overall performance of SVM models during LOOCV at tier-I and tier-II. AC, SEN, SPE, MCC and AUC represent accuracy, sensitivity, specificity, Matthew’s correlation coefficient and area under the ROC curve respectively.

### Tier-II predictions

At tier-II prediction also, the SVM models were trained using only 5/6 fractions of total ARE proteins (178 in total). These proteins were also used as positive class examples during tier-I prediction. During tier-II, the prediction models were developed to predict the family of tier-I predicted ARE proteins. During training all proteins of a particular family were considered as an example of positive class while proteins of the remaining family were considered as negative class example. For instance, to predict proteins of ABC efflux family, all ABC efflux family protein sequences (total 34 in number) were used as a positive data, while the remaining families, namely MATE, MFS, RND and SMR (total 144 sequences) were considered as examples of negative class. During tier-II prediction, the accuracy achieved during LOOCV was 92.13%, 85.39%, 91.01%, and MCC 0.77, 0.71, and 0.76 in ABC, MFS and RND family prediction, respectively while in case of MATE family, the prediction accuracy and MCC achieved was 99.44% and 0.97, respectively. The sensitivity achieved was 88.24%, 87.50%, 90.00%, and 95.00% and specificity was 93.06%, 83.67%, 91.30%, and 100.00% in best models of ABC, MFS, RND and MATE families, respectively at the tier-II during LOOCV (Table 1).

### Performance on independent testing dataset

We re-evaluated the performance of all prediction models on an independent testing dataset. Prediction model of tier-I showed 94.24% accuracy with MCC as 0.79. The sensitivity and specificity were 86.84% and 95.61%, respectively (Table 1). The overall accuracy and MCC of the tier-II model was more than 93% and 0.8 for ABC, MFS and RND families. For the MATE family proteins we found 100% accuracy and MCC as 1.00 (Table 1**)**. Collectively both tier-I and tier-II SVM models are henceforth referred as BacEffluxPred.

### Receiver operating characteristics plot and area under ROC curve analysis

**Figure 1:**
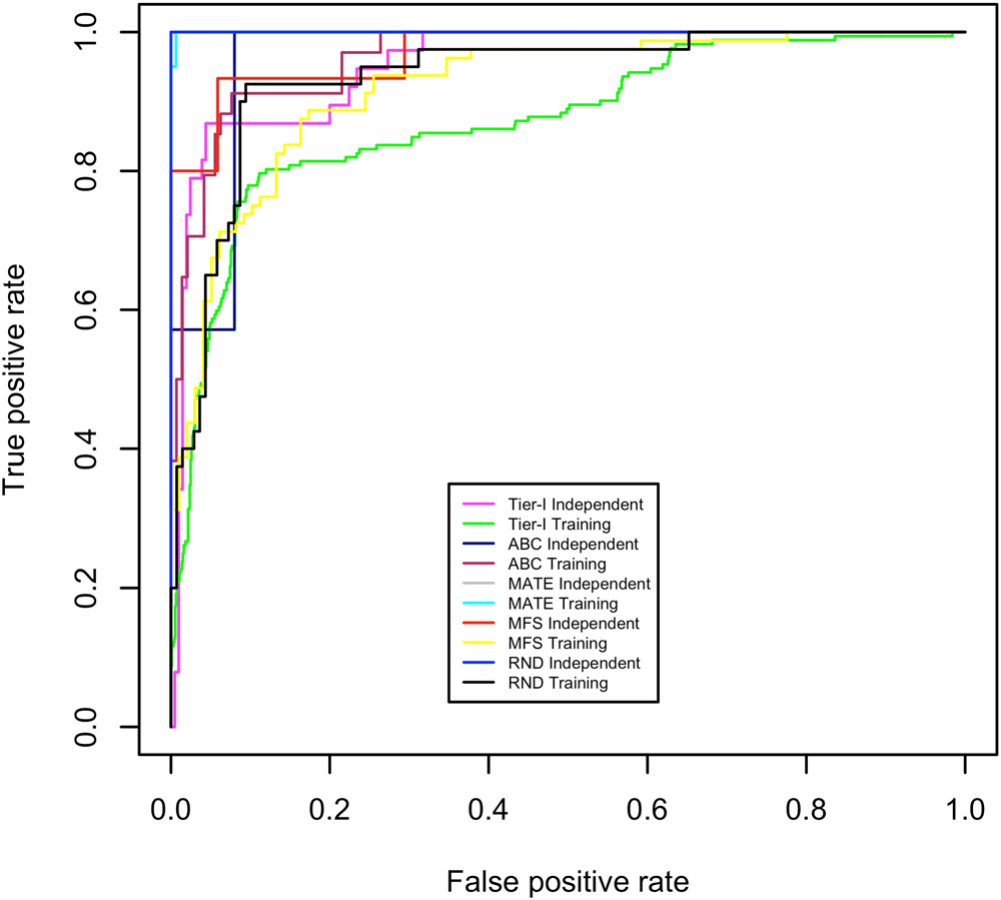
Receiver Operating Characteristics (ROC) Plot: ROC plots showing comparative performance at both tiers on training and independent datasets.

Overall accuracy can be a good indicator to measure overall performance of a predictor but simultaneously overall accuracy might be an unrealistic assessment of a classifier performance on an unbalanced dataset. Therefore, to avoid the impact of majority class during performance estimation, the prediction capability of all SVM modules, developed in the present study, was evaluated in terms of both sensitivity and specificity. We have selected those SVM learning parameters at which both sensitivity and specificity were nearly equal. An alternative way of impartial assessment of a classifier’s efficiency is by using the receiver operating characteristic (ROC) plot^25,26^, which is a very popular way to analyze the overall performance of a classifier system. It displays the trade-off between sensitivity and specificity at various thresholds and is created by plotting ‘sensitivity’ (True positive rate) vs. ‘specificity’ (False positive rate). The area under the ROC curve (AUC) can be used as a summary measure of diagnostic accuracy^27^. The ROC plots (Figure 1) and their corresponding AUC values (Table 1) also supported the conclusion that both SVM modules have very high prediction efficiency at their respective tiers.

### Implementation of web-server and standalone tool

**Figure 2:**
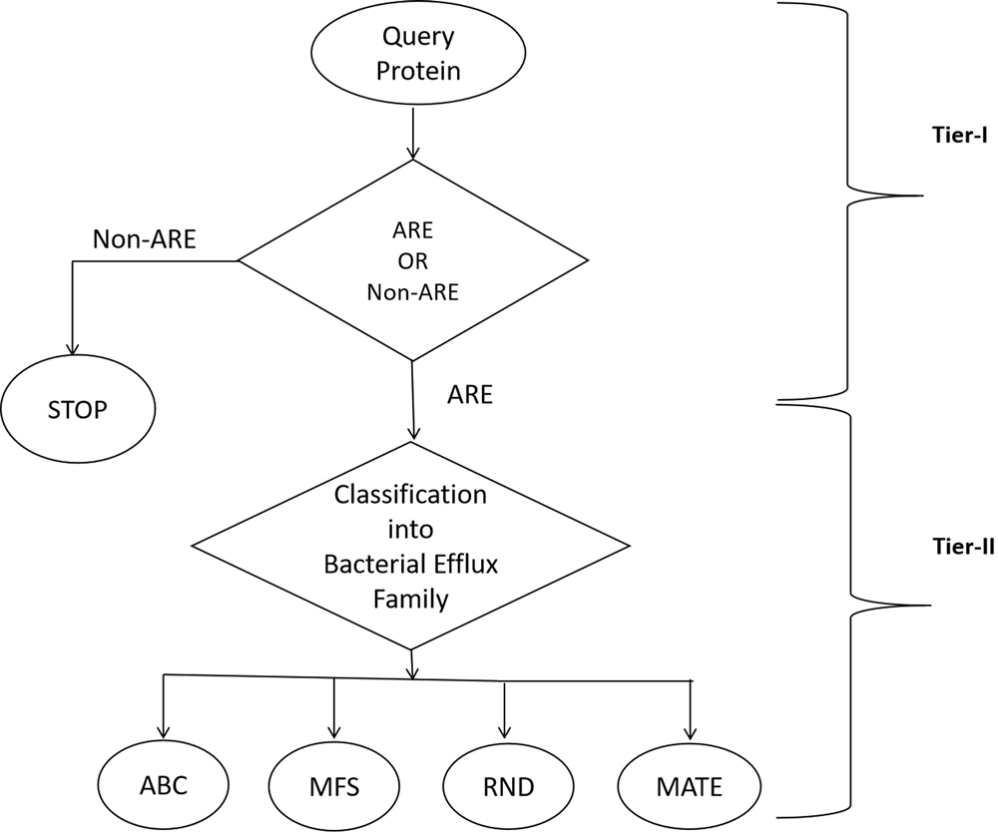
Prediction schema of BacEffluxPred: The prediction schema of BacEffluxPred. Tier-I screens out efflux proteins not involved in antibiotic resistance. If the query protein is predicted as efflux proteins capable of efflux out antibiotics also, it will be forwarded to tier-II, which predicts the efflux protein family to which it might belong.

**Figure 3:**
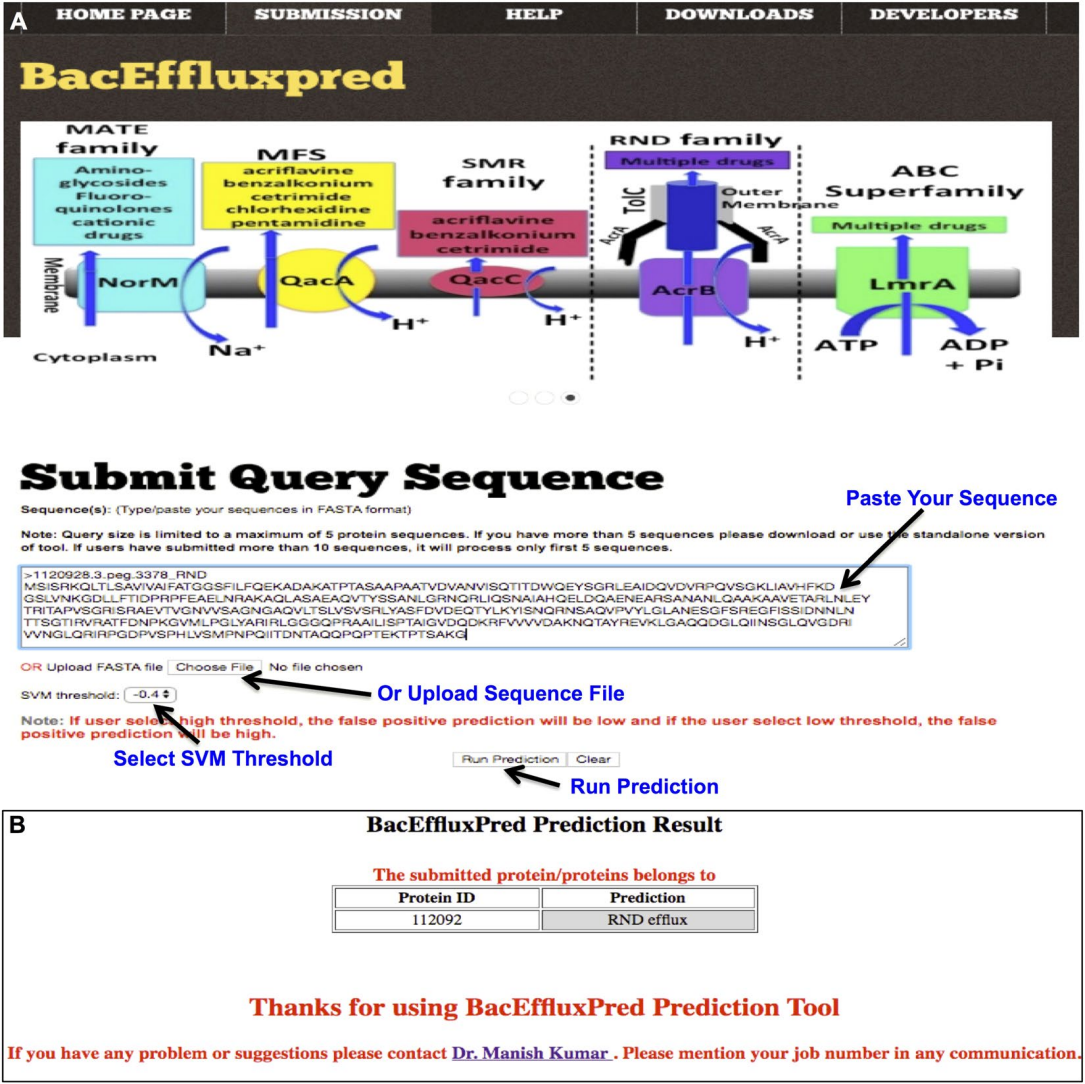
Snapshot of ‘BacEffluxPred’ tool: [**A**] Query submission page. [**B**] Prediction result page.

Using the prediction models developed during this work, we have also established a web-server, named as BacEffluxPred, to predict and classify unknown ARE proteins. Similar to the methodology adopted during training, BacEffluxPred also works on a two-tier prediction approach. At tier-I, BacEffluxPred would decide whether the query protein is an ARE protein or not. At tier-II the predicted ARE protein would be classified into one of the four efflux protein families on the basis of SVM score. The overall schema of prediction methodology of the tool is explained in Figure 2. Snapshots of the query submission and result page of ‘BacEffluxPred’ web-server is shown in Figure 3. The web-server allows users to submit up to five protein sequences at a time for prediction. The query submission page also allows users to set the SVM prediction thresholds. The result page of BacEffluxPred displays results in two columns. The first column displays the ID of the query proteins that users have submitted and the second column shows the prediction result. The BacEffluxPred is available at http://proteininformatics.org/mkumar/baceffluxpred. A standalone version of the tool allows users to analyze a larger dataset. Both web-server and standalone versions as well as datasets which were used to build the tool are freely available at the download section of BacEffluxPred http://proteininformatics.org/mkumar/baceffluxpred/downloads.html.

### Potential use of BacEffluxPred

Recent advances in DNA technology and advent of the genomic era have led to the identification of numerous new efflux pump proteins. As efflux proteins are one of the major factors underlying emergence of MDR in microbial pathogens. Hence, development of an *in-silico* tool, which is capable of predicting antibiotics efflux proteins, can be highly useful in annotation of novel efflux proteins.

## Discussion

**Figure 4:**
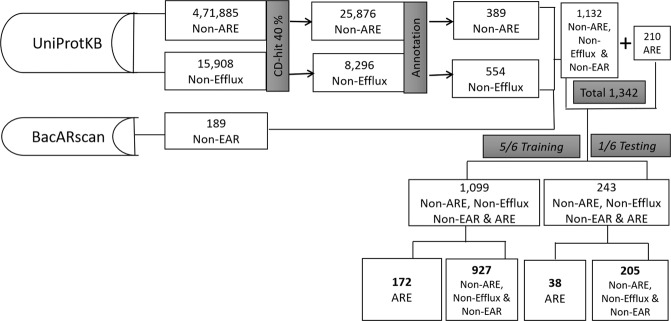
The overall schema of tier-I dataset compilation: Methodology adopted for tier-I dataset compilation. Numerical values indicates the number of proteins. ARE: antibiotic resistance efflux proteins, non-ARE: non-antibiotic resistance efflux proteins, non-efflux: non-efflux prokaryotic proteins, and non-EAR: non-efflux antibiotic resistance proteins.

**Figure 5:**
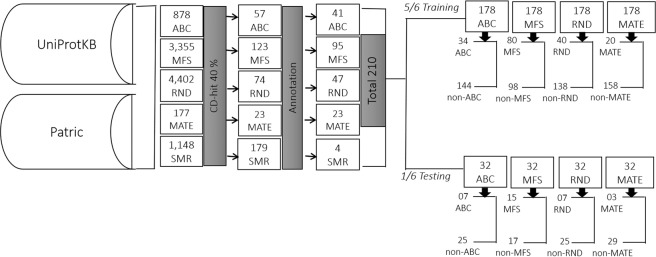
The overall schema of tier-II dataset compilation: Methodology adopted for tier-II dataset compilation. Numerical values indicates the number of proteins. ABC, MFS, RND, MATE and SMR are efflux protein families.

Efflux proteins are essentially transport proteins, which are involved in transporting different substrates (including antibiotics and/or other chemical substances) from the cell to the external environment^28–31^. Efflux proteins that are capable of pumping out the antibiotics from the cell are of the major reasons contributing to AR in several microbes^2,11–14^. Currently to the best of our knowledge, there is no method to predict the bacterial ARE proteins and their families. Hence, in this study we have developed a SVM based highly accurate and novel method named as BacEffluxPred, to predict bacterial ARE proteins and assign the predicted protein to its respective efflux family. To develop the prediction model, we created a manually curated dataset of bacterial ARE proteins and classified them on the basis of their families. During training SVM requires training examples to be labeled as positive and negative classes, hence we divided the training dataset into positive and negative classes. Positive class consisted of bacterial ARE protein sequences, which were retrieved from Patric^32^ and UniProtKB^33^ databases. In the negative class, we put efflux proteins which were unable to pump out antibiotics (non-ARE), non-efflux prokaryotic proteins (non-efflux) and non-efflux antibiotic resistance (non-EAR) proteins (Figure 4 and Figure 5). The complete dataset was further divided into two fractions, which were used to train the predictor and for their independent evaluation of prediction models.

It has been reported in several previous studies that evolutionary information in the form of position specific scoring matrix (PSSM) profiles provide more information during the learning phase of a predictor. Hence, use of PSSM as an input, has significantly improved the prediction accuracy of several prediction methods^34,35^. In the present work we extracted evolutionary information of a protein from PSSM profiles generated during PSI-BLAST search against a 90% non-redundant NR protein database. The complete prediction pipeline runs at two tiers. In tier-I ARE proteins were predicted with 85.81% accuracy (Table 1) and forwarded to tier-II. In the tier-II family of ARE proteins was predicted. The classification accuracies of 92.13%, 85.39%, 91.01% and 99.44% were achieved for ABC, MFS, RND and MATE families, respectively (Table 1). We also assessed the performance of the developed model on an independent data and found comparable performance (Table 1). Similarly, the rate of prediction at tier-II was also found consistent across all the classes. The overall performances of all SVM modules were also compared at both tiers in the form of ROC plot ( Figure 1). The AUC values of each ROC plot also supported the conclusion that SVM models of both tiers can predict AREs at a very high accuracy (Table 1). We also established a web-server and a standalone tool to predict and classify ARE proteins. It can be freely accessed at http://proteininformatics.org/mkumar/baceffluxpred.

## Methods

### Prediction schema

In the present study we tried to solve two different problems simultaneously, hence BacEffluxPred works at two tiers. The 1^st^ problem (referred as tier-I) was to identify the proteins involved in efflux protein mediated antibiotic resistance and the 2^nd^ problem (referred as tier-II) was to predict the family to which each predicted ARE proteins might belong. This indicates that the former is a binary classification problem, which can be addressed by a binary classifier that can classify a query protein into an ARE or non-ARE protein. In the second problem we had to identify the family of an ARE protein (predicted at tier-I), which was a multi-class classification. To solve this, we divided the multi-class classification problem into a series of binary classifications and developed multiple prediction models using one *vs*. rest approach. It involved the development of a classifier for each family of ARE using proteins of one family as positive examples and proteins of remaining families as negative examples. We feel adaptation of the two-tiered prediction approach would provide several benefits to the overall prediction quality of BacEffluxPred. For example, the tier-I would act as a filter and restrict the entry of non-ARE proteins to the ARE family prediction. Further, due to filtering at tier-I a relatively small number of proteins would be presented to tier-II. It reduces the chance of misclassification, which ultimately increases the overall accuracy of prediction. Overall, a complete prediction cycle works in following three steps: 1) the query protein is presented to the prediction algorithm; 2) If the query protein would be predicted to be a non-ARE protein, the prediction would stop after tier-I; 3) If the query protein would be predicted as an ARE protein at the tier-I, the query protein would be forwarded to tier-II for ARE family prediction.

### Data sources and compilation

The Bacterial efflux proteins, which are involved in antibiotics resistance (ARE), were collected from Patric^32^ and UniProtKB^33^ databases using keyword search (Efflux and their associated families i.e. ABC, RND, MATE, MFS and SMR, respectively). For the present work we have used only reviewed and non-fragmented ARE proteins. The ARE proteins were further divided on the basis of efflux protein families namely ABC, MATE, RND, SMR and MFS. In all five families the sequence redundancy was reduced to 40% using CD-HIT^36–38^, which resulted in a total 210 protein sequences.

The negative proteins were composed of non-ARE, non-Efflux and non-efflux antibiotic resistance (non-EAR) proteins were collected from three different sources. (a) First, we searched the UniProtKB database using the keyword ‘Efflux’. After reducing the redundancy using CD-HIT at 40% identity cutoff, we removed all bacterial ARE proteins and finally got a total 389 proteins. (b) Secondly we collected all non-fragmented, non-membranous and non-efflux bacterial proteins from UniProtKB whose existence was established at protein level. After redundancy reduction at 40% using CD-HIT, we randomly selected each 15^th^ protein (total 554). We have selected only 1/15th of total proteins because a large skew between negative and positive data may lead to prediction bias towards the over-represented class. For example in the present work the number of proteins in the negative class is more than the positive class proteins. Hence, a prediction model can achieve high accuracy simply by unilateral prediction of all proteins as negative class proteins irrespective of their correct class. We also added189 non-EAR proteins to the negative dataset. The details and overall statistics of tier-I and tier–II datasets are shown in Figure 4 and Figure 5. The complete data can be downloaded from Supplementary Material.

### Training and independent testing datasets

For training, we divided the complete dataset into two non-overlapping fractions. One fraction, having nearly 5/6 of the total data (1,099 out of total 1,342 protein sequences), was used to train and develop the prediction models while remaining, nearly 1/6 of the total data (243 protein sequences), for independent evaluation of trained models. Similarly in tier-II we used the 178 protein sequences from the complete datasets of 210 ARE protein sequences, which includes ABC, RND, MATE, MFS and SMR family, were used for training. The remaining 32 protein sequences were used as an independent dataset for benchmarking of trained models. The overall statistics and distribution of data is presented in Figure 4 and Figure 5. It is pertinent to mention that in tier-I both ARE and non-ARE proteins were used because its purpose was to discriminate between ARE and non-ARE proteins. On the other hand in tier-II only ARE proteins were used since it was intended to predict the family of predicted ARE proteins. As a result of redundancy reduction only four protein sequences of the SMR family was present in the final non-redundant dataset. Since a very small number of sequences wouldn’t be able to train an efficient prediction model, hence we did not develop the SMR family prediction model.

### Input feature encoding

To train the SVM we have used PSSM computed by PSI-BLAST search against a database that was derived from the NR protein database after reducing sequence redundancy at ≥90%. The PSSM of each sequence was computed by three iterations of PSI-BLAST search with an e-value threshold 0.001. The PSSM contains the probability of occurrence of each type of amino acid at each residue position of a given protein sequence. Therefore the values of PSSM can also be considered as an indication of conservation of amino acids at a given position. It means the PSSM summarizes evolutionary information of each amino acid in a vector of 20 dimensions and hence the size of PSSM for a protein with N residues would be 20 $\times$ N. In the present work, since we used complete protein sequences hence the size of PSSM also varied according to the protein length. Since SVM requires a fixed length input, hence a variable length 20 Χ N matrix was transform into a fixed dimension 20 $\times$ 20 matrix by column wise addition of PSSM scores of each of 20 types of amino acid as described in our previous work^39^.

### Support vector machine

SVM is one of the most popular kernel-based machine-learning method. It can efficiently classify complex, non-linear and high-dimensional data through kernel based calculation^40^. In the present work we have used SVM_light, a freely available software package. During optimization of the SVM model we used several parameters and kernel features (e.g. linear, polynomial, radial basis function, sigmoid etc.) to model the data.

Besides SVM there are also other machine-learning tools used to develop different predictors. In our previous work for predicting palmitoylation sites, we have evaluated three machine-learning methods namely Naive Bayes, RBF Network and Random Forest^35^. We found that the SVM classifier showed higher performance in comparison to Naive Bayes, RBF Network and Random forest classifiers. Hence, in the present work we have used only SVM to develop the predictor.

### Cross-validation

Cross-validation is a way to evaluate the performance of a prediction model on a dataset that is not used to train the model. The two most popular methods of cross-validations are sub-sampling (k-fold cross-validation) and jackknife analysis (leave-one-out or LOOCV). In k-fold cross–validation, as the name suggests, the dataset is arbitrarily divided into k number of non-overlapping sets. All, except one set is used for training, while the remaining one set is used as a test dataset. At each training parameter, the training and testing process is repeated using a distinct train and test set. Therefore at each training parameter k different models were obtained. The performance at each training parameter was calculated by averaging the performances of all test sets. In LOOCV all, except one example is used to train the model and the remaining one example is used to assess the performance of a trained model. Hence, in one cycle of LOOCV the number of prediction models developed is equal to the number of examples in the training dataset. In the present work, the jackknife or LOOCV method of cross-validation was used at both tier-I and tier-II because it is considered less biased in comparison to k-fold cross-validation^41^.

### Performance evaluation

We used sensitivity, specificity, accuracy and MCC to evaluate the performance of prediction models developed at each training parameter. These performance metrics have also been frequently used in several prediction and classification studies^15,16^. The mathematical expressions used to calculate the above-mentioned parameters were as follows:

1 $${\boldsymbol{Sensitivity}}=\frac{{\boldsymbol{TP}}}{{\boldsymbol{TP}}+{\boldsymbol{FN}}}\times 100$$

2 $${\boldsymbol{Specificity}}=\frac{{\boldsymbol{TN}}}{{\boldsymbol{TN}}+{\boldsymbol{FN}}}\times 100$$

3 $${\boldsymbol{Accuracy}}=\frac{{\boldsymbol{TP}}+{\boldsymbol{TN}}}{{\boldsymbol{TP}}+{\boldsymbol{FP}}+{\boldsymbol{TN}}+{\boldsymbol{FN}}}\times 100$$

4 $${\boldsymbol{MCC}}=\frac{({\boldsymbol{TP}}\times {\boldsymbol{TN}})-({\boldsymbol{FP}}\times {\boldsymbol{FN}})}{\sqrt{({\boldsymbol{TP}}+{\boldsymbol{FP}})({\boldsymbol{TP}}+{\boldsymbol{FN}})({\boldsymbol{TN}}+{\boldsymbol{FP}})({\boldsymbol{TN}}+{\boldsymbol{FN}})}}\times 100$$

where, TP, TN, FP, FN and MCC represents true positive, true negative, false positive, false negative and Matthews Correlation Coefficient respectively. Sensitivity and specificity corresponds to the proportion of correct predictions of positive and negative examples. The overall percentage of correctly predicted examples was calculated through accuracy, which was the arithmetic mean of sensitivity and specificity. Since MCC shows the balance between specificity and sensitivity hence, MCC is considered as a reliable parameter of binary classification for asymmetrical datasets^42,43^. The MCC value lies between −1 to 1. A highly successful predictor will have MCC value near to 1, while opposite and random predictions have MCC value −1 and 0, respectively.

**Figure 6:**
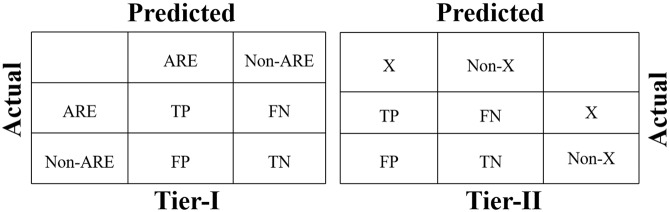
Classification schema of prediction on the basis of actual and prediction state: At tier-I, the decision was made on the basis of whether the query protein sequence was predicted as efflux protein conferring antibiotic resistance or not. At tier-II, the predicted protein was divided into different prokaryotic efflux families.

The Overall schema to classify a prediction into different categories is shown in Figure 6. At tier-I prediction, the input proteins would be predicted as either an ARE or non-ARE protein. Only ARE protein will move to tier-II where they would be classified into one of the four efflux families. Depending on the tier of prediction, the meaning of TP, TN, FP and FN also changes accordingly. For example, at tier-I TP and TN showed the number of proteins, which were actually ARE and non-ARE and also predicted as ARE and non-ARE proteins, respectively. Similarly, FP and FN were actually non-ARE and ARE proteins but they were falsely predicted as ARE and non-ARE proteins, respectively. At tier-II prediction, for a protein of efflux protein family ‘X’, if it is correctly predicted to class ‘X’ it would be a TP prediction, if it would be falsely predicted to class non-‘X’ it would be a FN prediction. Similarly, if a non-‘X’ would be predicted as non-‘X’ and ‘X’ it was an example of TN and FP predictions, respectively.

## Conclusion

To conclude, using machine learning we have developed a novel two tier *in-silico* tool for prediction and classification of efflux proteins capable of efflux out antibiotics from the cell of a bacterial cell. The proposed tool first predicts the efflux proteins that may have capability to efflux out antibiotics and then classifies the predicted protein into one of the four classes of efflux proteins. We also developed a web-server ‘BacEffluxPred’ and its standalone version. We anticipate that BacEffluxPred would be helpful to the scientific community in prediction and characterization of microbial efflux proteins, which are involved in antibiotic resistance.

## Supplementary information

Supplementary Information - Tier I Dataset.

Supplementary Information- Tier II Dataset.

## Data Availability

The tool and its dataset (tier-I and tier-II) are freely accessible without any restriction at download page of the web-server http://proteininformatics.org/mkumar/baceffluxpred/downloads.html.
