# Supplementary material for: BacEffluxPred: A two-tier system to predict and categorize bacterial efflux mediated antibiotic resistance proteins
Source: Sci Rep. 2020 Jun 9;10:9287. doi: 10.1038/s41598-020-65981-3 (PMC7283322; doi:10.1038/s41598-020-65981-3)
Supplement: Supplementary file 1 — Supplementary Information - Tier I Dataset. [file 41598_2020_65981_MOESM1_ESM.zip › BacEffluxPred_Supplementary_materials_Tier-I_Dataset.docx]

**Supplementary Information**

**BacEffluxPred: A two-tier system to predict and categorize bacterial efflux mediated antibiotic resistance proteins**

Deeksha Pandey^1,2^, Bandana Kumari^1,3^, Neelja Singhal^1,4^ & Manish Kumar^*^

^1^Department of Biophysics, University of Delhi South Campus, New Delhi, India

^2^Email: deeksha.pandey.biophysics@south.du.ac.in
^3^Email: vandanachaurasia.1@gmail.com

^4^Email: neelja30@gmail.com

*Correspondence to: Manish Kumar, Department of Biophysics, University of Delhi South Campus, New Delhi, India – 110021 Telephone Number: +91-11-24157263

E-mail: manish@south.du.ac.in

**Tier-I dataset**

**The dataset which was used to develop tier-I SVM models.**

1. **Bacterial antibiotic resistance efflux (ARE) : 210 protein sequences**

>1071078.3.peg.1866_ABC

MVDLLYTELLKLKRSQMFLVSILGAAAAPFICFISSLAKKAKYPDVPIRFSETFSDTNLYIVLLIGVPLYGVITSYLFNREYAESTLKNLLTIPVSRISLIISKLVLLFIWIMLLTLIAWVLTLLFGLIGQFEGLSSAVLIEELKQFMTGGALLFFLLSPIIFVTLLFKNYVPTIIFTIIISMVSIMVYGTEYSALFPWSAVWVIASGTFFPEYPPAFSFISVIATTVLGLAATIVYFKKIDIH

>1121863.3.peg.2626_ABC

MPSRSRRLNCSLFCFLWVAAFMELLRLVWRQYRLPFVLVLALSLASAALGIGLIAFINQRLIATVDLSLAVLPAFLGLLLLLMAVTLASQLALTMLGHHFVYRLRSEFIKRIMDTPVEQVEKLGSATLLAGLTSDVRNITVAFVRLPELVQGIILTIGSAVYLGWLSSKMLLVTAVWIAITLWIGYLLVQRVYKHIATLREVEESLYNDFQTLLEGRKELALNRERAEYIFDQVYKPDAQSYRQHIIRADTFHLSAVNWSNIMMLGVIGLVFWMANGLGWADTNVAATYSLALLFLRTPLLSAMGALPTLLTAQVAFNKLKQFQLADYEPAFKRPQKFADWQTLELRDVTFKYSDGSFGVGPLNLTIKRGELLFLIGGNGSGKSTLAMLLTGLYEPASGDILLDGKVIATSEMEAYRQHFSAVFTDVWLFDKLLGPQGEEADPALVDAWLNRLKMAGKLELDNGKILNLKLSKGQKKRVALLLALAEDRDIILLDEWAADQDPHFRREFYQVLLPLMQQMGKTIFAISHDDHYFIHADRLLEMRQGHLSELTGDERALASRDAVARTGS

>1156433.3.peg.1263_ABC

MKVLKQLLSRITLYPTVFLVGFICLLLATIFSELSPFILQKMIDGPLTALTHSGEQGQLLQMGGFYLLVLSIGQLISYLGNRILLHGSNQVTANLRDQAFQVMQGLPISYFDDKPAGKIATRIVNDTETLRTQFYNSCMVLIIFLVRFLFVLGILFYLSPMMGLLLCLVFPIFYGIQYLYKVMTDQPMKDFFDARSEVNTQVNELLHGASMIQLYGQEPHVIEEFEATTQKMLWANDRILLADSIASWTLTELLKYLVIAGILTIAGMSYLKGNIGVTAGFLFININYVMNLFELMAALSRQFPNIRRSLETGSRVLAFLDQPLEADGVLELKIEKAQVVFDDVQFAYEEGKPVLQDIAFQASPGETIALVGHTGSGKSSIMNLLYRFYDPQDGAILIDGQDIRQVSRESLRSHMGIVLQDPYLFTGTIASNVAMSQDHIDRDAIKDALKKVGAWPFVERLEKGIDHPVVEKGSAFSSGERQLISFARTLYMNPQILILDEATSHIDTETEEIIQKAMAVLQKGRTTFIIAHRLSTIQDADKILVLSEGRIVERGQHADLIAHGGIYAQMHAIQQTVE

>1156433.3.peg.1264_ABC

MIGAIWEYIRERKWRYISIAVVLILYDYTLLIPTQVIQRLVDHLSQQTLTQSNFVWDMVLLVGSAILNYLTAFYWQLRLFQSSIHFKSTLQEQAFRKLVAMRRPFFEKFRSGDLLTRFTTDVDGMADMAGYGMMIILYGGGLFTFIILAMFFLSWQLTLICFIPMIFLVVSTYFLSKKQEDYIEQNREAVAQLNDEVLESIEGIRVMRAYSRRDQQVKQFQTKTASLAKTGDKIASIQYSFGPLALLFIGVSTVLLLVFGGQSLASGQLSLGKLLALQLYLVFLVEPMWMLSDFILVYQTGQMSFKKLKEVIDETDDLEPDGPHFLEQIDSVEFKDYSFRYPGAERESLSGIDWTVQKGQTVGIVGRTGSGKTSLVRQFLRQYPVGEGEFLVNQQPIVAYNRRSIEDKIGYVSQEHILFSKSIRDNIALGKNGASEEDLVEAVAQAAFADDLERMSQGMDTMIGEKGVSVSGGQKQRISLARAFLRDADFLLLDDSLSAVDAKTEQAIIDSIQTERKGKTTIIVSHRLSAVHQADWIIVLDQGQIVEEGRASDLLAQEGWYYEQYQRQQKQEGE

>1158608.3.peg.145_ABC

MKLMWRYTMRYKKLLFLDFICVFGFILIELGLPTILARMIDVGIKNNDYDYVKQQGLLMIVITVIGVAMNIMLGYFGARMTTNIVRDIRDDLFEKVQTFSHREYETIGVSSLITRTTNDAYQIMLFMGNILRIGFMTPMMFFVSLYMVMRTSPSLGWFVLGALPFLLAAVVLIAKVSEPLSNKQQKNLDGINGILRENLSGLRVIRAFVNEKFEESRFSKVNEDYTKSSKSLFRLMAAAQPGFFFLFNIVMVLIIWNGALQIDQGSLLVGDLIAFIEYIFHALFSFMLFASVFMMYPRAAVSARRIQEAFDMEPVIRENEAGITETKTKGYLEFKNVTFAYPGHSESPVIRNVSFTASPGETVAFIGSTGSGKSTLIQLIPRFYDVSEGEVLLDGIDVRDYKLSALRNKIGYIPQKALLFTGTIAENMRYGKEDATIEEMELAADIAQATEFISQKPDGYDELLSEGGTNFSGGQKQRLAIARAVIRRPEVYIFDDSFSALDYQTDANLRARLKKETTESTVLIVAQRVGTIMHADKIVVLNEGDVVGIGTHRELLENCPIYYDIAASQLSEEELA

>1232427.4.peg.1332_ABC

MVRDMVGAYPGVLVLHILSFLIGSGIAAFAPVVVGMIVDGLVGEEKFNAWWLFGVLVGIFIIQFAGEATGDGLAAASVRRVTHNAQQHLSSGVLRRGAGAMSPGTVLNTIDADANTIGRYRELLSFPLMAIGYAAGAIVAMWTVSPWVSLAIPVSALVIALFAAWTAGPVTRVSLKRRAAEADVASLATDTSQGLRTVKGLGAGGTVAHRFHTETAKAKRLMLTHLRVEVWLGFARLCVAWLCNLGIVGLAAWMTLRGEITPGQLTSVALLVPPALNMAGFAFGDLASGWGRAVASGQRIQQLHHAGDDTAGPEPTDTPVPGAGLWILEPAERSYATAVAWAQRADVLFPPHTVNVFEGTIADNVNPRGDVPEDAVKQALAAAHCQDILRRLGGIGENGELPDAPLGEAGLNLSGGQRQRVALARALAADPEVLILDDPTTGLDSVTQADVVEAVAVLRADKTTVVITGNSAWQHAGTALEVA

>1234876.3.peg.1456_ABC

MENTKSTRKMSDTTRAIRFFYLYLKRYKLQFAVIMIFIVAATWLQVIAPSLLGDAITNLGVYVKDFFTHQHAGQSQDALQQIAQQLSQQMHQTVDWHNVPEVVKTLPQAAQDQITAHLPKGTTLETLKTVATSHAASTSTFMKGMWQLLAVYVATGVSMLIYTLLFSRIVAHSTNRMRKGLFGKLERLTISYFDRHQDGDILARFTSDLDNIQNTLNQALVSVISNAAVFVGVIIQIFLKDVTFAWLTVAASPVAILSAVIIIRQSKKATDKQQEEVSQLNAYMDEKISGQKAIIVEGLQEDSIDGFLKHNENVKKRTFAAQAWSGMIFPLMNGFQLLSIAIVIFGGTAYVLNNDSMSITTGLGLLVAFVQYVQSYYNPIMQISSNFGQLQLAITGATRLNVMFDEPEEVRPENGKKFDTIKDGIQIENLDFEYLPGKPVLKKVNIDVKKGQMVALVGPTGSGKTTVMNLMNRFYDVNGGAIKFDGTDIREFDLDSLRSNVGIVLQESVLFDGTIADNIKFGKPNATQEEIETVAKTTHIHEFIESLPDKYETHVSDDESVFSVGQKQQISIARTILTNPELLILDEATSNVDTVTEEQIQWAMEAAIAGRTSFVIAHRLKTILNADKIVVLKDGEVIEEGNHHELVAQGGFYSELYHNQFVFE

>1234876.3.peg.1457_ABC

MIFKSIMKHKWVALFSIFSTFVYAGVQLYQPQIMKRIMTVMSSTTYSRHEMADKVSGYGVELLIVAGIGILFAIFSTLSAARIAQEIGADVREATYKKINTFSYENVEKFNAGNLVVRMTNDVTQVQNLMMMVFQILMRIPVLLIGAVILSITTLPKLWWITVLLIVLILVVTAVLMGRMGPHFMAFQKLMDRINAIAKQNLRGARVVKSFVQEKNQIKEFDETSDELYDHNWAVGKLFSAMIPLFTVIAQGAIWLAIYFVSTFVTDSPTVAQDSIGGIATFMTYMGMIMFAIIMGGMISMFASRGMVSIGRINEVLKTDPAMKFDENAKDEELSGSVKFDHVSFSYPNDEEPTLKDISFEVEAGQMVGIVGATGAGKSTLAQLIPRLFDPTEGTVSVGGKDLKTVSRGTLKRNISIVLQKAILFSGTIAGNIKQGKSDATDEEMTRAAQIAQAAEFITTKDGQYESEVEERGNNFSGGQKQRLSITRGVVKNPNVLILDDSTSALDAKSEKLVQEALNKELKETTTIIIAQKISSVVHADNILVLDQGKLVGQGTHQELVAENKIYQEIYDTQKAQED

>1413510.3.peg.681_ABC

MLFLFEEKALEVEHKVLIPELTFSIEDHEHLAIVGVNGVGKSTLLKVIHQDQTVDSAMMEQDLTPYNDWTVMDYIIESYPEIAKVRSQLNHTDMINKYIELDGYLIEGEIVTEAKKLGIKEEQLEQKISTLSGGEQTKVSFLKVKMSKASLLLIDEPTNHMDLEMKEWLTKAFKQEQRAILFVSHDRTFLNETPDAILELSPDGAKKYIGKYDKYKQQKDIEHETLKLQYEKQQKEQAAIEETIKKYKAWYQKAEQSASVRNPYQQKQLSKLAKRFKSKEQQLNRKLDQEHIANPNKKEKAFSIQHHDFKSHYLVQFNHVSFAYDNRKIFEDVSFYIKRNQNVIIEGRNGTGKSTLIKLILGELEPTKGNITVHPELEIGYFSQDFENLNMHNTVLDEILEIPEMNEADARTILASFYFDKDRINDVVETLSMGEKCRLQFVKLYFSNPHIMILDEPTNYFDIGMQEKIIQLIQSFQGSVLIVSHDDYFKSQIKDQIWTIKNHQMTHENVQVKDPINTESMKHQLKELEQYTEERNRETEF

>1428628.3.peg.4541_ABC

MRPDSQITWTPPADAKEQPRQVRRILGLFRPYRGRLAIVGLLVGAASLVSVATPFLLKATLDTAIPQGRTGLLSLLALGMILSAVLNSVFGVLQTLISTTVGQRVMHDLRTAVYGRLQRMSLAFFTRTRTGEVQSRIANDIGGMQATVTSTATSLVSNLTSVVATVVAMLALDWRLTVVSLVLLPAFVWISRRVGNERKKITTQRQKQMAAMAATVTESLSVSGILLGRTMGRADSLTESFADESERLVDLEVRSNMAGRWRMAVITIVMAAMPAVIYWTAGMALQMGGPKVSIGTIVAFVSLQQGLFRPAVSLLATGVQIQSSLALFQRIFEYLDLPIDITERQDPVHLDRVKGEVRFEDVAFRYDDKSGPILDGIDITVPAGSSLAVVGPTGAGKSTLGYLVPRLYDVTGGRVTLDGVDVRDLDFDTLARAVGVVSQETYLFHASVADNLRFAKPDATDEELHAAAKAAQIHDHISALPDGYDTVVGERGHRFSGGEKQRLAIARTILRDPPVLILDEATSALDTRTEHAVQEAIDALSANRTTLTIAHRLSTIRDADQIVVLDGGRTAERGTHEELLELDGRYAELVRRDARQQPQAEARPEAQARTGPEPRVDSQPSRRGGSRPSPHGGGRPSPRGDSRPGPRVDSGLEPTS

>1460652.3.peg.5862_ABC

MSMIQVQDLTFSYPSSFDNIFEGVNFQIDTDWKLGFIGRNGRGKTTFFNLLLGNYEYSGKIISSVQFNYFPYPVSDKNKYTHEIFEEICPQAEDWEFLREISYLKVDAEVMYRPFKTLSNGEQTKVLLAALFLTEGQFLLIDEPTNHLDTGARKIVSDYLRKKKGFILISHDRIFLDGCVDHILSLNRANIEVQKGNYSSWKLNFDRQQEHEEATNQRLQKDIGRLKQASKRSAGWSNQVEASKNGTRNSGSKVDKGFVGHKAAKMMKRAKNLESRQEKAIEEKSKLLKNVEKTESLKLAPLEFQSNELIVLTDVSIKYDDQIVNKPISFNVEQGDRIVLMERMEAEKVVF

>1463857.3.peg.5322_ABC

MTATSTNETGATAGAAAGTTTEATHAAGGAAVERGAPPQGSAPPGGPGTRAGAEGRIGLRAHLRHIGALARRNALQIKQDPESMFDVLLMPIVFTVLFVYVFGGSVGASLGGDRHDYLNYVVPGLMAMMGMNIAMAVGTGMNDDFRKGVMDRFRTMPIARSSVLIAKIVVEVGRMIVATAILLGMGFALGMTVQTSVLGLLAAVGLSLLFGAALMWIFILLGLTMKTAQAVQGVAMIVLMPLQFGSSIFAPTKTMPGWLQAFTDYNPLSNLADAARGLVNGGPVAHSAWMTLAWAAGITLVMAPLAVRKFRDKT

>1638.4.peg.885_ABC

MSIIEINQLKIEVADRVLVEIPHLLVNQKARIGIIGQNGLGKTTLIEVIAGVQEPAVGKVTIQGRLAYIKQLPTDKSTKSGGEKTRKAIQQAMRQNPSVLLADEPTSNLDVESVKHLERQWKDWHGSLIIISHDRAFLNSLCTEIWEIKDQKIQVYKGNYQAYLKQRKQQENQAELAYKEFKNKKKQLEASQNYHEVEAGRIVKPGKRLNAKEASAFKAGKGTQQKKQHSTIKALDKRIERLGNVEKPHKAKPIKISTPENRIIKKGNTILTAAEATYEIAGKKLFSTTGFSIKSGDKVALIGENASGKTSFLKQILQNNSKLVCSNQAKIAYFDQELQGLDLTKTLLENMIDISVQSKQMTKEVLGSMHFKETDWHKKASLLSGGERVKLLLSMLLVSDANFLILDEPTNYLDIFAMEALETLIQNFTGTVLFVSHDRTFVSQVAEQLLVIESGKMAFYRMAFAEYEASITPSRITEEDKLILEMRMSEIAAKLMQPNLKAEDKALLEKDYQEVITKRRQFN

>226185.9.peg.2536_ABC

MSKIELKQLSFAYDNQEALLFDQANITMDTNWKLGLIGRNGRGKTTLLRLLQKQLDYQGEILHQVDFVYFPQTVAEEQQLTYYVLQEVTSFEQWKLERELTLLNVDPEVLWRPFSSLSGGEKTKVLLGLLFIEENAFPLIDEPTNHLDLAGRQQVAEYLKKKKHGFILVSHDRAFVDEVVDHILAIEKSQLTLYQGNFSIYEEQKKLRDAFELAENEKIKKEVNRLKETARKKAEWSMNREGDKYGNAKEKGSGAIFDTGAIGARAARVMKRSKHIQQRAETQLAEKEKLLKDLEYIDSLSMDYQPTHHKTLLTVEELRLGYEKNWLFAPISFSINAGEIVGITGKNGSGKSSLIQYLLDNFSGDSEGEATLAHQLTISYVRQDYEDNQGTLSEFAEKNQLDYTQFLNNLRKLGMERAVFTNRIEQMSMGQRKKVEVAKSLSQSAELYIWDEPLNYLDVFNHQQLEALILSVKPAMLVIEHDAHFMKKITDKKIVLKS

>226185.9.peg.2721_ABC

MKHAFSSMKRIGRYIKPYRVTFYLVILFTILTVAFNAALPYLTGLPTTEISRNIAAGESINFDYVIQCLIWILVVGTGYCVAQFLSGFLMTNVVQQSMRDLRRDIEEKINRLPVSYFDKNQQGNILSRVTNDVDAVSNAMQQSFINIVSAVLGIVMAVVMMFLINPLMAIFSVIMIPLSLIISRTIVKISQKYFQGMQNSLGDLNGYVQENMTGFSVLKLYGREKETLEGFKQVNHRLNGFGFKASFISGLMLPLVQMTAYGTYIGVAVLGSYYVVAGVIVVGQLQAFIQYIWQISQPMGNITQLSAALQSASASTMRIFEILDEPEEELNEQDVPLPEPILGSVEFENVSFSYDPEKPLIRNLNFKVDAGQMVAIVGPTGAGKTTLINLLMRFYDVTEGAIKIDGIDTKKMNRSDVRSVFGMVLQDAWLYKGTIADNIRFGKLDATDYEVVDAAKTANVDHFIRTMPDGYEMEINSEGDNVSLGQKQLLTIARAVISDPKILILDEATSSVDTRLEALIQKAMDRVMEGRTSFVIAHRLSTIREADLILVMKQGEIIEKGTHHELLEQGGFYEKLYNSQFAEEGDYEE

>29379.8.peg.680_ABC

MSFMIRRYLRFVKPYKWRIIITIIVGIIKFGIPMLIPLLIKYVIDDVINNGEIDTQQKMLRLAIALGIAIFIFVVIRPPIEFIRQYLAQWTSNKILYDIRRHLYNHLQALSARFYANNQAGQVISRVINDVEQTKDFILTGLMNIWLDCVTIVLALTIMFFLDVKLTLAAMFIFPFYIITVYFFFGRLRKLTRKRSQALAEVQGFLTERVQGMSVVKSFAIEENEAENFDAHNQHFLDRAFKHTRWNAYAFAAVNTVTDIGPLIVIGIGGFLAINGSITVGTLAAFVGYLEQLFSPLRRLVSSFTTLTQSFASMDRVFQLFDEDYDIKNKKGAQPIAIQQGDISLDHVYFKYNEDEDMILRDINLDVHQGETVAFVGMSGGGKSTLINLIPRFYDTTKGSITIDQHPIKDFLTGSLRSQIGLVQQDNILFSDTIRENILLGKPDATDEEIVQAAKMANAYDFIQELPHGFETEVGENGVKLSGGQKQRISIARIFLNNPPIIILDEATSALDLESEAIIQDALNVLSEDRTTLIVAHRLSTITHADKIVVMENGQIVETGTHQELLDRNGQYAHLFNIQNL

>315749.8.peg.286_ABC

MYVKDHRKWVFTIHTIIKTTNLTKVYGKQKSVDHLNINVNKGEIYGFIGRNGAGKTTTIRMLLGLIKPTNGKIEIFGEDFTKNQKDILRRIGSIVEVPGFYENLTAKENLLINAKIIGVHKKNAIEEALEIVGLQHETKKLVGKYSLGMKQRLGIARALLHYPELLILDEPTNGLDPIGIKEMRKLIKTLAQERNITIFISSHILSEVEQLVDHMGIIHKGKLLEETSLDALRKMNRKYLEFQVNNDNKAALLLEKQFHIFDYEVHDEGNIRVYSHFGQQGQINKMFVQNDIEVLKIIMSEDRLEDYFTKLVGGGTIG

>33009.3.peg.2745_ABC

MPTAQCALHDITKRYDDRVVFDRIGFSIAPGEKVGVIGDNGSGKSTLLKLLAGRERPDDGTLTVVAPDGVGHLAQTLELPLHATVQDAVDLALSDLRELEAAMRRAEAELAEHDTDGPGTELSATLRHYADLVERYQARGGYEADVRVEVALHGLGLPGLDRARELGTLSGGERSRLALAATLASAPELLLLDEPTNDLDDRAVEWLEEHLRGHRGTVVAVTHDRVFLDRLTTTVLEVDSGRVTRYGNGYEGYLTAKAVERERRLREYGEWRAELVRNQGLIASNVARMDGIPRKAPLSVFGHGAYRRRGRDHGAMVRIRNAKQRVAQLTENPVPAPADPLSFTARIDTSGPGAGEAEEAEEAVAELTGVRVADRLAVDSLRIRPGERLLVTGPNGAGKTTLLRVLSGELEPDGGSVRAGCRVGHLRQDETPWPPEATVLRAFAHGRDGYLDDHAEKLLSLGLFSPSDLRRRVGDLSYGQRRRIEIARLVSDPMDLLLLDEPTNHLTPVLVEELEQALVDYRGAVVVVTHDRRMRSRFTGARLTMEHGRVTGFRAA

>333849.13.peg.130_ABC

MENLAVNITNLQVSFGNQLELSIDSLRVYQQDRIGIIGENGVGKSTLLKLIAGELFPDHGKIQTEITFNYLPQLTYLAEAKDLNLELASHFQLRLEETSERKWSGGEERKIELIRLLSSYEQGMLLDEPTTHLDRKSIDRLIEELRYYYGTLVFVSHDRYFLDELASKIWEVKDGEIREFSGNYSAYLTQKELEKKTQLREAESIMKEKKRLEKSIQEKKKQAEKLEKVSSKKKKQQIRPDRLSSSKQKDSVQKAIQKNAKTLERRLQKIGETTKPQQMKQIRFPVPKSLELHSRYPIMGQNVQLERSGRTLLVNGDFQFSLGKKIAIVGENGSGKTTLLEHIRKQGEGILLSPKVSFQVYQQKGYQMTSEESIIRFVMRQTEFSESLVRSLLNHLGFAQETLTKPLCTLSGGEATRLTIALLFTKPSNVLLLDEPTNFIDMATIEALEKLMQIYPGTILFTSHDSYFVERTADEVYEIKGQKIKKVLTRNF

>411235.3.peg.357_ABC

MELRTNPKITPAVQVRRLTKHHGETTALDGVDLDVAEGTVMGVLGPNGAGKTTLVRILSTLVRPDAGARRRGGLRRRAPALPAAPGHRPHRAERLRRRAALRLRNLYLIGRLLDLNARGARPPGPSRRVRRPRAQRRGRGADGPPGSAGRPAGRGRTGLAPDLLDQRPGRCGSPLAQASAAARARSRAPERATGRWSSSTTSVLPRIPKRIPTF

>66692.6.peg.3722_ABC

MNTGQLVMRNLRKNSKTYGLYIFSLTFSAALYFAFVTLQYDPALDEAAASVKGAAAIQSASVLLIAIIAIFLLYANRLFLKRRSKEIGLFQLVGMRKGRIFWVLSGENVLVYFGALAVGIFIGFYLSKLAMVSLYRIIGVETAAKLHFSGAALTQTLLVFAAICVLMMGFTYVYIRKQTILSLFHIKGKTEMVAQGLRAFEIAFGVLGIVLIGFGYWLSSKLFEGQFVTQNELFLAMTTILAACVFGTWLFYKGTVSFVAKVIRRKKDGYLNIREVMSLSALMFRMKSNAVLLTVITTVSALAIGLLSLSYITYYSAEKSAKQWVPTDFAFTSVEDAEAFKNKLDQTGVDYQERTTEFIQGNVNVEGIINSSTEMMTGTAQEMAITSAAYMTDVDISPDEAVLTGSNDLLQRFVTFKEEGEIVIELGEETLSQHYRGLKKEFVLPSFYKVAGGMPTVVVNEETFVSLKKQQEAETSYGIDIIKESEVVAANSAYQEMDFQEQSESQWAMATNQKAHMGLYMFIVAFLGLTFLITSGCILYFKQVDETEGEKHNYTILRKLGFNRRQLEKGSYGKQLFAFGIPLLLGLSHSYFAVQSGWFFFGGELWTPMLLVMAVYTVLYSIFALLSVSHTKKVIKESL

>66692.6.peg.3723_ABC

MVVLEAANICKQYGNKWNKQEVLKGLDLTIEKGEFVSIMGASGSGKTTFLNVLSSIDQVTSGTIIIDGKDITTMKERELAWFRQRHLGFVFQEYHLLETLTVKENILLPLSVMGAGKKEADEAFRSVAAELGIYELKDKYPNELSGGQKQRTSAARAFIHQPSMIFADEPTGALDSKSATDLLNKLTEFNQKYEATILMVTHDAAAASFSRRVVFIKDGQMFTEVMQQDRTREDHYEEIMKTQAILGGIKA

>73044.3.peg.968_ABC

MDGLTDRPHPQFGHDPFGGVVAHLSDADDPLQPPLLEPEPYGGRGGLGGQPLPPVGASQPPADLDRRQYLRQEAGHREAGEPGQLAGGPDLHGEQTEALRLPLALPGPDPTAGLLLVTDAAVTDPPHDHGIGVDGSHRRDVFLAPATQDQAGSLKRDHPVILPCCRPSVGGLSVAPGTVLLMTRIDENPGGGRSAVSVRGMVKHYGETKALDGVDLEVREGTVMGVLGPNGAGKTTLVRILSTLITPDAGEALVAGYDVVRQPRQLRRVIGLTGQYASVDEKLPGWENLYMIGRLLDLSRKDARRRADELLERFSLTEAAKRPASTYSGGMRRRLDLAASMIGRPQVLFLDEPTTGLDPRTRNEVWDEVKAMVGEGVTVLLTTQYMEEAEQLASELTVVDRGKVIAGGRIEELKAKVGGRTLRVRPIDPLQLEPLATTLDELGITGLATTTVDRQTGTLLVPILSDEQLTAVVGAVTARGITISSIVTELPSLDEVFLSLTGHRASAPQDATPADSREEVAV

>768710.3.peg.1877_ABC

MELIVKAKDIRLEYTGRDVLDIDELELYDYDRIGLVGANGAGKSSLLKVLLGELTLPGCKINRLGRLAYIPQLEEAILEEVKDFALIGKLGVSQIEVQTMSGGEETRLKIAQALSEQVHGILADEPTSHLDREGMDFLIGQLNYFSGALLVISHDRYFLDEVVDKIWELNDGKITEYWGNYSDYLRHKEEERQSQAARYEQFVAERNRLERAAEEKRKQARKMDRKAKGAAKKNSSESGGRLGHQKTMGSKQKTLFNAAKSMEHRIAALGEAEAPENIRTIRFRQSKTLELHNPYPIIGTEINKGFGDKVLLEKASFSIPLGAKAALTGGNGSGKTTLIQMILNREEGISISPKAEIGYFAQNGYKYNRNQEVMEFMMEDCDYNISEIRSVLASMGFVQNDIGKRLAVLSGGEMIKLQLAKMLMGRYNILLMDEPSNFLDLPGLEALEVLMKGYAGTIVFITHDQWLLDNVADMIYEIKSKKLNLIR

>904314.5.peg.208_ABC

MKIMLEGLHIKHYVQDRLLLNINRLKIYQNDRIGLVGKNGNGKTTLLHILYKKIVPEEGIVKQFSHCELIPQLKLIESTKSGGKVTLNYIRQALDKNPELLLVDEPTTNLDNNYIEKLEQDLKNWHGAFIIVSHDRAFLDNLCTTIWEIEEGRITEYKGNYSNYVEQKELERHREELEYEKYEKEKKRLEKAINIKEQKAQRATKKPKNLSLSEGKIKGAKPYFAGKQKKLRKTVKSLETRLEKLESVEKRNELPPLKMDLVNLESVKNRTIIRGEDVSGTIEGRVLWKAKSFSIRGGDKMAIIGSNGKGKTTFIKKIVHENHGISLSPSVKIGYFSQKIDTLELDKSILENVQSSSQQNETLIRTILARMHFFRDDVYKPINVLSGGERVKVALTKVFLSEVNTLILDEPTNFLDMEAIEAFESLLKEYNGSIIFVSHDRKFIEKVATRIMTIDNKEIKIFDGTYEQFKQAEKPTRNIKEDKKLLLETKITEVLSRLSIEPSEELEQEFQNLINEKRNLDK

>P0A9U1_ABC

MNDAVITLNGLEKRFPGMDKPAVAPLDCTIHAGYVTGLVGPDGAGKTTLMRMLAGLLKPDSGSATVIGFDPIKNDGALHAVLGYMPQKFGLYEDLTVMENLNLYADLRSVTGEARKQTFARLLEFTSLGPFTGRLAGKLSGGMKQKLGLACTLVGEPKVLLLDEPGVGVDPISRRELWQMVHELAGEGMLILWSTSYLDEAEQCRDVLLMNEGELLYQGEPKALTQTMAGRSFLMTSPHEGNRKLLQRALKLPQVSDGMIQGKSVRLILKKEATPDDIRHADGMPEININETTPRFEDAFIDLLGGAGTSESPLGAILHTVEGTPGETVIEAKELTKKFGDFAATDHVNFAVKRGEIFGLLGPNGAGKSTTFKMMCGLLVPTSGQALVLGMDLKESSGKARQHLGYMAQKFSLYGNLTVEQNLRFFSGVYGLRGRAQNEKISRMSEAFGLKSIASHATDELPLGFKQRLALACSLMHEPDILFLDEPTSGVDPLTRREFWLHINSMVEKGVTVMVTTHFMDEAEYCDRIGLVYRGKLIASGTPDDLKAQSANDEQPDPTMEQAFIQLIHDWDKEHSNE

>P0AFP9_ABC

MFHRLWTLIRKELQSLLREPQTRAILILPVLIQVILFPFAATLEVTNATIAIYDEDNGEHSVELTQRFARASAFTHVLLLKSPQEIRPTIDTQKALLLVRFPADFSRKLDTFQTAPLQLILDGRNSNSAQIAANYLQQIVKNYQQELLEGKPKPNNSELVVRNWYNPNLDYKWFVVPSLIAMITTIGVMIVTSLSVAREREQGTLDQLLVSPLTTWQIFIGKAVPALIVATFQATIVLAIGIWAYQIPFAGSLALFYFTMVIYGLSLVGFGLLISSLCSTQQQAFIGVFVFMMPAILLSGYVSPVENMPVWLQNLTWINPIRHFTDITKQIYLKDASLDIVWNSLWPLLVITATTGSAAYAMFRRKVM

>P0AFQ2_ABC

MSNPILSWRRVRALCVKETRQIVRDPSSWLIAVVIPLLLLFIFGYGINLDSSKLRVGILLEQRSEAALDFTHTMTGSPYIDATISDNRQELIAKMQAGKIRGLVVIPVDFAEQMERANATAPIQVITDGSEPNTANFVQGYVEGIWQIWQMQRAEDNGQTFEPLIDVQTRYWFNPAAISQHFIIPGAVTIIMTVIGAILTSLVVAREWERGTMEALLSTEITRTELLLCKLIPYYFLGMLAMLLCMLVSVFILGVPYRGSLLILFFISSLFLLSTLGMGLLISTITRNQFNAAQVALNAAFLPSIMLSGFIFQIDSMPAVIRAVTYIIPARYFVSTLQSLFLAGNIPVVLVVNVLFLIASAVMFIGLTWLKTKRRLD

>A0LM36_ABC

MDLIELQDIRKTYRLGEIDVPVLRGISLKVSPGDFVALMGTSGSGKTTLMNILGCLDRPTSGHYRFDGQDVVDLTPDQRAALRNRKIGFVFQNFNLLPRMSAVENVMMPLSYAGGGVSDQNGRERAGALLTRMGLGEHLDNEPSQLSGGQQQRVAIARALINNPSLLFADEPTGNLDSATSEEVLRVFQRLNEEEGVTIILVTHDPSVAQCARRIVRIRDGVIEPESGAVGDMPQVSKAAPAQSKPVHSAMRRGDLDKFRRSLHTALSSLRRNVLRAALTTLGIIIGVAAVIAMMEIGRGSSTAIQRTIASMGAHTLALLPGTAASGGVSFGGGSVMTMTPQDSEAIVNECPAVLAAAPIVRARTQVVHGSRNWVPAGIYGTTPTFLEIREWPLAEGDVFTERDVRNASKVCVLGQRLVDELFQGENPIGLEVRIKNVAFKVIGVLSPKGANMMGMDQDDLLLAPWTAIKYRVTGSSLANVNQSAASTSSASITDQVNSLSNLYPTEKVVLYPEISTTQAFDTPLPVRFTNVDQILVGIRSTSGTRAAIRQIGEVLRERHRLRPGEPDDFSVRDMTEMTKTLASTATMMTKLLLAVALISLIVGGVGIMNIMMVSVTERTREIGLRMAVGARAKNILQQFLFEAVLLCFLGGAVGILVGRGISHLVTVLLNWPTELSLDAILAAVGVSATVGIVFGYYPAWKASRLDPIVALRYE

>P75830_ABC

MKKRKTVKKRYVIALVIVIAGLITLWRILNAPVPTYQTLIVRPGDLQQSVLATGKLDALRKVDVGAQVSGQLKTLSVAIGDKVKKDQLLGVIDPEQAENQIKEVEATLMELRAQRQQAEAELKLARVTYSRQQRLAQTKAVSQQDLDTAATEMAVKQAQIGTIDAQIKRNQASLDTAKTNLDYTRIVAPMAGEVTQITTLQGQTVIAAQQAPNILTLADMSAMLVKAQVSEADVIHLKPGQKAWFTVLGDPLTRYEGQIKDVLPTPEKVNDAIFYYARFEVPNPNGLLRLDMTAQVHIQLTDVKNVLTIPLSALGDPVGDNRYKVKLLRNGETREREVTIGARNDTDVEIVKGLEAGDEVVIGEAKPGAAQ

>P9WG20_ABC

MITTTSQEIELAPTRLPGSQNAARLFVAQTLLQTNRLLTRWARDYITVIGAIVLPILFMVVLNIVLGNLAYVVTHDSGLYSIVPLIALGAAITGSTFVAIDLMRERSFGLLARLWVLPVHRASGLISRILANAIRTLVTTLVMLGTGVVLGFRFRQGLIPSLMWISVPVILGIAIAAMVTTVALYTAQTVVVEGVELVQAIAIFFSTGLVPLNSYPGWIQPFVAHQPVSYAIAAMRGFAMGGPVLSPMIGMLVWTAGICVVCAVPLAIGYRRASTH

>P9WG22_ABC

MSGPAIDASPALTFNQSSASIQQRRLSTGRQMWVLYRRFAAPSLLNGEVLTTVGAPIIFMVGFYIPFAIPWNQFVGGASSGVASNLGQYITPLVTLQAVSFAAIGSGFRAATDSLLGVNRRFQSMPMAPLTPLLARVWVAVDRCFTGLVISLVCGYVIGFRFHRGALYIVGFCLLVIAIGAVLSFAADLVGTVTRNPDAMLPLLSLPILIFGLLSIGLMPLKLFPHWIHPFVRNQPISQFVAALRALAGDTTKTASQVSWPVMAPTLTWLFAFVVILALSSTIVLARRP

>P9WQL7_ABC

MTALNRAVASARVGTEVIRVRGLTFRYPKAAEPAVRGMEFTVGRGEIFGLLGPSGAGKSTTQKLLIGLLRDHGGQATVWDKEPAEWGPDYYERIGVSFELPNHYQKLTGYENLRFFASLYAGATADPMQLLAAVGLADDAHTLVGKYSKGMQMRLPFARSLINDPELLFLDEPTSGLDPVNARKIKDIIVDLKARGRTIFLTTHDMATADELCDRVAFVVDGRIVALDSPTELKIARSRRRVRVEYRGDGGGLETAEFGMDGLADDPAFHSVLRNHHVETIHSREASLDDVFVEVTGRQLT

>Q1C5W7_ABC

MTGPQQGKILLRLENVSREFITGEQTVRVLNNINLTLHSGEMVAIVGTSGSGKSTLMNILGCLDKPSAGEYWVAGRIPQYLGSDALAELRREHFGFIFQRYHLLNDLSARENVEIPAIYAGIDREERRKRAVNLLSRIGLAERLDYRPSQLSGGQQQRVSIARALMNGGDVILADEPTGALDTHSGNEVLNILKDLHQQGHTVVIVTHDMSIAEHAQRIIELKDGEIIADRPRDHAQEKPKMVDIPSVIDIPSMDEKISTGAQQETEIARKPLLTRWKVQYDRLHEAFKMAILAMAAQRLRTALTMLGIIIGIASVVSVVALGKGSQQQVLANINAMGTSTLEIFPGKDFGDMRSAAIHTLRDTDADVLAQQGYIHSVTPTVSTSVTLRYGNKSVSGTVNGVGEQYFLVRGYTIAQGMAFTRTSVNDLMQDAVIDENTRDKLFPNGETPLGKVILLGSLPCRVIGVAAKKQSGFGSDENLNVWIPYTTAMKRMLGQSYLKSITVRVNDDIDLANAEQGVIKLLSQRHGTQDFFVMNTDSIRQTIQATTSTMTLLVSMIAVISLIVGGIGVMNIMLVSVTERTKEIGVRMAVGARASDIMQQFLIEAVLVCLLGGSLGVALSLGIGLLFSLFSSNFSMVYSAASIITAFVCSSLIGVIFGFFPAKRAAEMDPIRALERE

>Q3B5J7_ABC

MAPTTPLLELVDVHRTYPVGESTVNALRGVSLEIREGEFVAIMGSSGSGKSSLLHILGLLDNPDRGEYRILGRNVNALPEDGQAGLRNHVAGFVFQQFHLLKRMSIVDNVRLPHIYSGLKGDFRHEALESLKKVGLMHRLDHTPGQLSGGEQQRVAIARALIGNPMILFADEPTGNLDSRNSLEIMKILEELHREGRTIVMVTHEDEIAAYADRVITMRDGLVVSDQRRDRVCLPAGPSVPLTLDPHAMMDASRNLSVWQDGRFIGFVQQAFQSIFANKVRSLLSVLGILVGVASVIAMMALGEGAKVSIEEELKSMGSNLISVRGGSARVRGAAQGDGAVARFTFKDVKDISRMHSLVKGAAGTVNGSGQIVFGNRNWSTTLDGVGYEYGSMRAFVPSIGRWFTRDEIRKREKVAVIGVTVARELFGNNNPIGHTVKINRINFKVIGIAPAKGFSTHRDQDDVVLVPVTTAMYRVLGRDYLNSIYVEVRSAEGIDGAKEAVSDLIVKNHRLREGDDSFNIRDMTEIQEMLSSTTRTMSMLLGAIAAISLLVGGIGIMNIMLVSVTERTREIGLRKAIGARREDIMLQFLVESVGLTLSGGIIGIIAGIGISALLAVFAGWAVKTSIVSIVLATFFSAITGIFFGLWPARKAAELRPVEALRYE

>Q7ULB5_ABC

MIQLYGLRKDYRVGDHDLPVLKGITLNIEAGEYVALMGSSGSGKTTLMNLLGSLDHPTDGDYHLAGIDVSSLTPLELAAFRSQHIGFVFQNFNLLPRATALDNVMLPTIYASDGRSRRECIEDATKLLESVGLGGRLDHMPNQLSGGERQRIAIARALMNRPKLLLADEPTGNLDTVTEQEILALFRQLNQEHGITLVVVTHDAEVAHEADRVVRMKDGLVAEDVRQRASTVDRSRLANSRAEPLREPASAWSLPATWNAIVVAVLALRRNALRTVLTMLGVIIGVASVISTMELSAGASTAIEETVASMGASMLTISPGKASSTSGRQRPIQIIPDDVVAVAEQCSAVKVAAPLVYSQVQLVRQNRRWSPNLALGTTSQYLAARNWDQLELGTPFTQEQVLDAAKVCILGKTVAHELFDSEYPIGEEIRVNGVPLRVVGVLTEKGGDVIGNDQDDIIIGPWTTFKLRVNSSTGATAQFSTFADQMPPMQLASTRRSTQREEIHQIYVEAESPDHVELARQQITQVLSRRHNVEPAGAYRINDITEVSKVVGQVVGGVSALGLVIAGVSLMVGGVGIMNIMLVSVTERTREIGLRMAVGANRSAILRQFLIEATVLCVVGGFIGIFAGHMWSVLVGRVIGWPTAMSIWAPIVAVTVAATVGIVFGYYPARTASRLNPIDALRYE

>Q7VMF9_ABC

MKQPLIELKNIERYHTNGDTLTTVLKSINLKIYSGEMVAIVGASGSGKSTLMNIIGALDVPNSGEYFIYGRNIADLSGDELAELRCRHFGFVFQRYHLLSHLTAVKNVEVPAIYAMADKILRNQRANALLCQLGLEKQLENKPAQLSGGQQQRVSIARALMNGGDIILADEPTGALDSQSSQDVLKILKDLNRKGHTVILITHDLAIAEHADRVICIQDGKIVSDTANALESMIKPQNKRTFIDDAVIEVCQQHNTEKLNRPNEKNNIDNDNKENNNGYNRNDNSFLNNPKKKLNSSILRSFNSYAESFFMAFNMMMAHKIRTFLTMLGIIIGIIAVVFVIALGEGTKKKVLDEFSSLGNNTIDIFPGKWGDESDNVHTLNMEDLELLYQQPYVQRATPVLLHIAKARYLNKTMRSLINGVSHDFFMLKNYQLVTGRLFDQNDLTLSQPVGVIDKKSAKLLFDMDDPINKIIFIDDIPLSIIGVVESSSLQQNSGKEILIWIPHSTMATRILNQSYIQQISVQLQPNVSPLKSDKAIIDLLTIKHGQKDFYTFSSSRFLQSLNKTTQALTLMISSIAFISLIVGGIGIMNIMLVSVIERTKEIGIRIAVGAKERDIRFQFLIESTMVSLIGGCIGVGCALLFGGLFSLAETSIKIQFTLSSFLIAFLCSSMIGIVFGYFPARNAAKLRPVDALSRE

>Q881Q1_ABC

MNQKVDIEALHETSINSDQPLLRLQQVSRSFMAGDREFQVLKHIDLAIHTGELVAIIGASGSGKSTLMNILGCLDHASAGSYQVNGQETRELDDDALAALRRDHFGFIFQRYHLLPHLDAVRNVEIPAIYAGTAQTTRHERAQALLTRLGLGGHLQHRPSQMSGGQQQRVSIARALMNGGQVILADEPTGALDTASGKEVMRTLLELHAAGHTVILVTHDPKVAANAERIIEVSDGEIISDRRTAQTTQPAPEAQPATPPGPAPRRLLASLGLFREAFNMAWIALISHRMRTLLTMLGIIIGITSVVSISAIGEGAKRYVLKDIQSIGSNTIDIYAGANFGDSRAKSIETLLPSDVAALNQLYYIDSATPVVGRSMLVRYRNVDVDAQLNGVSSRYFQVRNIQLAAGITFSDQDARRQAQVVVLDHNTAQRLFGPGVNPLGQVILVGKLPCTVIGVTSDHKNLFIAGNTLNLWMPYETAAGRVLGQRHLDSISVRVKDGMPSKAVEEQIKALMLQRHGTKDFFTNNLDSVMQTVQKTSRSLTLLLSLIAVISLVVGGIGVMNIMLVSVTERTREIGIRMAVGARQSDIRQQFLVEAVMVCLMGGVIGIGLSYAIGYLFTLFVQQWEMVFSLASVVTAFACSTLIGVLFGFVPARNAARLDPIEALARD

>P9WJB1_ABC

MTRLVPALRLELTLQVRQKFLHAAVFSGLIWLAVLLPMPVSLRPVAEPYVLVGDIAIIGFFFVGGTVFFEKQERTIGAIVSTPLRFWEYLAAKLTVLLAISLFVAVVVATIVHGLGYHLLPLVAGIVLGTLLMLLVGFSSSLPFASVTDWFLAAVIPLAIMLAPPVVHYSGLWPNPVLYLIPTQGPLLLLGAAFDQVSLAPWQVGYAVVYPIVCAAGLCRAAKALFGRYVVQRSGVL

>P9WJB3_ABC

MRAISSLAGPRALAAFGRNDIRGTYRDPLLVMLVIAPVIWTTGVALLTPLFTEMLARRYGFDLVGYYPLILTAFLLLTSIIVAGALAAFLVLDDVDAGTMTALRVTPVPLSVFFGYRAATVMVVTTIYVVATMSCSGILEPGLVSSLIPIGLVAGLSAVVTLLLILAVANNKIQGLAMVRALGMLIAGLPCLPWFISSNWNLAFGVLPPYWAAKAFWVASDHGTWWPYLVGGAVYNLAIVWVLFRRFRAKHA

>P0C068_ABC

MVRRTKEEAQETPAQIIEAAERAFYKRGVARTTLADIAELAGVTRGAIYWHFNNKAELVQALLDSLHETHDHLARASESEDELDPLGCMRKLLLQVFNELVLDARTRRINEILHHKCEFTDDMCEIRQQRQSAVLDCHKGITLALANAVRRGQLPGELDVERAAVAMFAYVDGLIGRWLLLPDSVDLLGDVEKWVDTGLDMLRLSPALRK

>1638.4.peg.962_MATE

MKQTDEFYLTKASIPKAIAHLSIPMMLGMSVGVIYNIINAFFIGMLHDTSMLTAVTLGLPMFTILMAIGNMFGVGGGAYISRLLGKKENSQAKQVSAFVLYGSLALGIICASILGLMINPVTHFLGADAASFLHTRNYTLALLICSPFIIANFALEQVVRAEGASKISMNGMFISTIVNLIFDPLLILYFDFNVVGAAVSVGLASAFSLVYYAWYLEKKSAYLSIHFKWFRVTKGTISNVFKIGVSELLLSLFLIVTTLILNYYSISYGEGVVAGFGVALRVVQLPEFICMGLYMGIIPLLAYNYSAGNIARFEKAIRFTAISIGLIVLVISSLVFLFRFQVMHLFSESPSVIMLGVHIMVAMLISSLFSGFTGLFTSTFQAIGKAIPATIMSVSQGIIFIPVIMLGQYYFGLVGVIWSLTATEILTCIIGVTLFTIYNIKIASSTKAKDLAV

>182217.3.peg.1605_MATE

MEKVFKRIGAYSMLKAKIDLHKDSIRKLFFYYFIPLAFSMISLSTYSMIDGMFVGKKLGKEAIAAVNIAWPIFPSLVAYELLFGFGAASIVGYFLGRGKTHRAKLVFSSVFYFVALSTFILSMALLPFSETIARLFGSNDALLAMSSRYIEIILMGAVFMVLHPLADVFVVNDKRPILAMVAMLIGSLTNVFFNYLFIFVLEVGVQGSAYATIIGHGVGFLVLMQHFLFKKGQLSFIKRFSFPAVISSAKSGVPQSTAELSFALMILIFNATIMHTAGERFLSMYGIIMYNAIIFWTTLFSISQGIQPIASFSYGARNLERVKGVFLFGLKVAFLVGVVLYGIYYFLDEFLIKMYLQANEQDLDFIQETKQAMNVYYLGYIFLGMSILCAVFFQSIQCTRSSFIITLSHTLIFIVVLLPLMSHFYGIKGIWATYPIAQFLAFLSAMGVTYYEIKKGVFTTYREQSLINGAKK

>272563.8.peg.1578_MATE

MENLFTRKFTTFEFLKFVSPAIISMIFISLYTIIDGIFVSTLVGSDALASINIVLPIINLVCGFGIMMATGGGAIVSIRMGENRQDEANSTFSFIVLFSLIVGILFTVISYFFIKEISILLGATDKLLPYCITYGKVMILCTPFYILKFIFEYFARTDGNSKFSLFLSVIGGVTNIILDYVFIKYFGMGLLGAAVATAIGIILTCVLGIIYFLSNKSTLKLRKPKTDFRLIRDTMINGSSEMVTELSTGITTFLFNVVALKLAGENGLAALTIVLYAHFLMTSVYLGFAAGVSPLISYNFGAENSDKLKETFKHSLKFIFISSLLVFIIALVFAPFIVRVFVNPDNTVFKLALQGLKIFAFAFLFVGINIFASGFFTAFHNGKISAIISFSRAFVFIIIGIIILPPMLNMTGLWLTVPFAEVITIFISILFIKKYKGRYKY

>1028805.3.peg.567_MATE

MNFRLLSQYHADIKKLIKISLPILLAQIAQNSMGLADTIMAGRVSSTDMAAISVGASIWMPLVLFGQGLLLALPPTISYLNGSGQRHRIAHQVRQGIWLVLGMSIPLGLLIYFCEIPLQYMQMESKMSDLARDYLHAMLWGLPAYLMLINFRCLNDGIAKTKPAMVITFLGLLLNIPLNYIFIYGKFGMPAFGAVGCGIATSIVNWAMCLMMMFYSYTNAQERSLKVFSQLIEMPNPKTLKKLLRLGLPIAIALCCEVALFALTSLMLSPLGSTIVASHQITLNTSSFIFMFPLSIGMATTILVGQALGAGSPQNAKKMSYAALLLGLTVTIITALITIFFRYEIASIFVTDEIVIAMAANLLLFAALYQFSDTVQMVVGGILRGYKDTKVILYITLFSYWVIGVPLGYTLGRTDWLVPHIDAKGFWIAFVVSLTFAAILLALRMKKMQAMSDNAILQRLEKLK

>Q4L8N9_MATE

MKDEQLFYFEESSIFKAMMHFSLPMMIGSLLSVIYGILNIYFIGFLDNSHMISAISLTLPIFAVLMAFGNLFGVGGGTYISRLLGAKDYIKSHYVSSFSIYSSLVLGLIIAVITLPFTDQIASILCASGETLNYTSDYLKIEFLSTPFVILFFVLEQFARAIGKPIISMIGMLSSVGINIILDPILIFGLHLDVVGAALGTAISNAIAGLFFIIYFSRKNETLSFNVKHAKPTKAMMQEIFKIGIPAFLMVVLMGVTGLVVNLFLATYGNYAIASYGISFRLVQFPELIIMGLSEGVVPLIAYNFVSNKTRMKDTIKVVIVSIAVIFAVCMTVVLVAGHSIVQLFSTDPQIVVLATFILKVTMTSLLLNGIGFLFTGMLQATGQGRGATIMAIAQGTVIIPVLFVLNSLFGLTGVIWSLLIAETVCAFLAMFIVYSLRNRLTVDKASLIEVE

>Q5MZD9_MATE

MNLRTIRAELQQFLQLAIPLAAAQVAQAAVGFVDTVMMGRLGPEPLAAGGLASALFQFILATASGVVMAVSPLVAEAQGAGKDYKIAAIARQGLWLSVLLGLPVMLIISQLARLMPVLGQSATTIALARDYWMAVLWGIIPGLGFAMLRGYVAALEQARIILPLVLFGTLVNGLGNYLLGYGQLGFPRLELTGLGLSSALGLWVMFLGLLAYTAWQPKLRRYPFWQDWRRLQPSICRQILQLGWAIAVTVAVEFGLFTIITILMGAIGVEALAAHQTVSQTIILIFMVPLGCSFAVTVRVGWWLGRQDGLGARRAGLVGVGAIALWMLLLAIPLALFPRAIVGIYVDLNNPVNAGLLNLALPMLRVASLALVLDGVQRVAMGALHGLQDTRIPLLLSLLAFWMVGVGSSAMLGFQLGWGSTGLWIGQSLGVAIAGGLFLQRFLKLTQNRTFKQRLQPQPLATHP

>Q5NYX9_MATE

MSAPILFPLSAPESSFTIAGRLFHHAWPVLVAQLLSMSMLIADTVITGRYGTLDLAAVAVGSGVYISIVMLLVGVLQAVAPTVAHHFGARRVDAIGPALQQGFWLALMLALPGIALLAFPGFLLELSSVPADVAGKTRDYLLATAFGLPAVLLYRTFYAFNNALGRPRALMMISFIVTSTHIPLAWALVHGAFGLPPLGAIGCGISTAIVNWIAFACGAGYLAHNRDYRPYRLFANWQPPRRRDLLALLKLGIPMGLSTFIEVSSFTLIALFAARLGAEAVAGHRVVANLAALIYMLPLAISIAILVLVGQAAGAREPARARATVRVGMGLTVGLVALIGVLLWVGREPVVALFSADPAVRAVALGLVFYICIYQIFDAVQTVAAHALRGYKVTFMPMLLHALCFWGIALAGGYWLAFHAPGREQSPTVAGFWEASVVATILASVLFGWLLRVVMRRPQNVQT

>Q62LW6_MATE

MSPTGFTRAAAAPPPTLSRHAADTARLAAPLAIAQLSQMAMSVTDTVLLGSLGPDALAAGGLGANLFFVVVTLLQGVLTSVSVSVAHARGAMAEDRVPHIYWTGFALSLLLAVPAFALLSFAQPLLLAFGEPAALARNVGEYAAVLRFAAPGSLIGVGLMRSFLPAIGAAKRLLWVSLAGVGVNAFLNYGLIHGAFGLPRLGFLGSATATTITIWLTAITLVALLHGRSTFRHFVAATRPRLPLMGELFGIGWPVAITYGVESTLFLATGLTVGVLGESSLAAHQIALNVASVAFMVPLAIGQAANVRVGYWAGAGAPVAARHAGFVALGLGVAFMSLSGLVLIVAPHAIVGLYLKLDDPANARTVVLATSLLGIAAVFQIVDGMQTVGSGCLRGLKDTRVPMLAATLGYWGIGFPTGYWFAFHAGLGARGLWWGLAAGLASVAMLMTWRFHRKSAALGVRADARGQA

>Q6FEY7_MATE

MAKVAGFRFELKQLFHLMWPILITQFAQAGLGLIDTIMAGHLSANDLAAIAVGVGLWMPVMLLFSAIMIATTPLVAEAKGARTPEHIPVIVRQSLWVAVSLGVIAMLILQLMPFLLPILGVPESLQPKAGLFLHAIGFGMPAVTMYAALRGYSEALGYPRPVTVISLLALVVLVPLNYIFMYGIGPVPHLGSAGCGFATAILQWLMLITLASYIYRAKAYQSTQVFSHWERINLTLVKRILKLGLPIGLAVFFEVSIFSTGAIVLSPLGDTLVAAHQIAMSVTSQLFMIPMSLAIALTIRVGMYYGEKNWVSMRLVQKLGLATATFFAMCTMSLIWFARPQIVAIYTQDPAVFDIALYLLLFAMAYQLMDAWQVGAAGCLRGMQDTKGPMWITLIAYWVVAFPVGTYLARVAKMGPAGVWLGLITGLSIACVLLLMRLYRNNHKLAQQS

>Q6NB79_MATE

MVRAMTAPGSNIAAGALAPAKSSAWRTELIETLWLAWPMALTQLGQIAMMTTDLALIGRLGDAAVAAAALAHFVLFSTFTMGLGLVSAVTPLAAQAFGARAPRQVRASLRVGLWAGVIAGVPLTLGQLYGEELLVALGQNPATSRLAGDYLDGLAWSLVPGWLFIALRGLMGAVNRPEPALWIMLTAIPINLGLAYVLIHGSFGLPRLEIFGAGLATSIVSWAMCIAAAVVCVTMRPFRKYQVFGELFRFDGELMRRLLQLGLPISGASVLEYGVFGAAALLMGKFGTTALAAHQIALQVAAIMFMVPMGISVAATVRVGHAVGRGDPPSARRAGFAAIGLGFVFMAAMTLLVALTRHQIPQLFLGDSDTSIETATLTAALLIVGASFFIADGLQVVANGALRGRNDTKVPLLFAVLGFWVIGFPFCWVLGFHTDLGPFGVWIGLAVGLVVYAALLVWRFHRLTRDAMAAAVAA

>Q7N1G0_MATE

MAKFSNWRELKQLLFFSFPIIVSQIARTAMSFVDIVMSGHYATADLAAVTLGSSIWFPIFVLGYGTIIMLAADVAKQKAQHDDEGIKDSLKNYLFLAVILSIPIIILLMLVSWLLSFIGIDEHILEITQGYVIALACGVPSVMIFNVFRSFLQGLEDTKIAMYLSAGALLLNIPLNYILIYGKLGLPEMGGIGAGITTAIINNLIAVCLIIYFLLKKEYRRYRPDFSLPKYNSLIRTFYIGMPSGLALFVEMVFLDVIAITAAPLGAQVIAAHNIMLNITSIIYTITGGIAAAVTVRVGSYIGKRDKISLTGTIKISIALILSISAVIGVLIYYFAGSFISLYTNDNGVIIIALNIIFLLCLFQFFDSCQAALSGILRGFHDTRSVFYAPLFGYWLVGLPLGFILALTDWVTERMGIIGFWYGLVLGLFVNAILLFIILKVRQRGMISRLISY

>Q879Z5_MATE

MFLPRPDFRIALSICFMAVSFVISRFGSEVRPTLLLALPLVLGHVSTGLIGFVLNVIAGHHSTVTLAASTIGTALLWLPMLVPMGTLISLTVLVSQLHGAERERDIGPLFRQALWLAMLLGLVMFTFLSVVPALLPLFGIVPDIVPGAAKFLHVVRWGSLAFPLYFCMRYFCEGMHCTFPTMLLGFGGLLVLVPLSYALTYGRFGFAEYGVEGLGIATVTVMWLQAVVFALYLWRSRRFAHLQLFAHLELPCWARIRDLLNIGLPIGISILMEGGLFIVTTLLIGRFGTDEIAAHQIALSVAQLCFMIPMGVAEATTVRIGHAVGRCDLLVMRRVAWAGYAIVIGTQTLSASVLLLGYDVIVAAYTDDLVVASLASKLLLFAAIFQFPDGLQMLSSGVLRGMKDTRVPMLLAMISYWGLGMPLGLGLGFALEWNSRGMWIGLIIGLTAAALLLGWRFRVVSERMFAGIP

>Q89AX2_MATE

MKKHLHEIKMLLKITIPIFLAQISQTSMSLINSIMIGHLKENNIAAISVGISIWSPIILFGHGLLLSLVPTVSRIHGSGKINKIPEQINNAYWLATLISLVIMIVLWNSDVIIHTISQVNPIIEQESIKYIRILLWSTPGYLYFQVIQNQCEGLLKPKPAMVIGLIGLLFNIVVSYTLISEKFHCFNYGSTGCGISAIIVYWFMFIAMKKITKNDILINYNIKNKNISNLEMYLPNYKIIWNLFKMGFPIALSLFCEITLFTLITLLIASMETFQIIAHQIALNISSTIFILPLSIATAASIRLGFYLGKKSFSKISTIILSSQIIGLIISTTISTFIILFHYQIITLYTKNANIIKLTKQMLFITASYQIFDFFQIIGNGILRSYKDTNIIFIITCTSYWIVGFPFGYFLALTNYIVPHMGAIGFWYGILIALITSSIMILFRIYILQKK

>Q8G2I1_MATE

MDGTFDAGFREPTISKANRWGREMVVALKLGWPLIFTNLSQAALTATDVIFIGRLGADTLASALLATSFYHTLMIFSMGLVSAVMPMIAIALGKNRHSVRDVRRTVRQGFWSAIMIVIPLWVVLWHCEEIFLFLGQRPDIAARSTDFMHTLQWALLPYLFYIVLRSFFAAMEKPMWTLLVAALAIGFNALAGWTLIFGHFGFAPMGLHGAGMATTASSTMMFLGLAFITLRHPRFRRYHLFGRFWRPDWPRLIELWRIGLPMALTFVFETSIFYAAVVMMGRIGPTAMAAHAVAIQIASLSFMVPLGFGQVATVRVGRAYGRGDPKAIAYAGWSAYALGVGFMALMGILMVLMPRVFIGIFLNLNDPQNLPVMELAVTFLALAALFQIVDGAQAVAAGMLRGLRDTRIPMLLALFGYWGVGLPLGAVLAFQFGMGGVGIWLGLAAGLGMVAVLMTIRWRRHLAHVSAVAAA

>Q8UDF5_MATE

MSSSVVAETVPSGSGSWFSHFKATLVLGIPLIGAQLAQLGIHTTDMVIVGQLGAEKLAAMVLAGQFFFVVFIFGSGFSVAVVPMVAQAYGQGDATSARRSLRMGMWVAIAYWLLALPIFFNAERILVYLGQNPNVAALTGHYLAIAKFGLLPALLFYVLRGLVSAIGRAGIILYVTIIMLVMNGLMAYVLVFGHFGLPAMGMNGAAVVAVIVNAFSFIFIVAYVQTREETKKYELFVRFWRPDWHALFEVLRLGLPISITILAEVTLFAAASILMGQIGTVQLAAHGIALQLASIAFMIPLGLSQAATVRVGVARGQGDFKNLIRASIMIYAIACGIALCGGILFAAVPEFLAKWFLDPKLPEAAEVLAYASSLVVIAGIFQLVDGIQAVTAGLLRGLKDARIPAMLALISYWPIGLALAWTMAFPLGFGGRGVWFGFVIGLSTAAVLLTVRFVLLVKREMKTAR

>Q9HTR0_MATE

MSSPSLVELKAILRLAGPLIAAQLAYVAMVFTDTVMMGKLGPDALAAGGLGAVSYAFVSTFCVGVVAAVGNLVAIRHGCDDAAGAAAAARSGLWVGAALALAAGLLLWNLRPLLLVFGQAPQTVDGAMQFLHSLTFALPGYMAFMVLRGFTSAIDRAGPVMAISVLGALANLALNYSFIEGLFGLPRLGLAGIGLVTALVMNCMPLLLALYIRLQPAYAEYSLLRGLGRPQRAMVEEILRLGLPIGGTYAVESGMFTVATLCMGIIGDHALAAHQIAIQAVYVAFMVPVGLSYATTYRIGQHFGAGRLLEARRAGRVGIGFGALCMLLFAGLFWWMPEAIIGLFLDRDAPANREVAAMAVSLLAIAAWFELFDGTQNVAMGAIRGLKDARTTFLVGLACYWLVGVPLACLLAFAAGWGAAGVWWGLAGGLACAAIGLTLAFEWKTARLLPKATASEASALNCRAAGRGAPSARLCPGNAPVPPTAAAD

>Q9JV27_MATE

MLLDLNRFSFSVFLKEVRLLTALALPMLLAQVAQVGIGFVDTVMAGGAGKEDLAAVALGSSAFATVYITFMGIMAALNPMIAQLYGAGKTDEVGETGRQGIWFGLFLGVFGMVLMWAAITPFRNWLTLSDYVEGTMAQYMLFTSLAMPAAMVHRALHAYASSLNRPRLIMLVSFAAFVLNVPLNYIFVYGKFGMPALGGAGCGLATMAVFWFSALALWIYIAKENFFRPFGLTAKFGKPDWAVFKQIWKIGAPIGLSYFLEASAFSFIVFLIAPFGEDYVAAQQVGISLSGILYMIPQSVGSAGTVRIGFSLGRREFSRARYISGVSLVSGWMLAVITVLSLVLFRSPLVSMYNNDPAVLSIAATVLLFAGLFQPADFTQCIASYALRGYKVTKVPMFIHAAAFWGCGLLPGYLLAYRFDMGIYGFWTALIASLTIAAIALVWCLELCSREMVRSHKAV

>Q9KEJ2_MATE

MKPTETLQEKGKLFLVVMMPILITQIGLYAMNFFDTVMSGQAGANDLAGVAIGSSLWVPVFTGLNGVLLALTPIIAQSIGAEKRDDVPYVFLQGLYLSIAISIAVILIGAVVLDPILSAMSLEDEVGRIAKEYLIGLAFGIVPLFIYTTIRCLIDSLGETRVTMFITLLSLPINIFFNYVLIFGKLGFPRLGGVGAGYASAITYWFILAVAIVVVVKVRPFTDFQLFKKLYHVSLKKWKEILLLGLPIGFTIFFETSIFAAVTLLMSTFDTATIAAHQAAVNFASFLYMIPLSIAFTLTIAVGYEVGAKRVEDARQYSRLGITFALIMGLVAGVIIYVLRAPVASLYTNDSQVAWLIQQFLIYSIFFQLSDALATPIQGVLRGHKDVNVPFVMALVSFWIIGLPTGYLLANFSPLGPYGYWIGLITGLASCAIALSWRLKQMQRKFERAARLSQNGNS

>Q9RY44_MATE

MTTLPAPTISTTAELRALLRLAGPVVVSQFAANALALIATAVIGRLGERELAAAAYANAAYYLVFIMVVGVMLSVAPRVAQAHGAGDARGVARALGGGLRLALLLSAVMLPLMWALSFVLPNFAPAGVSRDLVAAYLRVYSLGMLPNLAFIALRGTLEGTGKPGAVTGVALTGVVWALLVAPALAFGWGPLPRLGLAGAAGASASAAWIMAALLWPLARRRVAYAGPLGPLGDEVRALFRLGWPIGLTLGAEGGMFSVTTLLMARFGPEVLAAHNVTMQTITAFFMVPLGIASATGVRVGTEAGAGRLAQARRAGLVGLGLSSAVMLTFAVIELAAPRTVFSVFVNVNDPANAGLIAAATGFLSIAALFQLMDGLQVTANGALRGLQDTRVPLLVSLVAYWVVGLGLGSVLSSVAGLGARGLWFGLTAGLTLAGLSLVGRFLYRTRAGRAA

>Q9WZS2_MATE

MRYSLFKNYLPKEEVPEIRKELIKLALPAMGENVLQMLFGMADTAFLGHYSWKAMSGVGLSNQVFWVVQVVLIAASMGATVTIANAIGAGNRKAVRSLAWNSVFLAIFTGVILTALTPLSDVLINIFPNLEGEIESSAKEYLKVILSGSMGFSIMAVFSAMLRGAGDTRTPMIVTGLTNFLNIFLDYAMIFGKFGFPEMGVRGAAVATILSRFVGAGILTYVIFKREEFQLRKGLVPPKWSSQKEILRVGFPTAIENFVFSTGVLMFANILLIAGAEAYAGHRIGINVESLSFMPAFGISVAITTLVGRYNGMGNKEHVLGVIRQGWILSLLFQVTVGIIIFLFPEPLIRIFTSDPQIIEISKLPVKIIGLFQFFLAIDSTMNGALRGTGNTLPPMIITFISIWTARLPVAFVMVKYFQLGLLGAWIGMIADIIFRSTLKLLFFLSGKWEKRAVLTRERVKELG

>P58163_MATE

MTVVTTMPRDAAGTALLPERPRGPIMTDLIELLRLAGPVVLSRLGIMVMGLTDAIVVGHFSAQQLGYHAMAWAPSSVFVTATVGLLVGVQVMTARAMGAGNPHETGAVLRRGLVYAGWLGFGSMALLALFGPMFLQAMGLKDGLAEGATLPLIVFSLSLPVYAISVVLTFWLEGLSRPGPGAAMMWLANVVNLGANLLLVPGVLGPPALGAVGGAWATFIARTALALALAIFVIRMKEARELGVFDKPARDRPAEIEQRRIGYGAGASNFFEVSAFAGMNLICGWISAVAVAAYTVVLNVSAIIFMVPLGVASATAVMVGRAYGARDPAGMTRAGWIAFAVIGVIGVLFGLLLYPTKHWVALAYTTDPAALALILPALVLACLFFAPDAVQVVAAQALRARGEVWVPTITHLISYALVMGPLAWWLAIPKGMGLNGVLVSIIVTSFLAAGFLLMRFRMLDWRDRKAAQEAA

>P45272_MATE

MNFRLLSQYHADIKKLIKISLPILLAQIAQNSMGLADTIMAGRVSSTDMAAISIGASIWMPLMFFGQGLLLALPPTISYLNGSGQHHRIAHQVRQGIWLVLGVSIPLGLLIYFCEIPLQYMQMESKMSDLARDYLHAMLWGLPAYLMLINFRCLNDGIEKTKPAMVITFLGLLINIPLNYIFIYGKFGMPAFGAVGCGIATAIVNWAMCLMMIFYSYTNTQERSLKVFSQLIEMPNPKTLKKLLRLGLPIAIAICCEVALYALTSLMLSPLGATIVASHQITLNTSSFIFMFPMSIGMATTILVGQALGAGSPQNAKKIGYAALLLGLTVTIVTALITIFFRYEIASIFVTDEIVIAMAANLLLFAALYQFSDTIQMVVGGILRGYKDTKVILYITLFSYWVIGVPLGYTLGRTDWLVPHIDAKGFWIAFVVSLTFAAFLLSLRMKKMQAMNDNAILQRLEKLK

>Q9I3Y3_MATE

MNSPALPLSRGLRIRAELKELLTLAAPIMIAQLATTAMGFVDAVMAGRASPHDLAAVALGNSIWIPMFLLMTGTLLATTAKVAQRHGAGDQPGTGPLVRQALWLALLIGPLSGAVLWWLSEPILGLMKVRPELIGPSLLYLKGIALGFPAAALYHVLRCYTNGLGRTRPSMVLGIGGLLLNIPINYALIYGHFGMPKMGGPGCGWATGSVMWFMFLGMLFWVNKASIYRASQLFSRWEWPDRATIGPLVAVGLPIGIAVFAESSIFSVIALLIGGLDENVVAGHQIALNFSALVFMIPYSLGMAVTVRVGHNLGAGLPRDARFAAGVGMAAALGYACVSASLMLLLREQIAAMYSPDPAVIAIAASLIVFSALFQFSDALQVTAAGALRGYQDTRVTMIMTLFAYWGIGLPVGYSLGLTDWFQEPTGPRGLWQGLVVGLTGAAIMLCIRLARSARRFIRQHERLQREDAEAASVLGR

>757424.7.peg.2755_MFS

MSDSTTQQQPQANNNGNGKRKRQLILLTLVLLVIAVACFLYWFLHARFFEETDDAYVGGNVVQISAQVGGTVVAVKADDTQVVKAGQQLVALDAADTRLALDQAQAALAQAVRQTRQLFLNNDTLAANVAAADSNLARAREDLQRRQAGLSSGAVSQEDVSHARDAVKSAVAALDQARAAAAANRALTDHTSVTEHPNVLQAATAVRNAYLNYARVNIVAPVSGFVSKRSVQVGQRIAAGNPLMAIVPLEQIWIDANFKESQLQHIRIGQPVEVIADVYGSSVKYKGTVIGFSAGTGGAFSLLPAQNATGNWIKVVQRVPVRIALDPEQVRAHPLRIGLSTTATVDIHGDGRALEAVPTNYQTNVYDDLGKQADAIVDRIISDNASGLPQAHKSKAAAAPVAVPHT

>757424.7.peg.3311_MFS

MIIMSHSDSQAAHTLAEEAKQRNEAASAAAAAQKRKKLFSIFGGVVAIAAIGYGAYWYLIGSRYVETDNAYTATEIATVTPAINGIVAAVDVVDTQAVKKGDVLVRIDDADARLAVDQAAADLDRTERKVKGFFANDAGLAAQVLAREAEQKRASAQLLSAQADLKRAEIDLQRREALAKSGSVSGEELSNARTALLTAQANLKAAEAAEVQSRANIKATQGAQKASTVLTANTTVDDNPEVVLARAKLEQAKLDLERTVLRAPVDGVIARRQVQVGQRVQSGATLLSVVPLQQMHVDANFKEGQLTKVRIGQPVTMKADLYGGSVEYHGVVTGLSGGTGSAFAVIPAQNATGNWIKVVQRLPVRISLDPKELAQRPLSVGLSMVVEIDTRGQIQAGDAQRKSARNDNAQAAAL

>1006000.3.peg.2818_MFS

MIKSSPHCGRFLSLLAQFLDIYSYKPNFYKVISEMKRDRNVNILVMLVLLVAVGQMAQTIYIPAIAQMANDLNVREGAVQSVMAAYLLTYGVSQLFYGPLSDRVGRRPVILVGMSIFMLATLVAITTHSLTVLIIASAMQGMGTGVGGVMARTLPRDLYEGSQLRHANSLLNMGILVSPLLAPLIGGVLETLINWRACYGFLLVLCAGVTFSMARWMPETRPTGAPKTRLISNYKTLFGNSGFNCYLLMLIGGLAGIAVFEASSGVLMGGVLGLSSMTVSILFILPIPAAFFGAWFAGRPKKRFPTLMWQSVVCCLAAGVMMWIPGLLGVMNIWTLLVPAALFFFGAGMLFPLATSGAMEPFPFLAGTAGALVGGLQNIGSGVLAWLSAMMPQTGQASIGLLMTLMGLLIFLCWLPLASRFAHQGQAV

>1007096.3.peg.27_MFS

MNRIKNWKKQFVVIYTGQAFSILGSAAVQFAVIWWLTIQTESAITLTIASLVAFLPNMLIGPFAGVWIDRYNRRTVMILADGLVAVSSIILGAAFLLVETPPIWFIYIVLFLRGLGNTFHGPAMQAAIPMFVPADMLTKAGGWGNMIQSISNMMGPVLGAALMSFLPISSIMIVDILGAAFAIVCLLFVIIPDIPQTNEKMSVLSDMKQGFIAMKANKPLMAVFFPMLLMTILYMPLGSLFPLLVRSHFMGEAWHNSIVEFVFATGLLLSSLVIGVWGGMKRRFFMASLAIGLMGLATLISGALPTSGFWIFAICCFFLGASGTFMNVPVMAYVQESIAPEMMGKVFSLLMTAMTLSMPIGLLVAGPVVEVIGVNTWFFWSGVVLMADAILCRLLTRRYDKETMRPQAD

>1041522.3.peg.1113_MFS

MTALNDAERAVQNQASARPDRPAPVSSAFPAETASKPPAETALKRISKYYPAWLPSRRFIAAVIAIGGMQLLATMDSTVAIVALPKIQNELSLSDAGRSWVITAYVLTFGGLMLLGGRLGDTIGRKRTFIVGVALFTISSVLCAVAWDEATMVIARLSQGVGSAIASPTGLALVATTFPKGPARNFATAVFAAMTAVGSVMGLVVGGALTEVSWRLAFLVNVPIGLVMMYLARTALRETNRERMKLDATGALLATLACTAAVFAFSMGPEKGWVSITTIGSGVVALGAGLAFIIVERTAENPVVPFDLFRDRNRLVTFTAIFLAGGLMFSLTVCIGLYVQDILGYSALRAGVGFIPFVIAMGIGLGVSSQLVSRFSPRVLTIGGGIMLFWAMLFGWAFMHRGAAYFPNLVLPIVVGGIGIGMAVVPLTLSAIAGVGFDQIGPVSAVTLMLQSLGGPLVLAVIQAVITSRTLYMGGTTGPVKFMNDAQLAALDNGYTYGLLWLAGVAVIVGGAALLIGYTPDQVAHAQEVKEAMDAGEL

>1042163.3.peg.2341_MFS

MKKDGREIRSNAMADLEADRPSHSFQNVKTILLWLSFLAFFSVFNETVFNVSLPDIAQQYGLQPAYVNWINTSFMIAFAIGSAVYGKISDTYGVKKLLVIGLLIYSGGSLFGILAQAYFPAVLVARAIQGAGASAVPAIFMVIVVKYINAESRGKAFGMIGSMVAFGEGIGPAIGGMISHHFHWSLLFVLPIITLISLPFFIRVLPNEPARKGKVDIFGAALLSIGIVLFTLYATGDNWFYLLFSLVVLLIFSLYIRRAKQPFIEPALFQNRMFVMGVLAGSILLGTVAGFISMVPYMMRDVYHLSTGMIGGGILFPGTLSVIFFGIMGGSLVDKRGNTFVMYLGAFLIVLSFLVISLFVEKSPWITSIMLIMTFGGLSFVKTVISSSVADTLASEEAGAGMGMLNLSCFLSEGIGVAIVGGLLSKHVLDFPILPTLSVPTAFLYSNVSLVLIVAIMLGVAIYTWTYKRKERF

>1089544.3.peg.284_MFS

MTTVAAPIDRATWRICWVIVFGAFASGLDASVVTIGLDSISRDLHADLSVTQWVASGYLLALALSLPLTGWLSRRFGAGRVWLVALAAFTVASGLCALAPEVGLLIVFRLLQGLAGGMLIPAGQTVLGQQVGAARLGRVMATLGIAVSVAPALGPLVGGVLLQSLSWPWLFAINLPIGAIGLALGLRYVPRGTPTETHRIDFAGLALVAAGLPLALFAVTSWGESGQLPWPILLPALGLLAWFVLRCRRHPHPLLDFSLYRNRLYRAASLAAAFNGALIFGSGIVVTLYFQIGRQLSFVGTGLSLLGFAGATAAAAPFTGRAVDRYGTAPVALAGAVLAVASTMPFAFLPANAPMAVVQLLLAGYGASVALVSMPMGIAAYKTVSPAKLPDAAAQVTILLRLGGSLGGAAFTVLIANHLPDVAAAFRLGFLAVSVGAGGALAAAWLVARAARNPGRAETMAG

>1095552.3.peg.572_MFS

MSIVGKKAQPGLAASPRDRLKVVRAASQGALSGGQFFLLNFALLLGNVLVLFNTGAFASISLHATGGLGVSPSHASWMQTYYFISMAIALPVSSWMAARFGRVRLFIVAMMLMALGSLLCSVADELVWFLLGRVLQGFFGGLTIPLSQTLLLNEYPEPKKAFAVALWSMAALSPFTLGPAAGGWIADALGWRWLFYLNFPLALVSAALVWALLFDRTSNRTDKPFDRMGFLLLAVALGCLQTALNQGQDADWYNSGLIVSLALIGLLALAGFIIWELAERHPLLDIRLLTRRNFAIGSIVLSVSFLLMYGLLSILLVRLQSVAGYTSFMVGSVLLPLIFLAKPMAVFFHRIVHYFDARWLAGLNLAAFAAFCFWTSTYDFFRRNSLFSDTLGSQVLEGFCLGGLFVPLTTLFLSGLTPRRQNQAVELGGLLRVLGGSIASPLLGVIWERRAAFHQSRLIETLTPYDIVGRETIASLNAADMPGQIATARLAELAGGHAAILGLNDTFRIAAWIFLALAALVWFAHPAGPTRRLLPRQAVRKTALEALVEEP

>1104996.3.peg.2243_MFS

MAEGDFIPRDRVITMVREIMLFKTLPTQTIIGLCALFACTFTALTSEVAPVGLLIDMAQAFHIAEGQAGLAVSAFALMVALGAVPLTILTVAVDRKKLMLLSLGGYILSNLIVALAPTFLILCAGRAVGGVAHALLMSIVSAYAARLAPANMTGRAISFVYGGTSLGAILGVPGAAAIGHFASWRIAMFVMTGLAVLLAICIAFFLPPVAPTGTGSAQLPSIGSRKAMRVFLVVVAIDALFFVAHNLLYTYVTPLLLLHGLPKAVLSLALLLTGVVSISGLWAAGQVVDRWPAAGLLGGGLAMLVGMGLMSGHIVTGWVAVASVGLWCTGYSAIIPFVMSGAIRARATRPDVAGAAINGASNLGILLGSALGGQILTWSGFNILTPLAVGVALAAILLAVFSPDAFPRILHPHEDEASS

>1120960.3.peg.3781_MFS

MSTPRALRPLRNPAYRWLAAALVASMVGSGIWMVALVWQIVAIGGGAAELSLVAGASAVGMLLTTLLGGALADRIPQKRILLVVEVVRAASVGVVALLSLTGGLAAWQLAAVAFVGGVMAGLYYPAYSALLPSVLPEDELLAANGFEGMARPILMQAGGPALASGLIAISSPGAALAVAALTGVVAAVCILRLPETSVRGAEASTGDTGYTGAASDTDVAVDTAPRHPALALLVDVRDGFTYMVRTPWLFGTLVFASLLILLIMGPFEVLVPFVIKDVAGGGPDDHALILAAFGIGGAAGSMAVASLELPKRYLTVMNLLWAFGCLPLAVFGLTDQIWVMAIAAFLVGAAFNGGVVIWGTLLQRRVPPHMLGRVSSLDFFVSLAFMPVSMAFAGPAGEAVGLPTVFLIAGAAPLLIGVVAIFAARMRRDEIAHPLDAVDEPTDVADTADVPDAAAVAEITSDDTDAAELSRSSAREFAASVA

>1121096.3.peg.137_MFS

MNHWKSTLAVIGIGQLISILTSTIVGFSIIFWISNEFKSPTALSLAILAGFLPQFVLGLFTGVYVDRWNRKKTMFYSDLFIAFCTLCLFIVITKGYKDLSFFYLLTACRSIGSTFHAPALQASIPLLVPKHHLVRVSGLYHSIQSFSEVIAPVVGASLVVWLPIQYILLIDVIGAVAACLTLLCVQIPSLQKTKVLPDFKKELTECWHTLRRTMGILPLFVCFTLVTFVLMPVFTLFPFMTLLHFNGNILQMGVVEMGWGSGALLGGLVLACKALKSKQTLVMHTAYVILGLYLISASYLPSSAFIGFVCLTFTGGIAYSIYHALFIAIIQQNLASDMLGRTFSLIFSLSTFPSMLGIVASGYWVEAWGITSVFMISGWVIFLIGVGANFISSIKQLDNYA

>1122999.3.peg.2079_MFS

MPHVFLPERDDLSLEARVLWDARVANDGEITNMKRTLLHAPVAYDALMTWFPLRDALLPRIGERGVIVFSHAISTTNDCLLCSLYFRRTLLARGEDPEARYDLNAEEADLAEFGRSLAADGRASDELTGRLRERYGEDGLVELVAFAGLMAATNLVNTALGIDLDSELLALHTAGVRE

>1123032.3.peg.1828_MFS

MPETNDKITGKIIFITIALAMGSFLNMLNASIVNVSLTHIAGDFGMATSKSTWIITSYSVAEAIVLPLIGWLTLQFGTVKQYIWSTILFAIASLLCGLSFSLSSMVAARLLQGVVGASMIPLSQTLIMKIFPKKKQGIGIAIWTMTLILGPILGPVIGGAITDVASWRWCFYFSIPLCFLSSGVIYYMFKKDYASEKFIRVKTDVVGIFLLISGIGSLQVFLEQGTDLDWFASPSIVVLAVISFMSLVILGIWEWYHENPVINVRLFLNKNFTIGVFSLLIVSAAFYMTAVILPFWLQNVMGYTSFISGKTTATLGLPILLLSPIIGKYTDRIDNRYITITGFIIFTIVTVFTANYSLDVTSSYVSYTRALSGIGLAFFFVALNNVSLGSIKPTEIVAAAGIFNFMRNLGNSIGSSLFIPLWNHSQAYHHEVLASHIHTGNPNFLPLINSIPGSIQAKLVVINGLITKESATMGVNDVLLIAGFITLALVPFVLLANRTTGSTQGGH

>1123308.3.peg.229_MFS

MKRYKIQNIYFLISSRAISRIGDIMFDFANNTFLAGLNPTSLSLVAVYQSLESIIGVLFNLFGGVIADSFKRKKIIIATNILCGFVCIILSFISQEHWLVYAIVITNVILAFMSDFSGPSYKAFTKEIVKKDYITRLNSSLETTSTIIKVTIPMVAIFLYNILGIHGVLLLDGLSFLIAASLIFFVVPVNEEVISKKKVTIKGILIDLKMGFKYVYSHKSIFIIIILSAVVNFFLAAYNLLLPYSNQMFGNISSGLYGIFLTAEAIGGFIGAVLSGFVNKELSSKRLMMFLTLSGLMLMLATPLYTIFHNLIILSFSPALFSLFLSIFNIQFFSIVQRDVDNEFLGRVFGIIFTIAILFMPIGTGIFSIILDPRNVFNFLIIGVSITLLSLIFGTLFKKYNVH

>1123497.3.peg.2297_MFS

MSQSQEFQPANMALCVFAIALGVFMQVLDTTIANVSLPTIAGNMGVSLNQGTWVITSFTVSNAIGLPITAWLSRRIGEVHLYVGALIAFSVTSFLCGISQTMGELVIFRTLQGLAAAPLFPMSQVLLMSVFPKEKRSMALALIGMVAVVGPIVGPILGGWLTYDYSWPWIFFINIPIGIFSVTVILSQLKDRPHQPMKTKLDIVGLATMALGVGALQIVLDKGNELDWFANNWIVGGAVFSVIMLIFMVIWELTDENPIINLRLFANRNFCIGTIILTLGFAGFFSINLILPQWLQSQMDYTALWAGLAAAPMGIIPLFMTPILGRFGSHLDMRKLASLSFVVIGLSCYARARFNSDVDFATIALVQLFMGIGISLFFMPMTTILLSDLHGPEIADATSLSTFIRTIGASFASSLTSWIWSRNAGVHHSIMAEQISPYNPQIAPSLQHGDPVSFLAQWNGIITSQSFMMSTIDLFSILTLLFAALVPLIFLTRKAVKEA

>1128399.3.peg.2447_MFS

MDEWVVQAVEHKPSMRLGHGAASTSPKPVPDQIRPHREPSAHSVGACALASTRNAPMTRSLIVIFTAIVLDAVGIGLIFPILPSLLQDITHAANVAPFIGAMTALYALMQFIFAPVLGALSDRLGRRPVLLISLAGAAVNYLFLAFAPNLTLLFIGRAIAGLTSANISVATAYITDISPEEKRARRFGLFNAMFGLGFIIGPVLGGVLGDHWLRLPFIAAAVLNGANLLLAVFVLPESRPGRREKIDLAALNPLKPLRSVLEVKSLLPIVILFFIFSATGEAYGTCWALWGADAFQWNGLSIGLSLGAFGICQTFAQALLPGPAVKLLGERAAILVGVAGVSLALTVMAFAGQGWMIFAIMPVFTLGGIGVPALQSLATRQVDENSQGQFQGVLASAVSLASIAAPLGFSSLYFLFRDEWPGAIWLSVVAVYALAVPLVLGLRLKMPERAAVS

>1134055.3.peg.2323_MFS

MKPEIVLRGWRFWTIQFVLPLEFVLALYGSSSYAAFNLYSVGDLGQSPSHASWSSAIFFAGRGFGMFLAPLVSRRFRSIPSLLASCFGLSAVSFFCGLIGDFYLFLVLRLLLGFLSGTAMILAQFITLRLHPVERWPNVITGFGLLLGSTFAFGPNVGAILEEAVGWRAFFLIAALLHLFFGSLLWAVLVRRQEEPVAFRFDWVGLGLMLLSMLFLQAVVVRGQDEDWYNSTFVIVLAAVSVLSLIAFVIWELGQKEPLIDVRLFLQPHYTTAVLASSVLLMLAFGMLSLILLNLQAVGGYTPDLAARSFLPVFLLMPAGWILATYLNRHVDPRWPSALYLLGFAAFAYWVSTYDYFGRRSWYTNLLGSQVLEGFCLGAIATLTAVALQRTPRHRESTASQTLMLVRTYGMSWGPGILGTFLTHRTAFQQTRLVETAPWGDPAFGLALDRLLQAGASSLQGVRLLGRYASSHAVMLATEDVFRFCFWCFLGLAILVCTPLARKREPTRANQSPE

>1138383.4.peg.3759_MFS

MAGQAMTSNGTRPAAKFPGGMQAWGMQTDSTDTPEIGAGVRWSIMVVSLLATASSFLFINGVAFLIPSLRVRGVRLDEAALLASMPSWGMVVTLVLWGYVLDRVGERVVMATGSALTAAAAYAAASAHSLVLMSVYLFLGGMAAASCNTAGGRLVSAWFPPQQRGLAMGIRQTAQPLGIALGAMVIPELAEHGPQHGLRFAALACAVGAIASVIGIVDPPRKPRASASHQELASPYRKSLTLWRIHAVAGLMMMPQTVTVTFMLVWLIRNLHWSVTAAGGLVTLSQLLGALGRVAVGRLSDRVGSRMRPVRYIAAVAVLALLLLAWADYMNSRWQAGLMVVIAVISVLDNGLEATAITEFAGPYWSGRALGIQNTTQRMMAAAGPPLFGALIAAAKYPPAWLLCALFPLAAVPLVPTRLLPPGLETRARRQTVRRVRWWRAIRSHAMPNRPERRLPQRSGENY

>1154756.4.peg.1911_MFS

MSLVSSNTLEQGFEAPRRYLAAAAILIGVVMAALDSSIVNISLPSIAEALRVDSASVIWVTNGYQVASAATMLICASLGSRIGERRFYTAGMVLFTLASLGCSLSSTFGMLVAMRVLQGVSYAVMISVGLGLYRVIFPPNALGTILGINALAFAVGTAIGPALGGLIISYLDWPWLFYINIPLGALAIVFSLISLGVDTDEREKGFDWGGAVTSAAALGLMVIAVDQIGRWDSRILILCGVASVVLVAIFLNAQRRSKNPLLPLDIFHSRRYSFAVISSVSMFVAQGMALVGLPFVLQHAYHYSVLEAAFIFTPWPIAVAICAPIAGRLSNRLNPTQISTVGVMIFCLGLGSLALLPEAATMNDFLWRVAVCGIGYGLFLPPNNKEMFSNVAANRTVTASGVLSTARTAGQSIGAALVAMVIALLNGLTNDAGAQFAVYVFGLACLISALSSLSSMLRLHR

>1156937.4.peg.1301_MFS

MNKTIKTFLLFTLYFLLQCLCFFQSGSYTDIIPYATAELGQSQSHGSWTNGFFFLGQSFGLLIATQISLQYGRKKTVFFFSFLLAASSLFCALSQNFYLFLVGRTIQGICCGVLILGSQSLVFEQSPDSWRLMPLMLGAVASVLPFTIGPVVGGYGKELVGRESMSWKYWFVLSAACLLILSFLLHLCLEDTKERIEKRPWDWKGLILLSSMLGPLQMIFNMGDDYEWFISPIIDFLFFLVVISFVCFIYVETTTKEPLVRIDLFLRKNFLIGTFSLAFGFLLFYGLWTTLLVRLQNQSLFPPHLAGILFVSMALFSTPIVIWFPRLLGRISLRLSSFVVFFLLGIVYCWMGYFDFYQKRWFWMQPSFSFILQGISLGLFFLSLTNLIISGLSPKNQLRAIELSSSLRILAQGWASPLIGTLIYHRIVYHKMRLDEWLDRGNLFLTDLFLRFKEQGLGKEIAIRLLDQSAITHAFILSLNDAFRLCGVGFLILSGIILLAKEKR

>1193181.3.peg.769_MFS

MTSYDSSSSGAADAPASPASSAPAQDNPKRAVPVLLGLFVFSLIVDSAFRFTSKPIADDLGLSVTTVSLQTTLAGIIIGVGAVVYATLADSISMRKILLAAIAMICAGSLIGFAFRENWSMILTGRIIQTSGLAAAETLYVIYVTKYLSKEDRRTYLGFSTSAFQLAMLVGILTTGYISTYISWSVLFLVPLLSVLAVPSVLKTVPDHQLSGSRLDVFGIVLIAALATNVMLFLQNFNWWFMVPVVISIALLWWHISSHTNVLVDRAFFADRRYVSMLLVVFILYSVQLAYIFMFPFMVSELYGISFDNISLLTVPGYACAVVVGALSGKIGERLSVRSTITLAMVLIVASLLIPAVFVTTSVVPFVLSMVVFGSGFALMYAPLVATAIREITPERSGVAIGFYNLTINVAVSVGIAYTAKLLDLKPSLFDGIVSTPDGFDPSFSNVLVIVAVVALLGLVVYRVASSLLARADRAAGRPVETAALDG

>1195763.3.peg.611_MFS

MTLTMERYCGVGRGYSPPFLILFTHDMTLCFAVGLQPGTVVVEIMNIVDFSHSVVSIIPPLVALGLAILTRHVLFSLGVGIVLGALLLSDFAPLQAASYIGTTVKGLFIDDGSINSWNMSIVAFLILLGMTTALLTLSGGTRAFAEWAQTKIKTKRGAKLLAAFLGVFIFVDDYFNSLAVGSISRPVTDRFYVSRAKLAYILDSTAAPMCVLMPASSWGAYIITLIGGILVSHGVTEYTPLGAFLQLAPMNFYAVFALLMVFVVAWFQLDIGPMKKHELEASHCRGFDEGDADKRAKDLNEELEIVESANGKVSDLVMPIIALIIATFFFMIYTGSQALSADDLPFTLLGAFENTDVGMSLVYGGLIGLVSALIPIFRQRIAMGDVVSTMWIGAKSMFGAILILLFAWSIGSVIGDMATGKYLSTLVEGSLDPMLLPAILFLLAGVMAFATGTSWGTFGIMLPIAGDLAAATDIMLMLPMLGAVLAGSVFGDHCSPISDTTILSSTGARCHHIDHVSTQLPYALSIALVSTIGFLVLGATDSLAVAFLAATVAFIIMCSVLYWISRRSSDLTAKA

>1202785.3.peg.1721_MFS

MALESKKELKPALLVVLQGLFSIEFLLGTYSPPAYATFNLYPAGDLGVSPSHASWISTIYFAGQAFGLFIGPWFDRAFGRVKSLLLSIGFFALFDFLVAISSDYYLSLFFRLLLGISGGSTMTLCQLNLLDYYPISRWPFVTTYFGFLQVSVFGFGPVVGGFINESFGWRAYFLTSCSLHIICGLIISWILLVLTEKRQEDTPVPHPFDWIGFMLLLFAALCFQTLVTRGQDEDWYNSTFIDLLFLFGGISLLYFVVWEMGEKNPFINLKLFFKPTFLISSIITPISFAIVYGLFSTLVFTLQVLKNHTNFSSFQAGLAMAPLLFFLPIIYPLSVFLSPRINPKIVASILLILLGIFCYWTGYYDFFNKRAFFDQFFNQYILFTQVLNGAYVGLVPALNAIAINGLSKKNQESAVNTSILLRTYFLTWGGGLLGTMLMEHRRDFQQTRLVETFTGQNSESLSFIASLQHLGLNNLQIQSKMVEQAASHSIILALDDTYRLCSWIFFLMAILVWIPAMKKKEYNFLKIFYNVCRKEKNDPKIILALAKYYHGMLFIYGALDKRQILQRSFSAISVNAYWHDGCRLCYSAKRHTCCSSP

>1206730.4.peg.6049_MFS

MPFALYMLALAVFVMGTSEFMLAGLLPAIASDLDVSVGTAGLLTSAFAIGMVVGAPAMAASARHWPPRLTLLVCLLAFASCHVVAGVTPAFTVLFISRVVAALANAGFLAVALSTSTTLVPEDRKGRALAILLSGTTIAMVAGVPAGALLGTALGWRATFWAIALLCIPAALGILQGVPNQSAGAATGNGPAPGIASELRQLRSSRLLLAMSLGALINGGTFAAFTFLAPIVTRYAGLSDGWISVVLVVFGLGSFLGVTIAGRMSDQRPGLVIAVGGPLLSAGWVALASFGSHPALLIVLVLAQGVLAFGVGSTLITRVLYAATGAPTMGGSYATAALNLGAAAGPALGAAGIAAGLGGPAPVWVAAAMTTAALGVALLSGRMLTCDAMEVTRWRRGDRYSRPSPRKLPGSASKVWGPDRKHISTKARSPWSSPRSTACRSTPCWCTSWWCWCRSRR

>1212819.3.peg.2871_MFS

MDEALRITGARGILLTPGDVPAGGASLLVRRFVPMRALLPRCRALVHHGGIGTAALAYEAGIAQVVTPFAHDQFDNAQRVAASGCGVRLDGPVDGVRLGAALARVLDDPARRRAGRLRARGGFHRAVRAGAGARGRAIRTVRRRRRGGKRMSTASPLHDAHAAPASPAAAPDTSARPLRGARLALLTFALSLATFIEVLDSTVTNVAVPAISGSLGVSNSQGTWVISSYSVAAAIAVPLTGWLARRVGELRLFVGAVLLFTLTSLLCGLARDLHVLVVCRALQGLFSGPMVPLSQTILLRAFPADKRTVALALWAMTVLLAPIFGPVVGGWIIDSFSWPWIFLINLPIGIFSFAVCTAMLRPDAQRGAAGPVDVPGIVLLVVGVGALQAMLDLGHDKGWFGSPLIVTLAIVAALAIVSLLIWEAGDAHPVIELSLFRDRTFSFCVLIISLGMMSFSVVGVVFPLWLQAVMGYNAFHAGLATAPLGILALVFSILVGLHAHRFDARVLATFGFLVFAGVLAWDAHFTLNMTFAQIVAPGLIQGIGLPCFFIPLTAATLSRIPDDKLAAASSLSNFLRTLSAAFGTAMSVTLWDNRATYHYDVVSQSVTQASANTQRFVHALNAMGINGVRELTTLNRVVMQQAYMMATGDMFWMASMTCVALAAMMWLTRPKRGAAASFGH

>1235279.3.peg.2664_MFS

MIERTGIKGENFMKKNIMLTTVLINLFIAFMGIGLVIPVLPALINELGLSGSAAANLVAAFALTQLIVSPIAGKWTDKYGRKRMIVVGLILFSLSELLFGLAQSISLLFVSRLLGGISAAFIMPAVTAFIADITTIDERPKALGYMSAAISTGFIVGPGFGGFLAEIGTRVPFFAAFGLAFIAALFSMAALREPKRQKAEAEEMVPGTTGIRKIFAPVFFIAFVIIFILSFGLAAFESLFALYTDHKYGFTPKDIAIMVTGGGVVGAVAQVFLFDRLNKWLGEIRLVRWCLIVSAVLVYCVTLVSSYFMILLVTMTVFVGFDLVRPAVTTYLSKVAGNEQGFAGGMNSMFTSLGNVFGPVIGGILFDMQLDYPFYFATATLAAGVILSYFWKKPKALAAGPS

>1235795.3.peg.2132_MFS

MKTKSKQNESVQNRVSTKLFMMVLVLSSLLAAITVDMVNPVLGLISESLQASTVQVSWVVTGITLLLAIGIPLYGRMSDFIELKKLYTFATFVLSIGSLICVLAPSLPVLVLGRMVQGAGMSAIPVLSVVAVSKFFAEGKRGTALGVIAGCIGIGTALGPIFGGVVGQTWGWPALFWITFILSLFTVVGSIFALPGNKPITADEAGRGFDLAGGALLGLAVGLFLLGVTQGFTSLSTLGSLLGSLISMIGFIWRIGVARNPFVRPDLFKNKFYVSSVVVAFLSAFSYFAVLVYVPLLNLEVNQLTPGEAGLTLLPGGAAVALLSPWVGRISDRVGTKSLIFTGLIVMGSSTFFLSTFASGASPIMSSVGVMGAGIAFALVNSPATNSAVKVLQKDMIGVGMGFFQGALYLGAGAGASLVGAFLHARRDANFPLNPMYRLDVVNYSDSFLVVTIAVIVALIASIGLKNDKQGSRLVKPTK

>1242245.3.peg.4106_MFS

MRSKDFSWRYSLPATLLLLSPFDLLASLGMDMYLPVVPFMADALGSGAGTIQLTLTAYLVLLGAGQLLFGPLSDRLGRRPVLLGGGIAYIAASFGLTVVSSPELFLSFRVLQACGASACLVSTFATVRDIYSGREESNVIYGLLGSMLAMVPAIGPLLGALVDAWLGWRAIFGLLGMAMIGAVIAAWRLWPETRRHRTADLQWSQLLTPVKHLNFWLYTLCYSAGMGSFFVFFSTAPWLMMGRQGLSQLSFSLLFATVAIAMMATARIMGRLIPRWGSLKTLRVGMGCLMAGALLLAVGETLAPVSVLGFIAPMWLVGVGIATAVSVAPNAALRGFDHIAGTATAVYFCLGGLLLGIIGTLIITLLSTGTTWPIIAYCLILATAVLCLSCINPNRRHLSQEEHDALALQGTDSAQSVHDHD

>1243664.3.peg.3878_MFS

MASFFSKTSDMMTAKQRWTALIVLAASLFVVMMDMTILIMALPDLVRDLNPTSTQQLWIVDIYSLILAGFIIPMSALADKWGRKKALLTGFALFGLVSLLIFFAESASYVIAIRFLLGFAGALIMPTTLSMIRVIFENPKERATALAVWSIVSSVGTVFGPIIGGALLEEFSWHSAFLINVPFALLAVVAGLFLLPESRVSKSQAHSWDIPSTFLSVAGMIALVWSIKEFSKEGLSELTPWIVIVAAFVMLILFVRRNLTSSKPMLDVRLFNSRPFSAGTIAALMTMFAMASVILLVAQWLQVVEGLSPFKAGFYLLPMAVGAMVFAPLAPGLAARLGAKIVLPIGIAIAAIGMFIMYFFGHPLTYPTLAVALILVGAGTASLAVASALIMLETPTEKAGNAAAIEESMYDLGNVFGVAVLGSLASQLYRSYLDIEAFSSNGIVGELAHIANESVVGAIEVAKITGFTKLATEATAAFNDSFVTTALIGGIIMMIVAVIVFILIPKSLDITKQNHH

>1265868.3.peg.2237_MFS

MSSANPGPAGTADQAGGAFTHRQILTAMSGLLLAVFLAALDQTVIATAMRTIADDLHGQTEQAWATTGYLIASVLAMPFYGKLSDIYGRKPMYLISIVVFIGGSVLCGTAGSMWELALFRAVQGLGGGGLMSLPTAVVADLAPVRERGRYFAFLQMAWVVASVAGPLAGGFFAEAGQVFGIDGWRWVFLLNVPLGLLALVTVRKALNLPHERREHRMDVLGAAALALFLVPLLIVAEQGRTWGWGSPAALALFALGAAGLAVFIPVELRRGDEAILPLGLFRRGSIALCSAVNFTIGVGIFGTVTTLPLFLQMVQGRTPTQAGLVVIPFMLGTIASQMVSGKLIASSGRFKKLAIVGLGSMAGALLAMATTGATTPMWGIVLIVLWLGVGIGLSQTVITLAMQNSAPKSQLGVANGASGLCRQIGGSTGIAVLFSVMFAVALGRLADLLHTPRYERLLTDPAITGDPANHRFLDMAESGQGAGINLDDTSLLNGIDARLMQPVTDSFAHGFHIMFLAGGVVLLAGFVMTWFLRELQEETAPEEERPAESGAGAKNGPLPASDA

>1332070.3.peg.3602_MFS

MIAKVNNWRQNSRHNSLSLRSPALAVPYFFFTISKIKMKTSLPPAALLGRQALLFPLCLVLFEFATYIANDMIQPGMLAVVADFNAGVEWVPTSMTAYLAGGIFLQWLLGPLSDRRGRRPVMLAGVLFFIVTCLAILLVTNIEQFIVMRFLQGIGLCFIGAVGYATIQESFEEATCIKITALMANVALIAPLLGPLAGAAWVHVASWQSMFVLFAALAAIAFVGLWKAMPETATLRGEAFSAANLWRDYRQVLANRRFICGSLAIGFASLPLLAWIAQSPVILIKGESLSALDYGLLQIPVFGALILGNLTLARITGKLSIERPIKLGAWPMLLGLLLAALATVFSAHAYLWMTAGLSLYAYGIGLANAGLYRLTLFSSNVSKGAVSAVMGMLSMSVFTIGIELAKVAYVWGGNGLFSLFNLVSGLCWLMLTALFLNKHRGGATPAPSVTV

>1378168.3.peg.1312_MFS

MNKLKWKQTFYFLWVGQAVSVLTSSILQMALIWHLTVITQSAFVLSMASLAGFLPNAIFGIVAGTFVDRMDRKGILIGADLFIAVISLTLAIAAQNGNIAVWLVLAVLAIRSIGTAFHTPAISAVTPLIVPPEELTKCAGFTQSLQTIGYMAGTAIAGILYPIWSISGMVALDVFGAIVASLVVALIKIPKIENADRANQSKSFFEETKAGYSALKKEKGIFALVWIAAAFTILYFPINALFPLMSLDYFGGTTFQASVTEIAFSVGMLVGSVILGIGGGIKNRGLAIPFSIMLMGVPITFSGLLPQSGFWAFAFFCIIMGASAPFHNGPVTALIQEKLPPEYLGRAFGFYGSIASLAMPVGLLISGAFADIVGITKWFFITGTLIVILALICLAVPSIRTIDKDGKKADG

>1410653.3.peg.3382_MFS

MVKKLSAYKIYLLFSAITAMCFSLVATVMVVYHIEKVHLNPLQLILVGTTLEAACFIFEIPTGIVADVYSRKLSIVIGAVLTGLGFILEGSISSFAFVLTAQIVWGLGSTFISGSVEAWIAEEEKEQDLNRMYIKGAQAGQIGAVIGIILSTMIGNLSVRLPIIISGCLFVISALFLALYMPENNFTPSAPEDLNTFRKMGYTFKSGLKFVKSKSIIMILLSVTLFYGLSSEGYDRLSNAHFLQDTTLPKIGNLQPVTWFGIFGIAGMVLSAIAMQFIIKKLEEGDKNQSGKILFIVNIFYISFMLAFALTRNFNLMLVAYLSTNLFRAINDPIFNAWLNNHIDDSARATILSMNGQINALGQIIGGPIIGIIATKFSISIGIACTSLLVTPVLVLYILSLIMDKKDVKMAKGREDTYENN

>1429438.4.peg.3523_MFS

MNAATGKPPAREKWLIAVTVMLATYVAVIDLTIVNVALPQMRGTFGVTLDAVTWVAVSYNIAEIVMVTMASWFTQLMGRKRFYLACLTLFTIASIFSGLARSLEMMILMRTLQGLGGGALIPMAQAIMLEVFPEEEHGMAMAVFMMGVVLAPAMGPVLGGWLTDAYGWPWIFYINIPIGVISILLVMAFLKESAYLQQGLSRIDVVGIILLVVGLTALQLFMEQGERRDWFESNFVIAMAVLALVGLTALVIWELRVEEPIVNLRVLKNLPFLGGIAMGLIFGLTTFGSIFMLPLFLQQLQGYSVMDSGLIQMPRMLIVVAVAPIAGRLYGKLDSRLLAAIGTAVMMAGYLDMSRFTLEVGWQRMLPGLLLTGGGMAFLFSVLSAATMRTMPPALLTAAAGLFTLSRRIGGNIGYAFVANQISHRSTFHETRLVDHLTPYDSNTMQALDGLTGRLAVYGLPPGVAEQGALKLLDGAVVRQATMMAYNDVFWMMGMMFVVTFPFVLLLGGRRS

>1432558.3.peg.787_MFS

MMHRISHWLSRHAAALFFPAALILYDFSAYLTTDLIQPGILHVVRDFNADVALAPASVSLYMAGGMALQWLLGPLSDRIGRRPVLLTGALIFTLACLATLFTTSMTQFLIARFVQGTSICFIATVGYVTVQEAFEEKRSIRLMAVITSVVLVAPIVGPLSGAALMHFIHWKALFGIIAAMGLVAWLGLLLTMPETVRRGDVPFSPLGVLRDFRNVFRNRIFLLGAATLSLSYIPLMSWVAVSPVILMDAGGLTTSEFAWSQVPVFSAVIIANLSVARWVKDPTRPRFVLSAVPVQMLGLAILIVGNLVWPHVWLWSVLGTCFYAFGIGLIFPTLFRFTLFSNDLPKGTVSASLNIVILSVSALSIEGARWLWFHGGRLPFHLLACRRDCRRLLPGRTVTPPARTSGDRSPAVVSPFTEKAPS

>1432558.3.peg.3586_MFS

MCGSSLWIWLAAIIGLSAMNMLIGILIACSIVSLVLLLVVTPPRVAQYDEEAAVES

>1432561.3.peg.1625_MFS

MTPFNPNAQQIYDQLQGMGMTQQQASGWIAQQITNQGLIISANEIFWISAAIFILLLGLVWFARPPFSAGGGGGGAH

>1463858.3.peg.4357_MFS

MPDSADRTSPSPSPTVDAAGADGSTAEPTAATKADEAVHQSPRVDGTERAANTAGAGNTERAANTAGAANGPADGPARARMPIAVYILGLSVFALGTSEFMLSGLLPPLAEDMDVSIPTAGLLISAFAIGMVIGAPLLAIATLRLPRRTTLIALITVFGLGQVIGALAPSYGILFASRVISAFACAGFWAVGASVAIAMVPRDARARAMAVMIGGLSIANVLGVPAGAFLGEHLGWRSAFWAVGAASAIALVGVVTLIPRIPLPAEKPRLARELTIYRDRQVWLAIAVTALAAGGVFCAFSYLAPLLTDVAGLDGGWVPTVLALFGIGALVGTAIGGRYADAHLFGVLISGVSASTVLLGVLALAAGSPVVVVAVAFLLGVSAFYTAPALNARMFNIAAAAPTLAGATATASFNLGNTSGPWLGGVVIDADFGFASTAWAGAAMTAVAIVLAALSLRLHRTAARSRVVAGSAGTGTGTGTGTAGTLNASSSSHSLSTCSASAAQADRA

>1505605.3.peg.2079_MFS

MSDLALSAQSPEQPAAGAALAEAPTYEVGFRKWLITITVITCAIMELIDTSIINVATRQIAGNLGATIEETAWVITAYAVANIIIIPLTGFLSDFIGRKVYFTISVAVFTAASLLCGFSHSIETLIFWRIVQGLGGGALLATAQTVLVETFPPEELDTANGIFGAGIVMGPTLGPVLGGYLTDNYHWGWIFFINVPIGILATFLSWKYIKGTKSPLEGKIDYLGILFMALGIGGLQIVLEEGERKDWFSSNFVVTATIVSAVSLVLFVIRELRIKNPVVDLRVLANRNVAIGSVLRFAFGVSIYASVFLYPVFVQGFLGWNATRTGLLMLPSSLITGVLMGAMGALLNRGVSPKLLITIGFTSVIGYEVATYFLATPQAGEWDFFWPQLIRGVGFGFIFVPVSGLILAGLKGKDIAQAAGLTNMLQLLGGAVGIAAVNTYVVRRISTNRMDLLPNLSTGHPAAVERLDNLTRFFQGAGNSLDEAQRMAYGVLEGTVSTQAAIISYAEGFMLIGLICAVALPLVFFARIRKGEAIVAGAAH

>1522311.4.peg.159_MFS

MLASINSVTSIFGPVAFTTIFAFTYINADGFLWLCAAALYVPCVILIVRGTAASPKFGSWASGDSM

>1536652.3.peg.3452_MFS

MSDITESIPTNVVKSNRRKKLLIALAAVVVFSGAGATAYWALYGSHIISTDNAYAAAEVAQVTPAVGGTISEVLVTDTQAVKKGDVLVKIDQTDARLALAQAEAELGQAIMVPLTGWLAARFGPVRVFVWSTALFGIFGMLCGLSTSLGMLVVARIFRGFSGGPLMPLSQTLLLRIFPKEKAAAAIGLWSMTTLIAPVTGPILGGYLCDEYSWHWVFMISTPFAAVCAFIAWNMLKRCEAAAIRTPFDMIGLVLLVIWVAALQVMLDEGKNLDWFACDKIVALCIIAGIGFAAFIIWELYDDHPIVDLRVFRHRGFTVSVTTIGLAFAAFFGINVLIPLWLQNFMGYTATIAGLAMAWSGLSSIFVAPMAAQLARKTDPRKLVFFGVIWFGIVTLWRAVATTDMGFFDVAMPLIVMGFGMPFIFIPTTDLALGSVEAHEMDSAAGLMNFLRTLSGAFATSMITTVWGDQITRNHAELVGLADQDLSVRAMLDGSGAPLDVVNQVIDYLIVQQSVMLATNQMMVAIGAIVIVAALITWLSPKPARVVEPGTGGH

>1560354.3.peg.1457_MFS

MASWRSMFWLFALLSAIAFLILWRVMPETAGDRSHSVALPQLARARMVMLSSFAIELVVYSHSDLGSALAGDPDA

>1590596.3.peg.2139_MFS

MGKPKWFRTYLFIWSGQFVSMLSSYAVQFAIIIWLSLEYKSAQVLAYAGIASILPQAIIGPIAGVYIDRLNRKNVMMLSDAFIAVCTFVILVVLKNGAINLFWIYILLGLRSVGNAFHTPALQAIAPLIVPQNELIRVAGINQIIQSVTSIAGPAIGTLAIASFPISEVLYLDIIGAVLAITSLLLVRIPNLVEQNKGSLLTVLYDLKEGLRTVSQNRGLSLLFFFAMAITLVVMPAAIMFPLLTTGHYGGGKWEMGLIEVAWGSGMLIGGGILSVFKFESSKVILINTMYTLLGLTLLASGLLPEEAFIIFVIITIVGGLSLSVFNGCFTAIVQIEVVPEKLGRVFSLYFSLAILPSLIGLLFTGWIVDTIGINQTFIICGLLAIVLGLAAFTFPTLMQLGNNKTIDNNEN

>1678637.3.peg.358_MFS

MNSPAHDEPLAAARPREAGLRGRPWPTLLAVAVGVMMVALDSTIVAMANPAIQQDLGASLADVQWITNGYLLALAVSLITAGKLGDRFGHRQTFLVGVAGFAATSAAIGLSGSVAAIVVFRVLQGLFGALMQPSALGLLRVTFPPERLNMAIGIWSGVVGAATAAGPIIGGLLVQHVSWEAVFMINVPVGAAALAVGVVILKDTRADKAPESFDVPGIALLSAAMFCLVWGLIKAPAWGWGDLRTLGFLLAAVVAFAGFALREGRAGEPLVPLSMFHSTALSAGTVLMVLMSFSFIGGLFFVTFYLQNVHGMSPVSSGVHLLPLTGMMIVGAPVSGAVISRFGPRPPLVAGMLLTAAALWGMSTLEAGTGMGVASCWFVLLGLGLAPVMVGTTDVIVSNAPAELAGVAGGLQQSAMQVGGSLGTAVLGVLMASRVKDVLPGEWSGAGLPPLAADRAGAVEDAAKVGVAPSLPGLPRGAVAEAVHASFISGMGLAFVVGAVVAVAGAGFALFTRKGQGGEPPAAAGRAEAAEAAAPAGQG

>1682204.3.peg.5478_MFS

MALFFAPLTVIILSGQPPEKVPAAAGLSTFGRVFFGGIGTSLANVVWNNRTIMHHEILTQQSSPTNPIFNAQMNTYHSALGLSQQASYALFDHTVQSQAAMLGLNDVFYGAAIIMIIIIPLIWITKPGKAGGSSDAAAAAH

>1688405.3.peg.76_MFS

MSTSAQATAAPKPPAQPPGTYPPLEGATRIIGSVALSTAVFMNVLDTSIANVSIPTISGDLGVSTSQGTWVITSFAVANAITVPLTGWLTQRFGQVRLFLMSTLLFVLASWLCGFSPSLEALIAFRVLQGAVAGPMIPLSQALMLASFPKAKAGMALAVWSMTTLVAPVAGPLLGGWISDNYTWPWIFYINVPVGLLAAWISWRIYGERESVTRKLPIDKVGLALLVVWVGALQIMLDKGKELDWFASPTIILLACLAFVAFVFFLIWELTDAHPVVDLRLFKERNFSVGAITLAVAYGVFFGNVVLLPLWLQSNMGYTATYAGLVTAPVGFLAILLTPIVGKMLATRDPRQLVTVAFMIFALVCFMRSGFNTQTDVRTLMVPTIIQGAAMAAFFVPLTSITLSGIEPWRIPAASGLSNFLRLTAGAFGTSISTTLWENRATLHHAQLTEAARPGQQAFDQTLQTLNGLGMSHHQALSTIDGLINAQAFTMSAVDVFYASAIIFLLLTGLVWLAGPSRRAAAEAARRKRPPGRTERAPPFLSRKRASSRGCPFASGRQPASVLEEGLPVQPFHDVARQRADGREVEHRHAARAPQLRREPERGLDQVGELAAQPAGLRVFLFVEAQPVLGRQVEPVHVRLAHAVGHQHLQRRVGHGVRIEDAQRHVEAVAQAALGVQQLHVGRGRGHALEHRRRGGAFVGARGNQLDNADAAADVFLRDALHDVAQHFGHHVQRQVARHDLPHQVDAVAHEVLVDDAVVLERQIELAGQRDRAVHPHGHLDVHAVVGNLGHRGAAQDEMHLLVGRIEPGDRGVIGNERPKAL

>169963.11.peg.2889_MFS

MTSTAYKGTNKLIVGIVFGVITFWLFAQSMVNIVPAVQSDLGISSDLLSIAISLTALFSGIFIVVAGGMADKFGRVKLTYIGLILSIIGSLLLVVTQGSTLLIIGRIIQGLSAACIMPATLALMKTYFDGADRQRALSYWSIGSWGGSGICSFAGGAIATYMGWRWIFIISIVFALLGMLLIKGTPESKVVQNTKAKFDSFGLVLFVIAMVCLNLIITRGATFGWTSPITITMLVVFLVSAGLFFRVELRQANGFIDFSLFKNKAYTGATLSNFLLNAAAGTLVVANTYVQIGRGFTAFQSGLLSIGYLVCVLGMIRIGEKILQRVGARKPMILGSGITAVGIALMALTFIPGTLYTVLVFIGFALFGIGLGMYATPSTDTAISNAPEDKVGVASGIYKMASSLGGSFGVAISATIYGVIALSGNIDLAAMVGLLTNVGFCVVSLISVAITTPSAKKALELKAAKE

>1736316.3.peg.4820_MFS

MQEKTSASIDAHPRATSREWIGLAVLALPCLVYAMDMTVLNLALPVLSRELQPTSAQMLWILDIYGFFVAGFLITMGTLGDRIGRRRLLLIGAAFFAAASALAALAHTAELLIAARALLGLAGATIAPSTMALIRNMFHDPRQRQFAIGVWIAAFSLGSAIGPLVGGVLLEFFHWGSVFWAAIPVMVLTLALGPRYLPEYRDPDAGHMDLPSVALSLAAVLLTIYGLKHLAEQGVHAEGLAATVAGLALGGLFVRRQRHIAYPLLDLRLFQHAPFCAALAAYALTCLAMFGVYIFITQYLQLVLGLSPLQAGLATLPWSLAFVAGSMAAPHLAARLPRARIIVVGLAAAAVGFCGVAAGQGLWLLVPATVIMSLGMAPVFTIGNEIIITTAPPERAGAASALAETASEFSGAMGIALFGSAGMVVYRRALNAAPLSDLPADALRAAGASLGGAVHLAETLPAVQGQALLLAAHGGFTLALQAVALAGALIVMASAWLVARMLRGVDLSAAPH

>1778.10.peg.4006_MFS

MTQTTSETGSWRQLLGRHLGTSTVLAGGVAMYATNEFLTVSLLPSTIADIGGDRLYAWVVTLYLVGSVVAATTVNSILRRFGARSSFLLGLAVFGVASVACAMAPTMEVLIAGRTLQGIAGGTLAGLGYALINAALPRELWTRGSALVSAMWGVATVVGPAMGGLFAQFGLWRWAFGAMAVLAALLAILVPAVLAAIATVEDEPAAPALRVPVGSLLLVGAAALAVSVAQLPHNSAAIGALLVVGVLLVAAFVLVDRRSRATVLPPSVFGTGPLKWIYLTLAVLMMAVMVDTYVPLFGQRLGHLTPVAAGFLGASLAVGWTLSEVASASLTNPRVINRVVLVAPLLMASGLAFGAVTQRADASSGLIAVWALALLIAGTGIGMAWPHLSARAMDSVDDPSESGAAAAAINTVQLISASFGAGLAGVVVNSASGGELMEARWLYGVFTVLAALGVLASYRATRTARRSTPPAAELRP

>1791.3.peg.3532_MFS

MTQPTATAGRRTPLLLIMFAALMAGAGNGISIVAFPWLVLQRNGSALDASIVAMAGTLPLLAATVLAGAAVDFLGRRRVSMISDTLSALSVAAVPVLALIFGAHVINVAVLAGLAALGAFFDPAGMTARETMLPEAAQRAGWTLDHANSVYEAIFNLAYIVGPGIGGLLIATLGGIDTMWVTAGAFVLSIVAIGVLRLEGAGKPDPSAMSAGVWAGIVEGLRFVWNSKVLRTLAFVDLAATGLYMPMESVLFPKYFTDRNEPAQLGWVLMALSIGGLVGALGYAVMSKYMKRRTVMLTAVLTLGVAMTVIAFLPPLPVILLLCVVVGFVYGPIAPIYNYVMQTRAPQHLRGRVVGVMGSLAYAAGPLGLILAGPLADASGLHATFLALSLPMLALGVAAVFMPALRDLDSPPGDGARADSGIP

>216594.6.peg.1390_MFS

MSGVSISSFEKVTSRHSKRPGATPARTHLAGHARKGFANLTHRRQPSSAAVLLVAAFGAFLAFLDSTIVNIAFPDIQKSFPSYDLGSLSWILNAYNIVFAAFLVAAGRMADLLGRRRTFTFGVVIFTIASGLCAVAGSVEWLVAFRVLQGIGAAVLVPASLALVVEGFEPARRAHAVGLWGAAAAIASGLGPPIGGMLVDWASWRWVFLVNIPLGVVAVLATSRALVESRAAGRRRKPDLRGATLLAGALGLLTLALVKGPDWGWVSVPTLAVFAASAITLVGFVLSSMAAPVPLVEPAYLRSRPFVVGNVLTLVAAAGFYCYVLTHVLYLNYVWGYSLLKAGFAIAPAALVAAVVAALLGRVADRHGHRLIVTLGALVWAGSLFWYLQRVGTEPDFLRRWLPGQLLQGIGVGATLPVLSSAALTGVAKGGSYATTSAVVSTTRQLGAVIGVAALVILIGKPEHGAAADALRRGWAMAAICFVVVAIAAVLLGRTNSKPGQELEPEPAAAARAAPTTTEPAAALIANRATDEADLLGNLPLFAGLDAAALAELADRVEEVELQAGSYLFLAGDASDSLYVIRRGRVQVLHGDIVIKELGRGEVLGELGLLIDAPRSASVRALRDSRLVRLTKAQFDQIANRGVLAALVRVLATRLREAPPPAVHTTSPGVVVSVVGVGADAPVQSVAAGLLTALSKQLRVVDPGRVDIDGLDRAERGADKVLLHAGAQDADWRDFCLRVADRIVLVTGDPDPGAAALPARAQGADLVLAGPTASREQRRSWEELITPRSVHAVHYRRVVQDLRPLAARLAGRSIGLVLGGGGARGFAHLGILEELEQAGVAIDRFAGTSMGAVIASLGASGLDAATADAYAYEYFIRNNPLRDYAVPIKGLVRGRRTLTLLEAAFGDRLVEELPKEFRCVSVDLIARQPVVHRRGRLVDVVGCSLRLPGIYPPQVYQGRLHVDGGVLDNLPVSTLATSDGPLIAVSLASGEVPGAPLQPDGPPRVPGIGDTLIRTMTIGSQRGADVALGLAQVVIRPDTSAVGLLEFHQIDAAREAGRAAARESMPQIMALLNQRR

>225992.4.peg.2582_MFS

MRSSAIIALLIVGLDAMGLGLIMPVLPTLLRELVPAEQVAGHYGALLSLYALMQVVFAPMLGQLSDSYGRRPVLLASLAGAAVDYTIMASAPVLWVLYIGRLVSGVTGATGAVAASTIADSTGEGSRARWFGYMGACYGAGMIAGPALGGMLGGISAHAPFIAAALLNGFAFLLACIFLKETHHSHGGTGKPVRIKPFVLLRLDDALRGLGALFAVFLNDRLASPPGKSAPFPTTAPCGHTTTILKMKQRVSLRTLALLSVVVLITGCSKPEAQQAAQEPAEVGVIVAAATPTSVATELPGRLEPYREAEVRARVAGIVTARLYEEGQDVARGAALFQIDPAPLQAAYDSEAANLARAQANLSAAADKLRRYADLVSDRAISERDHAESVAQERQARAEVALARANLQSAKLKLDYARVTSPIDGRARRALVTEGALVGEGQATPLTVVQQIDPIYVNFAQPAAEVMQLQKQIRAGALESVAPDQVRVRLLLPDGSEYARGGTLSFADLAVDPGTDNVTMRALFENPGRDLLPGMYVRVRLEQAINRDTYLVPRNALLRNAEGAHVLAAGPDGELKKIAVTAHRLQGANWIVTQGLAGGERIVVENAAHLAAGQKIKPVERAAPSAQAAAAENPEAGVQARTAAEGKKG

>333849.13.peg.1278_MFS

MENEQSVVLTNWKRNYLFFLSGQFLSGITSMVVQYAIIWYLTRETGSATILSFATLLGMIPMVLLSPFVGPLVDRWDKKALLIVTDIIVAIFALILAVVGTISESFPIWLVFVSLFMRSVAQTFQMPTIQSIMPTIVPSSHITRTNGQLGMVQSANFIIAPALGAALFSVVPVNYLILLDVLGAVFGVGLLIFVKIPKVSPEILEVPLTIFKDAKFGLQQLMDNKGLWYITINGAFVMLLFMPAISLYPLMTLDYFGGSVGQAGAVEVVYAVGMLLGGALISFIGTWKDRMKPIIIAYIIMGLTIGASGLVPNDSQGFLYFLILNAGAGCATPYFNTLLMAMIQQSYESNVLGRVLGNFNSLMNLAGPIGLLFAGPLADRLGVEKMFLFSGIGILLCGIVLFLTSAARKYDKELQKKLVKEHHEQKDE

>339854.8.peg.2484_MFS

MSLLNFPGFLTKGMGIAIVGGLLSIHLLNRKLLPMNANSFTHLYSNLLLLFAGIILISWLVTMKMYKCSRRNIYQVKRFIFQ

>371042.3.peg.3824_MFS

MIHGKNMKPLRFCLPLSAFMVIMDSTIANVALPAIAGNIGASQSQSAWIISSFVAACALSVPLTRWLALRIGESHLFIAALSVFTLSSCGCGVSTNFLMLIFFRVIQGISAGPIIPLSQSLLLKLYKSDEKRDALAIWSMTAVVAPVIGPVIGGIITSYYAWNFVFLINLPLGILVVVMCRKTISNTSKNTQDKKFDFTGYLLICLLVALWQYISFRKNSGNDNGFLVIISLLLLLFFLISQMCRKNTLLDLSFFLNRNYAIGTLCIFFSYIINFGSLVPSTLFNIYNYDLVTIGLLCSPAGIAPLFLSKLSGRMCKYVDSRILISISFLIFAMCYYWRACYFSLGMTPLMFASSQFFIGIASTLFYIPLTEKLFSDISKDDLTAATTLRQLCRTLSTAFGTILTSELWNNRLFFHTSRLSEKVYAGSLEYENFYQKFKLLGLNQQETLLYIRDQISFHSKLLSLNDIYWLDAGIFLFLACFTWLLTPTKK

>381666.6.peg.5563_MFS

MTWAIYRDRKTPTRKLSIDMVGLASLVTWVASLQIMLDKGKNLDWFSSPVFTVLMAPVVGKILPKSELRVLATLSFLGFAAVYFMRSHYTTGVDTYTRRTSRSHDRTPD

>382638.14.peg.388_MFS

MRFLGLFIVLPVISLYADSFHSSSPLLIGLAVGGAYLTQIIFQTPMGILSDKIGRKVVVVVCLLLFLVGSLVCFVADDIVLLVIGRFIQGMGALGGVVSAMVADEVKEEERTKAMTIMGVFIFISFTISMAIGPGVVAFFGGAKWLFLLTAILTLLSLLMLLKVKDAPKISYQIKNKIAYQPNSKALYLLYLSSFFEKAFMTLIFVLIPLALVNEFHKDESFLILVYVPGALLGVLSMGIASVMAEKYNKPKGVMLSGVFLFIVSYLCLFLADSSFLGKYLWLFIVGVAFFFIGFATLEPIMQSLASKFARVHEKGKVLGQFTTFGYLGSFVGGVSGGLSYHYLGISNTSLVVVILGLVWGLSLFFLNNPSKQKNVYFPLDAYNGEQFETLGDKIIEWYVNISEEIIIVKYNSDQISEEEIIHLAQNFRK

>388357.3.peg.2216_MFS

MPLPRALEPFRLGEYRVLAFAMFVSVFGAGMWAVALVNQVLELDGTAVDLSAVTAVGALGMLVVVLVGGIAADRFPLAALLRLVEAGNALTAGTVAVLALTGGLRLWHLGAAAFVFGAGVGFFYPAYSAALPRVLPARQLLAANGVEGTARPLLQQAAGPAAAGVLIGLLAPGGAVALIAACHLAALVLLLRLQVPEREALPVTGPTARAPLGDPAVAPATEPMVRPVAESAEPVVESVAESAAEPVVRPVAEPVVEPVVRPSAEPVVEPAAEPGAEPVVGSAPGPEAAAGPSDHSGGVFASVRRDLMEGVRYTLHTPWLLWTLLWAVCAVFLLLGPLEVLVPFLVRDRLGGDAATFGYLLACYGGASALASLVVASLPLPRRYLSWMIGLWGLGTLPFGLVATTESFWVMAVCLACVGAGDGAGMVLWGTLLQRRVPRHMLGRVSSLDFFVSIALMPVSMAIAGPVAQVVPMPVICWTVAVLTPVLGFVALRAGRMRQDELAHPLAG

>421052.3.peg.2788_MFS

MNNTILKTNLKGIRLLIAAFIVALANFMVVLDMTIANVSLPTITGSLAISTSQGTWIITSYAIAEAIGLCVSGWIAQRFGLVRSFSIALMGFTVFSICCGLSNSLELLVMCRVGQGLFGGPIMPLSQTLIISIFPQEKYIHALGIWAATTVLGPILGPILGGIISENWAWNWIFLINVPIGFFLIYGVYLFLSKIKSPLSKSKFDVIGMIFLLVWVGALQMMLDMGHDYDWFNHPKIWVLAMITLIVFSLFLAWELTGRQPIIQLHIFANKSFCIATLALSVAYGAFFGGIVVIPQWLQLNMGYTATWAGYLMATMGVGSLLMSVVVAKLIYWIDQRLLVSIGFIVFALSCYLRTDWANNVDFIDLAWPQILQGFALPFFFIPLSNIALAAVQSHELAMATGMMNFIRTLSGAIGASISMSLWSNYSQIARHEMVARIQMTQSQHALLSAHISQQNSLELVSNVVDHEAMTISINHIFWGFSLIFILISVLIWLLPKPQNMLGQIHLP

>42253.5.peg.3181_MFS

MMALVDHTQPRLRGWHFILFNLVLGLAHMVVLFNAGSYVALLPHAAGDLGGVLPSFGTWAQTDFMIALALAFPLARWLSCRYGEQRVFVAAFVVYAAASALCAIDGSIAAFVPARILLGLAGGVTLPLSQSLLLQEYPDRVKSLGLAIWGLFTLMPFTVGLGAGGWLADHWGWRALFYLNIPVALLIAALTAALLHGRSHVVRCERFDLVGFLLLAVIFGGLQTMLNEGNDYDWFDDPFLRGMLVLVIVAVPVWIVWELGERRPAVDLRLFAHRNFAVGLLCLGLGFLSIQGLLALFVVQLQVLMGYSSELAGLVFVPMMLLGLPTIAVMHDVAKRLDVRWLACVNGLGFAATFYWIGLFDDPHSYDQIFWPMVLEGVFLGSFFTPLTVLTLHGLSGEQMLRAAEAANIFRIAAGALGISWQGVVVFRRMPFHHLQLSDHFGGRMSASYDALHQLTSKLQALGFDPAMIQRQLQLAIKQEAGILALNDAFLLSSALCMVLAVLVWFAHSSRVPALKPAEAVRELQAEELMEQP

>439375.7.peg.2032_MFS

MRSPNQLCLDNLSLINFKSSSLSLINCEAGMKRTLMIVLAVTALDAVGIGLVMPVLPSLLRDVAHSDDVAGHYGVLLSLYALMQVFFAPILGGMSDRFGRKPILLGSLIGAMIDYAIMSAAPHLWVLYAGRILSGMMGATMAVAGACIADTVEEGTRARAFGWLGACYGGGMILGPVVGGALGSISLTAPFAAAAAVNGLMALSVYLVMPEVRRTTKPEPQAKGLHCALVPSGVQKGLKPLLWVFFLLQLVGQIPAALWVIFTEDRFHWDTTYVGLSLAAFGLLHAMFQWLGTGRLVATIGAGYTIIIGIAADGLGMASLAIATEGWMTVPILVLLAFGGIAMPALQSVLSDKTSQDEQGALQGMLASLTNISAVAGPVIFTAFYMRTAASWNGWVWLFGPAIYLAAAPLLIFVRRQSAGLPSRR

>470.1295.peg.2468_MFS

MPCAVFLILLTRQIEHFLTLRFLQGIGLSVISAVGYAAIQENFAERDAIKVMALMANISLLAPLLGPVLGAFLIDYVSWHWGFVAIALLALLSWVGLKKQMPSHKVSVTKQPFSYLFDDFKKVFSNRQFLGLTLALPLVGMPLMLWIALSPIILVDELKLTSVQYGLAQFPVFLGLIVGNIVLIKIIDRLALGKTVLIGLPIMLTGTLILILGVVWQAYLIPCLLIGMTLICFGEGISFSVLYRFALMSSEVSKGTVAAAVSMLLMTSFFAMIELVRYLYTQFHLWAFVLSAFAFIALWFTQPRLALKREMQERVAQDLH

>479431.6.peg.2835_MFS

MTGISGAPRHGGRTGKLLAPTVVDRAASWTPVVPDGLRPGRDRPPAGTREHHRQESKIMTERRAPNREPRRKRLWRPRSVPRALSLPRALSLPRALRPFGNPQYRWLTTALACSLFSVGIWLVASVWQVIQLGGSASDLSLVAFGSSLGLTLSVLIGGVVADRVPQRKILLVVEAVRGVCFALAGVLALTGAIQIWHLAVLGLVLGLADGFFYPAYSAWLPAIVDADQLLAANGIEGMLRPAVMQGLGPAAAGVIIAVWSPGAAFAAVAILQIGTAAALWTMRTTAVRRELDPDVHPLRSALIDVRDGFSYMVRTRWLLTTLLFATLLVLMVVGPVEVLLPFAVKDQTGGGPGAFAVALAAFGIGGAAGSLAAASIRMPRRYLTLMILGWGFGSLPLVVVGLTSSLAVMVVALFVTGFVFSAAQVLWGTLLQRRVPPALLGRVSSLDFFVSLALMPISMALAGPVGDLVGIGPTFLVAGLVPGLLAVGTLLIAKLGPDELAHPLDALPEAAEPPVPIPDQ

>557599.3.peg.5424_MFS

MTSPTASRTAAADTRSTCISLSPARRNIIFMALMLGVLVAAMDQTIVVPALPTIVDELGVSVHQSWAITSYLLGGTIVVVVAGKLGDLFGRKRVLQGSVLVFLLGSMLCGAAQTMTTLAVSRAVQGVGAGAISVTAAALVGEAFPLRDRGRYQGILGAVFGVTTVAGPLLGGFCTDYLHWRWAFWINLPISIVVLAVTATAIPALPRRPKPAIDYLGIMVITLATTALITATSLGGSTYSWGSAPIMGLFIGATVALGVFVWVEGRAPAGILPPRLFRNQVFAVCSVLSLMVGFAMLGALTFVPMYLRYVDGASATVSGLRTLPMVVGLLTTSVGAGIMVGRTGRYKIFPVAGTGLMAVAFLLMSQMDESTPALVQSLYLVLLGAGIGLSMQVLILIVQNTSRFEDLGVATSGVTFFRVVGASFGAAIFGALFATFLGRRMGPALVAGDAPVDAAHSPAVLHRLPHYVAAPIVRAYAESLNQVFLCAAFVALAGFILALFLREVPLADIHDSPSCLGDGFAVPRTKSPEDVLEIAVTHLLHEAPEVRLPNLAAAYQDSELDVAGLWGVLRIYQYERFFDTARLTDIAQHLHLPHQVLEPVFDRLVQTGYASREGDTLSLTPAGLGQIETLSGLLRRWLVDHLAVAPGVEQQPDHQEFEAALQRLTDGVLVQRDWYEDLDELAPAGTLVAAK

>566461.4.peg.7124_MFS

MAGSYATAARNVGAAVGPLVAATTLGTAVGHLGPLGASGLLVAVALLIAFPFRTVVVAAGRGAEVLQ

>575.7.peg.222_MFS

MATLLPIIVYKICLKSLLNHRHIVVLSFLCGRFLQNLRDVPVTYSSFTSTHEVFIDMKRHKNFTLLLMLVLLVAVGQMAQTIYIPRSPTWLSR

>60547.6.peg.7263_MFS

MASPKNASTPAPFTRWQFALGTFAVAVASFMNVLDSSIANVAIPTLAGDLGVSVDEGTWVITLYATPNAVAIPLTEWLTQRVGQVKLLVVAILLFVVSFTMCSLAPNLPILLVARVIQGAMAGPLVPLSQVKRRQGRSDSECSPVQLLGNQQPIRLVVLARWERVDAAKSVRVTDSMFMAAAQGIASAPSAYINAGINLLPPVTALRDIAVSVSLAVALQAHKKVPSSGLSPDQTKGLIRGKVWAPHYVPWRKIKSSAI

>637389.3.peg.233_MFS

MVVASWSLAWSIPMLPYLAGAYSTSLDHAVWSLTFYLMAWALGVVPATWLYRRIGELRSFQLSIALLLLATLPDVLSNNYSLFLVGRFFQGLAAGFLTPLIRRLLIQYAPPKWQGFAADLSIVNLVLPLLAGPSLAGWIAYNWDWRAAPLLTFPVGMLALGVCSALIPARDSERHKAPFDWIGLALLALAAGSVQILLNRGEDWNWWDSQRFQGLTALGAIFSIAFFVWERHHPQPCLDLSLLRRRNFVLPIPALIFGWGLLLGGNSLFVSALITQAGYTAYLAGLVLFPMALTGVPLIAMMSRISHSIGPRILASVCFLLVALYGFSTQINRSSSLDSLLLAHLIEGAALGFYLVPLSLIMFSRLPSNRLPAAATLQNFVRILGGAYLSSIFSALWLRHGSYFRAHLAWQSPAAPLAELAQNLPEINSTEKAAVDVHLLVMQSLALSMQSMLALWGLMALLVLALLWFTKAPFRRRPGQKRRVTIEQEIVESADLIPIRKAATTTSSSVTDADTSSVHA

>637389.3.peg.799_MFS

MAKIDPPSVADEARVRPLSQSWPLFVGVGLGLALGSFEGAGVQAIFPYVAGGLATSSDHALWTLTYFIVNWSLGITLMPWTTARFGMRRVFLTATGVAAAGSVISGMTHNLWIMLLSRTLEGLAAGLLVPLSQSLFLRHSPKSKHALVTVFWSNAMLVPFFFGPAIGGWLATGPGFRWIFWLSLPLWLLAAVLGGRAIPAGGGDPSLPAFDLAGFVLLYAGLMGLQITLDNGEQYGWWHSPLILSSSVFALIAFVLFAWRESEARYPLLRFHYLRQRNYWLGLSLLCLGWAMFMGWAAALPLWVEQNLGYNGYWGSIVLVPIAIGAIPVSMVMDRLRSLVGLRRLATLCFLLFAASYGNFTLSPISSLGDTVLPMLFMGLAVGSLFVPLTLILLSEVPAAEIPRAATTSNFIRVFSANIGVSLISVYWTRGSALVATQMRDKIDPYTHSTWPLWQLQHLLEVEAATLSMNNLLRLCMWIALLAALAAYLLIIPPRSIARPDGPHNYVEEEELETAETPAAGELPASTTTS

>637390.5.peg.2638_MFS

MAFAHHAPAVSPLSTSMVLLLNLVIGLGHFLVLFNTGAYLPMIPHVAGSLGVNPDFADWTQADFFLAMALAFPTSPWFLQRWGEMRVLAGAFMAFALASAICAQTGHYDAFLSARIVQGFSGGLTIPVSLQIILRHYQAHRRNIGLGLWGVAALTPFTLGPIIGGWITDSIGWRWLFYLNIPIAISVAVIIVILLFGREMEHRHPPLDWPGLLLLLIALATLGSALNAGEVISWWRSLPIIFLGSISLITLIFFGIWEWYSTHPLLELTLLKRRNFMIGGIVLFFTALFFQGSIAIYIVGFQLVMGYSAWLVGLLILPMAIFSKISFILTQRLLNHLDARILAIFSLLGFAAASFWVASYNHPASFSELLWPQAFVGIFLGSLFPSIIAIALSGLSGPAEIRGTAFLNVLRLCGQAMGIPLIATLFDRRMILHAHFLAEGNRPTIYTLNPSVSNIRTAHYIAHQAAMLAFNEIFYIAAWGFLLGAGLMLFSKPVVYAEPDIRVRRAIEELVDL

>637905.5.peg.2019_MFS

MFRYLLCSFALVLVYPLGIDLYLVGLPDIARDLNASQADLHLAFSIYLAGMASTMLLAGWLADRIGRKPIALMGAATFAIASWYAASSVTVDYFLFARFGQGIGAGFCYVVTFAILRDTLDDDKRAKILTMINGITCIVPVLAPVIGHLILMGFEWPSLFISMAIMATLIFQLCLLILKETKPSHIETTHRNMNTSTQSCHRVNQAHDHKANLANEATKKICDEPLGSRLFISRLIMTSLAVTAILTYVNTSPMLLMEQMGYSTGKYSAAMAGLAVISMTSSFLAPKLLTHFGQQRIMLASQGLYICSAVVFMAGYQFELDSRVNLLGISLICAGFSLGFGTAMSQALSPFSRRAGMASSVLGIFQIACSAAYITAMGWLGISTLNMLIFLLLTTGLTSIILLHVVPSDTASSDKLTHKVSELNSNDKVPASS

>661478.3.peg.216_MFS

MSAVAAPISAAPALPAKSLRWMIAVSVSLAALLEVIDTSIVNVALTDMQATLGATLSEIGWVVTGYGIANVVMIPLSAWLGDAFGKKRYFVFSMIGFTVASIMCGMATTLPVLIGARIFQGLMGGGLLAKAQAFLFESFPKEEQGMAQALFGACVIAGPAIGPTLGGWLVTNFSWPWIFYINLPVGIAATLMCIAYLPEDVKRFGRKAVDYLGIVLLILWVGSLQILLEQGYENDWFDSRFISVLAVISSVGLVLWIWRELRTKAPAVDLRVLRHRSLTAGSVYAFVVGVGLYGALFAIPIFAQQVLGYTAYQTGMLLLPGAIASALMMPVMGRLSKVDARVLIALGSLVLIGSLVVVSRISILTGPEDLFWPLVFRGIGTVMIFLPLSLATFSGVPKEEVSAASGFYNLTRQLGGSVGIAVLTTILAQREAFHRSNLVEYVSAYSSTAAERLSALTGGFVARGAAPATARTMALKAMDRSVDVQAAVLSFGDMFHIVAFLFVVSLGLLFLMGSGKRSAAPVDVH

>66429.3.peg.1684_MFS

MNKAGQTEQPTAPETPLLPDAPEPDPKRWLALTVLLVATFMDLLDSNIITVAIPSIQRDLGASAVAVQAMTAGYTLSFAVLLITGGRLGDIFGRKRMFLTGVAGFVLSSALCAAAQNTEMLVASRALQGLTAGIMVPQVLALIHVSFAPQEIGRVVSLYASMIGLAVVSGPVVGGALVEWSPLDLGWRSIFVVNLPIGVAALAGAGKWMRESRSPHAQRLDIVGMLLAIVGLLLLMLPLTLGRELGWPVWSIVALVAALPVIALFVVHQRLKTRKDGSPLVSLSLFKVRAFSAGIGVQLLFSAVPAGFFLSWTLYLQGGLGWTALHTGLTAIPFSVCVPLVGGLAVRKLSPLYGRYCLVAGALSMLAGIASYAWAADRLGADITSWHAVPSMILLGSGMGLLMPPLTALVLREVKPQEAGAASGIINATGQLGAALGVAIIGGIFFSALAGNAGPQADRVLPAHRTVAARQAAEVKDCATDSLGQDDLTKVPRSCAALAGKSDPESMSAIGSALGEIRTKTFVATYSDTLYWAAAGLVPVAGLLFLLPHHRVRRGETA

>665792.3.peg.2625_MFS

MEKTMEAAAAPQEGASFKVVPIMTALLLAGFIGMFSETALNIALNELMSTFNVEPATIQWLTTGFLLVLAILVPISGLLLQWFTTRQLFAASLVFSIAGTLIAASAPSFAFLFIARLVQAVGTGLLIPLMFNTVLVIFPPHKRGAAMGMMGLVIMFAPAVGPAIAGLFLEYASWRTIFWTALPLLVVALLFGLMFMKNVSELARPRIDIYSIALSSFGFGGVVYGFSSAGEGDHGWSSPKVIIGIAVGIVALILFTVRQLRMKQPMMNLRAFRFPMFTLGTLMIFIGMMIILSTVILLPLYLQAGIGLLPLAAGLLLLPGGLINGVMSPIMGRLFDKYGPRWLVLPGLVLVFVVLWLLTGINTGTSKGEIILLHSLLMIGISMIMMPAQTNGLNQLPRELYPDGTAIMNTLQQVAGAIGTALAISIMTAGSKAYYADGKHSPADLSTVPAAMTQGVQNAFLFVMIFAVLGFVCALFIKRVKVGKQEQVHHAGH

>703.8.peg.1654_MFS

MIILYPVGIDLYLVAVPHIADSLHADDAQIHTAFSIYLFGMAATVLIGGVIADRYGRRRVVLAGALLFVIASLVAATATHIYGFYFGRFWQGAGAGTLYIMSFTILRDVLSQERLASALAMINGVICVIPVLAPVLGYIILSHSSWRGIFITMASIAICCGLINLVLLKETRPVTPHQRGLSTSFAVLRAPRFMLLSLLTSASVTNILVYVSVSPLLLMKQLGFTAEQYSIVMMVMAGVSMATSFLTPLLLRCFGSHNVLAFSHLAYLLALLSLIGSWHLNGNIELLLLAFSLICIGFSCGFGIAMGDALNECQQDNVAFASAILCIMQISLSGLYIWLMGYLEFTPSEMLMYSLLVSLLSYLAVKVLVPWFMVNHPNQLRG

>745310.14.peg.3050_MFS

MSTAAASASAPPAPASPALPSPAKRLAITITVMAGTLMQVLDSTIANVALPHMQASLGATQESIAWVLTSYIIAVAIATPVTGWMESRFGRRELFVASVVGFTLASAACGLAPTLETMVAARVLQGVFGAFIGPLTQAIMLDSYPREKHAQALTIWGMGVMIAPIMGPVLGGWLTDQWNWRWVFFINVPFGIVTTIASWLLLSSSRLEKTRLDITGFILISLFLVGLQLVLDRGTHLDWFDSREIVIEAALAVAALWMYVIHSATTARPLIPLALFRDRNFLIANLFMFVASGVSIAGSALTAPMLQTLLGYDAYGAGILVAPRGLAMMVSMLATSFVTKYVDGRVVIAIGLVLVAVSQMMMSGFDLEMGSRPIIFAALIQGLGLGMFVLPLNLLAFATLAPYLRTEGAALYSLSRNMGSSIAISILSALLARNTQVSHSDLAAHVSASSLPFLTPGTLERFGQQGHDILRMVDAEVNRQALMIAYIDDYWLMGWAVAVLLPFVVLMRGVGRKAGDPPPPMME

>754436.4.peg.3313_MFS

MFRYLLCSFAFVLLYPTAIDLYLVGLPQIAQDLGASESQLHIAFSVYLAGMAATMLFAGTLADRIGRKPVAMVGAAIFAMASWLGGQVDSSTPFLLARFAQGIGAGACYVVAFAVLRDTLDDQRRAKVLSMLNGITCIIPVIAPVVGHLIMLYFPWPTLFTTMAAMGVLVCLLATGVLKETNPNRRHRTDRVLEKQTETDTQETFTTPYFISRMLISSLAVTVILTFVNVSPTVLMNGMGFSRGEYASTMALTALVSMITSFATPFAMAWVAQRTLQLLSQGLFVLAAILLASASLYDLHNMVTLVGLGMICAGFSVGFGVTMSQALSPYARRAGMASSLLGIAQVCSSACFIWLMGLCDVTGLAMLIGILLTTGIINAALLLCVATPPRLSQQAPQKPQQQSPQAAEPHEEISCSS

>82380.11.peg.1417_MFS

MPPAYREADTKLALHQNWYGYTFGMTSLTSSIRNANRAWIMLVVLTMLTVIGMTVVLPVLPFVVLQYVSEEKDLALWVGVLEAVNGLCAFLIAPFLGRLSDRFGRRPVIIAAAFGAAFAMALFGIGCALWVLVLARVIQGLTAGDLPALFAYLADITPPEKRAQRFGLLGALSGIGMMIGPAIGGLLASVSLQLPVFLTAAVGLTIAILSIFLLPESLKPENRITSISVRDVQPFAVFKNAFGRKELRGLMIGFGLLALPFGFFVNNFSVLALDSIQWGPTQIGLMTAAVGIIDILIQGVLLGILLPRIGERGVIVSGIVAQMVGLIGLAVVASIFAQPWLFIVGALMLAAGQGASQAAMDGAMSNAVGDDEQGWLGGATQSLNAAMGTIAPLIAGALYVAVSHSAPYWLGAALMVVAVIVVARAHIVNTAKVGSAKVTATDAPLELLDARD

>83332.12.peg.2605_MFS

MNRTQLLTLIATGLGLFMIFLDALIVNVALPDIQRSFAVGEDGLQWVVASYSLGMAVFIMSAATLADLDGRRRWYLIGVSLFTLGSIACGLAPSIAVLTTARGAQGLGAAAVSVTSLALVSAAFPEAKEKARAIGIWTAIASIGTTTGPTLGGLLVDQWGWRSIFYVNLPMGALVLFLTLCYVEESCNERARRFDLSGQLLFIVAVGALVYAVIEGPQIGWTSVQTIVMLWTAAVGCALFVWLERRSSNPMMDLTLFRDTSYALAIATICTVFFAVYGMLLLTTQFLQNVRGYTPSVTGLMILPFSAAVAIVSPLVGHLVGRIGARVPILAGLCMLMLGLLMLIFSEHRSSALVLVGLGLCGSGVALCLTPITTVAMTAVPAERAGMASGIMSAQRAIGSTIGFAVLGSVLAAWLSATLEPHLERAVPDPVQRHVLAEIIIDSANPRAHVGGIVPRRHIEHRDPVAIAEEDFIEGIRVALLVATATLAVVFLAGWRWFPRDVHTAGSDLSERLPTAMTVECAVSHMPGATWCRLWPA

>926569.3.peg.1825_MFS

MIKKTSARLVYLILSGGNTLADTIMFTVNMVYFVEIIGLSPLQLVLVGTVLEGAILLFEIPTGVLADTIGRKVSIVTGWFIMAGGFLLVGIVPELWAVFIGQVLWGLGYTFTSGATEAWLADEIGEDLVGKINIESGQINRILGLIGSAISVAIASVALNLPIVIGGLMYLFLAVFLLFTMPETQFTPRYKSSKSLETPFQSFIQTFQEGVKAVGKSPILLALLLVELFIGAASEGYDRLSSAHLLKNFQIPPIGALQPVVWFGILNITGSLASFSTTAMFRKKLEVISQSYQLAARYLVLLHSLGIAMVVMLALTGNFYAAIAAILVKGVMGALIFPLYNAWLVQNILPTTRATVISIVGQANAFGQVVGGPGIGAVGNRSLRLAILLTALLSIPALPLYTSAQKKQTVFSPESTR

>95606.3.peg.1781_MFS

MLTSYLVANAVIVPISGWLSDVIGRKRFYMISVLLFSIASLMCGLAPSLGFLVISRILQGIGGGGLAPSEQSFLADTFPPSKRGMAFAAYGVVVVIAPVLGPSIGGWITDNISWHWIFLINVPVGAISLVLVHFLVVEPKALEKERKKKLRKGLNVDAIGFALVALGLGCLEVFMDRGQRDDWFGSGFITSMAIIAVISLVLLVVWELNQKEPIVDLKLLGVPNFAICFVMMLGVGVIIYGSTQLIPQLLQEVFGYTATDAGLALTLGGAAALLAMPLVGALSGEIQGRWFLGWAFFMQAASMWYFTGINADVSFDHIAVGRLIQAIAIPALFVPINAQAYAGLQPNRYNHASALMNVARNLGGSIGISTAQALLLQREQFHQSRIVESLNPLDPNYVEGLKQIGASLGGAKGGDADQSQLAALYQMATKQAAMISYIDVFHVLAVVMILMVPLSILLKPAKGEH

>P76242_MFS

MTCSTSLSGKNRIVLIAGILMIATTLRVTFTGAAPLLDTIRSAYSLTTAQTGLLTTLPLLAFALISPLAAPVARRFGMERSLFAALLLICAGIAIRSLPSPYLLFGGTAVIGGGIALGNVLLPGLIKRDFPHSVARLTGAYSLTMGAAAALGSAMVVPLALNGFGWQGALLMLMCFPLLALFLWLPQWRSQQHANLSTSRALHTRGIWRSPLAWQVTLFLGINSLVYYVIIGWLPAILISHGYSEAQAGSLHGLLQLATAAPGLLIPLFLHHVKDQRGIAAFVALMCAVGAVGLCFMPAHAITWTLLFGFGSGATMILGLTFIGLRASSAHQAAALSGMAQSVGYLLAACGPPLMGKIHDANGNWSVPLMGVAILSLLMAIFGLCAGRDKEIR

>P46104_MFS

MSVFARATSLFSRAARTRAADEAARSRSRWVTLVFLAVLQLLIAVDVTVVNIALPAIRDSFHVDTRQLTWVVTGYTVVGGGLLMVGGRIADLFGRRRTLLFGAFLFGASSLAAGLAPNLELLVLARFGQGAGEALSLPAAMSLIACSSRTAPFQGVERLASVASVGLVLGFLLSGVITQLFSWRWIFLINIPLVSLVLVAVLLLVKKDETTARNPVDLPGALLFTAAPLLLIFGVNELGEDEPRLPLAVGSLLAAAVCAAAFVAVERRTAHPLVPLTFFGNRVRLVANGATVLLSAALSTSFFLLTMHLQEERDLSPIEAGLSFLPLGLSLILACVLVRGLIERIGTTGAAVLGMALAGPRHRLFALLPSDNSLLTSVFPGMILLLRMATGLVALQNAALHAVTEADAGVASGVQRCADQLGGASGIAVYVSIGFSPHLGGDWDPFTVAYSLAGIGLIAAVLAVLALSPDRRLAAPREQED

>P46105_MFS

MSSVEADEPDRATAPPSALLPEDGPGPDGTAAGPPPYARRWAALGVILGAEIMDLLDGTVMNVAAPAVRADLGGSLSVIQWITVGYTLAFAVLLVVGGRLGDIYGRKRMFVVGAVGFTAASVLCSVAAGPEMLTAARFLQGGLGALMIPQGLGLIKQMFPPKETAAAFGAFGPAIGLGAVLGPIVAGFLVDADLFGTGWRSVFLINLPIGVAVIVGAVLLLPEGKAPVRPKFDVVGMALVTSGLTLLIFPLVQGRERGWPAWAFVLMLAGAAVLVGFVAHELRQERRGGATLIELSLLRRSRYAAGLAVALVFFTGVSGMSLLLALHLQIGLGFSPTRAALTMTPWSVFLVVGAILTGAVLGSKFGRKALHGGLVVLALGVLIMLLTIGDQAGGLTSWELVPGIAVAGLGMGIMIGLLFDIALADVDKQEAGTASGVLTAVQQLGFTVGVAVLGTLFFGLLGSQATASVDDGASRARTELAAAGASTTEQDRLLADLRVCLRESASQQDSERTPDSCRNLQQARPAVAEATARAWRTAHTENFSTAMVRTLWVVIALLAVSFALAFRLPPKPREEEGF

>D0ZXQ3_MFS

MFRQWLTLVIIVLVYIPVAIDATVLHVAAPTLSMTLGASGNELLWIIDIYSLVMAGMVLPMGALGDRIGFKRLLMLGGTLFGLASLAAAFSHTASWLIATRVLLAIGAAMIVPATLAGIRATFCEEKHRNMALGVWAAVGSGGAAFGPLIGGILLEHFYWGSVFLINVPIVLVVMGLTARYVPRQAGRRDQPLNLGHAVMLIIAILLLVYSAKTALKGHLSLWVISFTLLTGALLLGLFIRTQLATSRPMIDMRLFTHRIILSGVVMAMTAMITLVGFELLMAQELQFVHGLSPYEAGVFMLPVMVASGFSGPIAGVLVSRLGLRLVATGGMALSALSFYGLAMTDFSTQQWQAWGLMALLGFSAASALLASTSAIMAAAPAEKAAAAGAIETMAYELGAGLGIAIFGLLLSRSFSASIRLPAGLEAQEIARASSSMGEAVQLANSLPPTQGQAILDAARHAFIWSHSVALSSAGSMLLLLAVGMWFSLAKAQRR

>Q99S97_MFS

MRLKSIITVIALILIMFMSAIESSIISLALPTIKQDLNAGNLISLIFTAYFIALVIANPIVGELLSRFKIIYVAIAGLLLFSIGSFMCGLSTNFTMLIISRVIQGFGSGVLMSLSQIVPKLAFEIPLRYKIMGIVGSVWGISSIIGPLLGGGILEFATWHWLFYINIPIAIIAIILVIWTFHFPEEETVAKSKFDTKGLTLFYVFIGLIMFALLNQQLLLLNFLSFILAIVVAMCLFKVEKHVSSPFLPVVEFNRSITLVFITDLLTAICLMGFNLYIPVYLQEQLGLSPLQSGLVIFPLSVAWITLNFNLHRIEAKLSRKVIYLLSFTLLLVSSIIISFGIKLPVLIAFVLILAGLSFGYIYTKDSVIVQEETSPLQMKKMMSFYGLTKNLGASIGSTIMGYLYAIQSGIFGPNLHNVLSAVAVISIGLIVLWVVFFKEQSSQSKE

>P96712_MFS

MDTTTAKQASTKFVVLGLLLGILMSAMDNTIVATAMGNIVADLGSFDKFAWVTASYMVAVMAGMPIYGKLSDMYGRKRFFLFGLIFFLIGSALCGIAQTMNQLIIFRAIQGIGGGALLPIAFTIIFDLFPPEKRGKMSGMFGAVFGLSSVLGPLLGAIITDSISWHWVFYINVPIGALSLFFIIRYYKESLEHRKQKIDWGGAITLVVSIVCLMFALELGGKTYDWNSIQIIGLFIVFAVFFIAFFIVERKAEEPIISFWMFKNRLFATAQILAFLYGGTFIILAVFIPIFVQAVYGSSATSAGFILTPMMIGSVIGSMIGGIFQTKASFRNLMLISVIAFFIGMLLLSNMTPDTARVWLTVFMMISGFGVGFNFSLLPAASMNDLEPRFRGTANSTNSFLRSFGMTLGVTIFGTVQTNVFTNKLNDAFSGMKGSAGSGAAQNIGDPQEIFQAGTRSQIPDAILNRIIDAMSSSITYVFLLALIPIVLAAVTILFMGKARVKTTAEMTKKAN

>P11545_MFS

MTTVRTGGAQTAEVPAGGRRDVPSGVKITALATGFVMATLDVTVVNVAGATIQESLDTTLTQLTWIVDGYVLTFASLLMLAGGLANRIGAKTVYLWGMGVFFLASLACALAPTAETLIAARLVQGAGAALFMPSSLSLLVFSFPEKRQRTRMLGLWSAIVATSSGLGPTVGGLMVSAFGWESIFLLNLPIGAIGMAMTYRYIAATESRATRLAVPGHLLWIVALAAVSFALIEGPQLGWTAGPVLTAYAVAVTAAALLALREHRVTNPVMPWQLFRGPGFTGANLVGFLFNFALFGSTFMLGLYFQHARGATPFQAGLELLPMTIFFPVANIVYARISARFSNGTLLTAFLLLAGAASLSMVTITASTPYWVVAVAVGVANIGAGIISPGMTAALVDAAGPENANVAGSVLNANRQIGSLVGIAAMGVVLHSTSDWDHGAAISFLAVGLAYLLGGLSAWRLIARPERRSAVTAAT

>P39642_MFS

MKQLKPNSKYLLYGQALSFMGDYCVLPALLILSTYYHDYWVTSGVIVVRSIPMVFQPFLGVLVDRLDRIKIMLWTDIIRGIIFLGLTFLPKGEYPLIFLALLFITYGSGVFFNPARLAVMSSLESDIKSINTLFAKATTISIIVGAAAGGLFLLGGSVELAVAFNGVTYLVSAFFISRIKLQFVPIQSENIKEAFQSFKEGLKEIKTNSFVLNAMFTMITMALLWGVVYSYFPIVSRFLGDGEIGNFILTFCIGFGGFIGAALVSKWGFNNNRGLTYFTVLSIVSLALFLFTPIFAVSVIAAILFFIAMEYGEVLAKVKVQENAANQIQGRIFSVAEASIGLCISIGSMFINILSAPVIMGLIVVIVCGLFLHTKLVNKSFLERDNKTEQKGVF

>P42670_MFS

MARKPDISAVPVESAACQGPDPRRWWGLVVILAAQLLVVLDGTVVNIALPSVQRDLGMSDTSRQWVITAYTLAFGGLLLLGGRVADAFGRRRIFAVGILGFGLASLLGGAAPDPGTLFLARALQGVFAAALAPAALALINTLFTEPGERGKAFGVYGAVSGGGAAVGLLAGGLLTEYLDWRWCLYVNAPVALLALLGCRLLPRDRRTGRAVRLDLPGTLLGCGGLVAIVYAFAEAESGWGDPLVVRLLVLGVLMLVAFALVERRVQDPLLPPGVVAHRVRGGSFLVVGLPQIGLFGLFLFLTYYLQGILDYSPVLTGVAFLPLGLGIAVGSSLIAARLLPRTRPRTLIVGALLAAAAGMALLTRLEPDTPQVYLTHLLPAQILIGLGIGCMMMPAMHTATARVAPHEAGAAAAVVNSAQQVGGALGVALLNTVSTGATAAYLADHGTSPAATVDGTVHGYTVAIAFAVGVLLLTAVLAWVLIDSRTEAADETGSASVTPARPR

>A0QYL8_MFS

MSAPQAAIDTDHADRHGPRRAWAAVGVLALVGTLNYVDRFLPSVLAEPIKHDLELSDTAIGVINGFGFLIVYAVMGIAVARVADRGAFGAVVAGCLTLWGTMTMLGGAVQSGFQLALTRVGVAIGEAGSTPAAHAYVARNFVPQRRSAPLAVITIAIPLASTASLLGGGLLAQSLGWRTAFVIMGAVSVVLAPLVLLVVGVRQSLPAAPAVVDKTAGGWWNLLRKPSFLIVVAGTAFISAAGYSLTTFSPAFLMRTRGMSLGEVGVEYGLATGAIGVLGLLIVGRLADRLAERDPRWLLWIVVTLTLVLLPASVLAFVVEDRMLCVLFLALSYAIGTSYLAPSIAAIQRLVLPEQRATASAMFLFFNAVFGSVGPFVVGMLSDSLTDDLGAQALGRALLLLVAAMQLVGAICYWLASARYRRDIIEEAR

>A0QWU7_MFS

MSSRGNRNIAISAGSLAVLLGALDTYVVITIIVDIMADVGIAINQIQQVTPIITGYLLGYIAAMPLLGRASDRFGRKMLIQVGLAGFAVGSVVTALSSDLTMLVIGRIIQGSASGALLPVTLALAADLWSARSRASVLGGVGAAQELGAVLGPMYGIALVWLFNHWQAVFWVNVPLAVIAMVMIHFSLPARQQVDEPERVDVIGGVLLAIALGLTVVGLYNPEPDGKQVLPSWGLPVLAGALVAAVAFFAWEKVAKTRLIDPAGVRFRPFLAALAASLCAGAALMVTLVNVELFGQGVLGQDQDHAAFLLLRFLIALPIGALIGGWLATRIGDRLVVLIGLLIAAGGFVLISHWSVDVLADRHNLGLFTLPVLDTDLAIVGLGLGLVIGPLTSATLRAVPAAEHGIASAAVVVARMIGMLIGIAALGAWGFYRFNQHLATLAARAAGDAGSPMSLAERLTAQAVRYREAYVMMYGDIFLSAAVVCVIGALLGLLISGKHEHAEEFEPAYAPTYGGGGAIDPYDAGDADDAPTEMLDLPTQVLSAPPSDPGDERPGRHRAP

>A9MWE8_MFS

MNENIAEKFRADGVARPNWSAVFAVAFCVACLITVEFLPVSLLTPMAQDLGISEGVAGQSVTVTAFVAMFSSLFITQIIQATDRRYIVILFAVLLTASCLMVSFANSFTLLLLGRACLGLALGGFWAMSASLTMRLVPARTVPKALSVIFGAVSIALVIAAPLGSFLGGIIGWRNVFNAAAVMGVLCVIWVVKSLPSLPGEPSHQKQNMFSLLQRPGVMAGMIAIFMSFAGQFAFFTYIRPVYMNLAGFDVDGLTLVLLSFGIASFVGTSFSSYVLKRSVKLALAGAPLLLALSALTLIVWGSDKTVAAVIAIIWGLAFALVPVGWSTWITRSLADQAEKAGSIQVAVIQLANTCGAAVGGYALDNFGLLSPLALSGGLMLLTALVVAAKVRITPMS

>P45123_MFS

MNQQKSTFIFILTLGILSMLPPFGVDMYLPSFLEIAKDLDVSPEQVQHTLTSFAYGMAFGQLFWGPFGDSFGRKPIILLGVIVGALTALVLTEINSVGNFTALRFVQGFFGAAPVVLSGALLRDLFSKDQLSKVMSTITLVFMLAPLVAPIIGGYIVKFFHWHAIFYVISLVGLLAAALVFFIIPETHKKENRIPLRLNIIARNFLLLWKQKEVLGYMFAASFSFGGLFAFVTAGSIVYIGIYGVPVDQFGYFFMMNIVTMIFASFLNSRFVTKVGAETMLRIALAIQFLSGMWLILTALLDLGFWPMAIGVAFFVGPNPVISSNAMASALERCPQMAGTANSLIGSVRFAVGAIMGSLVASMKMDTAAPMLFTMGACVVISVLAYYFLTSRNLKSRG

>C5BC70_MFS

MQNHLSSTRRLGRRALLFPLCLVLYEFATYIGNDMIQPGMLSVVQTFGVDESWVPTSMTAYLAGGMFLQWLLGPLSDRIGRRPVMLIGTLYFAATCLAILLTNSIEQFTLMRFLQGISLCFIGAVGYAAIQESFEESVCIKITALMANVALIAPLLGPLAGAAWVHLFPWEGMFILFAALSLLAFLGLYKAMPETATRRGEKLSLSALGRDYTLVLKNRRFLCGSLACGFASLPLLAWIAQSPVIIISGEGLSSYDYGMLQVPIFGMLILGNLTLARLSGRRPVRRLIQLGAWPMVGGLAIAAASTLYSAHAYLWMTAGLSLYAFGIGLANAGLYRLTLFSSTMSKGTVSAAMGMISMFIYTLGIEVGKYAWLLGGNGAFNLFNLISGLLWLALIARMLRDQLVGRMAGR

>757424.7.peg.1547_RND

MTNTPNPHPPSPSNASARGSILRRWWFWVLVAALAAGGGYKMWSKKKAEQEQMAAMGGPGGRPGPGAAGARRPGGPGAFGPQTMPVGVAKARLQDVNVFLNGLGAVTPTATATVRARVDGQLMKLHYKEGQVVKAGDLLAEIDPRSLQAALTQAEGQLARDRALLASARLDLKRYQTLLAQDSIASQQVDTQVALVKQYEGTVKADEGNVASARLQLSFTRVTAPISGRLGLRQADVGNNVTTSDTNGLVIITQLQPITAIFSIPEDNIPKVLQQLQSGRKLPAQAWDREQKNKLADGVLLTIDNVVDATTGTVKLKAQFPNTDYALFPSQFVNIRLQLNTEQGATVIPTAAIQRGSKGLFVYVVKDDSSVTVRPVKTGPVQDDLTVITDGVSAGETVVIDGIDRLREGAKVEAVARGGADDPANKLTTENPERRHGKRGQGNPGAQAGAGGDAGQGAQGGMSPEERQKRWAELNKRIDAGEFGEEIKKLPEDQRRQKMMELRRQREAAGNGNGNAAK

>757424.7.peg.2067_RND

MKLAQLRRPKFIFLALLVLLIAAWIIRSVLTPPAPPTYLSATARVADIQDVVLASGTVKAYKQVSVGAQVSGQIKSLKVALGDQVKKGQLVAEIDSLTQANALASAEFSLQNLQAQLRAKEASLKQAQLAYARQKMMLAGDASSRENFESAEATLNTTQADIAALQAQIKDGAIKVDTARLNLGYTRISSPIEGQVVAIVAQEGQTVNANQSTPTIIKVARMDTVTIKAQISEADVVRVKPGQPVFFTILGDPDHRYRTTLRAIEPAPDSILQDDTSSSTTSITSSSSASSTAIYYNGLLDVPNPDGKLRISMTTQVNIVLSEASNALVLPSTALGAKAADGSYTVRVLDDQGQAHERKVRIGINTNALVQIVEGVKAGERVVTGTVLPGAAASSSAHDGPPPHM

>757424.7.peg.2754_RND

MNRINSSAFRLRAIAAVAALAGVLGLSGCASFAGIGSDRQVAQAGDFATQRSLSDPNPGAPNGQWPGSDWVRQFGDAQLVALVEQALTSSPSLQQARARIAAASALAESRGAPLLPSVNAEASVTRNQFSSTTIYPPPYGGNWYNEKKAGLNVGYELDLWNKNQAALAQAISSEKAAQASEQEARLALTASIVTVYSQLAAQYALHDILQSTVDQRTSLEKITAERLRTGLDSQIERDQSRTSSADARAQLAQSEGQIVLLRQQLGALAGKGPDYGLQLAPPALQGLATPGLPAELPLNLMGRRPDIVAARWQVEAASRGVDVAKARFYPDINLSAMIGFDTLLDSNPFTAASKSIAFGPAITLPIFEGGALRAGLKGEYASYELAVATYNKTLNDAYADVARQIAAIHATERQLPIRSEALQAAERAYALARERYRLGLVSQLTLLSAQTGVLAQRQAMVALQAQRRDQQVALYKALGGGFDAQRDGLAYGAQP

>757424.7.peg.3310_RND

MAGCASFSDLGERAQPKSIDRYQSQQSLAASAVQAAWPSDQWWRVYGDAQLNALIDEALQSAPSMAVAKARLMKAEGAAQQQGAALYPQVSANASLDRMKQSYNNGVPPDFVPKDYNNATRATLDFSYEIDFWGKNRAALAAATSELEASRADAAQARITLATSIASAYAELAQLYAQRDTNEAALKVRVESLDLFNQRFTNGLETRGSVKQMEARRAIAQADLKATDESIGLQRNKLAALLGAGPDRGLQLTRPQIDLSRPFALPAQLPVELLGRRPDIVAARLRAEAAGKQIKVARAAFYPNVNLTAYFGFQSLGIDMLTRAGSDIGSIGPAISLPIFNGGRLRGQFRSASASYDEAVANYDQAVTQALQDVADVGVSEKALAGRLADVQAAADAAEEAYRIVSNRYNGGLATYLDVLNAQDTLISNLRQLSDLRSRMFTLDVALVRALGGGYRAADDSSSQNSADAPLHDTQAKG

>1006551.4.peg.3864_RND

MSSAASCPAATNTGIKIMSLQKYWGNFHLTVPGVMLLSALLVGCDEGVAQNAAPQAPAVSAADVVVKSISQWDSFNGRIEAVESVQLRPRVSGYIDKVNYTDGQEVKKGEVLFTIDDRTYRAALEQAQATLARAKTQASLARSEANRTDKLVNTNLVSREEWEQRRAAATQAQADIRAAQAAVDAAQLNLDFTKVTAPIDGRASRALITSGNLVTAGDSASVLTTLVSQKTVYVYFDVDESTYLHYQNLARSGQGASSNHLALPVEIGLVGEEGYPHQGKVDFLDNQLTPSTGTIRMRALLDNAQRQFTPGLFARVRLPGSAEFNATLIDDKAVLTDQDRKYVYVVDKEGKAQRRDITPGRLADGLRIVQQGLKPGDRVIVDGLQKVFMPGMPVNAKTVAMTASTALH

>1069631.3.peg.2211_RND

MLFSLFVTIGLNVYLYAVVPKGLFPQQDTGQLMGFFRVDRGTSFQSMVPKLEYFRSILNQDPDIRSVAVFAGGRSGSTSSFILVELKPMDERKASTTDVVNRLREPLSTTPGARMFMVPQQDIPVGSGGGGRSGSYDYSLLGSDLELLKTWLPKVQQAMAELPELVDVDTGTDDKAGLVQLEIDRDMATRLGIDMSMVAGTLNNSFSQRQVSTIFGRLNQYYVVMEVEPRFAQDLESLKEIEVVAKDGTRVPLSAFTRFTTGTAPRSINHMGLLVAESVSFGLAEGVTLSQATAAIEQAMARIQLPTREIQAGFEGNTAQMLDALAKQPMMFLAAWWLCISCWGCCTRAICTRSPFCPLCLRPGLGHCWL

>1120928.3.peg.3378_RND

MSISRKQLTLSAVIVAIFATGGSFILFQEKADAKATPTASAAPAATVDVANVISQTITDWQEYSGRLEAIDQVDVRPQVSGKLIAVHFKDGSLVNKGDLLFTIDPRPFEAELNRAKAQLASAEAQVTYSSANLGRNQRLIQSNAIAHQELDQAENEARSANANLQAAKAAVETARLNLEYTRITAPVSGRISRAEVTVGNVVSAGNGAQVLTSLVSVSRLYASFDVDEQTYLKYISNQRNSAQVPVYLGLANESGFSREGFISSIDNNLNTTSGTIRVRATFDNPKGVMLPGLYARIRLGGGQPRAAILISPTAIGVDQDKRFVVVVDAKNQTAYREVKLGAQQDGLQIINSGLQVGDRIVVNGLQRIRPGDPVSPHLVSMPNPQIITDNTAQQPQPTEKTPTSAKG

>1154758.3.peg.1457_RND

MIVATVGLFVLSVVMFKFVPQQFFPASGRLELMIDLKLAEGASLTNTAEQVKRLEQMLKDHQGIDNYVAYVGTGSPRFYLPLDQQLPAPSFAQFVVLARSIEDREAIRGWLISSLNEQFPTLRSRVTRLENGPPVGYPVQFRVTGEHIEVVRALARKVQDRVRENPHVANVHLDWEEPSKVVHLNIDQDRARALGVTTADLSAFLRNSLTGSSVSQFRDDDELIDILLRGTRNEREQLGALSSLAIPTQNGTSVALSQVATLDYGFEEGVIWHRNRLPSVTVRADIYGKGQPATLVKQILPTLDSVRAELPDGYLLEVGGTVEDSARGQNSVNAGMPLFIVVVLTLLMIQLRSFSRMLMVFITAPLAADRRHAVPADLQSAVRLCGHAGHHRAVRNDHAQFGDSGRSDRAGHHRRAGPMARDHRCHRAPLPPDRADCSGRSAGDDTAVTQPVFRPDGRGYHGWSDRRHGADPAVPARAVCRVVQGETGG

>1161913.3.peg.4313_RND

MALGTLVLTVVLYIFIPKGFFPVQDTGVIQGISEATQSVSFGAMAERQQALAKVVLEDPAVESLSSFIGVDGINATLNSGRMLINLKPHESRDISASDVIRRLQPRLNEKVPGITLYMQPVQDLTIEDSVSRTQYQFTLEDADAAELSTWVPKIVDRLRQLPELADVATLHVLDVLDDAVALVVHQHDDHVGLFLHGGRQLTQVEDEAAVAGQREGLLARGGHRCADGGADAHRQALADAAAECMHAGQRIENAQIAIAPGAVRHGDVAHPVELAAGGLLYLLNQRAVGTETVDQAGDGGIARLFQVGHEGRIDVDCALAFFEAIRQAFQRQCSIAADEVVAVVAAAFRRWIGVDAIQRTRQLQFVLQGFVAAQARADHDDGVAGLVEVLDRLVQVE

>1194405.4.peg.3028_RND

MIVSQGGAARDRLIERLRQRFRDDYVGVGGXRPWRWPPFSTPTPISVRSSTTGTNRARC

>1208660.3.peg.3555_RND

MTFTDLFVRRPVLALVVSTLILLLGLRATGELPVRQYPLTENTTITIITQYPGASPELMQGFVTQPIAQAVATVENIDYLSSSSTQGRSLITVRMKLNADSNKALTEIMAKVNQVKYRLPQEIYDPVLAKSSGEATSVAYVGFSSKTMPIPALTDYLQRVVLPQLSSIDGVASVDLYGGQTLAMRVWLDPARMAARGISAGEIAQALRDNNVQAAPGQTKGLYVVSNIQVNTDLNSLTDFRDMVVRQVDGAIVRLGDVGTVELGAASYDSSARMDGEKAVYFGLNATPVGNPLTIVERINALLPGIKQNLPPGVEVQVPFELARFINASIDEVRNTLLEAVLIVVAVIFLCLGSLRAVLVPVVTIPLSMLGAAAIMLSLGFSINLLTLLAMVLAIGLVVDDAIVVVENVHRHIEEGKSPVHAALVGAREVAGPVIAMTFTLAAVYAPIGLMGGLTGSLFKEFAFTLAAAVGVSGVIALTLSPVMSSFLLNSRVSEGWMARKAEHFFQRLGDAYGRVLDVSLRHRWVTGLIAVVVLASLPVLYGSAQRELAPVEDQAMILTAVKSPQHANIDYVEKFGQKWDTVMQEIPEQNGRWLINGSDGVANSIGGVNLVTWQARKRSADEIQGDLQNRVNAIEGSNTFAFQLPSLPGSTGGLPVQMVLMSAADYRVVYDAMETLKHAARASGLFMVVDSDLDYNNPVVRVDIDRAKANSLGVTMKAIGDTLAVLVGENYVNRFGMDGRSYDVIPQSPRGMRLTPQSLGQFYVKSASGAQVPLATLVKISMGVEPNRLTQFDQLNSATFQAIPMPGVTMGDAVQFLTEQARLLPPSFSHDWQSDARQYSQEGSALVVTFLFAIIVIYLVLAAQYESLRDPLIILVSVPMSICGALIPLALGMATINIYTQIGLVTLIGLISKHGILMVEFANEMQAHAGLDRRAAMERAARIRLRPILMTTAAMVVGLVPLLFASGAGAHSRFSLGLVIVVGMLVHPVHPVRAAHHVHPAGARPPRRRPIRARPRTGAAGRPGGRRLPRTRDRIMTHPVPTTFARTAGALLAALALAGCAVGPQYQAPTPAPVKLASPEQALFSADLLQREWWRQLQDARLDALIGLALARNLDIRQAQARLREARAALDEKELDRWPTVTAAGGYTRSLSQINPGPDQRNLAQSYRAGFDATWEIDLFGRLQRRAEAAAARDQAAAADLAQTRLVVVAELARNYFEMRGAEQRLAVARANLATQQETLRVTAALVETGRGYAGDLASARAELAGTRALLAPLETQRRLAQYRIAVLAAMRPAELGELRQEQPLAPLAAQLPIGDVAMLLQRRPDVRAAERLLAATNADVGAITAELYPRIDLGGFLGFIALRGGDLGQASSKAFALAPTISWPALHLGSVQAQLRAGQARHDAARARYEQVALQAIEEVEGALTRYGQNQQRLRDLLDSATQSQRAADLAQTRYREGAAPYLTVLDAQRTLLRAQDAVAQSESESYTSLVALYKALGGGWNTDAAAPARSARTAALPASP

>1218169.3.peg.6920_RND

MRRYNFLSAAGEVKGEYVVTSINASTELKSAEAFAALPVKTSGDSRVLLGDVARVEMGAENYDTVSSFDGTPIGVHRHQGHTSRQPAGRHQGSAAHHARAGKPAALGAEGIDRL

>1224163.3.peg.561_RND

MFRTEADVMRAAAGNVDDTNTSVQGELKRLQNVVDTVRGSWAGTAQVSFDNLMIRYNESARDLHEALASIADNIRSNAVGFEDMEATNAQSFDRVGAQGLAL

>1231351.3.peg.1176_RND

MNLSRPFILRPVATTLLTLGLVISGLLGYSQLPVADLPNVDMPVIMVQAQQPGGSPSEIASTIAEPLERHLGAIAGLTEMTSQSMVNQVRILLQFDLARDVNGAARDVEAALQAARQDLPAGSLRSNPTYQKANPNGAPILVLALTSKTRTPQAIYDFTTNVVQQQLSEIRGVGGMEIGGGALPAVRVELNPLKLYKFGIGFEDVRAALVSANAHTPKGFIEQNGQRFTLDTNDQATQAQAYRNLVIAYRDNAAVRLSDVSIVRDSVENLRTSGYFNGERAVIALVFAQAGANVVQTIDQIKQRFDLIRAALPPDIELHLAVDRSQTIRAALDDTKLTLIIAVVLVVLVVLLFLRSLPAIMIPAIVVPTSIIGTFGAMRLLGYQLDNMSLMALTISTGFVVDDAIVVLENVSRYLEQGVAPVPAALRGAGEVAFTVISITVSLIAVFIPILLLGGLPGRLFHEFAITITLTLVISMGLSLSLTPMICALLLKPMPSGETRGRVSHAIERGLSAVTRGYAASLEWSLHHQWLMVLSLPATLVLAGALFVEMPKGFFPTEDTGLLMGHLVGDETSSFGQMSQRAQLGTRIMAHDRDIANVVGFVGGRQANTANLFSSLKPKSERNDTVLQTIVRITRHFRGMVGTQFYLMQPGAVRAGARGGNGAYQYSLQGPDADELYAWTPKVVAAFQRLPELMDVSSDLDEGGAALDVRIERPTSARVQITPQLISNILYDAYGQRAASVIYRSNNQYRVIMEAAPRFWHDPHSLYQTWISVSGGTAAGGTASNNIRARLTTTTSSGSSDTTSSASSQAAQSYQNQMANSLAGGSNASSGAAVTTSAETMVPLTIVSRITPGVTSLSVNHQGQSVATTVSFNLRPGVSLGPAIAAINAALVKMHMPTEIRGGFAGNAAQFQKSVSAEPLIILAALITVYVTLGVLYESLVHPLTILSTLPSAGVGAILALQVFREEFSLIAMIGVILLIGIVKKNAIMLIDFALQAQRAGSSAYDAIHEASLLRFRPIIMTSLAAALGAVPLIVANGYGSELRRPLGIAILGGLVVSQALTLYTTPAIFLMLERAREATHRAVRSFRRPHQQDIPST

>1267562.4.peg.5000_RND

MPADPTFAASPSPAAPVSSLPCRLRATSLVLLTALTVAACGRGEAPAAARTPEVAYVTLQPQPVTLSTELPGRTVAYRVAEVRPQVDGIILKRLFKEGSEVRQGQQLYQIDPSTYQAAHASAAATLESARQTAQRYERLARERAVSQQEYEQARAAWLTAQAAVDRAAIDLRYTRVLAPISGRIGRSFASEGALATNGQANALATVQQLDPIYVDVTQPSSALLGLRRDLAAGRLEAAGENAARVRLILEDGSEYAEPGRLEFTEVGVDTGTGSVTLRAVFPNPRHELLPGMFVRARMQQGVRPAAMLAPQRGVTRDAKGQATALLVNANDEVELRRIDAERVIGDNWLVSGGLQPGERLIVDGLQFVRPGMKVRALPLAGAAPASAPASAVAAPRRAPPSRSVERSAPMSRFFIERPIFAWVIALVIMLAGALSIGALPVSQYPAIAPPTIAIQVNYPGASAQTVQDTVVQVIEQQLNGLDRLRYISSESNGDGNMTITVTFEQGTNPDIAQVQVQNKLQLATPLLPQEVQQQGIRVTKSVRNFLMIVGVVSSDGSMTREDLANYIVSNIQDPLSRTPGVGDFQVFGAQYAMRIWLDPARLTAYQLTPSDVRAAIQAQNVQVASGQLGGLPSVAGQQLNATVVGKTRLQTPEQFREILLKVNGDGSQVRLKDVAEVGLGGQDYNINAQYNGRPASGIAIRLASGANALDTAKAIRATLGELEPFFPPGMQVVYPYDTTPVISASIEGVVRTLLEAVVLVFLVMYLFLQNVRATLIPTIAVPVVLLGTFGVLAAFGYSINTLTMFGMVLAIGLLVDDAIVVVENVERLMAEEGLPPKEAARRSMGQIQGALVGIALVLSAVFLPMAFFGGSAGVIYRQFSITIVSAMVLSVLVALVLTPALCATMLRPLPKHGDGHGHGAPRRGPLGWFNRGFEAATRGYERGVVAVLNRRGRYFAVYLLILALAAWMFTRIPTSFLPDEDQGVLFAQVQTPPGASAQRTQQVLDRLRDYLLQEEGGVVQSLFTVNGFNFAGRGQSSGFAFVLLKPWHERIGEATSVFDLARRAQARFSGMRDAMAFAFVPPAVMELGNATGFDVYLQDRAGVGRGVLMQARDRFLQLAAQRPELQRVRMNGLNDEPQYRLEIDDEKARALGVSLAEINSTVSIAWGSSYVNDFIDQGRVKRVYLQGRPDARMHPDDLAKWFVRNDRGAMVPFTAFASGSWGHGSPKLQRYNGVAAIQILGEPAPGHSSGEAMAAVEAIMAQMPAGVGHSWSGLSYEERLSGAQAPALYALSLLVVFLCLAALYESWTIPFSVMLIVPLGIVGALAATLLRGLPNDVFFQVGLLTTMGLSAKNAILIVEFAKALHDQGKGIVEAAIEASRMRLRPIVMTSLAFVLGVVPLATSVGAGSGSQHAIGTGVIGGVITATVLAIFWVPLFYVAVHRWFGGRRGTPASEISTRTA

>1343158.3.peg.407_RND

MVFCRLFIDRPVATTLLALAIFLSGMIALPFLPISTMPDMTATSIMVIANQPGSDPQQMATSVSTPLERRLATIADIQTLESVTTRGQTSIFLDFSSSRNINGALRDVQAALHAARSDLPTSTLEADPQAFKLDGDKPIYLLHLTSDQLPRAQLYDLATIRVRPILAQIAGVGRVELFGASNPAVRVELNPYPLYRWGLNPEDVRAALASANAFTPKGFITSGNQRIQLQTNDQATEAAHYRDLIVAYRNGKNPIYLKDIATVRDDVQDVYQNSTLNGKTAITIAVIPQPHANAVEIVNDIVRRLPRLQQALPASAELRTGLDLSLTIRASLADAKQTLVISIFLVVLVIALFFRHMASTLIPAITIPVALSGTLTAMAWFNFSLNILSLMALTIAVGFVIDDAIVVLENIARHMENGMNRYQASIVGTSEIAFTIISISLSLIAVFIPLLCIPGTLGSALHEFALTMAATIAISMVLSLTLTPMLCAHFLTIEPAGGTPAIPERPRYSPLADPVSWLLYGAMRTVRAVETGLYHLTSLYDRSMHWSLRHPIIIGLTLPGSFLLMVGIIILMPKTAIPSMDLAILQGSINGEPSLSFKALTRRMHQVESIIQKDPAVQTVVTFNRTSHTGRIFVTLKAKSMRDSIPVILARLRKAIPQQAGAEAFFWALNNGRQGGGDSNTTGNYRYVLQSDSNGPLYATMPPLLAQLRASGKFRNLSTDAEDLSFFANVLIHRDLEARYNITPQLVQNALFDAYGQSIVSTIHLPLTNHRVVMVVAEPFREYSNTLHHLWLSTSAGTAAGGIASNLIRVRTKGTLSTQASLSRDSVTNSLANKLSGNSSNGAAVSSSQETMIPLDNVASIVKTPMPLSITHHNGYYATTLSFDLAEGTSYDDAISLIHRALVNLHASDSIHGEFTGTTGETTDLMLNALLAFLAAITIMYIALGVLYESLLHPITILSTLPSAGVGGVLGLWASGEQFSLVAIIGVILLTGLVKKNAILVIDFALHIHHHHPDMTAEETIRHASVTRFRPILMTTLAAALGGIPLLMSQGYGCELRRPLGVAILGGMAISQLLTFYTTPAVYLLMEKLKHHSLSLMRRVRAAL

>1385369.3.peg.6651_RND

MTGFNLSEWALRHRSFTWYLIISLTLAGGIAYTRLGREEDPAFAIKTMVVQTVWPGATIDDMIDLVTDPIEKKLEEVSYLDYVKSYTRPGFSVVYVNLKDFTPAGEIPDLWYQVRKKIADMKGTLPQGVQGPAFNDEFGDTFGTVYAFTADGFSYRELKDYAETARAELMRVPDVGKIQFVGIQNEKIYLDFSTRQLAALGIDRNQIVAELQAQNAVAPAGVVQAGDEKVTVRVSGEFTSEESLKAINLRAGGKFYRLADLAQVRRGYADPPSPIFRYNGEPAIGMIISMAAGGNVLDFGKDIQERMRQVEANLPVGINTHLVANQSVVVDHSVAGFTKALKEAVVIVLVVSFISLGIRAGIVVACSIPLVLAMTFIGMEYYGIALQRISLGALIIALGLLVDDAMITVEMMITKLEEGFSLDKAATFAYTSTAFPMLTGTLITVAGFIPIGFAQGGAAEYCFSLFAVVAMALLFSWIVAVMFAPLIGVKVLRPPKPGKGHSGHGEPGRMMRAFRASLRLAMRARYIVIVLTVALFGLSVFGLRFVQQQFFPASDRAELLVNLTLPQTSSIKATEEVVNRFEKVLAADPEIESWSFYIGQGAIRFYLPLDVQLANDYFAQAVVVTKGYDVRDGVRARLEKVLNEDFSDLSTRVSPLEMGPPVGWPIQYRVSGPDVGEVRDAAYRLADTIGANPYTLLINYDWNEPSKVVRVDVEQDKARQLGISSKSLSEALNATVSGAVFTQVRDGIYLIDVVAQASNAERSSIETLRNLQVALQDGRTVPLREVAILRYDLEQPLIWRRERLPTITVQADLVPPLQAPTIVNQLAPVVDELRRSLPPGYSIEVGGTVENSAKGMTSIVAVFPIMIFVMLTILMIQLQSFQKLFLVISVAPLGLIGVVAALAPTGTPLGFVAILGVVALIGMIVRNSVIMIAQIDEHLEAGEHPWDAVINATMHRVRPILLTAAAASLGMIPIAPEVFWGPMAYAIIGGLVVATALTLLFLPALYVAWFRIKEPGHEKTIDGKTPVEAESPGHRPIAGPYGPMPPAGVVSGD

>1439940.3.peg.2461_RND

MRFNLSAWALHNRQIVVYLMLLLAVVGALSYSKLGQSEDPPFTFKAMVIQTQWPGLPPRKCRARSPSASRRS

>1469502.3.peg.806_RND

MNLSAPFIARPVATTLITIAIALAGVLGLETIPVSPLPQIDFPTILVQAVLPGASPETMASTVATPLERKLGLIAGVDEMTSVNSLGMTRISLQFDLHRDIDGAARDVQAAINAARAVLPPMPVNPKYWKVNPANAPVMILSLTSRSMTRGQMYDAASTVLAQRIAQVSGVGQVRINGSALPAVRIDVDIEKLARMGISLESVHAAVAAANVDSPKGIIETGGRSWLIGANDQTTTAAAYRRLIVAYRGDRPVRIGDVATVHDSVENIRNAGATNGRPSVLLLIYRQPGANILDTVGRVNALLPRLRASIPSAIDLNVDMDRTSTIRASLHEASRSLLLAVLLVILVVFAFLRSARAIWIPAVAIPVSLVGSFAAMKLLGYSLNNLTLMALAIATGFVVDDVIVVLENIVRHLEEIPGDLAEPGRSGTIPAGNAFALDRRRDAVRLAALRGVRKVGFTVLSMSLSLIAVFIPILAMDGLIGRIFREFAVTLSVSILISLAISLTTTPMLCAVLLRPGAAGADRARRPTSGAGGGSAIRGLRNAWIRVARSASNGTRAIGSRASIAYERSLDAALRHPRITLLILAATVAANISLYIAIPKGFLPAEDIGLIKGKVQGDQSISFQSMTRKLDRFMAIVQSDPAVARVNGFTGGDEANSGFVFAILKPFRERGEISPEAVIDRLRSRLAKVPGATLYLQPARDLHFGGRPSNAEYQYTLESDNLDDLQTWGARIRQALSRLPELVDVNSDAQDRGLGTAITVDRDSLSRLGLTMSQVDTTLDDAFGQRQVSTIFAPRNQYHVVEEADPRFLQDSASLVALNLIGPTGSPIPLQAFARWETRDAPLVVNHQGSFMATTISFNLAPGVSLGTAAAAIDRTMARIGVPATIHGGFQGTAKLFRNSLAAEPLLGLLALFAVYIVLGILYESLTHPITILSTLPSASIGAMLAMMVFRIPMTVIAFIGVILLIGIVMKNAIMMVDVAIDLERRDRLDPREAIRRACLHRLRPIMMTTTAALFGAMPLALGGGDGAELRQPLGIAIVGGLLFSQVLTLYTTPVVYLTLDRLRIRLLRLRHRDSGPSGGQRIPGL

>1500894.3.peg.2187_RND

MNLSKPFVNRPIATVLLTLGLALAGIGAFFVLPVSPLPQVDFPAISVTANLPGGSPDTMASSVATPLERRLAVIAGVNEITSQSGTGQTRINLQFDLNRQIDAAAREVQAAINASRADLPSTLRQNPTYRKANPSDAPVIILALTSKTRSPGQIYDEVSNLVQQKLAQVKGVGDVEIGGGSLPAVRVDLIPYQMNNYGVSAEDIRAAIQATNPNRPKGELEGQGQRLQIYSQVNTPTGGRTAADYKGLVVAWRNGAAVRLQDIAEVSDGVEDIHTLGLFNGRPAIIVLVTSQPGANVIETVDGVRALLPQLQAQLPEDVTMRVASDRTNSIRASLREIEFTLMISIALVVLVVSVFLRSVRATVVPAVATVVSLLGTFGVMYLLGFSLNNLSLMALTVATGFVVDDAIVVLENTSRHVEEGMDKVKAALLGAQEVGFTVLSISLSLIAVFIPLLFMGGQVGRLFREFAVTLSVAVMISLVISLTTTPMLCALLLKGDKEDHKHQLKREQSRIGRFFERGFSVVMKSYEHALDWALDSKPLVMLILLFVVGLNVYLFAAAPKGFFPQQDTGQVAGGMRADQSISFQAMQGKLRQLVNIITSDPAVDTVVGFTGGSRAGGGFMFLNLKPVGERAKGESGQAVIARLRPKLAHVTGVQLFLNPVQDLRMGGRQSNSTYQYTLKSDSSADLKKWATRLADAMKAQKGLTDVDTDQADNGVETYVDIDTATAARLGISARDVDNAMYDAFGQRQVANIYDELNQYHVIMGVAQRYAQSPNALNDVYVPVSSAGAPGTTGTTSGTGSATQGTGGTGTSTASGAGSVTSSVSASVSTSASTSVSSGSAGSAAPATQNAGGAGAGNGSALGTLGAGAASSGGGGSPGSTTNLTAARDPSSGSALSTSAKTMVPLTTMARFSERSTPSSVNHQDGVVATTISFNLAPGVSLSQAQDQVRAAEAQIGMPTNVRGSFEGQAKQAQESNQQQPLLILAAIVVIYIVLGILYESLVHPLTVLSTLPSAGVGAVLALLMFHMEFSIIALIGIFLLIGIVKKNAILIIDFALDAERARGLSATEAVREACLLRFRPILMTTLAAALGALPLAIGFGEGSELRQPLGIAIIGGLIASQLLTLLTTPVVYVYLDKLRTKKPDEHELARQPVEHPSSVPSHS

>1502724.3.peg.3508_RND

MLFAPLIGVAQLPKVMQGHADKKPSRISGWFRQSLAIAMQFRWATIAFTVALFAVALFGLICVVAALLPTGTPLGFVALLGVLALAGIIIRNAAILIGQINDNLRDG

>1736280.3.peg.4105_RND

MSALSPSRPFIERPVATALLMVAIVLAGLLGFRLLPLSALPEVDYPTIQVQTLYPGASPEVMSRTVSAPLERQFGQMPGLARMASTSAAGVSIVTLQFNLGLALDVAEQQVQAAINAGASLLPTDLPAPPVYAKVNPADAPVLTLAISSETLPLTEVQNLVNTRLAQKISQVPGVGLVTLAGGQRPAVRIQADTKALASYGLGLDTLRTAISAANANSAKGSFDGPQRAYNINANDQLVTADDYQRLIVTWKNGAPVRLSDVARVVDAPENNRLGAWAGTTEPPPGRPKAASAPPGGSEPREAGSVGATEPPPGRPKAASAPSGGSEPREAGSVGATEPPPGRPKAASAPLGGSEPREAGSVGATEPPPGRPKAASAPSGGSEPREAGSVGATEPPPGRPKAASARSGGSEPREAGSVGATEPPPGRPKAASAPPGGSEPSVAGSVGATLTPAIILNVQRQPGANVIATVDGIKRQLPELQAQLPASIQVQVLSDRTTGIRASVEHVQMELVLAVLMVVLVIFFFLHSLRATVIASLAVPISLIGTCGVMYLLGYSLNNLSLMALTIATGFVVDDAIVMIENIARYIEEGEPPFQAALKGATQIGFTIISLTVSLIAVLIPLLFMSDVVGRLFREFAVTLALTILISAVVSLTLVPMMSARWLKAEPAHGSQRGWAGAVQRGFDRVIGRYDGWLQWVLRHQRATLVVAVLTMALTALLYVLIPKGLFPTQDTGQLQARLQASQEVSYARMSELQQAAAQAILQDAEVQSLSSFVGVDAANNTMLNAGRMLINLKPGHDAQAEVMQRLRDRVAGVAGVTLFLQPTQDLTIDTETGPTEYRASIGGVEAAQVNGWTQKLVERLKTVPEVRNATTDAGAQGLSAYVDIDRNTASRLSVTASAVDDALYSAFGQRIVSTIFTETNQYRVILEAQQEQLGSLEGLGTLPLRTGSAAPTPLAAVATIREQLAPLQVTRVAQYPAATLGFDTAPGVSLGRAVSAIRAAAQEIGMPAGLSMEFLGAASAYEKSLTSQLWLILAAMVCVYIVLGVLYESYVHPLTILSTLPSAGVGALLALMLTGNDLGVIGIIGIILLIGIVKKNAIMMIDFAIDAERHQGMGPQQAIHQAALLRFRPILMTTLAALFAALPLMLGWGEGAELRRPLGLAIFGGLVLSQLLTLFTTPVIYLAFDRLGRRWTGRGTAAAPVTHAEAGPAAP

>1736456.3.peg.2622_RND

MPHPNTASAVIYSLMLTCRACGVAPLTWLRHVQTGLPQRDEAADIVDLLLFMAYAMMGGIIVGTVVTLLCQFSTWPGSAYRVKTRRRRRRSLGPLEPKTFRISLFSKACCR

>198822.17.peg.3135_RND

MNSDQQQGGLEAMVTFDRSTAARLGIKPAQIDNTLLTPSASARSRPSTTRCRSITW

>199.248.peg.1235_RND

MIKTAINRPITTLMIFLSLVVFGIYSLKTMNVNLYPQVNIPIVKITTYANGDMNYIKTKITQKIEDEISSIEGIKKIYSTSFDNLSVVSIEFELNKDLESATNDVRDKMQKARVGANYEIEKLNGLSSSVFSLFITRLDGNETKLMQEIDDVAKPFLERISGVSKVKTNGFLEPAVKILLDRFKLDKNALSANEVANLIKVENLKAPLGKIENEQIQMAIKSNFSAKSIDEIRNLTIKQGVFLKDIASVDLSYKDANEAAIMDKKSGVLLGLELAPDANALTVIALAKSKLDQFKSLLGSEYDVKIAYDKSEVIQKHIDQTAFDMILGILLTIVIVYLFLRNFSITIISVVAIPTSIVATFFIINALGYDINRLSLIALTLGIGIFIDDAIVVTENIASKLKDEPNALKASFAGIKEIAFSVFAISLVLLCVFVPIAFMSGIVGKYFNSFAMSVAAGIVISFFVSIFLVPTLSARFVNAKQSGFFLKSEPFFEALENFYEKILALALKFKLIFLAITLVVVVCSFTLAKFVGGDFMPSEDNSEFNIYFKLDPSLSLQASKDKLKDKISLINADPQVAYAYFILGYTDAKQPYLVKAYVRLKELKDRVNHERQNAIMQSFRDRLKSDDMSVIVADLPVVEGGDVQPVKLTITSENGKELEKFVPKISKMLKEINDATDVNSPEEDLLKRVQISIDEDKAKRLILDKASVASAVYSAFSQNEVSVFENENGKEYELYMRLDDKFRSDTDDILKTKIRSKEGFFVTLGDVATISFEQKPASISRFNRADEIKFLANTKNNAPLNSVANEISKKLDEILPANFKYKFLGFVELMDDTNASFIFTVSASAVLIYMVLAALYESFLLPFLIMLAMPLAFCGVVIGLFISGNPFSLFVMVGVILLFGMVGKNAILVVDFANHFANNGIEANEAVKMAAKKRLRAVLMTTFAMIFAMLPLALGRGAGFEANSPMAISIIFGLISSTLLSLLVVPVLFAWVYNLDKFIRKFYERERI

>208964.12.peg.1484_RND

MQALRSGGGRVLVGVLAAGLVAFGGWAWLGGDAGAKAAPAPARVPVIVARVERRDVEQQVSGIGTVTSLHNVVIRTQIDGQLTRLLVSEGQMVEAGELLATIDDRAVVAALEQAQASRASNQAQLKSAEQDLQRYRSLYAERAVSRQLLDQQQATVDQLRATLKANDATINAERVRLSYTRITSPVSGKVGIRNVDVGNLVRVGDSLGLFSVTQIAPISVVFSLQQEQLLQLQALLGGEAAVRAYSRDGGSALGEGRLLTIDNQIDSSTGTIRVRASFDNRQARLWPGQFVAVSLHTGVRRDQLVLSSKAVRRGLEGNFVYRVADDRVEAVPVRVLQDIDGLSVVEGLASGDQVVVDGHSRLMPGALVDIQEPRPSLAQATERRP

>244582.5.peg.1519_RND

MNISIPFIRRPIGTTLLAFGLALAGILAFNLMPVSPLPQIEFPTISIQATLPGAAPETMATSVATPLERQLGRIAGITEITSSSRLGTAQITLQFDLSRNINGAARDVQAAINAARSNLPADLPSNPTYKIVNPSDAPIIILALTSDTYSSGQMYDIASTILQQKLSQVNGVGQVIVGGSSLPAVRLELNPTALNKYGISLEQVRTAVAAANNNRPKGQLSDEMHSYIIMTNDQLFKAADYQPLIISYQNSAPIRLSDLGEVIDSVEDLRNAGLSNGKPSVLLIIFKQPGANIIGTVDNVKSALRNLKADIPAAIDLSVVMDRTTTIRASLKDVEFTLILAVCLVIWVIYLFLGNFRAALIPSVVVPLSLLGTFCVMYLCGFSLDNLSLMAMTIATGFVVDDAVVVLENISRHIEAGLKPIQAAILGAKEVGFTVLSMSASLIAVFIPILLMGGIVGRLFREFALTLSIAILMSMVVSLTVTPMMSAYILKPEKKGHHQGRVMNFMMRHYRQSLGWALRRPKFMLTLTAATIASDIFLFVIIPKGFFPQQDVGRIVASIQAQQDISFQALKQKLNDYVKIVKDDPAVETVVGFIGGNSASGNAGTMYISLKPLEERKLPIDDIMGRLRGKLAAIPGASVYMRATQDLVIGGRQSNALYQYTLTSYDLNELNTWAPRVLEKLATLPGIVDVNSDQLSNGKEVFVTIDRDAASRLGVSPQTIDNTLYDAFGQRQIAIMYTALNQYHVVMELAPQYWQRPETLDLIYAPSATNNQIPLSVVTKSKISNTLLLVNHQGQFPAATISFNLLPGYSLGQAVEMINEATTEIGMPKATMHGSFQGTAQAFQDSLSSQPLLILAALIAVYIVLGILYESTIHPITILSTLPSAGIGAMIALLLTGTELSIIAIIGMILLIGIVKKNAIMMIDFALEKERQQHKSAIASIYEACLLRFRPIMMTTMAAILSAVPLAFGSGVGSELRKPLGISIIGGLIFSQMLTLYTTPVIYLSMERVSSWWKRRHKQTSVVVLPLLLLLLNACEVGPDYVRPVIETPAQFKEPPAGWKFATPQDTVDRGTWWDMFNDPLLSNLVAEVELTNQNLALAEAQHRQSQALVDQARAGFFPTINATTSATRQKSFSTGSTNLASAPTNLYNVGLNATWELDVWGSVRRSVESSEAGAEAAAANVALTKLSSEASLTQFYYELRAVDATQKLLDETVGSYQKLLVLTQNRHRMGVSTGLDIAQAESQLKTAEVKAIDNKVTRAQYEHAIAVLVGKAASDFSIPVDSSALPEPPTLPSALPATLMERRPDIAQAERQMAQANATIGVNIAAYFPNLTLNGSGGYESTLWHKLFTAPSQIWSMAGQMAQLVFDGGLVSGKVEAARAAYDQSVANYRQVVLTAFQETEDNLAALRILESEIKSQVEAVKAAKKQLNLTINEYKSGTIYFSDVMTAEINYFTARSNYIAIAARRLTATASLVKSLGGGWCSSDLIREGNWEHKPSPTQQENNR

>318161.16.peg.3236_RND

MFSQFFIKRPIFAAVISLMFFIAGAIAVWKLPITEYPEVVPPTVVVTASYPGANPKVIAQTVASPLEQEINGVEDMLYMSSQATSDGLMTLTITFAIGTDVDRAQTQVQARVDRASPRLPQEVQRLGIVTEKSSPDLTMVVHLTSPDKRYDMLYLSNYAALNVKDELARIEGVGAVRLFGAGEYSLRIWLEPNKMAGLNLSPAQVLAAVREQNQQAAAGSLGAQPSGGADFQLLINVKGRLSTVEEFEDIIINVGPQGELSRLRDVARVELGASTYALRSLLDNQDAIAIPVFQASGSNAIQISDDVRAKMSELSASFPDGLSYDIVYDPTVFVRGSIEAVVKTLFEAILLVVLVVVLFLQTWRASIIPLVAVPVSLVGTFAFMHLLGFSLNALSLFGLVLAIGIVVDDAIVVVENVERNIGDGLSPIAATQKAMREVTGPIIATTLVLAAVFIPTAFMAGLTGQFYKQFALTITISTFISALNSLTLSPALAALLLKGHDAPKDRLTRAMDKLFGTWLFNPFNRMFEKASRGYGFIVKKVIRFGAIVGIIYLALVALTGVMFASTPTGYVPGQDKQYLVAFAQLPDAASLDRTEAVIKQMSEIALAQPGVAHSVAFPGLSINGFTNSPNSGIVFTPLDDFSERTDPSLSAEAIAMQLNQKFAGIEDAYIAIFPPPPVQGLGTIGGFRLQIQDKGNLGYDELYKVTMQVMQKAWGTPELTGVFSSYQVNVPQLDLNIDRTKAKQQGVSLDEVFQTLQTYMGSTYVNDFNQFGRTYQVKMQADEQFRQTPEQISQIKVRNQQGDMVPLGSFINVTQVAGPDRVMHYNAYTTAELNGGPAPGYSSGEAQAAIEKILAETLPNGMTYEWTEITYQQILAGNAGLLVFPLVILLVFMVLAAQYESLSLPMAIILIIPMTLLSALSGVLLYGGDNNIFTQIGLIVLVGLATKNAILIVEFAKELQDEGMNVMDAILEATRLRLRPILMTSIAFIMGVVPMVFSTGAGAEMRQAMGVAVFAGMIGVTIFGLLLTPLFYHFMAKRQKTNVDKNVEPDDSQGQLFAPVVNPAVNTLVTHKGANADA

>343509.12.peg.4097_RND

MTGLDNLLYMASQSTNTGRATTTLTFLAGTDPNEAMQQVQSTAGRTAPSTPGGAKPGHDRKQNRRHQFDDGGFRVYRRVHG

>360107.7.peg.546_RND

MFSKFFIHRPVFACVISIIITLAGLVSLRGLPIEEYPNLTPPQINVFASYPGADAQTIAETVAAPLEDALNGVEDMIYMQSTSSSAGTMRLSIYFKTGTSPQIAQVNVNNRVNLASKLLPDNVTRQGISVFERSDSILEVISFYDPSGQMDIIDLSNYLTINVVDEIKRVNGVGEAFIVGDKKYSMRVWIKPDLLNKYDITTSDVINAISEQNTQYSVGKIGELPENSNSAYVFSIRTEGRLVKVSDFENIIIKSLPNGSALKLKDVANVELGSENYMSNNLINGHYMMPMLVFMQTDGNAIATADAVNKRIEELSKNFPGNLTYNVNYNTTDFVKVSMKEIFQTFIEALVLVLIIMYLFLGNLRSTIIPMIAIPVSIIGTFAGIYAVGFSVNLITLFAMILAIGIVVDDAIIVVENVERNLEENPNISVIEATEKAMEEIMAPIISIVLVLCAVFLPASFIEGFVGIIQRQFALTLVISVCISGIVALTLTPALCAKFLRRDMAKKPKISQWFNKIFDISTNIYAAGVAKILKHIIPSLIVVAILCFCTWRLFTMVPASLVPEEDKGVSIAVSQLPPASTITRTENVIKKQSDELLKNPLIDAVGAMMGYDLFAGGLRENATVIFLKFKDWSERKEKDQSSFAINKKYNILFSQDRNSTTFVLNPPPINGLSLTGGFELFAQNTTGKSFAEIEKDMKVVAAKANARGDLVRVRTTLDTNFPQYKLIVNTQKAKMLNVNIKNLYMTINTMLGQYYVNDFNFLGKTFKVNVKAAGEYRNSVDDLRAIFVKSNDGKSIPVNSLIKLENALGPDTVNRFNGFPAAKIMGDPAEGYTSGQAIDAIAQVFKEEFPNEYTLGWSGTSYQEVQSSGKGATAFIFGLIFVYLILAAQYERWLMPAAVMTAVPFSVFGAILFTYLRGLTNDIYFQIGLILLIGLGAKNAILIVEFAMTEHKKGKNIIEASIAAARLRFRPIVMTSLAFAFGVLPMVISSGAGSASRHSLGTGVIGGMIAASTIAIFFVPLFFYLLETFNNWQAKLSRTKEIKRIRKIRREENA

>401053.4.peg.383_RND

MSHEFQPGDKAPRDGATASDIYRLEHEDTRGGDHAPRDHASPRDEKKQKEDEGPNGGGVHFSAPFIRRPVATFLLSAAIILAGAVAYKLLPVSSLPQVEFPVISVGANLPGADPETMASAVATPLERQFSRIAGINQMTSSSSIGSASITLQFDLTRDINGAARDVQAAINAARSQLPANLPSNPTYRKINPSDAPIMILALTSETLSVPQLYDAADSVLAQKLASVDGVGQTFVGGSSKPAVRIEANPTQLTSYGLGLDALRAAIATINVNQPKGYLNGAGTEGQRWSITTTDQLFGAAAYKPLIVATDRGPVSSAAASNGLQSNVASATTSTTTTNSVSSSGTTGTSSGTATSSASTTSSTASTYSTTATPITTTTATSAAGMASAQVTPSVSNVATPTIGGHGIVRISDVSDVVDSVEDIHNGGLFNLHPAILVIVFKSPGANVIQTVDAINKMLPSLSASISPAIKVQVALDRTATIRASVDDITRTMLITIVLVVLVVFFFLREVRSTLIPAVSVPLSLLGTFGVMYLLGYTLDNLSLMALTISTGFVVDDAIVVIENISRHLEEGLTPYDAAMKGSAEIGFTVVSMSISLIAVFIPILLMGGIVGRLFREFAVTLSVSILVSLCVSLTTTPMLSAKFLQPHSANKHGRIYLLGERFFDWMVGEYTLGLRWVLRHQGLVMLITIGTFLLNIYLFILVPKGFFPQQDTGRLGGRILGQQDVSFDAMKAKAIEMTDLVKQDPGVLNVMTNLGGGGPGGGSSNSANMFIFLKDPAARAKDGDTAEVIINRLRPKLSRMPGVQVYLQSQQELNIGGRQSATQYQYTLQADSVQDLNLWSPKMMAAMQKMPELRDVATDQLENGLESTLVIDRDTASRLGITPLAIDNILSDAFGQRQVSTTYKPLNQYHVVMEVAPQFQKDPDAIRQIYVKNSSGKSIPLTAITHFEMQRIPLQVNHQGLTPAATLSFNLAPGIALSQAAEAIDRARNSISMPASVTGGFQGSAQAFQQSLSSEPVLILLALTTVYIVLGMLYESFIHPLTILSTLPSAGVGAILALLITHTDLSVIAMIGIILLIGLVKKNAILMIDFALVAEREHGKEPVDAIYEACLLRFRPIMMTTMAALFGGLPLAFGTGVGSELRRPLGITIVGGLIVSQCLTLFTTPVVYIYFDKWRQRMESWRGKPVEKKLPRGLRSHPEPVAGD

>436717.3.peg.1877_RND

MQKHLLLPLFLSIGLILQGCGSQETAQAEPAPAKVSVLSIQSQSVNFSENLPARVQAFRTAEIRPQVGGIIERVLFKQGSEVRAGQALYKINSETFEADVNSNRASLNKAEAEVARLKVQLDRYEQLLPSNAISKQEVSNAQAQYRQALADVAQMKALLTRQNLNLQYATVRAPISGRIGQSFVTEGALVGQGDANTMATIQQIDKVYVDVKQSISEYERLQAALKTGELSANSEKTVRISNSHGQEYNVTAKMLFEDINVDPETGDVTFRIEVNNTERKLLPGMYVRVNIDRASIPQALLVPAQAIQRNINGEPQVYVINAKGSAEIRPIEIGQQYEQYYIANKGLKVGDKVVVEGMERIQPNQKLAMTTWKKPASENSASNVETKPSTNQGAQP

>547045.3.peg.622_RND

MASYASKVMRMAAIAAATALALSACNKGSDATQGAKDGKGQQAAAQKEAPPPVVGVVTVHPETVALTTELPGRLESLRTADVRAQVGGIIQKRLFQEGSYVRAGQPLYQIDSSTYQADLESSRAQLAGAQATLAKANADLARYKPLVAADAISKQDYDAAVTAKRSAEASVKAAQAAIKSAGINLNRARITAPISGFIGQSKVSEGTLLNAGDTTVLATIRQTNPMYVNITQSATEVMKLRQQVAEGKLSSVDGAIEVGIKFDNGEVYPHKGRLLFSDPSVNETTGQITLRASVPNDKNILMSGLYVRVLMEQVAADNAFVVPQQAVTRGTKDTVMIVNAKGEMEPREVTVAQQQGTNWVITAGLKDGDKVIVDGIAIASMSGGKKVTPKEWTPPEKAAASAAGAAPKAASEAKKDVQTTSEAKPASAAK

>575.7.peg.976_RND

MKKGEEHGKKGFFGWFNRMFNRNASRYETAVGKILHRSLRWIAIYALLLGGMVFMFLRLPTSFLPQEDRGMFLTSVQLPSGATQQQTLKVVQKVEDYFFNHEQANVASIFATVGSGPGGNGQNVARMFIRLKDWDERDAKTGTSFAIIERATKAFNSINEARVFATNPPAISGLGSSAGLIWSLKTTPATGTRRSWQPAIRCSIWRRKMSG

>648757.4.peg.592_RND

MSGPTAEEPGGVGAQGADAAGEDTYSGISAPFIARPIATSLLAVAILLASLLAYSLLPISSLPQVDFPVVQVTTRLPGANADTMARLVTAPLERQLGQIPSLENMSSTSSEGLSQITLRFMLSRDINAAGQDVQSAISAAGGSLPQNLPYPPVYAKVNPSDPPIVTIALTSQSVSLERLSDFADTLLAPRLSQVAGVGRVTVQGNIRPAIRIQANPLQLASLGIALETVRSAIANANVTGSKGLISGPEKSYIVGANDQLETAGAYEDVVVAYRNKAPVLLRDVATVVAGLENERVAARYNGTPAVVIDVQRQPSANIVGTVDELKKILPKLVDALPAGVKLDIVADRTGTIRASVEEVQFTLVLSVALVIMVVLLFLRTLSATIVAGITLPLSLMAAFGVMYYAGFSLNNLSLMALTIATGFVVDDAIVMIENVMRYIEKGEKPLVAAYKGAGEIGFTIVSLTLSLIAVFIPLLFMEGIVGRLFREFALTLTAAVVTSMIVALTLTPMMAARLLRAPRHGETAPWYSRAFEAPFNALLSVYRVTLDWALNARRFMLLVAAATFVLTVVLYIAIPKGFLPDQDTGFLTAETEAAPGVSFERINALQAEVERIIRRDPDVLGVVSVIGVGTTNATPNAAHLALTLKPKTERKATATEILQRLTEATADFPGLRTTFQIVQDIQIGTARSRTQYQYVIVGLDREGFSGWAQKLEAELSRDRRLIHVASDLQEDGNAVLIKTDRVIAGRLGVTMQALNDTLYDAFGQRQISTIYGQSNQYRVVLEVAPAFQTDTAALGSIYVPGTAISNSTSGNASTGNATASGATNSSITATSASGTGVGSQVPLSSFSVIERATAPLSVNHVQQYPAATISFDVAPGFSLDAAVQAVTDAQSRIALPSSIVGSYTGAAAEFNASLANQPLLILAAVVTIYIILGVLYESFIHPFTILTTLPSAGIGALLALEILGMEFSFIALIGIILLMGIVKKNAIIMIDFALDAERTRGLAPFDAIREACLLRFRPIMMTTVAALLGALPLVIGSGPGSELRMPLGVTIIGGLLLSQLLTLYTTPVIYLAMDGLKRRIERRFGIDEPNYPPPALRPEPGLPDPGPRGGSPRGTGGGGAAGLLPIFGTPSLPMLPPRADWLLLPSPEGNAALPSPNEPLALPPPDKPAS

>679897.3.peg.488_RND

MYKFAIQRPITTLMFAIAVMFFGILGIKKIPVALFPNIDFPIIVISTTYPGGSPEIIESKVTDKVEEAVMGIDGVKKITSNSARNVSIVVVEFYLEKPVEQAMTDVIGKISSIKFDDSNIQQPSIRKFDTSGQAIISLFMSSKQKGPTEIMRHADLIVKPILQSILGVGGVQLNGYRERQIRIYADSTLMNKYGITYDNLFGMLGKENLEANGGRIESATKDFSITVDANSTSIKDIANIRIGKDNVRLSDVAVVEDGLQEETTYAAFNNEPGVIFEVMKVSGANELEVADGVYKALPKIQVASHGYEIVPFLDTTQYIRHSIKDVQFDLMLGGVLAVLIVFLFLRSVTITLVAAISLPISILGTFALIEMLGHTLNMMTMMALTLAIGIIIDDAIVVIENIHKKLELGMSKKQAAYEGVNEIAFAIIAISAMLLSVFVPIANMSGIIGKFFASFGVTVALAIVISYVVVITVIPMVSSLIVSSKQSRFYHFTEPFFNGMENFYLKILRLGLSHKLLFSALTFLIFGFSIYVAKGLGMEFMLKEDKSQFYVWLETSPGISIHEMKVRTLALQEAIAKHEEIEYTTLQVGYGSIQSIFKAKIYAKMKPIEERKISQFDMMKSITDELKKMPQAKGLNVFSSEVPVLGGGDSTPFQVTIYGMTQQAVDKSVAKLKKMLDEDPRFQGKITNYHTSTSDIQPEYKITVLRQNADKYGVRTQEIANVVSAAFSGVNQAAYFKQGGKEYKITMRVPDDERVSVDDIRKLQVMNSSGKLMFLDGLVEITRSQSPSLINRYGRQRSVTVYAAPLKNSGLSLGSMISIVQTNSKDWLEEGVNFAFSGESNNAAESAASFMTAIITAFILIYLILAALYESLLEPFIIMITMPLSFAGVFFSLKLAHQPFSMFSFMGLILLIGIVGKNATLLIDVANEYRKKFKAGVHEAIIFAGKSRLRPILMTTIAMVFGMLPLAVATGSGYAMKSPIGISMIGGLLISMFLSLLMVPILYVIVAPIDDKLKRFYQSEDGEGILQSVVKKIKPGKKEKEKQEEDKDSKKKKKKKDKKKD

>754502.3.peg.2425_RND

MNISRLFILRPVATLLLMIALVLVGLIAMRVLPVSSLPNVDYPTIQVQTFYPGASPTVMATTVTAPLEVQLGEIPGLQQMTSYSSDGASVITLQFDLSLNLDIAEQNVQQAINAANSYLPSGLPAPPTYAKVNPADQPILTLAVTSKSMSLTQLEDVANNRLGTKISEVSGVGVVTTSGGNVPAIRVEADPHKLAAYGLNIDDLRTLLSYVNVSQPKGNFDGPDLDYTINGNDQITDPKDYLDTVIAYQNGSPVFMRDVARVSQAAQDVERGAWYNGSPAIVLNVQRQPGANVIKTVNQIMKELPQLESTLPAGMKVTVVSDSTGVIRASVADAAFELILAIVLVVAVIFVFLRNVPATLIPSISVPVSLIGTLAVMYQLNYSIDNLSLMALIIATGFVVDDSIVMIENIVRYLEEGMSPLEAALEGAGQIGFTILSLTVSLIAVLIPLLFMGGVIGRLFSEFAVTLAVTIVISAVVSLTVVPMLCARMLRAQAERHPSRFERISEGLFDKTLAAYERGLRWVLDHQTLTLMVAIATVVLTGILYVVIPKGLFPVQDVGVIEGISVADNSVSYAAMVQRQSALADAVLKDPDVVSLTSYVGIDGTNATLNNGRFLINLRERDKRSDNAQEIARRLAQEVAHVPGVKLFMQPEQDLTLDTTVSPNQYSFALRGPSQQAFQKYVPELVARLKRIPSLSDVQSDLNSDGLSVNVEVNRQLAARFGITPATIDNALYDALGQRIVSTIFEQSAQYRVILVAKPETMPTLQSIGDLYLPSQTSSTGQVPLSGIAKIEIRKAPLVISHLAQFPAVTVSFNLAKGASLSTAVKEIHQAEQAIDLPPSITSSLQGATAAFEDSLSSEVYLLIAALVAVYIVLGVLYESFIHPVTILSTLPSAGIGALLSLMLAGMDLDVIGIIGIVLLIGIVKKNAIMMVDFALDAERNHGKAPRDAIFEASLLRFRPILMTTLAAMLGALPMLLGTGTGSELRRPLGLAIIGGLTLSQMLTLFTTPVIYLFFDRMAARVNRWRAARAERNGGDEPGGRPPEGGAGGTRVNIPAIFIRRPVATTLLAIAILISGTLAYFRMPVAPLPNIAFPVIVVQANMAGASPSVMASTVAEPLERRLATIADVEELTSISYVGSSMIIVEFGLKRDINGAARDVEAAIQAARADLPTTLRSNPSYRQYNPADAPIMVLSLTSDTLTKAQLYDSADSVIQQQLSQVRGVGQITLGGGALPSVRVELQPGKLNSYGIGMEDVRAAISAANADSAKGHLDVGDQRYVVTSNDQITHAAPYRDLVVAYRDGAPVQLRDVAQVRDSNENIRNAGLFNGKSAILVIVYPMPGSNVVSTVRQIRNVLPSIQATLPSSVHVDVAIDRSQSVTSSVSDTERTLFIAVLLVVGVVFIFLQSPRATLVPAVALPLSIVGTFGPMYLLGYSIDNLSLMALTIGTGFVVDDAVVVLENVVRYIEQGLSPKEAALKGAGEVGFTVISMSLSLIAVFLPIILFPGIVGLMFHEFAITLSIAILISLVISLTVTPAMCAYVLSRDHAGHSRARWAQWIERQFDRFKGVYARSLTAVLDHSLLVILLLFALLVGNVFLLKLVPATFFPEQDTGILIGQIIADQSISFSAMQKKLAQLQSIVQRDPAVQSVAGFTGGRALNTANVFIELKPLSQRHATAAQIVNRLRPKLNQVSGARLFLQAQQDLRIGGRQSAAEYQYTLTSDDSAALFTWTPKLVAALSKERGRLLDVNSDLQQNGLQTYVSINRATAARYGFAPNQVDNVLYDAFGQRTVSTIYNPLNQYFVVMEVAPEYWQYPQTLNQIYLSKSAGNPSGTAATQMPHGTVSALSSTNASTSSTSSTTNSRNSDAQSNATNNSIANSKGGSSTGSADSTAAETMVPLAVMASYASSHTSTQVNHQSGLVAATISFNLPAGGSLSQAGAAINDTIREIGMPASIHGSFAGAAAAYSQSMGVVPLLILAALAVVYIVLGVLYESSIHPLTILSTLPSAGIGATLALLIFGTPFSVIAMIGIILLIGIVKKNGIMMVDVAIQLQRQQQMTARDAIHEAALIRLRPIMMTTFAAVLGAVPLAIGIGQGGSLRQPLGITVMGGLILSQMFTLYTTPVIYLYLDRLRARLVRWSAGLRWNRDAKPGQPDTMA

>887898.3.peg.390_RND

MNLSRPFIRRPIGSTMLALAILLAGWLAWRQLPVAPLPQIDTPMVVVSASLPGASPTSMAATVAGPLERALGAIAGLSSISSSSSTGTTEVRLFFDIDRDLNEASREVQAAINGVIDQLPPGMPGRPTFRKLNSSTSPILALALSSATLPPSQLYDLADNIVLQKISRVQGVGEVSLGGASLPAVRIRFEPSALAALGMSLEDARQVVVAASAEAPEGFLEDEGNRWLVATGHKLKNAADFSDLVLRWKNGQAVRLSDVAEVSDSVENRYSSGFHNHQPAIIALVTRQPDANVVATIDAIKATLPQLQAILPPQASLTVVMDRSLGIRGSLAEAQWTLVFSCLIVAAVVWLFVARLRTALIPVAVIPVSLIGTFAVIWLAGFSLNNLSIMALVVAAGLVVDDAIVVLENITRHTERGLSPYRAAMRGAGEVSFTLLALNVALVVVFVAVLFMGGIIERLFREFSLTLAAAIVISLVVSISLTPALCAHGLPRERRQKAAEHGAAQAAALPGGMPDVTHDAGQGMPGSVSLQDDAEVARAPWHRRLLGLHASYFHHLQAAYEQSLAWMLRYAWYGVVALVGLIAASVWLFANLPRSDLPEQDTGVIGAFIRGDDGFSFQIMQPRIERYRRWILSDPAVQDVAGISGGNGGLTNARLVITLKPLAERKVSARQVIDRLRRNAPQMAGTMFFGRVEQDLQLSPPKFGDDADHVIVLKSGDRDLLRTWNQRLGVALSKRPELENVRYSLGEDTRQIVLDIDRNTASRLGVQLTDISAALSNSFAQRQVATLYQDRNQYRVVMEVSERFTENPLALDRVQIITSEGKSVALAEVARWHFGMVQDRERHVDQFSASTISFSVAADVTDTAALEAVRKVIDAERMPVTVIADIDGDDGRPKSLVKADGQGWLILGVVLAVYLVLGILYENLLHPITVLSTIPSAGVGALLALWASNTPFSLIALLGLFLLIGVVMKNGILMIDVALKKQLHEGLAPQVAILQAAGQRLRPILMTNVAALAGAIPLAMGLGDGGELRRPMGLVIIGGLAVSQLITLYTTPALYLLLERLQQRLRRGRG

>P9WJV1_RND

MIVQRTAAPTGSVPPDRHAARPFIPRMIRTFAVPIILGWLVTIAVLNVTVPQLETVGQIQAVSMSPDAAPSMISMKHIGKVFEEGDSDSAAMIVLEGQRPLGDAAHAFYDQMIGRLQADTTHVQSLQDFWGDPLTATGAQSSDGKAAYVQVKLAGNQGESLANESVEAVKTIVERLAPPPGVKVYVTGSAALVADQQQAGDRSLQVIEAVTFTVIIVMLLLVYRSIITSAIMLTMVVLGLLATRGGVAFLGFHRIIGLSTFATNLLVVLAIAAATDYAIFLIGRYQEARGLGQDRESAYYTMFGGTAHVVLGSGLTIAGATFCLSFTRLPYFQTLGVPLAIGMVIVVAAALTLGPAIIAVTSRFGKLLEPKRMARVRGWRKVGAAIVRWPGPILVGAVALALVGLLTLPGYRTNYNDRNYLPADLPANEGYAAAERHFSQARMNPEVLMVESDHDMRNSADFLVINKIAKAIFAVEGISRVQAITRPDGKPIEHTSIPFLISMQGTSQKLTEKYNQDLTARMLEQVNDIQSNIDQMERMHSLTQQMADVTHEMVIQMTGMVVDVEELRNHIADFDDFFRPIRSYFYWEKHCYDIPVCWSLRSVFDTLDGIDVMTEDINNLLPLMQRLDTLMPQLTAMMPEMIQTMKSMKAQMLSMHSTQEGLQDQMAAMQEDSAAMGEAFDASRNDDSFYLPPEVFDNPDFQRGLEQFLSPDGHAVRFIISHEGDPMSQAGIARIAKIKTAAKEAIKGTPLEGSAIYLGGTAAMFKDLSDGNTYDLMIAGISALCLIFIIMLITTRSVVAAAVIVGTVVLSLGASFGLSVLIWQHILGIELHWLVLAMAVIILLAVGADYNLLLVARLKEEIHAGINTGIIRAMGGSGSVVTAAGLVFAFTMMSFAVSELTVMAQVGTTIGMGLLFDTLIVRSFMTPSIAALLGKWFWWPQVVRQRPIPQPWPSPASARTFALV

>P32714_RND

MINRQLSRLLLCSILGSTTLISGCALVRKDSAPHQQLKPEQIKLADDIHLASSGWPQAQWWKQLNDPQLDALIQRTLSGSHTLAEAKLREEKAQSQADLLDAGSQLQVAALGMLNRQRVSANGFLSPYSMDAPALGMDGPYYTEATVGLFAGLDLDLWGVHRSAVAAAIGAHNAALAETAAVELSLATGVAQLYYSMQASYQMLDLLEQTHDVIDYAVKAHQSKVAHGLEAQVPFHGARAQILAVDKQIVAVKGQITETRESLRALIGAGASDMPEIRPVALPQVQTGIPATLSYELLARRPDLQAMRWYVQASLDQVDSARALFYPSFDIKAFFGLDSIHLHTLFKKTSRQFNFIPGLKLPLFDGGRLNANLEGTRAASNMMIERYNQSVLNAVRDVAVNGTRLQTLNDEREMQAERVEATRFTQRAAEAAYQRGLTSRLQATEARLPVLAEEMSLLMLDSRRVIQSIQLMKSLGGGYQAGPVVEKK

>Q2EHL7_RND

MFTIKKLTLTIVVATTLTGCANIGDSYRASLKNYKQYEEITKQYNIKNDWWKLYKDAQLNRVVEKALLNNKDLAKATISVNRALYSANLAGANLVPAFSGSTRSTAQKNIKTGGNSTISHTGSLNVSYTLDLWFRLADTADAAEWAHKATVQDMESTKLSLINSVVTTYYQIAYLNDAISTTKESIKYYTDISNIMRNRLAQGVADSISVDQAQQAVLTARNNLITYQLNRKTAEQTLRNLLNLKPDETLKITFPHILKVKSVGVNLNVPVSVIANRPDIKGYQARLSSAFKNVKATEKSWFPEITLGGSLNSSGKKLNSATNTLIGGGALGISLPFLNWNTVKWNVKISEADYETARLNYEKSITVALNDVDTNYFSFTQAKKRFTNAQKTYIYNQRITQYYRNRYNAGVSELREWLTAANTEKNSQLSILQAKYNVIQAENAVYSSMAGYYSVKK

>Q83KF5_RND

MNRDSFYPAIACFPLLLMLAGCAPMHETRQALSQQTPAAQVDTALPTALKNGWPDSQWWLEYHDNQLTSLINNALQNAPDMQVAEQRIQLAEAQAKAVATQDGPQIDFSADMERQKMSAEGLMGPFALNDPAAGTTGPWYTNGTFGLTAGWHLDIWGKNRAEVTARLGTVKARAAEREQTRQLLAGSVARLYWEWQTQAALNTVLQQIEKEQNTIIATDRQLYQNGITSSVDGVETDINASKTRQQLNDVAGKMKIIEARLNALTNHQTKSLKLKPVALPKVASQLPDELGYSLLARRADLQAAHWYVESSLSTIDAAKAAFYPDINLMAFLQQDALHLSDLFRHSAQQMGVTAGLTLPIFDSGRLNANLDIAKAESNLSIASYNKAVVEAVNDVARAASQVQTLAEKNQHQAQIERDALRVVGLAQARFNAGIIAGSRVSEARIPALRERANGLLLQGQWLDASIRLTGALGGGYKR

>Q8FWV8_RND

MVAFWTCRNAWFQHLPFAKRGDENAPSGPRRLRPWFLVLALGLAACSEDKSAPQQAAPLPPIPVGVIKITERPTHPQLSFVGRVEATDSVDLIARVDGFLDKRTFTEGQAVKTGDLLFVLQKDALQAALDAAQANLAKAQADADNLKLQTERARSLYKQKTVSQAMLDDRVAAEKQALAVVQQAQASLEQAQINLGYTDIRAPFSGRIGMANFSVGALVGPSSGPLATIVSQDPIYVTFPVSDKTILDLTEGGRTATDRSNVAVSLTLSNGMTYPQTGAIDFTGIKINPNTDTLMVRAQFPNPNNVLIDGQYVQVTAASKHPVEALLVPQKAIMTDQSGNYVLAVGEDNKVIQRQITQGSTFGSNVVVKSGLAVGDQVVVDGLQRIRPGQKVDPQIVDATTPAQKAMSVGN

>Q8FWV9_RND

MLSSVFINRPRLAIVIAIVITLAGLIAVTRIPVAQFPDIVPPQVSVTATYPGASAETVEAAIAQPIEAQVNGVDDMIYMSSTSGNNGTYTLTVTFKVGSDPNLNTVNVQNRVRLAEANLPQEVTRLGVTVKKQSSSFLQIITLLSPDSRYDELFLNNYGVINVVDRLARVPGVGQAQSFGTFNYSMRIWFNTDALTSLNLTPNDIVNAISSQNVQAAVGRLGAPPMTDQQQIQLTLTTQGRLTDAKQFENIIIRANPDGSSVRLKDVARVELAAQSYDTIGRLNGKPASVIAVYQAPGSNAVAAAEGVRNVMEQLKQSFPAGLDYKITYDTTVFVSSTIHEVIKTLLEAFVLVVVVVFIFLGNFRATLIPTLAVPVSLIGTFAVLLVLGFSANTISLFAMILAIGIVVDDAIVVVENVERVMAETGLPPKEAAKQAMQEITAPIIAITLVLLSVFVPVAFIPGITGALYAQFALTVSVAMLISAINALTLSPALCGVFLKPHQGRKKSLYGRTMDKLSSGIEKISDGYAHIVRRLVRMAFLSIVLVAGLGAGAYFLNTIVPTGFLPEEDQGLFFVQVNLPPAASQSRTAAVVSEIEADITKMAGVADVTSVTGFSFIDGLAVSNAGLMIVTLKPLEERLKDNITVFDVIAEVNRRTAAIPSAVAITMNLPPILGLGSSGGFQYQLEDQEGQSPQQLASVAQGLVMAANQNPKLSRVFTTFATDTPQLNLNIDRQKALSLGVSPNNIIQALQSTLGGYFVNNFNTLGRTWQVIIQGEQQDRKTVEDIYRINVRSSHGDMVPLRSLVSVEERLGPLYITRYNNYRSASIQGNAAPGVSSGEALAAMAQVSKTTLPSGYGYEWTGTALQELQAAGQTSMILALAVLFAYLFLVALYESWTIPVGVLLSVTAGLAGAMLALWITGLSNDIYAQIGIVVLIALASKNGILIVEFAKERREEGVPLEQAAIIGARQRFRPVMMTSFAFILGLVPLVIAVGAAAASRRAVGTSVFGGMIAASAVGIFLIPMLYVVLERVREWGHARILRKPLYEEEKQEKADGDASGPTVPPTQPEDRGLS

>Q8G2M7_RND

MTLNRTIRCFAAGAAFIVFAAQPALAQAPGGATPPPPQVFVVDIKPHDVPVTYEYAARINAYRNVQVRARVGGILLHRNFVEGTQVKAGEVLFEIDPAPYQAELEKAQAQVAQAEAQYQQSIRDAERAEQLVQQKVQSAAVRDSAFATRDLNKAAVAAAKAQLRTAELNLSYTKVTAPISGITSQEQVNEGSLIGTDASSSLLTSVTQLDPVYVNFSFTDTEAAEIAKLRAERGATGEDADRLKIKILFGDGKAYDHEGTIDFTSSSLDTETGTLGVRAVVENPNHRLIPGQFVRAEILDIQVKDAITVPKAALMQSAQGQFVYVVNKDNVVEVRPVTGARELKNDWLISQGLNSGDRVITEGVIKAVPGRPVQPVVQGVDDKAQAEAGKEQAADKK

>A0A0P7CXJ9_RND

MGFNLSAWALRNRQIVLFLMILLAAIGAMSYTKLGQSEDPPFTFKAMVIRTLWPGATAEEVSRQVTERIEKKLMETGEYERIVSFSRPGESQVTFMARDSLHSKDIPELWYQIRKKVADIRHTLPPEIQGPFFNDEFGTTFGNIYALTGEGFDYAVLKDYADRIQIQLQRVKDVGKVELIGLQDEKIWIELSNVKLATLGVPLEAVQQALQEQNAVSTAGFFETPSERLQLRVSGRFDSVEQIRQFPIRIAERTFRIGDVAEVHRGFNDPPAPRMRFMGEDAIGLAVSMKDGGDILVLGKALESEFERLARSLPAGMELRKVSDQPAAVKAGVGEFVQVLVEALVIVLLVSFFSLGLRTGLVVALAIPLVLAMTFAAMHYFGIGLHKISLGALVLALGLLVDDAIIAVEMMAIKMEQGYDRLKAASYAWSSTAFPMLTGTLITAAGFLPIATAASSTGEYTRSIFQVVTIALLTSWVAAVVFVPYLGERLLPDLAKLHASRHGKDGHAPDPYATPFYQRVRRVVEWCVRRRKTVILLTIAAFVGSILLFRFVPQQFFPASGRPELMVDLKLAEGASLANTAERVKQLEALLKQQEGIDNYVAYVGTGSPRFYLPLDQQLPAASFAQFVVLAKSMEDRERLRSWLISTMDQQFPDLRARVTRLENGPPVGYPVQFRVTGEHIEKARALAREVADKVRQNPHVVNVHLDWEEPSKAVFLEIDQDRARALGVSTAHLSSFLQSSLTGTTVSQYREDNELIEILLRGTRQERSELGNLGSLALPTDNGQSVALSQVATLEYGFEEGIIWHRNRLPTVTVRADIYDKEQPATLVKQIEPTLRDIRAKLPDGYLLEVGGTVEDSERGQKSVNAGMPLFVVVVLSLLMIQLRSFSRTVMVFLTAPLGLIGVTLFLLVFRQPFGFVAMLGTIALAGMIMRNSVILVDQIEQDIAAGLDRWQAIIEATVRRFRPIVLTALAAVLAMIPLSRSVFYGPMAVAIMGGLIVATVLTLLFLPALYAAWFRVKKA

>W0HU59_RND

MNISRLFIFRPVATLLLTLAILLLGLLGYRLLPVAPLPQVDFPTIMVSASLSGASPETMAATVATPLERSLGQIAGVTEMTSSSSTGSTRIILQFELDRDINGAARDVQAAINAARSLLPSSMPSLPTYRKANPSDAPIVMLALTSNTRASGELYDLASSTIQQKIAQVQGVGQVSLLGSALPAVRIDLQPQMLNHLGISLDTVRSAIANSTTNLPKGMLQGATTSFVVDGNGQLDKARDYRSLIITYINGTAIRLSDVATVTDSVEDKYNIGFYNQTPSVMIGVTRQAGANMLETIDAINAALPALQAELPGDVELHKVVDRSPTIRASLYDTEETLLIAIFLVIAVVFIFLRNLQAVIIPALALPVSLIGTCAVMYLLDYSLDNLSLMALIICTGFVVDDAIVVLENITRYIEEGLGPVRASIKGAQEVGFTVLAMTLSLVAVFIPILLMGSIVGRLFREFAVTLTVSLLISMVVSLSLTPMLCSRLLRRKPPVSKRPNRLYLLIESGLARLLAGYALALGWVMRHQRLTLFSLVLTIMLNLFLYGVVQKGFFPNQDTGLLMGMVRADQNISFQAMKPKVEAIAKLIQQDSAVDGVMSSIGGGAFGSRNSGTFFVRLKDYDKRSDSATVVANRLTNKFRNEAGMQLFLMAAQDLHIGGRSANASYQYSLQADDLNLLRVWTPKVKAALEKLPELTSVDADSENGGQEIMLNIDRDKATRLGVNADMLDAMLNNSFSQRQVATIYKTLNQYHVIMGLNEAYTGDAEVLKKLFVVNDNGESIPLSAFITFSSANAALSVAHQGQSATSTVAFNLADGVSLEQAQAAIKDAMVKIALPSTIQAGFQGTAKAFAALAASMPWLILAALAAVYIVLGVLYESYIHPLTILSTLPSAGLGALLLMLVTGTQLTVIALIGILLLIGIVKKNAIMMIDFALAAERNQGLTPQQAITQACLMRFRPIMMTTLAAFFGALPLALGSGGDADLRSPLGMAIAGGLALSQLLTLFTTPVVYLYLDRLSRNSQRAWHRLRKTGTA

>sp|Q65JB2|EBRB_BACLD Multidrug resistance protein EbrB OS=Bacillus licheniformis (strain ATCC 14580 / DSM 13 / JCM 2505 / NBRC 12200 / NCIMB 9375 / NRRL NRS-1264 / Gibson 46) OX=279010 GN=ebrB PE=3 SV=1

MKGMIFLAAAILSEVFGSTMLKLSEGFSAPLPAAGVIIGFAASFTFLSFSLKTLPLSAAY

ATWAGTGTALTAAIGHFIFQEPFNLKTLIGLTLIIGGVFLLNSKRTEAADQKAQLTIEI

>502347.3.peg.3992_SMR

MPFVFSAIVTKVIVEIPLPPGKISVQLPALRDDLQTRLFIGDGPNSSEPDMSWIILVIAGLLEVVWAVGVMTPTY

>56780.15.peg.233_SMR

MLSPFRALAAVACPPAISEQKTGASRIFPEIGLASSSSSSRSLVFTISFRTQIGRYIMKGWLFLVIAIVGEVIATSALKSSEGFTKLAPSAVVIIGYGIAFYFLSLVLKSIPVGVAYAVWSGLGVVIITAIAWLLHGQKLDAWGFVGMGLIIAAFLLARSPSWKSLRRPTPW

>214092.21.peg.591_SMR

MAVFCYLGLAILPLIIEHDNVSRLSLCWLGRPSYASVFRGRPRLSEVVTMAWIILVIAGLLEVIWAIGLKYSHGFSRLTPSIITLVAMAASVFLLAYAMKSLPAGTAYAVWTGIGAVGTAILGIVLLGESASLARILSLGLILAGIIGLKLAS

1. **Non-antibiotic resistance efflux (Non-ARE) : 389 protein sequences**

>tr|A0A1E7GHI5|A0A1E7GHI5_9DELT Nickel/cobalt efflux system OS=Desulfobacterales bacterium S3730MH5 OX=1869298 GN=BA861_05440 PE=3 SV=1

MQVNLIYYLTATLLGGLHALEPGHGKTVVAAYLIGSKGRKMDAVVLGLVVTLTHTLSVILLAIAAKVASTRITLTEEALHGYLGIVAGLMILAVGIWMLVQRIRGREPFHFHSHDHGHGHSHSHDPLQSHSHPHDHHHEEDHDHHHYHEGHHLEIHGDLHSHSHDHGDHSHGHSHDHDHTYNHAHGHDLLHRHSHDDSRGSNPGDVHHHDHYHEGHEVETDDSVHSHDHSHGHSHDHDHTHNDESSEGHSHPHDHSHEHDGEHNHREGHDLATHSSSHTHVHPHNHDHGNNPHSVDMREGKRVSFWQLFLLGVSGGLVPCPAAIAILLAAVGAGRLGEGLTYILLFSLGLAAVLIAIGIAVVSAGSFASRFLDAKRFARKVAIGGAALVTFIGCLTLVSSVRHLI

>tr|A0A1Q8SUR7|A0A1Q8SUR7_9GAMM Nickel/cobalt efflux system OS=Salinicola socius OX=404433 GN=BTW07_05975 PE=3 SV=1

MPGLNATLRFLIPLALLGAIAAAGYSLGWWQHVAVQIVYWQGKFYHALIEAVTALNRAPSATTWSVLLGVSFGYGVFHAAGPGHGKVVLSTYLASQGGAWRRALGLSVLAALLQGVMAIAIIGVLVFGLGWLTRQAMGSIDQAELASFVIVALVGLWLCVRSLRRLWRARHAVAAHAPTATEPRVTPNFSSVSAWSAAPTPSQGALDGASPAQRHSPGASPGAHCGCGHDHHIDPREVGDWRVALLTVLSIGIRPCSGAVLLLGAAALLDQFGKGVVAVLAMSLGTALTVSSLALLSVLARDWVQRHLKPATGGGQWQAWVGLAGGALILMLGLSLTLAQWQRGPAAAPPMLGAPAAQQAQSPRGPFGSVPIQSATSKPTRGREIP

>tr|A0A1R3TVP2|A0A1R3TVP2_9RHIZ Nickel/cobalt efflux system OS=Agrobacterium sp. DSM 25559 OX=1907666 GN=DSM25559_2797 PE=3 SV=1

MLRVVSAFCAQVSGSANGRPPLPCRASPPQVGRSARSAPLASSSTFETGKRVAAMKSPHLWGRCPAGQRGVSPAHQGLIGSSPTDMKQFTSARFLLILCAVFLATLSTAHAQSPLGIGSAEPSISIGGPLAPLFQWINVHQQSFYRALTGALKAMREDPWALTSLIGLSFAYGVFHAAGPGHGKAVISSYMIANETQLRRGIVISFISAILQGAVAIALVGAAYLVLRGTSITMTKATQAMEIASFAMVALFGAWLLFRKLRSLMVKVEPAPALELASPSPVTVGPQRTGMGSGLRFQGKPVFADHAQSGTGDLCTTCGNAHAPDPSMLRAKDFSLHEAWSAIIAVGLRPCSGAIIVMSFSVLNGLLMGGILSVLAMSIGTAITVSLLACLAVKAKDIAVRFAGTGSTKASRITHGIEIAGAVFVLLMGLGLLGASLQV

>tr|A0A1V4MH18|A0A1V4MH18_9FIRM Nickel/cobalt efflux system OS=Firmicutes bacterium ML8_F2 OX=1775675 GN=AVO34_06030 PE=3 SV=1

MMRLLLLHVTALILVYPVMPSCPQSVAYAAATEMRSSQPPEWRRMYHKTLQRLVHWQKVLRAKLTRLTRDMRDDPWGASFWTFLAIAFLYGIVHAVGPGHGKAMVGSYFLNRSGTLKQGVLLGFLFAFTHVFSAVVLLLAGRAWLQTSARSLLASADHWLQKISAILLLVIGLLLTGRTLWTCLPHRKKHLPHNPKADLKSLCSIAAAAGLVPCPGAALILLFALSQQLLVPGLLAMLALALGMALTVTLSAVATIVTRGALLRVLPTSRSMVVTGRILGVGGGLVITTLGALLLLSAT

>tr|A0A2E4Q009|A0A2E4Q009_9GAMM Nickel/cobalt efflux system OS=Cobetia sp. OX=1873876 GN=CL809_07080 PE=3 SV=1

MSSEPKDTSGLGDIKSLKGMRDGLLKPASSRGRRLAGWLAVGCGLLVLGMALWPGLVQGWGEALGWVFAEQSRFQRSLGRSMSELAAHPGTPWALIGLSFAYGVLHAAGPGHGKVVISTLLVSQPIVRRRALWLSLLAALLQGVSALVLVGLGAGLLDWAGRDVLGQVEKVTLLSHLGVLVLGLLLLWRAARTLWRVARAQPVSSVGAPQAPAMQGLAFKPSPGHDHSHSHSHSHSHGHDHSHGHDCGCGHAHGVTAEQASGDWRTMGMAVLAIGLRPCSGAILVLLAALALNMVGSGVLAVLAMSLGTALTVGSVAMATLIMKASGRLAAAGTRLGGPHSNGRARRQWPWAALVGLLGGGIITVFGALLVASSLKALDSPTGRGASPFDRSAPGTSLQSPLGPRSSGQSSGDK

>tr|A0A2K1QDK0|A0A2K1QDK0_9GAMM Nickel/cobalt efflux system OS=Mixta theicola OX=1458355 GN=COO59_04115 PE=3 SV=1

MLRRTLCLPGRQSLGVIATLLALLALFCWWNWNDFLAWSLATQITLHRYLVLHLLQINNGQYSGGLWLLFFTFLYGVLHAVGPGHGKFVVTTWLSTQQHSSPALRAVPLIGSLVQGLSAILFVFILAVGFNLMAGDLSLSRWVMEKISALLIAAFGGWMLLRGLRSFPHTFAKRKAVTSQHTSADAVHYAPLMAHTPVDAVSPHSTSHHDTHQPCGCGHHHIPLVQPASRKELLGVIIAIGLRPCSGAITVLLFSNAIGIVKWGMLAVMTMALGTGLSLLLLAIAVSRLRDTVAAIWLRESPAATTTIIALVRIAGGVLLLFFALILFLTVVPVSPNGDFIAAGC

>tr|A0A351RRR3|A0A351RRR3_9BACT Nickel/cobalt efflux system OS=Nitrospinae bacterium OX=2026769 GN=DCQ99_07715 PE=3 SV=1

MDSLFYISSAFLLGAFHALEPGHGKMILMTYLISSKGRIIDAILLGIISTFTHTFSILILGIIATLSSVLIIPETIERLTEIIGGILVLIVGVWMLISGFKNNHTHIEGHAHKKREGLIGLITIGISGGIVPCPAALAVLSATIAGGRTADGFFLVLIFSLGLGAVLISMGVLFVKASNFFEKYIGGIFGKKVRMASAVLIIMLGLFLLLKNILPHLFLAP

>tr|C5B6Q0|C5B6Q0_METEA Nickel/cobalt efflux system OS=Methylobacterium extorquens (strain ATCC 14718 / DSM 1338 / JCM 2805 / NCIMB 9133 / AM1) OX=272630 GN=MexAM1_p1METAp0026 PE=3 SV=1

MLLPMAMPSSTRTIPTFATAGPTTPIPTSSTPFTSAGQPCGRAERLPCLPGTSDRGKPTEMLDLLTAVQRGLHTLLTDRIGGFAQTRNLAVLVSMLPFGIAFGTVHALTPGHGKTVLSGYLVGSRLTPLRSLAVSGALTVTHIGSAVLLAVAGAPILSRTFGMFGRAPTLERASHLLLIGLGLWILVRAMRGRSHAHDQRDGVLVGVSAGLVPCPLTLFAMLMAIAKGVPEAGLIFALAMTGGIGLTLGVVALAALAAGRWLQERLKENQTRLRSIGRALDIGSGAALVGLALYGMVQA

>tr|T0PGS6|T0PGS6_PHOTE Glutathione-regulated potassium-efflux system protein KefB OS=Photorhabdus temperata subsp. temperata M1021 OX=1221520 GN=kefB PE=3 SV=1

MEHSAQLNAGVLFLFAAVVAVPIAQKFRIGAVLGYLFAGIVLGPWGLSFIRDVDDILHFSELGIVFLMFIIGLELNPSKLWQLRRSIFGVGAAQVIFTAGVLAGLLYLTDFSWQAAVIGGIGMAMSSTAMALQLMKEKGMNRNEGGQLGFSVLLFQDMAVIPALALIPLLAGETASSDWYRIALKIAAFAVMLLGGRYLLRPLFRLVVRAGVREVFTAAALLVVLSSALFMEALGFSMALGTFIAGVLLADSEYRHELEISIEPFKGLLLGLFFYFGRYVPESGRATDTFVRCVTWRIGFGHCERRCSLSYRMDCGLTGFILLAIFRRSQQGGEFAFVLFSAALGQNVLNSGQMALLLVVVTISMMTTPLVMQLIDAILARRYNAPDETEEQPFVEDNDPQVILVGFGRFGQVIGRLLMVNKIHITVLERDVSVVSTMRRYGYKVYYGDAVELELLRAAGANKAKAIVITCNEPEDTMMIVHLCQKHFPNLHIMARARGRLEAHELLQNGVENFTRETFSSALELGRKTLVGLGMHPHKAYRAKHHFQRLDMRMLRELMPQIQGDVAQISRIKEARRELEELFEREMLNERLQPDGWNEHQHMTSSGANNDSKP

>tr|A0A1X7APD7|A0A1X7APD7_9GAMM Glutathione-regulated potassium-efflux system protein KefC OS=Parendozoicomonas haliclonae OX=1960125 GN=kefC_2 PE=3 SV=1

MDFGFFNQLLIIFSVSVFAIALFHRLRIPDTLAYLMVGIALGPTATGIIDTSFDITLLAEIGVVFLLFSLGLEFSLANVLAMRRIVFGLGGLQVMICTLLIGFCGLMLGFSPVGTLVMAAGLSLSSTAIVSKELTRRNELRSNHGQLAIGTLIFQDIAAVFFLILIPAMAGIGENSLAVSLLLSLGKGLGFVAFMVLFGRWVLPRMFHEIASTKSEELFVLSAIVVCLVAAWLTHLLDLSMALGGFVAGMMLGESHYRHQIETDIRPFRDILLGLFFVSVGLMLNLDLFLENWSMILLASLGLILFKATMIAMLAWFIQNNKKHAIRTGICLAQSGEFCFALVALAGQYGLLEMSTSSMILSITIVSMAATPLLIRYSGPLASRITQQKQSKEPKKAVDVISEQTCDVDRHILILGYGRVGQVISRFLREDNLPYVAIDDDPIHVREASRAGEPVFFGDCRRTELLQAAGLERARMVVICIDSSRAAQAALEGIRSINKTIPILVRTRDDNKMELLKQGGATEVVPEVLESSLVIVSHVLTMLGQPEFSIRQRIQSVRRERYDILHGFFFGQSETLRTQEGEDCELLQGITLADKAWAVGKTVAELPLDEAGIHLKRITRDGEELDIDEQLRLTVGDALLIKGTQKQIEQGEILLLRG

>tr|A3YEU4|A3YEU4_9GAMM Putative glutathione-regulated potassium-efflux system protein OS=Marinomonas sp. MED121 OX=314277 GN=MED121_19614 PE=3 SV=1

MEFIWILFAFVCGLLVKLINLPPLIGFLIAGFALNAYGIQPSDSLNTIADLGITLMLFTIGLKLHVKDLLKREIWAGTLSNMLLWSVLFIAMCLFGFALSLPYFTSLDWQTSALVGFALSFSSTVCIIKLLEESGELKTRHGQISLAILVMQDIVAVIFLVVATGTIPSIGALALFGLIFARPLFGIVLNKAGHGEMLPLAGIFLALAGYELFYLFNVKGDLGALIFGILLSSHPKASELTKSLMGFKDLFLIAFFLSIGFTALPTLDMLVSAFLVSFALILKFALFFGLFILLRLRGRTAFLTALALSNYSEFGLIVAQLSVDSGWLDKEFLVILALAVSISFVVTSLLYRRAHDIYHTYQRIIRSFEKAEPLAVDTFIQPAQADVLVVGLGRVGCGSFHSLNRIMPNSAVGMDADRLRIDRLKAEGHNVFFGDGEDADLWEKFDVSHYKLVLLALPSIEDCSNITIQLRKAGFKGKVAAIARYQDEREPLMASGIDNVFNFYTEAGNGFAEESMQLLAKNNPSLDASPQ

>tr|I0WDT1|I0WDT1_9FLAO KefB-and KefC-like glutathione-regulated potassium-efflux system protein OS=Imtechella halotolerans K1 OX=946077 GN=W5A_08452 PE=3 SV=1

MHFPLLQDIVVILGLSILIIVAFQKLKLPSILGFLLAGIIAGPYAFNLISSSHEVELLSEIGIIFLLFVIGIEFSLKELAAIKNKVFIGGGIQVFGTIGFTTALALLMDIPWNTAVFLGFLFSLSSTAIVLKLMQEKGEVKSPHGKLAVGILIFQDIIVVPMMLFTPLLTGEADNILTTIGILTLKVLLVLVFIYILAKYIAPIIFKLVVKTRNKELFLLTVTVFCFAVAWLTASVGLSLALGAFFAGLIISESEYSHQATANVLPFREIFVSFFFVSVGTLLNLNFFFSHIGTIVLITLGVIVLKITVIALTAMFMHYPPRTIFLTAFTLFQIGEFSLLLSSTGVQNGLLEDAYYQYFLAVSILTMAATPFLMAAAPKLTDYIIQAPIPKAVRRRLKAYKARHVQDTLITEENLHDHLIIIGYGINGKNIAKAARNAKIPYAIAELSPDAFKEAKKNNEPVLFGDAAEDVILQHLHVQEARVIVIAISDPSATKKIVTIIRDYTKTACIIVRTRYVKEIDENLKIGADEVIPEEFETSIQIFTRVLKKYMVPNDDIQGFINQLRSSDYEMLTTVEGINTSLPTRQIRIPDKEVTSLYVESNTNKIVGKTVELSGIRKKYGVTILAIQRDKKYITEIKPDTLIMQGDLLFLFGNPDAINKLNKLFSV

>tr|A0A0U5NV43|A0A0U5NV43_9CLOT Manganese efflux pump MntP OS=Clostridium sp. C105KSO13 OX=1776045 GN=mntP_1 PE=3 SV=1

MHLISSLLFAISANIDSFIVGLSYGIKKANISLLKSTIISLVTLVGTVTAILLGAEISQFLPSSSSQAIGCALLIGLGMYYIIKSLYTYLCIQIKKAEVKASESSSEPKDSSQTEDSLLTIKEGLFLGLTLSINNVGMGIGASITGLKLLPTAILSLIVSVTFLYAGNVIGKSKVPHISDRAADVLSGLILVGLGIYELF

>tr|A0A110A6P6|A0A110A6P6_CLOPR Manganese efflux pump MntP OS=Anaerotignum propionicum DSM 1682 OX=991789 GN=mntP_1 PE=3 SV=1

MFFSLLLAFSLSIDALGIGISYGLRRITFPAASKFLLALETFLMMEVFIMAGRGLALLLPSATGETLAPCFLLLFGLWLCLQGFRKAKEPPSPLASVHQPSVCDKDASQTLDPKETLLLGFILSLDSLGVGISAAASGMEIGKLPVFAAIFQVVFLSLGAFCGKKLTNTEKIRENLWTTISGGILIFIAILRLI

>tr|A0A1F5KFN0|A0A1F5KFN0_9BACT Putative manganese efflux pump MntP OS=Candidatus Daviesbacteria bacterium RIFCSPHIGHO2_02_FULL_43_12 OX=1797776 GN=mntP PE=3 SV=1

MDIFSLFVLAISLSLDTLSVGTAQGLHFHQHRLRNGLKLAITFGLFHLSMPILGWVIGQSLRIFVSQIDHWIAFGLLSFIGIKMVKEALSSKKQVHRKHIQKQTLLLLGIATSVDTLAIGITLAFVEISIFLAGVIMGSVAFCLTMIGFMTGNKIGKMFSEKAELVGGIILIGLGIKILIEHLNGG

>tr|A0A1G6GJW3|A0A1G6GJW3_9BACI Mn2+ efflux pump MntP OS=Pelagirhabdus alkalitolerans OX=1612202 GN=SAMN05421734_10198 PE=3 SV=1

MFYQFLLAIVLGLDAFSVCLAVGLNGFKIKQMVLMSGLIGFWHGLFPIVGFLCGQVIVYYVDDLIDLITGSLLVSLGVYLCLNSFTESVSIKWNKTKLIMMSLTVSLDSIPIGLTLVREPTAWIYSIGLFFTMTMMMSMLGLLLAKRLTVSLHRMSDRLGGVILIGLGLHILVTM

>tr|A0A1M4XWE4|A0A1M4XWE4_9BACT Putative Mn2+ efflux pump MntP OS=Mariniphaga anaerophila OX=1484053 GN=SAMN05444274_103147 PE=3 SV=1

MSILTLILIAIAISLGILEISMSAGKALSRIRFWQAVKIAFILVLLQTPVFLIGWVSGNKFEDLIHSYDRWVPLALLSALGIKMIFESLRHFNNKGKIRSLSVTMLPGVFLAILIDALLVGISFAFFSQKLLLTLLVVGLLTFLATIAGILWGKAPQSKLSFHTKILGGFLLIGICVNLLLKLPPIN

>tr|A0A1S8TB62|A0A1S8TB62_9CLOT Manganese efflux pump MntP OS=Clostridium puniceum OX=29367 GN=mntP_2 PE=3 SV=1

MLESLLLVSSICIDSFVASIAYGTSKIRIPPLSTIIINLICTITLACSLFMGSIFKSFLPGNLPIILGFLLLMIIGIYRLFEYIFKSSISKCSKSDKPLTFKIFDFQFVLQVYANEIKADFDNSKCLNIKESFYLAIALSLDSLAVGFGSSLCNINYLEVLILCFIIGILSVSIGVFVGRKFAQKLHLELSWLSGVLLIILAILRILK

>tr|A0A2N2D8K7|A0A2N2D8K7_9FIRM Putative manganese efflux pump MntP OS=Firmicutes bacterium HGW-Firmicutes-15 OX=2013776 GN=mntP PE=3 SV=1

MTMIEQLVTIMLVAIVLGADSFSLAMGMGLKGVTRSYELKFALMVGIFHILMPLIGLNLGIVTGNLLGVWAGRLGAVVLAYIGGDMLWKAYCETRPQVFRFNQGKQQFTSQVKLAEGWINLTVLTTSVSIDALTVGFSLGTLIQTPVFYTVVTIGLVAGAMTLLGFKGGKLFSRVVGSYAQMLGGLVLLLLAAKMAFYPN

>tr|A0A2S5D4E1|A0A2S5D4E1_LYSSH Manganese efflux pump MntP OS=Lysinibacillus sphaericus OX=1421 GN=mntP_1 PE=3 SV=1

MHWITIIFIGIAANLDNLGIGLAYGVKRVKIPILSNAVIAVMSMIVTFVAVTAGSTVIEYISPHTANLLGSLLLCIIGIFTLFSNRFSKHSIAKNPEVFDEDKNHIISMREAMTLGFVLSANCLAGGIAIGANGISAIWTVISIGTFSFITVGIGSHFGVLLSKTFIGKYSTAISGWLLIIIGVFEVFAK

>tr|A0A377Z8H6|A0A377Z8H6_KLEPO Transcription regulator protein of MDR efflux pump cluster OS=Klebsiella pneumoniae subsp. ozaenae OX=574 GN=yofA_1 PE=3 SV=1

MDRIQAMQMFMRVAEAGSFVRAAETLSLPASTVTSTIKNLEKYLKVRLLNRTTRRVSLTPEGMQYLAQCREILALIEHSESTLSESVARPQGRLRVDMARGHRAFHRHAASAGLLPALSRYLPDDRRQRSPGRSHSGRRRLRDTNGRAEQLQPGGPPSGPLSLGHLRVTGLSSGIWCAAVTGGALSAPGGALFLRSGEAGR

>tr|A0A3D1K6X7|A0A3D1K6X7_9BACT Putative fluoride ion transporter CrcB OS=Lentisphaeria bacterium OX=2053569 GN=crcB PE=3 SV=1

MKQVILFSLYVGAGGLIGAVTRYLCTLFFLKYSFSFPAGTFLSNVIGCLIIGIIIQIATGTELLSPEARLFLATGFCGGLTTMSSFVYETSQFINDGEYFHASSYFFLTLTLSFAAFVSGCIIVKLIMRYGGQYGT

>tr|A0A1V5K2D0|A0A1V5K2D0_9BACT Ferrous-iron efflux pump FieF OS=Candidatus Aminicenantes bacterium ADurb.Bin508 OX=1852832 GN=fieF PE=3 SV=1

MERQNNGNVESENDRIKQGERAGTVGVGVNLVLGTVKLATGLVVNSLAVVADGVNNLSDSFSSLVTLFSFRWAGKPADREHPFGHGRVEYIAALTLSFLVMAVGLQFVKSSAARILHPVPLKFSPWAILLMLLSVFAKLLLGVFYKRVASKIRSGTLQAAAIDSFSDMAITSCVALSLVVPRFTTFPADGIIGMVVALFILYSSFRMIRATLTPLLGTSPDPDLAKDIRRTILEHEPIQGVHDLIVHTYGPEKHFASAHAEVPAGLSTTELHEVIDHVERELEETLRVSVVIHMDPVNPDSEELRVVREEVDRILEHHPSVLSMHDLRIVGQGDQKKLLFDIVLSCSCSLNRAAQEKLTQDIDKELKRTHPFYTTHITVDREMA

>tr|A0A1W9UVF4|A0A1W9UVF4_9DELT Cation-efflux pump OS=Desulfobacteraceae bacterium 4572_35.2 OX=1971628 GN=B6I37_01455 PE=3 SV=1

MDSRARVRAAKIAITTAISLAIIKMITAFATGSMALLSSATDSLLDIMMSFGNLLALRQANKPADDDHPYGHGKFETAATLLQSLLIAASGLFILNESIHRLQHSDNKLAHLNIGIAVLAFSSIVSWFLSRYLKQVGIKTDSSALQADALHYATDVYSNAVLLIGLIGVRLLGWNWVDPVLSIGVGCYILYAAFELLKGSMNDFLDAGLPEEQRKQIVTCITNNESEITGYHNIRTRRSGKFKMVDFNLTFCRFKTIEEAHDSADKIEKEIKQCIDNADITIHLEPTKCTECPKHGQCARSKNIAKTSQQALQDRTSPP

>tr|A0A257B5V2|A0A257B5V2_9BACT Cation-efflux pump OS=Chloracidobacterium sp. CP2_5A OX=2012633 GN=CFK52_04760 PE=3 SV=1

MAAEDTQAALASVAVRRVLWTLLVANLLVVAAKALVGWQAGSLAILGDAAHSLTDAVNNLVGVWLIRAAAKPPDREHPYGHAKLEPIGAFVVAALMGLLSYEIGREAALRLWSGTVAPVAPTPLTFAVMVGALIVNLWVVWHERRAGRRLGSAFLLADAQHTLSDVYVTLGVLAGLVGMRLGWAWLDPVIALVVVAAVGWGAYHVLMTAIDDLMDAAAVDGNALIALARQDLDVVDVLRVRSRGRGAYGFAELTLVFRHNDLRRAHATSDLLEERIRRAYGIAHVTIHLEPAEPAANAPNAGEVSV

>tr|A0A345WS00|A0A345WS00_9SPHN Cation-efflux pump OS=Sphingomonas sp. FARSPH OX=2219696 GN=DM480_14115 PE=3 SV=1

MPASLAGSFLLPSADRSCSSICPARREGGVERGRDANRKPHCQLRAGHQLRQPPVTGAAAAKPTNILKLAAGSILVSLVVLGLKYLAYALTGSVALYSDAIESIINVVTAVAAFFAIRISLRPADADHPYGHSKAEYFSAVLEGVLILVASLAILREAYGAFRDPHPLQAPALGLAVSAGASALNGLWSWLLIRTGRQRRSPALVADGKHLLIDVYTSGGVIVGVLLVAVTGWEILDPILAALVALNILWAGWHLITESVGGLMDTALPPEDLAAVEATINAHMAGALEAHDLRSRHAGRMTFIDFHLIVPGSMTVAASHAICDRIEAALKVEHPDSIISIHVEPEAKRKHGAMTIGSTA

>tr|A0A380NGG7|A0A380NGG7_9FIRM Ferrous-iron efflux pump FieF OS=Veillonella criceti OX=103891 GN=fieF PE=3 SV=1

MQEQLIRWFVKDYDCIKAPAVRTRYGNLTSIVGIVTNLIISLGELIVGFLIGSIAMISDAIHDLADAGGSTISLVSFRFSAKKADQEHPYGHGRIEYLLSIGFSILLFVVAIQLLIESVGRILHPEIVEFSIWALVVMLCAMGLKVWLYSFFKSIGERINSPILKANGLEYLSDVWATLGITLGLVVGGLFQIPVDGYLGAIVSVMIGRAGYHVLADAISRLLGNEPSPEMVKDIANFVKSYPGVLGIHDLMIHDYGPGHVFASIHVEVDAKEDVIKSHSLIDRIERDAQKELYIQLTIHMDPLLVTKESMALYDKIHTVVKAYDEKLSLHDLRAVKSDDKIHVLFDLVVPYSDRQRMDDIAKAIKKILEAMDPTFEITITAEHSYTGDENIHDYN

>tr|D7JCG8|D7JCG8_9BACT Cation efflux family protein OS=Bacteroidetes oral taxon 274 str. F0058 OX=575590 GN=HMPREF0156_00171 PE=3 SV=1

MPNNPAKSYFKFKTQRVIAIASLLIFIGKITAYLITSSVGILTDALESTVNVATGFISLYAVYISLKPKDSNHPFGHGKAEFLSASIEGFLILAAGAVIMFEAVRRLFSPTVIKQLDVGIVIVAVAGLINYIIGWYSKKIGRQHNSVALVSGGRHLQSDTYSSIGLVVGLILLYFTGWQWLDSLIAIVFGLIILVTGFRILSETTSNLMDKADMKLIERFGRLINENKKPQWIDIHNFKLVKYGDVFHINCDLVLPFDTSLADAHREGEELKAVMTANFSEDIVCNLHIDECFVSYCKHCRKADCRLRREPFVEQLDFDIDIFVREKAETPPNQTS

>tr|K6YP35|K6YP35_9ALTE Ferrous-iron efflux pump FieF OS=Paraglaciecola mesophila KMM 241 OX=1128912 GN=fieF PE=3 SV=1

MNVKRVLLIEGCVNLFICCVKLFVGISANSAAVIADAVHSFTDVVNNIMAWMATNIANSPADKDHQYGHQKFEQLAVFGLASLLSIVAFEMLINAYNRFGQAVEQNYLGLIVLTGTLVVNILLTIWQRYWAKKLASDLLEADASHTLSDVLTTIVVIVGWQLAAHGYYWLDTVFAILVSLLIFYLAFKLFQRAIPILVDYSDVDPSAVSAEINRLDSVDSVVRVRSRKVPNGRVADLIVTVDPQLTTADSHLIADEIERVLAKKFNIQDVLVHIEPRHRITDHET

>tr|M7N453|M7N453_9BACT Ferrous-iron efflux pump FieF OS=Cesiribacter andamanensis AMV16 OX=1279009 GN=fieF PE=3 SV=1

MYVLGCKSRHSGPLPAITLAPASCWQQAAFQQQNKKQGQGRIFWAKNRQLFSSFHTFKPYTRRIQRLTSGSLLQDKKKYQGLALLIGALLMLVKFAGWWITGSNAILSDALESIVNVAASGFALYSIGYAARPQDLDHPYGHGKIEFLSAGLEGALIAIAGLAAMGKGVYNLFHPQPVSALGLGIGLTLFTGGVNWALAHMLLKKGRSLNSISMQADGRHLMTDVVTSGGLVLGLGLIYLTGQVWIDNVVAIVFGGIIILSGYRLLRDFVGGILDEADLSLVERVIVLLNRHRSKNWMDVHHLRIQRFGAGIHLDFHFTMPYYFTLEEAHREIDEVTRLVQQNLPHEAEFSIHGDPCLPPHSCGICLKDDCPVRRQPLQRRIEWTMSNAYQNEKHSLQTPD

>tr|Q67QQ7|Q67QQ7_SYMTH Cation efflux system protein OS=Symbiobacterium thermophilum (strain T / IAM 14863) OX=292459 GN=STH1001 PE=3 SV=1

MLSSASGDRRQRLIHRAGLLTIAVNLCLTVARAAAGFLAGSTAVLADAANSGTDILATLVVMGGSRIAARPPDWNHPYGHDKAEPVAAKLVGILVTFAGLATAAGAVQALRAGGEPVGLAAAVVTAVSIAAKEALARYLARLGRRLRSQAVLADAANQRTDVLASATALAGALGGRFGLPILDPLMGLLVSALILRMGLGLYWQAVRDLMDRAPEPETVDAMRRAALSVPGVREVGDLRARVFGPGIYAECKVSVDAGLTVAEGHRIGKRVKEAVMRAVPGCRDVLVHVNPYPGLEEEGDVLPLYPVADELPAVEGAEDAWAGREGEGP

>tr|A0A376MJA9|A0A376MJA9_ECOLX p-hydroxybenzoic acid efflux pump subunit AaeB OS=Escherichia coli OX=562 GN=aaeB_3 PE=3 SV=1

MCDYGGFALFSAIDQTRSGSRAGKFAGRAISINATLYQHGDGEVVDKAWGDLVRRTTALQGMRSNLNMESSRWARANRRLKAINTLSLTLITQSCETYLIQNTRPELITDTFREFFDTPVETAQDVHKQLKRLRRVIAWTGERETPVTIYSWVAAATRYQLLKRGVISNTKINATEEEILQGEPGSQSRVSRTSSCDG

>tr|A0A011QEF5|A0A011QEF5_9PROT Potassium efflux system KefA OS=Candidatus Accumulibacter sp. BA-93 OX=1454004 GN=kefA_3 PE=4 SV=1

MTEPCLARRGASERRFGESIKRLAFAVSFRFFLALLLALPVRAEVAPQPDLTRAIVAENARLVREIAAGTAALEQARSDLRQLRSRRSELDQRMQRIERHAQVNALGQLFAQAVIEQLSRLPSSEGFENDRRQRLDRLEAASDATLRAERALDELADMETATVMRFAASKPPLPDALWPQFEAAARPLLGEQRTLLTGLDEQQGQLLQALQASDAAALELAQRTQAVRAELTRLLFWVPARPSLQTVGEFSRSWAWMTSLANWRAAAVSLAEELASRPFWPVLALLLAVTLLFARARLQAQLVVLAPADANSDRYWIGYTVTALAITLALALPGPLLMWTAATLLAASLDAEPFALALGAALAATGKLLLALSALAWLLDRRGVAGGHFGWDESLLGFTRHALRRFSLLFVPLLLVVTLNGLDHAPFANRESIGRMSFSFAMLVFAAFLVRLLRRRSPLIQRLYARAPRSWAVRLYALWFSAAVAVPLAIAGLSAAGYFVAAGYFFGHTLMSLFLILGAVALYGLIALWVQVERRRLRRHQAREALRQAREAAAETGEDGSEVAEPAPARLDIAAISEQTRSLLDLFITLLLLGGIWEVWKGGLPALSVIGDYVLWTYHATVDGTATTLPLTVGNLFMAIVVGVVTVVAVRNVGALLDIVLLQRFEVQADATYAIKIITRYVLAAVGMVSALSIVGIGWADVHWLIAAMGVGLGFGLQEIVANFVSGLIVLAERPIRIGDIVTVGEVTGTVARIRARATAVVDFDGKEVIIPNKAFITGSVVNWTLSNQTTRLLLKVGVAYGSDIALVQRVLLEAVRANADVLADPSPSVFFMGFGDSSLDFEIRAFVGSFDKRLRVQHELNVAMEAVLREQGVEIPFPQRDLHVRSAPALVGLQESVQGQGDGLPTAPAQAMPSASPSA

>tr|A0A024QBW8|A0A024QBW8_9BACI Efflux protein A OS=Virgibacillus massiliensis OX=1462526 GN=efpA PE=4 SV=1

MSTHSQTLSQPDPKRWKALFLLCFANFLVIMDASIIQIALPSIQESLGYTQESLQWVMSAFLLIFGGFLLLGGKLADLYGNRRIFNLGVFILAVSSLFAGLAWNEISLNIFRGIQGLGSALIAPSALSMVMRLFNFSTPEKEKALAFWGLSGAAGGAFGIVFGGLITGFFGWRWTLFIYVPLSILVLILSPKLLQKSGQRLKGRIDYVGAALATASLMLIVYGIVSAEHSGWTSSNVVISLIVGVVLFLIFLIVESKMKEALLPLNIFKTPNLGIGNVGVFLTQAAWFPLIYMLILYLQQVLQYSPTAGAMAVLPVPLFMAFFIIVVAEKVLAKLGIKMTMVVGFVILGLGNILFSQFATVDGTYVISVLFPSFVAALGNALAYLASTTASVSEVEPKKSGLASGLYNTNFQIGSAIGLAILVAIAGVATASSTAGSQLVALNEGFQQAFFWGGIIAFLGAVLALLFTRSPKQEESNR

>tr|A0A061BZ57|A0A061BZ57_LACDE Mechanosensitive ion channel OS=Lactobacillus delbrueckii subsp. bulgaricus OX=1585 GN=AT236_01558 PE=4 SV=1

MKNLDFKIAGISVDVDNLFTSGVTILWKLLISTLVFYLVSHFGRKIIKRYLDKHNDKLVLSKRSQTISALVNSLFHYTMVFFYVYSILTILGIPVGTLIASAGIFSLAIGLGAQGFMSDLVNGFFILSEGQYDVGDNVEIGTEAGTVTQLGLRTTQIVTTDGTLIFIPNRQISIVRNLTHGGIGLNLDLNLDANTDLKQLASLLDQADEDLLAWHDKLVSGPTQIGVVGQQGQTITYRVHFQVKPGFEGKIRQAYWQTYLQYLRVNQVKFGQEPVIINNSKKA

>tr|A0A077FNP1|A0A077FNP1_9RICK Putative amino-acid metabolite efflux pump OS=Rickettsiales bacterium Ac37b OX=1528098 GN=eamA PE=4 SV=1

MRYIDIMKALLVAVLWGGSFVAAKLVLEYFPPIFSMVIRSIIVISILLSFVGLPNISLKKVLCMSFSYNIGHMVLLYLGIKLGLPVSAAVIATQMQVPITSILSIIILKEKMHLKQVIGMLISFIGIVIIVGHPSIMDKIFPFMIVIIAAFFFALFNIQIKQVGNLNILSYILWSSILILPQLMILSYISEPISWSSIFIADVKLWLSIAYISMVNIIAFLLWISLLKIYPVSLVMPFALCIPVFAILGSALILSEYISWHLIIGGTITLIGVAIITIKFTNLTRYQST

>tr|A0A083ZVB9|A0A083ZVB9_9GAMM Putative amino-acid metabolite efflux pump OS=Serratia sp. DD3 OX=1410619 GN=eamA PE=4 SV=1

MSTIWFIVAIGVPFIWGIQAVMLKFGIGQFPPIFMVSLRFLFMFILLMPFLSRLKGQFRLAATVGFTQGVAHFALLYIGFKYADVTSGMIVYQTNAIFTLLLGSILLGEKMTKYAMSGTAICLIGVSLILGMPQENTNITGLFIIACSALMFALGNICVRKFGPFDPVGLNATVSLIAFPALLFISFLTEKGQLESLRTASLEAWGALFYTAIFGGVLAFILWYKLLIKFSVDQISPFSLLMPFFAMMGSIIILDEKVTIVNWIGAVITIGGIAIIQYSNKLVTLKGRRSVIVTDQ

>tr|A0A086P4H5|A0A086P4H5_SPHHM Auxin efflux carrier OS=Sphingobium herbicidovorans (strain ATCC 700291 / DSM 11019 / NBRC 16415 / MH) OX=1219045 GN=BV98_003876 PE=4 SV=1

MAMTIFGALMPVFGLILIGYVCGRYDILGDRAFEVLNRFVIAITLPILTFRSIAHMDPANLAVPGMFAAVTLGALLTYGIAFAVERHFGRHGSETNIAALCACFSNTGFIGLPIALLAWGPEAAAPVSVAMLIYSSIVFTVGLVMSEVTASEGHGPAAGLKLAARSIVRNPLILLAVAGCLWSIFRLPLTGPADILLATLAQATAPCALTAIGIFIALPRRSATPGPIGRVVALKLIGQPMITAAILWMLPPIPPLWAKVAILMAAMPSGASSFVLAGKAGRWAMELSAWAVMLTTTLASISLIGILWWLGA

>tr|A0A087C0A6|A0A087C0A6_9BIFI Cation efflux system protein OS=Bifidobacterium mongoliense DSM 21395 OX=1437603 GN=BMON_0842 PE=4 SV=1

MMRWQRMNLIAKLVLLTFVLLSVGTRVYQFVDPNVPTAASSIDYFLNVNAFATPEICPFLIVFCAGATRYEAFERVRVSAHHELGANVIWMVQCAAAVALFGLLSWCGVEFLLSGRTWDIPLGVSVAVFAQMLMNTFMELIVIGFVQFLPINAGVSWGKAMSAMLLFLLVCNWVFHNWAEIIPETLFYFLPLVPPTFDGLASAKLPPFVGAVVLLVHGQPVPL

>tr|A0A088MZ42|A0A088MZ42_9GAMM CO2+/MG2+ efflux protein ApaG protein OS=Candidatus Baumannia cicadellinicola OX=186490 GN=IM45_1239 PE=4 SV=1

MMTNASRICIKVQRMKSQLEENRYVFTDTITLQIMGRYLLITNAYGQETEVQSKDVIGEQLLIIASGKFQYTSGGVLETPLGTMQRYYEMLDYEGQSFRVALLIFRLAIPTIIN

>tr|A0A090JL79|A0A090JL79_9FIRM CHR family chromate ion efflux pump OS=Peptoniphilus sp. ING2-D1G OX=1912856 GN=ING2D1G_0018 PE=4 SV=1

MFLKIGILAFGGGYASIPLIQRYIVDDYHWLSMLEFLDLVSISQMTPGPIAINSATFVGQKVAGLIGSIVATLGFVTPQFTLMMILGYFLFQKNKKFKLLDWMLNGIKAGIVSLIFITALQLFVSSVFPEGFGSINIAAAICFIIGFIMYLKKYSIFQLVAVGATLGIIINCAFKFL

>tr|A0A090QRC9|A0A090QRC9_9GAMM Magnesium and cobalt efflux protein CorC OS=Photobacterium aphoticum OX=754436 GN=JCM19237_1051 PE=4 SV=1

MITAKQLLHYQQAAPHGDITPFISPVECIPEHWSGSQMLEHFRQTGASMVFVVDEYGDIQGIVTPTDVLEALAGEFRHPDPDDLWSVEQDDGSWSIDALIPILVLQDLLDLKRLPDEQRGGYHTLSGMMMWCLDGIPKEGDCMNWEGWQFEVITLAGNRIDKVWAHRLAGESSLTITDHPSEEEQPADGPTPSMASTVHQPDHHEDTENHPENAPKPRAKAGQK

>tr|A0A094ZQ85|A0A094ZQ85_9PROT Mechanosensitive ion channel protein MscS OS=Acetobacter tropicalis OX=104102 GN=AtDm6_1231 PE=4 SV=1

MVMENQVHSIWSQLNGLLPVVLGYVSQFALALLVLFVGWKLVNAVTRAMGRMMEASHIEPTLRGFLLSVVGLFLKALLLISVASMVGIATTSFVAVLGAAGLAVGMALQGSLANFAGGVLILLFRPFKVGDSITAGGSSGTVTSIEMFRTVLRDANNEIIYVPNGTLSNNIVVNSSETDRLLGSVTLLIDYNDDIDKARALLLGLTEQDELVLKNPAPSVSFLPKAANIQVTLGFWCAPGNVAPLVAKYSEAAIKVLQKEGYRLGVTARTAA

>tr|A0A0A1W0B9|A0A0A1W0B9_MICAE Auxin efflux carrier OS=Microcystis aeruginosa NIES-44 OX=449439 GN=N44_04073 PE=4 SV=1

MLAILSAIVPVGFIILIGVIAGKILTVEVHSLSQITVYILAPALVIDGLYHTTLSDSNIGLIILGFALISLVMAIVVEIMAYCLSLDGDTRKSLMAAAVMPNNGNMGLPVASFALGAAGLERAIIYMIGSSILLFGISPAYLQGKSFLSGFRLVFQLPLIWSIFIGISFQTFSFHLPLQLDKSISYLGQAAIPLALIILGIQLSQQKLAIGKLELLGACLRLLVAPLLAFAIGNSLGLTGMDLKILILQSAMPTAVNTVILVTEFGGSATLMARTVVVTTLASFLTIPFFLWLLKVYL

>tr|A0A0A8R1A8|A0A0A8R1A8_9ACTN Actinorhodin transporter OS=Propionibacterium freudenreichii OX=1744 GN=PFCIRM512_08510 PE=4 SV=1

MTQTLTAVEPTTSSRLGRRLWAILAVVLIADAIDLMDSTIMNIAAPTIQREIGGEEGLIKWLGASYALALGILLVVGGRLGDRFGRRRLFLIGIAGFGVASVLCAVAIDPAFLIAARLLQGAFGALLIPQGIGILIATFSREQFPTAASMFGPVLGGASIVGPILAGFLVGANIGGLTWRPMFLINIVLCAAGLIAGWKLLPPDRDLQKVSIDGLGSALLAVGMLGVLFGLIQGSTNGWTAVPVICLGLGVAGFIGFALRQRLATNPLIVPALFHNRGFTSGLLIGLGYFAVVNGFAYVVSLYFQIHLGLSPVGAALAMMPMMVGIIIASFGARPLIPKLGRNLVVAGLATTLAGIVALIAISIGAGDATNQWMLAPAILVLGLGMGASFSSIYDVAIGDLTTDLAGSASGSLSAVQQIASAIGSAVVTTIYFQTSATADANRPFIASLIVVGAITVVCLIAAPLLPKRAPQDAH

>tr|A0A0B0EJD1|A0A0B0EJD1_9BACT Heavy metal efflux pump protein CzcC OS=Candidatus Scalindua brodae OX=237368 GN=czcC_2 PE=4 SV=1

MDLKNIKNGLYFFSKLLLTGLFIVATFSGITIAEDRDDILRIRWVIDEALRSNPELNSARLNWDASKERVPQVSALDDPDLGFTYYGEQTQTRVGEVQAGFMASQKIPFFGKLRLRGEIAENEANAIGERYRALERDIVAKAKSAFYELYWVHKSIRINEENRELLQRFVKIAEIKYASGKATQQDVLKAQVELSDIMNELITLEQLKETAIARINTLLNKHPETPLGIPEEVDITEFDVPIAELYKEAKKISPEIETFKYRIERDKAAYKLSKKQYYPDFTLGFNYNLVNDLPSSVMMSPVGESRDSYTGTLSINVPIFQKRKYDAGVREANARLKSSEKAYRNMENKTLFEVKDFHFRTQTAERLVKLYRGSIIPLAEQSLKAAEIGYQAGRVDFLNLIDSQRVLLNFNLAYYRAIADFGTNFSELERVVGVELSKKPQSKKP

>tr|A0A0B4XLV7|A0A0B4XLV7_9GAMM CzcA family heavy metal efflux pump OS=Alcanivorax pacificus W11-5 OX=391936 GN=S7S_14210 PE=4 SV=1

MLTAPIVTRWNRSPAIRQDVDAAMIDGTLKCLRSMLMTMVTTTFELMPLLWKSNVGADMSARIMKPVVGRLWFCMSLTPLGTAGGLCHLVSLAVQRLRAPSRRPQSSD

>tr|A0A0C1NW99|A0A0C1NW99_9PSEU Arabinose efflux permease family protein OS=Prauserella sp. Am3 OX=1515610 GN=HQ32_04585 PE=4 SV=1

MTTSPTPEVRRRSGTLVAVICCCAVIFDGYDLSVFGTTIPALLEYEAWNLDAARAGVIASYAFMGMLVGTLICGLATDLLGRRRMLITSMSWFSVCMGACALAPNPELFGLFRFLSGVGLGGLLPTALALTAEFAPRGRRNLFNALVSSGFSVGTIAASLIGLVLIEPFGFRPMFAIGVLPLLTLVPIAYAVLPESADFLRSKGKHDQAHRTALRYGLAENATPHGESDEDGRRAGLRDLLRRPLLTTAIVFAFAGLIGQLFIYGLSTWLPEIMRSAGYPLGSALSFLATMSIGAIAGATVMSICADRIGPRTVAIWGFGIGVLSLVTMSLAPPTPVLYVAVALAGVGANGTAVILNGFIATWFPAAVRATALGSIMTVARLGGIIGPILGGLIVAAGVDVKWSFYVFVVPAAIGIGLVLLLPRRHLDGRTLGAAREPQPAPVRGGQA

>tr|A0A0D6EC09|A0A0D6EC09_9GAMM Cation-efflux pump fieF OS=Halomonas sp. R57-5 OX=1610576 GN=HALO3634 PE=4 SV=1

MKSESSTLSFSAFMALLIGCAGIVATLASNSQAILLDGLFNLIYFSVALVTIKVSKLASRPDSESYPFGYSYFESLVNLCKGLLILGVSIFALVDAIAALLTGGREIAAGLAVLYALFATAACSLTAWVMHRSQRHVSSPLVAADKLNWLVNSVISAAVLAAFCLVMLFERFGWQAILPYVDSVLVIAVVVLCLGVPVRMASQALRELLNKTPDETIAEPVRQAVARGLADIDTVEVRVRMVRPGRLLYVIVHVVLPEASDVSVTRQDSLRVRIDDEVRRYYSPVVCDVVFTTNTRWAAPSCGLLVEKHPA

>tr|A0A0E2UDU2|A0A0E2UDU2_9STRE Cation efflux family protein OS=Streptococcus parauberis OX=1348 GN=SS13_contig00001-0180 PE=4 SV=1

MLPLYREKLKRGIHDQKKINNKSIERKALAVSTIVNFITAIAGIIIYIITGLNALLLDSVFSAIGCASTLAGFYITKNSHRKTKNFPNGMYFLEPLFGILKSIATLMLLIIASLESATVAYAYFFKNQGSPITIGPAFPYAIITGTMCLLLAYYNHKKNKSINNLSTMLQAEIKANFVDGIISYGIGLTLLMLYFIAIDGKLGFLHYTGDFFITIMLVAISFKEPLMVLIHSFKEFAYSTVQDQEIKASIISVFKAELPNHLENLDITIYKQGMQINVRVFIIGIDNTDTIEELALEKANLLNHLRKEFEQISLEFTF

>tr|A0A0F6RC18|A0A0F6RC18_9GAMM Cation efflux protein OS=Kangiella geojedonensis OX=914150 GN=TQ33_1072 PE=4 SV=1

MSTLKSAILLVAALLFSSVTNSQAETNGYGYTLESTLIMAIENDPWLRKSQFKELSLLSQAESQASLPDPKASINLANLPVDDFSFNSQPMTQFKLGFSQTFARGDSLDLKREINQLQASVEPTMRINRKNQLIMVVGALWLDVHKAQQSIALINDKKYLFEELREAAEISYTSALSQTSQQVIIRADLELAKLDDQVTVYSDKLHTSIRQLSEYIQLPQSYLSDREYGSVSFVSQSLPRMTLIEKKDGFQQHPLIKSVDQSIKASQKVVNLKKQSYKPQFTVNGSYAFREDAADGMQRPDFFSLGVSFDIPLFTGNKQDLDVQSAVYDKEAKLEEKALLLNHLMSAYNTELSRLNKLKERHKLYSESILPQVKHQGEASMNAYSNNEADFAELVRARIDEVNVQLTALDIAVEIEKTKLRANYYQAKTPSELLAQISSSYKTTDVTDELSPSNNPINSGDRHE

>tr|A0A0F7M318|A0A0F7M318_9GAMM Arabinose efflux permease family protein OS=Spongiibacter sp. IMCC21906 OX=1620392 GN=IMCC21906_02232 PE=4 SV=1

MSISHTKDKPPAKVFFALYLAMMAVGMGQTVVFAIIPMLGRELALDKLVFQLPILDITLAPKELTITSLSALAAVVFFFAAPFWGRLSDRVGRKPIIITGLLGYALGSMVFNFASYLGLAGILSGVALYFLLVISRGFHAMVMSGTHPGAAAYMVDITTVSGRTQGMGKLQAANQLGVMFGPVLAWFVSVSFLAPLFIQAVLATAAAILVWLWLPSIPPQPAADTRPRRMSYFDARYRLFIFVGFVLFSLIAMIQQTLGFYFQDTLSVDGVRSAQLFSVAMVVSSAMMIFAQLGVVQRFGGAPVTLLFAGMPFSLLSYLVLANADNLVMLLGGMALFGFGMGLTGPSFTACATLVVRAEEQGELAGMLGSIIGLGFVFGPLLGGALYSISPSYPYWAAALLIVVMMATLFMHWRAGHPSLVPSAVIKK

>tr|A0A0G0A4F2|A0A0G0A4F2_9BACT Auxin Efflux Carrier OS=Candidatus Roizmanbacteria bacterium GW2011_GWA2_33_33 OX=1618476 GN=UR42_C0013G0004 PE=4 SV=1

MNFNNFQILLSVFSLIFVGYISKAFKIIDEDFARKAIKFLFFLPLPILVFLSFATTKLDISLGIYPIISIIIQSILIVISYFIGKLLKFDNKTIGTLIAASGITSTLVFALPFIQAFYGIENLKYLFMYDFGNGLMAWTVVYLITGYLGNKKQLGIKKGIISFVKNPMIFALFFGVIFGLLNIKLPQFFTQFKTTLSGFINPLLFVSIGILLDFKYFLSKENLTKLFLSAGIIMGVSVLLAFMFTSLFDISGIGQKVILISAVSPAAALAVAFSVEHDLDQKFASALVAFTMVLGIILVPLIILL

>tr|A0A0G1BLV0|A0A0G1BLV0_9BACT Arsenite efflux ATP-binding protein ArsA OS=Parcubacteria group bacterium GW2011_GWA2_42_14 OX=1618820 GN=UV01_C0003G0005 PE=4 SV=1

MGKTSMSAATALGFAKEYAKRRKNDKVLIFTTDPAPSLADSFGQKIGNEPTQIEGAKNLFAMEIDAKKVLEEFKKEYGEDILDILQEGTYLANEEAEELFSLDIPGLDEVMGLKKITDFMDSQEFGLYIVDTAPTGHTLRLLTLPELLDDWIKFLASLRWKYHAMVRQFAREERVTKADQFLLEMKKTVKKVRELLQDEKRTEFIVVTIAESMGVRETEDLISTLEKFHIPSRHIIINNIFPKEDSDFAKFKRKNQEKYINEIKKKFSNHAITEVLLQADEIQGIKSLESLGKQLFV

>tr|A0A0G1H920|A0A0G1H920_9BACT Auxin efflux carrier OS=Candidatus Wolfebacteria bacterium GW2011_GWE2_44_13 OX=1619017 GN=UW32_C0003G0106 PE=4 SV=1

MLSTIFVSLPIFFIVFAGWLFQKMKIMQGDWVHQANAFAYYVALPALITVSLWGVNFRSPEILSLLGVSAVSMVVFLALLFIVLSVWKVSRETKAALFLTAATGNTLYMGIALVEAGFGKDHVPAGALVGNVYLIIPLVLSMLVVNYWHTKEHSIKNELMEFLKNPLVLSMVLGVVLSFIPEAGSAIIGSIKKTMTMLGSTSSPVALFALGGFLYGKFLKKDLHLVISIATIKMILFPLIVFGTYIYFGKGGDVEIPVLLASMPVAVTTFVIAEKFKLNTALVGNAIVFATILSFITTPIILLLLR

>tr|A0A0G1NKG4|A0A0G1NKG4_9BACT Cadmium efflux system accessory protein OS=Parcubacteria group bacterium GW2011_GWA2_45_14 OX=1618832 GN=UW94_C0005G0072 PE=4 SV=1

MLTKKQAEIIKNDVSKGSVQLAVMLNALSDTGRLKIFRLLTKYEDLCVTDLANVLEVSVPAASQGLRIMELSGLVKKERRGQMICYVVARDMPLVRALQRIVTGVHVTAKDSR

>tr|A0A0G3V367|A0A0G3V367_9ACTN Arabinose efflux permease family protein OS=Actinobacteria bacterium IMCC26256 OX=1650658 GN=IMCC26256_111595 PE=4 SV=1

MTTTSEPLDSKQGSNQLGGRGLRFWLPICVLAFAQFVMVLDSTVMAVSISAVVKDLGTTVSKMQLAIACFSLVMAAFMLAGAGLGNRLGRKRAFVIGLVIYACGSFTTALAPTFGALFIGWSVLEGLGAVLVIPAIAALTAANYSGKQRALAFGIIGGISGAAAAAGPVIGGWVTSAYSWRYIFASEVVICLAVVALSRFIKEGERPTVGKGFDFLGVALSALGFGLIVISLVQAGIWGWVMPREAPFTVLGFSPTIPMVVLGIFVVWIFLASQARLKESGGTPLLDPELLRIPSLSGGLATLGVQQFVIAGLFFVLPLYLQYVLGLDALESGLRILPLSVSLLVASFLGAALSSRFAARRLVRVGLSITILGVVFCIAAVDVHLRSNLFALAMSITGAGIGIVASQLGNVTQSSVNVTRSNEVGGLQGTAQNLGASLGTALIGAILLSGLNSAFVRTVGANPKVEPQTKVAVEAASKQGIAFVSEDQARAAAEASGLSSSEVDTVVNDYVTSQIDGLKAALGAVAILGLLGLVASRRIQNHPLAGDDPEELDELENEEIVKNQ

>tr|A0A0J1IQ49|A0A0J1IQ49_9FIRM Threonine and homoserine efflux system OS=Desulfosporosinus acididurans OX=476652 GN=DEAC_c14840 PE=4 SV=1

MNNVRGKGHGGGIAAIIISALGFAFYPIFGKFVFAGGANLATVLFVRFFIGAIAFWSLVILGKSIKHLTKRDFFRLWLLGGVVYAGQAGLYISAVKFIPSSMASLIFYVYPVLVTILALATKQERLSISKVSGLLFSILGLILVLGVSFEGLNLLGIFCSLGAAAIYSIYILTSNRIINSVSPLLSSAIITSAACVTYGITGLIQGFTWNIAAITWMDILGIAILSTIIAILTFFWGLQKVGPTTASIVSTLEPVLTVGLAYIFLGEYLNFTQSIGAACVLLGAVLAAWPHKIMTISVKEAKL

>tr|A0A0J8DEE2|A0A0J8DEE2_CLOCY Auxin efflux carrier family protein OS=Clostridium cylindrosporum DSM 605 OX=1121307 GN=CLCY_10c01100 PE=4 SV=1

MQYFNILNQIIVLFLIMGVGYVAARFKVISQEVNVGLSKILINITLPFMVIASFNFKFSQKMLSSGLILFALTFIVHGVLALVSLMLFRKCDSGKKSVLRFMTVFSNCGYMGYPLAHSVYGSEGVFYTAIYNVVFNIFLWTVGVMLFQKEKRKGLYKKVFKNPGMIAVFIGMIIFIFSIKLPFAVSNTISLIGSMTAPLSMIIIGVSLYDVNLKTAFKGIEYYGASLMRLVITPLAIYAVLSLLGFTGIILGISVLLSAMPAAATTVTFAQIYEGDVESASKITVVTTILSAVTLPLIMLLV

>tr|A0A0K1P877|A0A0K1P877_9DELT Magnesium and cobalt efflux protein CorC OS=Vulgatibacter incomptus OX=1391653 GN=AKJ08_0028 PE=4 SV=1

MRDSRAERRRGGPIGPPLLLLDRAHLTSSADFGLCSADGMTLQAPFPALALAAIVARFFFVAAETALVAVSPERADELHAARGSLAARSLLALKRDVESSFATTRVGSISALALGAGLAGIACASWLGAESAVLSAAVGGLAAALLCVLADTVARSLAIAAPEAWALRSAPPLRLASLVVAPPARAAQAVLDRLLSPLGVRATFKGADPALEDIERILLSESHRDGPAPELVHSLFEFPSRTVRDVMVPRTEVVAAPLGIAPEALVRLVAEQGHSRLPIYDGRIDRIVGVLHTRDIVPLLAHPELIQLADAIRPPVFVPWAMRIGRLLRQMQRDRIHLAMVADEHGGFMGIVTLEDILEELVGGIRDVPSGTEVVEADGGHLVDAGIPVVAFNEHFGTALPEHGEFGTLAGFLNGLAGEIPEVGATLESHGLAFTVADRNATRVLRVLVQAAVSAKRNSA

>tr|A0A0K1PPP6|A0A0K1PPP6_9DELT Putative transmembrane efflux protein OS=Labilithrix luteola OX=1391654 GN=AKJ09_02162 PE=4 SV=1

MRSQPSLAPLYGAAFVLAVDTAGVGHLLPGLEMATHAPPRGASWFISIYMLGALLGAPLLARIATRRGRTTVLAASLAMFALASFVVGLSSSYPLTLAARFVQGVAGSPVMPLGAAHLSALAPAGKKGRGLGLLSLSYSGGFLAGMAFVSLFLLVSFRLAYLFTGALASVACIAVARLPVAPTTSSSHDDAERPPPEVSTIAGWFVALALLALAINQVNVDGAGAIAMARFAAVPALALFAWRDRSVRAPLLPRDLFRSRVGLASASLALAAGVGQACVVLLPTCGMAELGVSAAASGPLLIPIVLGGLGTNLLASARLDRWGPKPFLAAGLLGMALGNLVTAWLGTNLLAFEVGALLLGAGVSAMSSGALRYLATLYGAADDADANQAAISLLTNVGVLVGGSLWGAIVPSTSGIGVAAVRVGIVVLTAALMPLSVGVLLVPSRR

>tr|A0A0K2Y7W2|A0A0K2Y7W2_HELHE Potassium efflux system KefA protein / Small-conductance mechanosensitive channel OS=Helicobacter heilmannii OX=35817 GN=HHE01_07640 PE=4 SV=1

MRFLLWALLVLFMGFLRADTHEGGDLDLIDLHLLLKQINQLNQVITRYQKDPTKHTEVSLYSDQKNELMHAFALRLLNSQEKIGINIQENTKQQHALQKALLKSSKINDFYAYFSQTIQLKNLEVEAQMYGFLEKIRTSTDLFSQERDIKNITSTYLLKLEAFATRTYTIPEHLADIKKHELLGALENYQIKLQTYTDVLRYIQKHPKDVLAKNVVFNINMQWVLERIAGAVNHIFPDMNGLQNAKILLSLGFLVLLLALRQIVTAIFVKALDYCVRFSRKNANINIQEKIRNSILAPISTFLFIYSFDISIDILYYPHPAPPRFDMYLGVVYVSLIAWLVIALFKAYGAAILATLASRKNGFRKEVINLILKIAYFFIFVLTILGVLKQLGFNISTIIASLGIGGLAVALAVKDVLANFFASVILLLDNSFGQGDWIVCGDVEGTVVEMGLRRTTIRGFDNALFFVPNSELASKSIRNWNRRKMGRRIKMIVGLTYGSSSEALQKCVLGIRQMLEQHPQIAKASDLENITDHDHHDYIMERQNIVSFNDLMGYKSNLFVYLDSFGDSSINIFIYCFSKSVVWGEWLAIKEDVMLKIMKVVEDCGLSFAFPSQSVYIESMPHPS

>tr|A0A0K8PUF3|A0A0K8PUF3_STRAJ LysE type efflux protein OS=Streptomyces azureus OX=146537 GN=SAZU_6288 PE=4 SV=1

MTVDLVGFLGVVLVAYVVPGPDFLVVVRSAAEAPSKGRAAALGAQTGLCVHMLAAAAGLSVIATRSPVVYDGIKLLGAAYLVHLGVRALLTARQAARKQRHANEDAAGAPPQDSAHPPGPPTPKAGPVPGRWQSGFTQGFLTNVLNPKAALFFLSVLPQFVDGHGSMAHQIFFLGILDILIGVVYWFGLVVVAARLRALLARPKIRHRWELTTGWLFIAIGISAAAVA

>tr|A0A0M4DAA6|A0A0M4DAA6_STRPR Transmembrane efflux protein OS=Streptomyces pristinaespiralis OX=38300 GN=SPRI_5798 PE=4 SV=1

MSTAPSAGRAGRSAHRNTGRDEDRSAGRGDGPTGESGPTGESAGRSPGRRAVHRRITLAGSVVGASVVALDGTVLTIVQPVMQRELHASFEQVQWTGTGYLIAVASLLVLAGRIGDRFGHRQVFAVGTLGFGAASAGIGLAPDIGWVIALRVAQGVFGALLQPATLGMLRAAFPPDRLGMPIALRTSAIGAAAAAGPLVGGALAAELGWRSVFLLNVAPALVIGLLVLAVRDPEPVEAPRTGLDPVGACLLAVTLVCLVHTLVGMPVSGRAAVTAAGCGAAVVAGAAFVRHVRRGRDPLVPPEVLGSAAVASALGVLVCASAVLSGSLFAGVYFLQDVLGLDPFRSALQALPGAVAVVLGAPVCAVALRRYGPRRATAVAMTLLVLGALALSRLERASGAVPVGGGFFLVGAGFGAAMVAATAVVVRGAPAEHAGVAGGLQQTAMNIGPVLGVAVATALMTLAAPRGGPAGSVVMSVSATGPAMTVLAAVAAAGALLAAVLPGPSGAAPPRPRTAP

>tr|A0A0M6ZSX5|A0A0M6ZSX5_9RHOB Homoserine/Threonine efflux protein OS=Labrenzia alba OX=311410 GN=LA5096_03432 PE=4 SV=1

MTVLEYVLIGFLVGILTTAPVGPVNVMAIQHAVKNGFSHGVFVGLGAVVADTIFASAAIFGVSAVTNFVDSRFGLIEIVGGALLIVFGIRIWNTHPHIEKDGNGREYSYLGDAAAAFFMAITNPGTIFAFAAIFGVLGDYRPAHDDHFGSLLMVAGVAGGATSWWLFVSATVSHFKSRIDDRWLGRANHIAGFVLIVFGGLIYLNLALDRLGHAAGIWGH

>tr|A0A0M7F1Q0|A0A0M7F1Q0_9BURK Efflux system membrane protein OS=Achromobacter sp. OX=134375 GN=ERS369989_00840 PE=4 SV=1

MPAISMPNRSRSYPVLSEFAIAGIYVPPFFLYACATVPLYWLLRVVMARSGLLRRVWHPALFEFAVSLALVSLLILTL

>tr|A0A0N0XLB5|A0A0N0XLB5_9NEIS Glutathione-regulated potassium-efflux system protein KefB OS=Amantichitinum ursilacus OX=857265 GN=WG78_08825 PE=4 SV=1

MNFLNQALLILALPLVVWWPLRRVMPLVLSQILCGIAIGPSVLGHLAPTAFAQIFPSGTLGALGGISSLAVVCVGFIAGMELDIDELRHQARHVVRTGLASFAIPALGGAAFAGWALTQWPALAGAHATQGAFIAAGAICFGVTALPVLVAILRELDLLGTAMGRVALSLAAINDMLLWVGLAGLMLVISHKTQTLGNWWWVLVLLLLTLVATLFAIRPKVSAWLADLDAGKELSTARFTLVLIGLLACAIITDTLGLHAVIGAFVAGVLMPKALRPRITQLAGPFSNALLLPFFFVSAGLSLDITAGPVWPFFIASVVIGSALKIISTAVPARAWGWRWAQALQLGALMQSRGLMEIIVLRVLYDAGVISQSCLSALMLMALVCTGLAMPLARWLDRQSAAPATAATIANLPAAR

>tr|A0A0N8KBL8|A0A0N8KBL8_9GAMM Auxin efflux carrier (AEC) family transporter OS=Idiomarinaceae bacterium HL-53 OX=1298881 GN=HLUCCO02_11535 PE=4 SV=1

MENLALILAYLAIGWFLQITRSLPEQSGHVLNQYVIYVAVPAMVLIHLPQLEVSRSVLAPLFTPWAMFGLAIGLVLLFSRMLHWSKELTGAMLIVVPLGNTSFLGFPMVTALYGEQGLPFAVLYDQAGSFLALILFTSVIAARYGQYQQNQGTNEPPSKRKQALKLLTFPPLLALITAILVGQETYPGFVQPLLESLAQTLVPVVMIAVGLQLKLKILRADLVPFTIALGIKLIVLPLVTLAVFASLNLTDLAAQVTVLEAAMPPMITAGALAMAAGLKPRLVAAIVGYGVLLGMITLPATAWLTGVVLGTA

>tr|A0A0Q0TW72|A0A0Q0TW72_9CORY Leucine efflux protein OS=Corynebacterium oculi OX=1544416 GN=leuE PE=4 SV=1

MDIALLMSFWGLSILMTCIPGPDWGLILRHVIGSESRAAVNQAVAGIGAGYVLMSAVVAAGVGVIVTQHPAVLTAISLGGAALLIYLGATLLWGLRPAARVAAGAQGQAQGNKASSPVWEGMGVSLLNPKALMFFVAMLPQFVNTQAAWSVSTQMFTLGMAFTVSVVVVYTCLSLAARRVLRASNRAVVVMQGAGGVAMLVLAAVMVGRW

>tr|A0A0Q9Z1S6|A0A0Q9Z1S6_9COXI Putative efflux system component YknX OS=Candidatus Berkiella aquae OX=295108 GN=yknX PE=4 SV=1

MMQMSQALAQVTMQITNSKPLEKILYDGNLLAVNLLEIQSPANGMLLKKHVHFGDKVSQGQVLFEFASQELQAQLFEASMAVIENKEAYLKLRDWEQSYEMMQANSQMDKAYHELARTEVRFQQTKKLYQSGIVAKEECLLDERFYKDSQQHYQNAKRQLAQIKEKANATALKLAELKLKQAQNKEAMLQNKIAALIVRSPMSGTVLAPHVEGTKQVFALYPQKPFQEREVIAWLADMSSLCISVKVDEFDIVRLQKGQQAKVVLAAFSTHSLAGKIMDISVQNNPANGTRQAMVYDVKVALDVIPEEIQNKLLIGMTASIQLEERLPEGLWIDKTAIHYENDEPYVNVIHDQTSTKQKVVLGDNVKNEVRVIKGLAVGDRIVLHG

>tr|A0A0R2L2N1|A0A0R2L2N1_9LACO Efflux transporter, HAE3 family OS=Lactobacillus pobuzihii OX=449659 GN=IV66_GL000910 PE=4 SV=1

MSKIFGKLSANIHQHAKWWIGIILAITIGLAFGLPNLEIKMGNDVFVSNNSAISKDSNKYMHHFGGDSYYIMQSGKQSDILSHDNMQELSKFDRKIRDVDNVRGTTDLVTVMNQELANAGKGKSDSLSGQFDMNNSKLQKDLMNSLSDKQKNKLQNQIQASLTDQQEQQVQKYVAGQLSDQQQQKLQNEIQASLSNQQKQQVQSYVVQNVLNDQQKQQMAQAQQSGSADPAQMQQMMQKALNQQQQVQVQKYTQSILTSKQQAMMQSALNDQQQDSLQKYTMSILNGQQKNSMVKTVVPMLPKVQNMSTALLRDIFLSDNGKVPSEMEQMLPKNGKMNLVLINTSEKASSMDTDVQLNHDINKVIKNANFSDGIKVKLAGQPGILGQIRTEVLSSMITMFAIAIVIMIIVLALIFPVRRRLLALLFVVPSLIWTFGLMGWFDLPITLATMATLPIIIGLGTDFGVQFHNRYEEEFRKRKDAKKAAKESIHKMGPAVGVALIVMTFSFLTLFLSRAPLMQQFGLTLAIGVISSYIVEFSLMFGSLSLLDSDKRKKNKNKKNKQTKIKQPSILSKVLARYANFVTHHAGIVMLIGVVLGVFGFSVEKNIDVETDITKFIPQNMTALKNTKALQNNIGSTTYITYLVDAGDLDVRSQDNIQTIDKLGKKVDNKYSGVTDVQSISSQYKSAGGKLNASQSDIDQQIKLLPKALTSTMISDDHHYATMQFKVKEDLSSADQLILMNKITKTLKHGNGDLKISPAGAQSMMLVAIDNISANHTLIELVGLAVIFVILFLIYKNWRVALYPVVPILIVLGLSPLTLWLIGTPYNPLTITLSALVLGIGTEFTILILERYREEYSKSHDTRKSIVQSVSSVGSAITVSGLTVVGGFTAIMFSNFPILRQFGLITVLDTAYALISALTILPAFIYLLRDRKEEKKNQRNEDTDKIE

>tr|A0A0S2W5M0|A0A0S2W5M0_9FIRM Putative efflux protein OS=Intestinimonas butyriciproducens OX=1297617 GN=IB211_02262c PE=4 SV=1

MRHIRLAFYSAAHFWVDLSCALLLLGVVCPEADLVRCILLYNFSAFAVQMPIGLLADRLNRNHQVAAAGCGLVAAAWFLTKGAEAAVLAGVGNALFHVGGGLDTMNRSGEKAGPLGVFVSPGAAGLFLGAAARGAAEALALPVCGVLVAAAVLILLWCRGPENPPAALPEGRGKWLPALLCLLLVVVLRSWNGFLFRFPWKGALEAPLTCAVVLGKTAGGLAADRFGTSRTAAITLGLSALCFLGSDWAPIGLLGVFLFQMTMPLTLWAAGRLLPSAKGLAFGVLTFGLFLGALPTHLGWEPIMPGPEVYAATALLSLPLLCYGLCTAQKRR

>tr|A0A0S4PTX6|A0A0S4PTX6_9HELI Magnesium and cobalt efflux protein CorC OS=Helicobacter typhlonius OX=76936 GN=BN2458_PEG0883 PE=4 SV=1

MGLLITYFLFAICISFVCSVLEAVLFSVTPPFMESYPKIHPKGGRILRYLKANIDNAIGAILIVNLFANTVGAAGVGAQAVKIFGETWQGVVAFVMTISILYISEIVPKTIGATYWKSLILPASYAIMVLYMITLPLVYVSRIITHLFRNNATNQMSRDEVLAVMELGEKSGSINELEGDILESLIEQKSLSVQDIMTPKERIFALNEEMSIKEANKAIRDHKYSRIPLYRGAEGNICSLIYRKKILQAMLDKKKKKPLKHFASEIAFVDMNLTLFDLLKLFINKKEHLFVVIDKRKNLVGIVSLDDVISATLGVVYVPEQQSETQGVAENVPLDSKNVETSIENFEIMTKDADYTETNDIGAMENTEIELDSKDIKFSLAQKD

>tr|A0A0S7BEL4|A0A0S7BEL4_9CHLR Arabinose efflux permease OS=Longilinea arvoryzae OX=360412 GN=LARV_00594 PE=4 SV=1

MHLPPSLRHRKFVLLWAGLFISIAGSQMQYWSLLWHIRELTDQPIAVSGIGLARFIPILAFALIGGLFADRYDRRKIMLITQATMALVALALGLLTLSGQIRLGWIYLLTAVQAVAISFDLPARQSLISNLVPRDDLASAFSMSSIAADLGAIIGPGLSGLTIAALGLFSVYMINAVSFLAVIVALLMIGTVPQQAVKRAENNHFRGRVDAGLFDIREGWRFILHQPVIMGSMILDFFATFFSSANTLLPFITRDVLHASVQQYGWLSAGQSIGAVSAALVISQRSRMRRQGSLLLGAVVIFGLATVFFGLSHSFWLTLVALILIGAGDSVSTILRNTIRQLQTPDELRGRMVSINQIFFQGGPQLGEVESGIVAQAFGPAAAIVSGGVGCVLAVGLVGGRWPQLRAYDGVEEAANA

>tr|A0A0T7CXI6|A0A0T7CXI6_9VIBR Putative threonine efflux protein OS=Vibrio crassostreae OX=246167 GN=VCR19J5_1210175 PE=4 SV=1

MTKNKHLESDYIFNSALETGIRAVCILSVNLSNKFDIHQLLAFDHLVVHTGDIINAPPSLHPANLQRNGELLVRRPLIEDGLALMVHKKLIKKEFTRNGFYYRATELACVFIESLTNRYIEEMSERAKWAIYMYQDSGDKLFSEVFNNAFERWTKEFHLVEKSIEQNWN

>tr|A0A0T9LB39|A0A0T9LB39_YERKR Cysteine/O-acetylserine efflux protein OS=Yersinia kristensenii OX=28152 GN=eamB_2 PE=4 SV=1

MLPGWLSKLRKVTRHRRLQKVYQSVSGYNFIILYGITALSTFVLPYTKDALWVISVSVLLAIIGTVGNVCWAAAGHLFQAIFRHYGRALNIVLSGLLFWVAIDMLI

>tr|A0A0U0K3N4|A0A0U0K3N4_STREE Cation efflux system protein OS=Streptococcus pneumoniae OX=1313 GN=czcD_2 PE=4 SV=1

MKAKYAVWVAFFLNLTYAIVEFIAGGVFGSSAVLADSVHDLGDVIAIGISAFLETISNREEDNQYTLGYKRFSLLGALVTAVILVTGSVLVILENVTKILHPQPVNDEGILWLGIIAITINLLASLVVGKGKTKNESIXLISRPSGTASKKILSVDQKRERAFERIKKEMTRDKRGSRIYQSVNRRTIAIRITATHPSVSSRKCRLRINSTMA

>tr|A0A0U1KPQ2|A0A0U1KPQ2_9BACI Glutathione-regulated potassium-efflux system protein KefC OS=Paraliobacillus sp. PM-2 OX=1462524 GN=kefC PE=4 SV=1

MEAHPESVSSLVIVISAAFITPILMHRLRLNVIPVVVAEIIVGLIIGQSGFNIVEESNWLETLSTLGFIFLMFLSGLEIDFSLFTRKKKKRSNDYDTGPNPVLIAILVFSGIILLSFGMSYLFVLAGFIDNVFLMTLIISTISLGVVVPTLKEAHAMKSPVGQTILLIAVIADLVTMILLAVFVSIYGDGSGNMWLLLILFAVGIVLYFVGRHFKNQSFLEAMSKGTIQIGTRAIFTLIIFLVALSESVGAENILGAFIAGALVSLLAPNQDLVQKLDSFGYGFLIPIFFVMIGVDINLWSLFGDPMVLMLIPLLLIALLLSKLIPVAILKKWYDTRTVVGAGMILTSTLSLVIAAAAIGERIGVIDSQMESALILVAVLTCIITPPFFKKLYIHEEEAIPKQIVSFIGSNQATLPVVRELDQELFESHLYHTRLDKIDEKITRSVFDIRELESYQINELQELGVFDSDIVVVSTGNEEINKDIACFAKHNGVERVIARAEISDIDKQLKENGVEVFSFLLSTKTLLKAMIESPNVVDILTNQESALYEIQMNNSVYDGIMLREFPFTGDVIMVRIFREDDSIVPHGDTRLRLGDNLVVTGSTEYVEELQGLLEFS

>tr|A0A0U3EEF9|A0A0U3EEF9_EUBLI Chromate efflux transporter OS=Eubacterium limosum OX=1736 GN=ACH52_0015 PE=4 SV=1

MDAKKLFKLFISTFSLSMFTFGGGYVIVPLMRKKFVKELGWIEEQEMLDLTAIAQSSPGAMAVNASILVGYRVSGVLGAFVAIVGTVLPPLIILSVISLFYTAFRDNLYVGFLLKAMQAGVSAVIVDVVIDMGGDIFKARKALPVVMMFVVFILSAFVKMNVIILILICGLIGALVTFTAKRTGKNLL

>tr|A0A0W0V7D1|A0A0W0V7D1_9GAMM Cation efflux transporter OS=Legionella israelensis OX=454 GN=Lisr_2182 PE=4 SV=1

MKAWHALSVEETREELAKDTQKYGPNELEEIETTRWYTMLARQFTNILILVLILATVLSFFIGDVVDALAILAIIIFNGLLGFVQEWKAETAIKNLKKILSPKCYIIQDDEKKEVDVKDLKPGDCVFLEAGNVVPADIRLSKSINLMINEASLTGESTSISKQTEAVSEQTPLANRKNMAYMGTHVVNGHGRGFVVAIGMDTEFGRIAELTGEIEESKTQLQKQLSVLGRQFGILALAASAVITLLGIMANRDILQMLMTGISLAVSAIPEGLPAVVTIALALGVRAMAKKKALMRRLQAAEALGVVSIICTDKTGTLTKNEMKVEKIWLPDRTIEITGVGYELEGDFKENKKTIDPSSQSDLMALLNTGLKCNHAKINKEKDRFKVEGSPDEAALVAAAVKSGLNQEHKSNITSEFTFDSNRKRMSVIEESKDERVVHVKGAPEVILKLSSHVLIADKKEKLNEKLQKKIEKAYIDFAEQGLRTLALARKTLSKDEVIDIDKAETGLTFLGIAGLLDPPRKAVPDALKKAKAAGIKIIMITGDSPLTAKAIAGQIGLKIEKTLTSSDLQDLSDEQLASLLKKEVLFARTIPKDKFRIVKLLQAQGDLVAMTGDGVNDTPALKQADIGIAMGIRGTDVARSVADIVLSDDNFASIIAAVEEGRRQYDNIRKFVLFLTSSNIGEMLAILINMIAGGPLILIPIQILWINLVTDSASAVSLSIEQAEKDIMERKPRKPEQPIITRLSFFLLGLFGSYIGIMTFILYQFYLNQSQALANTVAFTALVFMSNLHALNFRNLQNPIADIGWFSNKWLLIAILVMLSLQVLAIYLPWLQMILHTVPLSLFEWPVIILAALPLFLIPEFYKWLRKKDDTPTA

>tr|A0A0W0YSD8|A0A0W0YSD8_9GAMM p-hydroxybenzoic acid efflux pump subunit AaeB OS=Legionella sainthelensi OX=28087 GN=aaeB PE=4 SV=1

MSSLTIENRAALRTALAAVTSLIIAFICHLDKPYWSGMTVVLVANLYTGDILVKALMRILGTVIGALIGYFLSAFVVDSLLLYFCLNFFIVAIAVYYYNCSKYAYAWLLGAIAAFIVISGVAITPEDVLSSTIWRPIEIALGVIVSSIFAFFVLPNRITDKTVHDVNLIFATVDALFVNLDRLLMEQDRSIIQDIKDQNSQLKNRARQSLQTIVLIRYEFELDETQLNQYRFLLDTCLIFCRALNYFLSLNTFAAISTLPIKETISAIRFDISNLQANFFNISPPTTLLTESALNKFDQQYSWAVWATPNDFKSAKAWRHFFYQTNRMLIGIQVSLQGSYKHSNLDCINTSQQLKHDYNVITHSIKAGLTTMLALGLWLFMILPGGLNGIISSIVISARRNLYDMQNTGTMRLLGCLLGGGTGLYFIMTFTMSLYLLLVIIFFTVWAFSLFSFRHINYAYLGMQANVAIILVMAEHGASVMTVLPAIERMAGIVIGIAASFVVANLICRMSLTDMICRYLSKLQLNLAHNVACLLAENDKKTKYLDLIDTFWTCRELLSNATQKYALVHTPRTQWTPRQWQIDRAIEMERLLNSIQVTLNNVRESMDRKRAIKSASTYGIDLSVLERKILELYCSKPSCSMLVPDHLYAQQALEILMGVKGLSEPNHVELENCAAYVLALEQLEVNGRKMLLKIQEK

>tr|A0A100HWZ6|A0A100HWZ6_9RALS Transmembrane efflux transmembrane protein OS=Ralstonia sp. NT80 OX=1218247 GN=RSp1417 PE=4 SV=1

MKKWIVLFSIGIYLLLINLDLTIVNLALAEFSKDFNASIEQIQLVIVSYLAAAAAFFCLSGVLADRYGKKRVFMAGGLLFVLSSLYIGAFAHSIEAIIVARFVQGIGFSATLGLALILIGKAFPPEQKGLATGVAVTITGIGLAAGPPLGGFILQAFGWEMIFLINVPLGLLSLLLTAVFVDRDDANELSARKLDVVGVPLYLLGLGCLIVLSNALATLSWAQIGALTAVGAISTVLFVRRSLHVEAPLINLSLLRNGTYLTVVGIRIIFNFVMASFLFVLPLFMQNILNYSKVRAGLWVLCMTACIFVVGPITGKVIDRHGYKVPVLTGMTLLLVSCAAFLTLRVELSIVLFVIGLVSFGLSNGALTTATINGATSQVSPKHTGTAIGLFFTLSMVGAMFGVAVSGLILSKVGEFELARHIASTAATFTPEQLHTLHGLVNASQNLVNVAGTFADHPIDTVRDLVNTSYVPAMRALMAFNALLAAIGIGLSVALFKRKEARPADSTDPTKSRNEHLEGAV

>tr|A0A101F6V1|A0A101F6V1_9FIRM Auxin Efflux Carrier OS=Thermoanaerobacterales bacterium 50_218 OX=1635288 GN=XD63_1202 PE=4 SV=1

MVVLQTVFPVFVMIAIGYILGKITEIDIKPIAFLSIYVLAPALFFSSLVKTNLTAAEFFQIVGFIILLSSGTILLVKLWGKIEGWDPKTVKSVLLATLFPNCGYFGLPVLLFAFGEAGFERGIIYCVFMNLLHNTLGVYLAAQTHLSPRESLINVLKMPGLWAMSLGLGLTSLELTPPEMILKPLEMMGEAVIPVMLVTLGVHLARVRVGANIWLAGKVTTLRLVVAPLIGLLILYLFFDPTSLTSKVALVESACPVAVASTMFSIQFNARPELVSTAALTSTIASIFTFALILHFLV

>tr|A0A101I8T7|A0A101I8T7_9BACT ABC-type Na+ efflux pump, permease component OS=Thermotogales bacterium 46_20 OX=1635293 GN=XE05_1771 PE=4 SV=1

MLKDSFVIYRKELKNIMKDRRAIFSVFILPMIILPIIFLTIGFVTSAQQRTDAETLYVVNIIGDEDGRFTEVLQNFLVFERDEARVPDYTRAMESENYIIVELEGTDSDLTQVGNTLDARIYYRSTSRRSNFAAQQVRNALNQYSSMIMSERLRTLGLSLGDLNPVKTEMQDLAPEEARGTEFLAIMLPYFVLIFIFAGSMNIGLDTTAGEKERGSLAPVLVNQVSRTSIALGKVFYVMTVAVLNSLFTFVGLMVAFILGGSEAFGGEIPVNLAGFGIGSLIALLLVLLVLAGFAASLIILIGSFARNMKEGAGYVMPFYLLAIFAGIATMNMESVHQIHFYALPLVNSVFVMKDILTLQFFWSRFVVMLLSNLLYTSLLVLGVARLFNSEKVLNTGS

>tr|A0A124BR66|A0A124BR66_9DEIO Auxin efflux carrier family protein OS=Deinococcus grandis OX=57498 GN=DEIGR_100262 PE=4 SV=1

MIQALSNVLLPVMLVAGLGALVSARFPIDQATIARVTLYLLSPALALNVLLTTRVQAGEVLTLGAAYALTVAGSLLLGWLTGLRAPQAQRRSLTASVGIWNSGNMGLPIALFAFGQAGFAHATLLFLMSFVGMYVIAPVVYTARVRRPDQPPPSAGGMLLNMVRLPAVWMVALGVTLRALHLQPPEGLMRGVELLAQATLPMVLLSLGLQLGSGGWPRLDARVWLATAARLIGGPLLGLAAGLACGLRGEVLAVLVLSASMPTAVNALLIAREYGGDADTVARTAFLSTVLSVPTIAAVVALLPRLTG

>tr|A0A132BZ71|A0A132BZ71_9RHOB Cation efflux family protein OS=Tritonibacter horizontis OX=1768241 GN=TRIHO_15060 PE=4 SV=1

MSASKWRDTDDKVTNSRRELIEYIRGYRIMSKQKNDRQTNLTRGIRVEIASLVYNLIEVVVSVTVGLLTGSAALVSWGFDSTVEATSAGTLIWRLKAEKDGGDKRTVLHRNKVALYVVACAFWIVVAAILYEAVSAFISQEAPGFNWWGIAILFVSLVVNPLLAWGKYRYGKRLDSPALKYDAKDTMICEYQTIVVLAGIGLTQWMGWWWADPVAALLIVPYVAWEAFEATKDARSVGPGEAEATADA

>tr|A0A133SJG7|A0A133SJG7_9FIRM Transporter, auxin efflux carrier family protein OS=Megasphaera sp. MJR8396C OX=1603888 GN=HMPREF3201_00634 PE=4 SV=1

MEITIIKAAILVSFILCGYILKQLRLFGRSTFQTISTIVFNITLPAVIIANLNGIHFEVRYLFISLLAIVFNLLMVGLGYGVGRTKDEKAFYMLNLNGYNIGNFALPFVSYFFDSAAVLIVCLFDAGNSLMCLGGGYGLARYVRGEKGDNIFYILAKTIFSSLPVLSYMLMIILALGGLALPQVVIDWVKVPASANTFLSMLMIGVALGLSLKKEYLHLIYSDIGLRLLISAVFAVFVYVGLDYSMDIKRVLMVLVFAPIAGMACYYTARLKLKIEVAACISSLYILISIVVMSTLIVILETI

>tr|A0A133Z478|A0A133Z478_9CORY AEC family transporter OS=Corynebacterium kroppenstedtii OX=161879 GN=CJ202_00285 PE=4 SV=1

MPQVLSGFATIAIVIAIGFFVGKANLLGKHAQFTLQMYVYFLATPALLLDKLYVTDPLDVLGPQLAVASGSALTIGLIYFLWARTALHRPIHESAVGGLASSYCNASNLGIPVAAHVVGDSTVVVPTLLFQIAFYGPIVLAILDVVTAKEAHADGRAGRVNTRSLFITPFKNPMLLGALTGLVISILHAHAGVSVPHPLVEPVHLIGQSAVPVALIAFGMSLAGQKVLDPSTSPRLEVGVASATKILGQPLAAFLIAHFVFGMTGHALFAACVVATLPTAQNVYTYATRYGRGLTLARDAGVITTAASFVMIMLLALVFT

>tr|A0A136KTM2|A0A136KTM2_9BACT Arabinose efflux permease OS=Armatimonadetes bacterium OLB18 OX=1617424 GN=UZ18_ATM001002104 PE=4 SV=1

MSRSEWPLLAAVFLEMTGFGMAFPDIQLRAEVFGAPGQIIGAVLASYFVVQLLVSPAWGRLSDRVGRKPVLLVCTALSAGSMVIYALAHTVETILLSRVVAGLAAANVVAAQAYIADVNRGVQLERSMGRMSAAMLAGLVAGPAIGGFLATVGGNQLMGFAAAGCSLASLVWILFGVRSVPVVASATSEAPRLRRRSWALLQDTPGLMRFVAIAVAGWFVLACLEGTYGRLIKHNLGMGQFEFGLIFSYESLLGAAVGWTLGWLATRLGSSWMLKGGYLLQAIGLLAMPFAPGFGVLLLASTFYAFGIGVTNPTINSVCSKMTSNERQGELFGVLQAARSVGFVAGPILGGALFDVLPGLPYYVAAGVAGVAALMVVVPQDGTVPESAPVDAA

>tr|A0A137SMF1|A0A137SMF1_9GAMM Potassium efflux system KefA protein / Small-conductance mechanosensitive channel OS=Moritella sp. JT01 OX=756698 GN=AKG98_3020 PE=4 SV=1

MMTKHYVVLLILFSLFMTPVYAELSYGEAQLNNTLSQLEELESTPSVKLQIKYYQQALKDLTEDTNARQTAVRYQKIIDDYPVTSQTLKAKITDYISAKFADPSDWSLNKVEQAIAKQNSNLTDLKQQQQARSSELTTIGIRISSFQTDIERLRRKLTHTQKESDKLISAGYSSLNSEQEAIRISLQIKESSLSTQIQMLELEQLSASNRSELAQLNRRLIQREQKDVSKNLTTLTDLRNSILRKETEDAIARSKQINDSSLISSPFLQLQLEINQELSKELATVSAKSEIIQRKQQAVTQQVDALTTTLTNFNEQVEWLKISSAFGENLRAQVSSLPSEPPLAKLENEIVESRLARFRYKKMLTQLDSLPLATKTLTPVEHESLLRFIELRRLLLSQLISSLDNHIYEQTKLKVSYSKMNSTLVQIKQQADEHLFWVPSSPFVNTQTISELLASMLWITSIDNSITIPQAILSVPLATLSLALLFILGLTYLHAPLNKYFTKHIAEIYPKVGKVTKDKFSYTLRNLAYSFADALLLPLSLFIITELLISAWEFPFAVNIGHALQDSLFLLVIYLFMRNLTRYKGLLQIHLKIDKKLIAKIWGYYQVLFFIYWPSYIIQVLCNQYPEQAYDGSLGRLAFIIACCALTQFYYRLYREKLPLTYKKKNNDKPHIVHHSIWTVFIMAPIASAIIALMGYLYTAQVLLKQMESSLLMGVFFLLTYYLIRRGMHLQKRRLAFERAKAKRIDIIAQRMKEVEKGEQNTSQESHFDIEEPEIDLDQISAQSLGLLRTLLTLLFVALNALFWSEIQSAFTFLDTITLWDAANTLNGVEYIDPITLKSGLLAITIFALTLVLVRNLSGALELLILQHLDLSPGTGFAITTLAKYMTISIGFVVGFNFLGVDWAKTQWLVAALTVGLGFGLQEIFANFVSGLIILFEKPIRIGDTVTIRELTGSISKIQTRATTIVDWDRKEIIVPNKAFITEQFINWSLSDSITRVIINIGVEFNSDIELVTKLLLDCAEENSLSLENPGPEVFFIEFGQHSLCFEVRCYVAEMGHRLTMTHALNIRINQVFKEHHIRIALQQLDLNVKHGIKVSDSGHVMSMKKGSLR

>tr|A0A139T2R5|A0A139T2R5_9FIRM Transporter, auxin efflux carrier family protein OS=Candidatus Stoquefichus sp. KLE1796 OX=1574263 GN=HMPREF3037_02910 PE=4 SV=1

MNKIRDVKVKEEKFMTALSTLFPVLFMVLLGLISRIKGFITPEQKEGANTIVFNVLFPILIFNILLTSKIESSAIFIVVYVFIAFSLAMVIGKLLGKFTGQHFSHISHFMLTTCEGGNVALPLYTSIVGVAYASNTVIFDLAGTLIAFVVIPILVAQKSAGETTFKELMKTIFTNSFVIAVMLGLILNLTGVYDMLSQSALIDVYTNTVSTATAPIMGMILFVIGYNLKINLATIGSILKLLVVRVIFYIVVIAGFFILFPHLMAEKTYLMAVLIYFMCPTGFALPMQISPLYKSDEDANFTSAFISLNMIITLIVYAVVVIFIA

>tr|A0A142YFN9|A0A142YFN9_9PLAN Magnesium and cobalt efflux protein CorC OS=Planctomyces sp. SH-PL62 OX=1636152 GN=corC_3 PE=4 SV=1

MDGSPRTDLDCRMISIVVLVLGILALLLAASLFSLLEHASEAAQIQHLREQAAKGDRGAKAALRRAGDPESAATAARLGAIFALILAGALAGAAASDGRGGLDWLPGLAAVLGIAAAATILADVAPRVLAASRPEWFAARLARTVGPATAAIEPLARRLRRAGGFLAARLGARNAERASGVEQRIKDLMNAGAESGGFDPSKHAIFKRVFRFCDRRARALMTPRDQVVWLDVRDTPEEIARKIVLSPHASLPVCDETLDNLLGMVQMKSLLARGAEGQPTRFKGLLTLPDFIYEGTRGPQILDVLRKAATGAAVVLDEYGSVVGVITLADVRDALLGTMIEKPEEESPRAVQRPDGSWLLDGRFPIDEFVDLFQIPRPAQGEFDTLGGLVVTKLGRIPRVGEGFQDLGLRFEVVDMDANRVDHVLVRPLDLAR

>tr|A0A143WJC1|A0A143WJC1_KOMXY p-hydroxybenzoic acid efflux pump subunit AaeB OS=Komagataeibacter xylinus OX=28448 GN=aaeB_4 PE=4 SV=1

MTVHVPFLYVPVHRAPPISRSPLPHPAWGRALVKYLSRLYDIFRNDGQWRSFMATAAFSARVLVSMSIALFLAFSFQLQSPMSSVTTVMIVANPTVGALVSKSIWRMIGTVLGAIISVALMATFVQSPILYIMGLSVTVGLACMAATFLRLFRAYAAVLTGYTIVIIAAPAFGDPDGIFLSALSRLSAVVVGIVTTAAVFLVTSPRRSDPLLEQIRTVFCDTIRYVLSRHDGGASTTTDGAFHAQRAGMLARIASLSDAVEYAATDNYDISVRHREIRAGLARLSGIVASYHPHAVIALGAVPVSASQDAPPADGRIAELMRTLLQTMENVPLSEGCPPACRHIANTREALLEQAEHSPSPRQLLFLDDARDLLSRLEHALCDLAHRGGNDRSLRLRPYMEWPTALRNGARGALTTLLAGLAWYVLHWTGGPMMMLYVVAASSLLSTAPSASRASGLLASGTALGVPAGLLCHLFILPRIDGYPLLCLSLGLFLLPGIWLQFNPRLGIAAFGYSVFSTIMLQVNNPIHYNDIPLMNEWVAILMGCCMLVLSFRVILPPNHRLDGARLVASLSRSVRSLALARASFQGQWIVWEHLQLQKVARLAMRLSFCAPAEVTNLYVDAALAAISLGRLVERLHRLADRADISLPERQQLLAALGAFETLTRDPLATARTLHNICTRSGAGQALTTLSPRRMEALACMEQAEQIIVDIPAFLDRNGPIQWSDDYPRAREFLRAAYSGGAMSG

>tr|A0A143YSR6|A0A143YSR6_9LACT Auxin efflux carrier OS=Trichococcus flocculiformis OX=82803 GN=TFLO_2047 PE=4 SV=1

MISILLVRKIAQLFLIMILGYLLVKLRILKTEESIVLSKLSLYLVMPAVILSAFQVDFKPEIQAGLMLAFVAAVAIHILLLIIGHISGNLFHFEEIDIASIIYSNAGNLIIPIVTAVLGTEWVIYSTAFLSVQLIFLWTHCKLMFSKEKKPHFRKIILNVNMIAIFIGVLSMLWGSRLPAIILLLLKLSNAASLVNNGTEILLITFLATVTPAASTVTQFAQVHDKNAAYAGAINIMTTLLCIVTMPIFVILYYI

>tr|A0A160P4M2|A0A160P4M2_STRLU Transmembrane efflux protein OS=Streptomyces laurentii OX=39478 GN=SLA_4646 PE=4 SV=1

MGVQAGLARLQLDEVEDLGLAAEDQVVEAQQHPRARTDGGGGPGDLGGTGSGICLGDVLGRGLGQVRQLVPGEGGVVGGTAGADDPRVSRATSSGVTTSAASRAPAGAGAAGLVPAARSAPEGVVAAVEVPGSA

>tr|A0A171EY59|A0A171EY59_ACIBA Homoserine/Threonine efflux protein OS=Acinetobacter baumannii Naval-82 OX=903921 GN=ACINNAV82_1605 PE=4 SV=1

MWQLYGHEFLTLALIHFMAVILPGPDFVITVRQSVRYGYLIGCLTAIGIGVGISVHVFYTLVGIGFLIQQSEWLMSLIRTAGAAYLVYLGWQCLRSQPNPNIEINGQTDSDTPSLLKAFTMGFLTNALNPKATIFFLAIFTTIVSTTTPMKVQVFYGVWMCMVNAIWFMVVSVLFAQPIVRKRFLEFGVYFERVMGVLLIGIALRLIWSLFV

>tr|A0A174BMS8|A0A174BMS8_9FIRM Probable amino-acid metabolite efflux pump OS=Catenibacterium mitsuokai OX=100886 GN=eamA PE=4 SV=1

MVGFIVLKKESTYICATCRKCMNAMKNYTYHLVAVLTVGIWGLTFISTKVLIGHGLSPQEIFLLRFLIAYMGIWLISPRKLFADNWKDEFWMFLGGMTGGSFYFFTENTALEITLATNVSFIVCTAPLLTTILSLWVYKKEKATRGLMAGSLLALVGVALVVYNGSFVLKISPLGDFLTLLAAFSWAFYSLIMRKMSNCYGITFITRKIFFYGVLTILPAFLIHPWNFDIARLLEPAILFNLLFLGVLASLICFVVWNVILKQLGTIRASNYIYLNPLFTLIGSAFLLGERLTMVALMGAVLILGGVYWAGKR

>tr|A0A174MNE8|A0A174MNE8_9ACTN Magnesium/cobalt efflux protein CorC OS=Collinsella aerofaciens OX=74426 GN=ERS852514_01841 PE=4 SV=1

MDITISLITTLVLTLINGYFSMSEMALTTAKRAVLEHEAEEGDKRAERAIKLAADSDQLLATIQVAITLVGFASSAVASTSLSDPLATWLMSFGIAPLSAIARGLAPVIITVAVAFVSIVIGELVPKRIGLSNAEGVSKQVVGTLSFFQKIARPLVWLTGACSDGLARILRIKSADDRQNVSEEEIKYMVSEQDDLLDEEKRMIHEIFDLGDTVAREVMVPRVDTTMCEDDETVADVLSTMRQTGFSRIPVYHEDPDNVAGIAHIKDLIQPALDGKGDQPIAGFLRDATFVPDTKDILPLLSEMQTSHDQIVVVVDEYGGTAGIITIEDIVEEIVGEIEDEFDPDNKYLTRLSRREWLVDGRFSCDDAIELGWPLEESDDYETIAGWILELCDSVPDIGEVFEVAGYKFKVQSMRGQRISLIRVIAPAETDKKDSESSVDEPTTSGAGSANPHDGDE

>tr|A0A180F7C7|A0A180F7C7_9BACT Macrolide-specific efflux protein macA OS=Bacteroidales bacterium Barb4 OX=1633200 GN=macA PE=4 SV=1

MINDSTPNKVSNLVRGKQFIIKNQMTRKSCFIGIMGIMGIVAAGLVSCGNGKDGFDATGTFEATEILVSSEASGKIMALDIKEGERLEAGAFAGYIDTTQLYLKKRQLSAGLRSVDIRKPDIRKQIAALEQQIATAKTEQQRMENLVQAKAGNQKQLDDITNNIKLLQRQLDAQYSTLDKTAGASDAEAESLQYQIMQLDDLMEKSRIVNPRTGTVLVKYAEQGEVTAPGKPLYKIADTELLYLRAYITAGQLSRLKQGQEVKVYADFGSDSREYAGTVAWVSDKSEFTPKGIQTKDERANMVYAIKAAVRNDGYLKIGQYGEIVFEP

>tr|A0A1B1FV78|A0A1B1FV78_9BACT Cation efflux system protein CzcC OS=Flammeovirga sp. MY04 OX=1191459 GN=MY04_2659 PE=4 SV=1

MSLTLPQGEEIFLEKNLTLVAERHNIDIAKAEIIQAKAWPNPELGVEIAMYDNEDNKWFRTDSEAQRVVEIHQLIEMGGKRKKRTNIAQKEAEIAEYEFYTTMRELRTELRSLMVELHYLQEKSNSYLNVIEPLERLIEVYKEQSDKGNIAKSEVVRLKALLLDARKGWLDIEQEATDVSSQLKLILNLQPQVDLTITLPTFNYSQNVVDPELWVSEAQEHRMDFKIEQLRLQQVAESLALEKAENVPDIELGTMYDRRGAHQADYWALQIAFDLPVWNRNKGGIQAAKIAQEQQQVKVTQAENQLQIDVYNAAQKLNQITKVYDALDPELSEEMRAVMESVTKSYQKQEISLIEFIDFFESYKENLGQLFDTEYALFSALELINYTVGKDIYPIQ

>tr|A0A1B2FNM4|A0A1B2FNM4_9ACTN Na/H efflux pump OS=Streptomyces sp. CB02366 OX=1703935 GN=AMK24_28920 PE=4 SV=1

MHGPDVAALAAATSPLGGEPLTVFLLQVGVLLVCAYGLGRLGARVGLPPLVGELTAGVLLGPTLLGQIAPGLSGRLFPADMSQAHLLDAFCQFGILLLVAIAGAQFDPLILRRRGGLAARVSLAGLLVPLGLGIATGYLVPASLLTDSGERGVFALFLGVAMCVTALPVIAKTLADLNLTHRNVGQLLIAAAVFDDAVGWLLLALVTALASGGAGGPVVLTTMAWTAVFIAAACAVGGPIGRRMSRTGGGRVPVSAITVGVAVVVLYGALTAAAGMEALFGAFVAGATLLRHIDPARLAPLRTLVMAVFAPVFLGSVGLRMDLTALAEPSVLLTGVVVLFVATLGKFAGAYVAARSGGMSRYEGLALGAGMNSRGMIEVVIALVGLRIGVLDTVTFTIIVLIALITSVSAPPLLRWASSRIAFESDEQEREERLAGWSTEPAFSGGPPPKSARQEKTADTS

>tr|A0A1C0U3B1|A0A1C0U3B1_9GAMM Leucine efflux protein OS=Photorhabdus australis OX=286156 GN=leuE PE=4 SV=1

MNTVTLLTYLITCVVSAATPGPGTMSVIAYSAFLGWRKTLPVIFGIQVGMLAMALLAFSGVTAALSASPLLFNLLQYIGALYIAYLGVLSLKYARKGIGTDGAAYDKGAFRNFNHGALVTFASPKTLLFFTSIFPIFLDASRSVLPQMVFLLTLLLGCTFFVHIIYAFCMKYFSRLLKEHSVIFNITVGIIFLGLALYMALQVELYVI

>tr|A0A1C3GW61|A0A1C3GW61_9PSED Putative amino-acid metabolite efflux pump OS=Pseudomonas sp. 1 R 17 OX=1844091 GN=eamA_4 PE=4 SV=1

MNPIADKSYEVRLTSPLPIVFILLWSSGYIGGAYGVRYGEPFTMTFYRFALAALVFLGVALAIKAQWPKRLAPYFHAATVGLLLQALQFGGLYAGISQGVPAGQAALIVGLMPVFVVIGAYFCLGEQLSWRDLPGSILGVGGVAIVVASSFFGSEASVGGYGAVGLALLGITLGTLYQKRFLGGVNLWVGCFIQMVTASLVMLLLAYTTETMQVTEWVPFVASVAWITLMNSVGALTLLYLMIRRGEASKATNLFHVIPAVTQIMASLVLGEVPSGVAILGFVVSGAGVYWMNHVRAK

>tr|A0A1C4GVH3|A0A1C4GVH3_9GAMM Regulator RcnB of Ni and Co efflux OS=Acinetobacter albensis OX=1673609 GN=GA0116959_10817 PE=4 SV=1

MKKILTILAISFSALMASSVSTAAPHDKQDRSRGWDHPRHQESNKNRDFREDDDDERMQDKRRGREERGVKRLQQHKWQTGYVMPQHYRGNGYKVDYKNLDLPKPSRNQQWYKINNDYILVDSDSHNIVQIKGF

>tr|A0A1C5RPS5|A0A1C5RPS5_9FIRM Magnesium/cobalt efflux protein CorC OS=uncultured Blautia sp. OX=765821 GN=ytfL PE=4 SV=1

MESGSSMPLAGFVILLLLLWLNGIFYGFSAAVHNLSENEVEKRAQEGDKKAVFLLSLINNPVSFVNAIPLIVMASGVCFGAFIVPWATETFHPYIKHLAALILVLALVIILLASLGILTFRRIGTYHPEKYAYRYMKIVGFFTRILYPFTMCVTFIAKLAARPFGVAFNQSEDPVTEEEIISIVDEAHEQGVIEENEAEMIQNIMEFTDTEAKDIMTHRKNVIAFDEEDNLQTIVDTMLEEGNSRYPVFRETIDNIVGIIHYKDALKFLTRNSWAKFKPLKDLPGLIREASFIPETRGISDLFKSMQMKKIHMAVVVDEYGQTSGIVSMEDILEEIVGDILDEYDEDDSTFRTQKDNSVIIDALAYLEDVAEELGIDFGKVEFETLNGYLTNLLGHIPTEDDLDKEIVVNGYRFRILSLGNKTIGKVRAEKIKKEPKGEDKKCQDIQNSQT

>tr|A0A1C5UCE9|A0A1C5UCE9_9ACTN Spectinomycin tetracycline efflux pump OS=uncultured Collinsella sp. OX=165190 GN=stp_4 PE=4 SV=1

MNHAENRQSVEVARSGRGTCAAQAPCAVQAPGAAQASHPAESPRSAQAQGGERAPRPAQASQSAPTPHAVPAPRPTAGKWVVLFTVVAMTFMSTLDSSIVNVALPAMQRELGVGASDIQWVSSIYLLACCVTVLVFGRLGDRYGKVRFFQVGVALFTAGSALCGLATTLPVLIGARVVQALGAASATANNMGIVTEVFPASQRGRALGITSTFVSLGLMCGPTIGGMLVAVYPWESIFLINVPVGIVAFLVGLKTLPRDAPRTEADRSARSGFDIAGSLLLAPAIFFTFFSLTNLANGATPLLMGLLAAGLALLVVFVLVERRVEAPLVRLDLFGNAVFSANLAAMLLCFLAVGATEYLLPFFLQDACGYESNVAGFILTAIPLGMAIMGPLGGALSDRIGSFWPCLVGLVIYAAGIWFVGGLSDDAGVVVIVLLMAAMAAGTGLFQSPNNALVMGSVEAEDLGFAGSLVSLVRYMGMSAGVTGGTVLLYGQMSSLAGHAVTGYVEGRPELFLAGFSFTFDVLAVLVLLGAVLLVVGAVLKRGR

>tr|A0A1C5WDA2|A0A1C5WDA2_9CLOT Potassium efflux system KefA OS=uncultured Clostridium sp. OX=59620 GN=kefA PE=4 SV=1

MIIGKIINSVIIVLISYILIKLIQYLLHRLFEFTNFDVRYENTLSSVLSSITYYIVFVICVILVLREFGIVDATKFGSLVTGASIVGLIAGFASQSILKDIFNGFFILFEKQLQVGDFVIINEEFRGTVEEIGIRSTSLRDWDLRRITLPNGSINSIKNYSKDKMRVVVHVKVSYEEDPNKVISSLQEVCEIMNNQYSDYLYKDINSNKNKGFCVYGVTDIDKSSIGAQYTITGMVKSYRYFSALKESKLQILIVFNKNNIKIAYPRHINIISHENKDMNSLD

>tr|A0A1C6A3R7|A0A1C6A3R7_9FIRM Arabinose efflux permease OS=uncultured Flavonifractor sp. OX=1193534 GN=SAMEA3545394_00572 PE=4 SV=1

MRIASHAPSRSAGLRAWVVCLGCGLALFTVMGLGVNAFTVYQPYLLRVHGFTNAQGSWITTVRSLFALLSILTVDRLCRRLGLRNTMVLGMACFVGSYLLFGFARGFSAYCGGAVLSGLAYGYGGMIPLTLVISRWFPTGRGFALGMAAAGSGISTIFAPPLITGAIQALGLSAAFLWEAAAGVLLTLLVLLLVRDSSDCPELQRAGDSAPGGRGEGLSRPLLGMVLLSAFLTGGPCGPGFSHLTVLYTSAGFSSGTAALLMSYLGLVLIAAKVLYGWLSDRLGSRMANRLIFGVFLAGFALCCLAYTQSLPLAAAAITLTGLGMPLSSVTLSVWAGDLSAPEDYDRLVKWLSSAYMLGSLVTGPVPGLLADRFGGSYVPAYGLFLFFLLISMLLIQTVYRRTGVGGRPQR

>tr|A0A1C6HNM7|A0A1C6HNM7_9CLOT Auxin efflux carrier OS=uncultured Clostridium sp. OX=59620 GN=SAMEA3545404_01813 PE=4 SV=1

MTDIGMILGQVAILFIIMLVGVIARKTKILSDAGLGAMSQLALFVTVPCMVLVSFQSEFSQQLLLDMGHAVAWSLGVHIVMWLLGKKIFNRFALHQRKPLQFAAIFSNAAFMGYPVLQAIFGETGLLLGSMYTAIFNIFLWTVGMSIFSGSEKEDRKAAIKRVLLNPGTIATVLGLVMFVFSIKLPDMPMQALSMLGNMTTPLSMLIVGARLADVRIKEAFAGAGVYLACALRLVVIPLILMGLMKLCRVPPLAMGVVTIQAAMPIAANTAMFAEMFGGDAPFASRLVFLSTLLSIITIPIFMLLVA

>tr|A0A1C6RZP3|A0A1C6RZP3_9ACTN Threonine/homoserine/homoserine lactone efflux protein OS=Micromonospora aurantiaca OX=47850 GN=GA0070615_0179 PE=4 SV=1

MSDIQIVSFVAASLLIIIVPGVDFALVTRQTVRYGRRAGFVVLAGLFAAALVHASLATAGLSALLVSSPALYTVLRVAGALYLLYLGGTILWATRPRRAAAPAAQPVTVGAGGPGPDADTGPVAAPETVAADEPHVARRSFVMGVTSQLLNVKVVVFYVSFVPQFVKPGDGAAARTAVLAATFIGLAVLWWACYILLIDRLQPWLTRPSVLLVIERLTGLILIVLAVRIALSH

>tr|A0A1D8AZ91|A0A1D8AZ91_9BACT Sugar efflux transporter A OS=Lacunisphaera limnophila OX=1838286 GN=setA PE=4 SV=1

MKALLAPWRRLLGHREFGIMALSNLVLGMAYSFVAPFYSMFGTLEVGMTNWVFGVFMTVTSLSGIVITTFLSRWSDTRISRRAILLLACVCGVAGYAGYAYVRDVIWLTVIGSLALGVSSITFAQLFAYQREFLTRHGVPDAEAPLYMNIFRLLFSLAWTIGPAIAAWVMIKYSYEGIFLTCAAMFGLLFVIVWRYIPARPPTAAAMANKVPLSQVLRRPYLLCYFAAFVLVFICVTMGMMNLPLLILQTLGGTAEQVGIAFSVAPVFELPLMFWFGLLASRSHPGRLIRIGMIIAVAYYALLFFVTQPWHIYPLQILSAAMIAVVSGIAITFFQSYIPDQPGTATNLYTTANRIGSTIGYLSFGSLAGSFGYRAIFLVCAVLCSAAFLLLWLSREKHEQAVAPA

>tr|A0A1E1F3G7|A0A1E1F3G7_9SPHN CDF-family cation efflux system protein OS=Sphingobium cloacae OX=120107 GN=SCLO_1020190 PE=4 SV=1

MTMHGDHEQGHGHHHHHGHHDGPERHAHGSGHGNGHGDGQDDGRGGRDRFPPPRADAEISHYFDHIYLSAGHDRNAKRTVWVVWLTAATMVVEIVFGWITGSMALLADGFHMATHAGALAVAAAAYGYARRHARNPRFTFGTGKVGDLSGFASALLLGVTALFIAVESGMRLFEPVDVRFGEATLVAVIGLAVNLLSALLLGHDHGHDGGHDGGHDGHDHGAGQDHKHSDNNLRAAYVHVLTDALTSVLAIVALMAGRYLDWWWMDPAVGLLGAVVIARWAWGLMKDTAAILLDTAEPALMARVRDLTEAEGAVIRDLHVWRVGPHAHAAIISIAPGADSAAVRARVSALPRMEHVTVETV

>tr|A0A1G5ZL72|A0A1G5ZL72_9RHIZ Threonine/homoserine efflux transporter RhtA OS=Sinorhizobium sp. NFACC03 OX=1566295 GN=SAMN03159448_05462 PE=4 SV=1

MEDDQNKWLGTLLIIGSAIAYSLSGYFTRLITLDVWTVLFWRGIFGGLFIGAYVVWRYRKDLWVAIRAMGMAGFWVMILSTVATICFINALRLAPVADVMTIHAAIPFMTATLALVFAGEREEWATWAASFTALVGVMIIVNPQASSDYLAGYAFATTMALSYAAMIVIIRKNRHASMLPAASLSAFLCAFVALPFAQPMQLAAPVMLDLVLFGTVQFGLGLLLMTIGTRLISATRSALIGSMENPLAPLWVWLAFGEFPAWATWAGGGLVMGAVIFDVLTKSKRRQKSVEALAQG

>tr|A0A1G6CIV7|A0A1G6CIV7_EUBOX Threonine/homoserine efflux transporter RhtA OS=Eubacterium oxidoreducens OX=1732 GN=SAMN02910417_02434 PE=4 SV=1

MVCFLLTIFFLSSIVKQKKIKGMVSKNMKKFAIFMPLLAGICWGLTGLFVRNLGDHGLSNVSILGGRTVFAVLILLIGILIYNKERLKIRHLKDLVFIILAGFVGSFALNVCYNYTISVMTMSLAAILLCLAPIFTLILAAIFFREKITTKKVLCMVFAIFGCVLASGIVGDNSSVNVTLLGVFIGLLSAFFYGVYSICSKVVTNDGYTSLTITFYSQVVILIACIPFTNWNSVLHYMASDPFPHSAILIIHALLSSAVPYLLLVSSFHHMDTGLATIIASGAEPVTAAVLGMFFYQEIPTVIIGIGLVITIVALCILLKPDAPPKEEPSQVPS

>tr|A0A1G6KNG8|A0A1G6KNG8_9ACTN Threonine/homoserine efflux transporter RhtA OS=Olsenella umbonata OX=604330 GN=SAMN04487824_10923 PE=4 SV=1

MGNASGAGIGAGWTQRRADLMIAVIACAWGSSYLMMQVGLSSIPPFGMVALRFGIAFVAVAIIFRKRLRELTASVVARGAVLGFLLCVFFGLLMYGLKTTPASTAGFLTSAKVIFVPLIVAVATHRAPSRATLAGIGICVAGLALLTLSGPVSLGGGAGLCLAGSAVYALQIVATDTFSRSDDALLLGICQLGFAAVFGAAFNLAFEGPVLPQSPAEWGAVLGLALVCSAFGFAMQPVAQSRTTATHAGLLFSLESVSSAVLSFVFLGEVMAPQCYLGCALILAAVLLSSLADGKADAAEGAADARAFSASTHGGLARGLATARVHAKRAQE

>tr|A0A1G6W2J5|A0A1G6W2J5_9GAMM Regulator RcnB of Ni and Co efflux OS=Aquimonas voraii OX=265719 GN=SAMN04488509_10486 PE=4 SV=1

MRTQLSGRFLSHATLLIGLALAAGSAHAERPDRGADYGGRGSDSGVERGDSDKGHGDGGWGERGGGRGEREQSWQAGSQDRRDSWQERGSSRSEGWRGESRGESSQMQRIAGWQGGHREPEHRGGREPQWQGDRWGQDRRDHWNSGTRRDDARHWQSDRDWREEHRGRGDWRRDDFRRNDWRHDDRHRGDWRGDWRHPEWRRHWQHGWGGHRYRAEVRYVYPRGYRAQSWRIGYRLPPVFLVNDWYVDWRYYRLAAPPWGCRWLRVDGDLLLVDERSGEIVDVLYGFFYY

>tr|A0A1G7EKG7|A0A1G7EKG7_9BURK Cu and Ag efflux protein CusF OS=Massilia sp. PDC64 OX=1881046 GN=SAMN05428966_109297 PE=4 SV=1

MIRFSSLFGTLFVSAGLALAAADASAQMQMQMPMHDHDQHSAMAATSQEDTNALSEGEIKKVDKDTGKLTIKHGPLTNLGMPGMTMAFKVQNPAMLDQVKVGDHVRFRVERINDTFTITKLEASI

>tr|A0A1G7Z4I9|A0A1G7Z4I9_CHIFI Outer membrane protein, cobalt-zinc-cadmium efflux system OS=Chitinophaga filiformis OX=104663 GN=SAMN04488121_108121 PE=4 SV=1

MHACAYGSLQRLTINLYVPVLLISHKLFNTNFIINDSNMSSKDLHVASIYRILTCCLLLLAAGISAKAQDTVHINLPDAEKQFLDRNLQLLAEKYNVSIAKAQIIQARLYNNPNLTLSGNLYNPDQKKFFDISNQTGQYEIGIQQMISLAGKRNKQVKLARTNAEMAENAFFDLLRTLRFTLRSDFYQAYYLQSSMKAYEAQIATLEKMDATYKELQQKGLVTLKDAVRLRSLLYSLRAERTNMQNQVNDLEAELQLLLQNNHSWFAPEVQDNALAGIPEVRQTSLQSLVDSAYANRQDLLLAQNSLLYNQQNYSLQKAMAVPDLTLGASFDKRGSFVNNASFLNLGIDLPFFNRNQGNIKAAKFSVDQNKLLVQQQTQVVENEVQTAYVKAMNTDKMLESVDPAFRGQFEQLLQSITDNFMKKNISLLELTDFYDSYKENILQLNQLQNDRMQAIETLNFAIGKTLFNK

>tr|A0A1G9P3W2|A0A1G9P3W2_9RHOB Threonine/homoserine/homoserine lactone efflux protein OS=Paracoccus chinensis OX=525640 GN=SAMN04487971_1442 PE=4 SV=1

MEAAALLTYMATLGALTLAPGPLVAVLVARSSSQDRAGACALAIGMCVGDVLVILAICAGLGFWLQAHPEIFTVGKYAGVGLLLWMAFRMWSASAAPAAHPAPTCGIISSALVGLALCLSSPQTVVMYLVLLPRVIDLTGVRAQETLMLIAATILALLGVFLLVIFCADLTQRLLRSSVGVVLWARGTALAVATSAAGVFFW

>tr|A0A1G9SGW9|A0A1G9SGW9_9RHOB O-acetylserine/cysteine efflux transporter OS=Maricaulis salignorans OX=144026 GN=SAMN04488568_10983 PE=4 SV=1

MRIRDLLALIAVCAVWGVNFVVAKFSITGSPGWVPGFEGSPPLFFAFLRFALLFIILSPWLMPRPGDMKAMFGIALTMGALQYALMFLGLQWATPSGMAITLQMGVPFATLLSVVMLKERLGLPRITGIVIAFAGIILVVARPGIGGLSFGLLLGIGAAFSGALGMILVKRMPLDSMRMQSWIGLISWPPLLVLSLVFERDQISSVMAGGWPFMLTVIFTVLLVNVFGHGVFYKMLQRYDATMIAPITLLAPLIGVISGIVITGDEAGWRLFVGGGLALLGVGIIALRPNRALPEAGLAREKTL

>tr|A0A1H0R2I3|A0A1H0R2I3_9ACTN Arsenite efflux ATP-binding protein ArsA OS=Nakamurella panacisegetis OX=1090615 GN=SAMN04515671_3368 PE=4 SV=1

MSDTIERPAVRPATRTRIVLHTGKGGVGKTTMSAATALAAARAGHRTLLLSTDPAHSIGDVLDLEIGSDAAPVNGVDGLFAAQVDTRGRFEEAWADIRGYLVGVLAARGVSELQAEELTVLPGADEIIALLEVHRRALEGQFDVIVVDCAPSGESLRLLALPETIRFYADRLMGAPARLMRSLAAGFAGLTGGRASSGPSAAQVSDALTGLLDDLADARAMLADPAVTRIRVVVTPERVVINEARRLLTGLALHGFAVESVLVNRMLPEIAVGGEFMAAWYAAQQACRPLIEESFGRLPLRQVRLSAVEPIGLSMLEDVAAQLFDELDPIPEAAPAPSLRTDGSDGRYRLLIDLPLAERSAVGLSRAGDDLVITIGPLRRRISLPSTLQRCRTVGASFSGDTLVVEFVPDLDRWPAALSEPLTRRSTSPTDHPWSEGRPASQSRSGEPRTGVTADLAGAS

>tr|A0A1H2Q6E4|A0A1H2Q6E4_9BACT Cobalt-zinc-cadmium efflux system protein OS=Hydrobacter penzbergensis OX=1235997 GN=SAMN05444410_10152 PE=4 SV=1

MAKVRKLLALAAGLNTIIFVGEMLGGAEGHSTSLIMDGVHNFSDELALVCLFLAYLLPVTMSRNFQRLANALNSIGLISISVFLIWQSVNNIIHPVPTIGYIPLIAGLLAAIANWGVARILYSIKDRNAAIRLTYIHIVGDVYVSLAPVVAGLLVLLTGKYIFDPIIAILVGVWLIWATIKEIAHSHDELIWPEHVADQ

>tr|A0A1H3V967|A0A1H3V967_9PSED Threonine/homoserine/homoserine lactone efflux protein OS=Pseudomonas sp. PDC86 OX=1882759 GN=SAMN05444743_13944 PE=4 SV=1

MMRFIQEVVLGSGAHCLIIVFGEQVSARQCERPNVIHAVVDDPYVRDCIQHNFLFASGSLLNLRAFPVDGPSMIDLATLAVFSGAVVLLLLSPGPNMAFVISHGMTHGWRGGAASALGIGVADLLLTALTAMGVTALVASWPPSFDLIRYAGVIYLLWLVSKTLQAKPRGAATQVERVRLGRVCVQAMLNSLLNPKALLFFVVFLPQFVRPEAGPIATQLWVLGGVLTFIAAVFHLVLGVFGGAASRFFSGRPGTATLQKWGLATVLTVLAVRLALMARPT

>tr|A0A1H6F5S4|A0A1H6F5S4_9GAMM Cation efflux family protein OS=Thiotrichales bacterium HS_08 OX=1899563 GN=MBHS_00593 PE=4 SV=1

MGGAILADVHILVACDLTVSEGHQISEVVHQTLLKASHDICDVIVHIDPEDDEEQPRNSDLPLRDTVLTQLQQKWQHIPAAKHIHHINLHYLAGKISMDIHLSADIVENFAQARHIAEQFSSSAKDLVYIKQIRVYIDPYPGLSDNK

>tr|A0A1H6Q8U1|A0A1H6Q8U1_9GAMM Homoserine/homoserine lactone efflux protein OS=Pseudospirillum japonicum OX=64971 GN=SAMN05421831_10110 PE=4 SV=1

MVFEIWVTFLLAICVVSLSPGAGAVAAMSTGLNFGFPAALWTLAGLQCALILQVCLVALGLGIILNTSMIIFEIIKYLGILYLVFLAAQNWFITPRSLENQQQPTQAYNAHKLFIKAMLINLSNPKAIIFMLAVLPQFIQLQDPLLTQYLVMILTMISVDLLVMGAYATFAYRVLNLLKKPSHQTLLNRSFSCMFLLAASALFWFEPSST

>tr|A0A1H6Z3I2|A0A1H6Z3I2_9DEIO Threonine/homoserine efflux transporter RhtA OS=Deinococcus reticulitermitis OX=856736 GN=SAMN04488058_10841 PE=4 SV=1

MSPHSLGLLLLVLVTLLWGSTFAVVKELGEELPPAVLIAWRFLIATLALLPALWLWRPRSAGAAPAQRGPARPLWRDGLILGAWLIAGYGTQTIALQTTTANRAAFFTALSVVLVPLWLTVAQRRRLSPALWLALPLAVGGLGLLSWEGGALVVGDFWALACAVTYAGFIVALERMASRHEALRFTVAQLLTVTALAWVWALLTVPGQLWPPAGAWGPLLYLGLAATALTTLLQTVGQRHVSAAEASLIYALEPVTASVFSFLLIGERVGPRGALGGALVVVATILSSRAEGHAHPELPAPATAEEPG

>tr|A0A1H7W950|A0A1H7W950_9BURK Glutathione-regulated potassium-efflux system ancillary protein KefF OS=Variovorax sp. YR750 OX=1884384 GN=SAMN05518845_117176 PE=4 SV=1

MLVAGAFLPGETPASVLLRYVRGMTTTTSTGTPADRPDGGSGGIYVLAAHPHWRDSRVNRRMLAAARAVPGVDVNDLYGSYPDFAIDVEAEQARLARASLVVLLHPIHWYSMPALQKLWLDDVLSYGWAYGPGGTALQGKDLWLVATTGSPEASYHPQNYHRYFFDAFLPPYEQTAALCGMRFLPPLIFYGARSASEVDVKSHVETFAQRLGSYPDWPEIEEIDVCVSCPVPESDRPADNDDVAKVVSNAFQAAMSHGLATATAIASADDNGRKAP

>tr|A0A1H8NKT1|A0A1H8NKT1_9RHOB Threonine/homoserine efflux transporter RhtA OS=Salinihabitans flavidus OX=569882 GN=SAMN04490248_103232 PE=4 SV=1

MRTESRPTPTSSATLSMGPVEWAMLLLLSVLWGGSFFFVGVAVRDLPTLTIMVLRVGLAALVLWGVIAVLKRPLPRNPRAWIAFLGMGVLNNLIPFGLIVWGQQTIASGLASILNATTPLFTVAVAGFLLSDERINGRKLMGIAAGFAGVVVMIGPGALSGLGTDVAAQFACLGGAVSYAFAGVFGRRFKRLGVDPVVVAAGQVTGSTLVLAPLALVIDRPWALAMPAPSTWAAIVGLAVLSTALAYILYFQILQRAGATNLLLVTFLIPISAIALGVLLLGEHLSGLEIAGMVLIGAGLLAIDGRVLNIGKRRSATSQATIRR

>tr|A0A1H8UWU5|A0A1H8UWU5_9ACTN Threonine/homoserine/homoserine lactone efflux protein OS=Streptomyces rubidus OX=310780 GN=SAMN05216267_10907 PE=4 SV=1

MPAAVAAFLAVLPAFLGACVLIAASPGPSTMLIIRQSLRSRRAGFLTVLGNETGVFVWGVVAACGLTALLAASQAAYDAMRIVGAVVLVGFGLQTLKAARAGRHADGDPVPGAPPLDGSDPDAVRPGAANSAAAPGSGPGDRDPRHEGLRAYRSGLLLNLANPKAAVFALSFLPQFVPAGEPSLPAMIALAAVWAVFEVGYYGLYVWFVARMRAVISRAGVRRRLEQISGGVLLLLGLRLAVEG

>tr|A0A1H8XV75|A0A1H8XV75_9PROT Threonine/homoserine efflux transporter RhtA OS=Rhodospirillales bacterium URHD0017 OX=1380357 GN=SAMN02990966_06235 PE=4 SV=1

MDRVSPKVLALLALLTAVWGTNWPLFKIALDELPVLTFRSITMVTAFILLTAILVVRRESFAVPKGKWPALIAASAMNILVWNIATSLAVLYIPSGHASVLSYTMPLWVALIGFVAFGQRLTGRLLAAILIGAAAVLALMLPNFASYERAPAGLFWGLFAGFCWAVGTFIVKRTAWPGMGLSLTFWQIVISLPPVLLGALVIDGVPDHWPSAKALTATIYTGAIPMALGTATWFALVKLLPAQVAALSSIAIPIVAIVSGVLLLNEPLSTLQTIAIGSTVVALWLALVPKRER

>tr|A0A1H9FT58|A0A1H9FT58_9ACTN Amino acid efflux transporter OS=Friedmanniella flava OX=1036181 GN=SAMN05421756_103374 PE=4 SV=1

MTRITARAAAPGLHPDRVTESRVGTVRGTALFVAAIVGPGILTLPALAAGEAGPASLVTLGVLLTVSAPIAFTFAALNAAAPAAKGVAGYATVAFGPLAGRLVSAWFRSGVPIGVPALGLIGGGYVAEATGGGKATAVTVAAGICAVAVVASVLHRPGSGVLTLLLSAALTVLIVGTAVVALPHGHTASLRPFAPGGLAAVAASALVLTWVLTGWEAVTNFTDVLRDPRRTLPRVTGATLVVVALLYAAVAVPEILVLGPTAGGTQAPVAAMLRIATGSAGAVLAAVIAVVIATGNSIAYVGSLAEMGTTTRPTRGARAARTGRASALVVPVIIIAGGLAAAALTSVSTGELVSVCAGSQVPVYVAGLAAGIKVLPTWSRSWWSSVVATAAVALLLVPAGRYLLIPAVVALGVVARYAYQCRSPGVRTAGPPRSRAEQDA

>tr|A0A1H9IMA2|A0A1H9IMA2_9GAMM Outer membrane protein, cobalt-zinc-cadmium efflux system OS=Solimonas aquatica OX=489703 GN=SAMN04488038_11098 PE=4 SV=1

MSSLSKAGRSLALLALACQTLLYAQPPAEAPPEPAQLSLPAAAQLALRFNPELAKFGYDRQALQGRQTQAGLRPNPELGLEFDNFAGSGAAREITLRLSQAIEIGGKRDARLNQSQRLLDRLDAEQSLAQLEVLAETTRRFIDVVETQQQLRLAERGVDYAQQSLAAARRRVAVGAASSLEINRAQIAQERALLEREHQEHLLSTLRRKLSEQWGRSEAQFEAAQAQLLELPEVPDYSELLARLRRSPDFARFDLERRLREADLRLAQAKAHGDPVLSAGLRRTDSAGDVAMVASLLMPLPFANRNQGAIAEARALRERVDTEQQAAQVRSEVVLYDMLQELRHARTVVESLQTTLLPQAEEALTLTRRGYANGRYSQLDLIDAQRTRLELERELIANAADYHRYLAAVERMTALAPAAAAP

>tr|A0A1I0BDJ2|A0A1I0BDJ2_MYXFU Outer membrane protein, cobalt-zinc-cadmium efflux system OS=Myxococcus fulvus OX=33 GN=SAMN05443572_101955 PE=4 SV=1

MDSVTPVAVVLARNLLESLWVGVGTRLALGQGMSLVSVALSLVLATSPSEAWSLERVVSESLARSPEVAAAQAEEQGAEGVRATDGRWPRANPSVELALVTDALTGDTGEQRTELVLSQALEVAGQSGLRVERASAALSAARARRHAVMLSASAGAVESAVELERREARATLARESLELTREMEAATVRRFAAGDVSELDRNAAALERARAEARAAQALAEVVAARAELNRRLGRSMDSALRVSLVDTATQPLPSSLEGEPPSLVAARAEVAASGSEVDLLRRERIPSPTVSLGYERERRPESHGAFSDVHTEHLLIARLSVPLPLWDRNQPELAEARARRKVRESEQVARERDVSAEQSVARATFDAARSAHEALMAVRPSVDRNLELVRRAYEAGELGLDALFLARDRAFAAAAEGVDAAAALVRARVALLRSVGRLPTGQVPE

>tr|A0A1I0WMV6|A0A1I0WMV6_9CELL O-acetylserine/cysteine efflux transporter OS=Cellulomonas marina OX=988821 GN=SAMN05421867_10371 PE=4 SV=1

MPPRDRLTALLVALCWGVNFPAIHLTLEQFPPFLAGSLRFAVLAVPAVLLVPRPQVPLRWLLGYGLGFGTLQFAFLYLAMDGGMPTGLASLVLQSSAPFTVVLGALLLRERVTRRQAVGIAVAVLGLTGIAVLRAGAHGAGGLVPVLLTLCGGLGWALGNLASRRAQAPDPVRFTLWMSTVPVLPLLALALVVDGRDAVVRSFTTLGTATGAWALAGLAFTVLVATLLGTGRWTALMARHPSSVVAPWSLLVPVVGIGTSWWWFGERPAGGELALAALVVGGVLLGSTARPAPPPQPVPPSLSAAPSRDAARSQPAVAVPAGATGTTRAVAIPATPSPLPVRPRPSVVVAETETGAPTAADSAASASARRGPSRGRLPTTCTTTLPTA

>tr|A0A1I1U4N8|A0A1I1U4N8_9BURK Cobalt-zinc-cadmium efflux system protein OS=Acidovorax konjaci OX=32040 GN=SAMN04489710_104106 PE=4 SV=1

MQSRTTTAVLLGLAQTLAWASSYYLPAKYLKFNERKAMSAGHSHALQAEGKERSIWWALGLTSAFMIAEVVGGLVTGSLALISDAAHMFTDTAALAIAVAAIRVAKRPADALRTYGYHRFEILAAAFNALLLFGVAIYILFEAYQRFQSPPAIQTGAMIVIAALGLVINLMSMRLLSGGKDDSLNVKGAYLEVWSDMLGSIGVIAGAVLIRYTGWVWVDPLIAVAIGLWVLPRTWVLLKESLNILLEGVPQGVKIPDVMVAMAAVPGVQSVHDLHVWALTSGKAALTAHVVYQPGVESESLLRPLQEMLAKRFQVLHTTLQMEATMCEHTEDGCNFVAHPSASAGDHIHSH

>tr|A0A1I1XPA7|A0A1I1XPA7_9DELT Outer membrane protein, cobalt-zinc-cadmium efflux system OS=Nannocystis exedens OX=54 GN=SAMN02745121_02988 PE=4 SV=1

MPLLSALLSAALLASPPPPAGCQGPLGRAAVVTCALAEHPSIRAAEAGRAAAEGRKLGARTLLPSNPHVEVTAGRRVGLWNGERDINVYGRVSQELEIAGQRRKRMAMADAEVAQADRQIELSRRDVAAAALSAYFEWIAAREQRAMIERIARTSDTLVDLARTSERTGLGSGLNADVVVATSVRVRRQQIEADRRIAAARAVLAGLLGRDGAGLEVEGDLAPLAVPQELSALLTAALTKRAEIELAKAEREVFVRQVEVFRRLRAPNPSVVLYAQRDGFAEQVLGGGLAFPIVLPAPLGRTYKGEIAESQALARRAEAEVERWRRVVQAEVEVALREVEARKAELALFEAERLQRAESHLEALAQEMATGRVSIRDAVVLQQTLLEYLAAHIEARRALALGSVELARVAGLLPEEAQR

>tr|A0A1I2P9G5|A0A1I2P9G5_9BURK Threonine/homoserine efflux transporter RhtA OS=Duganella sp. CF458 OX=1884368 GN=SAMN05518865_108122 PE=4 SV=1

MNGLARTLPLVSLLVTLVIWASVPTVAKAALAHVSLVTYLMLRYTLAGLFMLPYLKQSMAGASRLSWWSWAVLIISSCMIIYVQTWAIQQVTASWYIVVFSSCPVLIALLLRYRFTVRAIGGLLATVGGLALYLQDSHAAGTPFQLGALLGVLTGMLAWVAYTVIITRFHKVYDDTQITAICSYIGAIFSLVLFLAAGDYSVQQANWQVAAAIVVSGALMPLSLWCYSYSMRKAEALTIFGQYLEPLIGLFIAFLVFGAELTAVACGAVALILGGTIAVTRYSVKPANSH

>tr|A0A1I3HGY4|A0A1I3HGY4_9BACL Threonine/homoserine efflux transporter RhtA OS=Paenibacillus sp. UNC496MF OX=1502753 GN=SAMN02799624_00514 PE=4 SV=1

MKFDMKYAIAVFLGAVSYGILSTIVVKAYGRGYELGEVVGSQLLVGFVLSWLLAAATKRTAIRKRRNAVGLGEGGRSAAPARPALSWKQRLLLMAAGMPTALTGLLYYQSLRYIPNSLAIILLFQFTWMGVLVDALRRRKRPSNLMLITLAVLFGGTLMAAGILDHGLANFDALGAALGLSAAVSYTMFVLFSGKAVPSAEPAYRSAWMITGGLILVFVLFPPAFLFNGLIWGPLLLFGFLLGLFGAFIPPVLFAAGVPHVGEGMAAVLGASELPVAVMLSAVVLHESVSVLQWAGVVLVLLGVAMPEVVRRLPHSRPKGAAHRA

>tr|A0A1I3KU14|A0A1I3KU14_9FLAO Threonine/homoserine/homoserine lactone efflux protein OS=Myroides guanonis OX=1150112 GN=SAMN04487893_10156 PE=4 SV=1

MSTEVLYAFFATCLLLILTPGPDLIFVISQSITRGRKLGFAVALGQVGGLVFHLSLFAFGVSALIVSSDWIYKGIKILGGVYLLWLAYSAYTSETKIKLNEVEITSSSFGSFMWKGLLMNVLNPKVMLFFLALFPGFISEQAGNVKEQIFILGLIFTVLTLIVFSIICGVAARFTDVLSNNRVFSLIIKWMQVILFTVLGLYILL

>tr|A0A1I4VWZ1|A0A1I4VWZ1_9GAMM Outer membrane protein, cobalt-zinc-cadmium efflux system OS=Dokdonella immobilis OX=578942 GN=SAMN05216289_103143 PE=4 SV=1

MVLMTMLAACASVSRREGADRVQALVGQRVPDAGFWSQAPQVPAAIESRVNELLAAPLTPVSAQKVALLKNPDLAASFAKLGIAQADVVEASRIGNPGFSASALRDGGPSKITMGLSLPLSDLLLLSSKRRFAEGEYERAQQLIAAEIVTLCADVARAWYEAAGARQVAAMRDAVSRAAAASSDLAQRYYEAGNISALALKLEQASASQARIAASMARAESTRARLALNARMGLNGDMAGRWQLDVPLAAPAETEDELETLRALARENRLDLLAARREVDQLGEALGVVRRWRLIGNIDLGVEREREPDGGKLSGPSLALAIPLFNQGQAAIARAQAQLEIGRANLARLELQIDNDVVLGRDRVAAMRSIVEDYRSALVPQREAIVARQQERTDFMLSGAFDLLLSRQQEFDAYAAYLDAVRDYWVARTELGRAVGTVLPSDASISTRVIDVEALLAPTEVSAPEHMHHGGSADAMPGMDHSGHGVPAMEAGPADDQAPAGGEGRTMDHSGHGSPPIGPAPTGTHGAKERHEKAQDTSDAMHDGYEGSK

>tr|A0A1I5VZW6|A0A1I5VZW6_9FIRM Threonine/homoserine efflux transporter RhtA OS=Lachnospiraceae bacterium XBB1006 OX=1520827 GN=SAMN02910358_00784 PE=4 SV=1

MNKKLFGSICGIMTGVCWGVSGVFGQFLFETRGVESFWLVPIRMLSAGIFLLLYGTITDLPNTKRLLHNRRDFLQAILTGVCGTMMFQLSFFLAVQHSNAGTATVLQYLCPVITMLYVCLRDRHAPKKVELLCIFLALAGIFLISTHGNVGSLVITPAALLWGVATAFFMFLNTVIPEGIYKRYPSTVVIGWAFLFGGIALCLIFRPWNYSVQIDFAVVISMLFIVLGGSVFAYLFYGNAIKRIGPAKSSLFAASEPVAAAALSIIWLKTSFSVIDILGFVLIISTLFILSSTNA

>tr|A0A1I6J1X9|A0A1I6J1X9_9BURK Outer membrane protein, cobalt-zinc-cadmium efflux system OS=Mitsuaria sp. PDC51 OX=1881035 GN=SAMN05428960_0692 PE=4 SV=1

MKRLIFISPRREGAGSTAETTSQRRRPTSRRDTLVLAAALMAGFPMTYASTAATNAATATATATATATAVATTAPIASNSSKLPSTLVVTAPTPDANTDTTGVPFAAYLEAVERFSNAIAAQREAVAAARAGVPMAGLRPDPSLSLGVGPAELGREVRPKPRLAQSIGLSYTIETGDKRERRVAAARSQVSADEAALAGAGRQAAADAAAAFIEACRTREALQRQEVSLAALGDIVRMNERRHGAGDLGGLELLQSRNERDQFLATVVRARADARTAMEALAAPLGRRWREAFGDASPLCTFRDGQAPEGLDGVEGIEGTGGSVGIQESHEDEALDALVARALDQRDDVRIARAAVDSARAAADLVRANRWVDPSVSLSYGYTPQGRRGVAADGSAVDPSPRSNTVSVSVSVPIPLSRLDRSDLVQAESAVTQALLALRQTELQAQADVRATHAQYRAALENLTRYRDATLGDARRVVEGLRLSYRHGAASLLELLSAQRAADDTELAYLQARSELAAATVRLQLSLGRAPAP

>tr|A0A1I7B382|A0A1I7B382_9FLAO Threonine/homoserine/homoserine lactone efflux protein OS=Lishizhenia tianjinensis OX=477690 GN=SAMN05216474_2511 PE=4 SV=1

MSGLIIKGIVTGLILSIMLGPAFFLLIETSIRKGVKAALSFDAGVLVSDIIYIVIVYALYQEVSGFADGENNAVIKLIGGIVFLGFGVVLFLKKVKSQKSDNSGKMVHDSKDYIMLFTKGLVLNMANPLVIFYWFSVLAFGGEGNNKVSLEPLDVFIYVAVILLTFFTIDVLKILGAKQLRPFITNAVLKSLNRITGTILFAFGIFLVVQSCYLIMFK

>tr|A0A1I7FXR2|A0A1I7FXR2_9FIRM Threonine/homoserine efflux transporter RhtA OS=Eubacterium pyruvativorans OX=155865 GN=SAMN05216508_1042 PE=4 SV=1

MKKGYFYIAVTTIFFSLMEIMLKSMGNAFNPVQVTFTRFLIGGLVLLPLAVRHLRKKNLHFTGGDIRRFALLGLIGVAVSMTFYQLAVVYTQASVVAVLFSSNSIFVMVFAFLLLGEPIYRRNLVSLSLDIVGILFVINVLQMKLSLAGVIFTMLATVTFALYGVGGKKPTEKFGGLVNTCMSFLLGSLEMILLALLTYIPGVSAAMNHAGLTMFSRIPLFSGYSLSVLPAFLFVCVGVTGIGYACYFQAMETVSVNTVSLVFFFKPVLAPILALLVLGDPMPVTKIVGICFILAGSLANILPPMLAARQKAEPLAASLDEEAAGVERILTEEADAAERVLKEVNE

>tr|A0A1K1MVV9|A0A1K1MVV9_DESDE Threonine/homoserine/homoserine lactone efflux protein OS=Desulfovibrio desulfuricans OX=876 GN=SAMN02910291_00649 PE=4 SV=1

MISLENLLLFVPMAALLVMLPGPDFALIAKISLLNGRPQGQAAACGVALGIGVHTTAAMLGISAIIAQSVLWFSILKYVGAAYLIWLGIQALRHGRQASAAVVRVAPQADDLKEHALAHGLMKKPAAAPRLTGRQWWSFFRQGFLTNALNPKAVIIFLTFLPQFMNPHAPLGPQFLELGGILSALCLLWYVPLAYILGRVRHIFENSRFQLWLQRFTGFIFIAFGLKLAAAQSR

>tr|A0A1L8QRL7|A0A1L8QRL7_9ENTE Arabinose efflux permease-like protein OS=Enterococcus aquimarinus OX=328396 GN=RU93_GL000336 PE=4 SV=1

MPNLGVGLLWAMNMTLIPMLVATFNVSNSKAALLITMGSFTGIFVQYLSGLLSDRSNFKMGRRKPFMIMGSVATTIAMCAMPFAGSYWTLFVVAFFFYFSLNFYQGPYYSLIPETVDDSQLGLANGFSKVVSVLGGAFIFVIGPRLWASESVLNKNHALPFFVSALLGIFTVILTIVFIKEKKVPKADTKQKIAFDFYKFPSAMKLFLGIFFIYMGYGGITPFFVKYCVQYLNLSEGTASFSLLLLTITGALFAYPLGVLSDKIERKKVLVFGTLLFVVALFFGIFVKETMGLYLMMSVIGIGFIAIQVTSYSILAEVVPPERLGEFMGIFNFFVSSSQFISGNLMGLLLDRVGYQVFFPLSIVWLTIASVILYFSRIEKIGLQATPVTK

>tr|A0A1M4DZK2|A0A1M4DZK2_9ACTN Putative transmembrane efflux protein OS=Nonomuraea gerenzanensis OX=93944 GN=BN4615_P1504 PE=4 SV=1

MKKSLLVPIVLMVSVFVVGTSEYLIAGLLPQVAADLDVSVSTAGQAVTAYALGVVVGGPLVTILTVRLPRKGLALGLLLLFAAGNAVCAAAGSYEVLIVGRVVASLSHAAFLTLALMVTTRVVEPQRVGTAIAAVGSGFSVATLLGVPLGVLMGESAGWRTPFAVLAGLALAVTALLAVVLPRQEAAVTSVREEVATVLGRRVLVVIATTAVGLAATSTVFTYLAPTLTEITGFGAAAVSTLLLVYGVGSLIGGLVAGRLADRSLAATVRGTFVGLAVVLAVFPFAVPWAGSAVVAVLVFGLLTSATTPVLQSLVLRHAGRAPTLAVSVNVCAFNIGIAGGSALGGGLVAVDGLRWLGLAAAVLSLAALAISYAAVPRRESRESPESPESLTGSPA

>tr|A0A1M5C8J2|A0A1M5C8J2_9FLAO Threonine/homoserine efflux transporter RhtA OS=Chryseobacterium sp. OV279 OX=1500285 GN=SAMN02787100_2197 PE=4 SV=1

MRIAASLLAAILFIYHNEVSGYFNYYVVTLQAVKKIMKKKNILKGVLFVGIGASIYGMLATFVKMAYHDGFTTSEVTTAQFVMGLVGLLFLNFMQTITSKQKLSSPSAKEVRMLMIAGTSLGCTSLFYYIAVQYINVSIAIVLLMQSVWFSVVVESIIAKKLPNARKVVSVIIVLVGTILATNLINMDIELDWHGVFWGLMAAASYTMTMFTSNTLATHLPVFRKSIIMLSGGAIVIFGFLFFAQIGPMYFDGLKSLYLNFTENTEHIHSFNYSIFWTYGFVLALFGTIVPPILFNVGFPNAGLGLGSIVSSLELPVSVTMAFVLLGEKVLLVQWGGIILILFAIVLMNLPAKKELKTIEVA

>tr|A0A1M5FHI9|A0A1M5FHI9_9BACE Threonine/homoserine efflux transporter RhtA OS=Bacteroides faecichinchillae OX=871325 GN=SAMN05444349_14515 PE=4 SV=1

MIESKNIEDKNRSCYESYKRHIVCSGIFLYFWVSSVFSITLLLAGFSSFEVLSYRWGVAAITLTIIGLLSGCNFRLSRRDFIVVFCLSLFRAATSFSLIVAYQNIASGVASTIHFMYPLAVALVMMFFFGEKKSMWVIIAVLMSLFGASMLSSGELNVENGNTTIGLIGACVSVFSYAGYIIGVRKTRAVQINSTVLTCYVMGIGTIFYLIGCGCTTGLRMVTDEYTWLIILGLALPATAISNITLVQAIKYAGPTLTSILGAMEPLTAVVIGVWAFHELFTMNSAVGILLILLAVSIVVFRERRIKG

>tr|A0A1M5NRD9|A0A1M5NRD9_9FIRM Threonine/homoserine efflux transporter RhtA OS=Asaccharospora irregularis DSM 2635 OX=1121321 GN=SAMN04488530_11153 PE=4 SV=1

MEKKKGYIFICIAGLLWSTLGLFGNVLMGYNLTPEQVAFTRLFLGFLVLTIYSIVKNPSALKISKKGIIYSIMIGIVCQGLFNLCYFKAINSVGVCISAVLLYTSPLFLTVFSKVFYKENINIQKIVSLGFCFLGAVLAVTGGKLDVDKLNGLGLILGISAAITYALMPIISKNILKECSSITILIYGFLFGSIFMLPLAKPLEMLKYSLNPKILVWMLVLGIVPAALAYIFYVEGVAKGVELSIAGVIASVELISSVLIGWTVLGENFSIVKLIGLGFMVVSALIAVKASKQEEPLLSEDIEQNTLHEAL

>tr|A0A1M5RW70|A0A1M5RW70_9GAMM Potassium efflux system protein OS=Ferrimonas marina OX=299255 GN=SAMN02745129_1778 PE=4 SV=1

MTLWRSMGLTALLFWLVCWPAAANMPVTEQLKQQLEEVKGAADADPQLVQSYETLLATIDANEKATQANQTLRDFMTAYNGQMSALQQQLDQVPEDPLFGPPPSEEPDDVELALTALDSAEADWRRQLSRNKQAMQQVERLPEVLPGELSDLSRQLRELTPVEPNEATPVPYWQFLANTKKLNLAIEGRQLQLQSYDKKKNALELEQQLLQSQLSAAQTQRERLQGMLSQTKQTVAQDLLRQSSALVGLAPEQDAQAKKTAEQLKRLARELSELVSSNDDQARRRQQLEQQTRQLTSERELIANNIQWLQKSTAFGATLRAKLRALPEQAPNDDLVGQIAQAHVRQFSLRQLPPIVDPRTLVNESEVEADPSEPASEPSGAANRIQGEADNDSVLPWDPEQSAAFWQQAESLQQQLVQQLDEEYEQFIITLTHLQTVREQYHLELTSSLSYLKQQQLWTRSHPPLWQWPEGFNRFTLLGIEQPLLDTLAQLRLQQPRQFALALVAVLAFGFALSRSRQHRRALAALPTIKQSFKPFRAKLFASLTGAVLMALMVVAMSKLLTAFWPQPEPLDIQALLTLAVVITLLMATLFTLGSPGGVLRDHLDWPKDYCNTLQRQAIASGLPSVLLLLAMMLGTLLAGAHGSELVRWLQLAVQALLLVLFLRMVAPRSLERVLPTVLRRPWFLKGLQLVVLTTQAVAFLLTVLGYYYAGLSVTLYLSTTMMVIVLFFIAGQLGRGWLLAEQHELRQQRLREEWLEQQAARQSEEGSAPAEPMPEIDEEQIELDEVNQQSFALLKGALLIGLGAALLGIWGSAVEQVQWFNDVVLWQVIEQTESGATLVNISLRSVLIAIGLLLLTLFAVQNLPGMLELLVLRRLDLQPGSGYAITTILRYLVILTGVMTAFAMVGFQWSKLQWLVAAVGVGLGFGLQEIFANFVSGLIILFERPIRIGDIVTINNLSGTVSRINTRATTIIDWDMKEIVVPNKAFITDQLINWSLTDPMTRVVISVGVAYGSDIDKAEELLHEVARDHPTVLDDPAPQVFFLSFGASSLDFELRLYIPAIESRNFVIHAINKAIDRRFREANIEIAFPQLDLHVRELPKAPPEDKGNAPND

>tr|A0A1M6HVG6|A0A1M6HVG6_9GAMM Threonine/homoserine efflux transporter RhtA OS=Cycloclasticus sp. DSM 27168 OX=1884353 GN=SAMN05519226_1788 PE=4 SV=1

MKNETLKAVVLLNIAGLLWGGNMILGRYLADFLGPWSIVSTRLVIGGFIFILLLIQTGELKKIKHITNWWTFIALAIFGVIFFQSLLYYGLRLTTSTNAGLINSLTPLLTAFMAAAFLKEKLNYHHWVAAAVTIFGLLFILGEGDLTNLLLLNFNVGDLLVLGAVISWVIYSLIAKNAMIGMSPLLLTALGVLLSLVVVIPLGIYEAKVIQTPHLTVNAFWALMFISVGPTVLSLLFWNKGMKVIGPSRASLFLNTVPVYIIIINAVFLEEMPYQYQIIGMVLIFMGSFYAGLKAHKPKLRD

>tr|A0A1M6R7L7|A0A1M6R7L7_9CLOT ABC-type Na+ efflux pump, permease component OS=Clostridium cavendishii DSM 21758 OX=1121302 GN=SAMN02745163_03561 PE=4 SV=1

MSSLIKFEFKKLAKKRTNIITVVVTTILTIIFFSLPAINFECLDAKGFKAISLARDNIKNISIKMTEEQVTKDIKEIQSLYADPKNVTKDEKGEKWFNNDVYDKFINPRRDYLSMISENYANPKEFLWISGLVDIKLKDGAKFYETRDSKVSKLLNQNHEGGNYSEQEKKFWLDKNSKIDKPYTYGYYHGWDGILGIFGSLIFMLLAICITVAPVFAGEYQCGADAVILSSKYGKTKVIRAKIGAVFIFVTMVYFVNAIFAVGMPLLTFGVDGWNLPIQICNTIIPYNLTFASCTLISVGIFYLVMLGIVSFTLFISAKCKSPFTVLIVDVLILFVPLFLGDGADNGLYQHIIYLLPYQKSMIHLFSAYISYSFGGLTLSLISMRMLAYIVMTIAFLPFIGNAFRKHQVQ

>tr|A0A1M7RTD2|A0A1M7RTD2_9FIRM Threonine/homoserine efflux transporter RhtA OS=Desulfitobacterium chlororespirans DSM 11544 OX=1121395 GN=SAMN02745215_00070 PE=4 SV=1

MSDRNQGYLLIILSAVFYSTLGILGKFIYNTGIEMSLVIVLRLFATVILLGLFLLITRKEPLLTFSRAVLFQGIFFVATAITFFLAVKYLSAGLATVILFTHPALVAVLAVIFYHEKIGAAQIAGLILALLGLFFISGLCIESSTALSPLGLILSVLSAVVYGIYALLGQRVVKTDGIWTITFTISLMGLVISALIFPYNLSALLSITPYQLFLGFAMAFLGTILPVVLFLKGVQKIGSLVGTLISIIEIPFALILAYLLLGEVLTSMQVVGTLLILIATTMAVTIKHQKENDGRN

>tr|A0A1N6EST4|A0A1N6EST4_9BACT Glutathione-regulated potassium-efflux system ancillary protein KefG OS=Fibrobacter sp. UWB11 OX=1896202 GN=SAMN05720758_0280 PE=4 SV=1

MNNQITILLSHPNISNSMFNKHLVDINRKNPNFVFHHLDKNRVNGYFDLEAEKKLLKESKAIVWQFPIYWYNSPASLRDWQDQVMSPIVYSADNFLKGMPVRVVFTAGAAAEHYTHEGLNRYTAEEMLIPFEMTANAAGMKWFKPLGFYGCSPDTTKATLDKAAQEYEESLLELL

>tr|A0A1N6N677|A0A1N6N677_9GAMM O-acetylserine/cysteine efflux transporter OS=Lysobacter tolerans OX=1604334 GN=SAMN05421546_0115 PE=4 SV=1

MGVPAALSRRDFALLLFVCVVWALNFLMSALGLREIPPFTFTLLRFVVLLLALAAFMRVPPRDQWPRLAIVSLLVGVVHFGLSFLALRLSGDLSSPAIVMQSYIPMTTLLAWWWLGERFKWWTGLAIAVSFMGVMVIGFDPHVLSRPAALITMLISALALAIGTILMKGLRGIDMPNQQGWMAAASVVPLLGISLWLEPGALATLPGVSATAWAGVAYAALASSLLGHGLYYSLVQRYPVALMMPWLLLVPVIAVALGIVFWGDRPGTRIWIGGAMVLGGVLIIALRQRFKSRTQQPVTEVVAEYPQG

>tr|A0A1N6ZG85|A0A1N6ZG85_9BACL Threonine/homoserine efflux transporter RhtA OS=Paenibacillus sp. RU4X OX=1907395 GN=SAMN05880555_3299 PE=4 SV=1

MGRRARGSYWTAVLFVLLGASSYGVMSPLIKHVYGFGYTFSQVVVHQLAAGSAMLWIAAGAARKRSSASLSPRLSLGQWAGLALIGTAGLAMTTVLYNQALQGLKASFAIVLLFQFTWITIALDSIWNRRLPGWGRLGCVAVIVAGTVLALGIGGVSGPHAAALPLLCGLGAAFTYSLYLAGTGRFRSDLDPAAASAIMVTFGFILVLALFGRGAWAGEAEPRLILWAAVLALLGQVIPTLLFTIGIPRIGSSLAALLGAMELPVAAAAAWLIGGETLSALQLGGIAAILAGIALAQKVPAKESPASLEE

>tr|A0A1N7E5P8|A0A1N7E5P8_9SPHI Threonine/homoserine efflux transporter RhtA OS=Mucilaginibacter lappiensis OX=354630 GN=SAMN05421821_11341 PE=4 SV=1

MFMVFAGACSYGILSTFVKLAYQAGYTIEELSVTQASIGFIVLTTLTLIQGYYKKPEAMSIPVSAWLYLLLTGACIGMTSYVYYLSVKYIPASVAIVLLMQFTWIGILLEWLFFNKKPAAIQFIIIGIIWIATIIASGVQGTQNSHLPAMGICYGLLSAVFYAVFILINSRLKYAVSSLMKSSVMIMGSAISLIIFTGHQLLAVHHFNIQLLKWGMFLALFGTIIPPLLFASGIPKTGHFKSSVLMTVEFPVAMCCSWFFLGEHISLLQWIGVIAMLMAIVGIKRKSA

>tr|A0A1N7M5T6|A0A1N7M5T6_9RHOB Threonine/homoserine efflux transporter RhtA OS=Rhodobacter aestuarii OX=453582 GN=SAMN05421580_105129 PE=4 SV=1

MKTDTGGPNAAPHSIAPADLAGADMPAPPLPEPEKSLIGKGIGLLLIALLFFTLMDVAAKKLGQSYAPAMVIWARFAVNLALVSLIFRGSFLKHARSRQPGLQLLRGAFQMATVALFFLAIRSIGLAEAAALTDLNPVLITLGAALFLGEKIGPRRVAGIFVSFLGALIILRPGAGVMDPAALFALAAAFTYAGGALMTRVVRHDSTATSVIWSAGVGTALSSLALPFFWQEVAPVDLPLFIAVGALGAAGQAALIVAFRHAPAGVLAPYGYLGLVLSSLWGWIFFAQLPDLYTVAGAAVIVLAGIWVWNAERRAALNAAAR

>tr|A0A1R4GLE3|A0A1R4GLE3_9MICO Putative transmembrane efflux protein OS=Gulosibacter sp. 10 OX=1255570 GN=FM112_13745 PE=4 SV=1

MVARNEGSGERMRENVSRPIGGGARRGALLVAVLAAVFAVPLSVSGTAVALADIAASLGESAAGQQWALNGFNITFAASTLAWGSLADRVGRRPSFQAGAILFIAGSVLSVLAESYVVLDAARILAGLGAGAVFSVGSALLSVVYAGEGRARVFSLLGAMAGLALAFGPTLCGIIAQTWTWRAIFGVQGGLLVVSFALMQAARPLLRDEPRSTAPFDWPAAILFFAAIASLVAALVTGSEAGWASAPTLGLALIAAFAFAALLRRERRAEHPLLDLALIRQPRFLGVTLVVAVASFTFAAGAVVSAWLLVPAMVMLGAGFGLHAGLVDNEGLAAAPDEDAGMAAGWINTMRVGTEAVAVSLFGAVFIPALGGGGEPGAGLRRDRARRGSRRARPGGDLDPRHAPPMSARASWRLRYSCSCTTGPRRCRSKRPGCRWSPWPSATPRRCGKRSRTPACIDTSAERPHRSGS

>tr|A0A1R4IUK9|A0A1R4IUK9_9GAMM Potassium efflux system KefA protein / Small-conductance mechanosensitive channel OS=Pseudoalteromonas sp. JB197 OX=1434839 GN=CZ797_03395 PE=4 SV=1

MKLIENIPLFKQMEIINRLGFFKEFSLNERQILLESFGLLYLVRENSFLFKQHDNDKRLFIVLSGALIVFKHNHLLELGTIRPGEFIGEGAFINNRARSISARAKTDAIVLAITSDALTRLPNVIREKIKDRIIEGMSLRIAKLSEHIENHG

>tr|A0A1S6RZ73|A0A1S6RZ73_STRHY Transmembrane efflux protein OS=Streptomyces hygroscopicus OX=1912 GN=SHXM_09667 PE=4 SV=1

MCLSRAAIRLETACWLIPSSSAAAWNWPVSATATNVRSTSTSTLPPYFHNCWLCLAWRRVV

>tr|A0A1S7U895|A0A1S7U895_9RHIZ Arabinose efflux permease family protein OS=Agrobacterium deltaense NCPPB 1641 OX=1183425 GN=AGR7A_pAt20117 PE=4 SV=1

MIAGRLKQTSWWLVLSVQFVTVAVPLAVLPFLSLHLQRLSGAGTTDIALWAAVIAAAPAIGAILSTPLWARLASSYPLGRLMGLSCLLNALSALLQAQSGTVEVFALGRSIQGLTGVGVLLLLAVEHSRTTRGSGYSGLQQALAAGCIAGPLLGGWAFDNDALQGLLTGFAVLLVALSVFCGYVFHDARPVKDEEQSSGFAGLLPPTPTRMLVLSGLLATAGASGFMPFFAGWALEQESAVLTASLIGLLHAGSWAAAIVVLPLWGRWIDAGRERAVMRLSTAGSLFALVSLLAGSTVVLISLSRIVHGAFNSGLAPSLFSVLGRSRHRVANLAAGRMATTLGQAIGPAICGLAVFAAGNNGALFAAALLTLLASILLYLQPEARSHDVE

>tr|A0A1T4K5B8|A0A1T4K5B8_9FUSO Threonine/homoserine/homoserine lactone efflux protein OS=Cetobacterium ceti OX=180163 GN=SAMN02745174_00309 PE=4 SV=1

MDMIFFKGVATGLFLSLPFGPIGIYCMEKTLVKGEKEGYISALGMVTVDIIYGLLAYLFINQIGHLILQYQSFVKVLVGCFLILIGYKKIKANIEIKDISHENKSLLQDYLGTLVICLFNISGILAIAGIYATLNVGLTGDHAGFFTPFKLASGILTGGASLWFLTTFILYNFKKKITNLMLIKISKLAGFFILIFGIFAIIFAFYK

>tr|A0A1T4XQZ9|A0A1T4XQZ9_9FIRM Threonine/homoserine efflux transporter RhtA OS=Gemmiger formicilis OX=745368 GN=SAMN02745178_02185 PE=4 SV=1

MKQKETAAVLMVLLAAACWGANGIFINILTAYGVNGTQMTLVRMASMAILTGIWLAAKNPAALKIDLRDLVWFVPAGALGLFMFGLFYTYSIQLVGMGTAAVLIYLMPSLVMLFSVVFLHEKFTPGKGLCLVLSLLGCALVSGVAGGVTLDAGGVAYGLGAALCYTLQNILLATKLKKYSPMTNLFYMFLFSAAASLVFTAAAGELPGVAYILTTPGALAANLGLGLVCSLAAQWLYTAALKTIPASRASIAATFEPVAAALFGLVLFGQKMDGFGVAGIVCEVAALVLLQLPAPAKRKG

>tr|A0A1T5EIX1|A0A1T5EIX1_9MICC Arabinose efflux permease OS=Arthrobacter sp. 31Cvi3.1E OX=1279032 GN=SAMN05660916_02894 PE=4 SV=1

MTGHGNHAKRAALWLCLGAGFITLLDQSMFVLAVPAMSASLHVDSGSVQWILAGYSLAFGVALVPAGRLGDIVGRRTLFIAGIAVFGASSLVGGLATDPSLVIIARLLQGLGAGTLNPQVLGLLQDIYSGHDRAKALGAYAAAGGSAAVCGPLLGGLVLSLGDPSIGWRILFLANVPLVLVLVPLAFRLLPRPAGKHPENGTTKTSIDVLGALLLGGVVIASLVPTIYGAGIIAVTSLAIGAGALLAFAGWEILYHRRGRTPLLSASLVKSRGYTLGTVVALCQFGVGAGMAAVTAFYFLSGTGMAPLAAAAILAPQAAGMLLASSFSWRFVARYGRAGIVYALVGSLACLIAKDLFVQMLDGGTAALAVAAVGLAQGVATGLVVAPNQTLTLAHAPAGTAGVAAGFYQLSQRFAAALCSAAAAGMFLQAQGAATGQASKDAFHQGIVMCCVLLGVALLAGGLDWFREARDRRNARAAVVTSGKETATAAVALTSERPTAAGAQRSEATESVDA

>tr|A0A1U7GES1|A0A1U7GES1_9BACT Efflux transporter periplasmic adaptor subunit OS=Planctomycetales bacterium 71-10 OX=1895807 GN=BGO49_24950 PE=4 SV=1

MFMLKSMLRWAAVAAIAAVIGIGAWLYYDARERAKLPPGIVSGNGRVESVQVDVAAKYPGRVLRIFAHEGDLVRAGQVLAQMDVAELEAELAAGKAKIAEGNETEAKIKADILSREAAVRYEDQQFIRNRELFSRRYISREEMEQTQTKVDIARTQLDAVKAQLLANERSIEAATADVQNTQAKIVDSTLVSPVTGRVLYRLAEEREVLGAGGKVLTLVNLDDVYMEIFLPSDEAARVDVGSEARIVLDAYPQYAGRARVSFISPEAQFTPKQVETRSERDKLMFRVKLKVPQEKLLPYIEKIKTGVRGVGYVKVDPNAPWPEKLEHPFPPPAAMGIKPAEGAKPEEKPESPKAEEPAAKPSGAEKPTNP

>tr|A0A1U7PNZ4|A0A1U7PNZ4_9BACI Threonine/homoserine efflux transporter RhtA OS=Edaphobacillus lindanitolerans OX=550447 GN=SAMN05428946_2298 PE=4 SV=1

MAMKQYLGDVLLLITAVVWGSGFVVTAIALEHLTAYQVMAGRFVLATLILCVLFHRKLRTLSRSVLWKGAVLGTILFVAFALQTVGLEYTTPSKNAFITAVNVVIVPVIAYLVFKRRIDRHEGLGSVLALAGIGFMSLQGSMTINIGDFLTLLCAVGFAFDIFYTNLFVKKEDALSLTIVQFATASILSVGAVLVLGEVPAGLNGAAIWPVVYLAVFSTTIAYVCQNIAFRYTTPTKAAVILSLESFFGTAMSVLFLHEVLTGRMVLGAVLILTAILIAELKPAFKFRPKIRPEH

>tr|A0A1V4X8C4|A0A1V4X8C4_9DELT Magnesium and cobalt efflux protein CorC OS=Syntrophaceae bacterium PtaB.Bin038 OX=1811716 GN=corC_2 PE=4 SV=1

MSDLLLLLPIAVCLALEGLFSGGEIALISADVHRIRQRAEAGSKSAAIALRLLDNPEWFLATCLMGTDLCVITATALATSLLISVFGPARGEWVSVAVMIPTILIFGEIVPKSYFRHRAERKAVFIAPFIWAASWVFYPFVFMISKIARGAVYTLAGERGKLSLPYITKDGLKHLLHEEALGTDVKHMEKEMVDRIFDFSETSVGQVMVPVSNVAALEENATFGDASKLINETGFSRFPVFQGNVINVVGVVNAFDILKTMPASASRPVREILRQPLFVPVSKPAGDLLLEMQRRGEPMAVVVDEYGGAVGIVTIEDILEEIVGDIRDEYDKRERDVRKLAPGRYLVTARIAIERLQEILPLGIPEGPYETLAGYLLHQMGRIPRRMEQFRAGGIQYVIEDADLKSIRQVQVILPADPAAVKKEEGPPEPQRPVGGL

>tr|A0A1V5CXS7|A0A1V5CXS7_9DELT Toluene efflux pump outer membrane protein TtgI OS=Syntrophorhabdus sp. PtaU1.Bin153 OX=1811705 GN=ttgI PE=4 SV=1

MILTRWRGAKAWARKYVPAMACFLLVFLLAVPVFCGTMSPTTPAPPVTPKAGEQFTLPAVIDYALKNNPRARISARDVETETYGIDAAKAERMPRIDFGSGAARYRYPMPLTPPVISGPFGSGLEIPEYDRNIYDAGGSFRLPLFRGGRLYRGVRVAEIRKAMAEDNLASTRQELVYNLSSVFYKIAQLDKLLAANEANVRQLEAHKQDVEFLLKAGSVPQLDLLKTDVELSHAVENRLLVRNNLESTYELLKTLMGIDDMTKEISILHQTVSGGPLPPLEESVSKALSQRPEYRAVEKKKRIYEERVKIAQGKRLPDVYAAGEYVGKAGDAQSYKENWYAGVRLSIPVFDGGLIAAEVNKEKVELQKVREEERSLKLSITREVKDAYLAVANAVERIDVGTKAIESARENVRVERLKYQSGAGTATDYLDAQTAYLRAETDYCQALYDRETALAFLRKAVGEHWPNGAAGDQGMPEK

>tr|A0A1V5FPK6|A0A1V5FPK6_9BACT Magnesium and cobalt efflux protein CorC OS=candidate division BRC1 bacterium ADurb.BinA292 OX=1852824 GN=corC_2 PE=4 SV=1

MTLALILTAIALCMALSFYFSGAEVAIVSANRYRLRSMEEQGDASAGRLVELLEDSQRLLVMALVGTNLANVLTALFFKLFLQRGWPELAATEALGVILWSEVLSLLILTPILIVFAEILPKALFRAHADALIHRLHFSLRLCLVLLKPVIWSIERVAQLILSPWSESHRATMRQLTREDVITLLSPEPAAAADSTTADAVEETIAHDAAERERHEPFGEAIAREHDGEEERLSESADQRRMIQNIIELHETLAREIMTPLVDLVAVDLKRYDLNMLKSLAQQSGFSRFPVYRDRIVNLIGYVDIFRVLREDDGTRKLEDFIERAHFVPETKRVDDLLEEFLQMRIKNAIVVNEYGGCSGWISREDMLEEIVGELEDELDEPGDEIVEQAEGVYLADGRTEIDHLNDVLGAEFDDQEWETIAGLFLSEFGHIPQVNDSVCVDGWRLTVVRMDGLAIDTIRLEREKN

>tr|A0A1V5HIJ7|A0A1V5HIJ7_9BACT Magnesium and cobalt efflux protein CorC OS=Lentisphaerae bacterium ADurb.BinA184 OX=1852901 GN=corC_2 PE=4 SV=1

MMTFLLMVAILACLLAMGFFSGTETALTSVDPLFIHAQEQRGDRNAPRVRQLLSRMEMVLVTTLVGTNLMHVSSATLAELLLHRHVPGQWEALVNTLLMTPIILVFCEMVPKATGRTHANRLSLLVARPLRAVELAFLPIVALVNVLSTGAARLFGGPRRRRGAVTRDDLQVITDMAAEEGTLPEGAVGMVQTVFELRDRPVSSVMIPLMQMAAVREEAMVEDALRLSAVTGFSRFPVYRGHIREVVGILDVRAVLYRLPAENLRQSSAPPRAPVRNFMQADFARVPEHRPVGELLHELHFHKTPMAAVINRGGAVIGFVTTEDLIEEVVGDIRDERETDAHTAADAAAR

>tr|A0A1V5IRA0|A0A1V5IRA0_9ACTN Threonine and homoserine efflux system OS=Actinobacteria bacterium ADurb.BinA094 OX=1852790 GN=BWY94_00760 PE=4 SV=1

MTSSGSGRPLGATTTAGARRSRGYLMVGSTAVMFGATGVWVGMTDLPVSTVLVMRMGLAAVMVALLGGGRRWLRQALRPGVLRRLLLLGVIDALQLYTFMLALRRLDVALAVFLSYMSPIYIALIAPRLLKQRTEPVVVVALVLAVSGIAAMLAPGLFEPGLRAAPDGIALGLVSGLVLAVFFLLAKALSADVDGSTLLISDAAVVAAVMLPLGLVQWAATGFAFSSTDLWAVLGLAVFSTAVSGTVFLHGMRYIPVQHTSIVGLLEPATAPVFAFVFLAERPSVWTLLGGLLILVGAVLVVVFGAAEEGLAGSPEEAVGEAGAAEGRGPL

>tr|A0A1V5L5E7|A0A1V5L5E7_9BACT Magnesium and cobalt efflux protein CorC OS=Verrucomicrobia bacterium ADurb.Bin474 OX=1852931 GN=corC_1 PE=4 SV=1

MNAPDNLAKLLVVVWATGSFMLFLLNGTRAALIDMRYRSMRAPIDEERKRWNTLIQWVRSSHPHHRLFRLLHPVFALVNSAAFGWIVAFLFVRWGFNNSITWFSGLLTFILAMAGLALHLWMLELLPRTLAVRFPAPFLFLSQALMPMAAWLVHPALKLADSMDGLMRKALIRSQHPLVEPLDHELQISALDIDDPDMTPVTEKIVAHALGLNDLTVYDVLLPRNQVQYFDLNDSLKDNLELSRTTGHTRFPLCEGDLDACIGIVHIKDIYRFRGAPEKLDLRKIRRDIIRFDLDMPLDTALQTLLSKRIHMALAQDEFGGVAGIITLERILEELVGDIHDEFDRGEERMIIRIKKDFFRVSGIAPIHELEEVLGIDIDNDDVSTLGGLVVAELGRIPEPGETLGMGRMVITVQEADEKRVLSCTLKLLPPLSEEPID

>tr|A0A1V5M1A0|A0A1V5M1A0_9BACT Drug efflux system protein MdtG OS=bacterium ADurb.Bin429 OX=1866930 GN=BWY76_00703 PE=4 SV=1

MSASLTHTPPTPPVPETQPRNPLAAAFGILFAYPVLIPLGLVTLFAQLTYSGVNNVTMHQYIKILGATSRNDGIILGWVGAMFLLSETFLRVPFGWLSDRFGRAKMVILAMLLSAPSFFISSTVTHYSWLFPLRWWDGMMAAALWPSVFALIGDTVPARARANAMGAINMMYMLALFTGGALAGILLDRSGSPRTFFVVGSAVMLLGGLTALTFFRTCPQLDAPHPEVHIEDEERAALSPLRHLPLLVITFVQNFAILILAQLLFDYVRHDLGFTLKQIGLLVGAPVVAIALFALPLSRVGDMVGKIAVVRVAFTAVAIALWAFAFNKTLIGLSIITAIIGIAFAMGIPAWLAIITSLSGRKSRGVTFAAYGTVQGLAAVCGPIAGGYIWNTLGHSAIFMASAAAISLGALLAWTTLPEHPKSSS

>tr|A0A1V5NUY2|A0A1V5NUY2_9BACT Magnesium and cobalt efflux protein CorC OS=bacterium ADurb.Bin374 OX=1866937 GN=corC_1 PE=4 SV=1

MTLLDGLVFLLLLCGSAFFSGSETALTSVSDVGLASLSDKGDRRAKMALSLIASRGTVIGALLIGNNIVNTLLAVYAATVFDSMILNSPLPAWMAPVAASVLSITVLLIGGEVLPKNIAIRFNERISLWVAYPCHYLVKMLTPILAVLNLVNRLVLLVIGTPKDRTGPSADELLAMVRMSQKAGIIDPMERELIGRSMFLNETMAREIMIHRTQMCAIAETASMNEVKDIYTKELYTRLPVYRESLDQIVGILNIKEVFRFDHLRETFRIPDMMSQPIFFPETARIGVIFDKMRQSRTHLAVVVDEFGTTSGIITLEDIVEQIFGEISDEYDQGAARIRWVSPRVFEAEGRTSISEIQAELASKKLPQLSEEACEDVETLAGIALRQTGRIPASGEAFVFEGFRFQVRKATGQKIQVMSVRVPDPEPERDRERTARQHPTGPEAAPMDNA

>tr|A0A1V5QAE7|A0A1V5QAE7_9CHLR Magnesium and cobalt efflux protein CorC OS=Chloroflexi bacterium ADurb.Bin344 OX=1852860 GN=corC_2 PE=4 SV=1

MEPGSCTPYEFNTHYLAFLIVFLFLYFLFTVIYSVFRFNRRPDIFSEENEKEKDTENASALSPEQVNVIWLAAKINAVIVSIFAFILINDSFCLYRSQWLLLLSIILIPLVLYFLEALISGIAARNADSWIKYFHGIARLTVFFFKPLYSVYESFKPDNIRNDYTFNEVEAHLREWVNNAPDNAALKEDERKMVRSILHFSDTLIREIMIPRIDMTAVDVETSLEEATSIVLASGHSRLPVYEDDIDNIIGVLYAKDLLKIYTENPENRALRDYLRPALFVPESKKAGDLLSEMQTSGIHMVIVVDEYGGTAGIVSMEDIVEEIVGEIRDEYDDSEEKLINEISENEYSLLGRIDLEDVNEILGTHITRESADTLAGFLYSQIGKVPVGDEEIEVEGWIFRIEELSGNRIRRVHVKKLEEDSPNKEGEQDEE

>tr|A0A1V5RTH8|A0A1V5RTH8_9SPIR Magnesium and cobalt efflux protein CorC OS=Spirochaetes bacterium ADurb.Bin315 OX=1852912 GN=corC_1 PE=4 SV=1

MNISLAVVFALLVLSALFSATETAYTSLSFFQLKTLENRKSRAGKLAYALSQDKDHLITTVLIGNNIVNLSASALVTTITIKYFSSALVGYTTGILTFMILVFGEIVPKRLALVHNVRIAILMAYPIKTLMLLLFPLAWLLQVLSSAITRLFGTKEEPVITTEGVMHVVDAAEDVGLVDQYESDLMQRAIHFSSTTVRTIMTHRTEVFTLPSDLTIEEAFPKIIKSGFSRIPIYQDNQENIVGILLLRDVLRAQQKQVVNKTLASLSRKPIYVVEQMHLDDLFYLFKKNKLQQAIVIDEYGGFSGVVTMEDVAEQLFGELFDEHESRFPDRVVQHKDKPGTFVVMADAPFQQVVDDLDLWYEGDRVSTVAAYLLQEAGSIPVEGEVITTDLGTWQILLMKGNKVEVVEFTPNPASVD

>tr|A0A1V5UPG1|A0A1V5UPG1_9BACT Putative amino-acid metabolite efflux pump OS=bacterium ADurb.Bin243 OX=1866936 GN=eamA_1 PE=4 SV=1

MLNFSITLILFSLLEVSSKPLMFFLDPVVLTFYRFLLGLLTMIAFAYHKNILSDIKKISYSDFKILSFLGIINIALAMSSLQLAVKYSNAATAAVIFCSNPFFVFIFSILSKSEKFNLKCFSGVVLGIAGVVLVMSRHGFHISHGALFAVMASMLFAFYIIVNKKVSANCRPVIVNIVSFFAGLAVTAAYLLISAKGLYLPSEIFTSYKYIMILLFLGIAVSGFGYITFINTIKKYTPISASVIFLLKPALATIFSLVFLGEKLGALFYYGLLLIMSGSWLILSSKYYKTN

>tr|A0A1V5Y0V2|A0A1V5Y0V2_9BACT Magnesium and cobalt efflux protein CorC OS=candidate division BRC1 bacterium ADurb.Bin183 OX=1852823 GN=corC_2 PE=4 SV=1

MSELVLLALAILVCSAMTAFFAGSETGAISANRHRLRNLQKSGDERADDTIVLLSDSQKILTITLVGTNIFSILGVLFAKNFFEIILESLNVHEAEGIADIVSLLTMTPFLLIAGEIIPKRLFRKYPDQLMLAFRKPLKFFSIIFMPAVSFFNSITYILLRPLGIKKGITQSNLTREDLQNLVESVEPAPSHGHRMPAPNGEADMIQSIINLEKTLVREIMKPLVDIIAIPINAATRETIIDTALRTGYTRIPVYTNYIFNMVGYIDVYDILRGDATAWKDLKSEIKDACYVPETKRIDDLLQEMLGKHISVAFAIDEYGGCSGFVTLEDILEEIVGEIDDEFDKSTFTFSEQKPGVYIVDPRMDLDDLNEKIGISLPKRHCETLGGFIYSTLGRVPQVNESFIFGDYRITITEMKTPKIIRVQIEQIKTKPEIDEQRE

>tr|A0A1V6DIS4|A0A1V6DIS4_9BACT Magnesium and cobalt efflux protein CorC OS=Verrucomicrobia bacterium ADurb.Bin118 OX=1852928 GN=corC PE=4 SV=1

MEASTVSATLLKLLAVLGLIAFNACFVAAEFAFVRLRDTQLEGLILKGHRRARIARRLVRNIETCVSAIQLGMTFCGLATGAMVQPVFRALLAPLFALLEVESVIARQTTELIVGFVVSTVLLVVVGELVPKAVAIRQTLPTALWTAQPLAWFSRLAYPVVWLLNYLSQAFLRLLGIRPLSESEVPHTEEELRLLVGAAQERAGATRLGRNIVLNALDLRHRIVRDVMRPRQEIVALDIEADIAACLEVVEQTNYSRFPLCEEGNLDRTRGVIHIKDLYALRNKARSGADLLPAARALIYVPETGRLEKLLTLFLERKLHMAIVVDEFGTTVGMVTLENVLEELVGQIQDEFDQEKPLLVRLSERVWEASGLLPLHDLGELVGEPLHESGIATTSGWVTQRLGGFPQPGNTLRLGQFELRVEEMEGLRVSRLKVTRTVESPPATPAPD

>tr|A0A1V6F7R1|A0A1V6F7R1_9BACT Magnesium and cobalt efflux protein CorC OS=Candidatus Cloacimonetes bacterium ADurb.Bin088 OX=1852835 GN=corC PE=4 SV=1

MPLIIEIILWTALMLLLLALMFLFAGFETGAISINQIELENRAKKNKSLYRLLDYVRHPDVFLGTTLLGTNITTVLLAAISTYLVHRINSPFFNPKYTALIVGGVALIFGEVFPKAYFRSHADTLVPKVFPLMRAISYILSPFVLVVTWLNRGVRKLLKISGEQDFNYLTKDDLAYLLSITSTDAKDEPQLEMIEDALDFTEQEAHNVMVPRTDVIAIQESATIAEAIEIAREEGFTRYPVYRQNLDDIVGILIIYDVLKREFTPQTPVSKLMLEPYFTPENTDLDVLLREMQKQHRSMAIVVDSYGGTSGIVTMEDILEEIVGDIEDEYDVEDEAPDVQQVSPNTWLASADVEIDTLAEDHGIDLPEGDYETLAGLILDRLERIPLRGQVIELEPWRIQVLQATEKKILKVKLHKMNKRGEST

>tr|A0A1W2GLI0|A0A1W2GLI0_9BACI Threonine/homoserine/homoserine lactone efflux protein OS=Bacillus sp. JKS001846 OX=1938743 GN=SAMN06272738_2729 PE=4 SV=1

MLARSNVKSRRAVVLFAEPEEKEKGFDYYNTYNRRIFMIITLFTYILLGLSLAIPAGAMTVQMTKQGMRNGFVHGWFVGIGGMTVDLSLIVLIYLGFSSVLTNPWVETVMWLLGFIFLMFIGIESIKEAKSEVNIDGEDPNKSLLSAYLSGFMIAISPANIVFWIGVFGPVLVSSLGNASTSTFILIAVGILLGIFMHDMILLSFVHFTRRFLNPTFIKRVSIFAAIVLFGFSGYFGYEFIKQVMTFISKA

>tr|A0A1X6XA45|A0A1X6XA45_9CORY Magnesium and cobalt efflux protein CorC OS=Corynebacterium xerosis OX=1725 GN=FM103_01800 PE=4 SV=1

MDLIGPLISLGVGLLLVVACGGFVAAEFSLITANRNDVEAAVTNGDKRARGVLEGMKTLSTQLSGAQLGITVTNLGIGFLAEPAIAALIGPALVDLGLGTVAARSVSVTIALVLATAMTMIFGELVPKNMAIAQPLRTAKAVVGFQRIFTTIFALPIRLFNGNANAVVRALGVEPQEELGSARSAEELSALVKRSADEGALAAETASLVQRTLAFGDRRAHDAMVPRGRMDSLDVDDTVEDLLELARTTGHSRFPVLTDENEIAGVAHIRHGLAVPFEARPTTLVDTVMGTATFVPDTVPLDDLMDTLRSGGLQMAVVVDEFGDHAGLITLEDLVEEIVGEVRDEHDEETDDTPEPDGSWDLDARMRPDEATERLGVTVPEHEDYDTLGGLVTMELGRLAEVGDEIVVATDPAPGEGPAQLRIEVTEVDGLRIETVHVKVEPLADEHEDAEDTDGQEDRSTRREREKAERRERSERRDSEKAERRAAKDREESEAAR

>tr|A0A1Y6B841|A0A1Y6B841_9PROT Threonine/homoserine efflux transporter RhtA OS=Tistlia consotensis USBA 355 OX=560819 GN=SAMN05428998_101593 PE=4 SV=1

MSGDGRDGRLGYLLLASITLFWGVNWPAIKLSVGVLSVWDFRLLSAGVGGLGLLTIARLGRERLAVPRSQVGPLLLCALFNVVGWHLCSAFGVLLMPAGRAAILAFTMPLWASLFAVPILGERLTATRVYGLLLGLAGLAVLVGPDLVVFRTAPLGAGFMVLAAVSWGLGTVLLKRFRWTIATTTLAGWQQLAGALAIGLAGLAAGGLDPWPADIAPVNLAAIAYAVSVPMIYCFWAFMYTVRLLPAPVAAIGTLAVPVVGVFSGALLLGEPVGLREVGALLLICSALAVVLVLPAWRGRRA

>tr|A0A1Y6CAI3|A0A1Y6CAI3_9PROT Threonine/homoserine efflux transporter RhtA OS=Pseudobacteriovorax antillogorgiicola OX=1513793 GN=SAMN06296036_113137 PE=4 SV=1

MMTVKPNLSLRYGLLAIFLWSTVASAFALTLELVSVYQLLCLSSLTSLLALALAMLVTRKWTKLLELRISQWLQLIGLSLLNPILYYICLFSAYDRLKPQIAQSINFSWPLFLALGSTLIGGQGKNRSSLMFMIPSFLGLLLITSQGQSFSGITSNGAGILFAFLSAIIWASYWIISSQINIDALVKLFAVFLVATPILALISWLDAPSAWLNYNPKAILGSLYIGLFEMGLTFFLWLKALEHSNDKVMLSNLSFLSPTLSLIWIQVILGESISLFTWVGFALILCGNGAPQISSSIRRKMTSQS

>tr|A0A212RT41|A0A212RT41_9PROT Predicted Co/Zn/Cd cation transporter, cation efflux family OS=Arboricoccus pini OX=1963835 GN=SAMN07250955_1148 PE=4 SV=1

MTAADATATTNAALEQRLLRISALATLAIGCVGIATGLLIGSRAIVFDGFYSLTDVLMTCVSMLVARLVALGASRHFQFGFWHLEPMLVAFNSSVLLLACGYAFLDGLSTFLVGGRTVAFGAGAAYALGVGLASLGIAAYAKRASRNLHSSLVEIDIRAYVLGGFLSLGLFASFVLGAVLTSQRATDLAPYVDPVILMLLTLVLAPVPIASLIRALAEVFQLAPADLDAEVDALMRALVAELGFKAFKAHVAQAGRAEFIEIDVIVDPAFPVSSVADLDAYRQRIADGLKPSAATRWLTIAFTTDPRWT

>tr|A0A221K649|A0A221K649_9RHOB Glutathione-regulated potassium-efflux system ancillary protein KefF OS=Sulfitobacter pseudonitzschiae OX=1402135 GN=kefF PE=4 SV=1

MHTHIVHVHPETASYNGALTRTAEHALRTAGGMVTVTDLYRAGFDPVERFGHYANRVEADRFAALGEQRNAWATDTLPGDVVDEIDNLERADLVILQFPLWWHGPPAMLKGWMDRVFISGGLYTRKMRYDAGYFRGRRALISVTTGAPRAAFGQGSRGGDFDTLLWPVQYSMHYMGFSVLRPFVSYGVQGHGYSYEGEGRLRDRLSRNLDDWSSCLLSLDEVEPLSVPSWADWNEDGSAIASRQN

>tr|A0A238WXH7|A0A238WXH7_9ACTN Threonine/homoserine efflux transporter RhtA OS=Actinomadura mexicana OX=134959 GN=SAMN06265355_103433 PE=4 SV=1

MSPLKQDGQGGLTAVMSRLGMFMGFGAMIVVVISQGSGLALGSALKSFQGSFSIVLVGTVIVTIVTVLKNGMRARGMHLSVAAVSTPEVWREGLRSLRWSLFTRGQRDAIFSLAVTSALINLGGVVAVRELGNGVNAAFSTAGALAAGALLLRFAPQWLLRFAVLTAVVFAAIGTGQGNLSLLGLVAALCAASHMWNLPKRVVRLGDKSDEGLTWANLISAPPVLIGTFWWDHSQGVSWEWGGKEIFGAVCAGLLVMVIPVFLQNWAGSRGVSEQDMGALSSLSSPLHAVVGVVLAPVTLALTGKEPVLPTFNQWGFFIIVAAVAIIAPQLPKDNRWKIQQAGPGVAVREDDEACEQPPVPHGDLDEQPQAPLVEPKNPWVPRQRGEEQSTSRTDVEHPVESGVGGSAYETPAWAVAQGANLDPDEGKLILTPRGRTVLTEEGVHYPGGCSLTYRKGNLSVEVEFVDEGTFDVGAFVGLRATGITVVIGGTEMRYPDAEKFSVDLRTGRFHVTKPGDVLFGGRAAE

>tr|A0A239W504|A0A239W504_9ACTN Arabinose efflux permease OS=Cutibacterium granulosum OX=33011 GN=SAMEA4412665_00314 PE=4 SV=1

MRSYREVLAIREVWTTILLSALTRLPIFGLTMLITLHVVETLGMSYRLAGAVTTFVTISSMISAPWRGSMTDRRGLRRTMIPSIVVMTAVYAVAPWLGYYPLLVILALGYLWNYPIYTIPRQVLIATVPLRKRRAALSLDAVSIEICYMFGPMVSIIIASSVGTRATMIGCAVLAALGATGLTVLDPPIAETSGAASEGQTPSSSSPDGNPSPDPRSATEPVAPTQSSATETLAVEGTTHDSDSPTIDVPTAHPSTTPALAWVNRFTVAILIGCLAAGYTLGGIELTTVGAMREMGSTQAIGWVLAFSGLGSALGGTIYGALNRSVPMPALLTLMGLTAAATTLATSPLQAGLILLIGGLFVSPTLTASIDQLTNLTPPSRRGSVIGWQGSFLNAGVAISAPTIGAVIDGVGWHQAFILSGAIAAVIGLAVGAVMRTRGS

>tr|A0A250JAW3|A0A250JAW3_9DELT Macrolide-efflux protein OS=Cystobacter fuscus OX=43 GN=CYFUS_006164 PE=4 SV=1

MIRGAMQPSHSASSLLGSNRNYRNLFIAHTLSVLGDWFNQFSLLAIVYLKTGSSAYVGLTLVSSALPALLLGPFIGALVDRSDCRRVMIISDVARVLLAATFILTVDWVWSIYPLLALMSLFETAFSTARNAIMPSVVDKPQLPIANVLMNVARGMMASVGAALGPIISGLIGQNGAFWLNSASFALSALLISRIVLNAPAQAHSRRWSREDLLAGYRYVLSNPIVLGVFITGVAWSLLGGAYYVLLTVYGAGVLKGGSSGIGVMYGAQGFGSVVGGLLVLRFLVHDEVRAIRLFSWGKIAQVLVFFGFLFAGELWTGAALILLMRTIGGLLTPYDTTLIQAYTPHELLGKVFAARSTFVEFATQLCTFVFGIWLSFYNEPRITGAVFGLGSLVLAVTGFVILNSRASRDMAAQRLEPG

>tr|A0A257N9K4|A0A257N9K4_9GAMM Potassium efflux system protein KefA OS=Methylococcaceae bacterium NSP1-2 OX=1917481 GN=CG439_295 PE=4 SV=1

MKIIAFSIVPSLFWWAKKPAYPTLLLLLITLCSGNVFAESPTTPPPPEPTNYNASQDITKDSLQAKIDALTARKGLDEALKSRIIAAYQSAQDELNNIKAFNEREIAFKTAIQQAPDLTKKLQKDIDQASEKPPKPNEEDFVKIPVEELTQRLVIEQDKVKQLDEQISKLSNELIEEQTNRPNLIRQERLNAKQELDEANKAIQDAIANADSDSDAKLAQDAQKIYLKTQIDAKTAKLNMLDAETLSYAPRLGVLKTRLQLLGLQKEAISPVIDTIENVLSDLQQQEEKDRQNALSQAEKDLAGKPLVIQEITRENIQYSQQLQTINGKISHYNEEKATADKQISDIDANFSSAAKKIDLASLSPPLGKILHEQRRNLLSQDKFVSQSESIQTETATTNLGQLAIEEKLKKLDDFDGYLQHKMELNVDKKLSRQDRMKIQAELRVLLNYQVELLNKLSVAYNSYLRTLGDFDFVRQQRHNKVEKFALYLDERLLWVRSSELAFTDNVVSEVYRSTLWLLSPTNWNSILKNVANLPAKNPFLTLFALLNITILLLAKNWAKRRLKITSAKVGKIYTDNFHYTLEALGYTLILVAPLPLTVTYIGWLLSNVSDSNFTQAVGLGLNRVAIAWFFLQFFYRLFEPTGIMRNHFQWQEDPATLMRTQLAWIRFVILPCGFIIRVTLASGVPAYSDNLGRLALNISLLAVVLFLTKLLHPRHGLLQHIVIGDALEWTRFVRYFCYLAAFSPLIIIGFSVTGYYLSALELQQQLMVTLALIFIIHILYEIALRWLTLANRQLVIKNLQQKRKSSADGQKHVSVTGSEDPVLPIDDEQVDIPEVNAQTKTILNVLFCFSLVVSFWMIWKNIFPAFSFLERIELWQNKTIINNKEVYQSITLVNLFLAGIYSFITVVSVRNFSGVTELLIFRRVSMEAGSRYAVNQLAKYTLTTIGFFCFANELGFNWSQVQWLVAALSVGLGFGLQEIFANFVSGIILLFERPIRVGDTVTIGNVSGKVSRIHMRATTLIDFDQKELIVPNKTFITTQLVNWTLSDAITRVVITVGIPYGSDIELAHKVMLDAVCATPLVLKDPEPSVMLIEFSDSALTFSVRVFVSETANRIPVTHALHIRLAKALSEHNIDIPFPQRDIHIRSIPSEWCANKAI

>tr|A0A259BET7|A0A259BET7_9GAMM Magnesium/cobalt efflux protein OS=Halothiobacillus sp. 24-54-40 OX=1970385 GN=B7Y07_02160 PE=4 SV=1

MNDIHLGALFTALIALVLLSAFFSSSETALISLNRYRLRHLAKRGHGGAIRAQKLLEKPDQLLGLILFGNTFANILVSSLATIIGLRLFGDSGIAIATGALTLILLIFGEVAPKTAAATAPEPIAWPAAYIYSFVMPLVLPLVRLIGLLANGLLKILGFNTNRHRSHGLTAAELQTLVRESSQHLPEQNVNLLLSVLELEQAQVEDIMIPRAEIVGIDLDEPWDHVLAQIKSASYSRMPVFNGSVENTVGVINVRRLFGPLMDNTLTLSKFKRLLREPYYVPEGTALTTQLLNFQTENRRSALVVDEYGDVQGLVTLEDILEEIVGDFTTSPLPDNTDIINEEDGSFILRGNMPIREINRELAIDLPTTDASTLNGLIIEALETLPTQGARITIENVQIDVLSVQNHAVDTARLKFIDEDNEPNTKSQTHGAG

>tr|A0A285PGC9|A0A285PGC9_9RHIZ Threonine/homoserine/homoserine lactone efflux protein OS=Cohaesibacter gelatinilyticus OX=372072 GN=SAMN06265368_3872 PE=4 SV=1

MISQLATAILPMALFSLTTSISPGPVNFIALSLGTQKHKRQAFAFVSGATIGFTVLLALLGLGMEQLLTTYQPLLTIFNLLGSLFIAYLGYKIFTSQSPISTNQNNGGGFMHGFLLQWLNPKAWGSCLAGHAAFQTSNAPELLALFTLIYLIVCFVGVGSWAVAGTQISHLLSNQRTLCLFNRIMGGALILLALFLALQPYL

>tr|A0A286DJW1|A0A286DJW1_9ACTN Arsenite efflux ATP-binding protein ArsA OS=Streptomyces zhaozhouensis OX=1300267 GN=SAMN06297387_101313 PE=4 SV=1

MSGAPRTLLVTGPGGDGVSTVAAATALASARSGRLTLLLSREPADRLGALLGVPADALAGARPVEVAPGLWAGRIVSGPPFRAAVLAAQRQARTALAALGSATLEEDELTELPGAEQWATLAALRAAQADERWARVVVDLPPAVDAVRLLALPGQLRRYLRRLLPQERRAARALRPLLAQLANVPLPAEALLGAAVDWDASLSEAEGLLADPGLAVRLVFEPTARSVALLGTAHAGLALHGIGVEEVLANRLVQGRSPDRLVRALAARQDALISGFAQSVGRSEAPRRVPHMGPDPDAATLAGLLPAPADRAIPRPEPVLEDRLAEDGRLVWRLPLPGAVKHDLDLVRRGDELVVTTGPFRRVLPLPSAPRRCVVDGAAFAHGELAVRFTPDPERWPRDAEGGSEGRTAAASAGPTAPTTPAGSEGAERGEAQRAGGGRGEAERDGVFERGGESAG

>tr|A0A286G4D3|A0A286G4D3_9PROT Threonine/homoserine/homoserine lactone efflux protein OS=Caenispirillum bisanense OX=414052 GN=SAMN05421508_101521 PE=4 SV=1

MTLHTVAALLATCFLAAVLPGPGTVALSARVMAQGVRRSLVFVAGMLSGDVVWIAFAVTGLTVIAKTLGPLFLAVKIAGGLYLVWLGIKLLRTRHDDAADNGPTPVPTEPSAWRTYLSGLALMIGNPKVMLFYVSVLPTVIDLHSLSLPGLIATVGVIGVGVGGGLVPWIVTASRLRGLMRSAVARRRIDRGAGVVMVGAGAAVAAT

>tr|A0A286RKK6|A0A286RKK6_9PLAN Cobalt-zinc-cadmium resistance protein CzcA, Cation efflux system protein CusA OS=Thermogutta terrifontis OX=1331910 GN=THTE_3895 PE=4 SV=1

MTAARGARIGSFIVFADDWGRHPSSCQHIFRHLLDEFPVMWVNTIGMRPPRLDLFTLRRGLEKVGQWLKIFDARPSPAILPRPNICEKTPRVVNPVMWPRLRQGWERAFNRMLLKWQLAAEVRRYPRPRILVSTVPVAAILVSALQVDRWLYYCVDDFRNWPEMDKEGIGDLERSLVEVADVIVAANEQLKKHIEEYGKTAVVITHGINWEIWSSPACQATPEPVSDWLQRYERPWIVFWGSINWQVDANAVAAISERLKRGSILLVGPVNTHDPKLKESARVTMPGPIPQVMLPVLACYADVLIMPYRRGPGVDESEPLKLREYLATDRPVVVCDIPATRRWADALDIAATPEEFAACVDFRVKTGVDPRQLEARRRVRGESWAEKARQFVEVAFDSLG

>tr|A0A2A9HFD0|A0A2A9HFD0_9CHLR Threonine/homoserine efflux transporter RhtA OS=Thermoflexus hugenholtzii OX=1495650 GN=A9A59_1261 PE=4 SV=1

MTTRHILTLALLSVVWGASFLFIKVQLDAGLDPLGVASVRTLLGAAALAPFAAAAVRRARPAPRDAVLLAALGVTNFAVPWTLIALAEHHISSGMASIANSTAPLWAAVLAVAFLREERVNRTKGVGLVLGFSGIVILAGPASLVHLSSDAAGVALVLASTLSYAASAIAIRRALGHLSPAVIAFGQVAAAAAALFPAAAATGAFAGVDWSPHVVASAATLGILGSGLAVVAYMGLIQQIGAVRSVLVTYLIPPWGVLFGWAFLSEAISWNLLAGLGVILAGVLLVQGVLRLPGARPPESAGSAALGK

>tr|A0A2D3WPN8|A0A2D3WPN8_9PROT Efflux transporter periplasmic adaptor subunit OS=Sulfurovum sp. UBA12169 OX=2015906 GN=CFH81_00865 PE=4 SV=1

MKKIIKIVLYVLAGVAGIFAFYAAYNAINKPKIPENFAYGNGRIEAAQINLAPKVSGRLLEIYVEEGDIVEKGQMLARLDTTELEARWEVASAQIKQAEQNKNRTMAIVEQKKSELALAQENHKRGESLYQSKSISLLQYQQYETAYKIALANLKSAEADVEASHAAIEAARAQAQAIRVTIDDSTLYAPKKGRVLYKLLQPGEVVAGGQRVLVILDLLDTFMTIFLPTAQAGVINYDSEARIVLDAFPRIAIPAKVTFISPQAQFTPKQIETQNEREKLMFRVKVAIDSDLLKEHIDKIKTGLPGVAYIRIDQTIPWPEQLRNVPKSYREDSR

>tr|A0A2E1JZC7|A0A2E1JZC7_9GAMM Magnesium/cobalt efflux protein OS=Legionellales bacterium OX=2026754 GN=CMF43_00545 PE=4 SV=1

MSSGKTPSSIIDKIASILGYLRQPSNKDMFRDYCNQAISQKVIDSYEGQQLIRILDLEQKAAEDIMITRSAVDVIQDSDSLDHIKKLIRKSGHSRFPVVDKDNKKVLGILLAKDLVVSRSKKSALDLVRKALFIPENKKLNNLLAEFQRKHQHMAIVINEYGDFTGVITIEDVIEQITGEIEDEHDPQHIGKNIVKENNGYYAVEGITPIEAFNQHFKCQLMDDDIDTIGGLVLKYLGYIPSKGEKLTIKQFEFIIRSASERQIKWLLVKKLKKSETNQSENT

>tr|A0A2E3QK85|A0A2E3QK85_9GAMM Lysine efflux permease OS=Gammaproteobacteria bacterium OX=1913989 GN=CMQ39_03630 PE=4 SV=1

MEWLGSLSCSVGSVINVISHPFITGLLVGFSLIIAFGAQNIFVLNHGLMRLYVFPIVLFCSLADFTLIWLGIAGIDYFEDSLELYKAEILAFAAXWLLFYAIXKLKSAAVGNILRENSGPGYASLSQTFGTLLXVTFGNPHVYLDTVLLIGTISMQFSAVEKAYYGLGACLASLTFFFSLGYLGVFLGRFLXTAWIWRLIDIGIAIIXLXISLSMLEGGGWIKV

>tr|A0A2E9BKE1|A0A2E9BKE1_9BURK Bcr/CflA family drug resistance efflux transporter OS=Variovorax sp. OX=1871043 GN=CMO32_06805 PE=4 SV=1

MKTGTAAVAAMGPALVVGVVTLSTAVQPLGTDLYLAALPAIRNEYAARVGVVQLTLAVLVFSFGLSQLLWGPASDRFGRRPVLAAGFLLYAAGATVGAMAPNIEVLIAARAAQGLGIAACMVCGRAIVRDLFEQQHGTHVMTVAMSVLACLTMLIPVTGALLAQTLGWRATLWSMALCGLAGALLVLARVPETARSLKPDALRLGPLLAGYARIARDPAFQSWTLLNAFGYAANFGFFSSSAYLFIETFGVSRVGFGLVIGGASVTYLAGTMLCRRWIAAHGIVTSVRRAGFLSLLAALVLVVPQFAGAHTAGTLTAGLWLMLLAYGIHQPCGHVGMATPFPLQAGAASALGGFIFAGAAFLCGGWMGLMYRSGSAAVLSLTTGVLAAAAGTIALTLVQRHGRPVPARPAVP

>tr|A0A2G7QIN9|A0A2G7QIN9_ACHLA Drug efflux system protein MdtG OS=Acholeplasma laidlawii OX=2148 GN=NCTC10116_00553 PE=4 SV=1

MKKFMLFYLIIYFVQGIVTNLHHPLMPYYVEAIGVPNFMFGFFFSFMNLGMMFGGPFWGNLADHNKKKISLIIGILIYCLMQILFGLGHVFDMWTLSAFRLISGFGMASALTILTGEMIITSDKDKRAKNIAFGAAAVGLGGAIGQFLGGFIHTNSFFIKAFRTDIFFNAFLLQGISVGFFAVLIAIWFKPKKTIVDPNKKRVQFWEGFKEVRNIKPELLYFLVALTLITIASTNVEKYLDVYFKDLGFLAKELGNFKMIAGVVSLLSGIILVPLFMNIKHRLKLISVFQIISAVLIFTVFRSSSSLFLILLYTMYMAYIAIKAIFTPLEQDHISKFSGNHNVATTLGIRQSFYSVGTIIGPIFGAFLYDYSPRLLFDTSVIFFLISLVLIFISNHYRKKDFIQNSINSNI

>tr|A0A2H1IG72|A0A2H1IG72_9MICO Threonine/homoserine efflux transporter RhtA OS=Brevibacterium antiquum OX=234835 GN=BANT10_00850 PE=4 SV=1

MTVAPRVVPEPNPSTALAAMVVLAGALCLSISAILVKLAGVDAATTAVLRCAIAVIALVPLALFERRRRGGLSRAGVLWAIAAGVALGIDYIAWTASIYLVGAGVATVLVNVQVIVLPLLALVIDRERVSARFLISLPLMLIGVGLVGGIVSFAEVGEHAVLGTGLALIAGIGYGVYMFLTRRGTRRKAEGTIQPLAWATASAAVTAAIIAQFTGGIGFTGIGPSSWMYLIALALLGQVVAWLLINRRSVRLVPSMTASLLLIQPVLALVLAALILGETLTVGQALGAGLVVVAVAVANGVWQMRSRRRRQVLRGPDSAPR

>tr|A0A2H5VHM1|A0A2H5VHM1_9BACT Magnesium and cobalt efflux protein CorC OS=bacterium HR07 OX=2035402 GN=corC PE=4 SV=1

MSIAVGVTILAILTVLSFFFSVSEMAIASVSRLRLKTMIQEHPRQARALQALSENPTALITALAIVNNFVNLFASSIATVLTFQLLPALSGSETALVATLLITIYLLIFGEITPKHLGKNNAERLTPLVIGPLYWLSKILYPLTVAFQAIAQGLLRLLPEHYRQREPVHVSEDQIKLLIEMSEERGMLQEEEGEMIRRIFVYDDLVVRQVMVPRTHVVAIEINTPLAEVREIIAREGHSRYPVYERSLDNIRGILHAKDLLRFGYAEKQKLDEYKKKLQDELPRRMKEATTPEELKKLTEEKAFYEAEIRRLRELLSNARLEHIIRPAFFTAPNKPIRKLLRDFQKNKKHMAIVVDEYGGMMGVVTLEDILEEIVGEIRDEYDEPEEKKAALQIKQLSPTVYLVDGETPLDELNARLSLELPISEAVTIGGLLLHRLAEIPKVGTTLVVDGARITVAEATEKEVRKVRLEVLAMSKV

>tr|A0A2H6JPM3|A0A2H6JPM3_9BACT Antibiotic efflux pump outer membrane protein ArpC OS=bacterium BMS3Bbin14 OX=2005737 GN=arpC PE=4 SV=1

MVKIGFTRLVSLCLLTWVLAAGLVLGSSLKTARAAGILTLDEAIRAALANSPQIKENQAVVRGAVSGTKAVRAGLFPQVNGYARYDRFSDPVSVVPIQGLNLPPPLFSRDQYQAGLSFRVPLYEGGRLRGGIRAAARDEGIARAGLAYSRENLIAAVTDTFNRILYLKALRRAKEKTLAAIEETRKEAALRLKLGRIAPLDLMEIDTQVASERVDLVRTRETLKRAGQQLCLLLGRSPATGIETRGSLEKNGKEEADLVASLTGPSGRRRLKACIGKRPDIIRAGKMVEKADELLRIARGLRLPNVDLVGDYGRHAGAGLDGEEGRWSAGIHVSLNIFNSGLIAAKVAGAMAKRAAAAEALKGLVLKAESQVYAALSSLREAGARITLAGQARLTAAEAYKVETLRYKKGTGTVTDLLQAQAAWWTAKALYIRALFDRQQAVTALRLATAVTWPAGPSGGQQ

>tr|A0A2I0BL97|A0A2I0BL97_9MICO ACR3 family arsenite efflux pump ArsB OS=Microcella sp. HL-107 OX=2035245 GN=CLT70_1307 PE=4 SV=1

MTTVPRPEGVGPVLGWPAQHPVPIILVGVGGGFALGTLAPGLATVPDAVVATVVAVAIAITLLPVPLASLGRAVVERRFLLAVIGLNIVVSPVLAYILSRVVFRDPDLQLGLLLVLLAPGVGIVAIFVRRAGGAVESLLSTAPIMLVIQAVSLPALMVLFTFADGFLTLDLSRLPLAMLFGIVLPAVVVTVIQLIASRAPRLQRLTRQGGALAVPATALAAAFVAAVWLPRATERSDLLSAVAPLFGVYLILMTPIGILVGTAAGLTLSQVRALTFSGGARNGVLVLPLAMAFEEGFELVPLVVVLGIGIEMIGLFIYRLLVPSVVQQSRGPLAQE

>tr|A0A2I6QNQ6|A0A2I6QNQ6_9BACT Magnesium and cobalt efflux protein CorC OS=uncultured bacterium OX=77133 PE=4 SV=1

MTSATAITVKLLAVVLLVAANGFFVAAEFALVGVRSSRIETLVAQGSRSAKRLMELLQNLNAYLSACQLGITLASLALGWIGEPAVAALLAQPLSGLSETLRHGIAFAIAFSIITSLHIVIGEQAPKLMGLAMAERVALAVALPMQLFYRIFSLPIRALDWASARAVGLVGIKATAEHASTYTEEELRKLIDISRESGHLRAEERRLIHRVFEFSDTVVREAMVPRTEMAAIPNTCNLEQITKAFDQHRYSRLPVYRESFDDVCGFIHSKDVMPYLLHPEKFKLEDVLQPPLYVVDTARLEHVLRQMQQAKMHFGFVVDEHGGLEGIITLEDLLEEIVGDISDEHDEEVNEQITEIDKHTFVLDGGLAVRDLNRRLKLSVPESEGYTTIGGFLMTEAGHVLKPGEVVQHDGLVFKVERVEKRRVMRVKLEIQQTDGEGEIEDELDRARSSTGNTGLAR

>tr|A0A2K8SXP6|A0A2K8SXP6_9NOSO Cobalt-zinc-cadmium resistance protein CzcA, Cation efflux system protein CusA OS=Nostoc flagelliforme CCNUN1 OX=2038116 GN=COO91_05519 PE=4 SV=1

MSNSNIEQFGNLVQDAQRDDEYINATKWCKHFGSRLDNWKQLPETKARSKHLKITESNTEPWIVERVGKTWVTWVHPIMAVHLASYLDPAFANYVAEIFIRYAEADPTLAADIASRQNTVEGLDIINEAVQKQYSLIFARDWLCETSKIRFDFLDKDPWLRHELSESNLVFLLNNKFGLPCKGLVVIHLINEELLEDFLKDKYPNIYQSACRIVQLPSGQHLDISMADETALFSYFFREINDWVKEKGGTDCSFLK

>tr|A0A2N0UUU1|A0A2N0UUU1_9FIRM Magnesium and cobalt efflux protein CorC OS=Ruminococcus bromii OX=40518 GN=corC PE=4 SV=1

MTSDIIMGVVILVLILLSAFFSAIETAFSFVNKVRVQRYKDDGNKKAAAALYIIEHFDNALTTILICNNVVNLSCSSIATVLCMNLFGDAGSAIATGATTFLVLTFGEIVPKCLAKEHCDAFSLKTAGLLRGLMTLLTPLVWIFTRFKMIALKIAGSSGDAPSVTENELKYIVESIEEEGVLEESESEMVRSALDFDETTAEEILTPRVDITFISIDDSPEKIKNIIIENRYSRIPVYEGTVDHVVGILHTRDYLERLADGKAPDVKELMQPPYFVFKTQQLSKILNAFKRTKIHLAVVTDEYGGTLGIVTMEDLLEEIVGEIWDEDEEIEHNYYKIGKGEFLVNGDMELEDMLGLFDMDEDSLECDSVTVGGYILEHAGTIPHKRDNIEADGFKFTVMEVKDQRILRVVVKKSDTSEENESDEKSENKKSE

>tr|A0A2N2RWX2|A0A2N2RWX2_9PROT Bcr/CflA family drug resistance efflux transporter OS=Betaproteobacteria bacterium HGW-Betaproteobacteria-4 OX=2013716 GN=CVU31_08275 PE=4 SV=1

MLASLASLGPFSIDAYLPSFPEIAEKLNATQLEVQQTLSIYLLSFAVMTLWHGAIADRFGRRNVILVAVGLFAVASAGCTLATRIEHLWFWRAMQGITAGAGIVIGRAIVRDLYDGAAAQRLMSQITMMFALAPAIAPVIGGWLQSWFGWRSVFAFLVVSTAALWLACWKLLACAAVSLNFGGFFIYVLSAPVFLMTHLGVPETGFLWLFGPAMAGMICGAGLSGRLAGRISPSRTVLIGYLVMGCAAAFNLTLNLALPPGLPWSVMPIFVYTTGMSLAMPSLTLFALDPFPEQRGLAASCQTFFQSGFNSISAALIVPVLWGSTLSMALGMAGLLALGGLAALLHQRWRHKPA

>tr|A0A2N5NC54|A0A2N5NC54_9BACL Potassium efflux system KefA protein OS=Paenibacillus pasadenensis OX=217090 GN=B8V81_0068 PE=4 SV=1

MNFLHQPLAAANPAASPPPSASPGPSASPEPSVAPLPTDLEGMKEEVANKTSAYWDAMTSYFDSQFWINATIICIKVAFILLVGQLIIFVVGKGIDKVMERETRVQQRTRRVVTMGRLLKNVTNYVVYFITGMLVLSMLSVNVAPLLAGAGVLGLAIGFGAQSLVKDVITGFFIVLEDQFAVGDVIQTGTYKGTVELIGLRTTRLKTWTGEVHIIPNGTISQVTNYSLNNSLAVVDVDVPNDVPIETAAERIRDMLEKIDNPNLVKVPDLLGVTSMTTAEYKLRIVAECMPNTEATVSRQINRELQLLLRSGEGPQEALA

>tr|A0A2N7R3E7|A0A2N7R3E7_9PSED Glutathione-regulated potassium-efflux system ancillary protein KefF OS=Pseudomonas sp. AD21 OX=396378 GN=kefF_2 PE=4 SV=1

MHALIVVAHHQPRSLTHSVATQIAEGLTQAEPANTCEIADLYAQGFQPVFGAADFAVHHREALPPADVQAEHARIDRADALVLVFPVYWWSMPALLKGWIDRVFSNGWAFDYGSDQKHIKKLQRLRVHLVGLGGADAGAFQRHGYAAAMKAQIEHGIFDYSGATVQSSTLLLESESSDPQGHLQTAYKIGVRIFPVPGGSEPAREGTRAVELIAS

>tr|A0A2N9KFR9|A0A2N9KFR9_9LACT Cation efflux family protein OS=Leuconostoc suionicum OX=1511761 GN=LES9216_01595 PE=4 SV=1

MNQKNIEQRSLIIGCLWLFLMGISALTAYFATHLEALFVDAYFTLITLTTALLSIVISKISTKVSTRFPNGLFVLEPLYAFFQSLLTIILLTVSLITVGGKAYQYFVYGHGHLLNIAPVIPYEIIMVILSLTLSYFYKHQNKKIHNTSTLLSAETKSAMIDGIMSAGIGAAAFFILFISKNSPLSFLLYTGDSFITVIIVLFIIRVPLRIMKNALIEISGGLTQDQGIKSFIEGSIRSHLSNDFAINDCKVYKVGMSFKACIAISSKTHMIDTEKLAIYKNNILNDLSQKLAFINIVFVYSNVGKNEKS

>tr|A0A2P7QRI3|A0A2P7QRI3_9SPHN Efflux transporter periplasmic adaptor subunit OS=Sphingosinicella sp. GL-C-18 OX=2116704 GN=C7I55_09660 PE=4 SV=1

MQTGDSPPLSWRPRAVPAALGAALLLLAGAGLYTAGAASGSDPAAPLNRPAAASADATSLLPLDAHVEPRTATLVTAVQGGQIAHIRAADGSMVAHGAPLAEIANPQFVLAVASQEAEIISRLGDLSAQNLGLQRGRRADSQEIAAAELALHEAEDELRRQTRLFEAGVVTAARIKPLEARAAFHRDKVAALRAAAATEHEAAASQQHRLAKAERQLDANLGTVRATLDTLVLRAPAAGRLTNFRLRPGQPVAAGDTLGQVDGDDGYKLYALVDEAHLGRVAAGQAARARIAGAAVPLVVARVDPQVAEGRFKIELHFRGPTPATLRRGQRVRAELALAPAETG

>tr|A0A2P8D480|A0A2P8D480_9BACT Threonine/homoserine/homoserine lactone efflux protein OS=Taibaiella chishuiensis OX=1434707 GN=B0I18_104110 PE=4 SV=1

MNFSEAVIKGVLLGLFMAISVGPTLFAVIRYSMHHSYKAGIAFIFGVSFSDIIYVTLANIATNWLNFLEAHQKTVGYIGSVLFIGMGLLSLLRKYKPKRPSQGKALDISAGAYFKIWGTGFLMNALNPAVILLWVGSAISVAGAALPPRIVFFGVCLGIVLGFDILKVFLADKIRRRLTLRRIMYLNRISAVCILVFGFILLAKVYFNIELSH

>tr|A0A2S6HQ54|A0A2S6HQ54_9BACE Threonine/homoserine/homoserine lactone efflux protein OS=Bacteroides xylanolyticus OX=384636 GN=BXY41_109192 PE=4 SV=1

MILKGLRFGMLLQLAVGPICLMVFHTSTTYGVIYGLHLVLAIALVDTLYIALSCVGVAAIIKKGKINEVIKVIGCLVLVLFGANTIAGVFNLSFMPHIPLFSNVSGKNLFVQGLLLTASNPLTIVFWSGMFSTQMVENQWNKKQLFFFASGCIMATVIFLTAVAFVGSALGGFLPQIIMQVMNVGVGIVVVFFGIKPLLIYLRHLT

>tr|A0A2S6S8F6|A0A2S6S8F6_9PROT Cysteine/O-acetylserine efflux protein OS=Alphaproteobacteria bacterium MarineAlpha5_Bin12 OX=2013087 GN=eamB_1 PE=4 SV=1

MIDYIPQLIIYVFIAAITPGPNNTIAFYTSYNFGIKNSLHIPIAATIGVSLIQLLCCIGLGSILLKFPIIQSILKVFGCIYLIYLAYQISKFKISKNQQNVKKINFFECFLFQFMNPKLYVFASTTSVIFTNYNYNFLLETFAIVSIMGGMTIIAISIWIFLGNFLLKLFNNDVQRKIINYTLSLFLLATAIWIFTS

>tr|A0A2S9XIX3|A0A2S9XIX3_9DELT Magnesium and cobalt efflux protein CorC OS=Enhygromyxa salina OX=215803 GN=corC_2 PE=4 SV=1

MLAKLIWTAVFVFLNGIFVAAEFALVKTRPARMQALAEQGDARAKRLLAMIDELDLYLSACQLGITIASLVLGYLAEPAFAALIELGAESVGIDTHGSTTLHVVSFGLALTIVTLLHMVLGEQWPKIWAIHTAERTSLRLSLPLKIFTMMFKPLIIVVNVLSNGLLRLVGVSGGHGEHNADVRELKGIIGAAASAGNISARQRIFAENILDLVELEVRHVMLPRTSVAFLDLSAPTKDNLERLRSLGHSRWPLCNKNLDEVVGIVLARDVLDTLLAGGEVELEAIARPTQFVPDTQPLSRFIVGSQQTGHQGAIVQDEHGTTVGMVFLEDALEEIVGPLHDERDELQEPFEKGEDGVIDMDGALDLPAASALLGVELEDSHDTIGGYIIATLGRLPRQGDKLVVGAFDAEVTRVGRRRSVARVCFTPREDAES

>tr|A0A2T0QLI2|A0A2T0QLI2_9BURK Cu/Ag efflux protein CusF OS=Paraburkholderia sp. BL25I1N1 OX=1938804 GN=B0G73_110223 PE=4 SV=1

MVDVDGRGLAVWTVFNVGACEMKNWLASMAMGCMVMISASAYAAGEASESQMSASADAQTAMSHGEVRKVDAAAGKLTIKHGPLENLGMDAMTMVFKVKDPAMLAQVKPGDTIDFVADEVAGVLTVVKLEKR

>tr|A0A2T1AK18|A0A2T1AK18_9RHOB Threonine/homoserine efflux transporter RhtA OS=Epibacterium scottomollicae OX=483013 GN=CLV89_103218 PE=4 SV=1

MSTSSAAQSLSDTDRPLLGIALMLGFCALIPLGDAVAKILSTRIPVGQIVLARFAAQGLILAPVALMLGISLRLPSRVMPKVILRTLLQMAGITAMFMALRYLPLADAVAIAFVMPFIMLLLGKYVLKEEVGLRRLLACVVGFAGTLLVIQPSFAEVGLNALWPLAVAVIFSVFMMVTRTLARDTDPIAIQAVSGGIACLLMAGFFALGYGFSIEELNTTLPATPELKLLALAGVLGTIAHLLMTWSLRYAPTSTLASMQYLEIPVAVFVGWLVFSELPNSIAACGIALTVAAGLYAVLRERQVNRAAREVNPAATTESGLPASPE

>tr|A0A2T4ZPP7|A0A2T4ZPP7_9RHOB Threonine/homoserine/homoserine lactone efflux protein OS=Oceanicaulis sp. PT13A OX=2135616 GN=C7969_0950 PE=4 SV=1

MSLITLAAFAGAIFILFLTPGPGNIAMVGRTLDAGPSHGVTYGLGILTGDVFWLTLAVFGLAAAADAASEYAEFFWIAKIIGAGILLWFAWGAFQGWRHPAPAHAPMAKISKRGLAMTYAAGVAMPLTNPKPIIFYLTFLPAFFDLTTVGPLSYLAMIGVMGAMFLLFALVYVGLAHKARGWLREKGVKRWADLVTAVIMTAVAVLLLTR

>tr|A0A2T5X788|A0A2T5X788_9MICO Threonine/homoserine efflux transporter RhtA OS=Microbacteriaceae bacterium MWH-Ta3 OX=207608 GN=C8A06_0667 PE=4 SV=1

MSRKGLFYFLATGVVWGIPYYFIAIANQAFSTVSIVWLRVVMGAIILIPIAIKRGVLIQAFRQWRWVLVFAVLEMVFPWWFITEAERSISTSFVGLMMTTIPFISALIMGILGEKAAWHPLTILGLVLGFTGVVSLIGIDALSGHIEVLPVLMLAGAALGYAIAPIIAAQKMAHTSTLAVIALSMVIVAVIYTPAVVVQLPVDIAAGITAEQWWAVIILGVVCSALAFVLYFELFKLIGPRRGSLITYVNLLVASILGIWLLNEPITPGIIVGFPLVVAGSYLAGKLHKPWSRKGQAEPAN

>tr|A0A2T5ZVU0|A0A2T5ZVU0_9ACTN Threonine/homoserine/homoserine lactone efflux protein OS=Nocardioides sp. CF167 OX=2135685 GN=C8K06_12351 PE=4 SV=1

MTVLTALAFTAAAAVIVVVPGPDQALLLQLSAAAGRPAAVRAAAGILLGIALWGVASIAGLSAVLVDGSAAFRVVTVLSAAYLAWLGVRALHAARWPALPEQEVVNGSSGRFFLRGLLMNCLNPKIGLFYLSILPQFVPSSATSTVGAELVLSAIYLAVSALWLFGFAFVAARLHPLLVRPQVRRPLELMVGLIFLVLGIAALVSL

>tr|A0A2U1DG33|A0A2U1DG33_PANAN Threonine/homoserine/homoserine lactone efflux protein OS=Pantoea ananas OX=553 GN=C7427_10298 PE=4 SV=1

MGVCWLYRQKTACYSERMFTGDTLMTASLLSFLLAIIILTLTPGFDTALILRSAAAQGWQRASATALGVATGCLLWGIAVGLGLGALLLASEMAYNLLKWAGAAYLLYLGIKLLWHPRAQSVSMHAEAAAQQRHLACFTRGLLGNLLNPKVGVFYVTFLPQFIPQGASVPIWCSLMALTHMLVGLAWSTVLIGSSHYFAEQLKRPRIVKIMDRLTGCVFIGFAAKLALSRR

>tr|A0A2U3KC48|A0A2U3KC48_9FIRM Putative efflux system component YhbJ OS=Candidatus Desulfosporosinus infrequens OX=2043169 GN=SBF1_1790002 PE=4 SV=1

MAEADSKKKKLPRALIGGIVALLIAVGAGYYLYMMRYVSTDDAQISVAEGNSVPITVAFPGRLSTWKVNLNDDVNQGEVIGTESNQSVLSANPLLLPMVTADQLLAGRLIEMENIRSPISGKVLQTNAAAGQGVQPGQVLAVIANANQLQVTANIQETDISKIRVGQIVDLDLDGLPGQQLHGLVSRIDDVTESVFSIVPNVTAASGSYTNVEQRVPVIIQITDKNLAKKTLVPGMSAHVQIHVQ

>tr|A0A2V2ZP82|A0A2V2ZP82_9BACI Potassium efflux system protein OS=Bacillus oceanisediminis OX=665099 GN=DFO73_11427 PE=4 SV=1

MEELNVSILLIKNVTIQEAALFLFFTGVILAAKWMINTVLNKTGKKKSIQNERILQGVASLVNWAAFYGIIILFLFYFSKEKWLSYILFTAGEVDVTLKLLIVAFLTVSLAHRLVQVLTKYLLTSVYEFYGVDRGLGYSFNRMVYYTVMIAALGISLTTVGLDLSAAAAILGVLGIGIGFGMRNVAGNFISGIIILFERPVEIGEMIEINNKIGKIESIRLRSTIIRTAKEGTLIVPNQYFIEQIIKNRTGSEMLAQVLISVAYGTDTEKVEELLHEVVVREIPNADGVLLKPPPDIRFVDFRNKAMDFLIEVPVAHFEAKQNFESRLRHGIAETFYKNGIELASPQGYPAD

>tr|A0A2V3VLX0|A0A2V3VLX0_9SPHN O-acetylserine/cysteine efflux transporter OS=Blastomonas natatoria OX=34015 GN=C7451_10483 PE=4 SV=1

MQPDSQLQDLPDRGFNRLDWAVAVMMMLLWGFNIIAMKSAVDSAGALPGAFLRQAIVAVVCLPFLRIVPGRMKLICTLGVLSGGLFYVAVGLSLKVTNNVSALAIAGQLGVPFSLILAVIFLKEKIGIPRLIGIVLALFGVLLLVFDPAAGKELPAIAISAVGSLIWATATLIQRNLGGIGVLNISGWLGLIGSLVILPFALLLEPDGMALLPHLSAETYAWTAYAAIGSSIGGHGAMVWLLQRHSVSTVSPLTIPTPVISVAFATWWFGTPLTWLMIAGGLIALLGVSIVAVRNAQKARELAEARLMKEGTRS

>tr|A0A2V4W4W2|A0A2V4W4W2_9GAMM Threonine/homoserine efflux transporter RhtA OS=Shewanella chilikensis OX=558541 GN=C8J23_11131 PE=4 SV=1

MSAKGQARTGLTELHLAVLLFGGTALFSRLIPLSALNITLLRCVIAAIVLALLVKLSRQRLRLLRGKDYLIALLLGVIVSLHWVTYFAAMQLSSVAIGMIAFFTYPVMTVLVEPWFTGSRLHLRDLVSGLAVLLGVILLIPEPSLGNDVTLGILVGIISAILFTARNLLHKRYFTAYSGQQAMFYQTAVAVLVLAPWHSLDAGDISNQTWGLLLLLGVVFTAAPHALFTSALRYLSAKTVGLVSCLQPFYGAMLAWLLLDESLMLTTAIGGTLVVATALFETSQSHKSQAPKKNLG

>tr|A0A2V5EIA3|A0A2V5EIA3_9BURK Threonine/homoserine efflux transporter RhtA OS=Acidovorax sp. OV235 OX=2135489 GN=C7505_12242 PE=4 SV=1

MNDVATQGLSSRRLFLLVACLTAVWGTNWVLFPIAVREVSVWTFRAICLLGSGALVLLIARLQGMQLAVPRGEWRPLVTAGLTYLVIWNVASTYAAVLLPSGQAAILGFTMPVWATVLSWIFLKQRPSARLLTSVVLASCGVGLLAYAAREAFSSAPLGFLVGLTAGLGWAAGTLILKRANITTPAMVSTGWQLVIAGVPIACVALLNGSHQLFMPSVATILVIGYITIIPMALGNLAWFSIVKVLPASVSGLSTVMVPIVAMLTGAVVRGEPLGTLEITAMCFCASAMAIVLLKRTE

>tr|A0A2W7NGJ0|A0A2W7NGJ0_9BURK Threonine/homoserine/homoserine lactone efflux protein OS=Paraburkholderia tropica OX=92647 GN=C7399_10389 PE=4 SV=1

MSLSTWFKPAFIAGFFLLRPRNRSMTGQMPARDRSLWTRAHWHNRRFAYFFLTSAVAMNLHTWWLFLATVFVVCAIPGPNMLLIMSHGAQYGLRRTSATMGGCLSALVLMLAVSAAGLGVFLQAWPTMFNVLRFIGAAYLVYLGVKAWGAPVEEHAASNAEAETLAARPARSPATLFRNGFLVASSNPKAILFAAALLPQFIDASRPTLPQFGVLVATFAVCEVSWYLVYAGFGTRIGATLKSRRVAKAFNRVTGGVFVGFGAMMALMRQ

>tr|A0A2X1BPW2|A0A2X1BPW2_BREVE p-hydroxybenzoic acid efflux subunit AaeB OS=Brevundimonas vesicularis OX=41276 GN=NCTC11166_01125 PE=4 SV=1

MARALSPRRAAEVRAALQMAVGAMAALYLATWLNLPHPYWSVISAIVVIQASVGGGVLTVARDRAIGTATGALAGAVFAFIRPPGLESMALSIAISAGLLAFFATGRPWLKVAPVTATIVIAGGTGAEGPASLALDRVMEILVGSGVGVLAILALFPRHAGQSFKLQAREAAGEAAGLLALVSKAAPEDAAEISRRHADLKRRLDALGQAAKNVIDLPGPQRETADRAALVRAFWRVRSDIVILGRGFQAEGAGARLDPWSQDAERAVEQLRALSEGRAAQPMGAIDQSLALSMAVEGDDVALGAAAIGVAHMHRDLDDLAARFADLKLV

>tr|A0A2X2IQ09|A0A2X2IQ09_SPHMU Arabinose efflux permease OS=Sphingobacterium multivorum OX=28454 GN=NCTC11343_00842 PE=4 SV=1

MMMPFGSAFAVNNLKVSHEQLPMLFMISGIASLIIMPIVGKLSDRYDKFKIFAFASLWLMVVVFIYTNLGVTPFYIVVILNILMMAGILSRMTPSSALITAVPEMKDRGAFMSISSSLQQLAGGVAASLAGVIIVQETKESPLEHYPTLGMSVILLSIIGIFMIYRVSSMIKKRKANS

>tr|A0A2X3B7J9|A0A2X3B7J9_9CLOT Arabinose efflux permease OS=Clostridium paraputrificum OX=29363 GN=NCTC11861_02027 PE=4 SV=1

MAALSLTAAAVLAGTSARSTARVGAGGRRGASDGMDAPAAPRPGRLPLATVLRNRGAAALIGISLLRTAGFMGALAVVAAVYAERHGLTGAGFTLVWTVSGAPFFAGNWFSGRLLSVRDADAALMAGGIVASLAGVALVFAASPFTLMVTGTAVLAVGHAMIAAGVTTGLARLADPARSAALAVNGVAQAAGTVAGAALAGAGYALAGWPGVAWALAAVTVPCAALLVMTRAGRN

>tr|A0A2X3K0F8|A0A2X3K0F8_9FUSO AEC family transporter OS=Fusobacterium necrophorum subsp. necrophorum OX=143388 GN=EO219_06115 PE=4 SV=1

MWIIIQKMLLLLCVSALGYWICKAKLITLEHNRGYSILISNVTVPCMVIFSIFSQPPIQNYGEIFSIFGVGFLFFGFFTLCSLFLPILFRAKNEEIGIYRFMTVFNNNSFMGFPIIQSVFGNKYLFYAAILNIVNALYLYTYGMHCITKDVEDYHFDWKKLCNPGMVVSVISLALYLLHFSLPEFFLEISRQVGNITTPLSMLVIGVNLSMIPFREVFSETKLYLFSFFRLLVFPLILWFLLKGFMANTDFLIVVLVTAAMPGAAMMVNLATEYKGNVYFASKYLVLSTLLSVIIVPVVIYVLQNYV

>tr|A0A2X4NAE1|A0A2X4NAE1_9BACL Threonine and homoserine efflux system OS=Gemella morbillorum OX=29391 GN=NCTC11323_00051 PE=4 SV=1

MRRFVSELILIGVVIIWGLAFIWQNIASKVLGPLTVVGLRSLIAVIFITLVAILVPSLYKSQAPKLIGEASSSKKLWLGIMCGVVLFFAMYIQQIGIGMTTAGKAGFITVLYICIVPFIGVFLGNKLNKFFIIGLILAVIGFYLLSVKEEFTLELGDVIVFISAIFFGVHIIVIDYSALRVNSMFLSIIQLVVVAIFSLGLAMIKETIILADILGVAAPLLALGILSSGLGYTGQIIAQREIPPHTTSLIMSLESVVAAIGGVLILNEHIGLREGIGMAIVLVGIIISQLREKKSPKLEQK

>tr|A0A2Z6IGJ1|A0A2Z6IGJ1_9PROT Cation efflux system protein CusC OS=Acidithiobacillus ferridurans OX=1232575 GN=AFERRID_06470 PE=4 SV=1

MRKRTLPWRIPARHPGSTRWVLACWMMIGILPGACAGSLTQNDADGAGLGSALMNALPASPETVAPGLLPPAQAQARRAVKKRSPVGSTPTPPAPIRTPAALSHVVPSTTVALALSSGHGVTKVRNSAADAGTHPADHAAAVTIMAPRASAPALPDLHATAHSSAAPVGAVAAGGGSKLHELLATALANNSDIRLADQSLREAQAESLDAFGQFLPHLNFQAQTQLYGNQTNHPAVSLIGSTIVVTQGNNYSNYLSVMASLNLFEGGQGIANLAASRQGVHAGHEQVSERRRRTALNVLAGYEELQSLQWQLRAIQRSLVFMRQDLALAEQRMRQGNESRIDLNQMRSQVANLEAQRQDTKKRLVKAQTNLALLTGKTGAFDLLQSGVHDSIPTPPEFDVATVEGAAVENLPSVQVARAALRKARDRVDAVRGSFLPNVNLQTGYNWIGTSSQGFGRAIGSTSPSNYTVGISITQTLAPFTGHMAKLDTAEARSEAALIRYQRALQEGRQELRVNQEEVRAGTARLAALESIYARARQNQKLMEELYAHGRISKTDRHAAQIKTMNAEDACRDARAQLQVARWMLYAMVDPRHVADSLLQKTQAGEADNAAPSSSGAGAEHA

>tr|A0A315ZCA4|A0A315ZCA4_9BACT Threonine/homoserine/homoserine lactone efflux protein OS=Sediminitomix flava OX=379075 GN=BC781_102754 PE=4 SV=1

MNQALLLPFLSYAIVTTITPGPNNITATSAGIQLSYKKTVPYLLGISVGFFVIMLIAGSFTSYFTSQNDNLFSVIKWFGAVYILYLAFIPFINVKNKKNKSLNKNYSFITGFGLQLINPKVILYGVTIYSSFTQLIGGSNIRVVSSAIFLSALAFVCTSIWAILGTTLSSYFENKTFSLVFNSILALLLIYIATTIILI

>tr|A0A316AT94|A0A316AT94_9ACTN Arsenite efflux ATP-binding protein ArsA OS=Quadrisphaera granulorum OX=317664 GN=BXY45_113116 PE=4 SV=1

MSTAWDVDALLDDPSVEVIVCAGSGGVGKTTTAAAIALRAAERGRRVAVLTVDPARRLAQALGTDGTPGDAADDAAEAGAEAGADEGDAPGPDEPRGVPGVDTRRGGSLDALVLDARRTLDGLVDAALPPARAAQVKANPVYVSLATSFSGTQEYMAMERLGQLRAGRDTAAGWDLVVVDTPPSRSALDFLDAPTRLASFLDGRFARLLMAPVRFAGSARTSTGLRAAGGRVVGALAGGVAAVMDRVLGGRLLRDVQALVEGLDEVFGGFRERAEVTSAALRDGRTAFVVVTAPEAEPLAEAVFLTERLRSAQLRPAAVVVNRAARAATGLDAATARQAAEALRTGTTGGAARGGKTSAAAEKAARVLDLQVELLERVERERRLVAERLADSGAPTALVPALDGDVADLDGLRAVGDALGRNR

>tr|A0A316CHN7|A0A316CHN7_9THEM Threonine/homoserine efflux transporter RhtA OS=Oceanotoga teriensis OX=515440 GN=C7380_11555 PE=4 SV=1

MVSSQLKGWVYLLITVFFFSTIEVVTKPFAGVFDPLQITFLRFFFGGLVLLIFLLISGKMKTYKLTVKSLVLMGLIGSLNSVLSMSLLQLSVKFSNASTAAILISANPIFVVILASLILKEKITLRKVVSMAVGALGIIIILMSNSSGDSTLGLLYGVLATISFALYTVLVKKYVKEIPSIIFVTFSFLLSSIIFYIVLLIFKIPVFTFEIESFNVIWMLAYISIFVTGIAYITFFKAFEVLDASKGSFSYLLKPVISMIMAYLFLNEIPNNMKLIGTVFIILSVAIIALKGKKNKI

>tr|A0A318J4V9|A0A318J4V9_9BURK Threonine/homoserine/homoserine lactone efflux protein OS=Undibacterium pigrum OX=401470 GN=DFR42_105357 PE=4 SV=1

MPELANLLSFILAACFVIIVPGPATLLVAELASLSVQGAAIAVAGIVMGDIVLIALSAAGFAVLMQSLPWLLPGLRMLGAAYLLYLGINLLRSAGTMTQLQPRPASVSFARGLLITISNPKPILFFSTFFPLYLSPAHDAAVQGFVTLGAMFEVINVLYFILLCSILRWTAKRLAHSKGRQGWLQKAGVHKICCVGLILCSLAMAWNF

>tr|A0A318TYH1|A0A318TYH1_9BACI Tetracycline resistance efflux pump OS=Lysinibacillus chungkukjangi OX=1202712 GN=BJ095_101161 PE=4 SV=1

MEGTIFSLIPPIVTIALIIITKRLFLSLGVGIVLGALLYNQWHIFDSVSNIFNIGVEVLVGDGKILIFVLLIGILSSLLYLSGGINAFSQWGTKVAKTRAQSQMATIFLGFFTWFDDAFSCLFRGTVMRSVTDKYNVSHSKLSYLIHSSSAPVALLIPVSALSAFIISIIDGVLKSNNIAEYQALEAFLLAIPSNFYTITTIVLVIIVAYTGINLGQMQRDEKRAIEKNILFDTKHGKIPGADESKLPSRNDGKMMDLLLPVLALILVTIITAVTIGSAGEGSNSPIELLKNTDIITSLLYGGICANIIILVRLIIKKTSAGHLSSTIFSGIKTTLPSVTILFLALVTAQIISSLGVGQYLASLIQGNMSIAWLPVIFFVFAAFISYSIGSTLGTAGIMIPIGAEIVATIDITFLIAIIGAVLAGTVFGEHSSPLSDTTILASIGSSVHPIDHMMTQLPYAVLSSIASIIGFLVLGFTNSILIGLGVSLVAVVIGIFFLKSRQAKLKST

>tr|A0A327TDZ3|A0A327TDZ3_9ACTN Threonine/homoserine/homoserine lactone efflux protein OS=Kitasatospora sp. SolWspMP-SS2h OX=1305729 GN=K353_04281 PE=4 SV=1

MTSSAFAVAASPAFLASCAAVVCSPGPDSLLVLRLVSRARHRRPVLAAAGGMLLAGAGYAALAVTGSMAVTALDPRLFLLWRAVGALVLAVTGARALWEAVRPGPPGPGAPEPGPARDRVGRHLLLGFLCTAGNPKVGLFLTVFLPQFLPADAASASALPLLAAVYLSIGALWLLVLTEVGVRVVTARGADGRAAFPPLAGRIGGGVVGAVLLLLAASLLFR

>tr|A0A327WX07|A0A327WX07_9GAMM Putative Co/Zn/Cd cation transporter (Cation efflux family) OS=Aliidiomarina maris OX=531312 GN=B0I24_10884 PE=4 SV=1

MLRSFSSEDRLILVSALMALGFAVGGIAIGTWLQSSIIIFDGLFSFISLGLSLISLVAGRYVRSVNESKYPFGKSIIQPITLVFKYLAIFILCTLSLFEGIQTLLGGGRALNFEQAFVYSAVVTLLCLLAFKFLQRRTQPEHSDLLVAEQREWLMDTGLSLMLTLGFALAIVGQWLGFDRFALMVDPLMLIIAALFFMRIPTRGILDAGKEVLGLKVAEALEADVRVHVEDIVLSYDFKDYYLRLQKVGSTVYLEVDFIVHANQVSLSILEQDKIRSALYKQIRVFPYRWWYTISFTADEQWAR

>tr|A0A328X155|A0A328X155_9BURK Cation efflux family protein OS=Paraburkholderia unamae OX=219649 GN=C7401_13360 PE=4 SV=1

MQVTCVAVLVNVVLMAMQFVTGWISGSDALLADGAHTLVDALGDGVVAGAIYFDQAMRAGRPHKLTPVAIVLANLLIAATGAELLSAGMLPNAVQGAGARPAAALAAFAVSVASVAAKAGLFLYLRAAAARVKPHDGDSLASALNAGAWHACADSVSSVVAAIGATGVLLGLPALDRSATALIGALILMAGLQRNASALRSLFRRLARMGRTRRAHAHAGQPEPTSSQA

>tr|A0A330PD81|A0A330PD81_9BACT Arabinose efflux permease family protein OS=Mesotoga infera OX=1236046 GN=MESINF_1359 PE=4 SV=1

MKDLGRNFWLYAIGRLVSLIGSGVQSLAIPLYILDLTGSGTIMGTFMVITMLPRILFGPIAGVLGDRFNRKMIMIYMDFARGAAILAMAALAGANSLTITVLFIFQLLISTFDISFDPATAAMLPDIIDSDKLLRGNSILGAINSLSYIIGPVLGGILYGMFGIEAVLILNGASYIASAISEIFIRYQQTTEKGKISLKSVFKDIVEGVGYMRKINGLILVMVFAMLSNFLLSPFFSVVFPFFARTIVGFTSEQYGFLQSGWVVGVLIGNVILGTLLSKKRQGNLFAMGLTAETLILFLLTVFFFPYFIDLFGWASWRYFAALGLPILVTGIFNAFVNTPLNTLFQKIVPTNYRSRIFSVISILTQIATPLGAAIYGFAVDRVPVHYLILVSSICNALLTLVFLLKGMTKLFDGKTPDSERGLVEKSVEAVKEGASL

>tr|A0A335EIV4|A0A335EIV4_ACIBA Glutathione-regulated potassium-efflux system protein OS=Acinetobacter baumannii OX=470 GN=SAMEA104305308_05530 PE=4 SV=1

MTHEFPLAKLLVRSYDREHSLHLVKQKVDYMIRETFESAIKFGGVILQELGVDEDEVERITEEIRDLDNERFETEIAADDVYAGADMQYTHAHHPRPTAPLIRPKQEGRILNKDDASDNENMDG

>tr|A0A344PLI0|A0A344PLI0_9RHOB HlyD family efflux transporter periplasmic adaptor subunit OS=Paracoccus sp. SC2-6 OX=2259340 GN=DRW48_11485 PE=4 SV=1

MTAPDTPDLPLDLGRDTGDAAAPARRRRWFRKRYLLVLLIPVFMFSGAVIGLYYQPPGLQNFYALTGLQPGGGADNPIALPPEIDLPEEMAETLLPSDVVGLARLMPRGDVAIVAAPYGAGDARVAEILVSVGDRVSRGDMLARLDNMQALESAVLTAEATLAVRQATLAQTRSAVAASRAEAQATLDQARATAREAQANLARTEGLAERGVATEATLDAARTAAEEAGLAVVRAEATLARFTGTALDDQPDVVVAARNVDAAQAELARARADMAQAEVRAPVDGTILEVNATPGQRPPAEGIMEMGDTSAMMAEVEVWQDRVAAVAPGQPVELAAPALGQGVRGTVESIGLTVGRQGLISDDAAANSDARVIRVLVALDPASSRLAARYVGLEAVARIDTGAPARVGQ

>tr|A0A346NMC3|A0A346NMC3_9ALTE Chromate efflux transporter OS=Salinimonas sp. N102 OX=2303538 GN=chrA PE=4 SV=1

MHEPSTSSAPPSSGSNQITRDATAGNLNAFGHYITFGEAVRVWLRVAILSFGGPAGQIAVMHRILVDEKQWVSESRFLHALNYCMVLPGPEAQQLATYIGWLLHGIKGGLVAGALFVLPGFLSILVLSLLYAGYQEASLVQALFFGIKAAVLAIVIQAVIRIGKRVLKNAYMYALAVAAFVAIFFFAVPFPIVIVTAGLIGLLGRHVDPERFVVIKGHDTPEDAGRAIDAMMEGGAASHTRASRGRALKVLAVWLPLWFAPLVALLVTLGYEDVFTQIGLFFSKLAVVTFGGAYSVLAYMAQEAVQNYGWLAPGEMLDGLGMAETTPGPLIQVVQFVGFMGAFRAPGTLDPFTAGILASVLATWVTFVPCFLWIFLGAPYVETLRGNQAVSAALSAVTAAVVGVMLNLAIWFAVHVAFNEVETVRAYGMLLLIPAWGSIHIVSVALAAGAFIAMLRFKVGMLPMILVSALLGIVYHLLFAGAPAA

>tr|A0A366EKV6|A0A366EKV6_9BACI Threonine/homoserine efflux transporter RhtA OS=Bacillus aquimaris OX=189382 GN=DET59_11161 PE=4 SV=1

MKEKVNGKPVYLYMGLLFCVICWGSNFIFGAILVHYFKPMEIAFLRLIFITLFLLIVFYKSIRQFSSLKSMVIPLLFIGFIGVTLNHWSFYASLTTASPVTAALILATAPICTSLINSVVFKERKSPFFWMWSLFSFFGVLLVIMKKGAIVIGMGEGYIFLTMLTFSVFMILVERYARHLSSILLTFYSTMVGLILMTVFLPFSDVTFLRSVPFSIWMLLFFTAIIMHGICPLIWNHCISEIGSTNTSLLLNIEPFVAMVVGYIVLKESVSTMQMIGAITILISVTMALHSNRMGQRDRHLVPTNSNTL

>tr|A0A367PMV5|A0A367PMV5_CUPNE HlyD family efflux transporter periplasmic adaptor subunit OS=Cupriavidus necator OX=106590 GN=DDK22_09775 PE=4 SV=1

MKDDARQGGLKPLSEALEDHSAEGIGLLSAEPSRLGLLTIVTTFALVLCGLVWSFVGHADVIVTAQGTLAPESEVRRFYAPVDGELADLYVAEGQPVSKDDVLARLNARGAIEAAANALEAQLKLEDSEREWKQFPDKKALMERRAAALKQQIDVATRQHETRIAEGTTRLAEQQRAQLQEARSNLENARRAREFARQEQDRYARLLALPGGGGVSQSQVDAKRAAAQDAENNLRVAQSRLAELDARLGRELTQASSQLESSGQDLAGLRVQYDAALREIANTEDKLRLQVQTARLVADAAARIRFENIDKDNFLLILAPVSGVITDVTSTQRGDKVQANTPLGGIAPKDARPVVKIVIAERDRAFLREGLPVKLKFSAFPYQRYGIIEGTLEFISPATKPGGPDKQPVYEGRVRLARDYYAVADNKYPLRYGMTATAEIVVRERRLIDLGLDPFREVAG

>tr|A0A369ACI5|A0A369ACI5_9BURK Threonine/homoserine/homoserine lactone efflux protein OS=Extensimonas vulgaris OX=1031594 GN=DFR45_1193 PE=4 SV=1

MNATEFTTLLLFCAAMTFSPGPNTTLSTALAANLGLRRALRFCLAVPTGWSLILLASGLGLATLIARVPALRWAITLLGVAYMLWLAYRLSTSARLAQADEARPGACRTLGVESENAPQRGQFLPDSPPHSGAMGQEAGKKWAAGAHSQPTIPKPDRLLGLGFWQGVALQFVNIKAWMLALTLSAGWVVNTAEQSAAIPAERLAIVSAVMLAFAFASNFTYALLGVLLRRWLAQGQRLLWFNRALALVLVATAVWMLRL

>tr|A0A369XW18|A0A369XW18_9FUSO HlyD family efflux transporter periplasmic adaptor subunit OS=Psychrilyobacter sp. S5 OX=2283384 GN=DV867_13815 PE=4 SV=1

MSKKMMTIKEYELTTDYFFRKELRLMIFYIYFLTSLIILLLTWSYFFNIDILVKSRGVVRPIKKISSILNQFEGNLTKVNYQDGKKVKKDDLLYSIDTFILENNYLKNSDLLRKEEKEIYFLATLKESLVAKKSFFKDKDNEYYYLYQKQKYKDKRLEAILRAAKMEYLKYKALGSDYVSEMDLEKYRRAYEEALYNYKMSDAELLSEINNKIFTLKEKTENIIKENIDLKNQIEKGSVKAPITGTIQCSKVFNKGDYIPKDQKVLDIVPQGSKLKMIVDIANKDISKIKVGQLIKYRIDSLLYKEYGISKGKVVKISPDSSNKGSFRMEGTIDREILENNQGDRESLKIGMTSDIRIVTTQKSILRVILEKLNFMNE

>tr|A0A370F3S2|A0A370F3S2_9NOCA Threonine/homoserine efflux transporter RhtA OS=Rhodococcus sp. AG1013 OX=2183996 GN=DEU38_12462 PE=4 SV=1

MSRGPGAAVNAGSRSLGTGAWAAGLASAAAYGLTPVVAVLAYRDGVSPSVLVTLRGLCGSAVLLLIAAGTGRLRGISRRPAAALLFLCGPLFGVQILAYFAAVQATGAQVALALVHIYPLFVLLLVCLARRQRVNVWTVALCVPMVCGIGLVAGGGAASSSITVIGVGAALLSASGYAVYLVLGEEWGRSVGVVNAALLVTVGATITTGIVAVATRQSFAVPQGVWNVAVVQGLLLNPVGIGCAFYAMRRLGSVAMSMIGLLEPIFGIVSAALVLGEHLDPVQWLGVGFILSLGGLLPWTMSALRRRPVASSGAVQAHQEVNRAGVVDGHESQ

>tr|A0A374DUW3|A0A374DUW3_9FIRM HlyD family efflux transporter periplasmic adaptor subunit OS=Ruminococcus sp. AM36-2AA OX=2293210 GN=DW846_02595 PE=4 SV=1

MQDEISQLDESIQEYQDSDEENVIESSVSGRVKKINVSAGSDLSDIMVSDGALMVLSLDGKMAVSLSGVSGVSAGDSVTVTLSSGTQVTGTVDSASGEDCVVTLTDNGTTYGDTVIVTDSSGQELGSGELTIHEPLEITGTSGTVSAVNVSENASVSEGTTLLTLEGSANETQYQELLAKREARTATLKKLIQLKADPEIKAEISGTVQSVNVSAGSSTTTDSSSGSSSGSSSSGSSGSGKTVSQMSYVVSGSDTTAGNAVQLISLGSTTSIAAIEANVNASVVRCSDTGAAVSSDSVTNIADSNLNSQETGETEEIISLQTDTEQQAEAVALASSDTDFSSDVGGEGDSSGENTSETSTTLQFAIATEGTSTASSLVIAAPVTGQTPVTSVSATDGSYTGTVAWNPGDDSFQEKTVYQAVVTLTAGDGYVFQAGSVSGITLGTVSGICVSQDGKSMSFQITFPETAAETEDIKKDDSGDGKTSDSTDNGKNADDNKDNDADQITDRAAGQTGNNSGQNGTGSTSSNGKDSSDNGTNSTSETNGNSTQSGNDQSGNGAGTSANNISGSSSGASQTEDTQETDSSASTSGTELSTSEYSTDVALFTISPDDTMTLEVSVDELDINSVEIGQEAAVTFDAIEDKEFTGEVTEIGNTASVNGGVAKYTVSVSVPKDEEMKQGMNASATITIENRENVITIPVNALQEKGNKVFVYTEKDEDGNLSGETEVTTGLSDGTTVEITEGLSEGDTVYYNKSGNTDSGSGNDSGMPDGMGDFGDMSGGPGGNSDSGNSGGPGGNGGGPGGSGSSGGNGGGTPPNM

>tr|A0A375AJ90|A0A375AJ90_9SPIO Threonine/homoserine/homoserine lactone efflux protein OS=Sphaerochaeta dissipatitropha OX=1945881 GN=SAMN06298221_10394 PE=4 SV=1

MQIDMLATLTFALITTFTPGPNTISSQAMGLNYGYRRSLPYFFGIATGFFSIMLLSAVLAAALTQLIPSVVTYLTIAGSLYILYLAYHVFTSSYSFSQTIVKPLGYSNGLLLQLLNPKVIILGLTVYSTFLRDMARTPFNLAASALCFTLMSFSALSTWALFGMGISRLLRTEHTRKVVNAALALLLVYTAVRMVWSLFSA

>tr|A0A376BGI6|A0A376BGI6_ALCFA Copper efflux oxidase OS=Alcaligenes faecalis subsp. faecalis OX=32001 GN=cueO PE=4 SV=1

MKRRDFFKLTLGASLLSSLPMRSWGQGMMGHHGSMSEGMPGHGMSTRPSLMPIEKMPAGQALQALPVLKNSSQKKGLFQAKITAAAHQRVLADGKSTELWLYNGQAPGPLIELYEGDQVEIEFENQLDQATTIHWHGLPVPSDQDGNPQDAVMPGQSRYYRFTLPEGCAGTYWYHPHPHGKSGEQVAHGLGGTIIVRSPKDPLKDYTEQHWAISDLRLDINGHIPTNTGPDWMNGREGQFVLLNGQRQPLIQASTAERIRVWNSCSARYLKLHIPGARLVQVGTDGGLLEQALAAAESILMAPAERVEFFIQTDKDLDSSLQALYYDRQKMMVQESPETLTLATLKIRHQEIELPKQLRTIPAIPAAASTAKVVFSEVMPMNHDMQSGHGSGMTNSSGMNHQGMAGMDNDMMPGMAAMRSMFRINNQVYDMDRIDLRCPTGQWQYWDVINDSHMDHPFHLHGTQFQVLARQTGMQSVPEAFRAWRDTVNLRPNETVRLAFRQELPGLRMFHCHILEHEDLGMMAQLMVE

>tr|A0A377TD10|A0A377TD10_9GAMM p-hydroxybenzoic acid efflux pump subunit AaeB OS=Ewingella americana OX=41202 GN=aaeB_3 PE=4 SV=1

MKWFSKSAVLFSLKTCFAAFLALYIALALNLDKPAWSIASVFIASQLYSASTLSKSVFRLLGTMLGGLFILLIYPATVQLPLLFSLCVSAWVALCLYLSLHDRTPRSYVFMLAGYSAAIMGFPDVTSPQAITYTVLSRIEEIGVAIVCSSLIHSLILPVSMSNILEKSITDWYDSAKKLCNALLTAPTPEKSPDHENILIQMAGYPANVEVLITHCIFEGNAARKLIRLVTVQYQHLSYLVPTLTSIELRLNMLAQRQIAFPENVQQTFRHFLLWLNNNDKAEDSATIQQNIAQTQTELQQAHQSNAMNVEDSLLLNGLLDRLGDFVRIAEANFSVGKRVDNFDDNKAKRSTAHWHIDKGMLLLSSFTAFLVTFLCCLFWIGSGWKDGATAPMMAAILCSFFAAMDNPVAPMKVFLTGVVVATAISIFYVSMLIPLTTTFEALVICLFPGLFVLGVLIANPATNLLGLIIATQIPGLISLGHHFKPDPLATLNGAISSLVGVLIGVVVTAIIRSKRPSWTARRALLRGIKELIQFLAEIKLHRASLNTRQRFVARMLDKVNVILPRKKNDTTEELASGGDLITEVWLGANYYDFHMKNQELLADHIHATDRIFYELKGYLKARLKSFQASPHPKLLREIDLLLIKLEAQSSKDARYYAPMLSLFNIRLVLFSRSHWPSFE

>tr|A0A378MET9|A0A378MET9_LISGR Macrolide-specific efflux protein macA OS=Listeria grayi OX=1641 GN=macA PE=4 SV=1

MKKWIKWLITIIIIVVVVGGIGLFFAMNKKDDASGTSKLVTTKVKQGDMKINATGTGAISPENQQLPDYDKLQLVAQMDELDIPDIKKDQKVKISVAAIPNKTYTGKVKEIAKQGQVQNGVSSFKVTISLDKKDKLKAGMTADASILVHENKHAIYVPIEAVQKNDDDKYYVYVPKKNKDGKTKQVKKFVETGLHNEDNIEITKGLEKGDTVILPTVDTGNNSDF

>tr|A0A378QSD6|A0A378QSD6_9GAMM Probable amino-acid metabolite efflux pump OS=Moraxella equi OX=60442 GN=eamA PE=4 SV=1

MHTKDYLVLFSVVLIWGVNFLAMKIGLNDVPTLILGMVRFLLILLPAVFFFKKPNAPWIYLILYGLTISFGQFSLMFLALSWHFPTGLSALILQAQVFLTVLFSCILLKESVKPNHLVGMITAGVGLTLIGVGQYQGGFSLIGMLPVLGAAFSWAIGNVIVKKIGQVNPLSLVIWDNISAFMAFTIFSVFNYGVGGVMSHLANFSTLGILSVMFLSYVASCVGYTGWGYLLAQHSASKVTPFIMLVPVIALVVGYVALKERLILWHYVGILTVLFGLGVHLLDGQWFDKKF

>tr|A0A378Y7T8|A0A378Y7T8_9NOCA Potassium efflux system KefA OS=Nocardia otitidiscaviarum OX=1823 GN=kefA PE=4 SV=1

MEDVLRPLIVFGGTLAVSIMAGLLIDRVLRYSANRHPGSSVATLLRRIQLPLQALLASAGLHFTYPLAQLELQQDTVIRNVLATLAILATAWLAMRAADTVAGNTLDKYANRTADTARVRRLHTQLGMVRRIVTTVLVVTTAAVAMLILFPNLRTLGTSLLASAGVIGIIAGVAAQSTLGNLMAGLQIAFGDSVKIGDTVVVEGEWGTVEEITLAFLTVRIWDDRRLTMPISYFNSKPYENWSKGGPQITGTVFLYLDHSTPVPELRQHLHEFLRGRKDWDGRKWNLLVTDSTPTAIVVRASMSARNADDVWDLRCAVREELLGWLARYHPYALPKIPTAMVSGGTPAMAE

>tr|A0A379TGA1|A0A379TGA1_SALER Potassium efflux system KefA protein OS=Salmonella enterica subsp. arizonae OX=59203 GN=NCTC8297_03919 PE=4 SV=1

MRWTLFIFFCLLGAPAHSVAIPSVTTGTSTSQQTATAPEPNTEQKKSGLRSPG

>tr|A0A380C4K7|A0A380C4K7_SPHMU Probable efflux pump outer membrane protein ttgC OS=Sphingobacterium multivorum OX=28454 GN=ttgC PE=4 SV=1

MKRVYIYSTLCFLLSYAKAGAQQTEQRLTERVAGTATIAAVDDKQITKRSLAECIQLAIKANPTLLQNELDVRRAEVNLAQAKANRLPDVDASLQHSLTSGRSQDNSTLQYISSNNSTGNFSLGASLPIFRGFRLFHDIRMRADAKTAGKLTFDTQINALKLDVITAYIQSLTAQDILRQSEMQTEVTREQVRRAESMHKEGAINPGDYFDLKGQLANDVNTIENNRQLLYSSRVKLAALLNMDENQLGELDNLGIKESGQRLDAKQLYEMAVDQLPDIQALDYRIKVAERDIKIAKSYYYPSLSLSAGLGSNYSKLGIQGTYWSQMRNNVGKYISLNLSVPIFNHLQVYNNVRLAKLDLQTARFQKEIQQNVLRSATSNAVFNLQNASNMITQLRSQNENYAESFRIAKVVFELGNSNSVIFLTAKNKFDNSQIQLVVKQYEWLLQKYINDYYAGSLNL

>tr|A0A380KFS5|A0A380KFS5_9STRE Lantibiotic efflux protein OS=Streptococcus hyointestinalis OX=1337 GN=NCTC12224_02243 PE=4 SV=1

MTDNKDAEGVKGEFDRLWSSLSLAVKELTSVPELRLSMIIVPVVNSIFVVLPSLLVLMMSQDRAFMLGNASITLAAVNIAMTVGMIVGSILVMNLLKKMSILSLLRMGTLGVFLMFFSLFLRQPYFLLVFLALTGIATGAINPKYNALIYNRLPEEQLATIEGGLMTYFQLGTVFSRLLVSTLILVLTVNQLVLLFLLAAFFLLLYSLKRVPLMTVDEDNKNI

>tr|A0A380W1C0|A0A380W1C0_ALCFA Potassium efflux system KefA OS=Alcaligenes faecalis subsp. faecalis OX=32001 GN=kefA_2 PE=4 SV=1

MKTLRLGVQVLLLFLALLGAWPQAQAQALSEVEQEREMIRELQQAQDEIRLMQYRLEGNQVSIRPERSYEEDQKLVRTQELSGRVVQSLERRSRLLKARLQELGEDSGDEAEQLIVHNERMQLRRADAALRADLRVARLVQVEAQQARQLLREATVRYEKAKRWRRNASLVLLESLPELAQAWPADRSKLSDFAQQWRGTWSNSSWQQSSASLWMAAAVLLVMLALFRVMPLWVSRYLPAGRLRRSSLIIANFFLWFIATWVVVDQLVELVFQRPGLSPTQFELLAYLKVGAWAAAVALACLKSMVVQPRASWRLIPVSTQTQHRLRYFAPAFFLLVYADITSSFFRGSIGLSDAFQSMADGVSSLLYLILYGYGLWVLRDELFNRTDVAGQGASRTGRSWARLAFKGGVLIYILVLGLFIGGWQGLSDDLMSHFITMPLILGLLAYVCMVWLQDIADSLLTYLRNRSHDPEAAPIRVQSQFIVVFFAIARMSVLLLAIWMVSGDWLTEPKQMLESGLDVSREALRLGAVQWRLDLWLIALGVMLVGAVLIHFLRTWLRQHYMPNTTLEPGLQNAIVGMVGYVAYFVLLVICLSMLGVPIESVTWIFTALTVGLGFGLRGIVQNIASGLMLMVERPVKVGDWVEVEGSEGNVRQIRLRATYVERFDRTMVMVPNSQMMGRQVRNLTYTPTSLGAIESRLLFPLDVDADAVMQILREAVQSEPEILTEPAPILSCDGIFGDGVAFSTRCFINTMRVQRRVRSNLMLDILRRLRQQGISLHPAQRWVQEAMDKDRAEDPDL

>tr|A0A381ILF1|A0A381ILF1_9BURK Spectinomycin tetracycline efflux pump OS=Burkholderia oklahomensis OX=342113 GN=stp_4 PE=4 SV=1

MDTHTPHPALSRAALVRIVSTVSAGFVITQLDVTIVNVALARIGIDLRTGVAGLQWIVDAYTLALAGLMLSAGALGDRFGARRLFAAGLALFAVASFVCGIAANATTLIAARALQGFAAAAMLPNSLALLNRACAHDPRLRARAVGWWTASGAISIAAGPVIGGVLIAQFGWRSIFFVNLPLCAAGLAATLRWIDKDETSAAASGKGAASASRFADSTANTEASRPIAAVRAADIRPRGIDLPGQCLAAVALTLFTGAVIDWHPTLVAVALAAAAAFVFVESRSAHPMMPLALFKQRTFSVAVLFGVCMNLSYYGIIFVLSLYLQRVRHDTPLEAGLAFLPLTGGFLLSNVASGWATAHYGARRPMIVGALIGATGFALLSMTRADTPVAALVVPFLLIPGGMGLAVPAMTTTVLASVERARAATASAVLNTARQAGGAIGVAGFGALASGALPAQIVSGLRASALVSAALFVAAAAIATAVRGVPHRASSAARTKHANPAGADAR

>tr|A0A383TXY0|A0A383TXY0_9FLAO Homoserine/Threonine efflux protein OS=Candidatus Ornithobacterium hominis OX=2497989 GN=SAMEA104719789_00671 PE=4 SV=1

MFEMILYAIMLGVSLSLILIGPAFFLLIETSLTKGWRSAIALDAGVIVADLICIAFAFFGSKDLIHYIETHRSLYIIGGFIIMIYGCYMFVSKPTLHINNEALVNKNYIKTFFNGFLMNILNIGIVIFWFVVVGWVILNYKKSYEIALFMGVALSVFFCIDLAKIFLARKFQRKMSDELVYKIRKALGVVLAIFGLVILLKGFISFAPADHIFFKKSQNIHNINTTSDENP

>tr|A0A385ZKQ6|A0A385ZKQ6_9ACTN Glutathione-regulated potassium-efflux system protein KefC OS=Streptomyces griseorubiginosus OX=67304 GN=kefC PE=4 SV=1

MLASPPVRTGQRVRLLSVRGVLGISWEFTGASGPGRGTTRPGEAVFSLVGSVPNQGPGVPGDTAAHMVVCGDDGLAHRLAAELRGVYGEQVTLVVPASERSVRPPVVVRTRAASALLDRVVTAAVGLTGNGTAGGNGGAGATGGHGSPAHSSGTGGDPRGGIRLMEAAEPSEAALAEAGVERADALALVYDDDETNIRAALTARRLNPRLRLVLRLYNRRLGQHIEALLDQAAALAAGSADGDSADGSGFDASTTVLSDADTAAPALAATALTGTSKVLQTGGLLLRAVERPPAGAGVSAAPGLATLALLSPTDPTDTTGPGGDQGPTLLPDAAAVRDGGERTTVVLEQVSYAGPALPDGRGVMPWFASLFSRRLRWSLAGMVGCVVALAVALWLVTGIHPLRAFYLTLLDLFAIDDPAIGQSVGRQILQLLSGLAGLLLLPVLLAAVLEALGTFRTVSSLRKPPRGLGGHVVLLGLGKIGTRVLTRLRELNIPVVCVESDPEARGLATARRLRVPVVLGDVTQEGVLEAAKIHRAHALLAVTSADTTNLEAVLYARAVRPDLRVVLRLYDDDFATAVYRTLRAAHPGASTRSRSVSHLAAPAFAGAMMGRQILGAFPVERRVLLFAAVEVGGHPQLEGKTVGQAFRAGSWRVLAREESGDAPGLTWDLPDTYVLQPSDRVVLAATRRGLAELLGRRGRVGT

>tr|A0A391NQJ0|A0A391NQJ0_9PSED Cation efflux system protein CusF OS=Pseudomonas sp. SCT OX=412955 GN=cusF_1 PE=4 SV=1

MKAVSLALLAVLWLSGPASAEDLLKPSTTPPPVDDVSAGNALEAEPTHEGEGVIRAIDVQQGSVTIAHGPVPDLKWPAMIMPFKASAAQLRGLAIGDAVEFRFTDGEMDPQIVSIRRR

>tr|A0A396CAP8|A0A396CAP8_9DELT HlyD family efflux transporter periplasmic adaptor subunit OS=Desulfovibrio sp. AM18-2 OX=2292040 GN=DW219_08870 PE=4 SV=1

MLRARTLCRAASRRAVLSFVRVPAVFLLAALLSAQPLTAPLAAPSVSAAEAQPSIGNGATILTGKVVTTVTRAVPVPFNAVVDQVLVKPGDAVHKGAPLLRYHLQEEAERVLQREVTTGAGTEDLKGQALDLERRLAETSAQRNKTRQLVASGLGSRQALSRLEDDVHSLQRRIELLRTTISKTESNFAARLKELGGYFGAPIREGEILPATLTLTAPIEGYVLSLDTTLNAGTLLPAGSAPIRVGQLDPVLIQVPVYEAEISAIKEGDAVEVEIPSLNNKKFLGKVNEISWVSSDMSVANPSYYTVELTVPNPGLELKPGFKAVVRFKGSR

>tr|A0A397L164|A0A397L164_9RHIZ Threonine/homoserine efflux transporter RhtA OS=Ochrobactrum haematophilum OX=419474 GN=BCF22_2514 PE=4 SV=1

MGQGKTGAQALGRASFDGLAVSLVVFIMFSWGLNQVAIKIGSRGFNPMLMAAARSALGGVCVFLWCYWRRIPLFSHDGTLKPGLLAGLLFGTEFVLIFLAMDLTSVSRVTLMMNVMPFWVAIGSHFLLGERMSVRAFIGMCVAFLGVFVVFSDHVSRPGPYAFYGDLLALLSGILWGLTTLLIKRSRLAHAVPEKILLYQLAVAALVPLPLIGLSGPLLRDPGLIPVISLLFQSFFVVAFTYPLWFWMISRYPASKLSNFAFLTPAFGVLLSGVVLGEALSWKIFAALFLIGLGLIIINRPAKATTAR

>tr|A0A3A4NGD1|A0A3A4NGD1_9BACT HlyD family efflux transporter periplasmic adaptor subunit OS=Candidatus Abyssubacteria bacterium SURF_5 OX=2093360 GN=C4520_15735 PE=4 SV=1

MIIHAYKGIIPLALLIPVMTVGYLSFDHWVQRESLPEGLIQANGRIEGDFVTIASKAAGRVQKLMVYEGDSVTAGQVLAQMDDIQVRAKVEQAKQGVAALDAQVRAERTALSKLKKEVPLNIEIAQAALSHAHSVLSEAKAKEERANRDAQRFRQLADEGIADKHSNEQADTAWTIARYEVDSASAALTQAERQLSQTKLGWNQVEAKEQELAALEAQRMRARAVLTEAESIVADMTILAPTSGVITTRMVNVGEVTPAGAPLLIVVDLDHLYLKAYVPEFQIGKLRLGLPARIHTDAFPDKPFEATVQYISSRAEFTPKEVQTSDERVKLVYATKLYLKENPDHSLTPGLPADAIIRWKEGVPWENPRW

>tr|A0A3A4PE42|A0A3A4PE42_9BACT HlyD family efflux transporter periplasmic adaptor subunit OS=Candidatus Omnitrophica bacterium OX=2035772 GN=C4527_08925 PE=4 SV=1

MRGARLYGHVFHVSGVSKNFTGRRTVSRRSSTCAGYVPRYFSSRRVAEGAAIMKRLIPLCLVFIGFALWWWIREGENNRPQFSGTIEAKDVSVASKIGGRTVSVEVEEGDVVKQGDILVQLDRESIEARLRETESELRRARERQRELENGSRPQEIEHANALLEAARQQWQLLKNGPREEDIRAARANTEAARAEVQLASITEKRQKELFASKNTTAENLDRAQKELSVANSRLRAAEAELEQLLAGFRQEDIQASYAQVLAASAALALTMEGPRQEQIAQAQADTARLASVLDRVRIDLQETRITAPSDGVVETSTLEPGDLLAPNQSAMTLILDKPLTVRIFVPESRLGDASIGREIELSVASFPDKRFQGRIVQTNRRAEFTPRNVQTPETRDDLVFGVKIEIDDPGHQLRPGMVADVFLPLVKQ

>tr|A0A3A4VTU6|A0A3A4VTU6_9CHLR HlyD family efflux transporter periplasmic adaptor subunit OS=Dehalococcoidia bacterium OX=2026734 GN=C4555_03815 PE=4 SV=1

MRIPRIWLALLLGVWGLALAGCGSGGASAVGGQLRTVERGDLKISVTGVGNLALSDKRDLAFEMDGTVLEVLVEEAQSVEAGQVLVRLSASDWQEQLVALEDKVTAAERNVTAKGRAVTSAERTLAAKEQAVIQAERNVAAKELAVLQAQANLNNAQLSLEQTQAATTDPLQVEIKRLQVQVAQGNLEAARQALEDARTIDIDNARQAVEDARAQVGDAELAVQDAQKALSEARSNLDEARNKSPEITAPFAGFITRVNVSGGDEVKKGTVAVVLADPAKFEADIPVSELDILQIKLGGTAEVQVDAVSGVTLPARVTRISPTATISQGVVNYKVKVELLSTSLAQAQSASTPVPGAASDNVTGASGSFLGRGNYGDDGFTQEELSAMLAQRQSGQLRQSAAQLLADLQLKEGLTVTVSIVTDSKANVLLVPNSAITTRGGQAFVQVPGADGALEQRAIQTGISDYQYTEVTGGLNEGEQVMVTTGTTATTTSGQTGQRQGGTFVPGGGVILR

>tr|A0A3A8ZYF0|A0A3A8ZYF0_9BACT HlyD family efflux transporter periplasmic adaptor subunit OS=bacterium 0.1xD8-71 OX=2320099 GN=D7V83_15155 PE=4 SV=1

MSQISENKTEEAKVTDVVNDTKENKKEKKAKKEKKPMDKAKKKKIRRRIAVLVIVVLVAGFFVRNSIMAKNTLPTVFTMEVTIEDVEQTLRTNGTVKSMETKSYFAAVMVPVSEVKVAVGDKVKKGDVLLKFDEAALSEARQEAELKLASSQGDYSSSLYKNNKYLADLSEANTNLPVLEQQISDHETYLKGLQKTIEDKKAWYANQGALLQVSLLEWEKTISDEKKALEERNAYEAESQDMDDKEKREKRERDDAAKNQIAQDEETLLSLQEQVQYNSYEQQNNQEIRDLEREAAEIEKIIADAKELKAKMESQKEASEDAMMDTGSKEKLEADTALQKLTNGGTLESIQEVESGITADFAGVVTEINAVEGATPAENGKLVVLESTDKVVIHANVSKYDLEKLAVGQSAEIDIAGNMYEGKVDKIEGMATTNNNGAAVVGVDIGIEKPDENIFLGVEAKVVVHTAKAEGVTTIPMELVNSDRDGDFVYVEENGLVAKRRITVGISNESLCEVKEGLSVGDKVIMSMGQEFEEGMAVTAVPQG

>tr|A0A3A9UN94|A0A3A9UN94_9ALTE HlyD family efflux transporter periplasmic adaptor subunit OS=Alteromonas sp. BL110 OX=1714845 GN=D7031_01095 PE=4 SV=1

MRTGLFRKEVIDEQSNTVEGSFLMTPKPAYLALAGLLVVWVVAVAVYLNMESYARKASVSGWLEPSHGVFKLYSDARRGKILDVIASEGQLVEKGAPLLKISYSSKDALGHRVSTQLLAELESKQNRTRQSIERLRTLHLAQQQRLEEQLSQAKHNSTALHDIITLTQSQWLLASTQWEKAQTLMDEGHISRTDFETYTLQRLNAEQKLALAKKDWSNEQANIAAISHELATLPEKHANELANIKNTYSDLTQQIVTHKSNAEEIIYAPRSGVISGLHVRTGYTVDSSRPLLTLLPQNADIQARIAVPVRSAGFLREGQALHIRYDAFPYQKFGVQFGEIVNISPSLVLPGELTDVPISISEPAYLVTATLNTNEVLAYGNSISLKAGMTFSADVQLSQRTLMEWLMEPLYSIKGKL

>tr|A0A3B0IWI6|A0A3B0IWI6_9RICK Magnesium and cobalt efflux protein CorC OS=Wolbachia endosymbiont of Aleurodicus dispersus OX=1288877 GN=corC PE=4 SV=1

MDWLLISVLSAIFFLLILSFLFSGAEIGLTSISRSRVNKLKLDGNKRAKIIDRLLNTKELTIGTILLCNTIINITCSALFTAIFINFFESEGIFFSTAMMTFCILLFCEVLPKTYAMQNPEKFTLLSAYFMLFFVNILSPLTLGIQFIVNLILKLCGLHKNREVISAADAMRNMITLHRSEGTMLQQDLDMLNSILDLAETEISQIMTHRRNLFSLDIDQNKEDLIREILTSRHSRVPLWQKEPDNIVGVVHVKNLINALREKDNKIEVVKVMSKPWFIPESTPLSVQLHNFRKNRKHLAFVIDEYGALQGIVTLEDILEEIVGEIADEHDLITENFIKKISDSVYHIEGKSTIRNINRQLHWNLPDDEATTLAGMIVNEIERIPEENEEFSMYGFYFKILKKDKNIITMIEVQVKTDNTVVAIN

>tr|A0A3B6Y8V6|A0A3B6Y8V6_PSEO7 HlyD family efflux transporter periplasmic adaptor subunit OS=Pseudoalteromonas piscicida OX=43662 GN=D0N37_23260 PE=4 SV=1

MRKKILAIVVPLWLLGCQEAPEQPTQLLYTVESKPFSITVDAEGELEAASETVISAPTSARGAQTLAWIMPEYTQVKKGDVIARFDGSQLERRKRFSEFDKGKVAQDITVTDSDLTTRKSHLDSDKVIVSEEKHFAETFSIDDERIRSKLDILDQMQNVEYLNAKEAYFGWQTEQFSSSALGEMELLKLQSKQHESKIAMYNANLEGLEVIAPHDGLLTLNADWRGEKPKPGQALWPGQKIGGLPDISSLQAKLFVHEKEALSLAIGQKVEFTLLSNSDERFGGKVTKVSPYPQSIRRGDPQKYYEVIANLDETPAYFKPGNKVLATLFVQENKQALLVPKHSIINDNNAFFVQVKDGSQFKRVKVELGQSNLSHTEVLAGLSPNQQIALVPNKEL

>tr|A0A3D5PVS0|A0A3D5PVS0_9FIRM Efflux transporter periplasmic adaptor subunit OS=Dialister sp. OX=1955814 GN=DGT53_06770 PE=4 SV=1

MAVMNHHGEDFMQLSWLHSKKAKIIIWTVILVFLLCGIYAYTHRSRVSVGKTSAHPLVKVEKMERKDMMKRVVLSGDTVPRESVDISPKYAGRLEKVYVDLGDKVTKGDILISQDTKDISFSIAQNRAGSHEAAADAVESRASYDAGTLKAQSDYDNALSTFNRYDTLFQEGAVSRQERDDKYQAMMEAKAALQSLTGQDVEGVPAVIASKEAAAEKAAYTVDSLESQKGDMTMYAPVSGTIGYRDAEAGEWASAGQKLLTIVDNSALYLDCAVAEQDIGVLREGMDMDVSIDSLGETVKGQIIYISPDLDASTHSYXDVSIDSLGEKVKGRIIYISPDLDSSTHSYKVRILLDGDGKNLRGGMFGRSTVMALERKNALYLPKEGVLENNGKKWAFLIDSSHKVKKVEVTTGLYNDDSIEILTGISEGDRAAVTNISKLKDGMTVDVEGTV

>tr|A0A3E0GZB4|A0A3E0GZB4_9PSEU Glutathione-regulated potassium-efflux system ancillary protein KefG OS=Kutzneria buriramensis OX=1045776 GN=BCF44_118152 PE=4 SV=1

MGESGTPDLWRSRPDLGDHCERGRPNFGGMDALVLLDHPDLSRSRINATLAEAVRELPAVTLHDLRAEYPDRVIDVAREQRLVRVHSLIVFQFPFHWYAVPSMLKQWMDDVLVKGFAYDGALPLLTGKTLQVVTSTGGVEEAYREGGFHRYPMSALLAPLENTAHRVGMAYAPPLVLHDVRGVTPLELAEHVERYRDLLASSGACLTA

>tr|A0A3E0LZX1|A0A3E0LZX1_9CHRO HlyD family efflux transporter periplasmic adaptor subunit OS=Microcystis wesenbergii TW10 OX=2060474 GN=DWQ51_08780 PE=4 SV=1

MPMPLSNCLREDLLVTRKIDRQTSAKRYFIQDPISQETFEFGEEEYFLCQLMDGVTSVPEILASFQERFNISLTEEDYQKFAGQIDSFGLLEPHQNQLPSSQSEEGNGHKNSSPTKKKSKQHSLRFIWKHPNPDAVFTSLARWTHPCHRWLRWSTWLLLPLLPIALLTFWNNRTVLWYDVGRFVDGLPFVLSYLVDILILNLCGRIIQGTVFAAYGGRSSVFGMTLALGFKPHFQVDLREYQSVPRKAQLWIYGTPLIMRLFIFSFGMIFWYAQRSSGTAFHIWLLLLAHAALVTFVLMACPLWPLYGYYFLIAFFRLPDNFMSQSFRAWGMVIKGRNLPSFLSTREKLMLVGFGLGSILFCLLMIYLIVTNFAKGLYTLFPEIFGAESAIIIVSVLVFIGFRKQISRLFFRGRNSQGTSGLPSNLTEETKNSKKPSSRSRQGFQSWLKKNLKFFILAGLVALLFLPYRTMPGGPLQLLTPAEVAIQAEVDGKSKITRVMFPGGNEQLIRKGTVIAQMEDVDIEDTIETLQSQIAKALGDVKIKQSYLAKLLATPRKEDVEVARNQVKIAREEVDKAKKEVAVDKQNLEVIKKQIESALTQADFYFREASRLEEGYKEGAIALNLVEDAQRNAQTKKIEAEEKRQALLQQQQVIEQARSQLASKQRVLETSESQLKLLLAGPYPDEIEAARQDVEVARAELERLRKQEQQERDKLKLTTLVMPLDGYLVTPYLDTKVGSYLDQGETFATAQDATKILAEVQVPEYDVGQFSIGKNVQIKLNAYPTETIMGKVVSITPAAGNSTTTADLSSEPVVKVLVEIPYGKHLFKTGMTGYAKIEGPMKPFIVAFSSPIVRFFQIEIWSWLP

>tr|A0A3E0PTY9|A0A3E0PTY9_9BACT HlyD family efflux transporter periplasmic adaptor subunit OS=Planctomycetes bacterium OX=2026780 GN=DWQ41_26960 PE=4 SV=1

MKTVLQCALLVFCVLLGNLLVEPWGWSVWEMISPAPDDAPPPPAEPDERSRELRVLGRLTPTHGIINLSATPGDRLKSLNVSEGDQVQAGTQLATLESETLRQLEVEAAEEQLAQATALRDAEIKAAEARLRASEKALEQAQAKNPQLEQQKRQVELAALNLAQAEADLKRVQELRADLVAVQEQEQLELLVEKARIEKTAAETALDQLQQADEFQLQTARSEYEVALAGLDQVKTLRRIEPYEAKLKLAQYQHDQTILTAPSDGTILAIPTRPGESVTPEPILQMADTSEMSCIAEVHKSAVDSLSVGQRVRIESDAFEGRQVAGEIVRIGNQVDPPQLRNLDPLAPQNRHVVEVLINIDEGVANVSNLIDLQVDVVILPRESHDGETGDDAQ

>tr|A0A3E4LNH7|A0A3E4LNH7_9FIRM HlyD family efflux transporter periplasmic adaptor subunit OS=[Eubacterium] rectale OX=39491 GN=DXD13_14930 PE=4 SV=1

MRSLLNSSRLILWMLKQPHNFDYEKRKIMKPIIVDLKDISDSTEVYDSKPNRFVPYTIYIICAILAIALIWMYLFRMDIVVKADSVFRGDDDSTAVSCAVTGKITKMSVKDGQYVSEGDELYEIDIENLGSTIEDYKSKLDSVQQRLDILNAYQKSLDGDNSEFDAMSDNQYYSEFKDRKELLNTSIDAGKEKNKTGEVYDENITVINDSIDKYNEKINKLKDVKQCIVSRNNTFDQNDTYYYSMVNSYISSYDYTALQYDNKKDETTMDSSQLAEVDTEKNQALSNLESNEISTIEQQIETANEQIESLKSNISSVELQKKQTENSNNTDDSDIKILTEKGNVSAEILTYEDKKQEYEAYLKDYDIKNNNCTIKAGTSGYFYTNQEISNGTYIQEGDSIGQIYPKEQSGYYAQVYVENSDIAKIKPDQEVKFEMASYPSSEYGYFTGTVKEIAKDVTVDQNTGNAYYVVKVECKNMEIKNKDGEKGNLKSGMAAQAKIVVDDDSVLHFVLDKINLVD

>tr|A0NKH3|A0NKH3_OENOE ABC-type Na+ efflux pump, permease component OS=Oenococcus oeni ATCC BAA-1163 OX=379360 GN=OENOO_63060 PE=4 SV=1

MNKTWIVAKHVFLKNLKSPSYYWMLLAPFVFVLIGIGASFLINKAVSGNQPTIGIVGQPQQVSLLKTALKSKASVKSESSLKAAKNALSNEKIDAYVKTNAGYTKTEIVANSKSSANFDSSSISQIISSLKTESAVAKLGLSAKQVKSITAPASVKTKYVSVENKKVSQNSNNGGRSVRYLFAQGATIIIFMFLAIYIQMTGSEIGTEKGSRILESILAAVPARQHFTGKIIAIVGLFIFQLIAYIFIALIAFALAKPFNYSKYLNLVDWSQLGTSFIVLTALITLGAIIIYIILAAVFASMVSRQEDVAKSTSVVMWIAMVLYFLSFAVASSANAPVFKVLSFIPLLSQSIMPIRMAVSSATTFDALIALALQILIILLLVKFASSIYARNSLDYGDGKPLKKLLKYFQSKNS

>tr|A0YH94|A0YH94_9GAMM Putative transmembrane efflux protein OS=marine gamma proteobacterium HTCC2143 OX=247633 GN=GP2143_06938 PE=4 SV=1

MTFAKHLTLSSPAFVQNSRSVLWVLLVGMLATGFPFTILAVALKLIAQELKVSEALASWSVSAPMLISAVCMPFLGKLGDLYGHRRIFLIGIVGSTMFALLCYFATNIWWLVGLRILSMAFAGATTPSAMALIFHVFDQDKRTQAISWWAMGGPASAALGLIIGGPLIDAMGWRSIFIFQAITGVLAFALALRSLPETGQRTAKFDHQGNILLIVSFCMLLFAVGSITDTSIAGSLKWLSLVLGVVGLIFLYKIETKVDEPIIPPSLLQQKSFMAPVATSFICQAAYLGGFVVTPIVLIDQFEFSIALAALFMLARTLSLTIASPIGGRLSVAFSERAVVLLGLLIQAGGLVVVGLGVLTSNIVLLGIGLVLQGIGHGFALPPLTSVISYCVPPQLFGTASGVSRLATQIGASLGLSFFSALLIMDRNDFGLAEIFYLGAGITLLGLLPAVAITGKTTTQVVLDS

>tr|A1R0X5|A1R0X5_PAEAT Putative arsenite efflux pump OS=Paenarthrobacter aurescens (strain TC1) OX=290340 GN=AAur_0057 PE=4 SV=1

MLEATNTPSKTEEPAAPVNPALAAEAKIARIAVTVFPLLVVVAGVLGFLIPDLFKPMGVAVPYLLGVIMFCMGLTLTPPDFASVARRPWAVALGIVAHYVIMPGAGWLIAVLLQLPPELAVGLILVGCAPSGTASNVMAFLAKGDVALSVAVASVSTLIAPIVTPTLTLFLAGSFLHIDAGAMVMDIVKTVLLPVIAGLLARLFLSKLVAKVLPALPWASAVVISLIVAIVVAGSASKIVAAGAIVFLAVVLHNGFGLGLGYLAGKLGRLDDKARRALAFEVGMQNSGLAATLATAHFSPLAALPSAVFSLWHNISGAIVAAWLARRPLKD

>tr|A3CLU3|A3CLU3_STRSV P-type ATPase-metal cation transport (Calcium efflux), putative OS=Streptococcus sanguinis (strain SK36) OX=388919 GN=ctpE PE=4 SV=1

MNKKELIGLNQTQVDEKISQGLTNDFTSDTSTSNWQIVKRNVFTLFNALNFVIALALVSVQAWSNLVFFAVISFNAVTGIITELRAKHMIDKLNLVSRELVTVIRDGQEIKIQPEEIVLGDLIKLSAGEQIPSDARVVEGVAEANEAMLTGESDLVLKEEGAELLSGSFLASGQIYAEVHHVGADNYANKLMTEAKTLKPINSRILYNLAKISRFTGKIIIPFGLALFFEALVIKGLPVKNSVITSSTALLGMLPKGIALLTVTSLLTAVIKLGMRKVLVQEMYSVETLARVDTLCLDKTGTITQGKMTVEALHSLSDKFSDETVGQILAAYIQTSEDNNPTAQAIRKGYGHLDHAYTSDNVIPFSSDRKWGAMHLSSVGTIFLGAPEMLLDSNPAAVGEAQKRGSRVLVLAHSDQVLDKHSIQLPEDMTALAVLEITDPIREGAAETLDYLRSQDVDLKIISGDNPVTVSHIASQAGFANYESYIDCSKISDQELVEQAEETAIFGRVSPHQKKLLIQTLKAAGRTTAMTGDGVNDILALREADCSIVMAEGDPATRQIANLVLLNSDFNDVPEILFEGRRVVNNIGRIAPIFFIKTIYSFILAIICISSILLGKSEYLLIFPFIPIQITLIDQFVEGFPPFVLTFERNIKPVEKHFLKRSLQLALPSSLMIVFSVLFVRIWGSSHGWSDIEMATLTYYLLGSISFLSVIRACLPLNLWRSLLIIFSVFGFYLSAFVLQHLLEIATLTAATLPVYLILMVVFGLVFVACTIKQKYRFD

>tr|A4CXI1|A4CXI1_SYNPV Putative efflux transporter family protein OS=Synechococcus sp. (strain WH7805) OX=59931 GN=WH7805_00330 PE=4 SV=1

MDPSTSGHGPWNLSSLLDFSRPAHAAWTDAMRGAGITTLLGWIALTLNAPRALLPLTLGSVFTAIAETGQGRDHPWRTMAWTTTWLMVAAGFGAAIGENTPLAVFASGAMGFICASAASRDKRTAVTSLLTLVVFTIYVGYPGPIVPALQDMGLILLGGVIQTLVCSVVRAFQQVKHERLCIPPIWRHLRTFRTSDAHVRHGIRLAITLMVATAISESTGLPHQYWLPMSVAWMSRAQLNSTCQRVLHRLLGTLLGLGFIALVVRWIGPQGAHWLPLSLLGAGILIAYVWVHYAAAVVGVTIWIIAAFALVGDPVIDTLWNRMLDTTIASAIVLMAVWIDPRASES

>tr|A7HZG4|A7HZG4_CAMHC Transporter, auxin efflux carrier (AEC) family OS=Campylobacter hominis (strain ATCC BAA-381 / LMG 19568 / NCTC 13146 / CH001A) OX=360107 GN=CHAB381_0033 PE=4 SV=1

MIYQSLFTIFILLAGGYISKLIKVLKQKQSRSLFDFVVVFALPCLIFDKIYHLNFNFSLILLIFAGFISTSLAGIISVIIGRVFKFSKPTILSMFVLSAFGNTLFVGMPVVSNVFGEEFVGEVIMYDSLAGAIPISILVPLILAMNNGEKVTIVKNIKTIIYFPPFIGLVLGLALKGFEIPEFVFAPIRMFGGSATPVALFAIGLSLGFNAIKSSYKSTVIVLFMKMILAPAIFILILQCFGAAFDKSTLIAVLESSMPTATIVCVMVMKAKLDSNLAASSVAFGLVLSVITLPILLNILTGLSTF

>tr|A9ED99|A9ED99_9RHOB Cation efflux system protein OS=Sulfitobacter indolifex HEL-45 OX=391624 GN=OIHEL45_16441 PE=4 SV=1

MIDPPALAGWTVVILGGVALVVDTLTALLTYSMQKGSVNIRALFLHNLSDALASVAVIFGGTLIIFYDLRWVDHTTASGSYISAMRMLLRLATFCLLLILAAVGSALILPHVSLAMTTGAAVYALHYDGIGPILGAILTASAASATVSKGVWLLAIGAVGRCLHQVAGGVMVLMGIGMMTGQLSAFS

>tr|A9M0F3|A9M0F3_NEIM0 Efflux transporter membrane protein AgrA OS=Neisseria meningitidis serogroup C (strain 053442) OX=374833 GN=agrA PE=4 SV=1

MFFDNNYHYNAIPVRLLVNIQDADSDGIQTASENNNGTRMGYNRPMEKNTLSARAPSPWLPLLLAIAIFMQMLDATILNTALPEIATDLDESPLDMQLAVVAYTLTVALLIPLSGYLADRFGTKKVFFGSIAVFMLGSALCAASGSLFELTLSRVVQGIGGSMLVPIPRLTILRMYDKSKLLNAINYAVMPALIGPVLGPLAGGYLVEYASWHWIFLLNLPIGLLGFILGRNIMPDVKGNDTALDFKGYLTFSAAACLLLLAAESLSHALPPYFALLPLCGGLLFARRYFRHMKTTSKPIYSADLFLIRTFRLGLAGNLFSRLGISSIPFLMPLMFQVAFGFGASLSGWLVAPVALSSLLVKPLIAPLMKRFGYRTVLLWNTKLLAAFIMLLALPDGNSSLWIWIFLSLAIGACNSLQFSAMNTLTLADLRPQQTGSGNSLMAVNQQLAISMGIVVGALILKNWTFLIPASSGLHSAFRMTLLSIGGITLASSLVFNRLHVSDGTNLTRNTPS

>tr|B0SJR0|B0SJR0_LEPBP Glutathione-regulated potassium-efflux system protein KefB putative membrane protein OS=Leptospira biflexa serovar Patoc (strain Patoc 1 / ATCC 23582 / Paris) OX=456481 GN=kefB PE=4 SV=1

MKTRSTVFYGFTLLLFGSLGYYLLQAGGVLESTKNLTIATNGHLDTENFFNRFHHPLALLFLQIIVVCGSARFVGYLFTRKLKQPSVMGEIVAGILLGPSLLGYYFPETMSFLFPPSSLPTLGTLSQIGLVLFMFIIGMELDLSVLKNKAHSAIIISHASIIFPFFLGMTLAYYFYTDYAPENVGFLSFSLFMGIAMSITAFPVLARILQERNLTRTPLGAMVLTCAAADDITAWILLAIIVTISKAGNLNTALFTIGLSFAYILTMIYLVAPFLKRLGSIYISRENLTRTAVALILMILFLSSLTTEVIGIHALFGAFLAGVIMPSEGNLKKLIAEKIEDIAVILFLPIFFVITGLRTEVTLLNGSHLWLVFGLVLFVAVVGKFLGSALAARVSGSNWEDSLSIGALMNTRGLMELVVLNIGYDLGILSPEIFAVFVLMALVTTLSTGPLLDGIQKFFARTANATYPEKPSDSKLRVLVAFAQEKMGKSLVRFAFSLSGNQKKNLELIALHISPNDSLSNEEIRKYRDASFEAIRQTGSSLGIQVQTEYRITDNVTYEIVNFAKIKHTDILLIGAAKPLFSRSYTGGKIKGILNYCPATVGVLIDNGLESIERVAILYKGEKDPILGFAQKLTSLKGMKFNKIKVENLIQPETDLNPFPIALSQITGYSLILIDLNVWEELGFEKMDLLPTSFLLVRFLTT

>tr|B2FS78|B2FS78_STRMK Putative transmembrane efflux protein OS=Stenotrophomonas maltophilia (strain K279a) OX=522373 GN=Smlt2498 PE=4 SV=1

MSGMTDGQQLRNAQWGKVSRLFKPAMIISAALTASAETFYRTGAYPRAIFEAGSTDVRTWLYVALMYLIALPVLFLWMRRLLAGYPMPWNPPLKRWLLGAFSLILCSGMIVLPVIVLTVGGSAAGRGKGLYQLFTGNLFGTFLVGTVLAYGAALGAWLLFIGTPKLLFPKLGSR

>tr|B2V8Y0|B2V8Y0_SULSY Auxin Efflux Carrier OS=Sulfurihydrogenibium sp. (strain YO3AOP1) OX=436114 GN=SYO3AOP1_0770 PE=4 SV=1

MLENLLQIFVFFILGYTAIKLKIIPQEYSKAYIDYIMNFGFPALVVYNIYRLRFSLDVLGIIILGWIAIFLTIFVSHLISKSLKLDKKRTVAFIMMSTFSNTGFLGYPFIHSLYGEEGLRYAVIFDNLAMFLPIFLLAPFIINYAKEGSTKINIKKLLLFPPFIALVIGVSLKPFDVPEIFLNLLKTLGMTVIPIILFSVGLNLRFSHIGKDLKLLTVNMLVKLFASPLILLLILLILKIDLTLPYKVAILQLAMPPMVLASIYLIDADLEKDFAVSSVAIGIILSFLSVPIWYFLLNSLST

>tr|B4EPG6|B4EPG6_BURCJ Putative auxin efflux carrier family protein OS=Burkholderia cenocepacia (strain ATCC BAA-245 / DSM 16553 / LMG 16656 / NCTC 13227 / J2315 / CF5610) OX=216591 GN=BCAS0049 PE=4 SV=1

MHTELAWVAVAPAFALICIGIAVRRLALVDAAFWPSAEKLTHYVLFPAFLVHSIGLAGPLDASSKSTIVLLTGLTLAVLAAVVLGCRWCAVPHASFTSIVQGSIRFNSYIFLSVASGLLSRADYGIAAVVVAYMVAISNTLVLLSFEHGQAGGRGLVRIVGKVAANPLIVASAFGIVLNLTGWRLPAAVDQTVDVLGGAALPLSLICVGAALRLPLPRKEAALVRAGLVTTAIRLVGFPLLALTATKAFAVTPLSGNLILLYSVLPCASNSYVLSTQYGGNHRLMAFVVALSTVLSFVPIFLVARTM

>tr|B4V7X1|B4V7X1_9ACTN Transmembrane efflux protein OS=Streptomyces sp. Mg1 OX=465541 GN=SSAG_03935 PE=4 SV=1

MMTGARPGGVGRGNCCIMQQDHPSPSSASHPPSPPRSPSMLRLASAALAGTAIEFYDFFVYGTAAALVLGPLFFPSFSPLAGTLAAFATFGVGFLARPLGSAVFGHIGDRYGRRPVLLGSLLLTGLATVAVGCVPSYASIGVAAPVLLLLLRFLQGLGLGGEWGGAVLLTAEHAPEGRRGLWSSFPQTGPAVGFLLANGLMLGLSATLTDGQFTAWGWRVPFWAAGLLALAGLWLRRSVEETPQFRALAETGGRAEAPLTEVVRGHWRLLLLTGGALAVGYAVFYSVTTWSLAYATEHLRVERTVMLACVMAAVALKALATPVMAVLGDRYGRRPLCLAGCTACALWMFPFVALLRTTDPLLMTAGCFVALLGMVAMFAVVGAYLPELYAPRIRCTGAAVGYNLGGVLGGALTPIVATALADGSGPPWGVAVYLTGVALVSLVCFALLPETNPALVRAKEAAGAGTTTGAAEAAAPA

>tr|B6YZ23|B6YZ23_9RHOB Transmembrane efflux protein, putative OS=Pseudovibrio sp. JE062 OX=439495 GN=PJE062_3819 PE=4 SV=1

MARKHLVLLNMIGALALVMIDQTVLGVILPSLQRTFMFGPVHLQWSVNAYLIALASMLMCGGWLGDKFGYFTSLRMGVALFTAASLACAYAPTGEFFITARVFQGAGAALMQPAATALVFSAYPADERGKALAHYVGAGLFFLACGPLVGGLLVEFFSWRVVFLLNVPIGLAVLIMAFAIGKSKTNKETGSFDFQGAILFVMALLVFTVAVQLFGDYRLSLGEGVVSAGFVVLACSLLFVRRHRVAHPFIQFSLFENKVYLGCCLLLFCIPFALLAQVVFGAVFLQNVLSLTPLEAGLSMLPVVLTIIICAQFGGRLVGNIKFRNLAVCGSLAMGIGFATQALVIHFNNLWLLFPGMILMGAGLGFLISTVSTEALSHVSLLARARATALLQTCRQVGGVFGIACVGALINWREKTMISAAAELMEPNDEDRELLQLLLYKFMGDQPAAAALLHERWPQSLYILKAISSRALADAYIFSAVVLFGAAVMSLWAFKGHTPRQRPQGPEGPPPMNSAND

>tr|B8DYY8|B8DYY8_DICTD Auxin Efflux Carrier OS=Dictyoglomus turgidum (strain Z-1310 / DSM 6724) OX=515635 GN=Dtur_0291 PE=4 SV=1

MFLTVFSKFLPVFLLFLLGNLFRIKSYVSENAVSELKKLVVNVFLPSLLFLSFSRTGIEPKHLIIVVIMFLVCTILLFIGRFFQKLLKVDSKYFYLLFTGFEAGMLGYSLFTVFYGTENVFKFAIIDLGQVTFVFFVLVGILLSIKEGRRSWNFKSMLYSFLKTPVIIAIFLGIIFQKTKLIDIFMKNILLSSILETIEMLSVMTVPFISLIIGYELKFQKENLSLSFKVVILRNVVLIVLGFLINYIVINKMLGLDNIFQRALITMFLLPPPFIIPLYIRDEDREDKVFISNTLALSTLFTMLIFLLMNILGGVTL

>tr|B9KBM6|B9KBM6_THENN Auxin Efflux Carrier OS=Thermotoga neapolitana (strain ATCC 49049 / DSM 4359 / NS-E) OX=309803 GN=CTN_0246 PE=4 SV=1

MERKAEKGGEKMPYTSFSSIIPSFLIILIGYAVGKVFSDEVVGLASKVAIWVMVPTVTFTFINKYTPGFSELRDFGLGIIVIFLFFYLYSSFFKHRRGVVLVTAVTSNAGYLGYPILMSLWGEQALALGVVYALLIVLMYTILPAFLGERFNLKNLFKLPYIYALPAGFITGKLGLHYEDLPSYLLSAINMLKQAAIPYLLLYVGLSVSRVKMDKRVTGLGGLIIFNKLFLSPLIALLFVMIYKLDGLSGKVFILETAMPTAINSVVIVSALGGDSKTVGLGVTLTTFFAIFTLPIWAVLLEKIFG

>tr|B9NVI1|B9NVI1_9RHOB Putative transmembrane efflux protein OS=Rhodobacteraceae bacterium KLH11 OX=467661 GN=RKLH11_3546 PE=4 SV=1

MHHWFIQASMDEPMDRKSIIKVIALMVTGFGIGIDFTGALMLVPAIENSFDTDITSTQWVLNIYALFFAMTMVAGGRLGDMYGHRKMMIIGLSIFLFASVLCFVSPGLDYLIGARALQGIGAGCVWPCTLAFGATKVSKEEHRALVMGLILAGVTTGNVFGPMISGAVVNLGDWRLFFLANVVFSSISMVTALLLMERETEHKTGEHIDFAGMGILSFAVLLLLYGLDIGADWGWTSPPLLLLFFVSAALFFLFPKVEKRVREPLLLPQLMQNREFLITLGLNMFNVSAAFVGLLYFPQYMQKVLGWSVFQSALGLAPLTILLAVGSVVSGTLYNDFGPKRLLFWGYVCATVGAASIVVMPAGLGYFQILPGMALIGLGATLTVGPSGTAAVCAVKPERAGLVGGLSFMTHLVYGAIAVAGATAVMYVTSLSSLKTQLAAAGINMPEADQRAINGGTLTTESAKAVLQKLSPGEVEKVKAAIATAFDSGMNMAFVFATFSVAVGIVLALMLDEKKLHKVEG

>tr|C0EE40|C0EE40_9FIRM Transporter, auxin efflux carrier (AEC) family protein OS=[Clostridium] methylpentosum DSM 5476 OX=537013 GN=CLOSTMETH_02119 PE=4 SV=1

MQAGLTILNQVFIMFLLILTGYFCHRLGLISARANKQLANLLITIVTAALIIDTYQTDFDPAAARNLLISFALSFGILFLGVVVSLLMKRKGSEYSVPTERFGVIYSNSAYMGIPLLLATVGPTGVFYSSAFMVAFNVMTWAQGATMLTGQRSLRQVLRALVTPVTFSILISLPMFFFRIRLPQPVGDAIGYLASLLTPLSMLVSGVFIAQTNLVQAFTSLRVYAVSALRLLVIPAITLFALWVLPIDGDLKLTMLILSAAPCATGTMLFASRFGGDVQRASGVFAVSTLLSIVTMPLLIIVAEAIW

>tr|C0K045|C0K045_9BACT Putative efflux outer membrane component OS=uncultured bacterium 34R1 OX=581113 PE=4 SV=1

MRAGNSVGVQFFSLCVLNGKMLSSCAFLHYLCAIINNVFMRYGLFIICVALSGVLASAQQVLSLDSCRSMALRNNKEIKQAQVGEEIAGYQRKQAQAAYLPSVDFQGTYIYNSKKISMVEKDELLPTKSFNLATGTYDYNLVINPATGQPLVVDGTPVPSTVALLPKSALTYNIHNIFVGALTITQPIYMGGKIKAMNEITRYAEQLARLTRNRKAEDLIYEVDAAYWQVVSLREKQKLAESYVQLVESLDRDVNNMLKEGVATKSTLLSVDVKVNEAHVDLTKVNNGVVLARMLLAQLCGLPVNEQFVLEDENGNDPDISNLKPARIDMEDVYSRRNDVNSLVLATKIYDEKAKVARAEMMPTVAAIGAAHTSNPNMYNGFKNRFGFGFSIGAVVKIPLWHWGGLSNKYKEAQAEARLKRIELEDAKEKIELQVTQANFRYEEALKTFEATKANLTEANENLRIAQIGFKEGVATADDVLAAQTAWLKAHSEEVDAEIDVRMCDVYLAKVLGKMNY

>tr|C0ZNM5|C0ZNM5_RHOE4 Putative drug resistance efflux protein OS=Rhodococcus erythropolis (strain PR4 / NBRC 100887) OX=234621 GN=RER_05690 PE=4 SV=1

MSSKSSISPCTPTKRCADVTQARLRHPLTTQASPIPRTGKELRMSTQPKSTRKWWALALIAAAQFMVIMDTSIIGVALPQMQSDLGFSQEGLTWVFNAYVIAFGGLLLLGGRLSDLFGARRVFSAGWLILLIGSVVAGAAGNVAVELAGRAVQGAGAALIAPSALTLLMMLFGSTQKEMTKALSIYGAAAPAGGTAGVFLGGVITEYTSWPWVFYLNIPIAVIALIATPLLMPNAPARTGSIDFLGALAVTAGLAVGVFGIVRAPDVGWGSGQTWLALAVSAALLGAFVLIQSKRREPLVRLGIFKAPNLGAANIAQLVLGAAWIPMWFFLNLYLQQVLGYSAFPAGAALLPMTIFVMLGMVVVAPRAMARFGAKAMIVTGLLVLGIGLGWMSLIRPTGNFWVDVLPASLVAAAGMTLAFIPSLGTAISAARPEEGGLASGIVNVSYQVGSAVGLAVMTAIAAVFGADQLGDLTELTNGFSAVFLGAAAIAVVGAAITAIVMRSTKVDDPRPVQSTVNS

>tr|C5ZWX3|C5ZWX3_9HELI Putative efflux system outer membrane protein OS=Helicobacter canadensis MIT 98-5491 OX=537970 GN=HCAN_0927 PE=4 SV=1

MSLKTLLFLCILTSFGYTLSLQEAIDLTLKANHAIKEQEFLLKEAQYNYKTYQSPFYPSINATYSTDRTNKISSQRSRKTSGNIGANIQFNLFNGLSDYYNLASYESLSKAQEHQLQATKEDIILLVKTAYIDVLRQKQNVIVAEQSKALLEEQRRESAEFYKVGLIPKNDLLKVEVELNNSIQALLSAKSNLAYSLKNLERYTRTKINLKDLVELTLHQPTLIESTLKNLMYQKRSELLFLDSVIQSKDYLVKSAKGNFLPNINIIGDYTRYGEDYRLSKRSNTYNDETMITLQINLNLFNGFNDKYTLESTKVNKLAFESQRITLLEDLDLQLFSALETYNLSLNAYQVALSALTQAEENYRISKNRYKERIQSTSDFLDAEYLLTQARTNVVLNRYAILQALAEIERITQTPQVLN

>tr|C7RFL9|C7RFL9_ANAPD ABC-type Na+ efflux pump permease component-like protein OS=Anaerococcus prevotii (strain ATCC 9321 / DSM 20548 / JCM 6508 / PC1) OX=525919 GN=Apre_0228 PE=4 SV=1

MSRFITVALDSWKKQLKSPAFWLVVFMPIIMMAISGAITYFSADDGIKETYIVAEDEIGAYFTENSAYKLKNKDEARKAMEDKEIGSFVEIREEDGSLSAKYHTRDLNGQEIAAFNSILREVQNSINIKRAGLGEDKLKILERKPSFKLVEEEGGESFIMYGAYFALVFYMYMMLVMYSNILVVEIATEKGSKMIEFIFSSVKAGVYFAGKIFGNFLAVITQTAIYLILALLAYFGAKRYGLFEKFNIDLGSLLGDINVLMLVELASLVILSLLIYMILAAMLGSLAKKQEDAGKVGTPLILVIIFAFVIALSFMGKEETLLIKVLSYLPFVSVFFMPMRLIRSSVGLGYGLISILIMLVSIILAYKIASRVYKKNILNYSSNSWIKKILRKA

>tr|C8X5M8|C8X5M8_DESRD Auxin Efflux Carrier OS=Desulfohalobium retbaense (strain ATCC 49708 / DSM 5692 / JCM 16813 / HR100) OX=485915 GN=Dret_2443 PE=4 SV=1

MVTDIVWAVVQIFCVFGLGWLARHLNYLQGMDIGRFSRLAIDFLMPCLVFSATTEHFATERLHELWPLPVIAFAIVVFGTCVGAGARLLLKKRTVAQRRTVHHLCAVNNAVFLPVILVENLWGDAAVANLFFFSLGAALGLWSIGVVLLGGGSWRDGVRHLLTPTHLALLMALTLCLLGATEAIPTIVAQTTAFVGSAAIPLVLFLVGASLYPLPDFSPRRLVLLTSCLRIIGIPVLLTLILRQLPIVDDVYNMAVVNAFMPAAAISTILTHRFGGDPQLAASTVVATTLASLITVPLGLSWALI

>tr|D0SWP0|D0SWP0_ACILW Transporter, auxin efflux carrier (AEC) family protein OS=Acinetobacter lwoffii SH145 OX=575588 GN=HMPREF0017_01714 PE=4 SV=1

MHIILTSLFPLIGLISFGYLLKRRQWLSDDFWRGAEKLNYYALFPVMLFLNLATAKIQMDVIQDVVLVVFSIMAVVSIALYILRHIYQISYARFGVYVQGLLRFNTYIGLAAVSALFHQQGMTIFAVIMVLCIPLVNILSVLAFTRSHDMQLKKIILDLSKNPLILGCIVGGLFNLSGLSLWTGAEQFLKQIALCSLPLGLMCVGAALQFQGFQRDVLPLSLITFGRLFGMPLIAFLVCKIFQIDALTTQVLVLFFALPTASASYVLTRVYGGDSELMASIISVQTVVAAVSLVLMLSWLI

>tr|D3CT55|D3CT55_9ACTN ABC-type Na+ efflux pump permease component-like protein OS=Frankia sp. EUN1f OX=102897 GN=FrEUN1fDRAFT_0722 PE=4 SV=1

MSTGADGGSDGDTGQLPSFPTQGGRPPDDRGAWVTGPPAAPPAAPASGLASGQASGLAAAEAAEPGGFPPLAAWVVVRLVAGRELAIRLRSKVFVITTAAFLVLLVGASVVISLLGGHESAKSVGFIAAESVLADPLEAVAGGLGVDVATREVPDEATGLREVSDGDLDVLVTAAPSGLRVTVKEELSEDLRGVLAVLARQQVLDNEISVLGGDPARVNETVAAAGLDVAELDPVPDDQDERLFLGLASAFLIYMGLMLFGPAVSQGVVEEKSSRVVELLLSTVRPWTLMAGKVLGIGLVALIQMVVIAGGGLIAALTTGALELPSSEATGTVIWSVAWYVIGFFLYALPFAAVGAMVSRQEDLGGISSPIVLALIVPWVVGVSILPGDPDNGLVEILSLVPLFSPLLMPMRIALGVAPLWQLVLSVLLALALIGLLIRITGRIYHNAVLRTGARVAFKEAVRRA

>tr|D4YYV2|D4YYV2_SPHJU RND-family efflux transporter OS=Sphingobium japonicum (strain DSM 16413 / CCM 7287 / MTCC 6362 / UT26 / NBRC 101211 / UT26S) OX=452662 GN=SJA_C1-07000 PE=4 SV=1

MNKAGYQANLGPNMFSRSSGSGLNDNMAGPSLYLQQRIWDFGRAKGEIGYARSTEEQRRYELESVADQLAEQAALAFLQVKRFELLGREAARQVEALEHLRELIGLRVDAGISDKSDLMLANVRVDSARGDAILAESSLITARAALANLTGAMPTVYQDPNPQITRFGAAEEEPDFASLPAIVAADKAEQAAAARVGQAKAERYPQLGLQLGYTRNNYTYNERNNAFSALVTVTGDLYKRGTRYQVRAAEEERRAARSARDSVLLDARGRALTARQEIRGGALRIEAFSHQEKQAEEASRIFFEEYKLGKRTLTELLNTQLEIYRAASARIVAEYDVLAARIRFENVRGTLRPSLGLPARLTEGEEEHG

>tr|D6KDL8|D6KDL8_9ACTN Transmembrane efflux protein OS=Streptomyces sp. e14 OX=645465 GN=SSTG_05043 PE=4 SV=1

MAVLLAANAVAGAGLGAAFAASAAGLAALRDSERAAATTVFGSTVVLALLLAGVPQADHRGGAAAGFAVLAGCCAAAWWLMRYLPDGQRPHPGPHPSAGGQAPPWWFIASVAVLAATDQGAWSYAGVLGERHAGLSAGAVSVILAVAGAAALVGVPVSAAVARRLGRVPTLVLVIAADAVAKLLAAASGFDPLYAAATVVWQICYLALLAQMLGAVAAVDRSGRWAAAAGGALAVGTGLGPAMTGWLLDTAGAPVLGGTLAAVTVVAAAPLLRTVRGLALPADSTDA

>tr|D6T174|D6T174_GARVA ABC-type Na+ efflux pump, permease component OS=Gardnerella vaginalis 5-1 OX=682148 GN=GV51_0406 PE=4 SV=1

MSGQINQSVDQATSNSYKTGRSMNAQHLRLTFMHSLKSEMVKLRGLTSTWWCMALAIVLPVVFSFIIAIVQKAISKVDFTKQGASASKSSGAVTMGPSDKSSLISSQEGIFNLVISFASISLIVIAIFAVLAITAEHSTTSIQASLTSVPRRGMFFTAKFVAIAIYVFVVQLIAMAVSLVAAELAFMGENISGLSGSRTWQLPLMLFLGSPVIMVVVAAMAYGFGMICKSTAGGIMCVIGAVMILPTVLSIIVISSGFAKWTLVLTQLLPATAVSQFLGDSSSSNGKFASNVNVAFEWWQSGLVVLAWAVVMYAIGYVIEKHRDI

>tr|D8F7G2|D8F7G2_9DELT Putative Potassium efflux system KefA OS=delta proteobacterium NaphS2 OX=88274 GN=NPH_3875 PE=4 SV=1

MFFLLLPACGAVGNAPNSWEALLKHQHRELIETSKSIDQLASKLPHRLSDLQKHLYLLKSRFERLMLYFDLKSDNPLVLRDIQGVLDWFESEADRLILPFKQEKASVTRQMENLADLSRKFRQEEILIEKAGPQIQAEVTSYLKDLADLEARLQPVNKNLAKGIDAAHAFILELEKNRARIEHASSLVLKTHLVKRAPVFFSASAWLTGMAALRSWTSRFGLYLLEPIDLRGLGWTMFIAKIVLFSLLIMGLWVGFKKKIQKRYEGLATVRLFPFFLSFSIGIGALLGILSTGLFPSSFFSTLVTVVLVYGLLSLSRNLHGVLFFDQDTRYCRLLPFWGAVSVSGLLVSIHLPEQAFIPVWSIWLLILCWYYAGTKVGKEGWETFSRFILIAATPILVLMGMLLFLLFLDIRLAEMLSTALGRMGPDRKIMTTAPDSSPSKKVRRLGFPLILLTLLLISFAWTFIFVGGGPLFLEVIRYRVGWENFTFSIYRVLCIFALLFIIRASIALARSALAKLPDRRRDLDAGSLQTLDTIITYVLWSLFTLGTLAFLGLGLRNLAVVAGGLSVGLGFGLQNIVNNFLGGLILLFGRSIQPGDLLEIDNIKGHVRKVTIRNTLIKAFSGATIFVPNPLLISQKMINWSHSDRRYRQEIKVGVAYGSDVQKVTDLLLEAAKQSPKVLDRPPSRVRFLDFGDSTLVFSLRVWIKGWADRYADSEVRYHIDRIFKENGIEISFPQLDLHVRSAISPKMEC

>tr|D9XY84|D9XY84_9ACTN Transmembrane efflux protein OS=Streptomyces griseoflavus Tu4000 OX=467200 GN=SSRG_05496 PE=4 SV=1

MNEPPSSSPAVAHRWVSLFAICTAAGMVWLAFGDLSVAIPQIADEFNGNLSSLQWANNAFSLVTGALVITAGKFGDLFGRRRMLQVGTVLLAVFSVPAALAPDIGWLVLSRGLMGIGAALILPASLALIPPEFSGKAETTAFSVWQAVAWGGLSVGPALSGVITDGLGWRWLFWINLPLAVITLVVVRVTTPESRDEKAGHTIDWLGLASIVLAVFALLYALTEGPSQGWGSPLIVALFVATVVLSVVWWFVERHVSQPLVDLKLFKIRAYNGALAANLTMNFTFAGMSFLLVLWLENARGYSAVEAGVLMLPATVGVFLFIPLGGRLAIRWGGRLPAVVGLVVASAGLTLLGSLGTRSSTEYLAVALIVIGLGLGLVSTPVANTTVGEVPIDLAGTAAGVFKMSSMLGGALGVAVLTAVARELTTRDAASVVEASGLSPADISQFRQALVNSSSFREAIASLPPDLGRTVQQAAVSAFSSGVADTMAVTAVLTFVGTAAVFFLWPRRRKADATVAGPGPEDRPNP

>tr|E0F2J6|E0F2J6_ACTPL AEC family possible malonate efflux carrier OS=Actinobacillus pleuropneumoniae serovar 10 str. D13039 OX=754259 GN=appser10_3610 PE=4 SV=1

MQAVKFVKNIANLTAYFLLGSRIMFFESLQFSIGVMLPTILLMLLGIFLRRRKFVDDDFCNTASKVMFNFALPTMLFLNVVKSPLDYSKDLNLIFAGLSGTLIIYLIAEWWAAKYIRERGYRCIFTQGVFRTNAAILGLALTINAYGEAGLATVSIYTASLVILFNVLSVITILNSLSDQKPSAARLAAAVAKNPLIQAIVLGIVVNYLQIRIPKSLMQTAQSLANITLPMALICIGATLDFKALSQFRQQTAESELTRVVLYASFSRLILAPLFLFILGKWVFALNPMQLGILFLTATAPVAAATYAMVRAYGGNGKGAANLIGITTIGSIFTASLGLFVLHHLGWI

>tr|E1SP64|E1SP64_FERBD ABC-type Na+ efflux pump, permease component OS=Ferrimonas balearica (strain DSM 9799 / CCM 4581 / PAT) OX=550540 GN=Fbal_1485 PE=4 SV=1

MIALIWLKEMREVLRDKKTLWFVVLFPTVLLPALMGGAIYVGASSVKQVYESDLRFQLVAPEPWRGEIAEALTNGERLVWDDQVQVSNREQFDAAINEGVLEFVLVVPDDFSATASEVSQWQLYYNQADDVGQFDRIHQALQPLFEQWQTEHRNAWNLTESQVQVLKQAVELEQVGVADQREFIGEKVGGFLPYALLLLCLMGALLPALDLGAGEKERGTLETLLMAPVSKTTVVMAKFMVIAICSLTVALLTMASGVVWSLVLGQVFAIEMLVEAISTIGMMDLVLILLLLLPIAMFFAALLLAVSFYARTYKEGQNYVAPLNFVAILPAMVALFPGITLTSTLAWIPLVNVTLASKALLKGTFDYWQLMPIMASNTLLAALLLAFCVKWCSREQVLFR

>tr|E2CI87|E2CI87_9RHOB Putative cation efflux system protein OS=Roseibium sp. TrichSKD4 OX=744980 GN=TRICHSKD4_2787 PE=4 SV=1

MHSQHQDIGPHARLHGRLFQARPHSPRRFGGGKFTSFWPIYICAGQIPIIAGKGGQALPGEAVLSGVIECFSMSDLV

>tr|E4LUR2|E4LUR2_9CLOT Transporter, auxin efflux carrier (AEC) family protein OS=Clostridium sp. HGF2 OX=908340 GN=HMPREF9406_2375 PE=4 SV=1

MELSILFMKQLLVMFSLSGIGFLLAKLKLISNEGCKELVNLLLYAVIPLTVLNSFLVEKTPEKTQLLLYSLLLSLAVFAVSMLLSYIIYGKRKRVENFSAAFSNAGFIGIPLVQATVGPHAVFYIAGFVAFLNIFQWIYGAYVMGAERRMISFQVIAKNAVLLSFITGFTLYLCDLGNILFIKDIANTVAYMNSPLAMIIIGVYMSQISFMRMLQRESSYICSLCRLFVIPLASLGLLAVIPLDCCEVKVAICIVLSAPVGANVAMFAQKFHQDYTYAVEIVILSTLMSVVTLPMIVYAAQIVL

>tr|E6L295|E6L295_9PROT Outer membrane component of efflux system OS=Arcobacter butzleri JV22 OX=888827 GN=HMPREF9401_0571 PE=4 SV=1

MFKNIFIFFLISTSLYAISLKELLNSVEVTNENYQAQQALQEMSKKQYESATKDNYPTFNLIGAYENNSKVLKTEPEDIAYAELKASYTLYDGERIKNNELSKKSLHESQQLKTQYLKQEIMLEVIKQYFSYQNTKSAIDVINYKINELNGQIKKFEILVKNDLETKDKLQALIASKKEALYDIETLKIDLENSILQLSLLTGFDILPQDNDKLMEPTYDEKDRFDIEAKRLEAKSVKYTSEGFNYLPTISINNSLKKQEYYHYDETYNDKFNNQIMLQINFPIFDFGKISKDKEASQLEALALNKEIAYKEKSIQIERKLALKSLESSKVKLDSAISGLEATNTTYEFSKKRFDANLISYTEYLTELTKKQDANYRVILAKNDIELKKANLAFALGIDLLTLIKE

>tr|E6XRJ6|E6XRJ6_SHEP2 Catecholate ferric siderophore efflux transporter, permease component 1 OS=Shewanella putrefaciens (strain 200) OX=399804 GN=Sput200_3684 PE=4 SV=1

MQRLFSPTSTLALALRPAIPALTFGMLLAAGAGVTTLGALWCMIQLIAGHHSLWLAGALIFGLVSALLSAGASWVNHNAEAQFSGRLRRDVAAHLVRLPPSTLAKYKGEKLKRLMVDDIASLHHMIAHLPSELATFIIVPAITIGLLILSAGLVVLLALIPGLVAALFYLVVIPRLAAKQGEARFNVMGNITAAVDDYARGAPVFRIYGAQTGAMADYQKATTHFIGDILERVSKVSTSVAVATSLLQAVCTFAIVYAIGYEWPPEKLAAALFFSLAIVTPALKLGHGLDYLATGRAAAQRLTDFLEQSRVPVGNGNIELNANMQLTLTNVVPSLSTSNQSVPINYQFSSGRVTAVTGISGVGKSTLLRLLAGMEPLNAGDIRLAGIALKEMDETSINRAIMLLPQGAGLLATSIADNLALTAPYATDDDYLAALHCAQLDNPLNTHASALSGGEIQRVNLARIFLSPARVILLDEPTSALDSETAFNVFTVLRHHAKTNHKTIVMVTHDLTLAELADDKLTLKHHHREGEGQ

>tr|F2IEW7|F2IEW7_FLUTR Auxin efflux carrier protein OS=Fluviicola taffensis (strain DSM 16823 / NCIMB 13979 / RW262) OX=755732 GN=Fluta_0425 PE=4 SV=1

MEFINILDVDIVESNLTVAQVNRENHELTSENKMQLFFLIALVCIILIIAYLNLKEK

>tr|F3ZIL8|F3ZIL8_9ACTN Putative transmembrane efflux protein OS=Streptomyces sp. Tu6071 OX=355249 GN=STTU_1482 PE=4 SV=1

MLRPATLIGLAATAGLLLATVYEAPDWTLFVFAAGIGCVPSVGSMVRSRWAALYRDEPRMLHTAYSFESVVDEVCFVLGPIISIGLCTAWFPEAGPLLAGCFLAVGVFWLSGQKATEPAVHPAGQHERGSAMRSVGLRVLVLTFVSTGVMFGAIDVVTVAFAEDRGHKAAASLVLAVYALGSCLAGAVFGLFHFTRPPARRWLIGVCLVGVSMIPLQLVGNLPFLAVALFFAGLTIAPTMTTTMALVEQHVPRAKLTEGMTWVSTGLAVGVALGSSVAGWVIDASGPKAGYAVPGVAGAAAVVVAFLGYHRLNRPVPERGGAQDEQRTGSAGGAGAGAGRDTAGGTGAQERAGGGAPERDLA

>tr|F8XUD2|F8XUD2_9PROT Plasma-membrane proton-efflux P-type ATPase, putative OS=Acidithiobacillus sp. GGI-221 OX=872330 GN=GGI1_18631 PE=4 SV=1

MPLFLSLGFLLFRSFVVTPLLVLLLLFANDFVTMSLAEDNVRPSPKPDRWDIHTLVFSSLVVAFAWLIYIFAVYGVGRSLGLPLASVQTLDFLGLVFSGLANVFLVRERGHLWASVPGRFLLWASLADILVVGGLAAMGWLMAPLPMPIIVGLLLATMVYTLILDQIKVPLLRRLTSA

>tr|G2IEB7|G2IEB7_9CLOT ABC-type Na+ efflux pump, permease component OS=Candidatus Arthromitus sp. SFB-rat-Yit OX=1041504 GN=NatB PE=4 SV=1

MNKFFIIAAYNFRDIIGRNFFKISTLVISLLIIIVSILPDLIIKFNFISKDKSEYLIYISDPKNYIFKDDLELNLYIRSIEKSLDNNYYVKLVDKGVKEDELKEKLYNGGIDGYIDVVSKSEINIFTKENYPEIKFILDRYILNQNSDLNISYPNYNVESLSLFKNKVIGIIKNYTYPFLLTIFIYMIFILYGQFISMNVNIERTSKIMDIFITKVKFSTIILGKLFGYLLAALIQLIYFIIILFLITGMMSNKYFPLIKEIIVFDGLFVLKYISYFVLGFMIYGLLFVFIGSVIDKIEELSLGIIPIVFLISIGYFLSMLNLQFPNNYFKNILVCIPFFAPFVVITESSFILYKDIFASLIMLITIVILIFINISINKQVIKFRGTNLKKNK

>tr|G2N9R5|G2N9R5_STREK Putative threonine efflux protein OS=Streptomyces sp. (strain SirexAA-E / ActE) OX=862751 GN=SACTE_0027 PE=4 SV=1

MRALNSPLEVGVRALVLLAASFPRPLDLPQLVYLDYAMLHSGEMDGPPSVHPSLPAGPGELAMKRQLLEQGLVVLMRAGLADVQADDSGLMYRASEEGPGFLDLLEAPYVGALRERAQWALAFHHRVPADSRAATDDITQRWMGTFENRLPRQGGDDD

>tr|G7M0I8|G7M0I8_9CLOT Auxin Efflux Carrier OS=Clostridium sp. DL-VIII OX=641107 GN=CDLVIII_2401 PE=4 SV=1

MIISQMLLLFGLMAIGYLAKITKIMDTISDKYFSKFITNIAIPATILSSAIGQNIEDKIGIFKVLLVAILVFVVTPFVSIFLVKILKLERTYELMLNYSNLAFMGIPIISSIYGKEYVFYVSIFMMVFNISLFSYGVSVLQKENNSGKMQLKSLINPGIISALAALVIFIFEISVPEFITNILKNIGSITTPLAMIIIGSTLAGVKINSIFTDHKIYIYTILKILVYPLLTWIILHSLISDPVITGITVILCGLPTAGNLSMLCADYNSNVELVSKGMFISTICSFITIPLLVIIF

>tr|G7WEL3|G7WEL3_DESOD Arabinose efflux permease family protein OS=Desulfosporosinus orientis (strain ATCC 19365 / DSM 765 / NCIMB 8382 / VKM B-1628) OX=768706 GN=Desor_1241 PE=4 SV=1

MSGNSKGTKTFFDGHPIGSAHKRFLIIASLAYVFDQMNVINFGFIGPILMKNYGWTMQQFANVNSFNMLGMFIGALFGGWLADKIGRKKGLLTCILIFSLSSLANAAFTNYNIFLIMRTITGFGTIGMVTIAMAYISEMMPSESRGKYQALSIAVGVCGMPISAILAKVVIPLSYNSWRSVFVLGGLGLVITVVGSFWLKESPRWLVAKGRLDEAAKVLNEIVPDAQLPLNAVELAKSNNSGYIETFRVMFSSAYGKRTATLFIVVFGATLGSFYLSNFYPSIHAQMGFSQAVVLNLAIYQLFLNPVGDYLVSFISDNGGRKTPITVIFSIFGCLFIIQGLCSTVLSISIMLLLKGLFVSAAMTITWTYLAESYPTHIRTTASGILFGSGRLAASFLLFTVPVVYESYGYFGVNLVNGLIYIIPGIVVLFIGDSTAKVSLEELSPSISMKETSI

>tr|G8R5H8|G8R5H8_OWEHD Putative threonine efflux protein OS=Owenweeksia hongkongensis (strain DSM 17368 / CIP 108786 / JCM 12287 / NRRL B-23963 / UST20020801) OX=926562 GN=Oweho_2279 PE=4 SV=1

MLEVILYAVTLGITLSFAAGPVFFVIIETSISQGKTKALMLDLGAALADVIFILIAFYGSQSLISSLEDNIWVSLIGGLAVIVFGGYYILKSKTPGQFKNRVAVKRKRLFFFKGFLLNFLNVGVLFYWIATTVAIGSLVHHERSKMIMVYALIMLTYLTVDMFKIYFANKFKERFKGRNLQMVEKIIGLILLLFGVYIVIRAFL

>tr|G8UI28|G8UI28_TANFA Efflux transporter, HAE3 family OS=Tannerella forsythia (strain ATCC 43037 / JCM 10827 / FDC 338) OX=203275 GN=BFO_3188 PE=4 SV=1

MNRIAEFIVKKRLAIITAGVAVTLILGYFSTKLTINSNFMSYLPDDDRKVMLFERTDSLYATGNIIVIGMSNTENTIITPEGISVIRQVTDSISEIEGVEKVTGLTNVIDIRHRNDGVEIGRLMDEEIIPDSLPSLQSYILNKEMYRGRLLSEDGRSTAIIVFVETAADKEKAAREVNDLLDEVRRSHPELTVYCDGLPMQQQSLTESTKKDLIRLVPLVCLLIALILASTLHSLRGIFLPLLSVAMGSIWSMGAMGLFHVQLSPISGSIPVVLFAVGSAYTIHVLNFFKLLENGENHKGIVVRGITIIGVPVMLAGLTTIVGFLSFIPGTYLSIIRDFGIFMALGTFFCLLLSLTFIPAVESYLPPLKHQEKKQKKHVLSGVLQWLAGVSIHRNKMVLYCAGGLILLMGAGLFRLKSNIDVLYYFPEKHPLRQSAAFLNREFGGTLPVQIKVTADLRRSETLAAMQDFEDFLSRLPHVHNPQSVTELIKEMNQAMGEGKQIPDTQEKIQNLWFLLEGEPVMEQLCNTDKSEGMIHATMCNAPTGDYHAVSRKIDEYAAAHSSGTVQFETTGLPSIYSNFDYNLMQNLFWSLLLACILVFICMTFLVKSLKSALVGFVPLMSAILFIFGLMGYLGIALNLATVLIAGVAVGIGVDYSIHFISGYRNALVSGKECNEAVIQTLQTSGKGILFNVTAVAFGFLVLVFADLVPLKEFGLIMFATMFVSGLAATLLLPSIILCFHINLNKTQKQ

>tr|H0UME5|H0UME5_9BACT Arabinose efflux permease family protein OS=Jonquetella anthropi DSM 22815 OX=885272 GN=JonanDRAFT_0192 PE=4 SV=1

MTPPETEAASSRRLRRSALAFIVLMGVVSMFSDMTHEGGKSILGAYLTLTGASAAAVGFISGFGELAGYSLRYLTGRLADRTKWYWTLTILGYAIDLFAVPALALVPENGWLWAAALLIVERGGKALKKPAKDTLLSFVASQNGVGKSFALQEFLDQLGAFLGPVILFAVMARSSAVSLAAYRRCFALLIFPALVTLALLFVARYLFPTPENFEPESRAENFSRFGWGRRFTLYIAGISLFSLGFMDFPLITMHAAKTNLLTPGELPLLYAGAMAVDAFAALFFGWLYDRWDTKALVISTLLTAPFGFFVFLAPGQWALWVGASLWGIGMGAQESVLKAAVARLVPKARRSSGYGTFQTAFGVCLFLGSWFMGWLYERSLWEMVLFSVAAQVLAAILFLLSGRTSTGRLSAEEQ

>tr|H5Y5H2|H5Y5H2_9FIRM Arabinose efflux permease family protein OS=Desulfosporosinus youngiae DSM 17734 OX=768710 GN=DesyoDRAFT_3554 PE=4 SV=1

MNWKRTNLVTAIMLVMFLAAVEGTIITMAMPTIAKELQGFELISLVFSVYLLTSAISTPIYGKLADLYGRKYVLSISILLFLAGSFLCGLSQSMVMLIAFRAVQGLGAGGIFTVSYTIIGDEFPLEERSKIQGGLSTVWGVATLVGPFLGGFLIDLLSWHWIFFINIPFGLVAVVLLQRSLQETFEKKKQSIDYGGIITLSLAVIALLSIFIFDQNSNSQAYPLFAGTAVTIAILMMLLFYKIEKKAKEPILPFGIFTKTSTIVNLLSFLIFAVLMGIDVYIPLYLQNVLGYRPTISGLAMLPMSVSWLIVSIILGKLLVKYGGKAVTVTANVVILIGALLLTTLGTASPILLVLIYCFVLGIGFGGASTVLTIIIQDSVDYHQRGSAVGANSLLRTLGQTIGISVFGNIFNSHITGYFINQGIEGVNSSNLYQPSPSDLALTSEQISLALNSSMHVLFIAFVIISGLSLILSMAMPGRKEKEANCGINPLQH

>tr|H8XNB6|H8XNB6_FLAIG Probable membrane fusion efflux lipoprotein OS=Flavobacterium indicum (strain DSM 17447 / CIP 109464 / GPTSA100-9) OX=1094466 GN=KQS_00090 PE=4 SV=1

MKNTVLFISTFFLIACSSKEEIHPKKETIKELVFASGTLEWDNAYNLTAQTDGVLKNVTFDVGSTVQINERIATIDNPTNENNTESNRKLTSISKENLTAASPALQQLKQNIQFAESKYQQDLNQANRYKRLYESQSVAKVEYENMRLAAENSLSQWNALKKQYEQLLQQAKQSNINSENQLKNSQVQLSYNQVTVPQTGTVVKKVKDAGDYVKKGEVIAVIADPKKIECVLNVDENSIAKVKIGQVVFIQLNTNKQAVFEGEISEILSAYDEQSQSFICKAIFKKPLPSSLFGTQLEANILIGEKKNALLIPRKYLGYGNKVRVKGKEEPVVVKTGIVSTDYVEIVAGLTTEDVVLPIIP

>tr|I1DNN8|I1DNN8_9PROT Putative threonine efflux protein OS=Campylobacter concisus UNSWCD OX=929793 GN=UNSWCD_1004 PE=4 SV=1

MNFLLFFITLAPISLMPGINMTYAMSIGMSFGYKHSFFVMAGQLLAIAFVSFSCMLGVGAVLHHFEYAFKALNIIAGLYMLYLGVMLFFGKGELSITNVSNLPSKKQMFINGLIVSVSNPKAWIFFSALLPTFLDKDDPFSLTRMCVITVTLVFVEFCALNIYALGGAMLKKFLQTHLRLLEICTAIIVCTIGVLLLFR

>tr|I2IC87|I2IC87_9BURK Arabinose efflux permease family protein OS=Burkholderia sp. Ch1-1 OX=243261 GN=BCh11DRAFT_03728 PE=4 SV=1

MSEKPATAATNVIEVERVLAETHHPAFQLMLLVLCGLCLVIDGFDAQAMGYVAPSVIGEWHVSKAALGPVFSASLFGMLLGALGLSVLADRVGRRPVLIGSTFFFALAMLATPFVTTIPALIALRFITGLGLGCIMPNAMALVGEFSTPVHRVKRMMLVSCGFTVGAALGGFISAALIPAYGWRSVFWVGGAAPLLLALAMLVVLPESLQFLVLKGHNERALRWLAKFNPMLPIDANTRLVVREKGNGGAPVAELFRAGRGPVTLILWAISFMNLIDLYFLSNWLPTVMRDAGYSPSTAVLVGTVLQTGGVVGTLLLGWFIERFGFVRVLFVCFAGAALAVGTIGTVAHMLPWLLIVVFAGGFCVVGGQPAVNALAGHFYPTSLRSTGIGWSLGIGRIGSVIGPLIGGQLIALNWSNASLFHAAAVPVLCSALLVIGLAAATRQRGRPSEPRTA

>tr|I3CIB6|I3CIB6_9GAMM Arabinose efflux permease family protein OS=Beggiatoa alba B18LD OX=395493 GN=BegalDRAFT_2516 PE=4 SV=1

MQFQNILIIFYITVLSFCALYAPQPLLPLLSQHFQISTDQVSLLISVALIPLGVAPILYGFILESMAATRLLKITIFLLALGQLPFILIDNFWVLVGFRTLEGLLFPAIFTALVTYISTVSTLETIKRNIALYVAATVLGGFLGRMLSGVMATYFHWTDAFLIIMLGLLLGFGLLYRLQSDTRLQLVRPTWGLLYKALQHPIYNKVYLIIFLVFFCFASVLNFLPFRMTELDSQVSSLRIALTYTGYLVGIMMALNALRIIRWCSGEMNAILLGLAFYMLSLILLAIPSLIVISLTLFLFCAGMFLMHSVLSGYLNHLAVENKGIINGLYIASYYAGGSTGSYLPVFIYKNWGWIAYLSCLLGLVFIIMYITLLLKRVQHNLA

>tr|I3YNM7|I3YNM7_ALIFI Arabinose efflux permease family protein OS=Alistipes finegoldii (strain DSM 17242 / JCM 16770 / AHN 2437 / CCUG 46020 / CIP 107999) OX=679935 GN=Alfi_2316 PE=4 SV=1

MSANSRKVSPLAWVPTVYFAMGLPFIIVNMVATLMFRGLGIDDARITLWTSLIILPWSLKPFWSPLMEMFRTKKFWVVATQLVSGLGLALVALSLPLPNFFPYAIALMAVVAFSGATHDIATDGVYITELSKDLQAKFIGWQGAFYNIAKVFAMGGLVYLAGALKDHVGIVQAWMTVMGLCGGILFLLGLYHIRMLPSGGAATAHADSFGGAMRETKRIFLEFFKKKYIWIYFAFILFYRFAEGLVIKIVPLFLNAPLDQQGMGLTEQQIGLYYGTFGVIAFVVGSILGGYFISWLKLRRALFPLVCIFNVPFVVYALLAWFQPSSPVLICAAIVFEYFSYGFGFVGLTLFIMQQVAPGPHQMAHYAFGSSLANLGVMLPGMISGWLCDSLGGYHYFFMWALLATVPAFLLAARIPFTHPDTEEVTAEEIDKELINE

>tr|I4AES7|I4AES7_DESDJ Arabinose efflux permease family protein OS=Desulfitobacterium dehalogenans (strain ATCC 51507 / DSM 9161 / JW/IU-DC1) OX=756499 GN=Desde_4201 PE=4 SV=1

MVDNKPMTSTRYMDEATMNKSHYKFLILLAFGYTFEQIDVFSFSFVAPALTKYWGVSMEWIGLVNSCTFVGMLLGCWLGGWFADRIGRRKTFLGSILLFSLCSLVNGGAPNQEIFLVARTLTGIGMMGMVVVAMVYIAELLPAASRGKWQAIALATALLSIPLIGQLASHIIPNNPEGWRWILYIGGLGFIVLAFGNNWLKESPRWLISKGRFKEAEAVIQFYRPDVKVDLSAEASGKVKEEKAQETTKTLEVLRLLFSKEYRKKTLVLINLVVWNTVGYFMFFAWMPTLLNEYGFSLEDSLWMVALVSFGSPIGNYLAAFFTDKGGRKVPIVIYGAIIGVLTVIFGTIKAPMLIVGIGFIIRILMDGVFVLMWSYLAEAYPTQFRSSGTGIIFSTGRILNVGAMAMVPLIYKQFGYSVLFAIIGAMYIMIAVVTGIWGERTAGRSLEEIAETDSNKTVSA

>tr|I4CDL2|I4CDL2_DESTA Arabinose efflux permease family protein OS=Desulfomonile tiedjei (strain ATCC 49306 / DSM 6799 / DCB-1) OX=706587 GN=Desti_5043 PE=4 SV=1

MANLLCNTQFLLIAATNLCLFLVVSTWSFLPIVIVELGGNSIDVGLVMGSIGVTSLAALPFIAPLIDSWGRKTFIVGGILVIGLTNALFMLFDSYSPLMIFIRLLQGAAFAACFNGCATAVVDIVPPDRRAQGIGLFGISGSLAVSVGPYLGELFLIHWGRTAYFSLLIAFGLTGFFTALLMRSTEKRTSQKKIQGFFLTALNDGHIGPMLMAAVFGSGFAAMNTFFPLLAKSLGIQAGLFFVFYGISLLSVRIFAGQLVDKVNRDRLILACLVGFGVLLVSTSQLAVRYETILLGSFFGILQGLSYPSMMARMVDRAGEHNRAVVVSLFTGSFGVGLNVSVLAWGVIADSNGLQFMFLMGGLAVFAYAFIALCAYMVSPAAESALPERAILETERD

>tr|I4EUA4|I4EUA4_9ACTN Putative Nickel/cobalt efflux system OS=Modestobacter marinus OX=477641 GN=MODMU_1521 PE=4 SV=1

MTGVGVLAVGLLLCFAGLASIHLAVLASGFALGWLLAESLGGSLGVISIVALCAAVVAWVLATLVFRAALLVVGGVAGGVIGAKLFGLLEGDDGNVLLAVLFTLAVAVLAGLAAQHLHDTALVWICAFGGAGLALSGAARAWPDGLEFLRTPDTTAETVIAAAAWLALGAVGWSVQRRWASRRDQSRSA

>tr|J0UTS9|J0UTS9_RHIL1 Arabinose efflux permease family protein OS=Rhizobium leguminosarum bv. viciae (strain WSM1455) OX=755176 GN=Rleg5DRAFT_0772 PE=4 SV=1

MRAIDVSEAIDNNPVGRFQWTVVGLCALLLIVDGYDVFIAGTVLPTLMAEWQLSKPEAGALQAWALFGMMFGALIFGPLADKIGRKKGIAISFMLFTIATLSTGFASTPTEFKIFRFIAGLGCGGLMPNAVALMNEYAPRRLRGTMVALMFSGYSVGGMVAAALGIGLIPHFGWQPMFYIAAVPLLMLPVILWKLPESLGFLIRQDRQEQAKRIYAKIAPSVHLSANDKLTFTETTGAAASIAELFRHQRALRTAMLWVAFFCCLLLVYLLSSWLPKVLQEAGYAERASLLSLFSLNFGGMAGAIAGGWLGDRFGLPKVVVGFFAAAAVSIALIGFNLPAGMLFMMVFIAGATTIGTQILLYASVAQLYNLSVRSTGLGWASGVGRIGAIVGPTLGGLLLAKEFPLQQNFLIFAIPAVISAAAMLVFAVSNARRVSAVSLAAA

>tr|J1I5I4|J1I5I4_9BACT Putative threonine efflux protein OS=Saprospira grandis DSM 2844 OX=694433 GN=SapgrDRAFT_2374 PE=4 SV=1

MLTQAILEGLGLGLLLSIMTGPIFFTILQVSIEKGSRSGIALVAGQWISDFIYIGISSYLAKFLISWTKESELGQDLEFYLSIGGGAFLILLGLLLLFSPLPKAKTKEKPLSNKQAGQYFLQGFLINSLTPFPLFFWFTSIGTAYSRGYSQTDLVFFGVAIMLMVILTDFLKVFLASRLRQLLNELWLKRIRWVASFGLIISGLLFWLRLLWLS

>tr|J2HTK7|J2HTK7_9BACL Arabinose efflux permease family protein OS=Brevibacillus sp. CF112 OX=1144311 GN=PMI08_01031 PE=4 SV=1

MKMRLQPLQALYFTQFLSAFADNMILFVIANLLRENGFSPAMLALVSISFFLPYVFLAPLVGPFADKHAKSFVLVIGNLIKALGVVLLFVIDHSSILLLMLCYFTVGVGAVVYSPAKYGILPELTRNEQELFQANARIEAYTIVAILTGIGGGGAIASMTAPLFSSSICLALYLLSLGMTFFIPRIRGNASIRYGTEARRFFIDFQHLMNRAETSFALIGTGAFWMSSAVLRVAVLAWIPAALGINPQSFSVSLILATTSIGIIIGAFLAPRLIPLSRFTRSVGYGFGMFLIIVLFPWIHVTVIAICLLLLVGFMGGVFIIPMNTILQDEGKKMVGSGKTIAIQNFIENFLMAAGSGIYYLVVFLGASISVAIVAQGLLLLAFLLYLMKHRSRIAG

>tr|J2TZ31|J2TZ31_9BURK Arabinose efflux permease family protein OS=Polaromonas sp. CF318 OX=1144318 GN=PMI15_02290 PE=4 SV=1

MNTMPPILYFFALCNLVIGSGAFVLGGILQPMSASLGISVAAAGQAMTAYAVATAVLAPLLIILTARWPRKRAVQLALALFTAGCLVCALAPNLPLLLLGRVLMGAGAMFTAAASALAVSMVVPALRGRALSITFLGMSISYAVGLPIGAWLGFEFGWRVPVWLSAAASGAALAAASWLIPANMASAGTSFAGFQAAARQGAVLRVWGRTLLYFIAIFSVFAYVGPVLHALNPMNSAQLSATLAVFGLAGVGGTLMGGWATDRFGALRTMRVQLAVLVAMMCLLPLTRGSVPATMAVLVLWGIAGFGLMAPQQSRLASLSPAQAPLLLSLNGSMLYVGTALGAVISGALLDHVGFAQLGWVGVPFGLLAMLTLVFDRMPARVPASAAA

>tr|J2ZNN3|J2ZNN3_9CAUL Arabinose efflux permease family protein OS=Caulobacter sp. AP07 OX=1144304 GN=PMI01_05344 PE=4 SV=1

MSSASRLGLFYVVSYLGTGVSLPFIATYFHARGLSGAQIGLILALPMLIRPFTGPALAVWADGFALRRTPMAWLALGAGAGYIAMLAAPGFSTLLLCWLVGMTCLTTLTPLVDVIALRRSRIEGFNYGLPRGAGSAAFIVGNLAMGALLTVAAPSIIPIWITVAVLGCALIAATVVPPDRVHETEVAPDKAARWKGLGALLRDRTFLLAVVTVGLIQGTHAFYYGFSTLLWRREGISEPMIGVLWGVGVAVEVGFMWFAEPWRRKVGPERLLVLGGAAAAIRWTALAFAPPLWLLFPLQALHALTFAASFMASLRLIERLAPPQSASAAQAINSALSAGFMLGVATLASGPLFDAFGVKGYLVMAAMGGLGLIGALRLTRLAPRSGFKGQIS

>tr|J3BM71|J3BM71_9RHIZ Arabinose efflux permease family protein OS=Rhizobium sp. CF122 OX=1144312 GN=PMI09_03320 PE=4 SV=1

MTHHRQKFRTLAAEAAMILGGFIFGTSEFAAMGLLPEMAKANGVAIDVAGASVTSYALGVVFGAPLIAIVSARTPRHLSILILLAIGAVGNILTSLTGNFPMLIVARFVSGLPHGAYFGIAALIAAAMAGHGRRAQAVARVMMGLSVANLLGSPIATFIGEGTNWRIPYFLIGAVALVAALGCHLTVPKMPAAEGSGAAKEMGALARPQVWLTFAIGSLGLSGLFAVYTYLVPTLISVTGIGEQKAPLFLVIIGCGMVVGNFFGGWLADKGVMRAIGLLLALNVIAFALFLVSVHSAVLIAGAAFLAGFSALALVAPLQARLMDVAGHAQSLGAMLNGCAINVANAVGASLGGALITTKFGPASTGVVGVGLGIAALVVFSVSLKIERHHHKRLSLG

>tr|J3EEW6|J3EEW6_9PSED Arabinose efflux permease family protein OS=Pseudomonas sp. GM21 OX=1144325 GN=PMI22_05569 PE=4 SV=1

MRHSDSRVILNFNYPDDHNMNNQIISPSMDVNLSVADTDAGHTAINKKAIAAAVAGNALEFYDFVIYAYFAIYIGKAFFPVAGEYGSLMAAVATFGVGFFARPLGGILIGAYADRAGRKPAMILTVAMITIGTIGLAMTPSYQSIGIAAPIIVVICRLIQGLALGGEVGPATSLLIEAAPPHRRGFYSSWQIASQGIAVAVGGVLGVALSYWLSAEQLETWGWRIPFFLSLVLIPIVIYMRRALPETHETAQERTSSEIVGVVLREHKKVLLLGILLFASIGVASQIGNYMVSYAVQVLKLSAPVAQGSVLVGGLVTFAFALIGGLLSDRLGRRITNFIPRVILTLAIVPLFMWLVSAPTLLTLFTVNTVIAALTAMFATAGLVQIPELLPIAVRSTGLSLVYAFGTAIFGGTTQFVVTWLIAVTNSPMAPAWYLAGVSVVSLLAMLFLPESKNVDIRK

>tr|K0EDU3|K0EDU3_ALTMB Putative efflux pump protein OS=Alteromonas macleodii (strain Balearic Sea AD45) OX=1004787 GN=AMBAS45_05530 PE=4 SV=1

MSQRTPDQEFARYIKLALVAFAICFIYFIIADIRMPMTPQARVYHQVTQISPQINGPINEVLVKNNQTVEEGQVLFVIDETPYLIALEKAQIDLANVELENAQIDAKAEAIRAKIEAASAVYDERNSEFQRLNSLVQTKAVSQQNVDKAYAQLKSAEAEISALEADLNQQVVARGKQGDKNLKYLEAVNAVKRAELNLAYTKIKAPHDGIMANMQVTQGTYAKQSTPLASVVDNTLDVVADFREKSLANVKEGIRANVVFDALPGKVFEAKVEEFEAGVSDGQLAANGTLASVEKSNRWVRDAQRQRVHIKLEEQEISLSHLTSGARATVQIVPDNPIESVAANIQIHFISLLHFIY

>tr|K6PMY5|K6PMY5_9FIRM Arabinose efflux permease family protein OS=Thermaerobacter subterraneus DSM 13965 OX=867903 GN=ThesuDRAFT_02000 PE=4 SV=1

MLKGALGYLVLLSLAHLVTDLNQGGMPALLPQVKESYGLTYAQLGVVMLVLNITSSLIQPVFGYWSDKRPQGWLVVAGPLLAALGLGLVGYARSYEGVLLAAILCGIGIALFHPEGARAARGVSGGQRATAMSIFSVGGNLGFALGPVAAAMVVGLWGPEGLGWLVLPAVVLAAAMVAALPGMKRLEQEAALHEAARRAAAAAGPAGNRHGAPAGDRQGHPQPPQDGPSVAGETNWLAELLLIGVVGTRSWLQFGVLTLMPFLYLEKAGPDGVSTGVLLFVFLVAGAVGTLVGGPLADRIGTRRVLLGSMAVLIPLHWGLVHGPAWATLPLLAATGFALVATFSITLVMSQDFMPRYVAVASGLNTGFSIGLGGIGAAALGALADRWGLETTLSAMVLLPVIGLLLTLLVPVPERDRRQQLEAAGRPRPAET

>tr|K7SFD5|K7SFD5_GLUOY Transmembrane efflux protein OS=Gluconobacter oxydans H24 OX=1224746 GN=B932_0297 PE=4 SV=1

MVGGFSPIPDLFLMGFIEPVDVERVRPPHGVEPAMFALMLATFTIGTGEFAMMGLLPEFSHSLGISISRASSVISAYALGVVVGAPLIAVAGAKLPRRTLLLAMLVLFLVGNIGTILMPNLLDIEVMRFITGLPHGAFFGVSALVGASMVERARRGRAVGRVLSGIMFSTVVGSPLSTYAANHLGWRAAYGAISVLGLLCFLALWYFAPRDKPHPDANALAELGAFKRPQVLLTLLTSAIGFGGLFEVYTFLTTALSDVTHLPDWAVALYQIVWGLGMVAGNSFGGAMADRNINRTILFSLAASCVFMLGFWLLLPSPIAMLLITFLIPATLIGISPAIQTHLMEVAGDAQTLAASLNNSAFNIANAAGTWLGAFLVSSGLGLASIGWGSALLSAGGFLAYLITMAQARLSASR

>tr|K9P6Y1|K9P6Y1_CYAGP Arabinose efflux permease family protein OS=Cyanobium gracile (strain ATCC 27147 / PCC 6307) OX=292564 GN=Cyagr_2041 PE=4 SV=1

MDQVNAAAQRPGEAPPANPDRWWTLAAVECGNFVVYMDGFIVTLALPAMARQFGVGLPVLKWVIVAYLLTVTVTLLPAGRLADIWGRRRIVVIGMGVLVVTSVLCALAPTVEILIGFRVLQGVGGGLVLANVMAEITAVFPKQERRKAMAVNASILALAQVTGLVLGGLLIGQFGWRSLFLVILTVSLAGLILSLWILKARPRSQDRTAMDWTGAVLAVVATSAPFLVIEQLSQDGLNPASVAILMGGAAALALFVGVEQRLAKPLLTLSLFRSRAFSFGSVAAAFYFVAAVACYFLLPLYAQLVLGRTPVMSGVLIVPLSLVLTATSLTVSSLGDRVGARTLSTAGMLCVSAGLVGLSWLGADAAVASIIWPLVLLGMGGGLFHPPNNSATLNTVPAQHLSVANGFLSMARNFGQAIGTALAASLLAHGLGAAGADATLAGEVGARLGGSQLEAFLGAQQLAFRLAAALGLVGALISVSRGAEAPAAQ

>tr|K9SX29|K9SX29_9SYNE Arabinose efflux permease family protein OS=Synechococcus sp. PCC 7502 OX=1173263 GN=Syn7502_03334 PE=4 SV=1

MLLLNRYQWTVLFAAWLGWGFDIFDGLLFNYVAPNCVPTLLGLTIGSQPAKTATLFWTGILTSVLLVGWAGGGIIFGYIADRIGRSKTLLLTMVLYALGTACCAFAPNIWILMTCRVIASLGIGGEWAAGASMVAEVVPEKSRVAAGALLYTSAPAGLLLATFVNYQIAGVILAGSPEISWRFVFLTGLIPAVIAFVMRLFVKESERWKNTVANTKPPKLVELFNRQNLPLTISGFLMAVTALLTWWSCNAFIPVVATGLARTAAVDQGLSQTATFALVEHWKVIATNSFNLGGFIGTLLTIPAAKYLGRKRMFSTYFILSSAAILMTFGLPLPPEVRLYMYFAIGLTVFGIFGSFTYYLPELFPTRLRATGSGFCYNTGRLIAAIGPFLVGTIASRGENALSSSLQVLFCIGFVPLIGLAFMPWVIETKDRVLADFEPIS

>tr|K9UQ14|K9UQ14_CHAP6 Arabinose efflux permease family protein OS=Chamaesiphon minutus (strain ATCC 27169 / PCC 6605) OX=1173020 GN=Cha6605_6061 PE=4 SV=1

MIKKLSLLLVCLFVVTIGLGVSLPVLPFFFRHLHSAAVPRETIVIHTTFLTSIYAFVQLIASPFWGQWSDRVGRRPLILLGIAGSAVAQVLFGLASSVAMLYVVRALGGFLSAAMLPAATAYVADITTDRDRAKGMALVGTASSLGAIVGPAFGGLTTREDIHFTLGVVDLKIENYAPPFFLAAALMFLTLLVAFRWLPESLSSRSTSTVGVGKASRSRSVSERREASRNENRQPPLNWQRLGKPLLLLLGLTTISQFGLTLFEVVFALQAQDKLGYSPIQTGYVFMMCGGVMTVFQIVAVSFLTRYVSSIAQVGLGFTLMGSGIFLLLVARSLPIVLGVVAIMAFGMALITPNLIALISKRSSQHTGTVLGIQNTANSLGQVGGAMLGGVLFAWQFNAPYGFTGVLLVGTGLLLGWRQKDRLQRL

>tr|L0MMP8|L0MMP8_9GAMM Arabinose efflux permease family protein OS=Serratia sp. FGI94 OX=671990 GN=D781_3868 PE=4 SV=1

MPSRLKISTRLFLLIVLTGAFLGQFDLFVVNVAAPSVQRSLALSDGGLELVVAGYAFMFAALLITGGRLGDLYGYQRVYVFGMLGFSLTTLLCALSPNGVTLIIARLLQGGAAGLMIPQVLALLTTVLPAEERTPAMGWYGAATGLGSVLGQFAGGALVSWDFVALGWRWIFLVTVPLGMVMAGIAWRYLPPCVGSRRRKFDVPGTLGLASAFGLMIGAFLFYGHTRALMAAALLLCAGMLILVVTLCHEQRLIRQGGDPVVDLRLCRVASLWRGLLAVCLFMLYFSSFIFLLTNVLQRGLMLSPLLAGLVFVPSGLTFISSSLFFRRWAAAHQRLAILTGCGVSAVGLLLAAWGTLFVAAPVWFLLAAVVITGCGNGLILPVLIGFALRQVPSEQAGMGSALLSSAQQFASALGISAFGTLFYLLSASQGLVHAMGWCVLIQLACMALVALITGSGRAAAHHQAA

>tr|L7UBA5|L7UBA5_MYXSD Cation efflux system protein CusC OS=Myxococcus stipitatus (strain DSM 14675 / JCM 12634 / Mx s8) OX=1278073 GN=MYSTI_04892 PE=4 SV=1

MSPPASRLDAPRSLVVVLALLAPMAALAQAPLSAPEAQATPVSSLATDETLSKLLTEALEARPELRQVEAQEKAAQERVPQAGALPDPVLQVGIQNDGFGELMIGEMEGSYFSIMASQALPFPGKRDLRTQVARLGAKAVSAQVLRARLTIEAELRRAYLDLLMTRERRGLLDRLEAIWKQSADLARIRYETGDGAQSDLLRAQLELNRIRQRRVALNAEERTRVQTLNRLSGRPLDEPLPTTTRVRDLGIPELGEGEAAEKDAMERSPELAEGRANIAQSQQQMALARRERWPDFTVSAGVMPRGGDFPTMWQANVGVNLPIFSGSKQNRAVAESVAMADAATRATETVEQVLRLRVRERLTALSALRETATLYRSGLLMQSAATAESTLTQYRVGRASFASVLEANSGIVRDEEDFLSTLVEAQRLAIAQAEVSLEPVAALGGGSAGAGGMPGAGSAPSAPARGAALSGGAAGAAPSSASSSMSGM

>tr|L8MH74|L8MH74_9PSED Auxin Efflux Carrier OS=Pseudomonas furukawaii OX=1149133 GN=ppKF707_1405 PE=4 SV=1

MTNVFFNVILPILIVAAAGAGLKRWRNIPAAPFSQMMLYLLSPALVLDSLLNASLPLEATGRIVGAILLMSISLVAVSALLSRSLGHNRPMQSGFMLATAFPNAGNMGLPVALLAFGQEGLAVAVIIFASQAILGWSLGVFIAARSHNAGLGPLKQTLKLPVVWAIGLAFLLRVTDTTLPLALAQPLEMLGQASIPIMLLILGFQLEKGVALDRGASLLAALGLRLIGSAVLAYLVSELLGLEGVAQHTFIVMAAMPTAVFTIILATEFDAEPRFVSSQVIASSLLGFLTLTVLIMLLQSFGGSI

>tr|M1MYD9|M1MYD9_9CLOT Putative threonine efflux protein OS=Clostridium saccharoperbutylacetonicum N1-4(HMT) OX=931276 GN=Cspa_c58280 PE=4 SV=1

MISVFSVAQAIAVGFSYGFIASIPIGPSGLESVSRSISNGFREGFKVSLGAISADIVYIIIINLGIFTILSKNPKFESLFWIVSGIILVLSNKVSFKRKKTDHNLEKPILKNTYNAFLSGFLITFLNPTTPSIWIVSSSTIFTVWRHHGRIFFTLSILSMIIGSISWFCLLNILVSKGVKRFKSNIANHTSKFVNYLLFALGIVFIIFGTYKFIF

>tr|M3CD80|M3CD80_STRMB Arabinose efflux permease family protein OS=Streptomyces mobaraensis NBRC 13819 = DSM 40847 OX=1223523 GN=H340_03334 PE=4 SV=1

MKTAYLHRFVVARTVSMLGDRAAESALPIILLLVTDDPLVAGLVTASNILPALLFSLPVGHLADTRERRGLLITADVWRAVLGLGLALVLLAPEPSVALLVSITFLMGCGDVLFSVASHTYLPALVPSTRIMRANTALEAGDAAATLTGPALAGVLVSRFPHPVALVVNAGSFVVSAVLLARLPTARAPHADDHRVSSNCRTARRHGDVLAGFRLLISDPLQRILQLACIYMHLAAALFVLVIVAVSVQTLHIGPFRTGLVLSAAGVGGLIVTLLVTRFVEHLPWGLLLGTALCGLAATFLWLAMAQGFVSLFFAALCMDACSAFAFVTAGSVRQVFTPAKALGRLTAAAGLVSAAVRAAGVLSGGAVVAWAGGRTAAVVLGVIGLVCAVPLLMARTARRPIAADATPAS

>tr|M5EHB2|M5EHB2_9RHIZ Amino acid efflux protein OS=Mesorhizobium metallidurans STM 2683 OX=1297569 GN=MESS2_1080020 PE=4 SV=1

MSEVLTIFSILGVFLLGAMSPGPSFVVVSRIAISGERMDGLAAAIGMGIGGLIFASIAVAGLTALLVQVAWLDIGLRIAGGLYLLWIGIRIWRGATEPVEITSDASARPGSFRKALMRALLVQLANPKTAIFYASMFAAMLPASPPLWMLLVLPPLLFCNEFVWYAIVAFAFSSSRPRSIYLRAKHWIDRAAGAVVGALGVKLVIEGMGAARRAGG

>tr|M6D6U9|M6D6U9_9LEPT Transporter, auxin efflux carrier domain protein OS=Leptospira sp. B5-022 OX=1242992 GN=LEP1GSC192_0879 PE=4 SV=1

MSNFIVIGLCFLFGILIRSKGKFPSDSHKVINSFIISVSLPCMEFGPLRHASLDGNFLTFASMPWVLFGFGFLFFSLVGKLLHWKESTVVCLCLSAGLGNTSFLGIPLIESYYSKEGLPTVLIIDQLGTFLTLAIPGTYLGTRARHALQSSETKSSIWKTLFTFPPFIALLVSLVSRPISVPPELESAIARIGDTLIPLALFSVGFQLPGTIRVNSEEPNAENPKRESDNSFRMRIPLLFGLIFKLIIGPILVWLCFGTFFHYSEKNLDSNFLRNFKILIMESAMAPMITGSLLAAEWGLAPRLAVSLVGIGIPLSFLTTLGLYYLLENQAWTGTIFFGQ

>tr|Q0AAZ7|Q0AAZ7_ALKEH Auxin Efflux Carrier OS=Alkalilimnicola ehrlichii (strain ATCC BAA-1101 / DSM 17681 / MLHE-1) OX=187272 GN=Mlg_0636 PE=4 SV=1

MEVVLAVALPFFALIFTGMAAGRTRLLEGTSTRPLNTFVFYFALPALLLSGTAEMAAADILRPALFLSWLLPALLLFFTTWLGLRWLFGRSAGEGAIQALVATFGNVGFVGLPLVVTAMGTHVLPAAMVVIIVDSAIMIAVATAIIEWERDEGSGFRRALRTAGLGVARNPLVIASAVGVTLALLSLSLPAPLLRYLELLGAAAGPTALFALGITLARQPVRSAGPEVAILVAAKLLIHAPVVWLATWLLGLDGPLQTALVILATLPVAANVHVLAQRYGLYAGPTSTAILISTVLSMVTVSLALSLLL

>tr|Q0RJR1|Q0RJR1_FRAAA Putative transmembrane efflux protein OS=Frankia alni (strain ACN14a) OX=326424 GN=FRAAL3608 PE=4 SV=1

MLALQGVGAFAARSASRRLTSRLGTRTVVLAGLALAAAGTLPFGVTDGPADAVLLAAGLLVRGAGIGIVTVLTLAAAYHGLSRHEIGHASGASRILIQLGGALGVAGVTTLLAGQLGSPGAPTHVAFAHTFWLLIASILVGLLPALALPGRAAEDG

>tr|Q39DB1|Q39DB1_BURL3 AzlC-like efflux pump OS=Burkholderia lata (strain ATCC 17760 / DSM 23089 / LMG 22485 / NCIMB 9086 / R18194 / 383) OX=482957 GN=Bcep18194_A5961 PE=4 SV=1

MLARLSATDRFALIQGARDYSPTLMAIFSWGLVTGIAMSKSVMTLGQASAMSLLVYAGSSQLAVLPLLAAKLPIWTILLTAAMVNTRFVIFSAGLAPHFSYLPLWRRLAIGYFNGDVIYLLFQKQGFAYGHVPGKEAYFWGMALASWLSWQVSSLAGILLASFFPASWGLELAGTLALIPIMVSAVANRSTLAAVAVAGVVSLIAFDLPYRLALPLAVLAALAAGCTADFFVERADWRRIRTETVHEKEIE

>tr|Q83MR3|Q83MR3_TROWT Putative efflux protein OS=Tropheryma whipplei (strain Twist) OX=203267 GN=TWT_684 PE=4 SV=1

MYHGDVAGYVELFRWKSVTKVVVVQLFARFPIVIFPIALLLFVQKVGHSIFSAGIVLAVFSLAQAVFSPVIARFTTIWPHGNVLITCACIFSGIAISVSVLKVPFWLMTIMFALCGVVMPPTQSIMRTLYRHLVPLRLRSALFSVDTLLQELIWVVGPIFVTSVAVSLSPEVSMIFLGVVLLFSSIWLAFCKEIKNLKIPPARNVFGKVLKKPVVSAITVVSILFMGSCTAIELAVVATFRGGEGGHTSVAHLTGVVIAIWSLGSMLGGLAFGHKPIGRWSIPLRMLPFFVGVVVASLSNNVFWIAIWLFVCGLGLAPVVSASYSYVASVTNSAESPEAFGWIASGQLLGGSAFSALAGGIIDSNGAAWGFILSGAGAVAAALLAVAVNKLLPALPDRAPTAPIDLIL

>tr|Q8EA69|Q8EA69_SHEON 10 TMS drug/metabolite efflux pump (DME) family OS=Shewanella oneidensis (strain MR-1) OX=211586 GN=SO_4040 PE=4 SV=2

MNQSHSGPLANNHAQLGLLFISVAVLFWGMLPIALKLSGSFIDPVTLTWFRFLVALIVSILVQWSAGSLKQFAALDAKVWLRLILAGLFLMLNYVSFVYSLDYLAPGAAQLNFQTSPFFLAFGGVLFFKERLNAIQLSCFASLALGMLMFFHPFLDFSATDNHEIWLGVMIVQFSALSWTTYALLQKSLLNRLSPANVLLVIYALGIFAMAPFSDFSQFAQMNSFDWQVALFCAANTLIAYGCFGQSMKYWPTAQVSAMLALTPVFSFSATALVVSIGWWPEVFRADELDALSLFGIGVIIVSVMVVQLLPLYRQRRARRLQPI

>tr|R0ELW7|R0ELW7_CAUVI Arabinose efflux permease family protein OS=Caulobacter vibrioides OR37 OX=1292034 GN=OR37_01103 PE=4 SV=1

MSAEDTQARASRADDGWIGQRPSAARIAAFLAVGSIALIMAGVQPVVLGGLVTAGRLDVSQLGWSVTIEFLAIGLGVGLADALLPPRRLKLIGLTAALVLAAINFAAFEVSGLGVVVTRGLAGLAEGAIVWLTTLMIVRSPTPGRWSGVFLVSQAVLQVACAAGIPILVSPALGANAGFATLGASAALAGIIALVLPDRLAPLAGNRSDEPVLAEPIPKAAYVSLAAVFLIFSFFIGFLAYVERLAGQAGLTPVQGGLAVALALGASIAGSGLAAVLADKIAYHRALLVCAPIFLAVLVGLWGLPGSGVFFVLAGLHGLAWGFLQALQAPFVIESDPSRRAVLLAPSVQAVGAAAGPMLCSFFVTVQDARGVLVASGACLALSFVLAIALWVSRRRRQARSALAVV

>tr|R5DBC8|R5DBC8_9FIRM pH adaptation potassium efflux system protein B 1 sodium/hydrogen antiporter subunit OS=Firmicutes bacterium CAG:83 OX=1262992 GN=BN795_01396 PE=4 SV=1

MSTVFRLLLLAGLLVCAVATALVKKPLRAVIIYMAYSIIMSVIWILLEAPDLALTEAAVGAGITGILFFLTLRRIDRIDRDADVEETQGEEEPHEENEATH

>tr|R5HEV3|R5HEV3_9FIRM Macrolide-efflux protein OS=Firmicutes bacterium CAG:114 OX=1263001 GN=BN469_01521 PE=4 SV=1

MGVALLPLVSPIVGYGLITVLSFLVMGVSTLFTVTILAAMQGQTPPDLLGKVMATVLATANCAQPLGQAVYGLLFEGLADHAWAVMLGAGLLAACLALRARPVFWALEKETDRSAGDATVR

>tr|R5JFN5|R5JFN5_9FIRM ABC-type Na+ efflux pump permease component-like protein OS=Coprococcus sp. CAG:782 OX=1262863 GN=BN781_01587 PE=4 SV=1

MKNKMHGFKTVFFHNLGIHMKSKKYLAVTIILAVFLLAGISIFIMIMSDKNSRKDQYTYNVDKVYVVDETGLGVPDYSMYGAALGYEDAADTEFVTSDKSPEELVDTDGAQYVVVQKKSGDSFVLCVIFGNTDNVTDEQVDFNTAAMEEYLVPCFKTHLFQVSGLTGEQIVQVMLPTAINVSKIGGESEKQSKHIVAVIMMMAFVMIIYFAVLIYGQQICADVPMEKTSKLVEQIMMSVTPYALVSGKILAMVFASVIQFIIWIGCIVGGVLLGDVLSKTVLGVDTSAVTTALDLLRDWFDGMGFSGISIVLAVLLFIAGLVFYLMLAGLAGSALARPEDAANVQSIFIIPLVAAFMLVLFASGLTASGNYNIPLAYNLVPFSAAMTAPASVLIGTLSVPMAIVSLIISVVSGMIILYVAARLYKGMMFFNGKKAKLKDFISAIKG

>tr|R6GXK2|R6GXK2_9FIRM Auxin Efflux Carrier OS=Oscillibacter sp. CAG:241 OX=1262911 GN=BN557_00130 PE=4 SV=1

MLEILLYAGRAIMPLLLTMALGCWLRRSAHWSDDFYRQLNSFCFHVLLPVQLFLNVYAIEDLSVLNWRLLGFIVLCIVGAAGLGVAVAPLFARERAQRVVIAQATFRANQVIMGIPLASALGGQDALIFASLVTSVCVPVFNMLAVLMLTAYSTDGKSISWREEVRQIFRNPLILGALAGLVTVLVRQLLPQVDGQPVFSLRSSLPSIYKACSDLSKVASPLVLLILGARLRFDAVQGLWKKILAAVSMRLVVVPLIVLTLVLLLREPLGLTTVEMPTVVAIFCSPVAVTSAVMVQEIGGDEQLAQQVVAWSSVLSMGTIFCFAAALRAMGVM

>tr|R6PKZ7|R6PKZ7_9FIRM Putative cysteine/O-acetylserine efflux protein OS=Eubacterium sp. CAG:274 OX=1262888 GN=BN582_00080 PE=4 SV=1

MELSVIGTFLGYVVVSSFTPGPGNLLALNNVTSYGLKKSRNIILGICCGYGIVQFLCTLALYIVNRHISSVLFMLKYIGGAYICFLALHIMLSKKSEDVSVKSPSFKSGCLLQLVNVKIYFYIITLITTYFIPNFPTITGLTLAGIGVVAVGCSAIITWALVGVGLKNFFNKYYRIINVVMGVFLLYCAWDIIRSN

>tr|R6T8R8|R6T8R8_9STAP Cation efflux protein OS=Staphylococcus sp. CAG:324 OX=1262969 GN=BN609_00329 PE=4 SV=1

MSKQNYEFRALIIGVIINLISAIVGFIFFYLTTSMSILLDGLISAILCGSTIVSIFVSNYVNKNDSKKYPFGRYAIENVFLLFRAIMMLGTIIFTILDGALTILDFINNQTIDNFNASNWQLIVYGLSMCGLCLLITLVYSILNKKSQVKSEIIRIEIKASLYDGLVTLVAISSLLLFSNIEFLSGIKEIGDSITVIILSIIYLYSPLKELIGQIKILIDRRRFVETEKELINNLQHKFLIFKFNDLYFAFSGDHYQIYISLYPKQNLKSEEITKKFQDIKNHLLNIYQDSKIYLLLSKEMIHNM

>tr|R7ZLT2|R7ZLT2_9BACT Potassium efflux system KefA protein OS=Lunatimonas lonarensis OX=1232681 GN=ADIS_4539 PE=4 SV=1

MKSTGLTLLSLCCFLLFWSVPAQSQVFDSSVPFVVDSTDFNNLSTPYHTTLTFFYNLQEETFDPEVSGRALNMSRLTNKDASNLAVKLKQVFDGRGVYVRIGEVPVDANFIDTLRNGQMRYFFDEQRLPGVFLEKVGQAWLFSSFTVGQIEELHLETYPLGTDRLLNLLPKIGNEVYFGLHLWQLVGMFLLLLLIFISHQVFTIVVDRGVYYILMKAGYGKMAKKYLLPVARVVSIYLIVLLLAVFLRVLQLPILVISWVTVLLNAVKPLLVTIIFYKLVNLLSSYLESMAAKTVSTLDDQLVPLVRKTLKTFVIVVGTLFILKDGLQLDIVPFLTGLSIGGLAFALAAQDTIKNFFGSVMIFIDKPFQVGDWITSGDVDGTVEEVGFRSTRVRTFRNSLVYIPNGKIADATIDNHGLRKYRRFSTHITITYDTPPELIEVFVEGLREMVRRHPHTRKDFFHIYLNNLSAYSLDIMFYVFFEVPSWGEELKAREDMLMATIRLANQLGVRFAFPTQTLHMETFPEKKGLNPTYSDDREAYQKKLAQFISKESGTTGRI

>tr|R9GNB9|R9GNB9_9SPHI Potassium efflux system KefA protein OS=Arcticibacter svalbardensis MN12-7 OX=1150600 GN=ADIARSV_3416 PE=4 SV=1

MLYFKKQFSKYFFLFLCFILTCIASQSNAQGNLLLNDTAENVKQEPSWPNDTLGRRTPRGTVEGFIKAVSQEDYAKAALYLSIDSTLKRKQDRVLQAQGLQQLLDQKGNIFPYSMISDKEEGQQGDNLGENFDHIGDATVDKDKFEILLENTKGSDGGPIWLFSIQTIQRIPLQLDSVSSAPLLSKLAPKVLEENKWRGVPLAHWMAMLLIIIVAYLLALGITKAAIIVIPLFWHKARIEPVSGIITAFALPIRLYLAVWLFVIGSRQAGIYIIVRQRFSDITVVVLLVAVLLLIWQLVDFVSRYAERRLARHGNQAGVSAVLFLRRAAKIALVIMCVIMILSTFGFDVTTGLAALGIGGIALALGAQKTVENFVGSVTLIADQPVRVGDFCKVGDVVGTVEQIGMRSTRIRTLNRTIVTIPNGEFSSNMIENYAHRDRFWFHPTFGLRFETTPDQIRYLLVELRSVLYAHPKVDPSPARVRFVEIGSDSLKLEVFAYVHAVNFDQFLEIQEDLYLRMMDIITESGTGFAFPSQTLYLAKDHAPSPEKAEEIHEKVKKWREAGDMPIPAFNPDYIDDLKNTIPYPPEGSSVYKNDNSFKDEKS

>tr|R9PLK5|R9PLK5_AGAAL Potassium efflux system KefA protein OS=Agarivorans albus MKT 106 OX=1331007 GN=AALB_2329 PE=4 SV=1

MTLSIFSRGFLVSLLFFCLAAQANVGGESHLKGLISSLDTQPSNLNTQQQQLIYEESLALLKEGNSYREKAKYYRGIINNFSRLRKEQQEQLNQYQAPVIENLAELNSNQLNEQLTIWQAKLSNTNSQIDELKQRQYRIDLDVSENHQRSGPLREQLNTVRAKLEQLEFGVLNEVEEAERVRAQVNEASLTAQLAMLELAAQSANHRNELLQLEISLLQRTQLAEKRIVESVKNRLSLEHRAEAQRLSDQLAFAAPELLNDPIVKNLVEQNQLLQTELSALFEQSEEIADLQEEVNDELEHIKLTFANFKEQVSWLKVTRSYGEYLREQISSLPKFKAVAPIEQQIIEVRISKHRHQNLLYTQRNQSQRKAQREFIATLNEEQARIFNKHLKLNQQLSDKYLLELDARLFELARLKLDYSKLNSQLNTINQEANQELFWTADVKAIDLSFFSDIRYSLIWLFSAQQYSQLSDAIAKLSWLWLGWLLLVAASVAYIYYLKKHWLKGYLQRIDSKTGNITRDKFSYTLANLLVSAVFATPVALFIGSVGFALMYEWESSFARDLGDALFNVAIVLWLFHLLQQFCQKNGLLNTHFKWPLVNISQAITLIRRVVYVSLPLVFILNLCILQTSQPAYSGLARLAFIALLLWVAYGFHSIYRIELPVNYHLDIFKAPQLAKKLIWGAAISIPLMAALASAAGFFATAFTVYWQVVLSFIIAAVFLLGYLLIHRWMLLQRRRIAFDRAKVRRAEMIAQRQSEEDDSSSSNEGVIDSIEEPVIDLDTISAQSLGLLRAALVLSLVLVLLLSWSEMTSAFSFLDNITLWESSSSRAGVATVDAITLRSLGSALLVFFFAVVLIRNLPGLLELMVLQHLSLNPGTGFAITTMINYMVILFSIFSGFGLLGIEWSKLQWLVAALTVGLGFGLQEIFANFISGLIILFEKPIRIGDTVTIRELTGTISNIETRATTIVDWDRKEIIVPNKAFITEQFINWSLTDPITRVVLMVQVKQGSDNQLVNKLLQEAVDANSLVLANPAPEIYFTEYTNNGMKFEMRVHVSEMRYRLPMTHELYTLINDKFKANNIEIAYPQLDISLNP

>tr|S0EX62|S0EX62_CHTCT Arabinose efflux permease OS=Chthonomonas calidirosea (strain DSM 23976 / ICMP 18418 / T49) OX=1303518 GN=CCALI_00540 PE=4 SV=1

MRRQSKSGEMRRSPLLILALTLFIDMLGFGLILPLIPVYISHYGGGAWVGGMLLGCYSLMQFLTAPIWGRLSDRIGRRPVILIGLCGSAGTFLTFGLAPNLLVLFLARVAAGALTSASLPTAQAYIADVTPPEKRASGMAVLGIAFGLGFAFGPVVGGYASRIAIGSLSPIATPALLAAFLSFCNFLWALAMLPESLSLARREASANATTEKGPLALLRSIAAAFHEPNIRAQLLVFAFVTFAFTAVESSFSWLVILRFHHTLEQTAIRTWQTHHLGQPWASLPDIVRRHQFEKIEAIITSRIFLIVGLSSLVVQGFIVRGLAHFIGEHYLVRFGAMLMTLTLIGIGLTPSLWGIYLLSICIAIAMGVMTPSLNALITHAADPSEIGALSGVQQGLGSLARIIAPPINNYLIGLPNATGVPFFCSALLMAVAFLLSLQLKPMTPSSKRDKEATETTTIEASSIH

>tr|S5R3S5|S5R3S5_9PROT Neutral amino-acid efflux system efflux protein, homoserine/homoserine lactone efflux protein OS=Candidatus Profftella armatura OX=669502 GN=SSDC_00755 PE=4 SV=1

MIFKTWISFLLFSIITSISPGPGNILTINHALRYGWRKTLSLIIGQEIALVLIILAISEGAELLLSSSSILIFIKIFGIIWLMYTSFQMWCASINKKDSTYICETISKKKSFIRGFFTTITNIKAITCLISTLPACLTPIYPIVPQIIIMSLTMTIIDSSVMLLFAITSSYLRPFFQKSKNIKIQNRISSIFFLFIAISICFL

>tr|S7XEF2|S7XEF2_9MICO Potassium efflux system KefA protein OS=Leifsonia rubra CMS 76R OX=1348338 GN=ADILRU_0143 PE=4 SV=1

MFEWKSWLGLPVAVVIALLAVVIVVGVISLIVRGIARLRPGVYATLAPLRQRLRVLIALLAVWVAVAATIPIVEENILNIINYAFRGAVIASGGWLLVAVMNLLFGRAVARYPLDVPDNRVARRVRTQVQVLRRVLTAVIAIVTIGAILMTLPGAQALGASVLASAGLVGVVAGIAAQSALANVFAGMQLTFSDAIRVDDVVIADGEWGRIEEITLTYVVLNIWDQRRLVLPSTYFTSTPFQNWTRNATELLGVVVFDLDWRVNVDQMRVQLKKVLAETELWDGRTANVQVVDSTGGFVRVRIVASAADSGAQWDLQTHVREEMVSWLQTKNPAALPRTRVLMVENEARSRGKAAAEPQDRGLFSGSAEADARRQEFTGAITVQANTPDNTPDNATDNATDNATDNAPEGAADHPPKKD

>tr|T0TZF8|T0TZF8_9STRE Potassium efflux system KefA protein/Small-conductance mechanosensitive channel OS=Streptococcus sp. HSISM1 OX=1316408 GN=HSISM1_2086 PE=4 SV=1

MRVLIDIPLDANTDLDKIYQVIAQVNQSEQDKHPEVLTGPTILGPQIEKMVVIPSELR

>tr|U1GP90|U1GP90_9ACTN ABC-type Na+ efflux pump permease component-like protein OS=Cutibacterium granulosum DSM 20700 OX=1160719 GN=H641_00110 PE=4 SV=1

MSNNATSSARRDGVGSDGVAGTSSMWWTVARREMSVKIRDRSLLVSLIIVLVIVAVSVGVSLITSGTSDDKPTSVAVTDDAGAAIVAQAQKMAQQTGERQILETVRTTSLEQGREVVTDEKAKLVLHHEDGTWHLESGDEPPSVTSGAGQLIAKAVEARATADLAQKAGVDPQQAVADGTVLPGTVDPGQADNWRDTVATFMGVGFAVVYMFTMMFFGNGIAASVVEEKQSRIVEILLACIPARQLLAGKIIGNTILAAGLMLILLVLGCVGVSFTPAADLLGTIAAPTAWFIVFFIVGFLSLACLWAAAGALASRTEDLQSTSMPLVMIVMIAYVFGIQAAQNHGVSAVVASYVPVASAIAMPTRVATGGAAWWEVVVSILISVLFSALTIWGGERIYRRSVLKTGGKVKLRVAWRSTDVVA

>tr|U2EMX6|U2EMX6_9PROT Formate efflux transporter OS=Campylobacter concisus ATCC 51562 OX=1242969 GN=ATCC51562_934 PE=4 SV=1

MLNPAETAQAVSSSMEHKAHMPLTSIIFLAIMAGAAIAMGDIFWAHSTVGMAENQSIGLSNFIGGITFSCGLMMVVFYGGHLFTSSVLSGVSAYEGKLKLGNTIVYWAIVWIFNFVGGALIAYMYYYSGLPLKYDGYILQHFVPAAIGKITAPFHELFIRGIFCNVFVCMSIWTATSESNLSGKFFAIMWMIGAFVACSMEHCVANMFIITEAIISKAHYIAANGGDIAAAAAALGHGITAEKLEVLNWGNFIGKNLVPVTLGNICGGLFFVGLVGFMANKFDMKKKA

>tr|U2IL81|U2IL81_9BACT Efflux transporter, HAE3 family OS=Prevotella sp. F0091 OX=1227276 GN=HMPREF9148_02286 PE=4 SV=1

MKKLAEFVIRYRWAVIVFFLALTAFMGFQMKNASFNPDLLTYLPEHLPSRMNQKQIEKMFGGTDMVMIVVQTDDVVNGKTLKRVEHFSQDMQNIKGIERVMSVFELKNVRSENDAMTVDAAVKMIPRTAEDVATIKKELAGNDLVYGSVISKDFTTTAIIGLLEPGAKDKDVIDQVEAMIAKYPGTEKVLLGGSPYMRMQNAGMMQKDMARLIPLGLLLMMVFLFISFRQFRGVWLPILIVVMAIFTALGATPLLGWKFAVTTIILVVLLIATANSYGIHMFARYQRDNLPGNNYTAKELSVKMVTSLGAPIILSGLTTIAGLLCMLGHVLIPGGQMGVLGSIGIGLALIGSLFFIPALSSVLPKTKPRLRADNNPKSKRGIGLDRLLDFIADWVTKKPKTILALFVVISLIGAAGLLRFSINSNPAELFPDGHPAKESAQIINKELGGFFPLCVVFEGDIKDPALLKKIDDLEKKVREIPEVGTTQSIAKVTRQISRALYNKGEEGYDKIPDTYDAVSQYFELYLMSGSQKDLEKMVDFNFEKALLMIRFKELNTPVLRQCVAQIKEMVKDDPNVKLVGGNADVFTDMDKHVVSGQFLSLLISLVVVFIIISLGFKSFKAGLLQIVPLMFAMLMLFGLMGYFGIDLNFMTAFQASILIGVGVDYTIHVVWRYREERRAGYDDKEAVHRLFKATGRGIVFNAIAVIIGFVVLLFSGFLPVRFFGMMMVTIIFVCLIAAVLLVPALCMVLKPKFLRQKIHCK

>tr|U2YUG8|U2YUG8_9CAUL Potassium efflux system kefA protein / small-conductance mechanosensitive channel OS=Brevundimonas abyssalis TAR-001 OX=1391729 GN=MBEBAB_1381 PE=4 SV=1

MAVFARWSRMTELGSLQALEKIENAPVLRLAGTSITAGGLISAAIILTVALVLAWLATRGIKRLRARSSRSGGALYLLEKLVGYGLIVAGGMLALSATGLNLSSLAVFAGALGIGVGLGLQGVVKEFVSGIFLIFDRMVSVGDYVEIEGGARGAIMEIGPRATRIRTNDNINILVPNSQLIEHPLTNWTLKGDTRRIHIPFSVAYGADRGEVRDVVLAAARASPFTSPETDARKCQVWLVNFGESGLDFELLVWPTQDAVKRPAAMHAAYTWAIAEALEQAGIEIPFPQTDLRIRSLFGREGDEAMEVMTTGKAPRPKPAARKPKKKATTSSENDAAEDLLLPSAAEAPEPDNS

>tr|W0B7D0|W0B7D0_9GAMM Arabinose efflux permease OS=Legionella oakridgensis ATCC 33761 = DSM 21215 OX=1268635 GN=Loa_00891 PE=4 SV=1

MHQQKSLSSMLSLFLVLFIDGMGLGLLFPILNTILIDPQAGFLSPNLSMGLRDFLYGLTIGVFMICWFFGAAILGDLSDSVGRKKSLMICLVGSFLGYFISAIAILTHSFTLLLIGRIIAGFTAGSQPIAQAAIVDVSSEEQKARNIGLILLSVSLGFVFGPIFGGLLSNERLVSWFSFETPMYFAAGLSLFNAIFLHLTFRETFVKAHDKIQVRWHHAVNIFISAFKQASIKKYSVVLLIMIFGWSNYFSFISLYLLQTYQYSALENSFFLAVMGIGFSIGCGYLVNVCTRHFSLDGTVISGLLVTASLVMMTLLGKQQWVAWVATLLIGMSLSVAYSVLLTIFSNQVNDDQQGWVMGVTGSIMALCFGLTSIFTGIIAHVGAVLPMLLAALGLGGSATLLFLFKRGDFQRSRKEMLQP

>tr|W2UC14|W2UC14_9GAMM Threonine efflux protein OS=Gammaproteobacteria bacterium MOLA455 OX=1411685 GN=rhtC PE=4 SV=1

MDTVFWLSVTMACLLGAMSPGPSLAVIGSLTLNQGRLSGMIGAVAHGLAITAFALLTALGLVGLVSRYESAFNLLQLAGCLYLVWMATKLLFAAPNKAPNRAFDQDPGQSADTSNSVVGPKWAAARDGFLIALINPKIMLFFSALFSQFVSVDSAFWVKLVMAAIAGTVDALWYMLVAVVISRPGNLLRYQQTGPWLNKLFALLLLFIVAGFFVDLTA

>tr|W4BAJ7|W4BAJ7_9BACL Arabinose efflux permease OS=Paenibacillus sp. FSL R7-269 OX=1226755 GN=C162_27052 PE=4 SV=1

MKAQSGNSASAPRSKRNTVYIMQLVTIFLGFVVFGISENIKGPAIPRIQLSFNLDEGQLGTLLSLNALGYLIACSFTAVLVRKWGIKAVTIISFASMVLSGVLIYLSHSYPLFASSYFLMYIGNGMLEIGLAILGARIFVKNTGMMMNLSHFFYGLSSTVAPLLATGVMSLSVFGHLLDWRGMYLVMLSLCLLPILSALRSTYPGDDLPQEDRTSFRTLTRDPALWMMVMILSFGVVSELAVGGWLVNFLEKAYAWDTVRASGLLSAFFLVFSLGRLLLGALTDRIGFVLSLIIFSCFSAVCTFAALAGGERLAFLFALSGAGIAIIYPTVMAFIARRYPNGSDTAITFVVTLMGLGSVIGNYIIGWVIEAVKAFYGSTTELGLLRGLQAGYGFIGLCAAVCSLSGIVLYVYLKRRRELI

>tr|W7W546|W7W546_9BURK Cation efflux system protein CzcI OS=Methylibium sp. T29 OX=1430884 GN=czcI_1 PE=4 SV=1

MRRWVFAFLLLVVPFQFVWGSAAPYCAHEASVLAKKHFGHHEHKHQAGGEVTPAADNQGDAVGAYHADCESCHLGCSAAVQANAPGIHALPQARAPGCREPRYTSYVPSVPQRPDRVVSAPAARSGSGVVV

>tr|W7ZKE7|W7ZKE7_9BACI Periplasmic component of efflux system OS=Bacillus sp. JCM 19047 OX=1460641 GN=JCM19047_3608 PE=4 SV=1

MKSWKKGIIASLVVASVGTFTVYGMTKGKDLAVSSYEETFDLISPMYEDLSTVVMVPGSLELVNRQVVQPSTEQGSYQVLVEVGDEVEEGTPILQYSTTEIDFEIQDLELQIEQGQTTIRNLTASEAEITKRKNGPDVKPTYLEDEETGDRTEIEPLVTVAELDAELAELADQKKAENYAISRLQNQLETAKKQKGELTLTSTINGRVLSINDQGGNTDDLGNSLPLMEIADTTQFTITGNVSERQSLDVELGHVASIYSDTIEDGFWSGEVIDVSYFPTEGDDWYGDSSGSQYPVTIKITEGETENLRPGYQVMAEIVTSEEMGLTLDMELVQYDEVGSFVFVYEDGVAVRREIEIDYANDYSVKIIEGLTEEDLVIADYMQMVTEGMTITVSEFNEEEFYEEGFIEGEFEEGEFEEGEFEEGDFDEMDQEDETGELEEQEGDDEL

>tr|W8VRK0|W8VRK0_9FLAO Potassium efflux system KefA protein / Small-conductance mechanosensitive channel OS=Nonlabens marinus S1-08 OX=1454201 GN=NMS_2305 PE=4 SV=1

MQNNTTNKVDFQNQLTETLSHYYEELIETLPRLGLGLIIIILGFLIAGMISRFATRRARARTNDPLMSRFLGRSIRFLLIVAVIVLGLRVAGFGDISAGIFATAGASAVILGFAFKDIGQNFIAGIILSFNRPFNVNDTVEIGSNFGKVKSLEFRYTKLKTFDGKDVYIPNSDVITQPVTNYTEDGFFRWDFLVGLDYEDDINLAKETIMRSINEDPKVVSDEEHSSYVMEDELATSTVNLKVMFWVDTVDYGRVASETKGRVIGNVKRALMAEGFYLPADIQEIKLYGRETNIPLSLNDLKKSTND

>tr|X4ZQW1|X4ZQW1_9BACL Cysteine and O-acetylserine efflux permease OS=Paenibacillus sabinae T27 OX=1268072 GN=PSAB_23200 PE=4 SV=1

MTRQKSGVLLLAFLVLVWGINWPLSKIALAYAPPLLFSGIRTVIGGVLLILIALPKARLLRFKTLWPVYLGSALLSIALYYGVQTIGLQYVPAGLFSAIVFLQPVLLGIFSWLWLGEEMHGQKIGGLVLGFLGVACLSAGGLTGSISLLGILLALATALCWALGTVYMKRNAVRVDMLWMTAMQITLGGLILLVAGSAAEPWKAIRWSTDFVAVTLFISIFVIALGWLVYFKLIHEGEAGKVASYTFLVPLVSIGSSVLFLNEKITINLVIGLILVVISIILVNVRFRRSPASAVAEIRALEEGDYDF

>tr|X5MF18|X5MF18_9RHIZ pH adaptation potassium efflux system protein D 3 OS=Candidatus Phaeomarinobacter ectocarpi OX=1458461 GN=BN1012_Phect3075 PE=4 SV=1

MMEFIMYHLPALQVVVPMLAAPICLLMMRGSLAGLVALVTGVLCFVMSLLLLQQVIVSGPISYQLGGWAPPFGIEYRVDAMNAFVLVIVAATSALVLPFARRSIRAEIEPSKQALFYTVFTLCLTGLLGVTITGDAFNVFVFLEISSLSTYVLVAMGARRDRRALTAGYTYLVMGTIGATFYVIGLGLLYQATGTLNMEDLAVRLQPLGDLTSVRAGFAFIMVGLALKLAMFPIHAWLPNAYTYAPSVVSIFLAATSTKVAVYVLLRFMFTVFGYDFPVVELSLSTVFLPLAVIAMFVASAVAVFQTDFKRLLAYSSVAQIGYMVLGFSMASVTGLTATMVHLFNHAAMKGVMFMVAGAVVYRVGSTAVTSFAGLGRQMPWTMAAMVVGGLSLIGVPLTVGFISKWYLILGALETGDWIIAFMIVASSLIAVIYVWRMVEMAYLTPAPEGSKPVREAPLSMLLPMWTLALVCLYFGINAELTASIGQAVAETLLNGGVDAAASVIPMDEVVEGVAP

1. **Non-efflux antibiotic resistance (non-EAR): 189 protein sequences**

>sp|P62593|BLAT_ECOLX Beta-lactamase TEM OS=Escherichia coli OX=562 GN=bla PE=1 SV=1

MSIQHFRVALIPFFAAFCLPVFAHPETLVKVKDAEDQLGARVGYIELDLNSGKILESFRPEERFPMMSTFKVLLCGAVLSRVDAGQEQLGRRIHYSQNDLVEYSPVTEKHLTDGMTVRELCSAAITMSDNTAANLLLTTIGGPKELTAFLHNMGDHVTRLDRWEPELNEAIPNDERDTTMPAAMATTLRKLLTGELLTLASRQQLIDWMEADKVAGPLLRSALPAGWFIADKSGAGERGSRGIIAALGPDGKPSRIVVIYTTGSQATMDERNRQIAEIGASLIKHW

>sp|P0A0B2|MECR_STAEP Methicillin resistance mecR1 protein OS=Staphylococcus epidermidis OX=1282 GN=mecR1 PE=3 SV=1

MLSSFLMLSIISSLLTICVIFLVRMLYIKYTQNIMSHKIWLLVLVSTLIPLIPFYKISNFTFSKDMMNRNVSDTTSSVSHMLDGQQSSVTKDLAINVNQFETSNITYMILLIWVFGSLLCLFYMIKAFRQIDVIKSSSLESSYLNERLKVCQSKMQFYKKHITISYSSNIDNPMVFGLVKSQIVLPTVVVETMNDKEIEYIILHELSHVKSHDLIFNQLYVVFKMIFWFNPALYISKTMMDNDCEKVCDRNVLKILNRHEHIRYGESILKCSILKSQHINNVAAQYLLGFNSNIKERVKYIALYDSMPKPNRNKRIVAYIVCSISLLIQAPLLSAHVQQDKYETNVSYKKLNQLAPYFKGFDGSFVLYNEREQAYSIYNEPESKQRYSPNSTYKIYLALMAFDQNLLSLNHTEQQWDKHQYPFKEWNQDQNLNSSMKYSVNWYYENLNKHLRQDEVKSYLDLIEYGNEEISGNENYWNESSLKISAIEQVNLLKNMKQHNMHFDNKAIEKVENSMTLKQKDTYKYVGKTGTGIVNHKEANGWFVGYVETKDNTYYFATHLKGEDNANGEKAQQISERILKEMELI

>sp|A4W6F7|RSMA_ENT38 Ribosomal RNA small subunit methyltransferase A OS=Enterobacter sp. (strain 638) OX=399742 GN=rsmA PE=3 SV=1

MNTRVHQGHLARKRFGQNFLNDQFVIESIVSAINPQKGQAMVEIGPGLAALTEPVGERLDEMTVIELDRDLAARLKTHPFLGPKLTIYQQDAMTMNFAELSEKLGQPLRVFGNLPYNISTPLMFHLFSYTDAIADMHFMLQKEVVNRLVAGPNSKAYGRLSVMAQYYCQIIPVLEVPPTAFTPAPKVESAVVRLVPHAVMPHPVKELRVLSRITTEAFNQRRKTIRNSLGNTFTVDVLTELGIDPAMRAENISVEQYCKLANYISDNAPPKES

>sp|P25910|BLAB_BACFG Metallo-beta-lactamase type 2 OS=Bacteroides fragilis OX=817 GN=ccrA PE=1 SV=1

MKTVFILISMLFPVAVMAQKSVKISDDISITQLSDKVYTYVSLAEIEGWGMVPSNGMIVINNHQAALLDTPINDAQTEMLVNWVTDSLHAKVTTFIPNHWHGDCIGGLGYLQRKGVQSYANQMTIDLAKEKGLPVPEHGFTDSLTVSLDGMPLQCYYLGGGHATDNIVVWLPTENILFGGCMLKDNQATSIGNISDADVTAWPKTLDKVKAKFPSARYVVPGHGDYGGTELIEHTKQIVNQYIESTSKP

>sp|P06107|LINA_STAHA Lincosamide resistance protein OS=Staphylococcus haemolyticus OX=1283 GN=linA PE=1 SV=1

MKNNNVTEKELFYILDLFEHMKVTYWLDGGWGVDVLTGKQQREHRDIDIDFDAQHTQKVIQKLEDIGYKIEVHWMPSRMELKHEEYGYLDIHPINLNDDGSITQANPEGGNYVFQNDWFSETNYKDRKIPCISKEAQLLFHSGYDLTETDHFDIKNLKSIT

>sp|P0AE05|AADB1_KLEPN 2''-aminoglycoside nucleotidyltransferase OS=Klebsiella pneumoniae OX=573 GN=aadB PE=1 SV=1

MDTTQVTLIHKILAAADERNLPLWIGGGWAIDARLGRVTRKHDDIDLTFPGERRGELEAIVEMLGGRVMEELDYGFLAEIGDELLDCEPAWWADEAYEIAEAPQGSCPEAAEGVIAGRPVRCNSWEAIIWDYFYYADEVPPVDWPTKHIESYRLACTSLGAEKVEVLRAAFRSRYAA

>sp|A4WEI9|UPPP_ENT38 Undecaprenyl-diphosphatase OS=Enterobacter sp. (strain 638) OX=399742 GN=uppP PE=3 SV=1

MSDMHSLLVAAILGVVEGLTEFLPVSSTGHMIIVGHLLGFEGETAKTFEVVIQLGSILAVVVMFWRRLFGLIGIHFGRPPQHEGEGKGRLTLIHILLGMVPAVVLGLIFHDAIKSLFNPINVMYALVVGGVLLIAAELLKPKEPKAPGLDDMTYRQAFMIGCFQCLALWPGFSRSGATISGGMLMGVSRYAASEFSFLLAVPMMMGATALDLYKSYHFLTAADFPMFAVGFVTAFLVALVAIKTFLQLIKRISFIPFAIYRFIVAAAVYVVFF

>sp|Q87K03|QNR_VIBPA Pentapeptide repeat protein VPA0095 OS=Vibrio parahaemolyticus serotype O3:K6 (strain RIMD 2210633) OX=223926 GN=VPA0095 PE=1 SV=1

MLKTDLIFERENFSHHDFQNATFKNCHFYMCSFDHADLRDAKFIDCRFIESKALEGCSFRFANLKDASFTNCMLAMSLFNGANCMGLELRKCDLKGANFQGANFANRVSNTMFFCSAFITGCNLTYCNFERVLLEKCDLFENRWNGANLAGATLKGSDLSRCEFSPEQWGTFNVEQCDLTHVELDGLDIRRVSLFGVKICDWQQEQLLAPFGLIIL

>sp|Q7DJ53|BLE_STAAU Bleomycin resistance protein OS=Staphylococcus aureus OX=1280 GN=ble PE=1 SV=1

MLQSIPALPVGDIKKSIGFYCDKLGFTLVHHEDGFAVLMCNEVRIHLWEASDEGWRSRSNDSPVCTGAESFIAGTASCRIEVEGIDELYQHIKPLGILHPNTSLKDQWWDERDFAVIDPDNNLISFFQQIKS

>sp|P31776|PBPA_HAEIN Penicillin-binding protein 1A OS=Haemophilus influenzae (strain ATCC 51907 / DSM 11121 / KW20 / Rd) OX=71421 GN=mrcA PE=1 SV=2

MRIAKLILNTLLTLCILGLVAGGMLYFHLKSELQQPMQIYTADGKLIGEVGEQRRIPVKLADVPQRLIDAFLATEDSRFYDHHGLDPIGIARALFVAVSNGGASQGASTITQQLARNFFLTSEKTIIRKAREAVLAVEIENTLNKQEILELYLNKIFLGYRSYGVAAAAQTYFGKSLNELTLSEMAIIAGLPKAPSTMNPLYSLKRSEERRNVVLSRMLDEKYISKEEYDAALKEPIVASYHGAKFEFRADYVTEMVRQEMVRRFGEENAYTSGYKVFTTVLSKDQAEAQKAVRNNLIDYDMRHGYRGGAPLWQKNEAAWDNDRIVGFLRKLPDSEPFIPAAVIGIVKGGADILLASGEKMTLSTNAMRWTGRSNPVKVGEQIWIHQRANGEWQLGQIPAANSALVSLNSDNGAIEAVVGGFSYEQSKFNRATQSLVQVGSSIKPFIYAAALEKGLTLSSVLQDSPISIQKPGQKMWQPKNSPDRYDGPMRLRVGLGQSKNIIAIRAIQTAGIDFTAEFLQRFGFKRDQYFASEALALGAASFTPLEMARAYAVFDNGGFLIEPYIIEKIQDNTGKDLFIANPKIACIECNDIPVIYGETKDKINGFANIPLGENALKPTDDSTNGEELDQQPETVPELPELQSNMTALKEDAIDLMAAAKNASSKIEYAPRVISGELAFLIRSALNTAIYGEQGLDWKGTSWRIAQSIKRSDIGGKTGTTNSSKVAWYAGFGANLVTTTYVGFDDNKRVLGRGEAGAKTAMPAWITYMKTALSDKPERKLSLPPKIVEKNIDTLTGLLSPNGGRKEYFIAGTEPTRTYLSEMQERGYYVPTELQQRLNNEGNTPATQPQELF

>sp|P52700|BLA1_STEMA Metallo-beta-lactamase L1 type 3 OS=Stenotrophomonas maltophilia OX=40324 PE=1 SV=1

MRSTLLAFALAVALPAAHTSAAEVPLPQLRAYTVDASWLQPMAPLQIADHTWQIGTEDLTALLVQTPDGAVLLDGGMPQMASHLLDNMKARGVTPRDLRLILLSHAHADHAGPVAELKRRTGAKVAANAESAVLLARGGSDDLHFGDGITYPPANADRIVMDGEVITVGGIVFTAHFMAGHTPGSTAWTWTDTRNGKPVRIAYADSLSAPGYQLQGNPRYPHLIEDYRRSFATVRALPCDVLLTPHPGASNWDYAAGARAGAKALTCKAYADAAEQKFDGQLAKETAGAR

>sp|Q06239|VANR_ENTFC Regulatory protein VanR OS=Enterococcus faecium OX=1352 GN=vanR PE=3 SV=1

MSDKILIVDDEHEIADLVELYLKNENYTVFKYYTAKEALECIDKSEIDLAILDIMLPGTSGLTICQKIRDKHTYPIIMLTGKDTEVDKITGLTIGADDYITKPFRPLELIARVKAQLRRYKKFSGVKEQNENVIVHSGLVINVNTHECYLNEKQLSLTPTEFSILRILCENKGNVVSSELLFHEIWGDEYFSKSNNTITVHIRHLREKMNDTIDNPKYIKTVWGVGYKIEK

>sp|I6YBX3|MFPA_MYCTU Pentapeptide repeat protein MfpA OS=Mycobacterium tuberculosis (strain ATCC 25618 / H37Rv) OX=83332 GN=mfpA PE=1 SV=1

MQQWVDCEFTGRDFRDEDLSRLHTERAMFSECDFSGVNLAESQHRGSAFRNCTFERTTLWHSTFAQCSMLGSVFVACRLRPLTLDDVDFTLAVLGGNDLRGLNLTGCRLRETSLVDTDLRKCVLRGADLSGARTTGARLDDADLRGATVDPVLWRTASLVGARVDVDQAVAFAAAHGLCLAGG

>sp|P25051|VANA_ENTFC Vancomycin/teicoplanin A-type resistance protein VanA OS=Enterococcus faecium OX=1352 GN=vanA PE=1 SV=1

MNRIKVAILFGGCSEEHDVSVKSAIEIAANINKEKYEPLYIGITKSGVWKMCEKPCAEWENDNCYSAVLSPDKKMHGLLVKKNHEYEINHVDVAFSALHGKSGEDGSIQGLFELSGIPFVGCDIQSSAICMDKSLTYIVAKNAGIATPAFWVINKDDRPVAATFTYPVFVKPARSGSSFGVKKVNSADELDYAIESARQYDSKILIEQAVSGCEVGCAVLGNSAALVVGEVDQIRLQYGIFRIHQEVEPEKGSENAVITVPADLSAEERGRIQETAKKIYKALGCRGLARVDMFLQDNGRIVLNEVNTLPGFTSYSRYPRMMAAAGIALPELIDRLIVLALKG

>sp|P67919|BLA1_MANHA Beta-lactamase ROB-1 OS=Mannheimia haemolytica OX=75985 GN=rob1 PE=3 SV=1

MLNKLKIGTLLLLTLTACSPNSVHSVTSNPQPASAPVQQSATQATFQQTLANLEQQYQARIGVYVWDTETGHSLSYRADERFAYASTFKALLAGAVLQSLPEKDLNRTISYSQKDLVSYSPETQKYVGKGMTIAQLCEAAVRFSDNSATNLLLKELGGVEQYQRILRQLGDNVTHTNRLEPDLNQAKPNDIRDTSTPKQMAMNLNAYLLGNTLTESQKTILWNWLDNNATGNPLIRAATPTSWKVYDKSGAGKYGVRNDIAVVRIPNRKPIVMAIMSTQFTEEAKFNNKLVEDAAKQVFHTLQLN

>sp|Q05709|VANH_ENTFC D-specific alpha-keto acid dehydrogenase OS=Enterococcus faecium OX=1352 GN=vanH PE=1 SV=1

MNNIGITVYGCEQDEADAFHALSPRFGVMATIINANVSESNAKSAPFNQCISVGHKSEISASILLALKRAGVKYISTRSIGCNHIDTTAAKRMGITVDNVAYSPDSVADYTMMLILMAVRNVKSIVRSVEKHDFRLDSDRGKVLSDMTVGVVGTGQIGKAVIERLRGFGCKVLAYSRSRSIEVNYVPFDELLQNSDIVTLHVPLNTDTHYIISHEQIQRMKQGAFLINTGRGPLVDTYELVKALENGKLGGAALDVLEGEEEFFYSDCTQKPIDNQFLLKLQRMPNVIITPHTAYYTEQALRDTVEKTIKNCLDFERRQEHE

>sp|Q06241|VANX_ENTFC D-alanyl-D-alanine dipeptidase OS=Enterococcus faecium OX=1352 GN=vanX PE=1 SV=1

MEIGFTFLDEIVHGVRWDAKYATWDNFTGKPVDGYEVNRIVGTYELAESLLKAKELAATQGYGLLLWDGYRPKRAVNCFMQWAAQPENNLTKESYYPNIDRTEMISKGYVASKSSHSRGSAIDLTLYRLDTGELVPMGSRFDFMDERSHHAANGISCNEAQNRRRLRSIMENSGFEAYSLEWWHYVLRDEPYPNSYFDFPVK

>sp|P00382|DYR1_ECOLX Dihydrofolate reductase type 1 OS=Escherichia coli OX=562 GN=dhfrI PE=1 SV=1

MKLSLMVAISKNGVIGNGPDIPWSAKGEQLLFKAITYNQWLLVGRKTFESMGALPNRKYAVVTRSSFTSDNENVLIFPSIKDALTNLKKITDHVIVSGGGEIYKSLIDQVDTLHISTIDIEPEGDVYFPEIPSNFRPVFTQDFASNINYSYQIWQKG

>sp|P52699|BLAB_SERMA Metallo-beta-lactamase type 2 OS=Serratia marcescens OX=615 PE=1 SV=1

MSKLSVFFIFLFCSIATAAESLPDLKIEKLDEGVYVHTSFEEVNGWGVVPKHGLVVLVNAEAYLIDTPFTAKDTEKLVTWFVERGYKIKGSISSHFHSDSTGGIEWLNSRSIPTYASELTNELLKKDGKVQATNSFSGVNYWLVKNKIEVFYPGPGHTPDNVVVWLPERKILFGGCFIKPYGLGNLGDANIEAWPKSAKLLKSKYGKAKLVVPSHSEVGDASLLKLTLEQAVKGLNESKKPSKPSN

>sp|P30897|BLAC_PROMI Beta-lactamase OS=Proteus mirabilis OX=584 GN=blaP PE=1 SV=1

MNVRQHKASFFSVVITFLCLTLSLNANATDSVLEAVTNAETELGARIGLAAHDLETGKRWEHKSNERFPLSSTFKTLACANVLQRVDLGKERIDRVVRFSESNLVTYSPVTEKHVGKKGMSLAELCQATLSTSDNSAANFILQAIGGPKALTKFLRSIGDDTTRLDRWEPELNEAVPGDKRDTTTPIAMVTTLEKLLIDETLSIKSRQQLESWLKGNEVGDALFRKGVPSDWIVADRTGAGGYGSRAITAVMWPPNRKPIVAALYITETDASFEERNAVIAKIGEQIAKTVLMENSRN

>sp|P85302|AMPC_PSEFL Beta-lactamase OS=Pseudomonas fluorescens OX=294 GN=ampC PE=1 SV=1

ATDIRQVVDSTVEPLMQQQDIAGLSVAVIQNGKAQYFNYGVANKDSKQPITENTLFEIGSVSKTFTATLAGYALANGKLKLSDPASQYLPALRGDKFDHISLLNLGTYTAGGLPLQFPEESDNTGKMISYYQHWKPAFAPGTQRLYSNPSIGLFGHLAAQSLGQPFEKLMEQTVLPKLGLKHTFISVPETQMSLYAQGYDKAGKPVRVSPGALDAEAYGIKTSTSDLIHYVEVNMHPAKLEKPLQQAIAATHTGYYTVDGMTQGLGWEMYPYPIKVDALVEGNSTQMAMEPHKVNWLTPPQAAPLDTLVNKTGSTGGFGAYVAYVPSKGLGVVILANKNYPNAERVKAAHAILSAMDQ

>sp|P13082|STR_KLEPN Streptomycin 3''-kinase OS=Klebsiella pneumoniae OX=573 GN=str PE=3 SV=1

MERWRLLRDGELLTTHSSWILPVRQGDMPAMLKVARIPDEEAGYRLLTWWDGQGAARVFASAAGALLMERASGAGDLAQIAWSGQDDEACRILCDTAARLHAPRSGPPPDLHPLQEWFQPLFRLAAEHAALAPAASVARQLLAAPREVCPLHGDLHHENVLDFGDRGWLAIDPHGLLGERTFDYANIFTNPDLSDPGRPLAILPGRLEARLSIVVATTGFEPERLLRWIIAWTGLSAAWFIGDGDGEGEGAAIDLAVNAMARRLLD

>sp|P0C003|DHP1_PSEAI Dihydropteroate synthase type-1 OS=Pseudomonas aeruginosa OX=287 GN=sulI PE=3 SV=1

MVTVFGILNLTEDSFFDESRRLDPAGAVTAAIEMLRVGSDVVDVGPAASHPDARPVSPADEIRRIAPLLDALSDQMHRVSIDSFQPETQRYALKRGVGYLNDIQGFPDPALYPDIAEADCRLVVMHSAQRDGIATRTGHLRPEDALDEIVRFFEARVSALRRSGVAADRLILDPGMGFFLSPAPETSLHVLSNLQKLKSALGLPLLVSVSRKSFLGATVGLPVKDLGPASLAAELHAIGNGADYVRTHAPGDLRSAITFSETLAKFRSRDARDRGLDHA

>sp|P18150|APHE_STRGR Streptomycin 3''-kinase OS=Streptomyces griseus OX=1911 GN=aphE PE=3 SV=1

MSDHPGPGAVTPELFGVGGDWLAVTAGESGASVFRAADATRYAKCVPAADAAGLEAERDRIAWLSGQGVPGPRVLDWYAGDAGACLVTRAVPGVPADRVGADDLRTAWGAVADAVRRLHEVPVASCPFRRGLDSVVDAARDVVARGAVHPEFLPVEQRLVPPAELLARLTGELARRRDQEAADTVVCHGDLCLPNIVLHPETLEVSGFIDLGRLGAADRHADLALLLANARETWVDEERARFADAAFAERYGIAPDPERLRFYLHLDPLTWG

>sp|Q47747|VANW_ENTFA Vancomycin B-type resistance protein VanW OS=Enterococcus faecalis (strain ATCC 700802 / V583) OX=226185 GN=vanW PE=2 SV=1

MNRKRLTQRFPFLLPMRQAQRKICFYAGMRFDGCCYAQTIGEKTLPYLLFETDCALYNHNTGFDMIYQENKVFNLKLAAKTLNGLLIKPGETFSFWRLVRHADKDTPYKDGLTVANGKLTTMSGGGMCQMSNLLFWVFLHTPLTIIQRSGHVVKEFPEPNSDEIKGVDATISEGWIDLKVRNDTDCTYQIWVTLDDEKIIGQVFADKQPQALYKIANGSIQYVRESGGIYEYAKVERMQVALGTGEIIDCKLLYTNKCKICYPLPESVDIQEANQ

>sp|P26839|VATA_STAAU Virginiamycin A acetyltransferase OS=Staphylococcus aureus OX=1280 GN=vat PE=1 SV=2

MNLNNDHGPDPENILPIKGNRNLQFIKPTITNENILVGEYSYYDSKRGESFEDQVLYHYEVIGDKLIIGRFCSIGPGTTFIMNGANHRMDGSTYPFHLFRMGWEKYMPSLKDLPLKGDIEIGNDVWIGRDVTIMPGVKIGDGAIIAAEAVVTKNVAPYSIVGGNPLKFIRKRFSDGVIEEWLALQWWNLDMKIINENLPFIINGDIEMLKRKRKLLDDT

>sp|E1ANH6|BLC97_ECOLX Beta-lactamase CTX-M-97 OS=Escherichia coli OX=562 GN=bla PE=1 SV=1

MMTQSIGRSMLTVMATLPLLFSSATLHAQANSVQQQLEALEKSSGGRLGVALINTADNSQILYRADERFAMCSTSKVMAAAAVLKQSESDKHLLNQRVEIKKSDLVNYNPIAEKHVNGTMTLAELGAAALQYSDNTAMNKLIAHLGGPDKVTAFARSLGDETFRLDRTEPTLNTAIPGDPRDTTTPLAMAQTLKNLTLGKALAETQRAQLVTWLKGNTTGSASIRAGLPKSWVVGDKTGSGDYGTTNDIAVIWPENHAPLVLVTYFTQPEQKAESRRDILAAAAKIVTHGF

>sp|P26841|CAT4_PSEAE Chloramphenicol acetyltransferase OS=Pseudomonas aeruginosa (strain ATCC 15692 / DSM 22644 / CIP 104116 / JCM 14847 / LMG 12228 / 1C / PRS 101 / PAO1) OX=208964 GN=cat PE=1 SV=3

MGNYFESPFRGKLLSEQVSNPNIRVGRYSYYSGYYHGHSFDDCARYLMPDRDDVDKLVIGSFCSIGSGAAFIMAGNQGHRAEWASTFPFHFMHEEPVFAGAVNGYQPAGDTLIGHDVWIGTEAMFMPGVRVGHGAIIGSRALVTGDVEPYAIVGGNPARTIRKRFSDGDIQNLLEMAWWDWPLADIEAAMPLLCTGDIPALYRHWKQRQATA

>sp|Q06242|VANZ_ENTFC Protein VanZ OS=Enterococcus faecium OX=1352 GN=vanZ PE=4 SV=2

MGKILSRGLLALYLVTLIWLVLFKLQYNILSVFNYHQRSLNLTPFTATGNFREMIDNVIIFIPFGLLLNVNFKEIGFLPKFAFVLVLSLTFEIIQFIFAIGATDITDVITNTVGGFLGLKLYGLSNKHMNQKKLDRVIIFVGILLLVLLLVYRTHLRINYV

>sp|P13018|STA_ECOLX Streptothricin acetyltransferase OS=Escherichia coli OX=562 GN=sat-1 PE=3 SV=1

MKISVIPEQVAETLDAENHFIVREVFDVHLSDQGFELSTRSVSPYRKDYISDDDSDEDSACYGAFIDQELVGKIELNSTWNDLASIEHIVVSHTHRGKGVAHSLIEFAKKWALSRQLLGIRLETQTNNVPACNLYAKCGFTLGGIDLFTYKTRPQVSNETAMYWYWFSGAQDDA

>sp|P17585|AADK_BACSU Aminoglycoside 6-adenylyltransferase OS=Bacillus subtilis (strain 168) OX=224308 GN=aadK PE=1 SV=1

MRSEQEMMDIFLDFALNDERIRLVTLEGSRTNRNIPPDNFQDYDISYFVTDVESFKENDQWLEIFGKRIMMQKPEDMELFPPELGNWFSYIILFEDGNKLDLTLIPIREAEDYFANNDGLVKVLLDKDSFINYKVTPNDRQYWIKRPTAREFDDCCNEFWMVSTYVVKGLARNEILFAIDHLNEIVRPNLLRMMAWHIASQKGYSFSMGKNYKFMKRYLSNKEWEELMSTYSVNGYQEMWKSLFTCYALFRKYSKAVSEGLAYKYPDYDEGITKYTEGIYCSVK

>sp|P50858|AAC6_KLEAE Aminoglycoside N(6')-acetyltransferase type 1 OS=Klebsiella aerogenes OX=548 GN=aacA7 PE=3 SV=1

MDSSPLVRPVETTDSASWLSMRCELWPDGTCQEHQSEIAEFLSGKVARPAAVLIAVAPDGEALGFAELSIRPYAEECYSGNVAFLEGWYVVPSARRQGVGVALVKAAEHWARGRGCTEFASDTQLTNSASTSAHLAAGFTEVAQVRCFRKPL

>sp|Q2PT27|QNRB4_ECOLX Pentapeptide repeat protein QnrB4 OS=Escherichia coli OX=562 GN=qnrB4 PE=3 SV=2

MMTLALVGEKIDRNRFTGEKVENSTFFNCDFSGADLSGTEFIGCQFYDRESQKGCNFSRANLKDAIFKSCDLSMADFRNINALGIEIRHCRAQGSDFRGASFMNMITTRTWFCSAYITNTNLSYANFSKVVLEKCELWENRWMGTQVLGATFSGSDLSGGEFSSFDWRAANVTHCDLTNSELGDLDIRGVDLQGVKLDSYQASLLLERLGIAVMG

>sp|A8A2C2|ARNA_ECOHS Bifunctional polymyxin resistance protein ArnA OS=Escherichia coli O9:H4 (strain HS) OX=331112 GN=arnA PE=3 SV=1

MKTVVFAYHDMGCLGIEALLAAGYEISAIFTHTDNPGEKAFYGSVAHLAAERGIPVYAPDNVNHPLWVERIAQLSPEVIFSFYYRHLICDEILQLAPAGAFNLHGSLLPKYRGRAPLNWVLVNGETETGVTLHRMVKRADAGAIVAQLRVAIAPDDIAITLHHKLCHAARQLLEQTLPAIKHGNILEIAQRENEATCFGRRTPDDSFLEWHKPASVLHNMVRAVADPWPGAFSYVGNQKFTVWSSRVHPHASKAQPGSVISIAPLLIACGDGALEIVTGQAGDGITMQGSQLAQTLGLVQGSRLNSQPACTARRRTRVLILGVNGFIGNHLTERLLREDHYEVYGLDIGSDAISRFLNHPHFHFVEGDISIHSEWIEYHVKKCDVVLPLVAIATPIEYTRNPLRVFELDFEENLRIIRYCVKYRKRIIFPSTSEVYGMCSDKYFDEDHSNLIVGPVNKPRWIYSVSKQLLDRVIWAYGEKEGLQFTLFRPFNWMGPRLDNLNAARIGSSRAITQLILNLVEGSPIKLIDGGKQKRCFTDIRDGIEALYRIIENAGNRCDGEIINIGNPENEASIEELGEMLLASFEKHPLRHHFPPFAGFRVVESSSYYGKGYQDVEHRKPSIRNAHRCLDWEPKIDMQETIDETLDFFLRTVDLTDKPS

>sp|P59676|PBPX_STRR6 Penicillin-binding protein 2X OS=Streptococcus pneumoniae (strain ATCC BAA-255 / R6) OX=171101 GN=pbpX PE=1 SV=1

MKWTKRVIRYATKNRKSPAENRRRVGKSLSLLSVFVFAIFLVNFAVIIGTGTRFGTDLAKEAKKVHQTTRTVPAKRGTIYDRNGVPIAEDATSYNVYAVIDENYKSATGKILYVEKTQFNKVAEVFHKYLDMEESYVREQLSQPNLKQVSFGAKGNGITYANMMSIKKELEAAEVKGIDFTTSPNRSYPNGQFASSFIGLAQLHENEDGSKSLLGTSGMESSLNSILAGTDGIITYEKDRLGNIVPGTEQVSQRTMDGKDVYTTISSPLQSFMETQMDAFQEKVKGKYMTATLVSAKTGEILATTQRPTFDADTKEGITEDFVWRDILYQSNYEPGSTMKVMMLAAAIDNNTFPGGEVFNSSELKIADATIRDWDVNEGLTGGRMMTFSQGFAHSSNVGMTLLEQKMGDATWLDYLNRFKFGVPTRFGLTDEYAGQLPADNIVNIAQSSFGQGISVTQTQMIRAFTAIANDGVMLEPKFISAIYDPNDQTARKSQKEIVGNPVSKDAASLTRTNMVLVGTDPVYGTMYNHSTGKPTVTVPGQNVALKSGTAQIADEKNGGYLVGLTDYIFSAVSMSPAENPDFILYVTVQQPEHYSGIQLGEFANPILERASAMKDSLNLQTTAKALEQVSQQSPYPMPSVKDISPGDLAEELRRNLVQPIVVGTGTKIKNSSAEEGKNLAPNQQVLILSDKAEEVPDMYGWTKETAETLAKWLNIELEFQGSGSTVQKQDVRANTAIKDIKKITLTLGD

>sp|C7C422|BLAN1_KLEPN Metallo-beta-lactamase type 2 OS=Klebsiella pneumoniae OX=573 GN=blaNDM-1 PE=1 SV=1

MELPNIMHPVAKLSTALAAALMLSGCMPGEIRPTIGQQMETGDQRFGDLVFRQLAPNVWQHTSYLDMPGFGAVASNGLIVRDGGRVLVVDTAWTDDQTAQILNWIKQEINLPVALAVVTHAHQDKMGGMDALHAAGIATYANALSNQLAPQEGMVAAQHSLTFAANGWVEPATAPNFGPLKVFYPGPGHTSDNITVGIDGTDIAFGGCLIKDSKAKSLGNLGDADTEHYAASARAFGAAFPKASMIVMSHSAPDSRAAITHTARMADKLR

>sp|Q06893|VANB_ENTFA Vancomycin B-type resistance protein VanB OS=Enterococcus faecalis (strain ATCC 700802 / V583) OX=226185 GN=vanB PE=1 SV=2

MNKIKVAIIFGGCSEEHDVSVKSAIEIAANINTEKFDPHYIGITKNGVWKLCKKPCTEWEADSLPAIFSPDRKTHGLLVMKEREYETRRIDVAFPVLHGKCGEDGAIQGLFELSGIPYVGCDIQSSAACMDKSLAYILTKNAGIAVPEFQMIEKGDKPEARTLTYPVFVKPARSGSSFGVTKVNSTEELNAAIEAAGQYDGKILIEQAISGCEVGCAVMGNEDDLIVGEVDQIRLSHGIFRIHQENEPEKGSENAMIIVPADIPVEERNRVQETAKKVYRVLGCRGLARVDLFLQEDGGIVLNEVNTLPGFTSYSRYPRMAAAAGITLPALIDSLITLAIER

>sp|P00555|KKA5_STRFR Aminoglycoside 3'-phosphotransferase OS=Streptomyces fradiae OX=1906 GN=aph PE=3 SV=1

MDDSTLRRKYPHHEWHAVNEGDSGAFVYQLTGGPEPQPELYAKIAPRAPENSAFDLSGEADRLEWLHRHGIPVPRVVERGADDTAAWLVTEAVPGVAAAEEWPEHQRFAVVEAMAELARALHELPVEDCPSDRRLDAAVAEARRNVAEGLVDLDDLQEERAGWTGDQLLAELDRTRPEKEDLVVCHGDLCPNNVLLDPGTCRVTGVIDVGRLGVADRHADIALAARELEIDEDPWFGPAYAERFLERYGAHRVDKEKLAFYQLLDEFF

>sp|P09885|KKA6_ACIBA Aminoglycoside 3'-phosphotransferase OS=Acinetobacter baumannii OX=470 GN=aphA-6 PE=3 SV=1

MELPNIIQQFIGNSVLEPNKIGQSPSDVYSFNRNNETFFLKRSSTLYTETTYSVSREAKMLSWLSEKLKVPELIMTFQDEQFEFMITKAINAKPISALFLTDQELLAIYKEALNLLNSIAIIDCPFISNIDHRLKESKFFIDNQLLDDIDQDDFDTELWGDHKTYLSLWNELTETRVEERLVFSHGDITDSNIFIDKFNEIYFLDLGRAGLADEFVDISFVERCLREDASEETAKIFLKHLKNDRPDKRNYFLKLDELN

>sp|P0A2Q8|MERR_SALTI Mercuric resistance operon regulatory protein OS=Salmonella typhi OX=90370 GN=merR PE=3 SV=1

MENNLENLTIGVFAKAAGVNVETIRFYQRKGLLREPDKPYGSIRRYGEADVVRVKFVKSAQRLGFSLDEIAELLRLDDGTHCEEASSLAEHKLKDVREKMADLARMETVLSELVCACHARKGNVSCPLIASLQGEAGLARSAMP

>sp|P06689|MERD_PSEAI HTH-type transcriptional regulator MerD OS=Pseudomonas aeruginosa OX=287 GN=merD PE=4 SV=1

MNAYPVSRLALDAGVSVHIVRDYLLRGLLRPVACTPGGYGLFDDAALQRLCFVRAAFEAGIGLDALARLCRALDAADGDEAAAQLALLRQFVERRREALADLEVQLATLPTEPAQHAESLP

>sp|O84955|BLO20_PSEAI Beta-lactamase OXA-20 OS=Pseudomonas aeruginosa OX=287 GN=bla PE=3 SV=1

MIIRFLALLFSAVVLVSLGHAQEKTHESSNWGKYFSDFNAKGTIVVVDERTNGNSTSVYNESRAQQRYSPASTFKIPHTLFALDAGAVRDEFHVFRWDGAKRSFAGHNQDQNLRSAMRNSTVWVYQLFAKEIGENKARSYLEKLNYGNADPSTKSGDYWIDGNLAISANEQISILKKLYRNELPFRVEHQRLVKDLMIVEAKRDWILRAKTGWDGQMGWWVGWVEWPTGPVFFALNIDTPNRMEDLHKREAIARAILQSVNALPPN

>sp|P12921|TMRB_BACSU Tunicamycin resistance protein OS=Bacillus subtilis (strain 168) OX=224308 GN=tmrB PE=1 SV=4

MIIWINGAFGSGKTQTAFELHRRLNPSYVYDPEKMGFALRSMVPQEIAKDDFQSYPLWRAFNYSLLASLTDTYRGILIVPMTIVHPEYFNEIIGRLRQEGRIVHHFTLMASKETLLKRLRTRAEGKNSWAAKQIDRCVEGLSSPIFEDHIQTDNLSIQDVAENIAARAELPLDPDTRGSLRRFADRLMVKLNHIRIK

>sp|Q45726|BLAC_BACTU Beta-lactamase OS=Bacillus thuringiensis OX=1428 GN=bla PE=3 SV=1

MMILKNKRMLKIGICVGILGLSITSLEAFTGGALQVEAKQKTGQVKHKNQATHKEFSQLEKKFDARVGVYAIDTGTNQTISYRSNERFAFASTYKALAAGVLLQQNSIDTLNEVITFTKEDLVDYSPVTEKHVDTGMKLGEIAEAAVRSSDNTAGNILFNKIGGPKGYEKALRKMGDRVTMSDRFETELNEAIPGDIRDTSTAKRIATNLKAFTVGNALPAEKRKILTEWMKGNATGDKLIRAGVPTDWVVGDKSGAGSYGTRNDIAIVWPPNRAPIIIAILSSKDEKEASYDNQLIAEATEVIVKALK

>sp|P12055|STR_STAAU Streptomycin resistance protein OS=Staphylococcus aureus OX=1280 GN=str PE=4 SV=1

MRTEKEILNLVSEFAYQRSNVKIIALEGSRTNENIKKDKFQDYDFAFFVSDIEYFTHEESWLSLFGELLFIQKPEDMELFPPDLDYGYSYIMYFKDGIKMDITLINLKDLNRYFSDSDGLVKILVDKDNLVTQEIVPDDSNYWLKKPTEREFYDCCNEFWSVSTYVAKGVFRREILFALDHFNNILRPELLRMISWYIGFNRGFDFSLGKNYKFINKYLTDKEFNMLLATFEMNGYRKTYQSFKLCCELFKYYSNKVSCLGNYNYPNYEKNIENFIRNNYEN

>sp|P05193|AMPC_CITFR Beta-lactamase OS=Citrobacter freundii OX=546 GN=ampC PE=1 SV=1

MMKKSICCALLLTASFSTFAAAKTEQQIADIVNRTITPLMQEQAIPGMAVAIIYEGKPYYFTWGKADIANNHPVTQQTLFELGSVSKTFNGVLGGDRIARGEIKLSDPVTKYWPELTGKQWRGISLLHLATYTAGGLPLQIPGDVTDKAELLRFYQNWQPQWTPGAKRLYANSSIGLFGALAVKSSGMSYEEAMTRRVLQPLKLAHTWITVPQSEQKNYAWGYLEGKPVHVSPGQLDAEAYGVKSSVIDMARWVQANMDASHVQEKTLQQGIELAQSRYWRIGDMYQGLGWEMLNWPLKADSIINGSDSKVALAALPAVEVNPPAPAVKASWVHKTGSTGGFGSYVAFVPEKNLGIVMLANKSYPNPARVEAAWRILEKLQ

>sp|Q9X3P3|VANT_ENTGA Serine/alanine racemase OS=Enterococcus gallinarum OX=1353 GN=vanT PE=1 SV=1

MKNKGIDQFRVIAAMMVVAIHCLPLHYLWPEGDILITLTIFRVAVPFFFMISGYYVFAELAVANSYPSRQRVFNFIKKQLKVYLLATLMFLPLALYSQTIGFDLPVGTLVQVLLVNGILYHLWYFPALITGSLLLTSLLIHVSFKKVFWLAAGLYLIGLGGDSWFGLIQQTPIEPFYTAVFHLLDGTRNGIFFTPLFLCLGVLVRKQSEKRSLSKTALFFLISLIGLLIESAYLHGFSIPKHDSMYLFLPVVLFFLFPLILRWHPHRTWKHPGQLSLWLYLLHPYTIAGTHFLSQKISILQNNLINYLVVLILTIGFICLFLRQKHSWFRHKQTTPVKRAVKEFSKTALLHNLQEIQRIISPKTKVMAVVKADAYGCGAKEVAPVLEQAGIDFFAVATIDEGIRLRKNAVKSPILVLGYTSPKRIKELRRYSLTQSIISEGHAVALSQRKVAIDCHLAIDTGMHRLGVTPTIDSILSIFDLPFLTISGVYSHLGSADRLNPDSMIRTQKQIACFDQILLELDQRQISYGITHLQSSYGILNYPDLNYDYVRPGILLTGSLSDTNEPTKQRVSLQPILTLKAQLITKRVVAKGEAIGYGQTAVANQETTVGVVSIGYCDGLPRSLSNQEFCLSYRGQSLPQIGLICMDMLLIDLSHCPTIPIESEIEILTDWSDTAEQVQTITNELICRIGPRVSARIK

>sp|P45439|ERMS_STRFR rRNA adenine N-6-methyltransferase OS=Streptomyces fradiae OX=1906 GN=ermSF PE=3 SV=1

MARAPRSPHPARSRETSRAHPPYGTRADRAPGRGRDRDRSPDSPGNTSSRDGGRSPDRARRELSQNFLARRAVAERVARLVRPAPGGLLLEVGAGRGVLTEALAPYCGRLVAHEIDPRLLPALRDRFGGPHHAHVRISGGDFLAAPVPREPFALAGNIPYSRTAGIVDWALRARTLTSATFVTQLEYARKRTGDYGRWSLLTVRTWPRHEWRLLGRVSRREFRPVPRVDSGILRIERRERPLLPSAALGDYHRMVELGFSGVGGSLYASLRRAHRAGPLDAAFRAARLDRSVVVAYVTPEQWLTVFRTLRPVRSRPAGR

>sp|P07944|PBP_STAAU Beta-lactam-inducible penicillin-binding protein OS=Staphylococcus aureus OX=1280 GN=pbp PE=2 SV=1

MKKIKIVPLILIVVVVGFGIYFYASKDKEINNTIDAIEDKNFKQVYKDSSYISKSDNGEVEMTERPIKIYNSLGVKDINIQDRKIKKVSKNKKRVDAQYKIKTNYGNIDRNVQFNFVKEDGMWKLDWDHSVIIPGMQKDQSIHIENLKSERGKILDRNNVELANTGTHMRLGIVPKNVSKKDYKAIAKELSISEDYINNKWIKIGYKMIPSFHFKTVKKMDEYLSDFAKKFHLTTNETESRNYPLGKATSHLLGYVGPINSEELKQKEYKGYKDDAVIGKKGLEKLYDKKLQHEDGYRVTIVRVDDNSNTIAHTLIEKKKKDGKDIQLTIDAKVQKSIYNNMKNDYGSGTAIHPQTGELLALVSTPSYDVYPFMYGMSNEEYNKLTEDKKEPLLNKFQITTSPGSTQKILTAMIGLNNKTLDDKTSYKIDGKGWQKDKSWGGYNVTRYEVVNGNIDLKQAIESSDNIFFARVALELGSKKFEKGMKKLGVGEDIPSDYPFYNAQISNKNLDNEILLADSGYGQGEILINPVQILSIYSALENNGNINAPHLLKDTKNKVWKKNIISKENINLLNDGMQQVVNKTHKEDIYRSYANLIGKSGTAELKMKQGETGRQIGWFISYDKDNPNMMMAINVKDVQDKGMASYNAKISGKVYDELYENGNKKYDIDE

>sp|P07287|ERME_SACEN rRNA adenine N-6-methyltransferase OS=Saccharopolyspora erythraea (strain ATCC 11635 / DSM 40517 / JCM 4748 / NBRC 13426 / NCIMB 8594 / NRRL 2338) OX=405948 GN=ermE PE=3 SV=2

MSSSDEQPRPRRRNQDRQHPNQNRPVLGRTERDRNRRQFGQNFLRDRKTIARIAETAELRPDLPVLEAGPGEGLLTRELADRARQVTSYEIDPRLAKSLREKLSGHPNIEVVNADFLTAEPPPEPFAFVGAIPYGITSAIVDWCLEAPTIETATMVTQLEFARKRTGDYGRWSRLTVMTWPLFEWEFVEKVDRRLFKPVPKVDSAIMRLRRRAEPLLEGAALERYESMVELCFTGVGGNIQASLLRKYPRRRVEAALDHAGVGGGAVVAYVRPEQWLRLFERLDQKNEPRGGQPQRGRRTGGRDHGDRRTGGQDRGDRRTGGRDHRDRQASGHGDRRSSGRNRDDGRTGEREQGDQGGRRGPSGGGRTGGRPGRRGGPGQR

>sp|Q9KJA7|BLAB8_ELIME Metallo-beta-lactamase type 2 OS=Elizabethkingia meningoseptica OX=238 GN=blaB8 PE=3 SV=1

MKGLKGLLVLALGFTGLQVFGQQNPDIKIEKLKDNLYVYTTYNTFKGTKYAANAVYMVTDKGVVVIDSPWGEDKFKSFTDEIYKKHGKKVIMNIATHSHDDRAGGLEYFGKLGAKTYSTKMTDSILAKENKPRAKYTFDNNKSFKVGKTEFQVYYPGKGHTADNVVVWFPKDKVLVGGCIVKSGDSKDLGFIGEAYVNDWTQSIHNIQQKFPDVQYVVAGHDDWKDQTSIQHTLDLISEYQQKQKASN

>sp|P58777|CAT_KLESP Chloramphenicol acetyltransferase OS=Klebsiella sp. OX=576 GN=cat PE=1 SV=1

MEKKITGYTTVDISQWHRKEHFEAFQSVAQCTYNQTVQLDITAFLKTVKKNKHKFYPAFIHILARLMNAHPEFRMAMKDGELVIWDSVHPCYTVFHEQTETFSSLWSEYHDDFRQFLHIYSQDVACYGENLAYFPKGFIENMFFVSANPWVSFTSFDLNVAAMDNFFAPVFTMGKYYTQGDKVLMPLAIQVHHAVCDGFHVGRMLNELQQYCDEWQGGA

>sp|O69773|AMPC_PROST Beta-lactamase OS=Providencia stuartii OX=588 GN=ampC PE=3 SV=1

MDNSMKNIFRQGRLFIALSLAMTSISAFALTQQEVDDIIKPLMKQEQIPGMSVAISVNGKQAIYHYGVQSKQTQIPVSDRTLYEIGSLSKTFTATLATYAQIQGKLDFSQSVSHYLPELKGSAFDNVSVMNLATHTSGLSLFVPSDIKTNDQLMAYYQKWLPDNEVGQYRSYSNLGVGLLGIVTAKQLNMPFSQAMEKLMLPSLGLKHTYIHVPKSQEKYYAQGYNKQNQPVRLNLEILGPEAYGLKSNAKDLIRYLEINMQSIKVAKTWQEAIENTHTGVYLTDSFVQDMMWESYPWPVSLSQLLQGNRDDMALKPQKVELIKPAMAPEVRAYYNKTGSSNGFATYAIFIPEEKIAIVMLSNKWIPIPQRITATYQLLEKIER

>sp|P14559|BLAC_STRAL Beta-lactamase OS=Streptomyces albus G OX=1962 PE=1 SV=1

MHPSTSRPSRRTLLTATAGAALAAATLVPGTAHASSGGRGHGSGSVSDAERRLAGLERASGARLGVYAYDTGSGRTVAYRADELFPMCSVFKTLSSAAVLRDLDRNGEFLSRRILYTQDDVEQADGAGPETGKPQNLANAQLTVEELCEVSITASDNCAANLMLRELGGPAAVTRFVRSLGDRVTRLDRWEPELNSAEPGRVTDTTSPRAITRTYGRLVLGDALNPRDRRLLTSWLLANTTSGDRFRAGLPDDWTLGDKTGAGRYGTNNDAGVTWPPGRAPIVLTVLTAKTEQDAARDDGLVADAARVLAETLG

>sp|Q44056|BLA1_AERHY Beta-lactamase AER-1 OS=Aeromonas hydrophila OX=644 GN=aer1 PE=3 SV=2

MYVLSVEKPTLRNKFAAGIGVVLVCVVASFIPTPVFALDTTKLIQAVQSEESALHARVGMTVFDSNTGTTWNYRGDERFPLNSTHKTFSCAALLAKVDGKSLSLGQSVSISKEMLVTYSPITEKSLSPETVTFGKICQAAVSYSDNTAANVVFDAIGGATGFNAYMRSIGDEETQLDRKEPELNEGTPGDVRDTTTPNAMVNSLRKILLGDALSASSRSQLTQWMLDDQVAGALLRASLPSDWKIADKTGAGGYGSRSIVAVIWPPSKQPLVVGIYITQTKASMQASNQAIARIGVVLKDTVAP

>sp|P26840|MATA_LYSSH Probable macrolide acetyltransferase (Fragment) OS=Lysinibacillus sphaericus OX=1421 PE=3 SV=1

DHKNSPEKFYDNIEHHYEFIGDKLIIGKFCAIAEGVKFIMNGANHRMDGITTYPFNIFGCGWEKVTPTIEQLPFKGDTVIGNDVWIGQNVTIMPGVIIGDGAIIAANSTVVKSVEPYSIYSGNPAKFIKKRFSDEKIEFLLKLEWWNWSGEEIFDNLEILTSEAGLEELMNKYSKRDAIN

>sp|P23364|CAT4_AGRFC Chloramphenicol acetyltransferase OS=Agrobacterium fabrum (strain C58 / ATCC 33970) OX=176299 GN=cat PE=3 SV=1

MENYFESPFRGITLDKQVKSPNLVVGKYSYYSGYYHGHSFEDCARYLLPDEGADRLVIGSFCSIGSGAAFIMAGNQGHRNEWISTFPFFFMPEVPEFENAANGYLPAGDTVIGNDVWIGSEAIIMPGITVGDGAVIGTRALVTKDVEPYAIVGGNPAKTIRKRFDDDSIALLLEMKWWGWPAERLKAAMPLMTSGNVAALYRFWRSDSL

>sp|Q52424|AAC2_PROST Aminoglycoside 2'-N-acetyltransferase OS=Providencia stuartii OX=588 GN=aac PE=1 SV=1

MGIEYRSLHTSQLTLSEKEALYDLLIEGFEGDFSHDDFAHTLGGMHVMAFDQQKLVGHVAIIQRHMALDNTPISVGYVEAMVVEQSYRRQGIGRQLMLQTNKIIASCYQLGLLSASDDGQKLYHSVGWQIWKGKLFELKQGSYIRSIEEEGGVMGWKADGEVDFTASLYCDFRGGDQW

>sp|Q55002|OTRA_STRRM Oxytetracycline resistance protein OS=Streptomyces rimosus OX=1927 GN=otrA PE=3 SV=1

MNKLNLGILAHVDAGKTSLTERLLHRTGVIDEVGSVDAGTTTTDSMELERQRGITIRSAVATFVLDDLKVNLIDTPGHSDFISEVERALGVLDGAVLVVSAVEGVQPQTRILMRTLRRLGIPTLVFVNKIDRGGARPDGVLREIRDRLTPAAVALSAVADAGTPRARAIALGPDTDPDFAVRVGELLADHDDAFLTAYLDEEHVLTEKEYAEELAAQTARGLVHPVYFGSALTGEGLDHLVHGIRELLPSVHASQDAPLRATVFKVDRGARGEAVAYLRLVSGTLGTRDSVTLHRVDHTGRVTEHAGRITALRVFEHGSATSETRATAGDIAQAWGLKDVRVGDRAGHLDGPPPRNFFAPPSLETVIRPERPEEAGRLHAALRMLDEQDPSIDLRQDEENAAGAVVRLYGEVQKEILGSTLAESFGVRVRFDPTRTVCIEKPVGTGEALIELDTRTHNYFWGAPWVCASDRPSPARAITFRLAVELGSLPLAFHKAIEETVHTTLRHGLYGWQVTDCAVTLTRTGVRSPVSAADDFRKANARLVLMDALGRAGTEVHEPVSSFELEVPAARLSPVLAKLAELGATPGVPTAEGDVFRLEGTMPTSLVHDFNQRVPGLTQGEGVFLAEHRGYRPAVGQPPVRPRPEGPNPLNRDEYILHVLKRV

>sp|P00552|KKA2_KLEPN Aminoglycoside 3'-phosphotransferase OS=Klebsiella pneumoniae OX=573 GN=neo PE=1 SV=1

MIEQDGLHAGSPAAWVERLFGYDWAQQTIGCSDAAVFRLSAQGRPVLFVKTDLSGALNELQDEAARLSWLATTGVPCAAVLDVVTEAGRDWLLLGEVPGQDLLSSHLAPAEKVSIMADAMRRLHTLDPATCPFDHQAKHRIERARTRMEAGLVDQDDLDEEHQGLAPAELFARLKARMPDGEDLVVTHGDACLPNIMVENGRFSGFIDCGRLGVADRYQDIALATRDIAEELGGEWADRFLVLYGIAAPDSQRIAFYRLLDEFF

>sp|P22782|CAT_CAMCO Chloramphenicol acetyltransferase OS=Campylobacter coli OX=195 PE=3 SV=1

MQFTKIDINNWTRKEYFDHYFGNTPCTYSMTVKLDISKLKKDGKKLYPTLLYGVTTIINRHEEFRTALDENGQVGVFSEMLPCYTVFHKETETFSSIWTEFTADYTEFLQNYQKDIDAFGERMGMSAKPNPPENTFPVSMIPWTSFEGFNLNLKKGYDYLLPIFTFGKYYEEGGKYYIPLSIQVHHAVCDGFHVCRFLDELQDLLNK

>sp|Q56415|FOSA_SERMA Glutathione transferase FosA OS=Serratia marcescens OX=615 GN=fosA PE=1 SV=1

MLQSLNHLTLAVSDLQKSVTFWHELLGLTLHARWNTGAYLTCGDLWVCLSYDEARQYVPPQESDYTHYAFTVAEEDFEPLSQRLEQAGVTIWKQNKSEGASFYFLDPDGHKLELHVGSLAARLAACREKPYAGMVFTSDEA

>sp|P26918|BLAB_AERHY Metallo-beta-lactamase type 2 OS=Aeromonas hydrophila OX=644 GN=cphA PE=1 SV=1

MMKGWMKCGLAGAVVLMASFWGGSVRAAGMSLTQVSGPVYVVEDNYYVQENSMVYFGAKGVTVVGATWTPDTARELHKLIKRVSRKPVLEVINTNYHTDRAGGNAYWKSIGAKVVSTRQTRDLMKSDWAEIVAFTRKGLPEYPDLPLVLPNVVHDGDFTLQEGKVRAFYAGPAHTPDGIFVYFPDEQVLYGNCILKEKLGNLSFADVKAYPQTLERLKAMKLPIKTVIGGHDSPLHGPELIDHYEALIKAAPQS

>sp|P94958|AMPC_MORMO Beta-lactamase OS=Morganella morganii OX=582 GN=ampC PE=3 SV=1

MKKSLSATLISALLAFSAPGFSAADNVAAVVDSTIKPLMAQQDIPGMAVAVSVKGKPYYFNYGFADVQAKQPVTENTLFELGSVSKTFTGVLGAVSVAKKEMTLNDPAEKYQPELALPQWKGITLLDLATYTAGGLPLQVPDAVKSRADLLHFYQQWQPSRKPGDMRLYANSSIGLFGALTANAAGMPYEQLLTARILAPLGLSHTFITVPESAQSQYAYGYKNKKPVRVSPGQLDAESYGVKSASKDMLRWAEMNMEPSRAGNADLEMAMYLAQTRYYKTAAINQGLGWEMYDWPQQKDMIINGVTNEVALQPHPVTDNQVQPYNRASWVHKTGATTGFGAYVAFIPEKQVAIVILANKNYPNTERVKAAQAILSALE

>sp|Q93A44|VANTE_ENTFL Amino-acid racemase OS=Enterococcus faecalis OX=1351 GN=vanTE PE=3 SV=1

MKHRANGIDLFRIFAATMVVAIHTFPFQSIAPFLDEVITLTVFRVAVPFFFMITGYFLLGRLSLNFSYNNNQRVKKYLYKIGMIYLYSILLYFPLSLLNGTISLKMNILLLLKVFIFDGTFYHLWYFPASIIGTILVTLLLRSIGFKLTVAFSTCLYLVGLGGDSWYGITNQVPLLNKLYTFIFSWSDYTRSGVFFTPVFLCLGIFAYRVSKKLTASKILNLLFYVFIIGMTFESIFLHRFTNVKHDSMYLLLPSCALILFLMLLNWQPKLKVKESADLTLLVYILHPLVIVIVHSISKYIPILKNSLLNFLLVVVCSFILAQLLLNLKRKLRVSKQKIPFERASKEISASAIHHNINEIRKIIPKNTNIMGVVKANAYGCGMVEVAYELEKIGISFFCVATIEEAIALRKSGNQGDILILGYTHPNRINDIKKYNLIQSIVSEEHGKVLNLKKIPIRCHLQVDTGMHRLGVTPNVTIIQQMYLFSNLKIEGIYSHLGSSDSLEQESIARTNTQIFLFNNILSDLEQMGISYGYTHIQSSYGILNYPELSFDFVRIGILCYGFLSDYNSPTKIPIDLQPIVKVKASLITERIVEAGEYVGYGLGAKVEKRTRIGVVSIGYADGIPRALSNAKLTLEFKGQSIKQIGNICMDMMLVDLSEVEDISLNDELIVLPNISKIADEEQTITNELLSRLGSRLGTELN

>sp|P45460|AMPC_YEREN Beta-lactamase OS=Yersinia enterocolitica OX=630 GN=ampC PE=3 SV=1

MMKKSIINTLIFTSIATFPLYTLAQTKLTELQVATIVNNTLTPLLEKQGIPGMAVAVFYDGKPQFFNYGMADIKAGRPVTENTLFELGSVSKTFTGVAGEYAMQTGIMNLNDPVTEYAPELTGSQWKDVKMLHLATYTAGGLPLQLPDSVTDQKSLWQYYQQWQPQWAPGVMRNYSNASIGLFGALAVKRSQLTFENYMKEYVFQPLKLDHTFITIPESMQSNYAWGYKDGQPVRVTLGMLGEEAYGVKSTSQDMVRFMQANMDPESLPAGNDKLKEAIIASQSRYFQAGDMFQGLGWEMYSWPINPQGVIADSGNDIALKPRKVEALVPAQPAVRASWVHKTGATNGFGAYIVFIPEEKVGIVMLANKNYPNPVRVQAAYDILQALR

>sp|P16898|ERMA_CORDP rRNA adenine N-6-methyltransferase OS=Corynebacterium diphtheriae OX=1717 GN=ermA PE=3 SV=1

MSAYGHGRHEHGQNFLTNHKIINSIIDLVKQTSGPIIEIGPGSGALTHPMAHLGRAITAVEVDAKLAAKITQETSSAAVEVVHDDFLNFRLPATPCVIVGNIPFHLTTAILRKLLHAPAWTDAVLLMQWEVARRRAGVGASTMMTAQWSPWFTFHLGSRVPRSAFRPQPNVDGGILVIRRVGDPKIPIEQRKAFQAMVHTVFTARGRGIGEILRRQGCFHHVQKHNHGCAREESTPRPYLPDCTPTTGSISSR

>sp|Q03680|BLA1_STRCI Beta-lactamase 1 OS=Streptomyces cacaoi OX=1898 GN=blaL PE=1 SV=1

MRIRPTRRLLLGAVAPLALVPLVACGQASGSESGQQPGLGGCGTSAHGSADAHEKEFRALEKKFDAHPGVYAIDTRDGQEITHRADERFAYGSTFKALQAGAILAQVLRDGREVRRGAEADGMDKVVHYGQDAILPNSPVTEKHVADGMSLRELCDAVVAYSDNTAANLLFDQLGGRRGSTRVLKQLGDHTTSMDRYEQELGSAVPGDPRDTSTPRAFAEDLRAFAVEDGEKAALAPNDREQLNDWMSGSRTGDALIRAGVPKDWKVEDKSGQVKYGTRNDIAVVRPPGRAPIVVSVMSHGDTQDAEPHDELVAEAGLVVADGLK

>sp|Q59514|BLA1_MORCA Beta-lactamase BRO-1 OS=Moraxella catarrhalis OX=480 GN=bla PE=3 SV=1

MQRRHFLQKTLLALPIIFSGNLLTGCKTNLSDDYLPDDKITNNPNLLQNKLKEILPIWENKFNAKIGMTIIADNGELSSHRGNEYFPVNSTIKAFIASHILLLVDKEKLDLNEKIIIKESDLIEYSPVCKKYFDENKPISISELCEATITLSDNGSANILLDKIGGLTAFNQFLKEIGADMVLANNEPLLNRSHYGETSDTAKPIPYTKSLKALIVGNILSNQSKEQLITWLINDKVADNLLRKYLPKNWRIGDKTGTGSESKNIIAVIWNENNKPYFISLFITQPHDGKSLDFKNQKDEIMAQIGKEIYPFL

>sp|P13079|CARB_STRTH rRNA methyltransferase OS=Streptomyces thermotolerans OX=80858 GN=carB PE=2 SV=1

MAALLKRILRRRMAEKRSGRGRMAAARTTGAQSRKTAQRSGRSEADRRRRVHGQNFLVDRETVQRFVRFADPDPGEVVLEVGAGNGAITRELARLCRRVVAYEIDRHFADRLREATAEDPRIEVVAGDFLKTSQPKVPFSVVGNIPFGNTADIVDWCLNARRLRTTTLVTQLEYARKRTGGYRRWSRLTVATWPEVEWRMGERISRRWFRPVPAVDSAVLRLERRPVPLIPPGLMHDFRDLVETGFTGKGGSLDASLRRRFPARRVAAGFRRARLEQGVVVAYVTPGQWITLFEELHGR

>sp|P52676|NMCR_ENTCL Carbapenem-hydrolyzing beta-lactamase transcriptional activator OS=Enterobacter cloacae OX=550 GN=nmcR PE=3 SV=1

MRARLPLNALRAFEASARYLNFTKAGLELHVSQAAVSQQVRTLEQMLGVALFTRVPRGLQLTDEGMHLLPSITEALQMMSSAMDKFHEGKIKEVLTIAVVGTFAIGWLLPRITAFLNENPWIDIRILTHNNVVNLAAEGIDASIRFGTGGWINTENILLFQAPHTVLCSPETSKKLYIPSDLKKVCLLRSYRKEEWNNWFKAAGIDPWTITGPIFDSTRLMIDAVKLGDYAALVPYHMFQKELNERSVAKPFEIYATLGGYWLTLQKSRVNHNSEALNVFKEWIIEHSREFVLKS

>sp|P14509|KKA8_ECOLX Aminoglycoside 3'-phosphotransferase OS=Escherichia coli OX=562 GN=aphA PE=3 SV=1

MNDIDREEPCAAAAVPESMAAHVMGYKWARDKVGQSGCAVYRLHSKSGGSDLFLKHGKDAFADDVTDEMVRLRWLAGHISVPSVVSFVRTPNQAWLLTTAIHGKTAYQVLKSDFGARLVVVDALAAFMRRLHAIPVSECSVQQWTTHAGLPERGSIEAGVVDVDDFDKEREGWTAEQVWEAMHRLLPLAPDPVVTHGDFSLDNLLIVEGKVVGCIDVGRAGIADRYQDLAVLWNCLEEFEPSLQERLVAQYGIADPDRRKLQFHLLLDELF

>sp|Q07448|S3AD_ENTFL Streptomycin 3''-adenylyltransferase OS=Enterococcus faecalis OX=1351 GN=spc PE=4 SV=1

MRRIYLNTYEQINKVKKILRKHLKNNLIGTYMFGSGVESGLKPNSDLDFLVVVSEPLTDQSKEILIQKIRPISKKIGDKSNLRYIELTIIIQQEMVPWNHPPKQEFIYGEWLQELYEQGYIPQKELNSDLTIMLYQAKRKNKRIYGNYDLEELLPDIPFSDVRRAIMDSSEELIDNYQDDETNSILTLCRMILTMDTGKIIPKDIAGNAVAESSPLEHRERILLAVRSYLGENIEWTNENVNLTINYLNNRLKKL

>sp|P50868|CAT4_KLEAE Chloramphenicol acetyltransferase OS=Klebsiella aerogenes OX=548 GN=catB4 PE=3 SV=1

MTNYFDSPFKGKLLSEQVKNPNIKVGRYSYYSGYYHGHSFDDCARYLFPDRDDVDKLIIGSFCSIGSGASFIMAGNQGHRYDWASSFPFFYMQEEPAFSSALDAFQKAGNTVIGNDVWIGSEAMVMPGIKIGHGAVIGSRSLVTKDVEPYAIVGGNPAKKIKKRFTDEEISLLLEMEWWNWSLEKIKAAMPMLCSSNIVGLHKYWLEFAV

>sp|P50870|VATD_ENTFC Streptogramin A acetyltransferase OS=Enterococcus faecium OX=1352 GN=vatD PE=1 SV=1

MGPNPMKMYPIEGNKSVQFIKPILEKLENVEVGEYSYYDSKNGETFDKQILYHYPILNDKLKIGKFCSIGPGVTIIMNGANHRMDGSTYPFNLFGNGWEKHMPKLDQLPIKGDTIIGNDVWIGKDVVIMPGVKIGDGAIVAANSVVVKDIAPYMLAGGNPANEIKQRFDQDTINQLLDIKWWNWPIDIINENIDKILDNSIIREVIWKK

>sp|Q49157|AAC2_MYCFO Aminoglycoside 2'-N-acetyltransferase OS=Mycobacterium fortuitum OX=1766 GN=aac PE=3 SV=1

MPFQDVSAPVRGGILHTARLVHTSDLDQETREGARRMVIEAFEGDFSDADWEHALGGMHAFICHHGALIAHAAVVQRRLLYRDTALRCGYVEAVAVREDWRGQGLATAVMDAVEQVLRGAYQLGALSASDTARGMYLSRGWLPWQGPTSVLQPAGVTRTPEDDEGLFVLPVGLPAGMELDTTAEITCDWRDGDVW

>sp|P05789|EREB_ECOLX Erythromycin esterase type II OS=Escherichia coli OX=562 GN=ereB PE=4 SV=1

MRFEEWVKDKHIPFKLNHPDDNYDDFKPLRKIIGDTRVVALGENSHFIKEFFLLRHTLLRFFIEDLGFTTFAFEFGFAEGQIINNWIHGQGTDDEIGRFLKHFYYPEELKTTFLWLREYNKAAKEKITFLGIDIPRNGGSYLPNMEIVHDFFRTADKEALHIIDDAFNIAKKIDYFSTSQAALNLHELTDSEKCRLTSQLARVKVRLEAMAPIHIEKYGIDKYETILHYANGMIYLDYNIQAMSGFISGGGMQGDMGAKDKYMADSVLWHLKNPQSEQKVIVVAHNAHIQKTPILYDGFLSCLPMGQRLKNAIGDDYMSLGITSYSGHTAALYPEVDTKYGFRVDNFQLQEPNEGSVEKAISGCGVTNSFVFFRNIPEDLQSIPNMIRFDSIYMKAELEKAFDGIFQIEKSSVSEVVYE

>sp|P10051|AAC6_CITKO Aminoglycoside N(6')-acetyltransferase type 1 OS=Citrobacter koseri OX=545 GN=aacA1 PE=3 SV=1

MNYQIVNIAECSNYQLEAANILTEAFNDLGNNSWPDMTSATKEVKECIESPNLCFGLLINNSLVGWIGLRPMYKETWELHPLVVRPDYQNKGIGKILLKELENRAREQGIIGIALGTDDEYYRTSLSLITITEDNIFDSIKNIKNINKHPYEFYQKNGYYIVGIIPNANGKNKPDIWMWKSLIKE

>sp|P23181|AACC1_PSEAI Gentamicin 3-N-acetyltransferase OS=Pseudomonas aeruginosa OX=287 GN=aacC1 PE=1 SV=1

MLRSSNDVTQQGSRPKTKLGGSSMGIIRTCRLGPDQVKSMRAALDLFGREFGDVATYSQHQPDSDYLGNLLRSKTFIALAAFDQEAVVGALAAYVLPRFEQPRSEIYIYDLAVSGEHRRQGIATALINLLKHEANALGAYVIYVQADYGDDPAVALYTKLGIREEVMHFDIDPSTAT

>sp|Q54441|AAC6C_SERMA Aminoglycoside N(6')-acetyltransferase type 1 OS=Serratia marcescens OX=615 PE=1 SV=1

MIVICDHDNLDAWLALRTALWPSGSPEDHRAEMREILASPHHTAFMARGLDGAFVAFAEVALRYDYVNGCESSPVAFLEGIYTAERARRQGWAARLIAQVQEWAKQQGCSELASDTDIANLDSQRLHAALGFAETERVVFYRKTLG

>sp|Q44057|AAC6_ACIHA Aminoglycoside N(6')-acetyltransferase type 1 OS=Acinetobacter haemolyticus OX=29430 PE=1 SV=1

MNIKPASEASLKDWLELRNKLWSDSEASHLQEMHQLLAEKYALQLLAYSDHQAIAMLEASIRFEYVNGTETSPVGFLEGIYVLPAHRRSGVATMLIRQAEVWAKQFSCTEFASDAALDNVISHAMHRSLGFQETEKVVYFSKKID

>sp|Q9R381|AAC6_SALEN Aminoglycoside N(6')-acetyltransferase type 1 OS=Salmonella enteritidis OX=149539 PE=1 SV=1

MDIRQMNKTHLEHWRGLRKQLWPGHPDDAHLADGEEILQADHLASFIAMADGVAIGFADASIRHDYVNGCDSSPVVFLEGIFVLPSFRQRGVAKQLIAAVQRWGTNKGCREMASDTSPENTISQKVHQALGFEETERVIFYRKRC

>sp|P18622|STRA_STRGA Streptomycin 6-kinase OS=Streptomyces glaucescens OX=1907 GN=sph PE=3 SV=1

MSTSKLVEIPEPLAASYARAFGEEGQAWIAALPALVEELLDRWELTADGASASGEASLVLPVLRTDGTRAVLKLQLPREETSAAITGLRTWNGHGVVRLLDHDPRSSTMLLERLDASRTLASVEDDDAAMGVLAGLLARLVSVPAPRGLRGLGDIAGAMLEEVPRAVAALADPADRRLLNDWASAVAELVGEPGDRMLHWDLHYGNVLAAEREPWLAIDPEPLAGDPGFDLWPALDSRWDDIVAQRDVVRVVRRRFDLLTEVLGLDRARAAGWTYGRLLQNALWDIEDGSAALDPAAVTLAQALRGH

>sp|P13081|BLE_KLEPN Bleomycin resistance protein OS=Klebsiella pneumoniae OX=573 GN=ble PE=1 SV=1

MTDQATPNLPSRDFDSTAAFYERLGFGIVFRDAGWMILQRGDLMLEFFAHPGLDPLASWFSCCLRLDDLAEFYRQCKSVGIQETSSGYPRIHAPELQEWGGTMAALVDPDGTLLRLIQNELLAGIS

>sp|P30899|BLAC_BACVU Beta-lactamase OS=Bacteroides vulgatus OX=821 GN=cfxA PE=3 SV=1

MEKNRKKQIVVLSIALVCIFILVFSLFHKSATKDSANPPLTNVLTDSISQIVSACPGEIGVAVIVNNRDTVKVNNKSVYPMMSVFKVHQALALCNDFDNKGISLDTLVNINRDKLDPKTWSPMLKDYSGPVISLTVRDLLRYTLTQSDNNASNLMFKDMVNVAQTDSFIATLIPRSSFQIAYTEEEMSADHNKAYSNYTSPLGAAMLMNRLFTEGLIDDEKQSFIKNTLKECKTGVDRIAAPLLDKEGVVIAHKTGSGYVNENGVLAAHNDVAYICLPNNISYTLAVFVKDFKGNKSQASQYVAHISAVVYSLLMQTSVKS

>sp|P96465|BLA2_STEMA Beta-lactamase L2 OS=Stenotrophomonas maltophilia OX=40324 PE=3 SV=1

MLARRRFLQFSGAAVASSLALPLLARAAGKTAASAPTDAALTAATDFAALEKAVRGRFGVTLLDTASGRRIGHRQDERFPMCSTFKSVLAATVLSQAERQPALLDTRVPVRDADLLSHAPVTRRHAGKDMTVRDLCRATIITSDNTAANLLFGVVGGPPAVTAFLRSIGDAVSRTDRLEPELNSFAKGDPRDTTTPAAMAATLQRVVLGEVLQLASRQQLADWLIDNETGDACLRAGLGKLWRVRDKTGSNGEDARNDIAVLWPVAGGAPWVLTAYLQAGAISYEQRATVLAQVGRIADRLIG

>sp|P0A9Z9|BLA2_KLEPO Beta-lactamase SHV-2 OS=Klebsiella pneumoniae subsp. ozaenae OX=574 GN=bla PE=3 SV=1

MRYIRLCIISLLATLPLAVHASPQPLEQIKLSESQLSGRVGMIEMDLASGRTLTAWRADERFPMMSTFKVVLCGAVLARVDAGDEQLERKIHYRQQDLVDYSPVSEKHLADGMTVGELCAAAITMSDNSAANLLLATVGGPAGLTAFLRQIGDNVTRLDRWETELNEALPGDARDTTTPASMAATLRKLLTSQRLSARSQRQLLQWMVDDRVAGPLIRSVLPAGWFIADKTGASERGARGIVALLGPNNKAERIVVIYLRDTPASMAERNQQIAGIGAALIEHWQR

>sp|P28585|BLC1_ECOLX Beta-lactamase CTX-M-1 OS=Escherichia coli OX=562 GN=bla PE=1 SV=2

MVKKSLRQFTLMATATVTLLLGSVPLYAQTADVQQKLAELERQSGGRLGVALINTADNSQILYRADERFAMCSTSKVMAVAAVLKKSESEPNLLNQRVEIKKSDLVNYNPIAEKHVDGTMSLAELSAAALQYSDNVAMNKLISHVGGPASVTAFARQLGDETFRLDRTEPTLNTAIPGDPRDTTSPRAMAQTLRNLTLGKALGDSQRAQLVTWMKGNTTGAASIQAGLPASWVVGDKTGSGDYGTTNDIAVIWPKDRAPLILVTYFTQPQPKAESRRDVLASAAKIVTNGL

>sp|Q47746|VANY_ENTFA D-alanyl-D-alanine carboxypeptidase OS=Enterococcus faecalis (strain ATCC 700802 / V583) OX=226185 GN=vanYB PE=1 SV=1

MEKSNYHSNVNHHKRHMKQSGEKRAFLWAFIISFTVCTLFLGWRLVSVLEATQLPPIPATHTGSGTGVAENPEENTLATAKEQGDEQEWSLILVNRQNPIPAQYDVELEQLSNGERIDIRISPYLQDLFDAARADGVYPIVASGYRTTEKQQEIMDEKVAEYKAKGYTSAQAKAEAETWVAVPGTSEHQLGLAVDINADGIHSTGNEVYRWLDENSYRFGFIRRYPPDKTEITGVSNEPWHYRYVGIEAATKIYHQGLCLEEYLNTEK

>sp|P0AG05|S3AD_ECOLX Streptomycin 3''-adenylyltransferase OS=Escherichia coli OX=562 GN=aadA PE=4 SV=1

MREAVIAEVSTQLSEVVGVIERHLEPTLLAVHLYGSAVDGGLKPHSDIDLLVTVTVRLDETTRRALINDLLETSASPGESEILRAVEVTIVVHDDIIPWRYPAKRELQFGEWQRNDILAGIFEPATIDIDLAILLTKAREHSVALVGPAAEELFDPVPEQDLFEALNETLTLWNSPPDWAGDERNVVLTLSRIWYSAVTGKIAPKDVAADWAMERLPAQYQPVILEARQAYLGQEEDRLASRADQLEEFVHYVKGEITKVVGK

>sp|Q7N8V7|RSMA_PHOLL Ribosomal RNA small subunit methyltransferase A OS=Photorhabdus luminescens subsp. laumondii (strain DSM 15139 / CIP 105565 / TT01) OX=243265 GN=rsmA PE=3 SV=1

MNNRVHQGHFARKRFGQNFLTDQFVIDSIAAAINPQPGQAVLEIGPGLGALTEPVGERMDKMTVVELDRDLAARLQVHPQLKDKLTIIQQDAMTVNFGELSQQRGKPLRVFGNLPYNISTPLMFHLFSYTDAIADMHFMLQKEVVNRLVAGPGSKTFGRLSVMAQYYCQVIPVLEVPPTAFTPAPKVDSAVVRLVPHKSIPHPVKNIRMLSRITTQAFNQRRKTIRNSLGDLFTVEQLTELGIDPSTRAENISVEQYCKMANWLSEQPEMQS

>sp|Q7N0B9|UPPP_PHOLL Undecaprenyl-diphosphatase OS=Photorhabdus luminescens subsp. laumondii (strain DSM 15139 / CIP 105565 / TT01) OX=243265 GN=uppP PE=3 SV=1

MTDLSTLFHAAILGVVEGLTEFLPVSSTGHMIIVGHMLGFTGDKAETFEVIIQLGSILAVVVVFWRRLFGLIGIHFGEVPHEGKTNGKLKLSHIILAMLPAVTLGLMFHDVIKSLFNPQSVMYALVIGGVLLITAEILKPKTPKAEGLDDITYRQAFMIGCFQCLALWPGFSRSGATISGGMLMGVNRYTASEFSFILAVPMMMGASGLDLYKSLHFLSASDIPMFAVGFVTAFVVALVAIKTFLALIKRISFIPFAIYRFIVAAAVYWVFM

>sp|Q00982|BLO5_PSEAI Beta-lactamase OXA-5 OS=Pseudomonas aeruginosa OX=287 GN=bla PE=3 SV=1

MKTIAAYLVLVFYASTALSESISENLAWNKEFSSESVHGVFVLCKSSSNSCTTNNAARASTAYIPASTFKIPNALIGLETGAIKDERQVFKWDGKPRAMKQWEKDLKLRGAIQVSAVPVFQQIAREVGEIRMQKYLNLFSYGNANIGGGIDKFWLEGQLRISAFNQVKFLESLYLNNLPASKANQLIVKEAIVTEATPEYIVHSKTGYSGVGTESSPGVAWWVGWVEKGTEVYFFAFNMDIDNESKLPSRKSISTKIMASEGIIIGG

>sp|Q51574|BLO15_PSEAI Beta-lactamase OXA-15 OS=Pseudomonas aeruginosa OX=287 GN=bla PE=1 SV=1

MAIRIFAILFSIFSLATFAHAQEGTLERSDWRKFFSEFQAKGTIVVADERQADRAMLVFDPVRSKKRYSPASTFKIPHTLFALDAGAVRDEFQIFRWDGVNRGFAGHNQDQDLRSAMRNSTVWVYELFAKEIGDDKARRYLKKIDYGNAGPSTSNGDYWIEGSLAISAQEQIAFLRKLYRNELPFRVEHQRLVKDLMIVEAGRNWILRAKTGWEGRMGWWVGWVEWPTGSVFFALNIDTPNRMDDLFKREAIVRAILRSIEALPPNPAVNSDAAR

>sp|P10738|ERMB_ECOLX rRNA adenine N-6-methyltransferase OS=Escherichia coli OX=562 GN=ermBC PE=3 SV=1

MNKNIKYSQNFLTSEKVLNQIIKQLNLKETDTVYEIGTGKGHLTTKLAKISKQVTSIELDSHLFNLSSEKLKSNTRVTLIHQDILQFQFPNKQRYKIVGNIPYHLSTQIIKKVVFESHASDIYLIVEEGFYKRTLDIHRTLGLLLHTQVSIQQLLKLPAECFHPKPRVNSVLIKLTRHTTDVPDKYWKLYTYFVSKWVNREYRQLFTKNQFHQAMKHAKVNNLSTVTYEQVLSIFNSYLLFNGRK

>sp|Q00014|ERMG_LACRE rRNA adenine N-6-methyltransferase OS=Lactobacillus reuteri OX=1598 GN=ermGT PE=3 SV=1

MNKKNIKDSQNFITSKHHINEILRNVHLNTNDNIIEIGSGKGHFSFELAKRCNYVTAIEIDPKLCRITKNKLIEYENFQVINKDILQFKFPKNKSYKIFGNIPYNISTDIIRKIVFESTATESYLIVEYGFAKRLLNTNRSLALFLMTEVDISILSKIPREYFHPKPRVNSSLIVLKRHPSKISLKDRKQYENFVMKWVNKEYIKLFSKNQFYQALKYARIDDLNNISFEQFLSLFNSYKLFNR

>sp|P29806|AADB2_KLEPN 2''-aminoglycoside nucleotidyltransferase OS=Klebsiella pneumoniae OX=573 GN=aadB PE=4 SV=1

MDTTQVTLIHQILAAADERNLPLWIGGGWAIDARLGRVTRKHDDIDLTFPGERRGELEAMVEMLGGRVTEELDYGFLAEIGDELLDCEPAWWADEAYEIAEAPQGSCPEAAEGVIAGRPVRCNSWEAIIWDYFYYADEVPPVDWPTKHIESYRLACTSLGAEKVEVLRAAFRSRYAA

>sp|P19650|AAC6_KLEPN Aminoglycoside N(6')-acetyltransferase type 1 OS=Klebsiella pneumoniae OX=573 GN=aacA4 PE=3 SV=1

MSIQHFQTKLGITKYSIVTNSNDSVTLRLMTEHDLAMLYEWLNRSHIVEWWGGEEARPTLADVQEQYLPSVLAQESVTPYIAMLNGEPIGYAQSYVALGSGDGWWEEETDPGVRGIDQLLANASQLGKGLGTKLVRALVELLFNDPEVTKIQTDPSPSNLRAIRCYEKAGFERQGTVTTPDGPAVYMVQTRQAFERTRSVA

>sp|P37711|VANY_ENTFC D-alanyl-D-alanine carboxypeptidase OS=Enterococcus faecium OX=1352 GN=vanY PE=2 SV=1

MKKLFFLLLLLFLIYLGYDYVNEALFSQEKVEFQNYDQNPKEHLENSGTSENTQEKTITEEQVYQGNLLLINSKYPVRQESVKSDIVNLSKHDELINGYGLLDSNIYMSKEIAQKFSEMVNDAVKGGVSHFIINSGYRDFDEQSVLYQEMGAEYALPAGYSEHNSGLSLDVGSSLTKMERAPEGKWIEENAWKYGFILRYPEDKTELTGIQYEPWHIRYVGLPHSAIMKEKNFVLEEYMDYLKEEKTISVSVNGEKYEIFYYPVTKNTTIHVPTNLRYEISGNNIDGVIVTVFPGSTHTNSRR

>sp|P69413|MERR_PSESP Mercuric resistance operon regulatory protein OS=Pseudomonas sp. OX=306 GN=merR PE=3 SV=1

MENNLENLTIGVFAKAAGVNVETIRFYQRKGLLLEPDKPYGSIRRYGEADVTRVRFVKSAQRLGFSLDEIAELLRLEDGTHCEEASSLAEHKLKDVREKMADLARMEAVLSELVCACHARRGNVSCPLIASLQGGASLAGSAMP

>sp|P00808|BLAC_BACLI Beta-lactamase OS=Bacillus licheniformis OX=1402 GN=penP PE=1 SV=1

MKLWFSTLKLKKAAAVLLFSCVALAGCANNQTNASQPAEKNEKTEMKDDFAKLEEQFDAKLGIFALDTGTNRTVAYRPDERFAFASTIKALTVGVLLQQKSIEDLNQRITYTRDDLVNYNPITEKHVDTGMTLKELADASLRYSDNAAQNLILKQIGGPESLKKELRKIGDEVTNPERFEPELNEVNPGETQDTSTARALVTSLRAFALEDKLPSEKRELLIDWMKRNTTGDALIRAGVPDGWEVADKTGAASYGTRNDIAIIWPPKGDPVVLAVLSSRDKKDAKYDDKLIAEATKVVMKALNMNGK

>sp|P30180|AACC7_STRRY Aminoglycoside N(3)-acetyltransferase VII OS=Streptomyces rimosus subsp. paromomycinus OX=92743 GN=aacC7 PE=3 SV=1

MDELALLKRSDGPVTRTRLARDLTALGLGDGDTVMFHTRMSAVGYVAGGPETVIGALRDVVGERGTLMVTCGWNDAPPYDFTDWPQTWQDARRAEHPAYDPVLSEADHNNGRLPEALRRRPGAVRSRHPDASFAALGAAATALTADHPWDDPHGPDSPLARLVAMGGRVLLLGAPLEALTLLHHAEALADAPGKRFVDYEQPILVDGERVWRRFHDIDSEDGAFDYSALVPEGTEAFEIIGRDMRAAGIGRRGTVGAADSHLFEARDVVDFGVAWMEEKLGRERGPGG

>sp|Q02652|TETM_STRLI Tetracycline resistance protein TetM OS=Streptomyces lividans OX=1916 GN=tetM PE=3 SV=1

MRTLNIGILAHVDAGKTSLTERLLFDHGAVDRLGSVDAGDTRTVDGGIERRRGITIRSAVAAFTVGDTRVNLIDTPGHSDFVAEVERALEVLDGAVLLLSAVEGVQARTRVLMRALRRLRLPTIVFVNKIDRAGARTDGLLGDVRRLLTPHVAPLTEVADAGTPRARVTRRPPDGRTAEALAEVDTEVLAALVDGPEPTGEDVARALAARTADGSFHPLYHGSALGGQGVAELVEGLLGLIPAATPGTSGGTSGGTEPRGTVFAVRPGPAGERTAYLRLYGGEVHPRRRLTFLRRESDGRTTEVSGRVTRLDVVGGDATLTAGNIAALTVPGGLRVGDRLGGPTDRAPQFAPPTLQTLVRARHPEQAAPLRSALLALADQDPLLHARPAASGATALLLYGEVQMEVLAATLAEDFGIEAEFTPGRVRFLERPAGTDEAAEEMPWLDRTRYFATIGLRVEPGPRGSGGAFGYETELGALPRAFHQAVEETVHDTLRTGLTGAAVTDYRVTLIRSGFSSPLSTAADFRGLTPLVLRRALARAGTVLHEPYQAFEAEVPADTLAAVTALLASLGADFTGTTGGDPAWIVTGELPARRVREAELRLPGLTHGEAVWSSRPCEDRPLKAGNSGPGTGVGGHSGE

>sp|B3U538|BL133_ACIRA Beta-lactamase OXA-133 OS=Acinetobacter radioresistens OX=40216 GN=blaOXA-133 PE=2 SV=1

MNKYFTCYVVASLFFSGCTVQHNLINETQSQIVQGHNQVIHQYFDEKNTSGVLVIQTDKKINLYGNALSRANTEYVPASTFKMLNALIGLENQKTDINEIFKWKGEKRSFTTWEKDMTLGEAMKLSAVPVYQELARRIGLDLMQKEVERIDFGNAEIGQQVDNFWLIGPLKVTPIQEVEFVSQLAHTQLPFSEKVQANVKNMLLLEENNGYKIFGKTGWAMDIKPQVGWLTGWVEQPDGKIVAFALNMEMRSEMPASIRNELLMKSLKQLNII

>sp|P24735|AMPC_PSEAE Beta-lactamase OS=Pseudomonas aeruginosa (strain ATCC 15692 / DSM 22644 / CIP 104116 / JCM 14847 / LMG 12228 / 1C / PRS 101 / PAO1) OX=208964 GN=ampC PE=1 SV=2

MRDTRFPCLCGIAASTLLFATTPAIAGEAPADRLKALVDAAVQPVMKANDIPGLAVAISLKGEPHYFSYGLASKEDGRRVTPETLFEIGSVSKTFTATLAGYALTQDKMRLDDRASQHWPALQGSRFDGISLLDLATYTAGGLPLQFPDSVQKDQAQIRDYYRQWQPTYAPGSQRLYSNPSIGLFGYLAARSLGQPFERLMEQQVFPALGLEQTHLDVPEAALAQYAQGYGKDDRPLRVGPGPLDAEGYGVKTSAADLLRFVDANLHPERLDRPWAQALDATHRGYYKVGDMTQGLGWEAYDWPISLKRLQAGNSTPMALQPHRIARLPAPQALEGQRLLNKTGSTNGFGAYVAFVPGRDLGLVILANRNYPNAERVKIAYAILSGLEQQGKVPLKR

>sp|P05364|AMPC_ENTCL Beta-lactamase OS=Enterobacter cloacae OX=550 GN=ampC PE=1 SV=1

MMRKSLCCALLLGISCSALATPVSEKQLAEVVANTITPLMKAQSVPGMAVAVIYQGKPHYYTFGKADIAANKPVTPQTLFELGSISKTFTGVLGGDAIARGEISLDDAVTRYWPQLTGKQWQGIRMLDLATYTAGGLPLQVPDEVTDNASLLRFYQNWQPQWKPGTTRLYANASIGLFGALAVKPSGMPYEQAMTTRVLKPLKLDHTWINVPKAEEAHYAWGYRDGKAVRVSPGMLDAQAYGVKTNVQDMANWVMANMAPENVADASLKQGIALAQSRYWRIGSMYQGLGWEMLNWPVEANTVVEGSDSKVALAPLPVAEVNPPAPPVKASWVHKTGSTGGFGSYVAFIPEKQIGIVMLANTSYPNPARVEAAYHILEALQ

>sp|Q03170|BLP1_PSEAI Beta-lactamase PSE-1 OS=Pseudomonas aeruginosa OX=287 GN=pse1 PE=1 SV=1

MKFLLAFSLLIPSVVFASSSKFQQVEQDVKAIEVSLSARIGVSVLDTQNGEYWDYNGNQRFPLTSTFKTIACAKLLYDAEQGKVNPNSTVEIKKADLVTYSPVIEKQVGQAITLDDACFATMTTSDNTAANIILSAVGGPKGVTDFLRQIGDKETRLDRIEPDLNEGKLGDLRDTTTPKAIASTLNKFLFGSALSEMNQKKLESWMVNNQVTGNLLRSVLPAGWNIADRSGAGGFGARSITAVVWSEHQAPIIVSIYLAQTQASMAERNDAIVKIGHSIFDVYTSQSR

>sp|P06548|BLA3_BACCE Beta-lactamase 3 OS=Bacillus cereus OX=1396 GN=blaZ PE=1 SV=1

MFVLNKFFTNSHYKKIVPVVLLSCATLIGCSNSNTQSESNKQTNQTNQVKQENKRNHAFAKLEKEYNAKLGIYALDTSTNQTVAYHADDRFAFASTSKSLAVGALLRQNSIEALDERITYTRKDLSNYNPITEKHVDTGMTLKELADASVRYSDSTAHNLILKKLGGPSAFEKILREMGDTVTNSERFEPELNEVNPGETHDTSTPKAIAKTLQSFTLGTVLPSEKRELLVDWMKRNTTGDKLIRAGVPKGWEVADKTGAGSYGTRNDIAIIWPPNKKPIVLSILSNHDKEDAEYDDTLIADATKIVLETLKVTNK

>sp|P80545|BLAC_SERFO Beta-lactamase OS=Serratia fonticola OX=47917 PE=1 SV=1

QPANAKANIQQQLSELEKNSGGRLGVALIDTADNSQILYRGDERFPMCSTSKVMAVSALLKQSETDKNLLAKRMEIKQSDLVNYNPIAEKHLDTGMTLAEFSAATIQYSDNTAMNKILEHLGGPAKVTEFARTIGDKTFRLDRTEPTLNTAIPGDKRDTTSPQAMAISLQNLTLGKALAEPQRAQLVEWMKGNTTGGASIRAGLPTTWVVGDKTGSGDYGTTNDIAVIWPANHAPLVLVTYFTQPQQNAEARKDVLAAAAKIVTAGL

>sp|P22390|BLAC_CITKO Beta-lactamase OS=Citrobacter koseri OX=545 PE=1 SV=2

MFKKRGRQTVLIAAVLAFFTASSPLLARTQGEPTQVQQKLAALEKQSGGRLGVALINTADRSQILYRGDERFAMCSTSKTMVAAAVLKQSETQHDILQQKMVIKKADLTNWNPVTEKYVDKEMTLAELSAATLQYSDNTAMNKLLEHLGGTSNVTAFARSIGDTTFRLDRKEPELNTAIPGDERDTTCPLAMAKSLHKLTLGDALAGAQRAQLVEWLKGNTTGGQSIRAGLPEGWVVGDKTGAGDYGTTNDIAVIWPEDRAPLILVTYFTQPQQDAKGRKDILAAAAKIVTEGL

>sp|P18623|VPH_STRVI Viomycin phosphotransferase OS=Streptomyces vinaceus OX=1960 GN=vph PE=3 SV=1

MRIIETHRDLLSRLLPGDTVGGLAVHEGQFHHVVIGSHRVVCFARTRAAADRLPGRADVLRALAGIDLGFRTPQPLSEGGAQGTDEPPYLVLSRIPGAPLEDDVLTSPEVAEAVARQYATLLSGLAAAGDEEKVRAALPEAPANEWQEFATGVRTELFPLMSDGGRERAERELAALDALPHLTSAVVHGDLGGENVLWETVDGVPRMSGVVDWDEVGIGDPAEDLAAIGASYGEELLGRVLALGGWADNGTAERISAIRGTFALQQALYAQRDGDEEELADGLSGYR

>sp|Q0K7S4|UPPP_CUPNH Undecaprenyl-diphosphatase OS=Cupriavidus necator (strain ATCC 17699 / H16 / DSM 428 / Stanier 337) OX=381666 GN=uppP PE=3 SV=2

MEIALALKAVILGIVEGLTEFLPISSTGHLILAGQLLDFNDEKGKIFEIVIQFGAILAVCWEFRARIGNVVRGLRAEPLAQRFAANVVIASVPAIVLAFIFGKWIKAHLFNPISVALAFIVGGVVILLAEWRDARRGTVSHPQGNALLEAAKAGAPRIESVDDLNWRDALKVGLAQCFALVPGTSRSGATIIGGMLFGLSRQVATEFSFFLAIPVIFGATVYELYKARALLNGDDLGIFAVGFVFAFLSAFLCVRWLLRFVATHDFKPFAWYRIAFGIVVLLTAYSGLVSWHA

>sp|P13661|BLO1_ECOLX Beta-lactamase OXA-1 OS=Escherichia coli OX=562 GN=bla PE=1 SV=2

MKNTIHINFAIFLIIANIIYSSASASTDISTVASPLFEGTEGCFLLYDASTNAEIAQFNKAKCATQMAPDSTFKIALSLMAFDAEIIDQKTIFKWDKTPKGMEIWNSNHTPKTWMQFSVVWVSQEITQKIGLNKIKNYLKDFDYGNQDFSGDKERNNGLTEAWLESSLKISPEEQIQFLRKIINHNLPVKNSAIENTIENMYLQDLDNSTKLYGKTGAGFTANRTLQNGWFEGFIISKSGHKYVFVSALTGNLGSNLTSSIKAKKNAITILNTLNL

>sp|Q8CTJ6|UPPP_STAES Undecaprenyl-diphosphatase OS=Staphylococcus epidermidis (strain ATCC 12228) OX=176280 GN=uppP PE=3 SV=1

MFLLELIKGIILGIVEGLTEFAPVSSTGHMILVDDMWLKSTNFLGSQSAFTFKVVIQLGSVFAAAWVFRERFLEILHIGQHKPEPSTSGDRRSKPRRLNLIHVLVGMVPAGILGFLFDDLIEKYLFSVPTVLIGLFIGAIYMIIADKYSKTVQHPQTVDQINYFQAFVIGISQAIAMWPGFSRSGSTISTGVLMKLNHKAASDFTFIMSVPIMLAASGLSLLKHYEYIHLAHIPFYILGFLAAFIVGLIAIKTFLHLINKVKLVPFAIYRIVLVIFIAILYFGFGIGKGI

>sp|Q51355|BLC4_PSEAI Beta-lactamase CARB-4 OS=Pseudomonas aeruginosa OX=287 GN=carB4 PE=3 SV=1

MKLLLVFSLLIPSMVFANSSKFQQVEQDAKVIEASLSAHIGISVLDTQTGEYWDYNGNQRFPLTSTFKTIACAKLLYDAEQGEINPKSTIEIKKADLVTYSPVIEKQVGQAITLDDACFATMTTSDNAAANIILNALGGPESVTDFLRQIGDKETRLDRIEPELNEGKLGDLRDTTTPNAIVNTLNELLFGSTLSQDGQKKLEYWMVNNQVTGNLLRSVLPEGWNIADRSGAGGFGARSITAVVWSEAQSPIIVSIYLAQTEASIADRNDAIVKIGRSIFEVYSSQSR

>sp|Q831R1|UPPP1_ENTFA Undecaprenyl-diphosphatase OS=Enterococcus faecalis (strain ATCC 700802 / V583) OX=226185 GN=uppP PE=3 SV=1

MLFANLWKAIILGIIEGITEWLPISSTGHLILVDEFIKLDLSKDFMEMFNVVIQLGAIMAVVILYFHKLNPFSPKKNGEEKKDTWILWSKVLVACLPAAVIGLKFDDYLDAHFYNFLTVSIMLIVYGIAFIIIEKRNKNVAPKCTNLKDFTYKAALIVGAFQVLALIPGTSRSGATILGAILIGASRFVATEFSFFLGIPVMFGASFLKIFKFLAKGNTFGSEEIIILITGSIVAFVVSIIAIKFLLNYLKKNDFTVFGWYRVILGAILIGYWLFS

>sp|P58740|UPPP1_AGRFC Undecaprenyl-diphosphatase 1 OS=Agrobacterium fabrum (strain C58 / ATCC 33970) OX=176299 GN=uppP1 PE=3 SV=1

MGDQSIISALLLGIIEGLTEFIPVSSTAHVLLAGHFLGFKSPGNTFAVLIQLGAILAILLVYFQKLVSIAVAMPTSAKARRFVLAVLVAFLPAAVIGALAHDFIKTVLFETPMLICVVLIIGGFILLAVDRMPLKPKYTDIMDYPPSLAFKIGLFQCLAMIPGTSRSGATIVGALLMGTDKRSAAEFSFFLAMPTMLGAFVLDLYKNRDALSFDDSALIAVGFVAAFVSGLFVVRSLLDFVSRRGFAPFAWWRIVIGALGLVALLVIG

>sp|A5W499|UPPP_PSEP1 Undecaprenyl-diphosphatase OS=Pseudomonas putida (strain ATCC 700007 / DSM 6899 / BCRC 17059 / F1) OX=351746 GN=uppP PE=3 SV=1

MDFWTAFQAIILGVVEGLTEFLPISSTGHQIIVADLIGFGGERAMAFNIIIQLAAILAVVWEFRSKIFEVVFGLTHQPKARRFTGNLLLAFMPAVVLGVLFADLIHEYLFNPVTVAAALVVGGVIMLWAERRKHRVEVDHVDDMRWSHALKIGFIQCLAMIPGTSRSGSTIIGGLLFGLSRKAATEFSFFLAMPTMVGAAVYSGYKYRDLFQPGDLPVFALGFVTSFIFAMIAVRALLKFIANHSYAAFAWYRIVFGLFILATWQFGWVDWSTAHG

>sp|P08988|AACC4_SALSP Aminoglycoside N(3)-acetyltransferase IV OS=Salmonella sp. OX=599 GN=aacC4 PE=3 SV=2

MQYEWRKAELIGQLLNLGVTPGGVLLVHSSFRSVRPLEDGPLGLIEALRAALGPGGTLVMPSWSGLDDEPFDPATSPVTPDLGVVSDTFWRLPNVKRSAHPFAFAAAGPQAEQIISDPLPLPPHSPASPVARVHELDGQVLLLGVGHDANTTLHLAELMAKVPYGVPRHCTILQDGKLVRVDYLENDHCCERFALADRWLKEKSLQKEGPVGHAFARLIRSRDIVATALGQLGRDPLIFLHPPEGGMRRMRCRSPVDWLSS

>sp|P62594|BLAT_SALTI Beta-lactamase TEM OS=Salmonella typhi OX=90370 GN=bla PE=3 SV=1

MSIQHFRVALIPFFAAFCLPVFAHPETLVKVKDAEDQLGARVGYIELDLNSGKILESFRPEERFPMMSTFKVLLCGAVLSRVDAGQEQLGRRIHYSQNDLVEYSPVTEKHLTDGMTVRELCSAAITMSDNTAANLLLTTIGGPKELTAFLHNMGDHVTRLDRWEPELNEAIPNDERDTTMPAAMATTLRKLLTGELLTLASRQQLIDWMEADKVAGPLLRSALPAGWFIADKSGAGERGSRGIIAALGPDGKPSRIVVIYTTGSQATMDERNRQIAEIGASLIKHW

>sp|P9WQG9|AAC2_MYCTU Aminoglycoside 2'-N-acetyltransferase OS=Mycobacterium tuberculosis (strain ATCC 25618 / H37Rv) OX=83332 GN=aac PE=1 SV=1

MHTQVHTARLVHTADLDSETRQDIRQMVTGAFAGDFTETDWEHTLGGMHALIWHHGAIIAHAAVIQRRLIYRGNALRCGYVEGVAVRADWRGQRLVSALLDAVEQVMRGAYQLGALSSSARARRLYASRGWLPWHGPTSVLAPTGPVRTPDDDGTVFVLPIDISLDTSAELMCDWRAGDVW

>sp|A9VRT9|FOSB_BACWK Metallothiol transferase FosB OS=Bacillus weihenstephanensis (strain KBAB4) OX=315730 GN=fosB PE=3 SV=1

MLKGINHLCFSVSNLENSITFYEKVLEGELLVKGRKLAYFNICGVWIALNEETHIPRKEIHQSYTHLAFSVEQKDFERLLHRLEENNVHILQGRERDVRDCESIYFVDPDGHKFEFHSGTLQDRLNYYRDEKPHMTFY

>sp|B9IY29|FOSB_BACCQ Metallothiol transferase FosB OS=Bacillus cereus (strain Q1) OX=361100 GN=fosB PE=3 SV=1

MLKGINHLCFSVSNLEDSITFYEKVLEGELLVRGRKLAYFNICGVWIALNEEIHIPRNEIHQSYTHIAFSVEQKDFERLLQRLEENDVHILQGRERDVRDCESIYFVDPDGHKFEFHSGTLQDRLNYYREGKPHMTFY

>sp|P04190|BLA2_BACCE Metallo-beta-lactamase type 2 OS=Bacillus cereus OX=1396 GN=blm PE=1 SV=1

MKKNTLLKVGLCVGLLGTIQFVSTISSVQASQKVEKTVIKNETGTISISQLNKNVWVHTELGSFNGEAVPSNGLVLNTSKGLVLVDSSWDDKLTKELIEMVEKKFQKRVTDVIITHAHADRIGGIKTLKERGIKAHSTALTAELAKKNGYEEPLGDLQTVTNLKFGNMKVETFYPGKGHTEDNIVVWLPQYNILVGGCLVKSTSAKDLGNVADAYVNEWSTSIENVLKRYRNINAVVPGHGEVGDKGLLLHTLDLLK

>sp|Q02736|CAT_CLOBU Chloramphenicol acetyltransferase OS=Clostridium butyricum OX=1492 GN=catB PE=3 SV=2

MNFNLIDINHWSRKPYFEHYLNNVKCTYSMTANIEITDLLYEIKLKNIKFYPTLIYMIATVVNNHKEFRICFDHKGSLGYWDSMNPSYTIFHKENETFSSIWTEYNKSFLRFYSDYLDDIKNYGNIMKFTPKSNEPDNTFSVSSIPWVSFTGFNLNVYNEGTYLIPIFTAGKYFKQENKIFIPISIQVHHAICDGYHASRFINEMQELAFSFQEWLENK

>sp|P07641|CAT_PROMI Chloramphenicol acetyltransferase OS=Proteus mirabilis OX=584 GN=cat PE=3 SV=1

MDTKRVGILVVDLSQWGRKEHFEAFQSFAQCTFSQTVQLDITSLLKTVKQNGYKFYPTFIYIISLLVNKHAEFRMAMKDGELVIWDSVNPGYNIFHEQTETFSSLWSYYHKDINRFLKTYSEDIAQYGDDLAYFPKEFIENMFFVSANPWVSFTSFNLNMANINNFFAPVFTIGKYYTQGDKVLMPLAIQVHHAVCDGFHVGRLLNEIQQYCDEGCK

>sp|P57296|PBPB_BUCAI Penicillin-binding protein 1B OS=Buchnera aphidicola subsp. Acyrthosiphon pisum (strain APS) OX=107806 GN=mrcB PE=3 SV=1

MFFNFKKYFLIKVFFFVLILTLCYGLYLYVKINRFINGKVWNFPTSIYGRIVNLEPGNSYSQKEVLHLLKSTMYRKVDLVMLPGEYSIKNNTIEFIRRAFDFPDIREDEFHARLYFNKDTLVKIKNIDNNHDFSFFRLEPKLIAMLKSPEAKKRMFIPRNQYPEMLVKTLLAIEDKYFYEHDGIHLSSIGRAFLVNLMAGRTIQGGSTLTQQLIKNLFLTNTRSILRKINEIYMALILDRFYTKDRILELYLNEVYLGQDGDEQIRGFPLASIYYFGRPINELNLEQYALLVGMVKGASLYSPWTNPNLALKRRNLVLFLLYKQKYITRKIYKDLCKRSLNVQPKGNIISSHPSFIQLVCEEFHKKIYNPIKNFPGTKIFTTLDYTSQNAVEQAVKIEIPILKRKKRLKDLEVAMIVIDRFTGEVQALIGSSKPEFNGYNRALKTRRSIGSLSKPITYLTALSQPEKYHLNTWISNYPLSIKLDSGQYWTPKNNNFSFSKKVLLLDALIHSINIPTVNLSINIGLKKLVDSWLLLGISKKYITPLPSISLGAINLTPFEIAQVFQIIGSGGYKSSLSSVRSIISDDGKVLYQNLPQSIHIESSEASYLTLYGMQQVVKSGTAKSLGTIFKEFSLAGKTGTTNNLVDNWFVGIDGKQIVITWIGRDNNHTTRLYSSSGAMQIYKRYLQYQRPVPLVLKAPNNINMFYINNLGELFCKKNNQHNRMLPIWSIKNKKICNDKLSERFSIKKKKNFLFWLKNLF

>sp|P18539|AMPC_SERMA Beta-lactamase OS=Serratia marcescens OX=615 GN=ampC PE=3 SV=1

MTKMNRCAALIAALILPTAHAAQQQDIDAVIQPLMKKYGVPGMAIAVSVDGKQQIYPYGVASKQTGKPITEQTLFEVGSLSKTFTATLAVYAQQQSKLSFKDPASHYLPDVRGSAFDGVSLLNLATHTSGLPLFVPDDVTNNAQLMAYYRAWQPKHPAGSYRVYSNLGIGMLGMIAAKSLDQPFIQAMEQGMLPALGMSHTYVQVPAAQMANYAQGYSKDDKPVRVNPGPLDAESYGIKSNARDLIRYLDANLQQVKVASVARRWPRRTSVITSAGAFTQDLMWENYPYPVKLSRLIEGNNAGMIMNGTPATAITPPQPELRAGWYNKTGSTGGFSTYAVFIPAKNIAVEMLANKWFPNDDRVEAAYHIIQALEKR

>sp|Q06240|VANS_ENTFC Sensor protein VanS OS=Enterococcus faecium OX=1352 GN=vanS PE=3 SV=1

MVIKLKNKKNDYSKLERKLYMYIVAIVVVAIVFVLYIRSMIRGKLGDWILSILENKYDLNHLDAMKLYQYSIRNNIDIFIYVAIVISILILCRVMLSKFAKYFDEINTGIDVLIQNEDKQIELSAEMDVMEQKLNTLKRTLEKREQDAKLAEQRKNDVVMYLAHDIKTPLTSIIGYLSLLDEAPDMPVDQKAKYVHITLDKAYRLEQLIDEFFEITRYNLQTITLTKTHIDLYYMLVQMTDEFYPQLSAHGKQAVIHAPEDLTVSGDPDKLARVFNNILKNAAAYSEDNSIIDITAGLSGDVVSIEFKNTGSIPKDKLAAIFEKFYRLDNARSSDTGGAGLGLAIAKEIIVQHGGQIYAESNDNYTTFRVELPAMPDLVDKRRS

>sp|P29753|VANC_ENTGA Vancomycin C-type resistance protein VanC OS=Enterococcus gallinarum OX=1353 GN=vanC PE=2 SV=1

MKKIAVLFGGNSPEYSVSLTSAASVIQAIDPLKYEVMTIGIAPTMDWYWYQGNLANVRNDTWLEDHKNCHQLTFSSQGFILGEKRIVPDVLFPVLHGKYGEDGCIQGLLELMNLPYVGCHVAASALCMNKWLLHQLADTMGIASAPTLLLSRYENDPATIDRFIQDHGFPIFIKPNEAGSSKGITKVTDKTALQSALTTAFAYGSTVLIQKAIAGIEIGCGILGNEQLTIGACDAISLVDGFFDFEEKYQLISATITVPAPLPLALESQIKEQAQLLYRNLGLTGLARIDFFVTNQGAIYLNEINTMPGFTGHSRYPAMMAEVGLSYEILVEQLIALAEEDKR

>sp|P37321|BLE1_PSEAI Extended-spectrum beta-lactamase PER-1 OS=Pseudomonas aeruginosa OX=287 GN=per1 PE=1 SV=1

MNVIIKAVVTASTLLMVSFSSFETSAQSPLLKEQIESIVIGKKATVGVAVWGPDDLEPLLINPFEKFPMQSVFKLHLAMLVLHQVDQGKLDLNQTVIVNRAKVLQNTWAPIMKAYQGDEFSVPVQQLLQYSVSHSDNVACDLLFELVGGPAALHDYIQSMGIKETAVVANEAQMHADDQVQYQNWTSMKGAAEILKKFEQKTQLSETSQALLWKWMVETTTGPERLKGLLPAGTVVAHKTGTSGIKAGKTAATNDLGIILLPDGRPLLVAVFVKDSAESSRTNEAIIAQVAQTAYQFELKKLSALSPN

>sp|P14171|BLAC_RHOCA Beta-lactamase OS=Rhodobacter capsulatus OX=1061 PE=3 SV=1

MRFTATVLSRVATGLALGLSMATASLAETPVEALSETVARIEEQLGARVGLSLMETGTGWSWSHREDELFLMNSTVKVPVCGAILARWDAGRLSLSDALPVRKADLVPYAPVTETRVGGNMTLDELCLAAIDMSDNVAANILIGHLGGPEAVTQFFRSVGDPTSRLDRIEPKLNDFASGDERDTTSPAAMSETLRALLLGDVLSPEARGKLAEWMRHGGVTGALLRAEAEDAWLILDKSGSGSHTRNLVAVIQPEGGAPWIATMFISDTDAEFEVRNEALKDLGRAVVAVVRE

>sp|P29809|AACC8_STRFR Aminoglycoside N(3)-acetyltransferase VIII OS=Streptomyces fradiae OX=1906 GN=aacC8 PE=3 SV=1

MDEKELIERAGGPVTRGRLVRDLEALGVGAGDTVMVHTRMSAIGYVVGGPQTVIDAVRDAVGADGTLMAYCGWNDAPPYDLAEWPPAWREAARAEWPAYDPLLSEADRGNGRVPEALRHQPGAVRSRHPDASFVAVGPAAHPLMDDHPWDDPHGPDSPLARLAGAGGRVLLLGAPLDTLTLLHHAEARAEAPGKRFVAYEQPVTVGGRRVWRRFRDVDTSRGVPYGRVVPEGVVPFTVIAQDMLAAGIGRTGRVAAAPVHLFEAADVVRFGVEWIESRMGGAAGGA

>sp|P16897|BLP4_PSEAI Beta-lactamase PSE-4 OS=Pseudomonas aeruginosa OX=287 GN=pse4 PE=1 SV=1

MKFLLAFSLLIPSVVFASSSKFQQVEQDVKAIEVSLSARIGVSVLDTQNGEYWDYNGNQRFPLTSTFKTIACAKLLYDAEQGKVNPNSTVEIKKADLVTYSPVIEKQVGQAITLDDACFATMTTSDNTAANIILSAVGGPKGVTDFLRQIGDKETRLDRIEPDLNEGKLGDLRDTTTPKAIASTLNKFLFGSALSEMNQKKLESWMVNNQVTGNLLRSVLPAGWNIADRSGAGGFGARSITAVVWSEHQAPIIVSIYLAQTQASMEERNDAIVKIGHSIFDVYTSQSR

>sp|P29810|AACC9_MICCH Aminoglycoside N(3)-acetyltransferase IX OS=Micromonospora chalcea OX=1874 GN=aacC9 PE=3 SV=1

MEEMSLLNHSGGPVTRSRIKHDLADLGLKDGDVVIFHTRMSAIGYVAGGTQTIIGALLDVVGARGTLMVPCGWNNAPPYDFLDWPRDWQDALRAEHPAYDPDLSEADYNNGRLPEALPRWPGAIRSRHPDASFAALGPAAAELMAEHPWDHPHGPDTPLARLIAHSGRVLLLGAPLDTMTLLHHAEALADVRSKRFVTYEQPILVNGQRVWRQFRDIDSEEGAFDYSTVRRGVEPFEAIARDMLSAGIGRQGRVGAADSYLFDAGPVFNFAINWIEAKLKR

>sp|Q00983|BLL1_PSEAI Beta-lactamase LCR-1 OS=Pseudomonas aeruginosa OX=287 GN=lcr1 PE=3 SV=1

MLKSTLLAFGLFIALSARAENQAIAKLFLRAGVDGTIVIESLTTGQRLVHNDPRAQQRYPAASTFKVLNTLIALEEGAISGENQIFHWNGTQYSIANWNQDQTLDSAFKVSCVWCYQQIALRVGALKYPAYIQQTNYGHLLEPFNGTEFWLDGSLTISAEEQVAFLRQVVERKLPFKASSYDSLKKVMFADENAQYRLYAKTGWATRMTPSVGWYVGYVEAKDDVWLFALNLATRDANDLPLRTQIAKDALKAIGAFPTK

>sp|P0A3M2|BLA5_PSEAI Beta-lactamase SHV-5 OS=Pseudomonas aeruginosa OX=287 GN=bla PE=3 SV=1

MRYIRLCIISLLATLPLAVHASPQPLEQIKLSESQLSGRVGMIEMDLASGRTLTAWRADERFPMMSTFKVVLCGAVLARVDAGDEQLERKIHYRQQDLVDYSPVSEKHLADGMTVGELCAAAITMSDNSAANLLLATVGGPAGLTAFLRQIGDNVTRLDRWETELNEALPGDARDTTTPASMAATLRKLLTSQRLSARSQRQLLQWMVDDRVAGPLIRSVLPAGWFIADKTGASKRGARGIVALLGPNNKAERIVVIYLRDTPASMAERNQQIAGIGAALIEHWQR

>sp|P00485|CAT1_STAAU Chloramphenicol acetyltransferase OS=Staphylococcus aureus OX=1280 GN=cat PE=2 SV=1

MNFNKIDLDNWKRKEIFNHYLNQQTTFSITTEIDISVLYRNIKQEGYKFYPAFIFLVTRVINSNTAFRTGYNSDGELGYWDKLEPLYTIFDGVSKTFSGIWTPVKNDFKEFYDLYLSDVEKYNGSGKLFPKTPIPENAFSLSIIPWTSFTGFNLNINNNSNYLLPIITAGKFINKGNSIYLPLSLQVHHSVCDGYHAGLFMNSIQELSDRPNDWLL

>sp|P05051|AMPR_ENTCL HTH-type transcriptional activator AmpR OS=Enterobacter cloacae OX=550 GN=ampR PE=3 SV=3

MTRSYLPLNSLRAFEAAARHLSFTHAAIELNVTHSAISQHVKTLEQHLNCQLFVRVSRGLMLTTEGENLLPVLNDSFDRIAGMLDRFANHRAQEKLKIGVVGTFATGVLFSQLEDFRRGYPHIDLQLSTHNNRVDPAAEGLDYTIRYGGGAWHGTEAEFLCHAPLAPLCTPDIAASLHSPADILRFTLLRSYRRDEWTAWMQAAGEHPPSPTHRVMVFDSSVTMLEAAQAGVGIAIAPVDMFTHLLASERIVQPFATQIELGSYWLTRLQSRAETPAMREFSRWLVEKMKK

>sp|Q04515|DYR10_ECOLX Dihydrofolate reductase type A10 OS=Escherichia coli OX=562 GN=dfrA10 PE=3 SV=1

MNISLIFANELITRAFGNQGKLPWQFIKEDMQFFQKTTENSVVVMGLNTWRSLPKMKKLGRDFIVISSTITEHEVLNNNIQIFKSFESFLEAFRDTTKPINVIGGVGLLSEAIEHASTVYMSSIHMVKPVHADVYVPVELMNKLYSDFKYPENILWVGDPIDSVYSLSIDKFVRPASLVGVPNDINT

>sp|P0A0U9|PBP2_NEIMB Probable peptidoglycan D,D-transpeptidase PenA OS=Neisseria meningitidis serogroup B (strain MC58) OX=122586 GN=penA PE=3 SV=1

MLIKSEYKPRMLPKEEQVKKPMTSNGRISFVLMAIAVLFAGLIARGLYLQTVTYNFLKEQGDNRIVRTQTLPATRGTVSDRNGAVLALSAPTESLFAVPKEMKEMPSAAQLERLSELVDVPVDVLRNKLEQKGKSFIWIKRQLDPKVAEEVKALGLENFVFEKELKRHYPMGNLFAHVIGFTDIDGKGQEGLELSLEDSLHGEDGAEVVLRDRQGNIVDSLDSPRNKAPKNGKDIILSLDQRIQTLAYEELNKAVEYHQAKAGTVVVLDARTGEILALANTPAYDPNRPGRADSEQRRNRAVTDMIEPGSAIKPFVIAKALDAGKTDLNERLNTQPYKIGPSPVRDTHVYPSLDVRGIMQKSSNVGTSKLSARFGAEEMYDFYHELGIGVRMHSGFPGETAGLLRNWRRWRPIEQATMSFGYGLQLSLLQLARAYTALTHDGVLLPVSFEKQAVAPQGKRIFKESTAREVRNLMVSVTEPGGTGTAGAVDGFDVGAKTGTARKFVNGRYADNKHIATFIGFAPAKNPRVIVAVTIDEPTAHGYYGGVVAGPPFKKIMGGSLNILGISPTKPLTAAAVKTPS

>sp|P38422|DACF_BACSU D-alanyl-D-alanine carboxypeptidase DacF OS=Bacillus subtilis (strain 168) OX=224308 GN=dacF PE=2 SV=2

MKRLLSTLLIGIMLLTFAPSAFAKQDGKRTSELAHEAKSAVLIERDTGKVLYNKNSNERLAPASMTKIMTMLLIMEALDKGKIKMSDKVRTSEHAASMGGSQIFLEPGEEMTVKEMLKGIAIASGNDASVAMAEFISGSEEEFVKKMNKKAKELGLKNTSFKNPTGLTEEGHYSSAYDMAIMAKELLKYESITKFTGTYEDYLRENTDKKFWLVNTNRLIKFYPGVDGVKTGYTGEAKYCLTASAKKGNMRAIAVVFGASTPKERNAQVTKMLDFAFSQYETHPLYKRNQTVAKVKVKKGKQKFIELTTSEPISILTKKGEDMNDVKKEIKMKDNISAPIQKGQELGTLVLKKDGEVLAESPVAAKEDMKKAGFITFLKRTMGDWTKFK

>sp|A2AXI2|CFR_STAWA Ribosomal RNA large subunit methyltransferase Cfr OS=Staphylococcus warneri OX=1292 GN=cfr PE=3 SV=1

MNFNNKTKYGKIQEFLRSNNEPDYRIKQITNAIFKQRISRFEDMKVLPKLLREDLINNFGETVLNIKLLAEQNSEQVTKVLFEVSKNERVETVNMKYKAGWESFCISSQCGCNFGCKFCATGDIGLKKNLTVDEITDQVLYFHLLGHQIDSISFMGMGEALANRQVFDALDSFTDPNLFALSPRRLSISTIGIIPSIKKITQEYPQVNLTFSLHSPYSEERSKLMPINDRYPIDEVMNILDEHIRLTSRKVYIAYIMLPGVNDSLEHANEVVSLLKSRYKSGKLYHVNLIRYNPTISAPEMYGEANEGQVEAFYKVLKSAGIHVTIRSQFGIDIDAACGQLYGNYQNSQ

>sp|P40883|PCHR_PSEAE Regulatory protein PchR OS=Pseudomonas aeruginosa (strain ATCC 15692 / DSM 22644 / CIP 104116 / JCM 14847 / LMG 12228 / 1C / PRS 101 / PAO1) OX=208964 GN=pchR PE=2 SV=1

MTITIIAPPQADAAAPAPGNRPGVAHIDPNMKLVTGTFCSASEDWFEEPLERGLRLILVQSGQLRCRIPGQPEHLIEGPSLCTIANDGDFTSAQIYGTDKPLRYTIVQLGVEALDSRLGWLPEQLIRRPGGDPRIMSCPAPRAMQALASQIATCQMLGPTRDLYLGGKALELAALSAQFLSGEGRPVEEPRITCSEVERIHAARDLLVGALQEPPSLDTLASRVGMNPRKLTAGFRKVFGASVFGYLQEYRLREAHRMLCDEEANVSTVAYRVGYSPAHFSIAFRKRYGISPSEIR

>sp|Q06650|BLAC_STRCE Beta-lactamase OS=Streptomyces cellulosae OX=1968 GN=bla PE=3 SV=1

MRKPTSSLTRRSVLGAGLGLGGALALGSTTASAASAGTTPSENPAAVRRLRALEREHQARIGVFALNLATGASLLHRAHELFPMCSVFKTLAAAAVLRDLDHDGSQLARVIRYTEADVTKSGHAPVTKDHIDTGMTIRDLCDATIRYSDNCAANLLLRELGGPTAVTRFCRSLGDPVTRLDRWEPELNSGEPDRRTDTTSPYAIARTYQRLVLGNALNRPDRALLTDWLLRNTTTLTTFRTGLPKGWTVADKSGGGDTYGTRNEAAIAWTPDGAPVLLTALTHKPSLPTAPGDTPLIIKLATVLSEAVAPA

>sp|Q59517|BLAF_MYCFO Beta-lactamase OS=Mycobacterium fortuitum OX=1766 GN=blaF PE=1 SV=1

MTGLSRRNVLIGSLVAAAAVGAGVGGAAPAFAAPIDDQLAELERRDNVLIGLYAANLQSGRRITHRLDEMFAMCSTFKGYAAARVLQMAEHGEISLDNRVFVDADALVPNSPVTEARAGAEMTLAELCQAALQRSDNTAANLLLKTIGGPAAVTAFARSVGDERTRLDRWEVELNSAIPGDPRDTSTAAALAVGYRAILAGDALSPPQRGLLEDWMRANQTSSMRAGLPEGWTTADKTGSGDYGSTNDAGIAFGPDGQRLLLVMMTRSQAHDPKAENLRPLIGELTALVLPSLL

>sp|A9HXK3|UPPP_BORPD Undecaprenyl-diphosphatase OS=Bordetella petrii (strain ATCC BAA-461 / DSM 12804 / CCUG 43448) OX=340100 GN=uppP PE=3 SV=1

MTDSTLYLIKAFFLGIIEGLTEFIPVSSTGHLILIGDWINFTSSSGKVFEVVIQFGSILAVMWIFRARLWQLIRGTLTGVPAETAFTRNLLLAFLPAAVVGAIFIKTIKQVFYHPGVVAVTLVLGGLIMLWVERKTHHTPGDAPGAADDTASDERASAHTLEQISWKQALGVGVAQCLAMVPGTSRSGATIIGGMIAGIQRKTATEFSFFLAMPTMLGAATYDLYRNIDLLSQHDLSAIAVGFAAAFISALVVVRAVLRFVANHTYRGFAWYRIALGIVVAAWLMTK

>sp|Q184H7|UPPP2_PEPD6 Undecaprenyl-diphosphatase 2 OS=Peptoclostridium difficile (strain 630) OX=272563 GN=uppP2 PE=3 SV=1

MMSLDVIFILKSVIIAIVEGLTEFIPVSSTGHMILVGNLIDFKGQFAEMFEVVIQLGAILAVVVLYWKKIKDSVIEFFKFIFTGGKEGKIGFRFGMNVIIGCIPFAIIGVLFYDNIKSLFNLQSVIIGFIVGGILLLVVETLFRKKNHSTDNIDKITPIQALKVGTLQVLSAWPGMSRSASTIMGGWIAGLNSPTAAEFSFFLAVPAMVASSGKDLFEFDYSIMTPTLWIALVVGFIVAFIVSIIVMEKFVNFLKKKPMRVFAVYRIIMGVVLAVLAFTNIISV

>sp|Q13V13|UPPP1_PARXL Undecaprenyl-diphosphatase 1 OS=Paraburkholderia xenovorans (strain LB400) OX=266265 GN=uppP1 PE=3 SV=1

MDWLLACKALILGVVEGLTEFLPVSSTGHLIVAGSLLNFTDEHAKTFDVVIQLGAILAVCWEYRRRIGSVVSGLPSRPDARRFTLNVIIATIPAIVLGLLFEKTIKAALFSPVPVAFALVAGGVVILWAESRQRTRGETVARVQNVDDLGALDALKVGLAQCFALIPGMSRSGSTIIGGMLFGLDRRVATEFSFFLAIPIIFGATAYELHKDWHLLSVDALGTFALGFVAAFVSAFACVRWLLRYIAAHDFTAFAWYRIGFGLLILLVGYSGALNWTE

>sp|P43433|MYRB_MICGR Mycinamicin-resistance protein MyrB OS=Micromonospora griseorubida OX=28040 GN=myrB PE=3 SV=1

MSSIRRRHAAASLDTPAVGGRHELGQNFLVDRGVCTRIAEVVSSTTAHPVLELGAGDGAITRALVAANLPVTALELDPRRVRRLQRTFADGVTVVHGDMLRYDFGPYPHHVVSTVPFSITTPLLRRLIGQRFWHTAVLLVQWEVARKRAGVGGTTMLTAASWPWYEFTLVERVPKTSFDPVPSVDGGILVIERRSAPLLDDRCVGDYQNLVREVYTGPGRGLAAILRTRLPGREVDAWLRRERVDPAALPRDLKAGHWASLYRLYREVGTRPAPAGRSVRARPGSVGPDRSLPPRGLRSGPPRARRRGGGA

>sp|A4VLM0|UPPP1_PSEU5 Undecaprenyl-diphosphatase 1 OS=Pseudomonas stutzeri (strain A1501) OX=379731 GN=uppP1 PE=3 SV=1

MDLWVAIQALILGVVEGITEFLPVSSTGHQIIVADLIGFGGERALAFNIIIQLGAILAVIWEYRRKIIDVVVGLPEERQAQKFTVNLLIAFMPAVVLGVAFADLIHEYLFNPITVAAALVIGGIVMLWAERRDHAIRAETVDDMTWTLALKVGFAQCLALVPGTSRSGSTIIGGLLFGLSRKAATEFSFFLAMPTMVGAAVYSGYKYRDLFQPGDFAVFAIGFVTSFIFAMLAVRALLKFIGNHSYAAFAWYRIGFGLLILATWQLGMIDWSTAIG

>sp|Q4KC13|UPPP_PSEF5 Undecaprenyl-diphosphatase OS=Pseudomonas fluorescens (strain ATCC BAA-477 / NRRL B-23932 / Pf-5) OX=220664 GN=uppP PE=3 SV=1

MDLWTAAQALILGIVEGLTEFLPISSTGHQIIVADLLDFGGERAMAFNIIIQLGAILAVVWEFRRKILDVVIGLPTQPKAQRFTINLLIAFLPAVVLGVIFADLIHAYLFNPITVATALVVGGLIMLWAERRQHQVHAETVDDITWKDALKVGCAQCLAMIPGTSRSGSTIIGGLLFGLSRKTATEFSFFLAMPTMVGAAVYSGYKYRHLFQPDDFPVFAIGFVTAFVFAMIAVKGLLKFIASHSYAAFAWYRIAFGLLILATWQFGWVDWTAAKP

>sp|O07293|BLO18_PSEAI Beta-lactamase OXA-18 OS=Pseudomonas aeruginosa OX=287 GN=bla PE=1 SV=1

MQRSLSMSGKRHFIFAVSFVISTVCLTFSPANAAQKLSCTLVIDEASGDLLHREGSCDKAFAPMSTFKLPLAIMGYDADILLDATTPRWDYKPEFNGYKSQQKPTDPTIWLKDSIVWYSQELTRRLGESRFSDYVQRFDYGNKDVSGDPGKHNGLTHAWLASSLKISPEEQVRFLRRFLRGELPVSEDALEMTKAVVPHFEAGDWDVQGKTGTGSLSDAKGGKAPIGWFIGWATRDDRRVVFARLTVGARKGEQPAGPAARDEFLNTLPALSENF

>sp|Q5WCX5|UPPP2_BACSK Undecaprenyl-diphosphatase 2 OS=Bacillus clausii (strain KSM-K16) OX=66692 GN=uppP2 PE=3 SV=1

MDVWEWVVAAILGLVEGLTEYAPVSSTGHMIIVDDLWLKSSELVGSQNAYVFKIVIQLGSILAVALLFKDRLLQLAGFKKQAATQSEGRGLTLGKVAVGLLPAAVLGLLFEDKMESIFHVRTVAFALIAGAFLMIAADFINKRNNKKKQQVDDISYKQALAIGLFQCLALWPGFSRSGSTISGGVMLGLTHRAAANFTFIMAIPIMVGASALSLIKNWDALDISLLPFYATGFISAFLVSLVVVRFFLKLINKIKLVPFALYRIALGLLLLFLFS

>sp|P14489|BLO10_PSEAI Beta-lactamase OXA-10 OS=Pseudomonas aeruginosa OX=287 GN=bla PE=1 SV=1

MKTFAAYVIIACLSSTALAGSITENTSWNKEFSAEAVNGVFVLCKSSSKSCATNDLARASKEYLPASTFKIPNAIIGLETGVIKNEHQVFKWDGKPRAMKQWERDLTLRGAIQVSAVPVFQQIAREVGEVRMQKYLKKFSYGNQNISGGIDKFWLEGQLRISAVNQVEFLESLYLNKLSASKENQLIVKEALVTEAAPEYLVHSKTGFSGVGTESNPGVAWWVGWVEKETEVYFFAFNMDIDNESKLPLRKSIPTKIMESEGIIGG

>sp|P29808|AACC3_PSEAI Aminoglycoside N(3)-acetyltransferase III OS=Pseudomonas aeruginosa OX=287 GN=aacC3 PE=3 SV=1

MTDLNIPHTHAHLVDAFQALGIRAGQALMLHASVKAVGAVMGGPNVILQALMDALTPDGTLMMYAGWQDIPDFIDSLPDALKAVYLEQHPPFDPATARAVRENSVLAEFLRTWPCVHRSANPEASMVAVGRQAALLTANHALDYGYGVESPLAKLVAIEGYVLMLGAPLDTITLLHHAEYLAKMRHKNVVRYPCPILRDGRKVWVTVEDYDTGDPHDDYSFEQIARDYVAQGGGTRGKVGDADAYLFAAQDLTRFAVQWLESRFGDSASYG

>sp|A0KQI1|UPPP_AERHH Undecaprenyl-diphosphatase OS=Aeromonas hydrophila subsp. hydrophila (strain ATCC 7966 / DSM 30187 / JCM 1027 / KCTC 2358 / NCIMB 9240) OX=380703 GN=uppP PE=3 SV=1

MTESYALFVAFVLGIVEGLTEFLPVSSTGHMIIVGHLLGFDGPKAATFEVVIQMGSILAVVAVFWRRLFGLIGIHFGQKPAQGHATLSLVHIILGMLPAVIIGLAIHSWIKAHLFGPQTVMYALVAGGILLIIAEKFRPAVRSETLDDISYKQALGIGLFQCLALWPGFSRSGATISGGMLMGISRQAAAEFSFILAVPMMVAASGLDLYKSRDLLSMADFPMFAVGFITAFVVAMIAIKTFLALIRRLDFIPFAIYRFVVAFAVYLVFVA

>sp|Q01515|AAC3_SERMA Aminoglycoside N(3)-acetyltransferase III OS=Serratia marcescens OX=615 GN=aac3-Vb PE=3 SV=1

MNTIESITADLHGLGVRPGDLIMVHASLKAVGPVEGGAASVVSALRAAVGSAGTLMGYASWDRSPYEETLNGARMDEELRRRWPPFDLATSGTYPGFGLLNRFLLEAPDARRSAHPDASMVAVGPLAATLTEPHRLGQALGEGSPLERFVGHGGKVLLLGAPLDSVTVLHYAEAIAPIPNKRRVTYEMPMLGPDGRVRWELAEDFDSNGILDCFAVDGKPDAVETIAKAYVELGRHREGIVGRAPSYLFEAQDIVSFGVTYLEQHFGAP

>sp|A0R8Y3|UPPP1_BACAH Undecaprenyl-diphosphatase 1 OS=Bacillus thuringiensis (strain Al Hakam) OX=412694 GN=uppP1 PE=3 SV=1

MSDIIIAFILGIVEGLAEFLPISSTGHLILVGHLLGFEGERAKTFEIVIQLGAILAIAILYHKRLVSLCNIKPLLRKEKKFNAFHVFLGVFPAVVAGLLLHDVIKTYLFQPYTVVIGLVAGAILMIFAEVKKQEATSYSLDDLTYRQALTIGLFQCLAVYPGFSRAGSTISGGLLAKVNYKTASEFSFLIALPVMVGATGLDLLKSWTYLSVDDIPMFAVGFITSFIVAMLAVVTFLKLLEKIGLKPFAYYRILLAILFTVFVLL

>sp|P14508|KKA7_CAMJU Aminoglycoside 3'-phosphotransferase OS=Campylobacter jejuni OX=197 GN=aphA-7 PE=3 SV=1

MKYIDEIQILGKCSEGMSPAEVYKCQLKNTVCYLKKIDDIFSKTTYSVKREAEMMMWLSDKLKVPDVIEYGVREHSEYLIMSELRGKHIDCFIDHPIKYIECLVNALHQLQAIDIRNCPFSSKIDVRLKELKYLLDNRIADIDVSNWEDTTEFDDPMTLYQWLCENQPQEELCLSHGDMSANFFVSHDGIYFYDLARCGVADKWLDIAFCVREIREYYPDSDYEKFFFNMLGLEPDYKKINYYILLDEMF

>sp|P37414|YTL1_SALTY Uncharacterized protein pSLT049 OS=Salmonella typhimurium (strain LT2 / SGSC1412 / ATCC 700720) OX=99287 GN=PSLT049 PE=4 SV=1

MKPAPGAEPVRMYKSPYGGKYGVWRLADCVPMRAKRPQTEKQRLASTRLGLQARMKSERGRFAMLAHTWLALGPVFLDTETTGLDAGAQALEIGLVNARGERIFETRLKPTVGIDPAAAAVQIVTEPYRFPVLSVRLA

>sp|P00487|CAT_BACPU Chloramphenicol acetyltransferase OS=Bacillus pumilus OX=1408 GN=cat86 PE=2 SV=1

MFKQIDENYLRKEHFHHYMTLTRCSYSLVINLDITKLHAILKEKKLKVYPVQIYLLARAVQKIPEFRMDQVNDELGYWEILHPSYTILNKETKTFSSIWTPFDENFAQFYKSCVADIETFSKSSNLFPKPHMPENMFNISSLPWIDFTSFNLNVSTDEAYLLPIFTIGKFKVEEGKIILPVAIQVHHAVCDGYHAGQYVEYLRWLIEHCDEWLNDSLHIT

>sp|Q47744|VANR_ENTFA Regulatory protein VanRB OS=Enterococcus faecalis (strain ATCC 700802 / V583) OX=226185 GN=vanRB PE=3 SV=1

MSIRILLVEDDDHICNTVRAFLAEARYEVDACTDGNEAHTKFYENTYQLVILDIMLPGMNGHELLREFRAQNDTPILMMTALSDDENQIRAFDAEADDYVTKPFKMRILLKRVEALLRRSGALAKEFRVGRLTLLPEDFRVLCDGTELPLTRKEFEILLLLVQNKGRTLTHEIILSRIWGYDFDGDGSTVHTHIKNLRAKLPENIIKTIRGVGYRLEESL

>sp|A7Z3A4|FOSB_BACVZ Metallothiol transferase FosB OS=Bacillus velezensis (strain DSM 23117 / BGSC 10A6 / FZB42) OX=326423 GN=fosB PE=3 SV=1

MLNEVGKINIKGINHLLFSVSNLEKSIEFYEKVFHAQLLVKGQKTAYFDLNGLWLALNLEADIPRNEIHKSYTHMAFTIDPKDFDAIHHRLKNLNVNILNGRPRDKQDQKSIYFTDPDGHKFEFHTGTLQDRLSYYKKDKPHMKFYI

>sp|Q9KBZ6|FOSB_BACHD Metallothiol transferase FosB OS=Bacillus halodurans (strain ATCC BAA-125 / DSM 18197 / FERM 7344 / JCM 9153 / C-125) OX=272558 GN=fosB PE=3 SV=1

MRIQGINHLLFSVKCLERSIEFYKKALGAKLLVKGRTTAYFDLQGIWLALNEEPDIPRNEIHQSYTHIAFTVGEEEMEEAYERLAGLGVNILKGRPRDPRDRQSIYFTDPDGHKFEFHCGTLNDRLDYYREAKPHMTFFDD

>sp|A4IS40|FOSB_GEOTN Metallothiol transferase FosB OS=Geobacillus thermodenitrificans (strain NG80-2) OX=420246 GN=fosB PE=3 SV=1

MRIGGINHLTFSVSDLEKSIHFYQNVFGAKLLVKGRNLAYFDLNGIWLALNVQQDIPRNDIQHSYTHIAFSVKEEDFDHVVEKLKELGVNILPGRERDERDKRSVYFTDPDGHKFEFHTGTLNDRLSYYKSEMHHMQFFD

>sp|P72533|TETO_STREE Tetracycline resistance protein TetO OS=Streptococcus pneumoniae OX=1313 GN=tetO PE=3 SV=1

MKIINLGILAHVDAGKTTLTESLLYTSGAIAEPGSVDKGTTRTDTMNLERQRGITIQTAVTSFQWEDVKVNIIDTPGHMDFLAEVYRSLSVLDGAVLLVSAKDGIQAQTRILFHALQTMKIPTIFFINKIDQEGIDLPMVYQEMKAKLSSEIIVKQKVGQHPHINVTDNDDMEQWDAVIMGNDELLEKYMSGKPFKMSELEQEENRRFQNGTLFPVYHGSAKNNLGIRQLIEVIASKFYSSTPEGQSELCGQVFKIEYSEKRRRFVYVRIYSGTLHLRDVIKISEKEKIKITEMCVPTNGELYSSDTACSGDIVILPNDVLQLNSILGNEMLLPQRKFIENPLPMLQTTIAVKKSEQREILLGALTEISDGDPLLKYYVDTTTHEIILSFLGNVQMEVICAILEEKYHVEAEIKEPTVIYMERPLRKAEYTIHIEVPPNPFWASVGLSIEPLPIGSGVQYESRVSLGYLNQSFQNAVMEGVLYGCEQGLYGWKVTDCKICFEYGLYYSPVSTPADFRLLSPIVLEQALKKAGTELLEPYLHFEIYAPQEYLSRAYHDAPRYCADIVSTQVKNDEVILKGEIPARCIQEYRNDLTYFTNGQGVCLTELKGYQPAIGKFICQPRRPNSRIDKVRHMFHKLA

>tr|D2JGC2|D2JGC2_STAAU Lincosamide nucleotidyltransferase OS=Staphylococcus aureus OX=1280 GN=lnu(A) PE=4 SV=1

MKNNNVTEKDLFYILDLFEHMKVTYWLDGGWGVDVLTGKQQREHRDIDIDFDAQHTQKVIQKLEDIGYKIEVDWMPSRMELKHEEYGYLDIHPINLNDDGSITQANPEGGNYVFQNDWFSETNYKGRKIPCISKEAQLLFHSGYDLTEKDHFDIKNLKSIT

>sp|A0A0D2YG02|FUB7_FUSO4 Sulfhydrylase FUB7 OS=Fusarium oxysporum f. sp. lycopersici (strain 4287 / CBS 123668 / FGSC 9935 / NRRL 34936) OX=426428 GN=FUB7 PE=1 SV=1

MAEQVFQNFETLQLHAGYTPDPHTRSTAVPIYATSSYTFNDSAHGARLFGLKELGNIYSRLMNPTVDVFEKRIAALEGGIAAAATSSGQAAQFLTIATLAKAGDNIVASSHLYGGTYNQLNVLLPRFGIKTKFVRSGKLEDYAAAIDDQTRAIYVESMSNPDYVVPDFEGIAKIAHEHGIPLVVDNTLGAGGYYIRPIEHGADIVVHSATKWIGGHGTTIGGVIVDSGRFNWNKHSERFPEMVEPSPSYHGLKYWEAFGPATFITRIRVEMLRDIGACLSPFSAQQLLLGIETLGLRAERHAQNTEKLAKYFESSPNVSWVLWPGSESHPTYAQAKKYLTRGFGAMLSIGVKGDASAGSKVVDGLKLVSNLANVGDAKSLAIHPWSTTHEQLSEDERLASGVTEDMIRISVGIEHVDDIIADFEQSFQKAYGS

>sp|A0R066|ILVE_MYCS2 Branched-chain-amino-acid aminotransferase OS=Mycobacterium smegmatis (strain ATCC 700084 / mc(2)155) OX=246196 GN=ilvE PE=1 SV=1

MNSGPLEFTVSANTNPATDAVRESILANPGFGKYYTDHMVSIDYTVDEGWHNAQVIPYGPIQLDPSAIVLHYGQEIFEGLKAYRWADGSIVSFRPEANAARLQSSARRLAIPELPEEVFIESLRQLIAVDEKWVPPAGGEESLYLRPFVIATEPGLGVRPSNEYRYLLIASPAGAYFKGGIKPVSVWLSHEYVRASPGGTGAAKFGGNYAASLLAQAQAAEMGCDQVVWLDAIERRYVEEMGGMNLFFVFGSGGSARLVTPELSGSLLPGITRDSLLQLATDAGFAVEERKIDVDEWQKKAGAGEITEVFACGTAAVITPVSHVKHHDGEFTIADGQPGEITMALRDTLTGIQRGTFADTHGWMARLN

>sp|A0AI83|CODY_LISW6 GTP-sensing transcriptional pleiotropic repressor CodY OS=Listeria welshimeri serovar 6b (strain ATCC 35897 / DSM 20650 / SLCC5334) OX=386043 GN=codY PE=3 SV=1

MTLLEKTRKINAMLQNAAGKTVNFKEMADTLTDVIEANTYIVSRKGKLLGYSESLPIENDRMKQMLTERQFPEEYTQSLFNVGETSSNLEVSSQYTAFPIENSDLFTKGLTTIVPIVGGGERLGTLILSRLESNFTDDDLLLAEYGGTVVGMEILHEKAEEIEEEARSRAVVQMAISSLSYSELEAIEHIFDELNGKEGLLVASKIADRVGITRSVIVNALRKLESAGVIDSRSLGMKGTFIRVLNDKFLVELEKLKNN

>sp|A0AJY3|MNTR_LISW6 HTH-type transcriptional regulator MntR OS=Listeria welshimeri serovar 6b (strain ATCC 35897 / DSM 20650 / SLCC5334) OX=386043 GN=mntR PE=3 SV=1

MPTPSMEDYIEKIYSLIETKGYARVSDIADELFVHPSSVTKMVQKLDKDEYLIYEKYRGLILTPKGTQMGKRLLERHALLESFLSIIGVDSSHIYHDVEGIEHHLSWNSIDRIGNVVQFFENHPDALEALKAMETTKPETNE

>sp|A0LLH2|YIDD_SYNFM Putative membrane protein insertion efficiency factor OS=Syntrophobacter fumaroxidans (strain DSM 10017 / MPOB) OX=335543 GN=Sfum_2596 PE=3 SV=1

MIRSIFLGLIRFYQIVLSPLKGPRCRFLPTCSQYAYEAIERYGIWRGLFLGGKRLLRCHPFHAGGYDPVPRPSANNHPSR

>sp|O06474|YFMP_BACSU HTH-type transcriptional regulator YfmP OS=Bacillus subtilis (strain 168) OX=224308 GN=yfmP PE=2 SV=1

MEWMKIDQVAKRSGLTKRTIRFYEEIGLIPAPKRTDGGVRLYSEDDMEELEKVISTKEVLGFSLQELQHFMETSRQLELNKEGYLLSLDPKERKEKLEEIQETLNHQLDLIDEKIRTFQSFKERLQGMKGKAERAIQSIE

>sp|B1Q2A8|NPHR_RHOSO Transcriptional activator NphR OS=Rhodococcus sp. OX=1831 GN=nphR PE=1 SV=1

MAEREQSNDSARTDVPAIVSLRTRELDTGEGRMQWASTLERLYCETDVAWPEPRRHFDAEWGGRPFGDLHVSTIRADAHTVVRSPAMIQSDSGEGYLVCLVTDGSVEVRQSGRATVVEPGSFALLDCAAPFVFHSPAPFRQVVVRSPREVLTSRLPGRIVEHGTARSIHGDTGAGGLVGRLFVDIADMDAPMSQGAAVSFASSAVDMLATALTEGLLATSAADLHRTEDLTRVQRVIEQNLHDADITLSDIAAAAGMSLRTVHKLFNAEGTTTRAWLYQARLEAARRYLLTTDLSVADVSECAGFRDVSHFSRLFRSTFGSSPGLYRKEHARIGS

>sp|P0A3M3|BLO9_KLEPN Beta-lactamase OXA-9 OS=Klebsiella pneumoniae OX=573 GN=bla PE=3 SV=2

MKKILLLHMLVFVSATLPISSVASDEVETLKCTIIADAITGNTLYETGECARRVSPCSSFKLPLAIMGFDSGILQSPKSPTWELKPEYNPSPRDRTYKQVYPALWQSDSVVWFSQQLTSRLGVDRFTEYVKKFEYGNQDVSGDSGKHNGLTQSWLMSSLTISPKEQIQFLLRFVAHKLPVSEAAYDMAYATIPQYQAAEGWAVHGKSGSGWLRDNNGKINESRPQGWFVGWAEKNGRQVVFARLEIGKEKSDIPGGSKAREDILVELPVLMGNK

>sp|A0A0H3MDW1|CHXR_CHLT2 Atypical response regulator protein ChxR OS=Chlamydia trachomatis serovar L2 (strain 434/Bu / ATCC VR-902B) OX=471472 GN=chxR PE=1 SV=1

MAGPKHVLLVSEHWDLFFQTKELLNPEEYRCTIGQQYKQELSADLVVCEYSLLPREIRSPKSLEGSFVLVLLDFFDEETSVDLLDRGFWYLIRPITPRILKSAISLFLSQHSLHSVPESIRFGPNVFYVLKLTVETPEGSVHLTPSESGILKRLLINKGQLCLRKHLLEEIKNHAKAIVARNVDVHIASLRKKLGAYGSRIVTLRGVGYLFSDDGDKKFSQQDTKLS

>sp|A0PWB3|MPRB_MYCUA Signal transduction histidine-protein kinase/phosphatase MprB OS=Mycobacterium ulcerans (strain Agy99) OX=362242 GN=mprB PE=3 SV=1

MVGFRRGPRAPLRATSSLSLRWRVMLLAMSMVAMVVVLMSFAVYAVISAALYSDIDNQLQSRAQLLIASGSLAADPGKAIEGTAYSDVNAMLVNPGRSIYTANQPGQTLPVGAPEKAVIHGDLFLSRRTVSDQRVLAIHLPNGSSLLISKSLKPTEAVMTKLRAVLLIVGGVGVAVAAVAGGMVTRAGLRPVGRLTEAAERVARTDDLRPIPVFGSDELARLTEAFNLMLRALAESRERQARLVSDAGHELRTPLTSLRTNVELLMASMEPGAPRLPEQEMVGLREDVVAQIEELSTLVGDLVDLTRGDAGVVVHEPVDMAEVVDRSLERVRRRRNDIHFDVDVVGWQVYGDAAGLSRAALNLMDNAAKWSPPGGRVGIRLRQLDPSHAELVVSDNGPGISPQERRLVFERFYRSTSARAMPGSGLGLAIVKQVVLNHGGSLRIEDTVPGGQPPGTAICMLLPGRPMPDSAYPAAPDDKKTEPVDTRGANGANSRGSANVISVDSQSARAR

>sp|A0A067XMV2|PTAH_PESFW Methyltransferase ptaH OS=Pestalotiopsis fici (strain W106-1 / CGMCC3.15140) OX=1229662 GN=ptaH PE=2 SV=1

MSTNDEVFAKDNEFWKTYLRGRAQPPESFFERIFRYHEDHGGHFGTVHDCGAGNGPYSQKLRSRFKHVIVSDVAPGNVELAKERLGNDGFSFRVARVEDFDDIPTGSVDLVFATNVMHWVEPSRGAKAIVSQLKSGGTFIAAGFGPARFEDQKVQDIWTRISQSGGRRLIMKADDPTKILKVAVRSSRYYDVAPTDTSLFVPGTQRIHLNMNNGGLTDIVYPEDYVAAAEPSYTGPQDDEIFESEDGWSFETDLEGVKDHFATFPFSKEDPEVFAELWAELEKYVADGRPIRGCWPAKIILATRV

>sp|O66565|Y178_AQUAE Universal stress protein Aq_178 OS=Aquifex aeolicus (strain VF5) OX=224324 GN=aq_178 PE=1 SV=1

MKVLLVLTDAYSDCEKAITYAVNFSEKLGAELDILAVLEDVYNLERANVTFGLPFPPEIKEESKKRIERRLREVWEKLTGSTEIPGVEYRIGPLSEEVKKFVEGKGYELVVWACYPSAYLCKVIDGLNLASLIVK

>sp|A0JZC7|TYSY_ARTS2 Thymidylate synthase OS=Arthrobacter sp. (strain FB24) OX=290399 GN=thyA PE=3 SV=1

MSIPTPYEDLLRDVLANGTHKSDRTGTGTLSVFGRQMRFDLSQSFPLITTKRVHFKSVAVELLWFLRGETNVKWMQDQGVTIWNEWADADGELGPVYGVQWRSWPTPDGGHIDQIAELVENLKSNPDSRRHIVSAWNVAELQDMALPPCHAFFQFYVADGKLSCQLYQRSADTFLGVPFNIASYALLTCMLAQQVGLEPGEFVWTGGDVHIYDNHMDQVLKQLKREPYEYPQLKILRKPDSIFDYTLDDFEVVGYQHHPTIKAPIAV

>sp|A0AJY0|TYSY_LISW6 Thymidylate synthase OS=Listeria welshimeri serovar 6b (strain ATCC 35897 / DSM 20650 / SLCC5334) OX=386043 GN=thyA PE=3 SV=1

MKQYLDLEKYVLENGTQKGDRTGTGTISTFGYQMRFDLQEGFPIMTTKRVPFKLVVSELLWFLHGDTNIRYLLQHNNNIWNEWAFERFVKSADYKGEDMTDFGLRAERDPAFKEVYQAEMEQFKTRILEDEGFANKYGELGNIYGKQWREWKTSQGETIDQLADVIEMIKTNPNSRRLIVSAWNPEDIPNMALPPCHSLFQFYVADGKLSCQLYQRSADIFLGVPFNIASYALLTHLIAREVGLDVGEFIHTMGDAHLYNNHIEQVKEQLSRTPHALPKLVLSDKPTTIFDFDVADISLDGYHPDPAIKAPISV

>sp|A0AHA7|RF3_LISW6 Peptide chain release factor 3 OS=Listeria welshimeri serovar 6b (strain ATCC 35897 / DSM 20650 / SLCC5334) OX=386043 GN=prfC PE=3 SV=1

MSQDLQKEVASRKTFAIISHPDAGKTTITEQLLLFGGVIRSAGTVKGKKSGKFATSDWMEIEKQRGISVTSSVMQFDYNGSRINILDTPGHSDFSEDTYRTLMAVDSAVMVIDAAKGIEAQTLKLFKVCRMRGIPIFTFINKMDRQGKMPLELLAELEEVLEIESYPMNWPIGMGKELAGLYDRYHRVIEQYRSEEDERFLPLGEDGDLKEAHEIQKSLYYDQALEEIMLLDEAGNDFSRERILAGEQTPVFFGSALTNFGVETFLRTFVDFAPAPSSHESNEGIIEADNPKFSGFIFKIQANMNPAHRDRIAFIRICSGEFERGMNVTLTRTGKSIKLANSTQFMADDRETVNRAVAGDIIGLYDTGNYQIGDTITNGSKKLEFEKLPQFTPELFMRVYAKNVMKQKHFHKGVEQLVQEGAIQLFKTWRTEEYIIGAVGQLQFEVFEHRMRGEYNSEIRMEPIGKKIARWVKEEDADEKLSTARSMLVKDRFDQPLFLFENEFAINWFNDKNPDIELTSLL

>sp|A0ALY8|EFTU_LISW6 Elongation factor Tu OS=Listeria welshimeri serovar 6b (strain ATCC 35897 / DSM 20650 / SLCC5334) OX=386043 GN=tuf PE=3 SV=1

MAKEKFDRSKPHVNIGTIGHVDHGKTTLTAAITTVLAKKGYADAQAYDQIDGAPEERERGITISTAHVEYQTDSRHYAHVDCPGHADYVKNMITGAAQMDGAILVVSAADGPMPQTREHILLSRQVGVPYIVVFMNKCDMVDDEELLELVEMEIRDLLTEYEFPGDDIPVIKGSALKALQGEADWEAKIDELMEAVDSYIPTPERDTDKPFMMPVEDVFSITGRGTVATGRVERGQVKVGDEVEVIGIEEESKKVVVTGVEMFRKLLDYAEAGDNIGALLRGVAREDIQRGQVLAKPGSITPHTNFKAETYVLTKEEGGRHTPFFNNYRPQFYFRTTDVTGIVTLPEGTEMVMPGDNIELAVELIAPIAIEDGTKFSIREGGRTVGAGVVSNISK

>sp|A4JGI0|RLMH_BURVG Ribosomal RNA large subunit methyltransferase H OS=Burkholderia vietnamiensis (strain G4 / LMG 22486) OX=269482 GN=rlmH PE=3 SV=1

MKLYILAVGHKMPGWIASGFDEYTKRMPPELRIELREIKPELRSGGRSAESVMAAERQKIEAALPKGARIVALDERGRDWTTMQLAQALPGWQQDGRDVAFVIGGADGLDPELKARADLLLRISSMTLPHGMVRVLLAEQLYRAWSITQNHPYHRA

>sp|B2IHG2|KHSE_BEII9 Homoserine kinase OS=Beijerinckia indica subsp. indica (strain ATCC 9039 / DSM 1715 / NCIB 8712) OX=395963 GN=thrB PE=3 SV=1

MAVYTHISETDLKTFLASYDIGNALVLKGIAEGVENSNFFLQTERGFFILTLYEKRVEEKDLPFFLGLMEHLSRRGLNCPQPVHNRSGHALGRLAGRPAVIVTFLEGVGADVADARRCAAVGEALARLHQAGADFAGKRTNALGLAAWRPLFETVRDRADTVAPALAATIAEELDFLEAHWPRALPQGVIHADLFPDNVLFRGETLSGLIDFYFACVDAYAYDIAICLNAWCFEPDLTFNIGKGLAFFTGYEHVRKLTAEEAAALPVLARGGALRFALTRLVDWLNVPKGAMVNPKDPLEYMGKLAFHQTVGTVRELGLLR

>sp|P0A5I5|KGUA_MYCBO Guanylate kinase OS=Mycobacterium bovis (strain ATCC BAA-935 / AF2122/97) OX=233413 GN=gmk PE=3 SV=1

MSVGEGPDTKPTARGQPAAVGRVVVLSGPSAVGKSTVVRCLRERIPNLHFSVSATTRAPRPGEVDGVDYHFIDPTRFQQLIDQGELLEWAEIHGGLHRSGTLAQPVRAAAATGVPVLIEVDLAGARAIKKTMPEAVTVFLAPPSWQDLQARLIGRGTETADVIQRRLDTARIELAAQGDFDKVVVNRRLESACAELVSLLVGTAPGSP

>sp|A1E9Q2|RR12_SORBI 30S ribosomal protein S12, chloroplastic OS=Sorghum bicolor OX=4558 GN=rps12-A PE=3 SV=1

MPTVKQLIRNARQPIRNARKSAALKGCPQRRGTCARVYTINPKKPNSALRKVARVRLTSGFEITAYIPGIGHNLQEHSVVLVRGGRVKDLPGVRYRIIRGTLDAVAVKNRQQGRSKYGAKKPKK

>sp|B3DMA2|ACD11_RAT Acyl-CoA dehydrogenase family member 11 OS=Rattus norvegicus OX=10116 GN=Acad11 PE=1 SV=1

MEMDVTRDTVEVLPQHKFDIRSLEAYLNQHLPGFGSDHRAVLTVTQYRSGQSNPTFFLQKGSQAYVLRKKPPGSLLPKAHKIDREFKVQKALFSVGFPVPKPLLYCSNASIIGTEFYVMEHVQGRIFRDFSIPGVSPAERAAIYVSLVETLAWLHSLDIHSLGLDRYGTGVGYCKRQVSTWTKQYQASAHQSIPAMDQLSTWLMRNLPDSDNEECLVHGDFKLDNIVFHPKECRVIAVLDWELSTFGHPLSDLAHLSLFYFWPRTLPMINRGSHIQENTGIPLMEELISIYCRRRGIDPNLPNWNFFMALSFFKLAGIAQGVYSRYLMGNNSSEDSFLTANTVQPLAETGLQLSRRTLSTVPPQADAKSRLFAQSRRGQEVLTRVKQFMKQHVFPAEKEVAEYYAQNGNSAEKWEHPLVIEKLKEMAKAEGLWNLFLPAVSGLSQVDYALIAEETGKCFFAPDVFNCQAPDTGNMEVLHLYGSEQQKQQWLEPLLRGDITSVFCMTEPNVSSSDATNMECSIQRDGGSYIVHGKKWWSSGAGNPKCKIAVVLGRTESPSVSRHKVHSMILVPMDTPGVELIRPLSVFGYMDNVHGGHWEVHFNHVRVPASNLILGEGRGFEISQGRLGPGRIHHCMRSVGLAERILQIMCDRAVQREAFGKKLYEHEVVAHWIAKSRIAIEEIRLLTLKAAHSIDTLGSAAARKEIAMIKVAAPKAVCKIADRAIQVHGGAGVSQDYPLANMYAIIRTLRLADGPDEVHLSAIAKMELQDQARQLKARM

1. **Non-efflux prokaryotic proteins (Non-efflux): 554 Protein Sequences**

>sp|P0C0R7|RLME_ECOLI Ribosomal RNA large subunit methyltransferase E OS=Escherichia coli (strain K12) OX=83333 GN=rlmE PE=1 SV=1

MTGKKRSASSSRWLQEHFSDKYVQQAQKKGLRSRAWFKLDEIQQSDKLFKPGMTVVDLGAAPGGWSQYVVTQIGGKGRIIACDLLPMDPIVGVDFLQGDFRDELVMKALLERVGDSKVQVVMSDMAPNMSGTPAVDIPRAMYLVELALEMCRDVLAPGGSFVVKVFQGEGFDEYLREIRSLFTKVKVRKPDSSRARSREVYIVATGRKP

>sp|P61068|RL4_RHOPA 50S ribosomal protein L4 OS=Rhodopseudomonas palustris (strain ATCC BAA-98 / CGA009) OX=258594 GN=rplD PE=1 SV=1

MELKVTTLEGKEAGSVQLSDEIFGLEPRSDIIQRCVIWQLAKRQAGTHKAKGRAEVWRTGKKMYKQKGTGGARHGSQRVPQFRGGGRAFGPVVRSHAIDLPKKVRVLALRHALSAKAKGGGLIVLDKAELEAAKTKTLVGHFSGLGLESALIIDGAEVNNGFAAAARNIPNIDVLPVQGINVYDILRRKKLVLTKAAVDALEARFK

>sp|B0B7N2|RL7_CHLT2 50S ribosomal protein L7/L12 OS=Chlamydia trachomatis serovar L2 (strain 434/Bu / ATCC VR-902B) OX=471472 GN=rplL PE=1 SV=1

MTTESLETLVEQLSGLTVLELSQLKKLLEEKWDVTAAAPVVAVAGAAAAGDAPASAEPTEFAVILEDVPSDKKIGVLKVVREVTGLALKEAKEMTEGLPKTVKEKTSKSDAEDTVKKLQEAGAKAVAKGL

>sp|P60456|RL3_RHOPA 50S ribosomal protein L3 OS=Rhodopseudomonas palustris (strain ATCC BAA-98 / CGA009) OX=258594 GN=rplC PE=1 SV=1

MRSGVIAQKVGMTRVFTEAGEHIPVTVLKLGNCQVLGHRTKEKNGYVALQVGSGSRKTVYMPKAERGQFAAAKVEPKRKVEEFRVSEDALLPVGAEIQADHFVVGQFVDVTGTSTGKGFAGGMKRWNFGGLRATHGVSVSHRSIGSTGGRQDPGKTFKNKKMPGHMGVDRVTTLNLRVVQTDVERGLILVEGAVPGTKGGWIRVRDAVKKALPADAPKPGKFRLANGDAAAEAPAAEQEGA

>sp|P9WN65|RMLB_MYCTU dTDP-glucose 4,6-dehydratase OS=Mycobacterium tuberculosis (strain ATCC 25618 / H37Rv) OX=83332 GN=rmlB PE=1 SV=1

MRLLVTGGAGFIGTNFVHSAVREHPDDAVTVLDALTYAGRRESLADVEDAIRLVQGDITDAELVSQLVAESDAVVHFAAESHVDNALDNPEPFLHTNVIGTFTILEAVRRHGVRLHHISTDEVYGDLELDDRARFTESTPYNPSSPYSATKAGADMLVRAWVRSYGVRATISNCSNNYGPYQHVEKFIPRQITNVLTGRRPKLYGAGANVRDWIHVDDHNSAVRRILDRGRIGRTYLISSEGERDNLTVLRTLLRLMDRDPDDFDHVTDRVGHDLRYAIDPSTLYDELCWAPKHTDFEEGLRTTIDWYRDNESWWRPLKDATEARYQERGQ

>sp|Q07465|RNI_AERHY Ribonuclease OS=Aeromonas hydrophila OX=644 PE=1 SV=1

MKKIVVLLGMLLAPWFSSAVQAKGEAGEFDYYAMALSWSPEHCAIKPADRDQCSRQLGFVLHGLWPQYQRGYPSSCTRERLDPAMEQEFAGLYPSRFLYRHEWEKHGTCSGLSQHDFHQLASDLRQKREDPGRLSVSCRAAAQKPLPAQGGSGQCQRLAGPGQHHGGLRRRWRFLREVYICLNKEGTDAVTCSDEMQKRELPSCGQPDFLLRTVR

>sp|P32684|RLUF_ECOLI 23S rRNA pseudouridine(2604) synthase OS=Escherichia coli (strain K12) OX=83333 GN=rluF PE=1 SV=1

MLPDSSVRLNKYISESGICSRREADRYIEQGNVFLNGKRATIGDQVKPGDVVKVNGQLIEPREAEDLVLIALNKPVGIVSTTEDGERDNIVDFVNHSKRVFPIGRLDKDSQGLIFLTNHGDLVNKILRAGNDHEKEYLVTVDKPITEEFIRGMSAGVPILGTVTKKCKVKKEAPFVFRITLVQGLNRQIRRMCEHFGYEVKKLERTRIMNVSLSGIPLGEWRDLTDDELIDLFKLIENSSSEVKPKAKAKPKTAGIKRPVVKMEKTAEKGGRPASNGKRFTSPGRKKKGR

>sp|A0R1W8|RNPH_MYCS2 Ribonuclease PH OS=Mycobacterium smegmatis (strain ATCC 700084 / mc(2)155) OX=246196 GN=rph PE=1 SV=1

MSRREDGRLDDELRPVVITRGFTSHPAGSVLVEFGQTRVMCTASVTEGVPRWRKGSGQGWLTAEYAMLPAATHDRSDRESVKGRIGGRTQEISRLIGRSLRACIDLAALGENTIAIDCDVLQADGGTRTAAITGAYVALSDAVTWLAAAGRLSDPRPLSCAIAAVSVGVVDGRVRVDLPYSEDSRAEVDMNVVATDTGTLVEIQGTGEGATFPRSTLDKMLDAALGATEQLFVLQREALDAPYPGVLPEGPAPKKAFGS

>sp|P17622|RIBT_BACSU Protein RibT OS=Bacillus subtilis (strain 168) OX=224308 GN=ribT PE=1 SV=1

MLIRYKKSFEKIAMGLLSFMPNEKDLKQLQQTIKDYETDTDRQLFLWKEDEDIVGAIGVEKKDSEVEIRHISVNPSHRHQGIGKQMMDALKHLFKTQVLVPNELTQSFFERCQGQQDQDISYNN

>sp|P9WH95|RL332_MYCTU 50S ribosomal protein L33 2 OS=Mycobacterium tuberculosis (strain ATCC 25618 / H37Rv) OX=83332 GN=rpmG2 PE=1 SV=1

MASSTDVRPKITLACEVCKHRNYITKKNRRNDPDRLELKKFCPNCGKHQAHRETR

>sp|P03051|ROP_ECOLX Regulatory protein rop OS=Escherichia coli OX=562 GN=rop PE=1 SV=1

MTKQEKTALNMARFIRSQTLTLLEKLNELDADEQADICESLHDHADELYRSCLARFGDDGENL

>sp|Q6G3V6|RPIA_BARHE Ribose-5-phosphate isomerase A OS=Bartonella henselae (strain ATCC 49882 / DSM 28221 / Houston 1) OX=283166 GN=rpiA PE=1 SV=1

MNVQQLKKMAALKALEFVEDDMRLGIGSGSTVNEFIPLLGERVANGLRVTCVATSQYSEQLCHKFGVPISTLEKIPELDLDIDGADEIGPEMTLIKGGGGALLHEKIVASASRAMFVIADETKMVKTLGAFALPIEVNPFGIHATRIAIEKAADNLGLSGEITLRMNGDDPFKTDGGHFIFDAFWGRILQPKLLSEALLAIPGVVEHGLFLGLASRAIVAMADSQIKVLEPFDF

>sp|Q5SII2|RSMF_THET8 Ribosomal RNA small subunit methyltransferase F OS=Thermus thermophilus (strain HB8 / ATCC 27634 / DSM 579) OX=300852 GN=rsmF PE=1 SV=1

MLPKAFLSRMAELLGEEFPAFLKALTEGKRTYGLRVNTLKLPPEAFQRISPWPLRPIPWCQEGFYYPEEARPGPHPFFYAGLYYIQEPSAQAVGVLLDPKPGERVLDLAAAPGGKTTHLAARMGGKGLLLANEVDGKRVRGLLENVERWGAPLAVTQAPPRALAEAFGTYFHRVLLDAPCSGEGMFRKDREAARHWGPSAPKRMAEVQKALLAQASRLLGPGGVLVYSTCTFAPEENEGVVAHFLKAHPEFRLEDARLHPLFAPGVPEWGEGNPELLKTARLWPHRLEGEGHFLARFRKEGGAWSTPRLERPSPLSQEALRAFRGFLEEAGLTLEGPVLDRAGHLYLLPEGLPTLLGLKAPAPGLYLGKVQKGRFLPARALALAFGATLPWPEGLPRLALTPEDPRALAFATGEGVAWEGEDHPLALVVLKTAAGEFPLDFGKAKRGVLRPVGVGL

>sp|Q8ZKB0|RSGA_SALTY Small ribosomal subunit biogenesis GTPase RsgA OS=Salmonella typhimurium (strain LT2 / SGSC1412 / ATCC 700720) OX=99287 GN=rsgA PE=1 SV=2

MSKNKLSKGQQRRVNANHQRRLKTSAEKADYDDNLFGEPAEGIVISRFGMHADVESADGEVHRCNIRRTIRSLVTGDRVVWRPGKAAAEGVNVKGIVEAVHERTSVLTRPDFYDGVKPIAANIDQIVIVSAILPELSLNIIDRYLVGCETLQVEPLIVLNKIDLLDDEGMDFVNEQMDIYRNIGYRVLMVSSHTQDGLKPLEEALTGRISIFAGQSGVGKSSLLNALLGLQNEILTNDVSNVSGLGQHTTTAARLYHFPHGGDVIDSPGVREFGLWHLEPEQITQGFVEFHDYLGHCKYRDCKHDADPGCAIREAVENGAIAETRFENYHRILESMAQVKTRKNFSDTDD

>sp|P26420|SCRK_KLEPN Fructokinase OS=Klebsiella pneumoniae OX=573 GN=scrK PE=1 SV=1

MNGKIWVLGDAVVDLLPDGEGRLLQCPGGAPANVAVGVARLGGDSGFIGRVGDDPFGRFMRHTLAQEQVDVNYMRLDAAQRTSTVVVDLDSHGERTFTFMVRPSADLFLQPEDLPPFAAGQWLHVCSIALSAEPSRSTTFAALEAIKRAGGYVSFDPNIRSDLWQDPQDLRDCLDRALALADAIKLSEEELAFISGSDDIVSGIARLNARFQPTLLLVTQGKAGVQAALRGQVSHFPARPVVAVDTTGAGDAFVAGLLAGLAAHGIPDNLAALAPDLALAQTCGALATTAKGAMTALPYKDDLQRSL

>sp|A5W4E3|TODS_PSEP1 Sensor histidine kinase TodS OS=Pseudomonas putida (strain ATCC 700007 / DSM 6899 / BCRC 17059 / F1) OX=351746 GN=todS PE=1 SV=1

MSSLDRKKPQNRSKNNYYNICLKEKGSEELTCEEHARIIFDGLYEFVGLLDAHGNVLEVNQVALEGGGITLEEIRGKPFWKARWWQISKKTEATQKRLVETASSGEFVRCDVEILGKSGGREVIAVDFSLLPICNEEGSIVYLLAEGRNITDKKKAEAMLALKNQELEQSVECIRKLDNAKSDFFAKVSHELRTPLSLILGPLEAVMAAEAGRESPYWKQFEVIQRNAMTLLKQVNTLLDLAKMDARQMGLSYRRANLSQLTRTISSNFEGIAQQKSITFDTKLPVQMVAEVDCEKYERIILNLLSNAFKFTPDGGLIRCCLSLSRPNYALVTVSDSGPGIPPALRKEIFERFHQLSQEGQQATRGTGLGLSIVKEFVELHRGTISVSDAPGGGALFQVKLPLNAPEGAYVASNTAPRRDNPQVVDTDEYLLLAPNAENEAEVLPFQSDQPRVLIVEDNPDMRGFIKDCLSSDYQVYVAPDGAKALELMSNMPPDLLITDLIMPVMSGDMLVHQVRKKNELSHIPIMVLSAKSDAELRVKLLSESVQDFLLKPFSAHELRARVSNLVSMKVAGDALRKELSDQGDDIAILTHRLIKSRHRLQQSNIALSASEARWKAVYENSAAGIVLTDPENRILNANPAFQRITGYGEKDLEGLSMEQLTPSDESPQIKQRLANLLQGGGAEYSVERSYLCKNGSTIWANASVSLMPQRVGESPVILQIIDDITEKKQAQENLNQLQQQLVYVSRSATMGEFAAYIAHEINQPLSAIMTNANAGTRWLGNEPSNIPEAKEALARIIRDSDRAAEIIRMVRSFLKRQETVLKPIDLKALVTDTSLILKAPSQNNSVNLDVVADDELPEIWGDGVQIQQLIINLAMNAIEAISQADCETRQLTLSFSGNDTGDALVISVKDTGPGISERQMAQLFNAFYTTKKEGLGMGLAICLTITEVHNGKIWVECPPAGGACFLVSIPARQGSGT

>sp|P9WFY9|TRMB_MYCTU tRNA (guanine-N(7)-)-methyltransferase OS=Mycobacterium tuberculosis (strain ATCC 25618 / H37Rv) OX=83332 GN=trmB PE=1 SV=1

MVHHGQMHAQPGVGLRPDTPVASGQLPSTSIRSRRSGISKAQRETWERLWPELGLLALPQSPRGTPVDTRAWFGRDAPVVLEIGSGSGTSTLAMAKAEPHVDVIAVDVYRRGLAQLLCAIDKVGSDGINIRLILGNAVDVLQHLIAPDSLCGVRVFFPDPWPKARHHKRRLLQPATMALIADRLVPSGVLHAATDHPGYAEHIAAAGDAEPRLVRVDPDTELLPISVVRPATKYERKAQLGGGAVIELLWKKHGCSERDLKIR

>sp|P05845|TNSE_ECOLX Transposon Tn7 transposition protein TnsE OS=Escherichia coli OX=562 GN=tnsE PE=1 SV=1

MVRLATFNDNVQVVHIGHLFRNSGHKEWRIFVWFNPMQERKWTRFTHLPLLSRAKVVNSTTKQINKADRVIEFEASDLQRAKIIDFPNLSSFASVRNKDGAQSSFIYEAETPYSKTRYHIPQLELARSLFLINSYFCRSCLSSTALQQEFDVQYEVERDHLEIRILPSSSFPKGALEQSAVVQLLVWLFSDQDVMDSYESIFRHYQQNREIKNGVESWCFSFDPPPMQGWKLHVKGRSSNEDKDYLVEEIVGLEINAMLPSTTAISHASFQEKEAGDGSTQHIAVSTESVVDDEHLQLDDEETANIDTDTRVIEAEPTWISFSRPSRIEKSRRARKSSQTILEKEEATTSENSNLVSTDEPHLGGVLAAADVGGKQDATNYNSIFANRFAAFDELLSILKTKFACRVLFEETLVLPKVGRSRLHLCKDGSPRVIKAVGVQRNGSEFVLLEVDASDGVKMLSTKVLSGVDSETWRNDFEKIRRGVVKSSLNWPNSLFDQLYGQDGHRGVNHPKGLGELQVSREDMEGWAERVVREQFTH

>sp|Q813X6|TOX1_BACCR Ribonuclease BC_0920 OS=Bacillus cereus (strain ATCC 14579 / DSM 31 / JCM 2152 / NBRC 15305 / NCIMB 9373 / NRRL B-3711) OX=226900 GN=BC_0920 PE=1 SV=1

MSLNMYLGEVQGQTQSMNAVCNATIQGMEQVIQSIDAFAIDTVLQGQTYSSAKSFFVQTFRPLAQGIIYLCEELIRQNDAFPSQFQSQVASTDVIEQEILEQIREIDRMKASMEAISQAMPIPGMDAMANLFTVMRKKLQEKLDHLYQFNQTSSNNYSTALQLAASIAAGLAEVQSGKGFSPASGTFSTQGLNMEWTTSIQAITEERARQAANSIEEGEMCGKLPEKSTGEKIWDGIVEGTGQAVSDTIDGIKALGDWETWENMGNAALHPIDTLSTMYNTLSDSFINDVINGDAESRAKWGSYALTQVGLGLIGDKGLSKASKLGQAGKVTKLAKNKIPQAVSHITSNLQMGDRFAFAGGNSLRFRFDTPDFKKAEEKLSTYQFARGESNYGGSNFVNENHRSSLSNREIISNLQHTEKFRPNTLKHILEGEINWRGDAMGYHTEVLENTPGKIISGTEEILNDQGIYKARVEVNGTPKTGNRGFSTFFPKDWSPQKIVDNINEAYNNRTYEFGNTYSGIGSEGIRISMYIDGNGKIISAFPAE

>sp|P9WGV5|TAER_MYCTU Trans-acting enoyl reductase OS=Mycobacterium tuberculosis (strain ATCC 25618 / H37Rv) OX=83332 GN=Rv2953 PE=1 SV=1

MSPAEREFDIVLYGATGFSGKLTAEHLAHSGSTARIALAGRSSERLRGVRMMLGPNAADWPLILADASQPLTLEAMAARAQVVLTTVGPYTRYGLPLVAACAKAGTDYADLTGELMFCRNSIDLYHKQAADTGARIILACGFDSIPSDLNVYQLYRRSVEDGTGELCDTDLVLRSFSQRWVSGGSVATYSEAMRTASSDPEARRLVTDPYTLTTDRGAEPELGAQPDFLRRPGRDLAPELAGFWTGGFVQAPFNTRIVRRSNALQEWAYGRRFRYSETMSLGKSMAAPILAAAVTGTVAGTIGLGNKYFDRLPRRLVERVTPKPGTGPSRKTQERGHYTFETYTTTTTGARYRATFAHNVDAYKSTAVLLAQSGLALALDRDRLAELRGVLTPAAAMGDALLARLPGAGVVMGTTRLS

>sp|Q03304|TMOF_PSEME Toluene-4-monooxygenase system, ferredoxin--NAD(+) reductase component OS=Pseudomonas mendocina OX=300 GN=tmoF PE=1 SV=1

MFNIQSDDLLHHFEADSNDTLLSAALRAELVFPYECNSGGCGACKIELLEGEVSNLWPDAPGLAARELRKNRFLACQCKPLSDLKIKVINRAEGRASHPPKRFSTRVVSKRFLSDEMFELRLEAEQKVVFSPGQYFMVDVPELGTRAYSAANPVDGNTLTLIVKAVPNGKVSCALANETIETLQLDGPYGLSVLKTADETQSVFIAGGSGIAPMVSMVNTLIAQGYEKPITVFYGSRLEAELEAAETLFGWKENLKLINVSSSVVGNSEKKYPTGYVHEIIPEYMEGLLGAEFYLCGPPQMINSVQKLLMIENKVPFEAIHFDRFF

>sp|P24546|T2A1_ACICA Type-2 restriction enzyme AccI OS=Acinetobacter calcoaceticus OX=471 GN=accIR PE=1 SV=1

MDYYDRIRELTKNVPVELVDFEQPRDLARTPTQASSNFITNKEQGDWAEDLVTRAINENSKNFVAVKYGKSDNLVAGENGFDTFYQDFQTELDTIGKRPDLLIFKKTDFDTTLGFDVSQIPHHQITDYVKKAIAGIEVRSSAFLIDKYEEAMQVRTQRFTEIAFQTRDKILAEFLDVLDHPSRSKYITLLNTLTLETISIFDFKVPGWRSNERLIEVNNLFKRLKVAIKEIQKRDYLSITPKVEDIKVVYKWIETFNVPHFYFQVFFDKVYGISFEQILTIISNSDNDGVIFSVEKDVQNQNKTTIKINSKTGYPIASKVDEPTHESIRKEMDRGRLLFYVTFKGGTAYLDLDNLRTILGIEEAEF

>sp|P83615|TPAP_STRMB Prolyl tri/tetrapeptidyl aminopeptidase OS=Streptomyces mobaraensis OX=35621 GN=ptp PE=1 SV=2

MRKALRSLLAASMLIGAIGAGSATAEAASITAPQADIKDRILKIPGMKFVEEKPYQGYRYLVMTYRQPVDHRNPGKGTFEQRFTLLHKDTDRPTVFFTSGYNVSTNPSRSEPTRIVDGNQVSMEYRFFTPSRPQPADWSKLDIWQAASDQHRLYQALKPVYGKNWLATGGSKGGMTATYFRRFYPNDMNGTVAYVAPNDVNDKEDSAYDKFFQNVGDKACRTQLNSVQREALVRRDEIVARYEKWAKENGKTFKVVGSADKAYENVVLDLVWSFWQYHLQSDCASVPATKASTDELYKFIDDISGFDGYTDQGLERFTPYYYQAGTQLGAPTVKNPHLKGVLRYPGINQPRSYVPRDIPMTFRPGAMADVDRWVREDSRNMLFVYGQNDPWSGEPFRLGKGAAARHDYRFYAPGGNHGSNIAQLVADERAKATAEVLKWAGVAPQAVQKDEKAAKPLAPFDAKLDRVKNDKQSALRP

>sp|E0U4V7|TARQ_BACPZ Poly(ribitol-phosphate) beta-glucosyltransferase OS=Bacillus subtilis subsp. spizizenii (strain ATCC 23059 / NRRL B-14472 / W23) OX=655816 GN=tarQ PE=1 SV=1

MKISIVIPVYNSEDLISECLDSLVNQTMPKEDYEIICVDDKSTDSSLDILNQYKKKYENVVVIERTVNSGGPGAPRNDAIKIAKGEYILFVDSDDYIGSEALLRWYNFSKENQSDITLGKLKGINGRGVPKSMFKETNPDVDLVDSKIVFTLGPQKLFKASLLKENKITFPTHIKAAEDQVFTMNAYLKAKKISVSADYDYYYLVKRDGEHMSVAYVPPENFYGAMEDIISAIKASDLEEARKIKLMAVFLNRHFDFSRTKNVTIKMKTDEERAEWFRYLSSFIHAVPEEADQFVLPHIKLRLLFIRNNDLRGLTQYEREEQDIKKFCTVNNGELIARYPSLERYSISEELLKVNYKNKLEHYLQNIEFSDHSLSIQGTITHKLLDDETNKNQSLTGVFVHRDTKAEKYIAPASYDNSTFTFECKFDELASAEEDLGVWDFFIESSIDGYKLRARIGNKRAAYKYSTKTMYLGHNALFVYSARPYFTMNYDNLSIDIKKHAYTEAELSYETESKDLSFIFKDKQIYLPNHSKIIVNTGQSEISLPVKRIDLEPNCTKLTVNVQSLLEQLAHVKKERLIEFAINTSQNKISAKVDNQAIILDTKSVERKSMLFFNKMVEVQYKLLTSKSKFYFQY

>sp|P0AEV1|RSSB_ECOLI Regulator of RpoS OS=Escherichia coli (strain K12) OX=83333 GN=rssB PE=1 SV=1

MTQPLVGKQILIVEDEQVFRSLLDSWFSSLGATTVLAADGVDALELLGGFTPDLMICDIAMPRMNGLKLLEHIRNRGDQTPVLVISATENMADIAKALRLGVEDVLLKPVKDLNRLREMVFACLYPSMFNSRVEEEERLFRDWDAMVDNPAAAAKLLQELQPPVQQVISHCRVNYRQLVAADKPGLVLDIAALSENDLAFYCLDVTRAGHNGVLAALLLRALFNGLLQEQLAHQNQRLPELGALLKQVNHLLRQANLPGQFPLLVGYYHRELKNLILVSAGLNATLNTGEHQVQISNGVPLGTLGNAYLNQLSQRCDAWQCQIWGTGGRLRLMLSAE

>sp|P46850|RTCB_ECOLI RNA-splicing ligase RtcB OS=Escherichia coli (strain K12) OX=83333 GN=rtcB PE=1 SV=3

MNYELLTTENAPVKMWTKGVPVEADARQQLINTAKMPFIFKHIAVMPDVHLGKGSTIGSVIPTKGAIIPAAVGVDIGCGMNALRTALTAEDLPENLAELRQAIETAVPHGRTTGRCKRDKGAWENPPVNVDAKWAELEAGYQWLTQKYPRFLNTNNYKHLGTLGTGNHFIEICLDESDQVWIMLHSGSRGIGNAIGTYFIDLAQKEMQETLETLPSRDLAYFMEGTEYFDDYLKAVAWAQLFASLNRDAMMENVVTALQSITQKTVRQPQTLAMEEINCHHNYVQKEQHFGEEIYVTRKGAVSARAGQYGIIPGSMGAKSFIVRGLGNEESFCSCSHGAGRVMSRTKAKKLFSVEDQIRATAHVECRKDAEVIDEIPMAYKDIDAVMAAQSDLVEVIYTLRQVVCVKG

>sp|P46228|RS1_SYNP6 30S ribosomal protein S1 OS=Synechococcus sp. (strain ATCC 27144 / PCC 6301 / SAUG 1402/1) OX=269084 GN=rpsA PE=1 SV=4

MVTQDIPAVDIGFTHEDFAALLDQYDYHFNPGDTVVGTVFNLEPRGALIDIGAKTAAFLPVQEMSINRVESPEEVLQPSEMREFFILSDENEDGQLTLSIRRIEYMRAWERVRQLQTEDATVRSEVFATNRGGALVRIEGLRGFIPGSHISTRKAKEDLVGEELPLKFLEVDEDRNRLVLSHRRALVERKMNRLEVGEVVVGAVRGIKPYGAFIDIGGVSGLLHISEISHDHIETPHSVFNVNDEVKVMIIDLDAERGRISLSTKQLEPEPGDMVRNPEVVYEKAEEMAAQYREKLKQQAEGLVVTE

>sp|B0FYK7|RPPA_STRC0 1,3,6,8-tetrahydroxynaphthalene synthase OS=Streptomyces peucetius subsp. caesius OX=55158 GN=rppA PE=1 SV=1
[truncated: 254,155 more chars]
