# Supplementary material for: BacEffluxPred: A two-tier system to predict and categorize bacterial efflux mediated antibiotic resistance proteins
Source: Sci Rep. 2020 Jun 9;10:9287. doi: 10.1038/s41598-020-65981-3 (PMC7283322; doi:10.1038/s41598-020-65981-3)
Supplement: Supplementary file 2 — Supplementary Information- Tier II Dataset. [file 41598_2020_65981_MOESM2_ESM.zip › BacEffluxPred_Supplementary_materials_Tier-II_Dataset.docx]

**Supplementary Information**

**BacEffluxPred: A two-tier system to predict and categorize bacterial efflux mediated antibiotic resistance proteins**

Deeksha Pandey^1,2^, Bandana Kumari^1,3^, Neelja Singhal^1,4^ & Manish Kumar^*^

^1^Department of Biophysics, University of Delhi South Campus, New Delhi, India

^2^Email: deeksha.pandey.biophysics@south.du.ac.in
^3^Email: vandanachaurasia.1@gmail.com

^4^Email: neelja30@gmail.com

*Correspondence to: Manish Kumar, Department of Biophysics, University of Delhi South Campus, New Delhi, India – 110021 Telephone Number: +91-11-24157263

E-mail: manish@south.du.ac.in

**Tier-II dataset**

**The dataset used to train Tier-II (family predictions) SVM models**

1. **ATP-binding cassette (ABC) : 41 protein sequences**

>1071078.3.peg.1866_ABC

MVDLLYTELLKLKRSQMFLVSILGAAAAPFICFISSLAKKAKYPDVPIRFSETFSDTNLYIVLLIGVPLYGVITSYLFNREYAESTLKNLLTIPVSRISLIISKLVLLFIWIMLLTLIAWVLTLLFGLIGQFEGLSSAVLIEELKQFMTGGALLFFLLSPIIFVTLLFKNYVPTIIFTIIISMVSIMVYGTEYSALFPWSAVWVIASGTFFPEYPPAFSFISVIATTVLGLAATIVYFKKIDIH

>1121863.3.peg.2626_ABC

MPSRSRRLNCSLFCFLWVAAFMELLRLVWRQYRLPFVLVLALSLASAALGIGLIAFINQRLIATVDLSLAVLPAFLGLLLLLMAVTLASQLALTMLGHHFVYRLRSEFIKRIMDTPVEQVEKLGSATLLAGLTSDVRNITVAFVRLPELVQGIILTIGSAVYLGWLSSKMLLVTAVWIAITLWIGYLLVQRVYKHIATLREVEESLYNDFQTLLEGRKELALNRERAEYIFDQVYKPDAQSYRQHIIRADTFHLSAVNWSNIMMLGVIGLVFWMANGLGWADTNVAATYSLALLFLRTPLLSAMGALPTLLTAQVAFNKLKQFQLADYEPAFKRPQKFADWQTLELRDVTFKYSDGSFGVGPLNLTIKRGELLFLIGGNGSGKSTLAMLLTGLYEPASGDILLDGKVIATSEMEAYRQHFSAVFTDVWLFDKLLGPQGEEADPALVDAWLNRLKMAGKLELDNGKILNLKLSKGQKKRVALLLALAEDRDIILLDEWAADQDPHFRREFYQVLLPLMQQMGKTIFAISHDDHYFIHADRLLEMRQGHLSELTGDERALASRDAVARTGS

>1156433.3.peg.1263_ABC

MKVLKQLLSRITLYPTVFLVGFICLLLATIFSELSPFILQKMIDGPLTALTHSGEQGQLLQMGGFYLLVLSIGQLISYLGNRILLHGSNQVTANLRDQAFQVMQGLPISYFDDKPAGKIATRIVNDTETLRTQFYNSCMVLIIFLVRFLFVLGILFYLSPMMGLLLCLVFPIFYGIQYLYKVMTDQPMKDFFDARSEVNTQVNELLHGASMIQLYGQEPHVIEEFEATTQKMLWANDRILLADSIASWTLTELLKYLVIAGILTIAGMSYLKGNIGVTAGFLFININYVMNLFELMAALSRQFPNIRRSLETGSRVLAFLDQPLEADGVLELKIEKAQVVFDDVQFAYEEGKPVLQDIAFQASPGETIALVGHTGSGKSSIMNLLYRFYDPQDGAILIDGQDIRQVSRESLRSHMGIVLQDPYLFTGTIASNVAMSQDHIDRDAIKDALKKVGAWPFVERLEKGIDHPVVEKGSAFSSGERQLISFARTLYMNPQILILDEATSHIDTETEEIIQKAMAVLQKGRTTFIIAHRLSTIQDADKILVLSEGRIVERGQHADLIAHGGIYAQMHAIQQTVE

>1156433.3.peg.1264_ABC

MIGAIWEYIRERKWRYISIAVVLILYDYTLLIPTQVIQRLVDHLSQQTLTQSNFVWDMVLLVGSAILNYLTAFYWQLRLFQSSIHFKSTLQEQAFRKLVAMRRPFFEKFRSGDLLTRFTTDVDGMADMAGYGMMIILYGGGLFTFIILAMFFLSWQLTLICFIPMIFLVVSTYFLSKKQEDYIEQNREAVAQLNDEVLESIEGIRVMRAYSRRDQQVKQFQTKTASLAKTGDKIASIQYSFGPLALLFIGVSTVLLLVFGGQSLASGQLSLGKLLALQLYLVFLVEPMWMLSDFILVYQTGQMSFKKLKEVIDETDDLEPDGPHFLEQIDSVEFKDYSFRYPGAERESLSGIDWTVQKGQTVGIVGRTGSGKTSLVRQFLRQYPVGEGEFLVNQQPIVAYNRRSIEDKIGYVSQEHILFSKSIRDNIALGKNGASEEDLVEAVAQAAFADDLERMSQGMDTMIGEKGVSVSGGQKQRISLARAFLRDADFLLLDDSLSAVDAKTEQAIIDSIQTERKGKTTIIVSHRLSAVHQADWIIVLDQGQIVEEGRASDLLAQEGWYYEQYQRQQKQEGE

>1158608.3.peg.145_ABC

MKLMWRYTMRYKKLLFLDFICVFGFILIELGLPTILARMIDVGIKNNDYDYVKQQGLLMIVITVIGVAMNIMLGYFGARMTTNIVRDIRDDLFEKVQTFSHREYETIGVSSLITRTTNDAYQIMLFMGNILRIGFMTPMMFFVSLYMVMRTSPSLGWFVLGALPFLLAAVVLIAKVSEPLSNKQQKNLDGINGILRENLSGLRVIRAFVNEKFEESRFSKVNEDYTKSSKSLFRLMAAAQPGFFFLFNIVMVLIIWNGALQIDQGSLLVGDLIAFIEYIFHALFSFMLFASVFMMYPRAAVSARRIQEAFDMEPVIRENEAGITETKTKGYLEFKNVTFAYPGHSESPVIRNVSFTASPGETVAFIGSTGSGKSTLIQLIPRFYDVSEGEVLLDGIDVRDYKLSALRNKIGYIPQKALLFTGTIAENMRYGKEDATIEEMELAADIAQATEFISQKPDGYDELLSEGGTNFSGGQKQRLAIARAVIRRPEVYIFDDSFSALDYQTDANLRARLKKETTESTVLIVAQRVGTIMHADKIVVLNEGDVVGIGTHRELLENCPIYYDIAASQLSEEELA

>1232427.4.peg.1332_ABC

MVRDMVGAYPGVLVLHILSFLIGSGIAAFAPVVVGMIVDGLVGEEKFNAWWLFGVLVGIFIIQFAGEATGDGLAAASVRRVTHNAQQHLSSGVLRRGAGAMSPGTVLNTIDADANTIGRYRELLSFPLMAIGYAAGAIVAMWTVSPWVSLAIPVSALVIALFAAWTAGPVTRVSLKRRAAEADVASLATDTSQGLRTVKGLGAGGTVAHRFHTETAKAKRLMLTHLRVEVWLGFARLCVAWLCNLGIVGLAAWMTLRGEITPGQLTSVALLVPPALNMAGFAFGDLASGWGRAVASGQRIQQLHHAGDDTAGPEPTDTPVPGAGLWILEPAERSYATAVAWAQRADVLFPPHTVNVFEGTIADNVNPRGDVPEDAVKQALAAAHCQDILRRLGGIGENGELPDAPLGEAGLNLSGGQRQRVALARALAADPEVLILDDPTTGLDSVTQADVVEAVAVLRADKTTVVITGNSAWQHAGTALEVA

>1234876.3.peg.1456_ABC

MENTKSTRKMSDTTRAIRFFYLYLKRYKLQFAVIMIFIVAATWLQVIAPSLLGDAITNLGVYVKDFFTHQHAGQSQDALQQIAQQLSQQMHQTVDWHNVPEVVKTLPQAAQDQITAHLPKGTTLETLKTVATSHAASTSTFMKGMWQLLAVYVATGVSMLIYTLLFSRIVAHSTNRMRKGLFGKLERLTISYFDRHQDGDILARFTSDLDNIQNTLNQALVSVISNAAVFVGVIIQIFLKDVTFAWLTVAASPVAILSAVIIIRQSKKATDKQQEEVSQLNAYMDEKISGQKAIIVEGLQEDSIDGFLKHNENVKKRTFAAQAWSGMIFPLMNGFQLLSIAIVIFGGTAYVLNNDSMSITTGLGLLVAFVQYVQSYYNPIMQISSNFGQLQLAITGATRLNVMFDEPEEVRPENGKKFDTIKDGIQIENLDFEYLPGKPVLKKVNIDVKKGQMVALVGPTGSGKTTVMNLMNRFYDVNGGAIKFDGTDIREFDLDSLRSNVGIVLQESVLFDGTIADNIKFGKPNATQEEIETVAKTTHIHEFIESLPDKYETHVSDDESVFSVGQKQQISIARTILTNPELLILDEATSNVDTVTEEQIQWAMEAAIAGRTSFVIAHRLKTILNADKIVVLKDGEVIEEGNHHELVAQGGFYSELYHNQFVFE

>1234876.3.peg.1457_ABC

MIFKSIMKHKWVALFSIFSTFVYAGVQLYQPQIMKRIMTVMSSTTYSRHEMADKVSGYGVELLIVAGIGILFAIFSTLSAARIAQEIGADVREATYKKINTFSYENVEKFNAGNLVVRMTNDVTQVQNLMMMVFQILMRIPVLLIGAVILSITTLPKLWWITVLLIVLILVVTAVLMGRMGPHFMAFQKLMDRINAIAKQNLRGARVVKSFVQEKNQIKEFDETSDELYDHNWAVGKLFSAMIPLFTVIAQGAIWLAIYFVSTFVTDSPTVAQDSIGGIATFMTYMGMIMFAIIMGGMISMFASRGMVSIGRINEVLKTDPAMKFDENAKDEELSGSVKFDHVSFSYPNDEEPTLKDISFEVEAGQMVGIVGATGAGKSTLAQLIPRLFDPTEGTVSVGGKDLKTVSRGTLKRNISIVLQKAILFSGTIAGNIKQGKSDATDEEMTRAAQIAQAAEFITTKDGQYESEVEERGNNFSGGQKQRLSITRGVVKNPNVLILDDSTSALDAKSEKLVQEALNKELKETTTIIIAQKISSVVHADNILVLDQGKLVGQGTHQELVAENKIYQEIYDTQKAQED

>1413510.3.peg.681_ABC

MLFLFEEKALEVEHKVLIPELTFSIEDHEHLAIVGVNGVGKSTLLKVIHQDQTVDSAMMEQDLTPYNDWTVMDYIIESYPEIAKVRSQLNHTDMINKYIELDGYLIEGEIVTEAKKLGIKEEQLEQKISTLSGGEQTKVSFLKVKMSKASLLLIDEPTNHMDLEMKEWLTKAFKQEQRAILFVSHDRTFLNETPDAILELSPDGAKKYIGKYDKYKQQKDIEHETLKLQYEKQQKEQAAIEETIKKYKAWYQKAEQSASVRNPYQQKQLSKLAKRFKSKEQQLNRKLDQEHIANPNKKEKAFSIQHHDFKSHYLVQFNHVSFAYDNRKIFEDVSFYIKRNQNVIIEGRNGTGKSTLIKLILGELEPTKGNITVHPELEIGYFSQDFENLNMHNTVLDEILEIPEMNEADARTILASFYFDKDRINDVVETLSMGEKCRLQFVKLYFSNPHIMILDEPTNYFDIGMQEKIIQLIQSFQGSVLIVSHDDYFKSQIKDQIWTIKNHQMTHENVQVKDPINTESMKHQLKELEQYTEERNRETEF

>1428628.3.peg.4541_ABC

MRPDSQITWTPPADAKEQPRQVRRILGLFRPYRGRLAIVGLLVGAASLVSVATPFLLKATLDTAIPQGRTGLLSLLALGMILSAVLNSVFGVLQTLISTTVGQRVMHDLRTAVYGRLQRMSLAFFTRTRTGEVQSRIANDIGGMQATVTSTATSLVSNLTSVVATVVAMLALDWRLTVVSLVLLPAFVWISRRVGNERKKITTQRQKQMAAMAATVTESLSVSGILLGRTMGRADSLTESFADESERLVDLEVRSNMAGRWRMAVITIVMAAMPAVIYWTAGMALQMGGPKVSIGTIVAFVSLQQGLFRPAVSLLATGVQIQSSLALFQRIFEYLDLPIDITERQDPVHLDRVKGEVRFEDVAFRYDDKSGPILDGIDITVPAGSSLAVVGPTGAGKSTLGYLVPRLYDVTGGRVTLDGVDVRDLDFDTLARAVGVVSQETYLFHASVADNLRFAKPDATDEELHAAAKAAQIHDHISALPDGYDTVVGERGHRFSGGEKQRLAIARTILRDPPVLILDEATSALDTRTEHAVQEAIDALSANRTTLTIAHRLSTIRDADQIVVLDGGRTAERGTHEELLELDGRYAELVRRDARQQPQAEARPEAQARTGPEPRVDSQPSRRGGSRPSPHGGGRPSPRGDSRPGPRVDSGLEPTS

>1460652.3.peg.5862_ABC

MSMIQVQDLTFSYPSSFDNIFEGVNFQIDTDWKLGFIGRNGRGKTTFFNLLLGNYEYSGKIISSVQFNYFPYPVSDKNKYTHEIFEEICPQAEDWEFLREISYLKVDAEVMYRPFKTLSNGEQTKVLLAALFLTEGQFLLIDEPTNHLDTGARKIVSDYLRKKKGFILISHDRIFLDGCVDHILSLNRANIEVQKGNYSSWKLNFDRQQEHEEATNQRLQKDIGRLKQASKRSAGWSNQVEASKNGTRNSGSKVDKGFVGHKAAKMMKRAKNLESRQEKAIEEKSKLLKNVEKTESLKLAPLEFQSNELIVLTDVSIKYDDQIVNKPISFNVEQGDRIVLMERMEAEKVVF

>1463857.3.peg.5322_ABC

MTATSTNETGATAGAAAGTTTEATHAAGGAAVERGAPPQGSAPPGGPGTRAGAEGRIGLRAHLRHIGALARRNALQIKQDPESMFDVLLMPIVFTVLFVYVFGGSVGASLGGDRHDYLNYVVPGLMAMMGMNIAMAVGTGMNDDFRKGVMDRFRTMPIARSSVLIAKIVVEVGRMIVATAILLGMGFALGMTVQTSVLGLLAAVGLSLLFGAALMWIFILLGLTMKTAQAVQGVAMIVLMPLQFGSSIFAPTKTMPGWLQAFTDYNPLSNLADAARGLVNGGPVAHSAWMTLAWAAGITLVMAPLAVRKFRDKT

>1638.4.peg.885_ABC

MSIIEINQLKIEVADRVLVEIPHLLVNQKARIGIIGQNGLGKTTLIEVIAGVQEPAVGKVTIQGRLAYIKQLPTDKSTKSGGEKTRKAIQQAMRQNPSVLLADEPTSNLDVESVKHLERQWKDWHGSLIIISHDRAFLNSLCTEIWEIKDQKIQVYKGNYQAYLKQRKQQENQAELAYKEFKNKKKQLEASQNYHEVEAGRIVKPGKRLNAKEASAFKAGKGTQQKKQHSTIKALDKRIERLGNVEKPHKAKPIKISTPENRIIKKGNTILTAAEATYEIAGKKLFSTTGFSIKSGDKVALIGENASGKTSFLKQILQNNSKLVCSNQAKIAYFDQELQGLDLTKTLLENMIDISVQSKQMTKEVLGSMHFKETDWHKKASLLSGGERVKLLLSMLLVSDANFLILDEPTNYLDIFAMEALETLIQNFTGTVLFVSHDRTFVSQVAEQLLVIESGKMAFYRMAFAEYEASITPSRITEEDKLILEMRMSEIAAKLMQPNLKAEDKALLEKDYQEVITKRRQFN

>226185.9.peg.2536_ABC

MSKIELKQLSFAYDNQEALLFDQANITMDTNWKLGLIGRNGRGKTTLLRLLQKQLDYQGEILHQVDFVYFPQTVAEEQQLTYYVLQEVTSFEQWKLERELTLLNVDPEVLWRPFSSLSGGEKTKVLLGLLFIEENAFPLIDEPTNHLDLAGRQQVAEYLKKKKHGFILVSHDRAFVDEVVDHILAIEKSQLTLYQGNFSIYEEQKKLRDAFELAENEKIKKEVNRLKETARKKAEWSMNREGDKYGNAKEKGSGAIFDTGAIGARAARVMKRSKHIQQRAETQLAEKEKLLKDLEYIDSLSMDYQPTHHKTLLTVEELRLGYEKNWLFAPISFSINAGEIVGITGKNGSGKSSLIQYLLDNFSGDSEGEATLAHQLTISYVRQDYEDNQGTLSEFAEKNQLDYTQFLNNLRKLGMERAVFTNRIEQMSMGQRKKVEVAKSLSQSAELYIWDEPLNYLDVFNHQQLEALILSVKPAMLVIEHDAHFMKKITDKKIVLKS

>226185.9.peg.2721_ABC

MKHAFSSMKRIGRYIKPYRVTFYLVILFTILTVAFNAALPYLTGLPTTEISRNIAAGESINFDYVIQCLIWILVVGTGYCVAQFLSGFLMTNVVQQSMRDLRRDIEEKINRLPVSYFDKNQQGNILSRVTNDVDAVSNAMQQSFINIVSAVLGIVMAVVMMFLINPLMAIFSVIMIPLSLIISRTIVKISQKYFQGMQNSLGDLNGYVQENMTGFSVLKLYGREKETLEGFKQVNHRLNGFGFKASFISGLMLPLVQMTAYGTYIGVAVLGSYYVVAGVIVVGQLQAFIQYIWQISQPMGNITQLSAALQSASASTMRIFEILDEPEEELNEQDVPLPEPILGSVEFENVSFSYDPEKPLIRNLNFKVDAGQMVAIVGPTGAGKTTLINLLMRFYDVTEGAIKIDGIDTKKMNRSDVRSVFGMVLQDAWLYKGTIADNIRFGKLDATDYEVVDAAKTANVDHFIRTMPDGYEMEINSEGDNVSLGQKQLLTIARAVISDPKILILDEATSSVDTRLEALIQKAMDRVMEGRTSFVIAHRLSTIREADLILVMKQGEIIEKGTHHELLEQGGFYEKLYNSQFAEEGDYEE

>29379.8.peg.680_ABC

MSFMIRRYLRFVKPYKWRIIITIIVGIIKFGIPMLIPLLIKYVIDDVINNGEIDTQQKMLRLAIALGIAIFIFVVIRPPIEFIRQYLAQWTSNKILYDIRRHLYNHLQALSARFYANNQAGQVISRVINDVEQTKDFILTGLMNIWLDCVTIVLALTIMFFLDVKLTLAAMFIFPFYIITVYFFFGRLRKLTRKRSQALAEVQGFLTERVQGMSVVKSFAIEENEAENFDAHNQHFLDRAFKHTRWNAYAFAAVNTVTDIGPLIVIGIGGFLAINGSITVGTLAAFVGYLEQLFSPLRRLVSSFTTLTQSFASMDRVFQLFDEDYDIKNKKGAQPIAIQQGDISLDHVYFKYNEDEDMILRDINLDVHQGETVAFVGMSGGGKSTLINLIPRFYDTTKGSITIDQHPIKDFLTGSLRSQIGLVQQDNILFSDTIRENILLGKPDATDEEIVQAAKMANAYDFIQELPHGFETEVGENGVKLSGGQKQRISIARIFLNNPPIIILDEATSALDLESEAIIQDALNVLSEDRTTLIVAHRLSTITHADKIVVMENGQIVETGTHQELLDRNGQYAHLFNIQNL

>315749.8.peg.286_ABC

MYVKDHRKWVFTIHTIIKTTNLTKVYGKQKSVDHLNINVNKGEIYGFIGRNGAGKTTTIRMLLGLIKPTNGKIEIFGEDFTKNQKDILRRIGSIVEVPGFYENLTAKENLLINAKIIGVHKKNAIEEALEIVGLQHETKKLVGKYSLGMKQRLGIARALLHYPELLILDEPTNGLDPIGIKEMRKLIKTLAQERNITIFISSHILSEVEQLVDHMGIIHKGKLLEETSLDALRKMNRKYLEFQVNNDNKAALLLEKQFHIFDYEVHDEGNIRVYSHFGQQGQINKMFVQNDIEVLKIIMSEDRLEDYFTKLVGGGTIG

>33009.3.peg.2745_ABC

MPTAQCALHDITKRYDDRVVFDRIGFSIAPGEKVGVIGDNGSGKSTLLKLLAGRERPDDGTLTVVAPDGVGHLAQTLELPLHATVQDAVDLALSDLRELEAAMRRAEAELAEHDTDGPGTELSATLRHYADLVERYQARGGYEADVRVEVALHGLGLPGLDRARELGTLSGGERSRLALAATLASAPELLLLDEPTNDLDDRAVEWLEEHLRGHRGTVVAVTHDRVFLDRLTTTVLEVDSGRVTRYGNGYEGYLTAKAVERERRLREYGEWRAELVRNQGLIASNVARMDGIPRKAPLSVFGHGAYRRRGRDHGAMVRIRNAKQRVAQLTENPVPAPADPLSFTARIDTSGPGAGEAEEAEEAVAELTGVRVADRLAVDSLRIRPGERLLVTGPNGAGKTTLLRVLSGELEPDGGSVRAGCRVGHLRQDETPWPPEATVLRAFAHGRDGYLDDHAEKLLSLGLFSPSDLRRRVGDLSYGQRRRIEIARLVSDPMDLLLLDEPTNHLTPVLVEELEQALVDYRGAVVVVTHDRRMRSRFTGARLTMEHGRVTGFRAA

>333849.13.peg.130_ABC

MENLAVNITNLQVSFGNQLELSIDSLRVYQQDRIGIIGENGVGKSTLLKLIAGELFPDHGKIQTEITFNYLPQLTYLAEAKDLNLELASHFQLRLEETSERKWSGGEERKIELIRLLSSYEQGMLLDEPTTHLDRKSIDRLIEELRYYYGTLVFVSHDRYFLDELASKIWEVKDGEIREFSGNYSAYLTQKELEKKTQLREAESIMKEKKRLEKSIQEKKKQAEKLEKVSSKKKKQQIRPDRLSSSKQKDSVQKAIQKNAKTLERRLQKIGETTKPQQMKQIRFPVPKSLELHSRYPIMGQNVQLERSGRTLLVNGDFQFSLGKKIAIVGENGSGKTTLLEHIRKQGEGILLSPKVSFQVYQQKGYQMTSEESIIRFVMRQTEFSESLVRSLLNHLGFAQETLTKPLCTLSGGEATRLTIALLFTKPSNVLLLDEPTNFIDMATIEALEKLMQIYPGTILFTSHDSYFVERTADEVYEIKGQKIKKVLTRNF

>411235.3.peg.357_ABC

MELRTNPKITPAVQVRRLTKHHGETTALDGVDLDVAEGTVMGVLGPNGAGKTTLVRILSTLVRPDAGARRRGGLRRRAPALPAAPGHRPHRAERLRRRAALRLRNLYLIGRLLDLNARGARPPGPSRRVRRPRAQRRGRGADGPPGSAGRPAGRGRTGLAPDLLDQRPGRCGSPLAQASAAARARSRAPERATGRWSSSTTSVLPRIPKRIPTF

>66692.6.peg.3722_ABC

MNTGQLVMRNLRKNSKTYGLYIFSLTFSAALYFAFVTLQYDPALDEAAASVKGAAAIQSASVLLIAIIAIFLLYANRLFLKRRSKEIGLFQLVGMRKGRIFWVLSGENVLVYFGALAVGIFIGFYLSKLAMVSLYRIIGVETAAKLHFSGAALTQTLLVFAAICVLMMGFTYVYIRKQTILSLFHIKGKTEMVAQGLRAFEIAFGVLGIVLIGFGYWLSSKLFEGQFVTQNELFLAMTTILAACVFGTWLFYKGTVSFVAKVIRRKKDGYLNIREVMSLSALMFRMKSNAVLLTVITTVSALAIGLLSLSYITYYSAEKSAKQWVPTDFAFTSVEDAEAFKNKLDQTGVDYQERTTEFIQGNVNVEGIINSSTEMMTGTAQEMAITSAAYMTDVDISPDEAVLTGSNDLLQRFVTFKEEGEIVIELGEETLSQHYRGLKKEFVLPSFYKVAGGMPTVVVNEETFVSLKKQQEAETSYGIDIIKESEVVAANSAYQEMDFQEQSESQWAMATNQKAHMGLYMFIVAFLGLTFLITSGCILYFKQVDETEGEKHNYTILRKLGFNRRQLEKGSYGKQLFAFGIPLLLGLSHSYFAVQSGWFFFGGELWTPMLLVMAVYTVLYSIFALLSVSHTKKVIKESL

>66692.6.peg.3723_ABC

MVVLEAANICKQYGNKWNKQEVLKGLDLTIEKGEFVSIMGASGSGKTTFLNVLSSIDQVTSGTIIIDGKDITTMKERELAWFRQRHLGFVFQEYHLLETLTVKENILLPLSVMGAGKKEADEAFRSVAAELGIYELKDKYPNELSGGQKQRTSAARAFIHQPSMIFADEPTGALDSKSATDLLNKLTEFNQKYEATILMVTHDAAAASFSRRVVFIKDGQMFTEVMQQDRTREDHYEEIMKTQAILGGIKA

>73044.3.peg.968_ABC

MDGLTDRPHPQFGHDPFGGVVAHLSDADDPLQPPLLEPEPYGGRGGLGGQPLPPVGASQPPADLDRRQYLRQEAGHREAGEPGQLAGGPDLHGEQTEALRLPLALPGPDPTAGLLLVTDAAVTDPPHDHGIGVDGSHRRDVFLAPATQDQAGSLKRDHPVILPCCRPSVGGLSVAPGTVLLMTRIDENPGGGRSAVSVRGMVKHYGETKALDGVDLEVREGTVMGVLGPNGAGKTTLVRILSTLITPDAGEALVAGYDVVRQPRQLRRVIGLTGQYASVDEKLPGWENLYMIGRLLDLSRKDARRRADELLERFSLTEAAKRPASTYSGGMRRRLDLAASMIGRPQVLFLDEPTTGLDPRTRNEVWDEVKAMVGEGVTVLLTTQYMEEAEQLASELTVVDRGKVIAGGRIEELKAKVGGRTLRVRPIDPLQLEPLATTLDELGITGLATTTVDRQTGTLLVPILSDEQLTAVVGAVTARGITISSIVTELPSLDEVFLSLTGHRASAPQDATPADSREEVAV

>768710.3.peg.1877_ABC

MELIVKAKDIRLEYTGRDVLDIDELELYDYDRIGLVGANGAGKSSLLKVLLGELTLPGCKINRLGRLAYIPQLEEAILEEVKDFALIGKLGVSQIEVQTMSGGEETRLKIAQALSEQVHGILADEPTSHLDREGMDFLIGQLNYFSGALLVISHDRYFLDEVVDKIWELNDGKITEYWGNYSDYLRHKEEERQSQAARYEQFVAERNRLERAAEEKRKQARKMDRKAKGAAKKNSSESGGRLGHQKTMGSKQKTLFNAAKSMEHRIAALGEAEAPENIRTIRFRQSKTLELHNPYPIIGTEINKGFGDKVLLEKASFSIPLGAKAALTGGNGSGKTTLIQMILNREEGISISPKAEIGYFAQNGYKYNRNQEVMEFMMEDCDYNISEIRSVLASMGFVQNDIGKRLAVLSGGEMIKLQLAKMLMGRYNILLMDEPSNFLDLPGLEALEVLMKGYAGTIVFITHDQWLLDNVADMIYEIKSKKLNLIR

>904314.5.peg.208_ABC

MKIMLEGLHIKHYVQDRLLLNINRLKIYQNDRIGLVGKNGNGKTTLLHILYKKIVPEEGIVKQFSHCELIPQLKLIESTKSGGKVTLNYIRQALDKNPELLLVDEPTTNLDNNYIEKLEQDLKNWHGAFIIVSHDRAFLDNLCTTIWEIEEGRITEYKGNYSNYVEQKELERHREELEYEKYEKEKKRLEKAINIKEQKAQRATKKPKNLSLSEGKIKGAKPYFAGKQKKLRKTVKSLETRLEKLESVEKRNELPPLKMDLVNLESVKNRTIIRGEDVSGTIEGRVLWKAKSFSIRGGDKMAIIGSNGKGKTTFIKKIVHENHGISLSPSVKIGYFSQKIDTLELDKSILENVQSSSQQNETLIRTILARMHFFRDDVYKPINVLSGGERVKVALTKVFLSEVNTLILDEPTNFLDMEAIEAFESLLKEYNGSIIFVSHDRKFIEKVATRIMTIDNKEIKIFDGTYEQFKQAEKPTRNIKEDKKLLLETKITEVLSRLSIEPSEELEQEFQNLINEKRNLDK

>P0A9U1_ABC

MNDAVITLNGLEKRFPGMDKPAVAPLDCTIHAGYVTGLVGPDGAGKTTLMRMLAGLLKPDSGSATVIGFDPIKNDGALHAVLGYMPQKFGLYEDLTVMENLNLYADLRSVTGEARKQTFARLLEFTSLGPFTGRLAGKLSGGMKQKLGLACTLVGEPKVLLLDEPGVGVDPISRRELWQMVHELAGEGMLILWSTSYLDEAEQCRDVLLMNEGELLYQGEPKALTQTMAGRSFLMTSPHEGNRKLLQRALKLPQVSDGMIQGKSVRLILKKEATPDDIRHADGMPEININETTPRFEDAFIDLLGGAGTSESPLGAILHTVEGTPGETVIEAKELTKKFGDFAATDHVNFAVKRGEIFGLLGPNGAGKSTTFKMMCGLLVPTSGQALVLGMDLKESSGKARQHLGYMAQKFSLYGNLTVEQNLRFFSGVYGLRGRAQNEKISRMSEAFGLKSIASHATDELPLGFKQRLALACSLMHEPDILFLDEPTSGVDPLTRREFWLHINSMVEKGVTVMVTTHFMDEAEYCDRIGLVYRGKLIASGTPDDLKAQSANDEQPDPTMEQAFIQLIHDWDKEHSNE

>P0AFP9_ABC

MFHRLWTLIRKELQSLLREPQTRAILILPVLIQVILFPFAATLEVTNATIAIYDEDNGEHSVELTQRFARASAFTHVLLLKSPQEIRPTIDTQKALLLVRFPADFSRKLDTFQTAPLQLILDGRNSNSAQIAANYLQQIVKNYQQELLEGKPKPNNSELVVRNWYNPNLDYKWFVVPSLIAMITTIGVMIVTSLSVAREREQGTLDQLLVSPLTTWQIFIGKAVPALIVATFQATIVLAIGIWAYQIPFAGSLALFYFTMVIYGLSLVGFGLLISSLCSTQQQAFIGVFVFMMPAILLSGYVSPVENMPVWLQNLTWINPIRHFTDITKQIYLKDASLDIVWNSLWPLLVITATTGSAAYAMFRRKVM

>P0AFQ2_ABC

MSNPILSWRRVRALCVKETRQIVRDPSSWLIAVVIPLLLLFIFGYGINLDSSKLRVGILLEQRSEAALDFTHTMTGSPYIDATISDNRQELIAKMQAGKIRGLVVIPVDFAEQMERANATAPIQVITDGSEPNTANFVQGYVEGIWQIWQMQRAEDNGQTFEPLIDVQTRYWFNPAAISQHFIIPGAVTIIMTVIGAILTSLVVAREWERGTMEALLSTEITRTELLLCKLIPYYFLGMLAMLLCMLVSVFILGVPYRGSLLILFFISSLFLLSTLGMGLLISTITRNQFNAAQVALNAAFLPSIMLSGFIFQIDSMPAVIRAVTYIIPARYFVSTLQSLFLAGNIPVVLVVNVLFLIASAVMFIGLTWLKTKRRLD

>A0LM36_ABC

MDLIELQDIRKTYRLGEIDVPVLRGISLKVSPGDFVALMGTSGSGKTTLMNILGCLDRPTSGHYRFDGQDVVDLTPDQRAALRNRKIGFVFQNFNLLPRMSAVENVMMPLSYAGGGVSDQNGRERAGALLTRMGLGEHLDNEPSQLSGGQQQRVAIARALINNPSLLFADEPTGNLDSATSEEVLRVFQRLNEEEGVTIILVTHDPSVAQCARRIVRIRDGVIEPESGAVGDMPQVSKAAPAQSKPVHSAMRRGDLDKFRRSLHTALSSLRRNVLRAALTTLGIIIGVAAVIAMMEIGRGSSTAIQRTIASMGAHTLALLPGTAASGGVSFGGGSVMTMTPQDSEAIVNECPAVLAAAPIVRARTQVVHGSRNWVPAGIYGTTPTFLEIREWPLAEGDVFTERDVRNASKVCVLGQRLVDELFQGENPIGLEVRIKNVAFKVIGVLSPKGANMMGMDQDDLLLAPWTAIKYRVTGSSLANVNQSAASTSSASITDQVNSLSNLYPTEKVVLYPEISTTQAFDTPLPVRFTNVDQILVGIRSTSGTRAAIRQIGEVLRERHRLRPGEPDDFSVRDMTEMTKTLASTATMMTKLLLAVALISLIVGGVGIMNIMMVSVTERTREIGLRMAVGARAKNILQQFLFEAVLLCFLGGAVGILVGRGISHLVTVLLNWPTELSLDAILAAVGVSATVGIVFGYYPAWKASRLDPIVALRYE

>P75830_ABC

MKKRKTVKKRYVIALVIVIAGLITLWRILNAPVPTYQTLIVRPGDLQQSVLATGKLDALRKVDVGAQVSGQLKTLSVAIGDKVKKDQLLGVIDPEQAENQIKEVEATLMELRAQRQQAEAELKLARVTYSRQQRLAQTKAVSQQDLDTAATEMAVKQAQIGTIDAQIKRNQASLDTAKTNLDYTRIVAPMAGEVTQITTLQGQTVIAAQQAPNILTLADMSAMLVKAQVSEADVIHLKPGQKAWFTVLGDPLTRYEGQIKDVLPTPEKVNDAIFYYARFEVPNPNGLLRLDMTAQVHIQLTDVKNVLTIPLSALGDPVGDNRYKVKLLRNGETREREVTIGARNDTDVEIVKGLEAGDEVVIGEAKPGAAQ

>P9WG20_ABC

MITTTSQEIELAPTRLPGSQNAARLFVAQTLLQTNRLLTRWARDYITVIGAIVLPILFMVVLNIVLGNLAYVVTHDSGLYSIVPLIALGAAITGSTFVAIDLMRERSFGLLARLWVLPVHRASGLISRILANAIRTLVTTLVMLGTGVVLGFRFRQGLIPSLMWISVPVILGIAIAAMVTTVALYTAQTVVVEGVELVQAIAIFFSTGLVPLNSYPGWIQPFVAHQPVSYAIAAMRGFAMGGPVLSPMIGMLVWTAGICVVCAVPLAIGYRRASTH

>P9WG22_ABC

MSGPAIDASPALTFNQSSASIQQRRLSTGRQMWVLYRRFAAPSLLNGEVLTTVGAPIIFMVGFYIPFAIPWNQFVGGASSGVASNLGQYITPLVTLQAVSFAAIGSGFRAATDSLLGVNRRFQSMPMAPLTPLLARVWVAVDRCFTGLVISLVCGYVIGFRFHRGALYIVGFCLLVIAIGAVLSFAADLVGTVTRNPDAMLPLLSLPILIFGLLSIGLMPLKLFPHWIHPFVRNQPISQFVAALRALAGDTTKTASQVSWPVMAPTLTWLFAFVVILALSSTIVLARRP

>P9WQL7_ABC

MTALNRAVASARVGTEVIRVRGLTFRYPKAAEPAVRGMEFTVGRGEIFGLLGPSGAGKSTTQKLLIGLLRDHGGQATVWDKEPAEWGPDYYERIGVSFELPNHYQKLTGYENLRFFASLYAGATADPMQLLAAVGLADDAHTLVGKYSKGMQMRLPFARSLINDPELLFLDEPTSGLDPVNARKIKDIIVDLKARGRTIFLTTHDMATADELCDRVAFVVDGRIVALDSPTELKIARSRRRVRVEYRGDGGGLETAEFGMDGLADDPAFHSVLRNHHVETIHSREASLDDVFVEVTGRQLT

>Q1C5W7_ABC

MTGPQQGKILLRLENVSREFITGEQTVRVLNNINLTLHSGEMVAIVGTSGSGKSTLMNILGCLDKPSAGEYWVAGRIPQYLGSDALAELRREHFGFIFQRYHLLNDLSARENVEIPAIYAGIDREERRKRAVNLLSRIGLAERLDYRPSQLSGGQQQRVSIARALMNGGDVILADEPTGALDTHSGNEVLNILKDLHQQGHTVVIVTHDMSIAEHAQRIIELKDGEIIADRPRDHAQEKPKMVDIPSVIDIPSMDEKISTGAQQETEIARKPLLTRWKVQYDRLHEAFKMAILAMAAQRLRTALTMLGIIIGIASVVSVVALGKGSQQQVLANINAMGTSTLEIFPGKDFGDMRSAAIHTLRDTDADVLAQQGYIHSVTPTVSTSVTLRYGNKSVSGTVNGVGEQYFLVRGYTIAQGMAFTRTSVNDLMQDAVIDENTRDKLFPNGETPLGKVILLGSLPCRVIGVAAKKQSGFGSDENLNVWIPYTTAMKRMLGQSYLKSITVRVNDDIDLANAEQGVIKLLSQRHGTQDFFVMNTDSIRQTIQATTSTMTLLVSMIAVISLIVGGIGVMNIMLVSVTERTKEIGVRMAVGARASDIMQQFLIEAVLVCLLGGSLGVALSLGIGLLFSLFSSNFSMVYSAASIITAFVCSSLIGVIFGFFPAKRAAEMDPIRALERE

>Q3B5J7_ABC

MAPTTPLLELVDVHRTYPVGESTVNALRGVSLEIREGEFVAIMGSSGSGKSSLLHILGLLDNPDRGEYRILGRNVNALPEDGQAGLRNHVAGFVFQQFHLLKRMSIVDNVRLPHIYSGLKGDFRHEALESLKKVGLMHRLDHTPGQLSGGEQQRVAIARALIGNPMILFADEPTGNLDSRNSLEIMKILEELHREGRTIVMVTHEDEIAAYADRVITMRDGLVVSDQRRDRVCLPAGPSVPLTLDPHAMMDASRNLSVWQDGRFIGFVQQAFQSIFANKVRSLLSVLGILVGVASVIAMMALGEGAKVSIEEELKSMGSNLISVRGGSARVRGAAQGDGAVARFTFKDVKDISRMHSLVKGAAGTVNGSGQIVFGNRNWSTTLDGVGYEYGSMRAFVPSIGRWFTRDEIRKREKVAVIGVTVARELFGNNNPIGHTVKINRINFKVIGIAPAKGFSTHRDQDDVVLVPVTTAMYRVLGRDYLNSIYVEVRSAEGIDGAKEAVSDLIVKNHRLREGDDSFNIRDMTEIQEMLSSTTRTMSMLLGAIAAISLLVGGIGIMNIMLVSVTERTREIGLRKAIGARREDIMLQFLVESVGLTLSGGIIGIIAGIGISALLAVFAGWAVKTSIVSIVLATFFSAITGIFFGLWPARKAAELRPVEALRYE

>Q7ULB5_ABC

MIQLYGLRKDYRVGDHDLPVLKGITLNIEAGEYVALMGSSGSGKTTLMNLLGSLDHPTDGDYHLAGIDVSSLTPLELAAFRSQHIGFVFQNFNLLPRATALDNVMLPTIYASDGRSRRECIEDATKLLESVGLGGRLDHMPNQLSGGERQRIAIARALMNRPKLLLADEPTGNLDTVTEQEILALFRQLNQEHGITLVVVTHDAEVAHEADRVVRMKDGLVAEDVRQRASTVDRSRLANSRAEPLREPASAWSLPATWNAIVVAVLALRRNALRTVLTMLGVIIGVASVISTMELSAGASTAIEETVASMGASMLTISPGKASSTSGRQRPIQIIPDDVVAVAEQCSAVKVAAPLVYSQVQLVRQNRRWSPNLALGTTSQYLAARNWDQLELGTPFTQEQVLDAAKVCILGKTVAHELFDSEYPIGEEIRVNGVPLRVVGVLTEKGGDVIGNDQDDIIIGPWTTFKLRVNSSTGATAQFSTFADQMPPMQLASTRRSTQREEIHQIYVEAESPDHVELARQQITQVLSRRHNVEPAGAYRINDITEVSKVVGQVVGGVSALGLVIAGVSLMVGGVGIMNIMLVSVTERTREIGLRMAVGANRSAILRQFLIEATVLCVVGGFIGIFAGHMWSVLVGRVIGWPTAMSIWAPIVAVTVAATVGIVFGYYPARTASRLNPIDALRYE

>Q7VMF9_ABC

MKQPLIELKNIERYHTNGDTLTTVLKSINLKIYSGEMVAIVGASGSGKSTLMNIIGALDVPNSGEYFIYGRNIADLSGDELAELRCRHFGFVFQRYHLLSHLTAVKNVEVPAIYAMADKILRNQRANALLCQLGLEKQLENKPAQLSGGQQQRVSIARALMNGGDIILADEPTGALDSQSSQDVLKILKDLNRKGHTVILITHDLAIAEHADRVICIQDGKIVSDTANALESMIKPQNKRTFIDDAVIEVCQQHNTEKLNRPNEKNNIDNDNKENNNGYNRNDNSFLNNPKKKLNSSILRSFNSYAESFFMAFNMMMAHKIRTFLTMLGIIIGIIAVVFVIALGEGTKKKVLDEFSSLGNNTIDIFPGKWGDESDNVHTLNMEDLELLYQQPYVQRATPVLLHIAKARYLNKTMRSLINGVSHDFFMLKNYQLVTGRLFDQNDLTLSQPVGVIDKKSAKLLFDMDDPINKIIFIDDIPLSIIGVVESSSLQQNSGKEILIWIPHSTMATRILNQSYIQQISVQLQPNVSPLKSDKAIIDLLTIKHGQKDFYTFSSSRFLQSLNKTTQALTLMISSIAFISLIVGGIGIMNIMLVSVIERTKEIGIRIAVGAKERDIRFQFLIESTMVSLIGGCIGVGCALLFGGLFSLAETSIKIQFTLSSFLIAFLCSSMIGIVFGYFPARNAAKLRPVDALSRE

>Q881Q1_ABC

MNQKVDIEALHETSINSDQPLLRLQQVSRSFMAGDREFQVLKHIDLAIHTGELVAIIGASGSGKSTLMNILGCLDHASAGSYQVNGQETRELDDDALAALRRDHFGFIFQRYHLLPHLDAVRNVEIPAIYAGTAQTTRHERAQALLTRLGLGGHLQHRPSQMSGGQQQRVSIARALMNGGQVILADEPTGALDTASGKEVMRTLLELHAAGHTVILVTHDPKVAANAERIIEVSDGEIISDRRTAQTTQPAPEAQPATPPGPAPRRLLASLGLFREAFNMAWIALISHRMRTLLTMLGIIIGITSVVSISAIGEGAKRYVLKDIQSIGSNTIDIYAGANFGDSRAKSIETLLPSDVAALNQLYYIDSATPVVGRSMLVRYRNVDVDAQLNGVSSRYFQVRNIQLAAGITFSDQDARRQAQVVVLDHNTAQRLFGPGVNPLGQVILVGKLPCTVIGVTSDHKNLFIAGNTLNLWMPYETAAGRVLGQRHLDSISVRVKDGMPSKAVEEQIKALMLQRHGTKDFFTNNLDSVMQTVQKTSRSLTLLLSLIAVISLVVGGIGVMNIMLVSVTERTREIGIRMAVGARQSDIRQQFLVEAVMVCLMGGVIGIGLSYAIGYLFTLFVQQWEMVFSLASVVTAFACSTLIGVLFGFVPARNAARLDPIEALARD

>P9WJB1_ABC

MTRLVPALRLELTLQVRQKFLHAAVFSGLIWLAVLLPMPVSLRPVAEPYVLVGDIAIIGFFFVGGTVFFEKQERTIGAIVSTPLRFWEYLAAKLTVLLAISLFVAVVVATIVHGLGYHLLPLVAGIVLGTLLMLLVGFSSSLPFASVTDWFLAAVIPLAIMLAPPVVHYSGLWPNPVLYLIPTQGPLLLLGAAFDQVSLAPWQVGYAVVYPIVCAAGLCRAAKALFGRYVVQRSGVL

>P9WJB3_ABC

MRAISSLAGPRALAAFGRNDIRGTYRDPLLVMLVIAPVIWTTGVALLTPLFTEMLARRYGFDLVGYYPLILTAFLLLTSIIVAGALAAFLVLDDVDAGTMTALRVTPVPLSVFFGYRAATVMVVTTIYVVATMSCSGILEPGLVSSLIPIGLVAGLSAVVTLLLILAVANNKIQGLAMVRALGMLIAGLPCLPWFISSNWNLAFGVLPPYWAAKAFWVASDHGTWWPYLVGGAVYNLAIVWVLFRRFRAKHA

>P0C068_ABC

MVRRTKEEAQETPAQIIEAAERAFYKRGVARTTLADIAELAGVTRGAIYWHFNNKAELVQALLDSLHETHDHLARASESEDELDPLGCMRKLLLQVFNELVLDARTRRINEILHHKCEFTDDMCEIRQQRQSAVLDCHKGITLALANAVRRGQLPGELDVERAAVAMFAYVDGLIGRWLLLPDSVDLLGDVEKWVDTGLDMLRLSPALRK

1. **Multidrug and toxic compound extrusion (MATE) : 23 protein sequences**

>1638.4.peg.962_MATE

MKQTDEFYLTKASIPKAIAHLSIPMMLGMSVGVIYNIINAFFIGMLHDTSMLTAVTLGLPMFTILMAIGNMFGVGGGAYISRLLGKKENSQAKQVSAFVLYGSLALGIICASILGLMINPVTHFLGADAASFLHTRNYTLALLICSPFIIANFALEQVVRAEGASKISMNGMFISTIVNLIFDPLLILYFDFNVVGAAVSVGLASAFSLVYYAWYLEKKSAYLSIHFKWFRVTKGTISNVFKIGVSELLLSLFLIVTTLILNYYSISYGEGVVAGFGVALRVVQLPEFICMGLYMGIIPLLAYNYSAGNIARFEKAIRFTAISIGLIVLVISSLVFLFRFQVMHLFSESPSVIMLGVHIMVAMLISSLFSGFTGLFTSTFQAIGKAIPATIMSVSQGIIFIPVIMLGQYYFGLVGVIWSLTATEILTCIIGVTLFTIYNIKIASSTKAKDLAV

>182217.3.peg.1605_MATE

MEKVFKRIGAYSMLKAKIDLHKDSIRKLFFYYFIPLAFSMISLSTYSMIDGMFVGKKLGKEAIAAVNIAWPIFPSLVAYELLFGFGAASIVGYFLGRGKTHRAKLVFSSVFYFVALSTFILSMALLPFSETIARLFGSNDALLAMSSRYIEIILMGAVFMVLHPLADVFVVNDKRPILAMVAMLIGSLTNVFFNYLFIFVLEVGVQGSAYATIIGHGVGFLVLMQHFLFKKGQLSFIKRFSFPAVISSAKSGVPQSTAELSFALMILIFNATIMHTAGERFLSMYGIIMYNAIIFWTTLFSISQGIQPIASFSYGARNLERVKGVFLFGLKVAFLVGVVLYGIYYFLDEFLIKMYLQANEQDLDFIQETKQAMNVYYLGYIFLGMSILCAVFFQSIQCTRSSFIITLSHTLIFIVVLLPLMSHFYGIKGIWATYPIAQFLAFLSAMGVTYYEIKKGVFTTYREQSLINGAKK

>272563.8.peg.1578_MATE

MENLFTRKFTTFEFLKFVSPAIISMIFISLYTIIDGIFVSTLVGSDALASINIVLPIINLVCGFGIMMATGGGAIVSIRMGENRQDEANSTFSFIVLFSLIVGILFTVISYFFIKEISILLGATDKLLPYCITYGKVMILCTPFYILKFIFEYFARTDGNSKFSLFLSVIGGVTNIILDYVFIKYFGMGLLGAAVATAIGIILTCVLGIIYFLSNKSTLKLRKPKTDFRLIRDTMINGSSEMVTELSTGITTFLFNVVALKLAGENGLAALTIVLYAHFLMTSVYLGFAAGVSPLISYNFGAENSDKLKETFKHSLKFIFISSLLVFIIALVFAPFIVRVFVNPDNTVFKLALQGLKIFAFAFLFVGINIFASGFFTAFHNGKISAIISFSRAFVFIIIGIIILPPMLNMTGLWLTVPFAEVITIFISILFIKKYKGRYKY

>1028805.3.peg.567_MATE

MNFRLLSQYHADIKKLIKISLPILLAQIAQNSMGLADTIMAGRVSSTDMAAISVGASIWMPLVLFGQGLLLALPPTISYLNGSGQRHRIAHQVRQGIWLVLGMSIPLGLLIYFCEIPLQYMQMESKMSDLARDYLHAMLWGLPAYLMLINFRCLNDGIAKTKPAMVITFLGLLLNIPLNYIFIYGKFGMPAFGAVGCGIATSIVNWAMCLMMMFYSYTNAQERSLKVFSQLIEMPNPKTLKKLLRLGLPIAIALCCEVALFALTSLMLSPLGSTIVASHQITLNTSSFIFMFPLSIGMATTILVGQALGAGSPQNAKKMSYAALLLGLTVTIITALITIFFRYEIASIFVTDEIVIAMAANLLLFAALYQFSDTVQMVVGGILRGYKDTKVILYITLFSYWVIGVPLGYTLGRTDWLVPHIDAKGFWIAFVVSLTFAAILLALRMKKMQAMSDNAILQRLEKLK

>Q4L8N9_MATE

MKDEQLFYFEESSIFKAMMHFSLPMMIGSLLSVIYGILNIYFIGFLDNSHMISAISLTLPIFAVLMAFGNLFGVGGGTYISRLLGAKDYIKSHYVSSFSIYSSLVLGLIIAVITLPFTDQIASILCASGETLNYTSDYLKIEFLSTPFVILFFVLEQFARAIGKPIISMIGMLSSVGINIILDPILIFGLHLDVVGAALGTAISNAIAGLFFIIYFSRKNETLSFNVKHAKPTKAMMQEIFKIGIPAFLMVVLMGVTGLVVNLFLATYGNYAIASYGISFRLVQFPELIIMGLSEGVVPLIAYNFVSNKTRMKDTIKVVIVSIAVIFAVCMTVVLVAGHSIVQLFSTDPQIVVLATFILKVTMTSLLLNGIGFLFTGMLQATGQGRGATIMAIAQGTVIIPVLFVLNSLFGLTGVIWSLLIAETVCAFLAMFIVYSLRNRLTVDKASLIEVE

>Q5MZD9_MATE

MNLRTIRAELQQFLQLAIPLAAAQVAQAAVGFVDTVMMGRLGPEPLAAGGLASALFQFILATASGVVMAVSPLVAEAQGAGKDYKIAAIARQGLWLSVLLGLPVMLIISQLARLMPVLGQSATTIALARDYWMAVLWGIIPGLGFAMLRGYVAALEQARIILPLVLFGTLVNGLGNYLLGYGQLGFPRLELTGLGLSSALGLWVMFLGLLAYTAWQPKLRRYPFWQDWRRLQPSICRQILQLGWAIAVTVAVEFGLFTIITILMGAIGVEALAAHQTVSQTIILIFMVPLGCSFAVTVRVGWWLGRQDGLGARRAGLVGVGAIALWMLLLAIPLALFPRAIVGIYVDLNNPVNAGLLNLALPMLRVASLALVLDGVQRVAMGALHGLQDTRIPLLLSLLAFWMVGVGSSAMLGFQLGWGSTGLWIGQSLGVAIAGGLFLQRFLKLTQNRTFKQRLQPQPLATHP

>Q5NYX9_MATE

MSAPILFPLSAPESSFTIAGRLFHHAWPVLVAQLLSMSMLIADTVITGRYGTLDLAAVAVGSGVYISIVMLLVGVLQAVAPTVAHHFGARRVDAIGPALQQGFWLALMLALPGIALLAFPGFLLELSSVPADVAGKTRDYLLATAFGLPAVLLYRTFYAFNNALGRPRALMMISFIVTSTHIPLAWALVHGAFGLPPLGAIGCGISTAIVNWIAFACGAGYLAHNRDYRPYRLFANWQPPRRRDLLALLKLGIPMGLSTFIEVSSFTLIALFAARLGAEAVAGHRVVANLAALIYMLPLAISIAILVLVGQAAGAREPARARATVRVGMGLTVGLVALIGVLLWVGREPVVALFSADPAVRAVALGLVFYICIYQIFDAVQTVAAHALRGYKVTFMPMLLHALCFWGIALAGGYWLAFHAPGREQSPTVAGFWEASVVATILASVLFGWLLRVVMRRPQNVQT

>Q62LW6_MATE

MSPTGFTRAAAAPPPTLSRHAADTARLAAPLAIAQLSQMAMSVTDTVLLGSLGPDALAAGGLGANLFFVVVTLLQGVLTSVSVSVAHARGAMAEDRVPHIYWTGFALSLLLAVPAFALLSFAQPLLLAFGEPAALARNVGEYAAVLRFAAPGSLIGVGLMRSFLPAIGAAKRLLWVSLAGVGVNAFLNYGLIHGAFGLPRLGFLGSATATTITIWLTAITLVALLHGRSTFRHFVAATRPRLPLMGELFGIGWPVAITYGVESTLFLATGLTVGVLGESSLAAHQIALNVASVAFMVPLAIGQAANVRVGYWAGAGAPVAARHAGFVALGLGVAFMSLSGLVLIVAPHAIVGLYLKLDDPANARTVVLATSLLGIAAVFQIVDGMQTVGSGCLRGLKDTRVPMLAATLGYWGIGFPTGYWFAFHAGLGARGLWWGLAAGLASVAMLMTWRFHRKSAALGVRADARGQA

>Q6FEY7_MATE

MAKVAGFRFELKQLFHLMWPILITQFAQAGLGLIDTIMAGHLSANDLAAIAVGVGLWMPVMLLFSAIMIATTPLVAEAKGARTPEHIPVIVRQSLWVAVSLGVIAMLILQLMPFLLPILGVPESLQPKAGLFLHAIGFGMPAVTMYAALRGYSEALGYPRPVTVISLLALVVLVPLNYIFMYGIGPVPHLGSAGCGFATAILQWLMLITLASYIYRAKAYQSTQVFSHWERINLTLVKRILKLGLPIGLAVFFEVSIFSTGAIVLSPLGDTLVAAHQIAMSVTSQLFMIPMSLAIALTIRVGMYYGEKNWVSMRLVQKLGLATATFFAMCTMSLIWFARPQIVAIYTQDPAVFDIALYLLLFAMAYQLMDAWQVGAAGCLRGMQDTKGPMWITLIAYWVVAFPVGTYLARVAKMGPAGVWLGLITGLSIACVLLLMRLYRNNHKLAQQS

>Q6NB79_MATE

MVRAMTAPGSNIAAGALAPAKSSAWRTELIETLWLAWPMALTQLGQIAMMTTDLALIGRLGDAAVAAAALAHFVLFSTFTMGLGLVSAVTPLAAQAFGARAPRQVRASLRVGLWAGVIAGVPLTLGQLYGEELLVALGQNPATSRLAGDYLDGLAWSLVPGWLFIALRGLMGAVNRPEPALWIMLTAIPINLGLAYVLIHGSFGLPRLEIFGAGLATSIVSWAMCIAAAVVCVTMRPFRKYQVFGELFRFDGELMRRLLQLGLPISGASVLEYGVFGAAALLMGKFGTTALAAHQIALQVAAIMFMVPMGISVAATVRVGHAVGRGDPPSARRAGFAAIGLGFVFMAAMTLLVALTRHQIPQLFLGDSDTSIETATLTAALLIVGASFFIADGLQVVANGALRGRNDTKVPLLFAVLGFWVIGFPFCWVLGFHTDLGPFGVWIGLAVGLVVYAALLVWRFHRLTRDAMAAAVAA

>Q7N1G0_MATE

MAKFSNWRELKQLLFFSFPIIVSQIARTAMSFVDIVMSGHYATADLAAVTLGSSIWFPIFVLGYGTIIMLAADVAKQKAQHDDEGIKDSLKNYLFLAVILSIPIIILLMLVSWLLSFIGIDEHILEITQGYVIALACGVPSVMIFNVFRSFLQGLEDTKIAMYLSAGALLLNIPLNYILIYGKLGLPEMGGIGAGITTAIINNLIAVCLIIYFLLKKEYRRYRPDFSLPKYNSLIRTFYIGMPSGLALFVEMVFLDVIAITAAPLGAQVIAAHNIMLNITSIIYTITGGIAAAVTVRVGSYIGKRDKISLTGTIKISIALILSISAVIGVLIYYFAGSFISLYTNDNGVIIIALNIIFLLCLFQFFDSCQAALSGILRGFHDTRSVFYAPLFGYWLVGLPLGFILALTDWVTERMGIIGFWYGLVLGLFVNAILLFIILKVRQRGMISRLISY

>Q879Z5_MATE

MFLPRPDFRIALSICFMAVSFVISRFGSEVRPTLLLALPLVLGHVSTGLIGFVLNVIAGHHSTVTLAASTIGTALLWLPMLVPMGTLISLTVLVSQLHGAERERDIGPLFRQALWLAMLLGLVMFTFLSVVPALLPLFGIVPDIVPGAAKFLHVVRWGSLAFPLYFCMRYFCEGMHCTFPTMLLGFGGLLVLVPLSYALTYGRFGFAEYGVEGLGIATVTVMWLQAVVFALYLWRSRRFAHLQLFAHLELPCWARIRDLLNIGLPIGISILMEGGLFIVTTLLIGRFGTDEIAAHQIALSVAQLCFMIPMGVAEATTVRIGHAVGRCDLLVMRRVAWAGYAIVIGTQTLSASVLLLGYDVIVAAYTDDLVVASLASKLLLFAAIFQFPDGLQMLSSGVLRGMKDTRVPMLLAMISYWGLGMPLGLGLGFALEWNSRGMWIGLIIGLTAAALLLGWRFRVVSERMFAGIP

>Q89AX2_MATE

MKKHLHEIKMLLKITIPIFLAQISQTSMSLINSIMIGHLKENNIAAISVGISIWSPIILFGHGLLLSLVPTVSRIHGSGKINKIPEQINNAYWLATLISLVIMIVLWNSDVIIHTISQVNPIIEQESIKYIRILLWSTPGYLYFQVIQNQCEGLLKPKPAMVIGLIGLLFNIVVSYTLISEKFHCFNYGSTGCGISAIIVYWFMFIAMKKITKNDILINYNIKNKNISNLEMYLPNYKIIWNLFKMGFPIALSLFCEITLFTLITLLIASMETFQIIAHQIALNISSTIFILPLSIATAASIRLGFYLGKKSFSKISTIILSSQIIGLIISTTISTFIILFHYQIITLYTKNANIIKLTKQMLFITASYQIFDFFQIIGNGILRSYKDTNIIFIITCTSYWIVGFPFGYFLALTNYIVPHMGAIGFWYGILIALITSSIMILFRIYILQKK

>Q8G2I1_MATE

MDGTFDAGFREPTISKANRWGREMVVALKLGWPLIFTNLSQAALTATDVIFIGRLGADTLASALLATSFYHTLMIFSMGLVSAVMPMIAIALGKNRHSVRDVRRTVRQGFWSAIMIVIPLWVVLWHCEEIFLFLGQRPDIAARSTDFMHTLQWALLPYLFYIVLRSFFAAMEKPMWTLLVAALAIGFNALAGWTLIFGHFGFAPMGLHGAGMATTASSTMMFLGLAFITLRHPRFRRYHLFGRFWRPDWPRLIELWRIGLPMALTFVFETSIFYAAVVMMGRIGPTAMAAHAVAIQIASLSFMVPLGFGQVATVRVGRAYGRGDPKAIAYAGWSAYALGVGFMALMGILMVLMPRVFIGIFLNLNDPQNLPVMELAVTFLALAALFQIVDGAQAVAAGMLRGLRDTRIPMLLALFGYWGVGLPLGAVLAFQFGMGGVGIWLGLAAGLGMVAVLMTIRWRRHLAHVSAVAAA

>Q8UDF5_MATE

MSSSVVAETVPSGSGSWFSHFKATLVLGIPLIGAQLAQLGIHTTDMVIVGQLGAEKLAAMVLAGQFFFVVFIFGSGFSVAVVPMVAQAYGQGDATSARRSLRMGMWVAIAYWLLALPIFFNAERILVYLGQNPNVAALTGHYLAIAKFGLLPALLFYVLRGLVSAIGRAGIILYVTIIMLVMNGLMAYVLVFGHFGLPAMGMNGAAVVAVIVNAFSFIFIVAYVQTREETKKYELFVRFWRPDWHALFEVLRLGLPISITILAEVTLFAAASILMGQIGTVQLAAHGIALQLASIAFMIPLGLSQAATVRVGVARGQGDFKNLIRASIMIYAIACGIALCGGILFAAVPEFLAKWFLDPKLPEAAEVLAYASSLVVIAGIFQLVDGIQAVTAGLLRGLKDARIPAMLALISYWPIGLALAWTMAFPLGFGGRGVWFGFVIGLSTAAVLLTVRFVLLVKREMKTAR

>Q9HTR0_MATE

MSSPSLVELKAILRLAGPLIAAQLAYVAMVFTDTVMMGKLGPDALAAGGLGAVSYAFVSTFCVGVVAAVGNLVAIRHGCDDAAGAAAAARSGLWVGAALALAAGLLLWNLRPLLLVFGQAPQTVDGAMQFLHSLTFALPGYMAFMVLRGFTSAIDRAGPVMAISVLGALANLALNYSFIEGLFGLPRLGLAGIGLVTALVMNCMPLLLALYIRLQPAYAEYSLLRGLGRPQRAMVEEILRLGLPIGGTYAVESGMFTVATLCMGIIGDHALAAHQIAIQAVYVAFMVPVGLSYATTYRIGQHFGAGRLLEARRAGRVGIGFGALCMLLFAGLFWWMPEAIIGLFLDRDAPANREVAAMAVSLLAIAAWFELFDGTQNVAMGAIRGLKDARTTFLVGLACYWLVGVPLACLLAFAAGWGAAGVWWGLAGGLACAAIGLTLAFEWKTARLLPKATASEASALNCRAAGRGAPSARLCPGNAPVPPTAAAD

>Q9JV27_MATE

MLLDLNRFSFSVFLKEVRLLTALALPMLLAQVAQVGIGFVDTVMAGGAGKEDLAAVALGSSAFATVYITFMGIMAALNPMIAQLYGAGKTDEVGETGRQGIWFGLFLGVFGMVLMWAAITPFRNWLTLSDYVEGTMAQYMLFTSLAMPAAMVHRALHAYASSLNRPRLIMLVSFAAFVLNVPLNYIFVYGKFGMPALGGAGCGLATMAVFWFSALALWIYIAKENFFRPFGLTAKFGKPDWAVFKQIWKIGAPIGLSYFLEASAFSFIVFLIAPFGEDYVAAQQVGISLSGILYMIPQSVGSAGTVRIGFSLGRREFSRARYISGVSLVSGWMLAVITVLSLVLFRSPLVSMYNNDPAVLSIAATVLLFAGLFQPADFTQCIASYALRGYKVTKVPMFIHAAAFWGCGLLPGYLLAYRFDMGIYGFWTALIASLTIAAIALVWCLELCSREMVRSHKAV

>Q9KEJ2_MATE

MKPTETLQEKGKLFLVVMMPILITQIGLYAMNFFDTVMSGQAGANDLAGVAIGSSLWVPVFTGLNGVLLALTPIIAQSIGAEKRDDVPYVFLQGLYLSIAISIAVILIGAVVLDPILSAMSLEDEVGRIAKEYLIGLAFGIVPLFIYTTIRCLIDSLGETRVTMFITLLSLPINIFFNYVLIFGKLGFPRLGGVGAGYASAITYWFILAVAIVVVVKVRPFTDFQLFKKLYHVSLKKWKEILLLGLPIGFTIFFETSIFAAVTLLMSTFDTATIAAHQAAVNFASFLYMIPLSIAFTLTIAVGYEVGAKRVEDARQYSRLGITFALIMGLVAGVIIYVLRAPVASLYTNDSQVAWLIQQFLIYSIFFQLSDALATPIQGVLRGHKDVNVPFVMALVSFWIIGLPTGYLLANFSPLGPYGYWIGLITGLASCAIALSWRLKQMQRKFERAARLSQNGNS

>Q9RY44_MATE

MTTLPAPTISTTAELRALLRLAGPVVVSQFAANALALIATAVIGRLGERELAAAAYANAAYYLVFIMVVGVMLSVAPRVAQAHGAGDARGVARALGGGLRLALLLSAVMLPLMWALSFVLPNFAPAGVSRDLVAAYLRVYSLGMLPNLAFIALRGTLEGTGKPGAVTGVALTGVVWALLVAPALAFGWGPLPRLGLAGAAGASASAAWIMAALLWPLARRRVAYAGPLGPLGDEVRALFRLGWPIGLTLGAEGGMFSVTTLLMARFGPEVLAAHNVTMQTITAFFMVPLGIASATGVRVGTEAGAGRLAQARRAGLVGLGLSSAVMLTFAVIELAAPRTVFSVFVNVNDPANAGLIAAATGFLSIAALFQLMDGLQVTANGALRGLQDTRVPLLVSLVAYWVVGLGLGSVLSSVAGLGARGLWFGLTAGLTLAGLSLVGRFLYRTRAGRAA

>Q9WZS2_MATE

MRYSLFKNYLPKEEVPEIRKELIKLALPAMGENVLQMLFGMADTAFLGHYSWKAMSGVGLSNQVFWVVQVVLIAASMGATVTIANAIGAGNRKAVRSLAWNSVFLAIFTGVILTALTPLSDVLINIFPNLEGEIESSAKEYLKVILSGSMGFSIMAVFSAMLRGAGDTRTPMIVTGLTNFLNIFLDYAMIFGKFGFPEMGVRGAAVATILSRFVGAGILTYVIFKREEFQLRKGLVPPKWSSQKEILRVGFPTAIENFVFSTGVLMFANILLIAGAEAYAGHRIGINVESLSFMPAFGISVAITTLVGRYNGMGNKEHVLGVIRQGWILSLLFQVTVGIIIFLFPEPLIRIFTSDPQIIEISKLPVKIIGLFQFFLAIDSTMNGALRGTGNTLPPMIITFISIWTARLPVAFVMVKYFQLGLLGAWIGMIADIIFRSTLKLLFFLSGKWEKRAVLTRERVKELG

>P58163_MATE

MTVVTTMPRDAAGTALLPERPRGPIMTDLIELLRLAGPVVLSRLGIMVMGLTDAIVVGHFSAQQLGYHAMAWAPSSVFVTATVGLLVGVQVMTARAMGAGNPHETGAVLRRGLVYAGWLGFGSMALLALFGPMFLQAMGLKDGLAEGATLPLIVFSLSLPVYAISVVLTFWLEGLSRPGPGAAMMWLANVVNLGANLLLVPGVLGPPALGAVGGAWATFIARTALALALAIFVIRMKEARELGVFDKPARDRPAEIEQRRIGYGAGASNFFEVSAFAGMNLICGWISAVAVAAYTVVLNVSAIIFMVPLGVASATAVMVGRAYGARDPAGMTRAGWIAFAVIGVIGVLFGLLLYPTKHWVALAYTTDPAALALILPALVLACLFFAPDAVQVVAAQALRARGEVWVPTITHLISYALVMGPLAWWLAIPKGMGLNGVLVSIIVTSFLAAGFLLMRFRMLDWRDRKAAQEAA

>P45272_MATE

MNFRLLSQYHADIKKLIKISLPILLAQIAQNSMGLADTIMAGRVSSTDMAAISIGASIWMPLMFFGQGLLLALPPTISYLNGSGQHHRIAHQVRQGIWLVLGVSIPLGLLIYFCEIPLQYMQMESKMSDLARDYLHAMLWGLPAYLMLINFRCLNDGIEKTKPAMVITFLGLLINIPLNYIFIYGKFGMPAFGAVGCGIATAIVNWAMCLMMIFYSYTNTQERSLKVFSQLIEMPNPKTLKKLLRLGLPIAIAICCEVALYALTSLMLSPLGATIVASHQITLNTSSFIFMFPMSIGMATTILVGQALGAGSPQNAKKIGYAALLLGLTVTIVTALITIFFRYEIASIFVTDEIVIAMAANLLLFAALYQFSDTIQMVVGGILRGYKDTKVILYITLFSYWVIGVPLGYTLGRTDWLVPHIDAKGFWIAFVVSLTFAAFLLSLRMKKMQAMNDNAILQRLEKLK

>Q9I3Y3_MATE

MNSPALPLSRGLRIRAELKELLTLAAPIMIAQLATTAMGFVDAVMAGRASPHDLAAVALGNSIWIPMFLLMTGTLLATTAKVAQRHGAGDQPGTGPLVRQALWLALLIGPLSGAVLWWLSEPILGLMKVRPELIGPSLLYLKGIALGFPAAALYHVLRCYTNGLGRTRPSMVLGIGGLLLNIPINYALIYGHFGMPKMGGPGCGWATGSVMWFMFLGMLFWVNKASIYRASQLFSRWEWPDRATIGPLVAVGLPIGIAVFAESSIFSVIALLIGGLDENVVAGHQIALNFSALVFMIPYSLGMAVTVRVGHNLGAGLPRDARFAAGVGMAAALGYACVSASLMLLLREQIAAMYSPDPAVIAIAASLIVFSALFQFSDALQVTAAGALRGYQDTRVTMIMTLFAYWGIGLPVGYSLGLTDWFQEPTGPRGLWQGLVVGLTGAAIMLCIRLARSARRFIRQHERLQREDAEAASVLGR

1. **Major facilitator superfamily (MFS): 95 protein sequences**

>757424.7.peg.2755_MFS

MSDSTTQQQPQANNNGNGKRKRQLILLTLVLLVIAVACFLYWFLHARFFEETDDAYVGGNVVQISAQVGGTVVAVKADDTQVVKAGQQLVALDAADTRLALDQAQAALAQAVRQTRQLFLNNDTLAANVAAADSNLARAREDLQRRQAGLSSGAVSQEDVSHARDAVKSAVAALDQARAAAAANRALTDHTSVTEHPNVLQAATAVRNAYLNYARVNIVAPVSGFVSKRSVQVGQRIAAGNPLMAIVPLEQIWIDANFKESQLQHIRIGQPVEVIADVYGSSVKYKGTVIGFSAGTGGAFSLLPAQNATGNWIKVVQRVPVRIALDPEQVRAHPLRIGLSTTATVDIHGDGRALEAVPTNYQTNVYDDLGKQADAIVDRIISDNASGLPQAHKSKAAAAPVAVPHT

>757424.7.peg.3311_MFS

MIIMSHSDSQAAHTLAEEAKQRNEAASAAAAAQKRKKLFSIFGGVVAIAAIGYGAYWYLIGSRYVETDNAYTATEIATVTPAINGIVAAVDVVDTQAVKKGDVLVRIDDADARLAVDQAAADLDRTERKVKGFFANDAGLAAQVLAREAEQKRASAQLLSAQADLKRAEIDLQRREALAKSGSVSGEELSNARTALLTAQANLKAAEAAEVQSRANIKATQGAQKASTVLTANTTVDDNPEVVLARAKLEQAKLDLERTVLRAPVDGVIARRQVQVGQRVQSGATLLSVVPLQQMHVDANFKEGQLTKVRIGQPVTMKADLYGGSVEYHGVVTGLSGGTGSAFAVIPAQNATGNWIKVVQRLPVRISLDPKELAQRPLSVGLSMVVEIDTRGQIQAGDAQRKSARNDNAQAAAL

>1006000.3.peg.2818_MFS

MIKSSPHCGRFLSLLAQFLDIYSYKPNFYKVISEMKRDRNVNILVMLVLLVAVGQMAQTIYIPAIAQMANDLNVREGAVQSVMAAYLLTYGVSQLFYGPLSDRVGRRPVILVGMSIFMLATLVAITTHSLTVLIIASAMQGMGTGVGGVMARTLPRDLYEGSQLRHANSLLNMGILVSPLLAPLIGGVLETLINWRACYGFLLVLCAGVTFSMARWMPETRPTGAPKTRLISNYKTLFGNSGFNCYLLMLIGGLAGIAVFEASSGVLMGGVLGLSSMTVSILFILPIPAAFFGAWFAGRPKKRFPTLMWQSVVCCLAAGVMMWIPGLLGVMNIWTLLVPAALFFFGAGMLFPLATSGAMEPFPFLAGTAGALVGGLQNIGSGVLAWLSAMMPQTGQASIGLLMTLMGLLIFLCWLPLASRFAHQGQAV

>1007096.3.peg.27_MFS

MNRIKNWKKQFVVIYTGQAFSILGSAAVQFAVIWWLTIQTESAITLTIASLVAFLPNMLIGPFAGVWIDRYNRRTVMILADGLVAVSSIILGAAFLLVETPPIWFIYIVLFLRGLGNTFHGPAMQAAIPMFVPADMLTKAGGWGNMIQSISNMMGPVLGAALMSFLPISSIMIVDILGAAFAIVCLLFVIIPDIPQTNEKMSVLSDMKQGFIAMKANKPLMAVFFPMLLMTILYMPLGSLFPLLVRSHFMGEAWHNSIVEFVFATGLLLSSLVIGVWGGMKRRFFMASLAIGLMGLATLISGALPTSGFWIFAICCFFLGASGTFMNVPVMAYVQESIAPEMMGKVFSLLMTAMTLSMPIGLLVAGPVVEVIGVNTWFFWSGVVLMADAILCRLLTRRYDKETMRPQAD

>1041522.3.peg.1113_MFS

MTALNDAERAVQNQASARPDRPAPVSSAFPAETASKPPAETALKRISKYYPAWLPSRRFIAAVIAIGGMQLLATMDSTVAIVALPKIQNELSLSDAGRSWVITAYVLTFGGLMLLGGRLGDTIGRKRTFIVGVALFTISSVLCAVAWDEATMVIARLSQGVGSAIASPTGLALVATTFPKGPARNFATAVFAAMTAVGSVMGLVVGGALTEVSWRLAFLVNVPIGLVMMYLARTALRETNRERMKLDATGALLATLACTAAVFAFSMGPEKGWVSITTIGSGVVALGAGLAFIIVERTAENPVVPFDLFRDRNRLVTFTAIFLAGGLMFSLTVCIGLYVQDILGYSALRAGVGFIPFVIAMGIGLGVSSQLVSRFSPRVLTIGGGIMLFWAMLFGWAFMHRGAAYFPNLVLPIVVGGIGIGMAVVPLTLSAIAGVGFDQIGPVSAVTLMLQSLGGPLVLAVIQAVITSRTLYMGGTTGPVKFMNDAQLAALDNGYTYGLLWLAGVAVIVGGAALLIGYTPDQVAHAQEVKEAMDAGEL

>1042163.3.peg.2341_MFS

MKKDGREIRSNAMADLEADRPSHSFQNVKTILLWLSFLAFFSVFNETVFNVSLPDIAQQYGLQPAYVNWINTSFMIAFAIGSAVYGKISDTYGVKKLLVIGLLIYSGGSLFGILAQAYFPAVLVARAIQGAGASAVPAIFMVIVVKYINAESRGKAFGMIGSMVAFGEGIGPAIGGMISHHFHWSLLFVLPIITLISLPFFIRVLPNEPARKGKVDIFGAALLSIGIVLFTLYATGDNWFYLLFSLVVLLIFSLYIRRAKQPFIEPALFQNRMFVMGVLAGSILLGTVAGFISMVPYMMRDVYHLSTGMIGGGILFPGTLSVIFFGIMGGSLVDKRGNTFVMYLGAFLIVLSFLVISLFVEKSPWITSIMLIMTFGGLSFVKTVISSSVADTLASEEAGAGMGMLNLSCFLSEGIGVAIVGGLLSKHVLDFPILPTLSVPTAFLYSNVSLVLIVAIMLGVAIYTWTYKRKERF

>1089544.3.peg.284_MFS

MTTVAAPIDRATWRICWVIVFGAFASGLDASVVTIGLDSISRDLHADLSVTQWVASGYLLALALSLPLTGWLSRRFGAGRVWLVALAAFTVASGLCALAPEVGLLIVFRLLQGLAGGMLIPAGQTVLGQQVGAARLGRVMATLGIAVSVAPALGPLVGGVLLQSLSWPWLFAINLPIGAIGLALGLRYVPRGTPTETHRIDFAGLALVAAGLPLALFAVTSWGESGQLPWPILLPALGLLAWFVLRCRRHPHPLLDFSLYRNRLYRAASLAAAFNGALIFGSGIVVTLYFQIGRQLSFVGTGLSLLGFAGATAAAAPFTGRAVDRYGTAPVALAGAVLAVASTMPFAFLPANAPMAVVQLLLAGYGASVALVSMPMGIAAYKTVSPAKLPDAAAQVTILLRLGGSLGGAAFTVLIANHLPDVAAAFRLGFLAVSVGAGGALAAAWLVARAARNPGRAETMAG

>1095552.3.peg.572_MFS

MSIVGKKAQPGLAASPRDRLKVVRAASQGALSGGQFFLLNFALLLGNVLVLFNTGAFASISLHATGGLGVSPSHASWMQTYYFISMAIALPVSSWMAARFGRVRLFIVAMMLMALGSLLCSVADELVWFLLGRVLQGFFGGLTIPLSQTLLLNEYPEPKKAFAVALWSMAALSPFTLGPAAGGWIADALGWRWLFYLNFPLALVSAALVWALLFDRTSNRTDKPFDRMGFLLLAVALGCLQTALNQGQDADWYNSGLIVSLALIGLLALAGFIIWELAERHPLLDIRLLTRRNFAIGSIVLSVSFLLMYGLLSILLVRLQSVAGYTSFMVGSVLLPLIFLAKPMAVFFHRIVHYFDARWLAGLNLAAFAAFCFWTSTYDFFRRNSLFSDTLGSQVLEGFCLGGLFVPLTTLFLSGLTPRRQNQAVELGGLLRVLGGSIASPLLGVIWERRAAFHQSRLIETLTPYDIVGRETIASLNAADMPGQIATARLAELAGGHAAILGLNDTFRIAAWIFLALAALVWFAHPAGPTRRLLPRQAVRKTALEALVEEP

>1104996.3.peg.2243_MFS

MAEGDFIPRDRVITMVREIMLFKTLPTQTIIGLCALFACTFTALTSEVAPVGLLIDMAQAFHIAEGQAGLAVSAFALMVALGAVPLTILTVAVDRKKLMLLSLGGYILSNLIVALAPTFLILCAGRAVGGVAHALLMSIVSAYAARLAPANMTGRAISFVYGGTSLGAILGVPGAAAIGHFASWRIAMFVMTGLAVLLAICIAFFLPPVAPTGTGSAQLPSIGSRKAMRVFLVVVAIDALFFVAHNLLYTYVTPLLLLHGLPKAVLSLALLLTGVVSISGLWAAGQVVDRWPAAGLLGGGLAMLVGMGLMSGHIVTGWVAVASVGLWCTGYSAIIPFVMSGAIRARATRPDVAGAAINGASNLGILLGSALGGQILTWSGFNILTPLAVGVALAAILLAVFSPDAFPRILHPHEDEASS

>1120960.3.peg.3781_MFS

MSTPRALRPLRNPAYRWLAAALVASMVGSGIWMVALVWQIVAIGGGAAELSLVAGASAVGMLLTTLLGGALADRIPQKRILLVVEVVRAASVGVVALLSLTGGLAAWQLAAVAFVGGVMAGLYYPAYSALLPSVLPEDELLAANGFEGMARPILMQAGGPALASGLIAISSPGAALAVAALTGVVAAVCILRLPETSVRGAEASTGDTGYTGAASDTDVAVDTAPRHPALALLVDVRDGFTYMVRTPWLFGTLVFASLLILLIMGPFEVLVPFVIKDVAGGGPDDHALILAAFGIGGAAGSMAVASLELPKRYLTVMNLLWAFGCLPLAVFGLTDQIWVMAIAAFLVGAAFNGGVVIWGTLLQRRVPPHMLGRVSSLDFFVSLAFMPVSMAFAGPAGEAVGLPTVFLIAGAAPLLIGVVAIFAARMRRDEIAHPLDAVDEPTDVADTADVPDAAAVAEITSDDTDAAELSRSSAREFAASVA

>1121096.3.peg.137_MFS

MNHWKSTLAVIGIGQLISILTSTIVGFSIIFWISNEFKSPTALSLAILAGFLPQFVLGLFTGVYVDRWNRKKTMFYSDLFIAFCTLCLFIVITKGYKDLSFFYLLTACRSIGSTFHAPALQASIPLLVPKHHLVRVSGLYHSIQSFSEVIAPVVGASLVVWLPIQYILLIDVIGAVAACLTLLCVQIPSLQKTKVLPDFKKELTECWHTLRRTMGILPLFVCFTLVTFVLMPVFTLFPFMTLLHFNGNILQMGVVEMGWGSGALLGGLVLACKALKSKQTLVMHTAYVILGLYLISASYLPSSAFIGFVCLTFTGGIAYSIYHALFIAIIQQNLASDMLGRTFSLIFSLSTFPSMLGIVASGYWVEAWGITSVFMISGWVIFLIGVGANFISSIKQLDNYA

>1122999.3.peg.2079_MFS

MPHVFLPERDDLSLEARVLWDARVANDGEITNMKRTLLHAPVAYDALMTWFPLRDALLPRIGERGVIVFSHAISTTNDCLLCSLYFRRTLLARGEDPEARYDLNAEEADLAEFGRSLAADGRASDELTGRLRERYGEDGLVELVAFAGLMAATNLVNTALGIDLDSELLALHTAGVRE

>1123032.3.peg.1828_MFS

MPETNDKITGKIIFITIALAMGSFLNMLNASIVNVSLTHIAGDFGMATSKSTWIITSYSVAEAIVLPLIGWLTLQFGTVKQYIWSTILFAIASLLCGLSFSLSSMVAARLLQGVVGASMIPLSQTLIMKIFPKKKQGIGIAIWTMTLILGPILGPVIGGAITDVASWRWCFYFSIPLCFLSSGVIYYMFKKDYASEKFIRVKTDVVGIFLLISGIGSLQVFLEQGTDLDWFASPSIVVLAVISFMSLVILGIWEWYHENPVINVRLFLNKNFTIGVFSLLIVSAAFYMTAVILPFWLQNVMGYTSFISGKTTATLGLPILLLSPIIGKYTDRIDNRYITITGFIIFTIVTVFTANYSLDVTSSYVSYTRALSGIGLAFFFVALNNVSLGSIKPTEIVAAAGIFNFMRNLGNSIGSSLFIPLWNHSQAYHHEVLASHIHTGNPNFLPLINSIPGSIQAKLVVINGLITKESATMGVNDVLLIAGFITLALVPFVLLANRTTGSTQGGH

>1123308.3.peg.229_MFS

MKRYKIQNIYFLISSRAISRIGDIMFDFANNTFLAGLNPTSLSLVAVYQSLESIIGVLFNLFGGVIADSFKRKKIIIATNILCGFVCIILSFISQEHWLVYAIVITNVILAFMSDFSGPSYKAFTKEIVKKDYITRLNSSLETTSTIIKVTIPMVAIFLYNILGIHGVLLLDGLSFLIAASLIFFVVPVNEEVISKKKVTIKGILIDLKMGFKYVYSHKSIFIIIILSAVVNFFLAAYNLLLPYSNQMFGNISSGLYGIFLTAEAIGGFIGAVLSGFVNKELSSKRLMMFLTLSGLMLMLATPLYTIFHNLIILSFSPALFSLFLSIFNIQFFSIVQRDVDNEFLGRVFGIIFTIAILFMPIGTGIFSIILDPRNVFNFLIIGVSITLLSLIFGTLFKKYNVH

>1123497.3.peg.2297_MFS

MSQSQEFQPANMALCVFAIALGVFMQVLDTTIANVSLPTIAGNMGVSLNQGTWVITSFTVSNAIGLPITAWLSRRIGEVHLYVGALIAFSVTSFLCGISQTMGELVIFRTLQGLAAAPLFPMSQVLLMSVFPKEKRSMALALIGMVAVVGPIVGPILGGWLTYDYSWPWIFFINIPIGIFSVTVILSQLKDRPHQPMKTKLDIVGLATMALGVGALQIVLDKGNELDWFANNWIVGGAVFSVIMLIFMVIWELTDENPIINLRLFANRNFCIGTIILTLGFAGFFSINLILPQWLQSQMDYTALWAGLAAAPMGIIPLFMTPILGRFGSHLDMRKLASLSFVVIGLSCYARARFNSDVDFATIALVQLFMGIGISLFFMPMTTILLSDLHGPEIADATSLSTFIRTIGASFASSLTSWIWSRNAGVHHSIMAEQISPYNPQIAPSLQHGDPVSFLAQWNGIITSQSFMMSTIDLFSILTLLFAALVPLIFLTRKAVKEA

>1128399.3.peg.2447_MFS

MDEWVVQAVEHKPSMRLGHGAASTSPKPVPDQIRPHREPSAHSVGACALASTRNAPMTRSLIVIFTAIVLDAVGIGLIFPILPSLLQDITHAANVAPFIGAMTALYALMQFIFAPVLGALSDRLGRRPVLLISLAGAAVNYLFLAFAPNLTLLFIGRAIAGLTSANISVATAYITDISPEEKRARRFGLFNAMFGLGFIIGPVLGGVLGDHWLRLPFIAAAVLNGANLLLAVFVLPESRPGRREKIDLAALNPLKPLRSVLEVKSLLPIVILFFIFSATGEAYGTCWALWGADAFQWNGLSIGLSLGAFGICQTFAQALLPGPAVKLLGERAAILVGVAGVSLALTVMAFAGQGWMIFAIMPVFTLGGIGVPALQSLATRQVDENSQGQFQGVLASAVSLASIAAPLGFSSLYFLFRDEWPGAIWLSVVAVYALAVPLVLGLRLKMPERAAVS

>1134055.3.peg.2323_MFS

MKPEIVLRGWRFWTIQFVLPLEFVLALYGSSSYAAFNLYSVGDLGQSPSHASWSSAIFFAGRGFGMFLAPLVSRRFRSIPSLLASCFGLSAVSFFCGLIGDFYLFLVLRLLLGFLSGTAMILAQFITLRLHPVERWPNVITGFGLLLGSTFAFGPNVGAILEEAVGWRAFFLIAALLHLFFGSLLWAVLVRRQEEPVAFRFDWVGLGLMLLSMLFLQAVVVRGQDEDWYNSTFVIVLAAVSVLSLIAFVIWELGQKEPLIDVRLFLQPHYTTAVLASSVLLMLAFGMLSLILLNLQAVGGYTPDLAARSFLPVFLLMPAGWILATYLNRHVDPRWPSALYLLGFAAFAYWVSTYDYFGRRSWYTNLLGSQVLEGFCLGAIATLTAVALQRTPRHRESTASQTLMLVRTYGMSWGPGILGTFLTHRTAFQQTRLVETAPWGDPAFGLALDRLLQAGASSLQGVRLLGRYASSHAVMLATEDVFRFCFWCFLGLAILVCTPLARKREPTRANQSPE

>1138383.4.peg.3759_MFS

MAGQAMTSNGTRPAAKFPGGMQAWGMQTDSTDTPEIGAGVRWSIMVVSLLATASSFLFINGVAFLIPSLRVRGVRLDEAALLASMPSWGMVVTLVLWGYVLDRVGERVVMATGSALTAAAAYAAASAHSLVLMSVYLFLGGMAAASCNTAGGRLVSAWFPPQQRGLAMGIRQTAQPLGIALGAMVIPELAEHGPQHGLRFAALACAVGAIASVIGIVDPPRKPRASASHQELASPYRKSLTLWRIHAVAGLMMMPQTVTVTFMLVWLIRNLHWSVTAAGGLVTLSQLLGALGRVAVGRLSDRVGSRMRPVRYIAAVAVLALLLLAWADYMNSRWQAGLMVVIAVISVLDNGLEATAITEFAGPYWSGRALGIQNTTQRMMAAAGPPLFGALIAAAKYPPAWLLCALFPLAAVPLVPTRLLPPGLETRARRQTVRRVRWWRAIRSHAMPNRPERRLPQRSGENY

>1154756.4.peg.1911_MFS

MSLVSSNTLEQGFEAPRRYLAAAAILIGVVMAALDSSIVNISLPSIAEALRVDSASVIWVTNGYQVASAATMLICASLGSRIGERRFYTAGMVLFTLASLGCSLSSTFGMLVAMRVLQGVSYAVMISVGLGLYRVIFPPNALGTILGINALAFAVGTAIGPALGGLIISYLDWPWLFYINIPLGALAIVFSLISLGVDTDEREKGFDWGGAVTSAAALGLMVIAVDQIGRWDSRILILCGVASVVLVAIFLNAQRRSKNPLLPLDIFHSRRYSFAVISSVSMFVAQGMALVGLPFVLQHAYHYSVLEAAFIFTPWPIAVAICAPIAGRLSNRLNPTQISTVGVMIFCLGLGSLALLPEAATMNDFLWRVAVCGIGYGLFLPPNNKEMFSNVAANRTVTASGVLSTARTAGQSIGAALVAMVIALLNGLTNDAGAQFAVYVFGLACLISALSSLSSMLRLHR

>1156937.4.peg.1301_MFS

MNKTIKTFLLFTLYFLLQCLCFFQSGSYTDIIPYATAELGQSQSHGSWTNGFFFLGQSFGLLIATQISLQYGRKKTVFFFSFLLAASSLFCALSQNFYLFLVGRTIQGICCGVLILGSQSLVFEQSPDSWRLMPLMLGAVASVLPFTIGPVVGGYGKELVGRESMSWKYWFVLSAACLLILSFLLHLCLEDTKERIEKRPWDWKGLILLSSMLGPLQMIFNMGDDYEWFISPIIDFLFFLVVISFVCFIYVETTTKEPLVRIDLFLRKNFLIGTFSLAFGFLLFYGLWTTLLVRLQNQSLFPPHLAGILFVSMALFSTPIVIWFPRLLGRISLRLSSFVVFFLLGIVYCWMGYFDFYQKRWFWMQPSFSFILQGISLGLFFLSLTNLIISGLSPKNQLRAIELSSSLRILAQGWASPLIGTLIYHRIVYHKMRLDEWLDRGNLFLTDLFLRFKEQGLGKEIAIRLLDQSAITHAFILSLNDAFRLCGVGFLILSGIILLAKEKR

>1193181.3.peg.769_MFS

MTSYDSSSSGAADAPASPASSAPAQDNPKRAVPVLLGLFVFSLIVDSAFRFTSKPIADDLGLSVTTVSLQTTLAGIIIGVGAVVYATLADSISMRKILLAAIAMICAGSLIGFAFRENWSMILTGRIIQTSGLAAAETLYVIYVTKYLSKEDRRTYLGFSTSAFQLAMLVGILTTGYISTYISWSVLFLVPLLSVLAVPSVLKTVPDHQLSGSRLDVFGIVLIAALATNVMLFLQNFNWWFMVPVVISIALLWWHISSHTNVLVDRAFFADRRYVSMLLVVFILYSVQLAYIFMFPFMVSELYGISFDNISLLTVPGYACAVVVGALSGKIGERLSVRSTITLAMVLIVASLLIPAVFVTTSVVPFVLSMVVFGSGFALMYAPLVATAIREITPERSGVAIGFYNLTINVAVSVGIAYTAKLLDLKPSLFDGIVSTPDGFDPSFSNVLVIVAVVALLGLVVYRVASSLLARADRAAGRPVETAALDG

>1195763.3.peg.611_MFS

MTLTMERYCGVGRGYSPPFLILFTHDMTLCFAVGLQPGTVVVEIMNIVDFSHSVVSIIPPLVALGLAILTRHVLFSLGVGIVLGALLLSDFAPLQAASYIGTTVKGLFIDDGSINSWNMSIVAFLILLGMTTALLTLSGGTRAFAEWAQTKIKTKRGAKLLAAFLGVFIFVDDYFNSLAVGSISRPVTDRFYVSRAKLAYILDSTAAPMCVLMPASSWGAYIITLIGGILVSHGVTEYTPLGAFLQLAPMNFYAVFALLMVFVVAWFQLDIGPMKKHELEASHCRGFDEGDADKRAKDLNEELEIVESANGKVSDLVMPIIALIIATFFFMIYTGSQALSADDLPFTLLGAFENTDVGMSLVYGGLIGLVSALIPIFRQRIAMGDVVSTMWIGAKSMFGAILILLFAWSIGSVIGDMATGKYLSTLVEGSLDPMLLPAILFLLAGVMAFATGTSWGTFGIMLPIAGDLAAATDIMLMLPMLGAVLAGSVFGDHCSPISDTTILSSTGARCHHIDHVSTQLPYALSIALVSTIGFLVLGATDSLAVAFLAATVAFIIMCSVLYWISRRSSDLTAKA

>1202785.3.peg.1721_MFS

MALESKKELKPALLVVLQGLFSIEFLLGTYSPPAYATFNLYPAGDLGVSPSHASWISTIYFAGQAFGLFIGPWFDRAFGRVKSLLLSIGFFALFDFLVAISSDYYLSLFFRLLLGISGGSTMTLCQLNLLDYYPISRWPFVTTYFGFLQVSVFGFGPVVGGFINESFGWRAYFLTSCSLHIICGLIISWILLVLTEKRQEDTPVPHPFDWIGFMLLLFAALCFQTLVTRGQDEDWYNSTFIDLLFLFGGISLLYFVVWEMGEKNPFINLKLFFKPTFLISSIITPISFAIVYGLFSTLVFTLQVLKNHTNFSSFQAGLAMAPLLFFLPIIYPLSVFLSPRINPKIVASILLILLGIFCYWTGYYDFFNKRAFFDQFFNQYILFTQVLNGAYVGLVPALNAIAINGLSKKNQESAVNTSILLRTYFLTWGGGLLGTMLMEHRRDFQQTRLVETFTGQNSESLSFIASLQHLGLNNLQIQSKMVEQAASHSIILALDDTYRLCSWIFFLMAILVWIPAMKKKEYNFLKIFYNVCRKEKNDPKIILALAKYYHGMLFIYGALDKRQILQRSFSAISVNAYWHDGCRLCYSAKRHTCCSSP

>1206730.4.peg.6049_MFS

MPFALYMLALAVFVMGTSEFMLAGLLPAIASDLDVSVGTAGLLTSAFAIGMVVGAPAMAASARHWPPRLTLLVCLLAFASCHVVAGVTPAFTVLFISRVVAALANAGFLAVALSTSTTLVPEDRKGRALAILLSGTTIAMVAGVPAGALLGTALGWRATFWAIALLCIPAALGILQGVPNQSAGAATGNGPAPGIASELRQLRSSRLLLAMSLGALINGGTFAAFTFLAPIVTRYAGLSDGWISVVLVVFGLGSFLGVTIAGRMSDQRPGLVIAVGGPLLSAGWVALASFGSHPALLIVLVLAQGVLAFGVGSTLITRVLYAATGAPTMGGSYATAALNLGAAAGPALGAAGIAAGLGGPAPVWVAAAMTTAALGVALLSGRMLTCDAMEVTRWRRGDRYSRPSPRKLPGSASKVWGPDRKHISTKARSPWSSPRSTACRSTPCWCTSWWCWCRSRR

>1212819.3.peg.2871_MFS

MDEALRITGARGILLTPGDVPAGGASLLVRRFVPMRALLPRCRALVHHGGIGTAALAYEAGIAQVVTPFAHDQFDNAQRVAASGCGVRLDGPVDGVRLGAALARVLDDPARRRAGRLRARGGFHRAVRAGAGARGRAIRTVRRRRRGGKRMSTASPLHDAHAAPASPAAAPDTSARPLRGARLALLTFALSLATFIEVLDSTVTNVAVPAISGSLGVSNSQGTWVISSYSVAAAIAVPLTGWLARRVGELRLFVGAVLLFTLTSLLCGLARDLHVLVVCRALQGLFSGPMVPLSQTILLRAFPADKRTVALALWAMTVLLAPIFGPVVGGWIIDSFSWPWIFLINLPIGIFSFAVCTAMLRPDAQRGAAGPVDVPGIVLLVVGVGALQAMLDLGHDKGWFGSPLIVTLAIVAALAIVSLLIWEAGDAHPVIELSLFRDRTFSFCVLIISLGMMSFSVVGVVFPLWLQAVMGYNAFHAGLATAPLGILALVFSILVGLHAHRFDARVLATFGFLVFAGVLAWDAHFTLNMTFAQIVAPGLIQGIGLPCFFIPLTAATLSRIPDDKLAAASSLSNFLRTLSAAFGTAMSVTLWDNRATYHYDVVSQSVTQASANTQRFVHALNAMGINGVRELTTLNRVVMQQAYMMATGDMFWMASMTCVALAAMMWLTRPKRGAAASFGH

>1235279.3.peg.2664_MFS

MIERTGIKGENFMKKNIMLTTVLINLFIAFMGIGLVIPVLPALINELGLSGSAAANLVAAFALTQLIVSPIAGKWTDKYGRKRMIVVGLILFSLSELLFGLAQSISLLFVSRLLGGISAAFIMPAVTAFIADITTIDERPKALGYMSAAISTGFIVGPGFGGFLAEIGTRVPFFAAFGLAFIAALFSMAALREPKRQKAEAEEMVPGTTGIRKIFAPVFFIAFVIIFILSFGLAAFESLFALYTDHKYGFTPKDIAIMVTGGGVVGAVAQVFLFDRLNKWLGEIRLVRWCLIVSAVLVYCVTLVSSYFMILLVTMTVFVGFDLVRPAVTTYLSKVAGNEQGFAGGMNSMFTSLGNVFGPVIGGILFDMQLDYPFYFATATLAAGVILSYFWKKPKALAAGPS

>1235795.3.peg.2132_MFS

MKTKSKQNESVQNRVSTKLFMMVLVLSSLLAAITVDMVNPVLGLISESLQASTVQVSWVVTGITLLLAIGIPLYGRMSDFIELKKLYTFATFVLSIGSLICVLAPSLPVLVLGRMVQGAGMSAIPVLSVVAVSKFFAEGKRGTALGVIAGCIGIGTALGPIFGGVVGQTWGWPALFWITFILSLFTVVGSIFALPGNKPITADEAGRGFDLAGGALLGLAVGLFLLGVTQGFTSLSTLGSLLGSLISMIGFIWRIGVARNPFVRPDLFKNKFYVSSVVVAFLSAFSYFAVLVYVPLLNLEVNQLTPGEAGLTLLPGGAAVALLSPWVGRISDRVGTKSLIFTGLIVMGSSTFFLSTFASGASPIMSSVGVMGAGIAFALVNSPATNSAVKVLQKDMIGVGMGFFQGALYLGAGAGASLVGAFLHARRDANFPLNPMYRLDVVNYSDSFLVVTIAVIVALIASIGLKNDKQGSRLVKPTK

>1242245.3.peg.4106_MFS

MRSKDFSWRYSLPATLLLLSPFDLLASLGMDMYLPVVPFMADALGSGAGTIQLTLTAYLVLLGAGQLLFGPLSDRLGRRPVLLGGGIAYIAASFGLTVVSSPELFLSFRVLQACGASACLVSTFATVRDIYSGREESNVIYGLLGSMLAMVPAIGPLLGALVDAWLGWRAIFGLLGMAMIGAVIAAWRLWPETRRHRTADLQWSQLLTPVKHLNFWLYTLCYSAGMGSFFVFFSTAPWLMMGRQGLSQLSFSLLFATVAIAMMATARIMGRLIPRWGSLKTLRVGMGCLMAGALLLAVGETLAPVSVLGFIAPMWLVGVGIATAVSVAPNAALRGFDHIAGTATAVYFCLGGLLLGIIGTLIITLLSTGTTWPIIAYCLILATAVLCLSCINPNRRHLSQEEHDALALQGTDSAQSVHDHD

>1243664.3.peg.3878_MFS

MASFFSKTSDMMTAKQRWTALIVLAASLFVVMMDMTILIMALPDLVRDLNPTSTQQLWIVDIYSLILAGFIIPMSALADKWGRKKALLTGFALFGLVSLLIFFAESASYVIAIRFLLGFAGALIMPTTLSMIRVIFENPKERATALAVWSIVSSVGTVFGPIIGGALLEEFSWHSAFLINVPFALLAVVAGLFLLPESRVSKSQAHSWDIPSTFLSVAGMIALVWSIKEFSKEGLSELTPWIVIVAAFVMLILFVRRNLTSSKPMLDVRLFNSRPFSAGTIAALMTMFAMASVILLVAQWLQVVEGLSPFKAGFYLLPMAVGAMVFAPLAPGLAARLGAKIVLPIGIAIAAIGMFIMYFFGHPLTYPTLAVALILVGAGTASLAVASALIMLETPTEKAGNAAAIEESMYDLGNVFGVAVLGSLASQLYRSYLDIEAFSSNGIVGELAHIANESVVGAIEVAKITGFTKLATEATAAFNDSFVTTALIGGIIMMIVAVIVFILIPKSLDITKQNHH

>1265868.3.peg.2237_MFS

MSSANPGPAGTADQAGGAFTHRQILTAMSGLLLAVFLAALDQTVIATAMRTIADDLHGQTEQAWATTGYLIASVLAMPFYGKLSDIYGRKPMYLISIVVFIGGSVLCGTAGSMWELALFRAVQGLGGGGLMSLPTAVVADLAPVRERGRYFAFLQMAWVVASVAGPLAGGFFAEAGQVFGIDGWRWVFLLNVPLGLLALVTVRKALNLPHERREHRMDVLGAAALALFLVPLLIVAEQGRTWGWGSPAALALFALGAAGLAVFIPVELRRGDEAILPLGLFRRGSIALCSAVNFTIGVGIFGTVTTLPLFLQMVQGRTPTQAGLVVIPFMLGTIASQMVSGKLIASSGRFKKLAIVGLGSMAGALLAMATTGATTPMWGIVLIVLWLGVGIGLSQTVITLAMQNSAPKSQLGVANGASGLCRQIGGSTGIAVLFSVMFAVALGRLADLLHTPRYERLLTDPAITGDPANHRFLDMAESGQGAGINLDDTSLLNGIDARLMQPVTDSFAHGFHIMFLAGGVVLLAGFVMTWFLRELQEETAPEEERPAESGAGAKNGPLPASDA

>1332070.3.peg.3602_MFS

MIAKVNNWRQNSRHNSLSLRSPALAVPYFFFTISKIKMKTSLPPAALLGRQALLFPLCLVLFEFATYIANDMIQPGMLAVVADFNAGVEWVPTSMTAYLAGGIFLQWLLGPLSDRRGRRPVMLAGVLFFIVTCLAILLVTNIEQFIVMRFLQGIGLCFIGAVGYATIQESFEEATCIKITALMANVALIAPLLGPLAGAAWVHVASWQSMFVLFAALAAIAFVGLWKAMPETATLRGEAFSAANLWRDYRQVLANRRFICGSLAIGFASLPLLAWIAQSPVILIKGESLSALDYGLLQIPVFGALILGNLTLARITGKLSIERPIKLGAWPMLLGLLLAALATVFSAHAYLWMTAGLSLYAYGIGLANAGLYRLTLFSSNVSKGAVSAVMGMLSMSVFTIGIELAKVAYVWGGNGLFSLFNLVSGLCWLMLTALFLNKHRGGATPAPSVTV

>1378168.3.peg.1312_MFS

MNKLKWKQTFYFLWVGQAVSVLTSSILQMALIWHLTVITQSAFVLSMASLAGFLPNAIFGIVAGTFVDRMDRKGILIGADLFIAVISLTLAIAAQNGNIAVWLVLAVLAIRSIGTAFHTPAISAVTPLIVPPEELTKCAGFTQSLQTIGYMAGTAIAGILYPIWSISGMVALDVFGAIVASLVVALIKIPKIENADRANQSKSFFEETKAGYSALKKEKGIFALVWIAAAFTILYFPINALFPLMSLDYFGGTTFQASVTEIAFSVGMLVGSVILGIGGGIKNRGLAIPFSIMLMGVPITFSGLLPQSGFWAFAFFCIIMGASAPFHNGPVTALIQEKLPPEYLGRAFGFYGSIASLAMPVGLLISGAFADIVGITKWFFITGTLIVILALICLAVPSIRTIDKDGKKADG

>1410653.3.peg.3382_MFS

MVKKLSAYKIYLLFSAITAMCFSLVATVMVVYHIEKVHLNPLQLILVGTTLEAACFIFEIPTGIVADVYSRKLSIVIGAVLTGLGFILEGSISSFAFVLTAQIVWGLGSTFISGSVEAWIAEEEKEQDLNRMYIKGAQAGQIGAVIGIILSTMIGNLSVRLPIIISGCLFVISALFLALYMPENNFTPSAPEDLNTFRKMGYTFKSGLKFVKSKSIIMILLSVTLFYGLSSEGYDRLSNAHFLQDTTLPKIGNLQPVTWFGIFGIAGMVLSAIAMQFIIKKLEEGDKNQSGKILFIVNIFYISFMLAFALTRNFNLMLVAYLSTNLFRAINDPIFNAWLNNHIDDSARATILSMNGQINALGQIIGGPIIGIIATKFSISIGIACTSLLVTPVLVLYILSLIMDKKDVKMAKGREDTYENN

>1429438.4.peg.3523_MFS

MNAATGKPPAREKWLIAVTVMLATYVAVIDLTIVNVALPQMRGTFGVTLDAVTWVAVSYNIAEIVMVTMASWFTQLMGRKRFYLACLTLFTIASIFSGLARSLEMMILMRTLQGLGGGALIPMAQAIMLEVFPEEEHGMAMAVFMMGVVLAPAMGPVLGGWLTDAYGWPWIFYINIPIGVISILLVMAFLKESAYLQQGLSRIDVVGIILLVVGLTALQLFMEQGERRDWFESNFVIAMAVLALVGLTALVIWELRVEEPIVNLRVLKNLPFLGGIAMGLIFGLTTFGSIFMLPLFLQQLQGYSVMDSGLIQMPRMLIVVAVAPIAGRLYGKLDSRLLAAIGTAVMMAGYLDMSRFTLEVGWQRMLPGLLLTGGGMAFLFSVLSAATMRTMPPALLTAAAGLFTLSRRIGGNIGYAFVANQISHRSTFHETRLVDHLTPYDSNTMQALDGLTGRLAVYGLPPGVAEQGALKLLDGAVVRQATMMAYNDVFWMMGMMFVVTFPFVLLLGGRRS

>1432558.3.peg.787_MFS

MMHRISHWLSRHAAALFFPAALILYDFSAYLTTDLIQPGILHVVRDFNADVALAPASVSLYMAGGMALQWLLGPLSDRIGRRPVLLTGALIFTLACLATLFTTSMTQFLIARFVQGTSICFIATVGYVTVQEAFEEKRSIRLMAVITSVVLVAPIVGPLSGAALMHFIHWKALFGIIAAMGLVAWLGLLLTMPETVRRGDVPFSPLGVLRDFRNVFRNRIFLLGAATLSLSYIPLMSWVAVSPVILMDAGGLTTSEFAWSQVPVFSAVIIANLSVARWVKDPTRPRFVLSAVPVQMLGLAILIVGNLVWPHVWLWSVLGTCFYAFGIGLIFPTLFRFTLFSNDLPKGTVSASLNIVILSVSALSIEGARWLWFHGGRLPFHLLACRRDCRRLLPGRTVTPPARTSGDRSPAVVSPFTEKAPS

>1432558.3.peg.3586_MFS

MCGSSLWIWLAAIIGLSAMNMLIGILIACSIVSLVLLLVVTPPRVAQYDEEAAVES

>1432561.3.peg.1625_MFS

MTPFNPNAQQIYDQLQGMGMTQQQASGWIAQQITNQGLIISANEIFWISAAIFILLLGLVWFARPPFSAGGGGGGAH

>1463858.3.peg.4357_MFS

MPDSADRTSPSPSPTVDAAGADGSTAEPTAATKADEAVHQSPRVDGTERAANTAGAGNTERAANTAGAANGPADGPARARMPIAVYILGLSVFALGTSEFMLSGLLPPLAEDMDVSIPTAGLLISAFAIGMVIGAPLLAIATLRLPRRTTLIALITVFGLGQVIGALAPSYGILFASRVISAFACAGFWAVGASVAIAMVPRDARARAMAVMIGGLSIANVLGVPAGAFLGEHLGWRSAFWAVGAASAIALVGVVTLIPRIPLPAEKPRLARELTIYRDRQVWLAIAVTALAAGGVFCAFSYLAPLLTDVAGLDGGWVPTVLALFGIGALVGTAIGGRYADAHLFGVLISGVSASTVLLGVLALAAGSPVVVVAVAFLLGVSAFYTAPALNARMFNIAAAAPTLAGATATASFNLGNTSGPWLGGVVIDADFGFASTAWAGAAMTAVAIVLAALSLRLHRTAARSRVVAGSAGTGTGTGTGTAGTLNASSSSHSLSTCSASAAQADRA

>1505605.3.peg.2079_MFS

MSDLALSAQSPEQPAAGAALAEAPTYEVGFRKWLITITVITCAIMELIDTSIINVATRQIAGNLGATIEETAWVITAYAVANIIIIPLTGFLSDFIGRKVYFTISVAVFTAASLLCGFSHSIETLIFWRIVQGLGGGALLATAQTVLVETFPPEELDTANGIFGAGIVMGPTLGPVLGGYLTDNYHWGWIFFINVPIGILATFLSWKYIKGTKSPLEGKIDYLGILFMALGIGGLQIVLEEGERKDWFSSNFVVTATIVSAVSLVLFVIRELRIKNPVVDLRVLANRNVAIGSVLRFAFGVSIYASVFLYPVFVQGFLGWNATRTGLLMLPSSLITGVLMGAMGALLNRGVSPKLLITIGFTSVIGYEVATYFLATPQAGEWDFFWPQLIRGVGFGFIFVPVSGLILAGLKGKDIAQAAGLTNMLQLLGGAVGIAAVNTYVVRRISTNRMDLLPNLSTGHPAAVERLDNLTRFFQGAGNSLDEAQRMAYGVLEGTVSTQAAIISYAEGFMLIGLICAVALPLVFFARIRKGEAIVAGAAH

>1522311.4.peg.159_MFS

MLASINSVTSIFGPVAFTTIFAFTYINADGFLWLCAAALYVPCVILIVRGTAASPKFGSWASGDSM

>1536652.3.peg.3452_MFS

MSDITESIPTNVVKSNRRKKLLIALAAVVVFSGAGATAYWALYGSHIISTDNAYAAAEVAQVTPAVGGTISEVLVTDTQAVKKGDVLVKIDQTDARLALAQAEAELGQAIMVPLTGWLAARFGPVRVFVWSTALFGIFGMLCGLSTSLGMLVVARIFRGFSGGPLMPLSQTLLLRIFPKEKAAAAIGLWSMTTLIAPVTGPILGGYLCDEYSWHWVFMISTPFAAVCAFIAWNMLKRCEAAAIRTPFDMIGLVLLVIWVAALQVMLDEGKNLDWFACDKIVALCIIAGIGFAAFIIWELYDDHPIVDLRVFRHRGFTVSVTTIGLAFAAFFGINVLIPLWLQNFMGYTATIAGLAMAWSGLSSIFVAPMAAQLARKTDPRKLVFFGVIWFGIVTLWRAVATTDMGFFDVAMPLIVMGFGMPFIFIPTTDLALGSVEAHEMDSAAGLMNFLRTLSGAFATSMITTVWGDQITRNHAELVGLADQDLSVRAMLDGSGAPLDVVNQVIDYLIVQQSVMLATNQMMVAIGAIVIVAALITWLSPKPARVVEPGTGGH

>1560354.3.peg.1457_MFS

MASWRSMFWLFALLSAIAFLILWRVMPETAGDRSHSVALPQLARARMVMLSSFAIELVVYSHSDLGSALAGDPDA

>1590596.3.peg.2139_MFS

MGKPKWFRTYLFIWSGQFVSMLSSYAVQFAIIIWLSLEYKSAQVLAYAGIASILPQAIIGPIAGVYIDRLNRKNVMMLSDAFIAVCTFVILVVLKNGAINLFWIYILLGLRSVGNAFHTPALQAIAPLIVPQNELIRVAGINQIIQSVTSIAGPAIGTLAIASFPISEVLYLDIIGAVLAITSLLLVRIPNLVEQNKGSLLTVLYDLKEGLRTVSQNRGLSLLFFFAMAITLVVMPAAIMFPLLTTGHYGGGKWEMGLIEVAWGSGMLIGGGILSVFKFESSKVILINTMYTLLGLTLLASGLLPEEAFIIFVIITIVGGLSLSVFNGCFTAIVQIEVVPEKLGRVFSLYFSLAILPSLIGLLFTGWIVDTIGINQTFIICGLLAIVLGLAAFTFPTLMQLGNNKTIDNNEN

>1678637.3.peg.358_MFS

MNSPAHDEPLAAARPREAGLRGRPWPTLLAVAVGVMMVALDSTIVAMANPAIQQDLGASLADVQWITNGYLLALAVSLITAGKLGDRFGHRQTFLVGVAGFAATSAAIGLSGSVAAIVVFRVLQGLFGALMQPSALGLLRVTFPPERLNMAIGIWSGVVGAATAAGPIIGGLLVQHVSWEAVFMINVPVGAAALAVGVVILKDTRADKAPESFDVPGIALLSAAMFCLVWGLIKAPAWGWGDLRTLGFLLAAVVAFAGFALREGRAGEPLVPLSMFHSTALSAGTVLMVLMSFSFIGGLFFVTFYLQNVHGMSPVSSGVHLLPLTGMMIVGAPVSGAVISRFGPRPPLVAGMLLTAAALWGMSTLEAGTGMGVASCWFVLLGLGLAPVMVGTTDVIVSNAPAELAGVAGGLQQSAMQVGGSLGTAVLGVLMASRVKDVLPGEWSGAGLPPLAADRAGAVEDAAKVGVAPSLPGLPRGAVAEAVHASFISGMGLAFVVGAVVAVAGAGFALFTRKGQGGEPPAAAGRAEAAEAAAPAGQG

>1682204.3.peg.5478_MFS

MALFFAPLTVIILSGQPPEKVPAAAGLSTFGRVFFGGIGTSLANVVWNNRTIMHHEILTQQSSPTNPIFNAQMNTYHSALGLSQQASYALFDHTVQSQAAMLGLNDVFYGAAIIMIIIIPLIWITKPGKAGGSSDAAAAAH

>1688405.3.peg.76_MFS

MSTSAQATAAPKPPAQPPGTYPPLEGATRIIGSVALSTAVFMNVLDTSIANVSIPTISGDLGVSTSQGTWVITSFAVANAITVPLTGWLTQRFGQVRLFLMSTLLFVLASWLCGFSPSLEALIAFRVLQGAVAGPMIPLSQALMLASFPKAKAGMALAVWSMTTLVAPVAGPLLGGWISDNYTWPWIFYINVPVGLLAAWISWRIYGERESVTRKLPIDKVGLALLVVWVGALQIMLDKGKELDWFASPTIILLACLAFVAFVFFLIWELTDAHPVVDLRLFKERNFSVGAITLAVAYGVFFGNVVLLPLWLQSNMGYTATYAGLVTAPVGFLAILLTPIVGKMLATRDPRQLVTVAFMIFALVCFMRSGFNTQTDVRTLMVPTIIQGAAMAAFFVPLTSITLSGIEPWRIPAASGLSNFLRLTAGAFGTSISTTLWENRATLHHAQLTEAARPGQQAFDQTLQTLNGLGMSHHQALSTIDGLINAQAFTMSAVDVFYASAIIFLLLTGLVWLAGPSRRAAAEAARRKRPPGRTERAPPFLSRKRASSRGCPFASGRQPASVLEEGLPVQPFHDVARQRADGREVEHRHAARAPQLRREPERGLDQVGELAAQPAGLRVFLFVEAQPVLGRQVEPVHVRLAHAVGHQHLQRRVGHGVRIEDAQRHVEAVAQAALGVQQLHVGRGRGHALEHRRRGGAFVGARGNQLDNADAAADVFLRDALHDVAQHFGHHVQRQVARHDLPHQVDAVAHEVLVDDAVVLERQIELAGQRDRAVHPHGHLDVHAVVGNLGHRGAAQDEMHLLVGRIEPGDRGVIGNERPKAL

>169963.11.peg.2889_MFS

MTSTAYKGTNKLIVGIVFGVITFWLFAQSMVNIVPAVQSDLGISSDLLSIAISLTALFSGIFIVVAGGMADKFGRVKLTYIGLILSIIGSLLLVVTQGSTLLIIGRIIQGLSAACIMPATLALMKTYFDGADRQRALSYWSIGSWGGSGICSFAGGAIATYMGWRWIFIISIVFALLGMLLIKGTPESKVVQNTKAKFDSFGLVLFVIAMVCLNLIITRGATFGWTSPITITMLVVFLVSAGLFFRVELRQANGFIDFSLFKNKAYTGATLSNFLLNAAAGTLVVANTYVQIGRGFTAFQSGLLSIGYLVCVLGMIRIGEKILQRVGARKPMILGSGITAVGIALMALTFIPGTLYTVLVFIGFALFGIGLGMYATPSTDTAISNAPEDKVGVASGIYKMASSLGGSFGVAISATIYGVIALSGNIDLAAMVGLLTNVGFCVVSLISVAITTPSAKKALELKAAKE

>1736316.3.peg.4820_MFS

MQEKTSASIDAHPRATSREWIGLAVLALPCLVYAMDMTVLNLALPVLSRELQPTSAQMLWILDIYGFFVAGFLITMGTLGDRIGRRRLLLIGAAFFAAASALAALAHTAELLIAARALLGLAGATIAPSTMALIRNMFHDPRQRQFAIGVWIAAFSLGSAIGPLVGGVLLEFFHWGSVFWAAIPVMVLTLALGPRYLPEYRDPDAGHMDLPSVALSLAAVLLTIYGLKHLAEQGVHAEGLAATVAGLALGGLFVRRQRHIAYPLLDLRLFQHAPFCAALAAYALTCLAMFGVYIFITQYLQLVLGLSPLQAGLATLPWSLAFVAGSMAAPHLAARLPRARIIVVGLAAAAVGFCGVAAGQGLWLLVPATVIMSLGMAPVFTIGNEIIITTAPPERAGAASALAETASEFSGAMGIALFGSAGMVVYRRALNAAPLSDLPADALRAAGASLGGAVHLAETLPAVQGQALLLAAHGGFTLALQAVALAGALIVMASAWLVARMLRGVDLSAAPH

>1778.10.peg.4006_MFS

MTQTTSETGSWRQLLGRHLGTSTVLAGGVAMYATNEFLTVSLLPSTIADIGGDRLYAWVVTLYLVGSVVAATTVNSILRRFGARSSFLLGLAVFGVASVACAMAPTMEVLIAGRTLQGIAGGTLAGLGYALINAALPRELWTRGSALVSAMWGVATVVGPAMGGLFAQFGLWRWAFGAMAVLAALLAILVPAVLAAIATVEDEPAAPALRVPVGSLLLVGAAALAVSVAQLPHNSAAIGALLVVGVLLVAAFVLVDRRSRATVLPPSVFGTGPLKWIYLTLAVLMMAVMVDTYVPLFGQRLGHLTPVAAGFLGASLAVGWTLSEVASASLTNPRVINRVVLVAPLLMASGLAFGAVTQRADASSGLIAVWALALLIAGTGIGMAWPHLSARAMDSVDDPSESGAAAAAINTVQLISASFGAGLAGVVVNSASGGELMEARWLYGVFTVLAALGVLASYRATRTARRSTPPAAELRP

>1791.3.peg.3532_MFS

MTQPTATAGRRTPLLLIMFAALMAGAGNGISIVAFPWLVLQRNGSALDASIVAMAGTLPLLAATVLAGAAVDFLGRRRVSMISDTLSALSVAAVPVLALIFGAHVINVAVLAGLAALGAFFDPAGMTARETMLPEAAQRAGWTLDHANSVYEAIFNLAYIVGPGIGGLLIATLGGIDTMWVTAGAFVLSIVAIGVLRLEGAGKPDPSAMSAGVWAGIVEGLRFVWNSKVLRTLAFVDLAATGLYMPMESVLFPKYFTDRNEPAQLGWVLMALSIGGLVGALGYAVMSKYMKRRTVMLTAVLTLGVAMTVIAFLPPLPVILLLCVVVGFVYGPIAPIYNYVMQTRAPQHLRGRVVGVMGSLAYAAGPLGLILAGPLADASGLHATFLALSLPMLALGVAAVFMPALRDLDSPPGDGARADSGIP

>216594.6.peg.1390_MFS

MSGVSISSFEKVTSRHSKRPGATPARTHLAGHARKGFANLTHRRQPSSAAVLLVAAFGAFLAFLDSTIVNIAFPDIQKSFPSYDLGSLSWILNAYNIVFAAFLVAAGRMADLLGRRRTFTFGVVIFTIASGLCAVAGSVEWLVAFRVLQGIGAAVLVPASLALVVEGFEPARRAHAVGLWGAAAAIASGLGPPIGGMLVDWASWRWVFLVNIPLGVVAVLATSRALVESRAAGRRRKPDLRGATLLAGALGLLTLALVKGPDWGWVSVPTLAVFAASAITLVGFVLSSMAAPVPLVEPAYLRSRPFVVGNVLTLVAAAGFYCYVLTHVLYLNYVWGYSLLKAGFAIAPAALVAAVVAALLGRVADRHGHRLIVTLGALVWAGSLFWYLQRVGTEPDFLRRWLPGQLLQGIGVGATLPVLSSAALTGVAKGGSYATTSAVVSTTRQLGAVIGVAALVILIGKPEHGAAADALRRGWAMAAICFVVVAIAAVLLGRTNSKPGQELEPEPAAAARAAPTTTEPAAALIANRATDEADLLGNLPLFAGLDAAALAELADRVEEVELQAGSYLFLAGDASDSLYVIRRGRVQVLHGDIVIKELGRGEVLGELGLLIDAPRSASVRALRDSRLVRLTKAQFDQIANRGVLAALVRVLATRLREAPPPAVHTTSPGVVVSVVGVGADAPVQSVAAGLLTALSKQLRVVDPGRVDIDGLDRAERGADKVLLHAGAQDADWRDFCLRVADRIVLVTGDPDPGAAALPARAQGADLVLAGPTASREQRRSWEELITPRSVHAVHYRRVVQDLRPLAARLAGRSIGLVLGGGGARGFAHLGILEELEQAGVAIDRFAGTSMGAVIASLGASGLDAATADAYAYEYFIRNNPLRDYAVPIKGLVRGRRTLTLLEAAFGDRLVEELPKEFRCVSVDLIARQPVVHRRGRLVDVVGCSLRLPGIYPPQVYQGRLHVDGGVLDNLPVSTLATSDGPLIAVSLASGEVPGAPLQPDGPPRVPGIGDTLIRTMTIGSQRGADVALGLAQVVIRPDTSAVGLLEFHQIDAAREAGRAAARESMPQIMALLNQRR

>225992.4.peg.2582_MFS

MRSSAIIALLIVGLDAMGLGLIMPVLPTLLRELVPAEQVAGHYGALLSLYALMQVVFAPMLGQLSDSYGRRPVLLASLAGAAVDYTIMASAPVLWVLYIGRLVSGVTGATGAVAASTIADSTGEGSRARWFGYMGACYGAGMIAGPALGGMLGGISAHAPFIAAALLNGFAFLLACIFLKETHHSHGGTGKPVRIKPFVLLRLDDALRGLGALFAVFLNDRLASPPGKSAPFPTTAPCGHTTTILKMKQRVSLRTLALLSVVVLITGCSKPEAQQAAQEPAEVGVIVAAATPTSVATELPGRLEPYREAEVRARVAGIVTARLYEEGQDVARGAALFQIDPAPLQAAYDSEAANLARAQANLSAAADKLRRYADLVSDRAISERDHAESVAQERQARAEVALARANLQSAKLKLDYARVTSPIDGRARRALVTEGALVGEGQATPLTVVQQIDPIYVNFAQPAAEVMQLQKQIRAGALESVAPDQVRVRLLLPDGSEYARGGTLSFADLAVDPGTDNVTMRALFENPGRDLLPGMYVRVRLEQAINRDTYLVPRNALLRNAEGAHVLAAGPDGELKKIAVTAHRLQGANWIVTQGLAGGERIVVENAAHLAAGQKIKPVERAAPSAQAAAAENPEAGVQARTAAEGKKG

>333849.13.peg.1278_MFS

MENEQSVVLTNWKRNYLFFLSGQFLSGITSMVVQYAIIWYLTRETGSATILSFATLLGMIPMVLLSPFVGPLVDRWDKKALLIVTDIIVAIFALILAVVGTISESFPIWLVFVSLFMRSVAQTFQMPTIQSIMPTIVPSSHITRTNGQLGMVQSANFIIAPALGAALFSVVPVNYLILLDVLGAVFGVGLLIFVKIPKVSPEILEVPLTIFKDAKFGLQQLMDNKGLWYITINGAFVMLLFMPAISLYPLMTLDYFGGSVGQAGAVEVVYAVGMLLGGALISFIGTWKDRMKPIIIAYIIMGLTIGASGLVPNDSQGFLYFLILNAGAGCATPYFNTLLMAMIQQSYESNVLGRVLGNFNSLMNLAGPIGLLFAGPLADRLGVEKMFLFSGIGILLCGIVLFLTSAARKYDKELQKKLVKEHHEQKDE

>339854.8.peg.2484_MFS

MSLLNFPGFLTKGMGIAIVGGLLSIHLLNRKLLPMNANSFTHLYSNLLLLFAGIILISWLVTMKMYKCSRRNIYQVKRFIFQ

>371042.3.peg.3824_MFS

MIHGKNMKPLRFCLPLSAFMVIMDSTIANVALPAIAGNIGASQSQSAWIISSFVAACALSVPLTRWLALRIGESHLFIAALSVFTLSSCGCGVSTNFLMLIFFRVIQGISAGPIIPLSQSLLLKLYKSDEKRDALAIWSMTAVVAPVIGPVIGGIITSYYAWNFVFLINLPLGILVVVMCRKTISNTSKNTQDKKFDFTGYLLICLLVALWQYISFRKNSGNDNGFLVIISLLLLLFFLISQMCRKNTLLDLSFFLNRNYAIGTLCIFFSYIINFGSLVPSTLFNIYNYDLVTIGLLCSPAGIAPLFLSKLSGRMCKYVDSRILISISFLIFAMCYYWRACYFSLGMTPLMFASSQFFIGIASTLFYIPLTEKLFSDISKDDLTAATTLRQLCRTLSTAFGTILTSELWNNRLFFHTSRLSEKVYAGSLEYENFYQKFKLLGLNQQETLLYIRDQISFHSKLLSLNDIYWLDAGIFLFLACFTWLLTPTKK

>381666.6.peg.5563_MFS

MTWAIYRDRKTPTRKLSIDMVGLASLVTWVASLQIMLDKGKNLDWFSSPVFTVLMAPVVGKILPKSELRVLATLSFLGFAAVYFMRSHYTTGVDTYTRRTSRSHDRTPD

>382638.14.peg.388_MFS

MRFLGLFIVLPVISLYADSFHSSSPLLIGLAVGGAYLTQIIFQTPMGILSDKIGRKVVVVVCLLLFLVGSLVCFVADDIVLLVIGRFIQGMGALGGVVSAMVADEVKEEERTKAMTIMGVFIFISFTISMAIGPGVVAFFGGAKWLFLLTAILTLLSLLMLLKVKDAPKISYQIKNKIAYQPNSKALYLLYLSSFFEKAFMTLIFVLIPLALVNEFHKDESFLILVYVPGALLGVLSMGIASVMAEKYNKPKGVMLSGVFLFIVSYLCLFLADSSFLGKYLWLFIVGVAFFFIGFATLEPIMQSLASKFARVHEKGKVLGQFTTFGYLGSFVGGVSGGLSYHYLGISNTSLVVVILGLVWGLSLFFLNNPSKQKNVYFPLDAYNGEQFETLGDKIIEWYVNISEEIIIVKYNSDQISEEEIIHLAQNFRK

>388357.3.peg.2216_MFS

MPLPRALEPFRLGEYRVLAFAMFVSVFGAGMWAVALVNQVLELDGTAVDLSAVTAVGALGMLVVVLVGGIAADRFPLAALLRLVEAGNALTAGTVAVLALTGGLRLWHLGAAAFVFGAGVGFFYPAYSAALPRVLPARQLLAANGVEGTARPLLQQAAGPAAAGVLIGLLAPGGAVALIAACHLAALVLLLRLQVPEREALPVTGPTARAPLGDPAVAPATEPMVRPVAESAEPVVESVAESAAEPVVRPVAEPVVEPVVRPSAEPVVEPAAEPGAEPVVGSAPGPEAAAGPSDHSGGVFASVRRDLMEGVRYTLHTPWLLWTLLWAVCAVFLLLGPLEVLVPFLVRDRLGGDAATFGYLLACYGGASALASLVVASLPLPRRYLSWMIGLWGLGTLPFGLVATTESFWVMAVCLACVGAGDGAGMVLWGTLLQRRVPRHMLGRVSSLDFFVSIALMPVSMAIAGPVAQVVPMPVICWTVAVLTPVLGFVALRAGRMRQDELAHPLAG

>421052.3.peg.2788_MFS

MNNTILKTNLKGIRLLIAAFIVALANFMVVLDMTIANVSLPTITGSLAISTSQGTWIITSYAIAEAIGLCVSGWIAQRFGLVRSFSIALMGFTVFSICCGLSNSLELLVMCRVGQGLFGGPIMPLSQTLIISIFPQEKYIHALGIWAATTVLGPILGPILGGIISENWAWNWIFLINVPIGFFLIYGVYLFLSKIKSPLSKSKFDVIGMIFLLVWVGALQMMLDMGHDYDWFNHPKIWVLAMITLIVFSLFLAWELTGRQPIIQLHIFANKSFCIATLALSVAYGAFFGGIVVIPQWLQLNMGYTATWAGYLMATMGVGSLLMSVVVAKLIYWIDQRLLVSIGFIVFALSCYLRTDWANNVDFIDLAWPQILQGFALPFFFIPLSNIALAAVQSHELAMATGMMNFIRTLSGAIGASISMSLWSNYSQIARHEMVARIQMTQSQHALLSAHISQQNSLELVSNVVDHEAMTISINHIFWGFSLIFILISVLIWLLPKPQNMLGQIHLP

>42253.5.peg.3181_MFS

MMALVDHTQPRLRGWHFILFNLVLGLAHMVVLFNAGSYVALLPHAAGDLGGVLPSFGTWAQTDFMIALALAFPLARWLSCRYGEQRVFVAAFVVYAAASALCAIDGSIAAFVPARILLGLAGGVTLPLSQSLLLQEYPDRVKSLGLAIWGLFTLMPFTVGLGAGGWLADHWGWRALFYLNIPVALLIAALTAALLHGRSHVVRCERFDLVGFLLLAVIFGGLQTMLNEGNDYDWFDDPFLRGMLVLVIVAVPVWIVWELGERRPAVDLRLFAHRNFAVGLLCLGLGFLSIQGLLALFVVQLQVLMGYSSELAGLVFVPMMLLGLPTIAVMHDVAKRLDVRWLACVNGLGFAATFYWIGLFDDPHSYDQIFWPMVLEGVFLGSFFTPLTVLTLHGLSGEQMLRAAEAANIFRIAAGALGISWQGVVVFRRMPFHHLQLSDHFGGRMSASYDALHQLTSKLQALGFDPAMIQRQLQLAIKQEAGILALNDAFLLSSALCMVLAVLVWFAHSSRVPALKPAEAVRELQAEELMEQP

>439375.7.peg.2032_MFS

MRSPNQLCLDNLSLINFKSSSLSLINCEAGMKRTLMIVLAVTALDAVGIGLVMPVLPSLLRDVAHSDDVAGHYGVLLSLYALMQVFFAPILGGMSDRFGRKPILLGSLIGAMIDYAIMSAAPHLWVLYAGRILSGMMGATMAVAGACIADTVEEGTRARAFGWLGACYGGGMILGPVVGGALGSISLTAPFAAAAAVNGLMALSVYLVMPEVRRTTKPEPQAKGLHCALVPSGVQKGLKPLLWVFFLLQLVGQIPAALWVIFTEDRFHWDTTYVGLSLAAFGLLHAMFQWLGTGRLVATIGAGYTIIIGIAADGLGMASLAIATEGWMTVPILVLLAFGGIAMPALQSVLSDKTSQDEQGALQGMLASLTNISAVAGPVIFTAFYMRTAASWNGWVWLFGPAIYLAAAPLLIFVRRQSAGLPSRR

>470.1295.peg.2468_MFS

MPCAVFLILLTRQIEHFLTLRFLQGIGLSVISAVGYAAIQENFAERDAIKVMALMANISLLAPLLGPVLGAFLIDYVSWHWGFVAIALLALLSWVGLKKQMPSHKVSVTKQPFSYLFDDFKKVFSNRQFLGLTLALPLVGMPLMLWIALSPIILVDELKLTSVQYGLAQFPVFLGLIVGNIVLIKIIDRLALGKTVLIGLPIMLTGTLILILGVVWQAYLIPCLLIGMTLICFGEGISFSVLYRFALMSSEVSKGTVAAAVSMLLMTSFFAMIELVRYLYTQFHLWAFVLSAFAFIALWFTQPRLALKREMQERVAQDLH

>479431.6.peg.2835_MFS

MTGISGAPRHGGRTGKLLAPTVVDRAASWTPVVPDGLRPGRDRPPAGTREHHRQESKIMTERRAPNREPRRKRLWRPRSVPRALSLPRALSLPRALRPFGNPQYRWLTTALACSLFSVGIWLVASVWQVIQLGGSASDLSLVAFGSSLGLTLSVLIGGVVADRVPQRKILLVVEAVRGVCFALAGVLALTGAIQIWHLAVLGLVLGLADGFFYPAYSAWLPAIVDADQLLAANGIEGMLRPAVMQGLGPAAAGVIIAVWSPGAAFAAVAILQIGTAAALWTMRTTAVRRELDPDVHPLRSALIDVRDGFSYMVRTRWLLTTLLFATLLVLMVVGPVEVLLPFAVKDQTGGGPGAFAVALAAFGIGGAAGSLAAASIRMPRRYLTLMILGWGFGSLPLVVVGLTSSLAVMVVALFVTGFVFSAAQVLWGTLLQRRVPPALLGRVSSLDFFVSLALMPISMALAGPVGDLVGIGPTFLVAGLVPGLLAVGTLLIAKLGPDELAHPLDALPEAAEPPVPIPDQ

>557599.3.peg.5424_MFS

MTSPTASRTAAADTRSTCISLSPARRNIIFMALMLGVLVAAMDQTIVVPALPTIVDELGVSVHQSWAITSYLLGGTIVVVVAGKLGDLFGRKRVLQGSVLVFLLGSMLCGAAQTMTTLAVSRAVQGVGAGAISVTAAALVGEAFPLRDRGRYQGILGAVFGVTTVAGPLLGGFCTDYLHWRWAFWINLPISIVVLAVTATAIPALPRRPKPAIDYLGIMVITLATTALITATSLGGSTYSWGSAPIMGLFIGATVALGVFVWVEGRAPAGILPPRLFRNQVFAVCSVLSLMVGFAMLGALTFVPMYLRYVDGASATVSGLRTLPMVVGLLTTSVGAGIMVGRTGRYKIFPVAGTGLMAVAFLLMSQMDESTPALVQSLYLVLLGAGIGLSMQVLILIVQNTSRFEDLGVATSGVTFFRVVGASFGAAIFGALFATFLGRRMGPALVAGDAPVDAAHSPAVLHRLPHYVAAPIVRAYAESLNQVFLCAAFVALAGFILALFLREVPLADIHDSPSCLGDGFAVPRTKSPEDVLEIAVTHLLHEAPEVRLPNLAAAYQDSELDVAGLWGVLRIYQYERFFDTARLTDIAQHLHLPHQVLEPVFDRLVQTGYASREGDTLSLTPAGLGQIETLSGLLRRWLVDHLAVAPGVEQQPDHQEFEAALQRLTDGVLVQRDWYEDLDELAPAGTLVAAK

>566461.4.peg.7124_MFS

MAGSYATAARNVGAAVGPLVAATTLGTAVGHLGPLGASGLLVAVALLIAFPFRTVVVAAGRGAEVLQ

>575.7.peg.222_MFS

MATLLPIIVYKICLKSLLNHRHIVVLSFLCGRFLQNLRDVPVTYSSFTSTHEVFIDMKRHKNFTLLLMLVLLVAVGQMAQTIYIPRSPTWLSR

>60547.6.peg.7263_MFS

MASPKNASTPAPFTRWQFALGTFAVAVASFMNVLDSSIANVAIPTLAGDLGVSVDEGTWVITLYATPNAVAIPLTEWLTQRVGQVKLLVVAILLFVVSFTMCSLAPNLPILLVARVIQGAMAGPLVPLSQVKRRQGRSDSECSPVQLLGNQQPIRLVVLARWERVDAAKSVRVTDSMFMAAAQGIASAPSAYINAGINLLPPVTALRDIAVSVSLAVALQAHKKVPSSGLSPDQTKGLIRGKVWAPHYVPWRKIKSSAI

>637389.3.peg.233_MFS

MVVASWSLAWSIPMLPYLAGAYSTSLDHAVWSLTFYLMAWALGVVPATWLYRRIGELRSFQLSIALLLLATLPDVLSNNYSLFLVGRFFQGLAAGFLTPLIRRLLIQYAPPKWQGFAADLSIVNLVLPLLAGPSLAGWIAYNWDWRAAPLLTFPVGMLALGVCSALIPARDSERHKAPFDWIGLALLALAAGSVQILLNRGEDWNWWDSQRFQGLTALGAIFSIAFFVWERHHPQPCLDLSLLRRRNFVLPIPALIFGWGLLLGGNSLFVSALITQAGYTAYLAGLVLFPMALTGVPLIAMMSRISHSIGPRILASVCFLLVALYGFSTQINRSSSLDSLLLAHLIEGAALGFYLVPLSLIMFSRLPSNRLPAAATLQNFVRILGGAYLSSIFSALWLRHGSYFRAHLAWQSPAAPLAELAQNLPEINSTEKAAVDVHLLVMQSLALSMQSMLALWGLMALLVLALLWFTKAPFRRRPGQKRRVTIEQEIVESADLIPIRKAATTTSSSVTDADTSSVHA

>637389.3.peg.799_MFS

MAKIDPPSVADEARVRPLSQSWPLFVGVGLGLALGSFEGAGVQAIFPYVAGGLATSSDHALWTLTYFIVNWSLGITLMPWTTARFGMRRVFLTATGVAAAGSVISGMTHNLWIMLLSRTLEGLAAGLLVPLSQSLFLRHSPKSKHALVTVFWSNAMLVPFFFGPAIGGWLATGPGFRWIFWLSLPLWLLAAVLGGRAIPAGGGDPSLPAFDLAGFVLLYAGLMGLQITLDNGEQYGWWHSPLILSSSVFALIAFVLFAWRESEARYPLLRFHYLRQRNYWLGLSLLCLGWAMFMGWAAALPLWVEQNLGYNGYWGSIVLVPIAIGAIPVSMVMDRLRSLVGLRRLATLCFLLFAASYGNFTLSPISSLGDTVLPMLFMGLAVGSLFVPLTLILLSEVPAAEIPRAATTSNFIRVFSANIGVSLISVYWTRGSALVATQMRDKIDPYTHSTWPLWQLQHLLEVEAATLSMNNLLRLCMWIALLAALAAYLLIIPPRSIARPDGPHNYVEEEELETAETPAAGELPASTTTS

>637390.5.peg.2638_MFS

MAFAHHAPAVSPLSTSMVLLLNLVIGLGHFLVLFNTGAYLPMIPHVAGSLGVNPDFADWTQADFFLAMALAFPTSPWFLQRWGEMRVLAGAFMAFALASAICAQTGHYDAFLSARIVQGFSGGLTIPVSLQIILRHYQAHRRNIGLGLWGVAALTPFTLGPIIGGWITDSIGWRWLFYLNIPIAISVAVIIVILLFGREMEHRHPPLDWPGLLLLLIALATLGSALNAGEVISWWRSLPIIFLGSISLITLIFFGIWEWYSTHPLLELTLLKRRNFMIGGIVLFFTALFFQGSIAIYIVGFQLVMGYSAWLVGLLILPMAIFSKISFILTQRLLNHLDARILAIFSLLGFAAASFWVASYNHPASFSELLWPQAFVGIFLGSLFPSIIAIALSGLSGPAEIRGTAFLNVLRLCGQAMGIPLIATLFDRRMILHAHFLAEGNRPTIYTLNPSVSNIRTAHYIAHQAAMLAFNEIFYIAAWGFLLGAGLMLFSKPVVYAEPDIRVRRAIEELVDL

>637905.5.peg.2019_MFS

MFRYLLCSFALVLVYPLGIDLYLVGLPDIARDLNASQADLHLAFSIYLAGMASTMLLAGWLADRIGRKPIALMGAATFAIASWYAASSVTVDYFLFARFGQGIGAGFCYVVTFAILRDTLDDDKRAKILTMINGITCIVPVLAPVIGHLILMGFEWPSLFISMAIMATLIFQLCLLILKETKPSHIETTHRNMNTSTQSCHRVNQAHDHKANLANEATKKICDEPLGSRLFISRLIMTSLAVTAILTYVNTSPMLLMEQMGYSTGKYSAAMAGLAVISMTSSFLAPKLLTHFGQQRIMLASQGLYICSAVVFMAGYQFELDSRVNLLGISLICAGFSLGFGTAMSQALSPFSRRAGMASSVLGIFQIACSAAYITAMGWLGISTLNMLIFLLLTTGLTSIILLHVVPSDTASSDKLTHKVSELNSNDKVPASS

>661478.3.peg.216_MFS

MSAVAAPISAAPALPAKSLRWMIAVSVSLAALLEVIDTSIVNVALTDMQATLGATLSEIGWVVTGYGIANVVMIPLSAWLGDAFGKKRYFVFSMIGFTVASIMCGMATTLPVLIGARIFQGLMGGGLLAKAQAFLFESFPKEEQGMAQALFGACVIAGPAIGPTLGGWLVTNFSWPWIFYINLPVGIAATLMCIAYLPEDVKRFGRKAVDYLGIVLLILWVGSLQILLEQGYENDWFDSRFISVLAVISSVGLVLWIWRELRTKAPAVDLRVLRHRSLTAGSVYAFVVGVGLYGALFAIPIFAQQVLGYTAYQTGMLLLPGAIASALMMPVMGRLSKVDARVLIALGSLVLIGSLVVVSRISILTGPEDLFWPLVFRGIGTVMIFLPLSLATFSGVPKEEVSAASGFYNLTRQLGGSVGIAVLTTILAQREAFHRSNLVEYVSAYSSTAAERLSALTGGFVARGAAPATARTMALKAMDRSVDVQAAVLSFGDMFHIVAFLFVVSLGLLFLMGSGKRSAAPVDVH

>66429.3.peg.1684_MFS

MNKAGQTEQPTAPETPLLPDAPEPDPKRWLALTVLLVATFMDLLDSNIITVAIPSIQRDLGASAVAVQAMTAGYTLSFAVLLITGGRLGDIFGRKRMFLTGVAGFVLSSALCAAAQNTEMLVASRALQGLTAGIMVPQVLALIHVSFAPQEIGRVVSLYASMIGLAVVSGPVVGGALVEWSPLDLGWRSIFVVNLPIGVAALAGAGKWMRESRSPHAQRLDIVGMLLAIVGLLLLMLPLTLGRELGWPVWSIVALVAALPVIALFVVHQRLKTRKDGSPLVSLSLFKVRAFSAGIGVQLLFSAVPAGFFLSWTLYLQGGLGWTALHTGLTAIPFSVCVPLVGGLAVRKLSPLYGRYCLVAGALSMLAGIASYAWAADRLGADITSWHAVPSMILLGSGMGLLMPPLTALVLREVKPQEAGAASGIINATGQLGAALGVAIIGGIFFSALAGNAGPQADRVLPAHRTVAARQAAEVKDCATDSLGQDDLTKVPRSCAALAGKSDPESMSAIGSALGEIRTKTFVATYSDTLYWAAAGLVPVAGLLFLLPHHRVRRGETA

>665792.3.peg.2625_MFS

MEKTMEAAAAPQEGASFKVVPIMTALLLAGFIGMFSETALNIALNELMSTFNVEPATIQWLTTGFLLVLAILVPISGLLLQWFTTRQLFAASLVFSIAGTLIAASAPSFAFLFIARLVQAVGTGLLIPLMFNTVLVIFPPHKRGAAMGMMGLVIMFAPAVGPAIAGLFLEYASWRTIFWTALPLLVVALLFGLMFMKNVSELARPRIDIYSIALSSFGFGGVVYGFSSAGEGDHGWSSPKVIIGIAVGIVALILFTVRQLRMKQPMMNLRAFRFPMFTLGTLMIFIGMMIILSTVILLPLYLQAGIGLLPLAAGLLLLPGGLINGVMSPIMGRLFDKYGPRWLVLPGLVLVFVVLWLLTGINTGTSKGEIILLHSLLMIGISMIMMPAQTNGLNQLPRELYPDGTAIMNTLQQVAGAIGTALAISIMTAGSKAYYADGKHSPADLSTVPAAMTQGVQNAFLFVMIFAVLGFVCALFIKRVKVGKQEQVHHAGH

>703.8.peg.1654_MFS

MIILYPVGIDLYLVAVPHIADSLHADDAQIHTAFSIYLFGMAATVLIGGVIADRYGRRRVVLAGALLFVIASLVAATATHIYGFYFGRFWQGAGAGTLYIMSFTILRDVLSQERLASALAMINGVICVIPVLAPVLGYIILSHSSWRGIFITMASIAICCGLINLVLLKETRPVTPHQRGLSTSFAVLRAPRFMLLSLLTSASVTNILVYVSVSPLLLMKQLGFTAEQYSIVMMVMAGVSMATSFLTPLLLRCFGSHNVLAFSHLAYLLALLSLIGSWHLNGNIELLLLAFSLICIGFSCGFGIAMGDALNECQQDNVAFASAILCIMQISLSGLYIWLMGYLEFTPSEMLMYSLLVSLLSYLAVKVLVPWFMVNHPNQLRG

>745310.14.peg.3050_MFS

MSTAAASASAPPAPASPALPSPAKRLAITITVMAGTLMQVLDSTIANVALPHMQASLGATQESIAWVLTSYIIAVAIATPVTGWMESRFGRRELFVASVVGFTLASAACGLAPTLETMVAARVLQGVFGAFIGPLTQAIMLDSYPREKHAQALTIWGMGVMIAPIMGPVLGGWLTDQWNWRWVFFINVPFGIVTTIASWLLLSSSRLEKTRLDITGFILISLFLVGLQLVLDRGTHLDWFDSREIVIEAALAVAALWMYVIHSATTARPLIPLALFRDRNFLIANLFMFVASGVSIAGSALTAPMLQTLLGYDAYGAGILVAPRGLAMMVSMLATSFVTKYVDGRVVIAIGLVLVAVSQMMMSGFDLEMGSRPIIFAALIQGLGLGMFVLPLNLLAFATLAPYLRTEGAALYSLSRNMGSSIAISILSALLARNTQVSHSDLAAHVSASSLPFLTPGTLERFGQQGHDILRMVDAEVNRQALMIAYIDDYWLMGWAVAVLLPFVVLMRGVGRKAGDPPPPMME

>754436.4.peg.3313_MFS

MFRYLLCSFAFVLLYPTAIDLYLVGLPQIAQDLGASESQLHIAFSVYLAGMAATMLFAGTLADRIGRKPVAMVGAAIFAMASWLGGQVDSSTPFLLARFAQGIGAGACYVVAFAVLRDTLDDQRRAKVLSMLNGITCIIPVIAPVVGHLIMLYFPWPTLFTTMAAMGVLVCLLATGVLKETNPNRRHRTDRVLEKQTETDTQETFTTPYFISRMLISSLAVTVILTFVNVSPTVLMNGMGFSRGEYASTMALTALVSMITSFATPFAMAWVAQRTLQLLSQGLFVLAAILLASASLYDLHNMVTLVGLGMICAGFSVGFGVTMSQALSPYARRAGMASSLLGIAQVCSSACFIWLMGLCDVTGLAMLIGILLTTGIINAALLLCVATPPRLSQQAPQKPQQQSPQAAEPHEEISCSS

>82380.11.peg.1417_MFS

MPPAYREADTKLALHQNWYGYTFGMTSLTSSIRNANRAWIMLVVLTMLTVIGMTVVLPVLPFVVLQYVSEEKDLALWVGVLEAVNGLCAFLIAPFLGRLSDRFGRRPVIIAAAFGAAFAMALFGIGCALWVLVLARVIQGLTAGDLPALFAYLADITPPEKRAQRFGLLGALSGIGMMIGPAIGGLLASVSLQLPVFLTAAVGLTIAILSIFLLPESLKPENRITSISVRDVQPFAVFKNAFGRKELRGLMIGFGLLALPFGFFVNNFSVLALDSIQWGPTQIGLMTAAVGIIDILIQGVLLGILLPRIGERGVIVSGIVAQMVGLIGLAVVASIFAQPWLFIVGALMLAAGQGASQAAMDGAMSNAVGDDEQGWLGGATQSLNAAMGTIAPLIAGALYVAVSHSAPYWLGAALMVVAVIVVARAHIVNTAKVGSAKVTATDAPLELLDARD

>83332.12.peg.2605_MFS

MNRTQLLTLIATGLGLFMIFLDALIVNVALPDIQRSFAVGEDGLQWVVASYSLGMAVFIMSAATLADLDGRRRWYLIGVSLFTLGSIACGLAPSIAVLTTARGAQGLGAAAVSVTSLALVSAAFPEAKEKARAIGIWTAIASIGTTTGPTLGGLLVDQWGWRSIFYVNLPMGALVLFLTLCYVEESCNERARRFDLSGQLLFIVAVGALVYAVIEGPQIGWTSVQTIVMLWTAAVGCALFVWLERRSSNPMMDLTLFRDTSYALAIATICTVFFAVYGMLLLTTQFLQNVRGYTPSVTGLMILPFSAAVAIVSPLVGHLVGRIGARVPILAGLCMLMLGLLMLIFSEHRSSALVLVGLGLCGSGVALCLTPITTVAMTAVPAERAGMASGIMSAQRAIGSTIGFAVLGSVLAAWLSATLEPHLERAVPDPVQRHVLAEIIIDSANPRAHVGGIVPRRHIEHRDPVAIAEEDFIEGIRVALLVATATLAVVFLAGWRWFPRDVHTAGSDLSERLPTAMTVECAVSHMPGATWCRLWPA

>926569.3.peg.1825_MFS

MIKKTSARLVYLILSGGNTLADTIMFTVNMVYFVEIIGLSPLQLVLVGTVLEGAILLFEIPTGVLADTIGRKVSIVTGWFIMAGGFLLVGIVPELWAVFIGQVLWGLGYTFTSGATEAWLADEIGEDLVGKINIESGQINRILGLIGSAISVAIASVALNLPIVIGGLMYLFLAVFLLFTMPETQFTPRYKSSKSLETPFQSFIQTFQEGVKAVGKSPILLALLLVELFIGAASEGYDRLSSAHLLKNFQIPPIGALQPVVWFGILNITGSLASFSTTAMFRKKLEVISQSYQLAARYLVLLHSLGIAMVVMLALTGNFYAAIAAILVKGVMGALIFPLYNAWLVQNILPTTRATVISIVGQANAFGQVVGGPGIGAVGNRSLRLAILLTALLSIPALPLYTSAQKKQTVFSPESTR

>95606.3.peg.1781_MFS

MLTSYLVANAVIVPISGWLSDVIGRKRFYMISVLLFSIASLMCGLAPSLGFLVISRILQGIGGGGLAPSEQSFLADTFPPSKRGMAFAAYGVVVVIAPVLGPSIGGWITDNISWHWIFLINVPVGAISLVLVHFLVVEPKALEKERKKKLRKGLNVDAIGFALVALGLGCLEVFMDRGQRDDWFGSGFITSMAIIAVISLVLLVVWELNQKEPIVDLKLLGVPNFAICFVMMLGVGVIIYGSTQLIPQLLQEVFGYTATDAGLALTLGGAAALLAMPLVGALSGEIQGRWFLGWAFFMQAASMWYFTGINADVSFDHIAVGRLIQAIAIPALFVPINAQAYAGLQPNRYNHASALMNVARNLGGSIGISTAQALLLQREQFHQSRIVESLNPLDPNYVEGLKQIGASLGGAKGGDADQSQLAALYQMATKQAAMISYIDVFHVLAVVMILMVPLSILLKPAKGEH

>P76242_MFS

MTCSTSLSGKNRIVLIAGILMIATTLRVTFTGAAPLLDTIRSAYSLTTAQTGLLTTLPLLAFALISPLAAPVARRFGMERSLFAALLLICAGIAIRSLPSPYLLFGGTAVIGGGIALGNVLLPGLIKRDFPHSVARLTGAYSLTMGAAAALGSAMVVPLALNGFGWQGALLMLMCFPLLALFLWLPQWRSQQHANLSTSRALHTRGIWRSPLAWQVTLFLGINSLVYYVIIGWLPAILISHGYSEAQAGSLHGLLQLATAAPGLLIPLFLHHVKDQRGIAAFVALMCAVGAVGLCFMPAHAITWTLLFGFGSGATMILGLTFIGLRASSAHQAAALSGMAQSVGYLLAACGPPLMGKIHDANGNWSVPLMGVAILSLLMAIFGLCAGRDKEIR

>P46104_MFS

MSVFARATSLFSRAARTRAADEAARSRSRWVTLVFLAVLQLLIAVDVTVVNIALPAIRDSFHVDTRQLTWVVTGYTVVGGGLLMVGGRIADLFGRRRTLLFGAFLFGASSLAAGLAPNLELLVLARFGQGAGEALSLPAAMSLIACSSRTAPFQGVERLASVASVGLVLGFLLSGVITQLFSWRWIFLINIPLVSLVLVAVLLLVKKDETTARNPVDLPGALLFTAAPLLLIFGVNELGEDEPRLPLAVGSLLAAAVCAAAFVAVERRTAHPLVPLTFFGNRVRLVANGATVLLSAALSTSFFLLTMHLQEERDLSPIEAGLSFLPLGLSLILACVLVRGLIERIGTTGAAVLGMALAGPRHRLFALLPSDNSLLTSVFPGMILLLRMATGLVALQNAALHAVTEADAGVASGVQRCADQLGGASGIAVYVSIGFSPHLGGDWDPFTVAYSLAGIGLIAAVLAVLALSPDRRLAAPREQED

>P46105_MFS

MSSVEADEPDRATAPPSALLPEDGPGPDGTAAGPPPYARRWAALGVILGAEIMDLLDGTVMNVAAPAVRADLGGSLSVIQWITVGYTLAFAVLLVVGGRLGDIYGRKRMFVVGAVGFTAASVLCSVAAGPEMLTAARFLQGGLGALMIPQGLGLIKQMFPPKETAAAFGAFGPAIGLGAVLGPIVAGFLVDADLFGTGWRSVFLINLPIGVAVIVGAVLLLPEGKAPVRPKFDVVGMALVTSGLTLLIFPLVQGRERGWPAWAFVLMLAGAAVLVGFVAHELRQERRGGATLIELSLLRRSRYAAGLAVALVFFTGVSGMSLLLALHLQIGLGFSPTRAALTMTPWSVFLVVGAILTGAVLGSKFGRKALHGGLVVLALGVLIMLLTIGDQAGGLTSWELVPGIAVAGLGMGIMIGLLFDIALADVDKQEAGTASGVLTAVQQLGFTVGVAVLGTLFFGLLGSQATASVDDGASRARTELAAAGASTTEQDRLLADLRVCLRESASQQDSERTPDSCRNLQQARPAVAEATARAWRTAHTENFSTAMVRTLWVVIALLAVSFALAFRLPPKPREEEGF

>D0ZXQ3_MFS

MFRQWLTLVIIVLVYIPVAIDATVLHVAAPTLSMTLGASGNELLWIIDIYSLVMAGMVLPMGALGDRIGFKRLLMLGGTLFGLASLAAAFSHTASWLIATRVLLAIGAAMIVPATLAGIRATFCEEKHRNMALGVWAAVGSGGAAFGPLIGGILLEHFYWGSVFLINVPIVLVVMGLTARYVPRQAGRRDQPLNLGHAVMLIIAILLLVYSAKTALKGHLSLWVISFTLLTGALLLGLFIRTQLATSRPMIDMRLFTHRIILSGVVMAMTAMITLVGFELLMAQELQFVHGLSPYEAGVFMLPVMVASGFSGPIAGVLVSRLGLRLVATGGMALSALSFYGLAMTDFSTQQWQAWGLMALLGFSAASALLASTSAIMAAAPAEKAAAAGAIETMAYELGAGLGIAIFGLLLSRSFSASIRLPAGLEAQEIARASSSMGEAVQLANSLPPTQGQAILDAARHAFIWSHSVALSSAGSMLLLLAVGMWFSLAKAQRR

>Q99S97_MFS

MRLKSIITVIALILIMFMSAIESSIISLALPTIKQDLNAGNLISLIFTAYFIALVIANPIVGELLSRFKIIYVAIAGLLLFSIGSFMCGLSTNFTMLIISRVIQGFGSGVLMSLSQIVPKLAFEIPLRYKIMGIVGSVWGISSIIGPLLGGGILEFATWHWLFYINIPIAIIAIILVIWTFHFPEEETVAKSKFDTKGLTLFYVFIGLIMFALLNQQLLLLNFLSFILAIVVAMCLFKVEKHVSSPFLPVVEFNRSITLVFITDLLTAICLMGFNLYIPVYLQEQLGLSPLQSGLVIFPLSVAWITLNFNLHRIEAKLSRKVIYLLSFTLLLVSSIIISFGIKLPVLIAFVLILAGLSFGYIYTKDSVIVQEETSPLQMKKMMSFYGLTKNLGASIGSTIMGYLYAIQSGIFGPNLHNVLSAVAVISIGLIVLWVVFFKEQSSQSKE

>P96712_MFS

MDTTTAKQASTKFVVLGLLLGILMSAMDNTIVATAMGNIVADLGSFDKFAWVTASYMVAVMAGMPIYGKLSDMYGRKRFFLFGLIFFLIGSALCGIAQTMNQLIIFRAIQGIGGGALLPIAFTIIFDLFPPEKRGKMSGMFGAVFGLSSVLGPLLGAIITDSISWHWVFYINVPIGALSLFFIIRYYKESLEHRKQKIDWGGAITLVVSIVCLMFALELGGKTYDWNSIQIIGLFIVFAVFFIAFFIVERKAEEPIISFWMFKNRLFATAQILAFLYGGTFIILAVFIPIFVQAVYGSSATSAGFILTPMMIGSVIGSMIGGIFQTKASFRNLMLISVIAFFIGMLLLSNMTPDTARVWLTVFMMISGFGVGFNFSLLPAASMNDLEPRFRGTANSTNSFLRSFGMTLGVTIFGTVQTNVFTNKLNDAFSGMKGSAGSGAAQNIGDPQEIFQAGTRSQIPDAILNRIIDAMSSSITYVFLLALIPIVLAAVTILFMGKARVKTTAEMTKKAN

>P11545_MFS

MTTVRTGGAQTAEVPAGGRRDVPSGVKITALATGFVMATLDVTVVNVAGATIQESLDTTLTQLTWIVDGYVLTFASLLMLAGGLANRIGAKTVYLWGMGVFFLASLACALAPTAETLIAARLVQGAGAALFMPSSLSLLVFSFPEKRQRTRMLGLWSAIVATSSGLGPTVGGLMVSAFGWESIFLLNLPIGAIGMAMTYRYIAATESRATRLAVPGHLLWIVALAAVSFALIEGPQLGWTAGPVLTAYAVAVTAAALLALREHRVTNPVMPWQLFRGPGFTGANLVGFLFNFALFGSTFMLGLYFQHARGATPFQAGLELLPMTIFFPVANIVYARISARFSNGTLLTAFLLLAGAASLSMVTITASTPYWVVAVAVGVANIGAGIISPGMTAALVDAAGPENANVAGSVLNANRQIGSLVGIAAMGVVLHSTSDWDHGAAISFLAVGLAYLLGGLSAWRLIARPERRSAVTAAT

>P39642_MFS

MKQLKPNSKYLLYGQALSFMGDYCVLPALLILSTYYHDYWVTSGVIVVRSIPMVFQPFLGVLVDRLDRIKIMLWTDIIRGIIFLGLTFLPKGEYPLIFLALLFITYGSGVFFNPARLAVMSSLESDIKSINTLFAKATTISIIVGAAAGGLFLLGGSVELAVAFNGVTYLVSAFFISRIKLQFVPIQSENIKEAFQSFKEGLKEIKTNSFVLNAMFTMITMALLWGVVYSYFPIVSRFLGDGEIGNFILTFCIGFGGFIGAALVSKWGFNNNRGLTYFTVLSIVSLALFLFTPIFAVSVIAAILFFIAMEYGEVLAKVKVQENAANQIQGRIFSVAEASIGLCISIGSMFINILSAPVIMGLIVVIVCGLFLHTKLVNKSFLERDNKTEQKGVF

>P42670_MFS

MARKPDISAVPVESAACQGPDPRRWWGLVVILAAQLLVVLDGTVVNIALPSVQRDLGMSDTSRQWVITAYTLAFGGLLLLGGRVADAFGRRRIFAVGILGFGLASLLGGAAPDPGTLFLARALQGVFAAALAPAALALINTLFTEPGERGKAFGVYGAVSGGGAAVGLLAGGLLTEYLDWRWCLYVNAPVALLALLGCRLLPRDRRTGRAVRLDLPGTLLGCGGLVAIVYAFAEAESGWGDPLVVRLLVLGVLMLVAFALVERRVQDPLLPPGVVAHRVRGGSFLVVGLPQIGLFGLFLFLTYYLQGILDYSPVLTGVAFLPLGLGIAVGSSLIAARLLPRTRPRTLIVGALLAAAAGMALLTRLEPDTPQVYLTHLLPAQILIGLGIGCMMMPAMHTATARVAPHEAGAAAAVVNSAQQVGGALGVALLNTVSTGATAAYLADHGTSPAATVDGTVHGYTVAIAFAVGVLLLTAVLAWVLIDSRTEAADETGSASVTPARPR

>A0QYL8_MFS

MSAPQAAIDTDHADRHGPRRAWAAVGVLALVGTLNYVDRFLPSVLAEPIKHDLELSDTAIGVINGFGFLIVYAVMGIAVARVADRGAFGAVVAGCLTLWGTMTMLGGAVQSGFQLALTRVGVAIGEAGSTPAAHAYVARNFVPQRRSAPLAVITIAIPLASTASLLGGGLLAQSLGWRTAFVIMGAVSVVLAPLVLLVVGVRQSLPAAPAVVDKTAGGWWNLLRKPSFLIVVAGTAFISAAGYSLTTFSPAFLMRTRGMSLGEVGVEYGLATGAIGVLGLLIVGRLADRLAERDPRWLLWIVVTLTLVLLPASVLAFVVEDRMLCVLFLALSYAIGTSYLAPSIAAIQRLVLPEQRATASAMFLFFNAVFGSVGPFVVGMLSDSLTDDLGAQALGRALLLLVAAMQLVGAICYWLASARYRRDIIEEAR

>A0QWU7_MFS

MSSRGNRNIAISAGSLAVLLGALDTYVVITIIVDIMADVGIAINQIQQVTPIITGYLLGYIAAMPLLGRASDRFGRKMLIQVGLAGFAVGSVVTALSSDLTMLVIGRIIQGSASGALLPVTLALAADLWSARSRASVLGGVGAAQELGAVLGPMYGIALVWLFNHWQAVFWVNVPLAVIAMVMIHFSLPARQQVDEPERVDVIGGVLLAIALGLTVVGLYNPEPDGKQVLPSWGLPVLAGALVAAVAFFAWEKVAKTRLIDPAGVRFRPFLAALAASLCAGAALMVTLVNVELFGQGVLGQDQDHAAFLLLRFLIALPIGALIGGWLATRIGDRLVVLIGLLIAAGGFVLISHWSVDVLADRHNLGLFTLPVLDTDLAIVGLGLGLVIGPLTSATLRAVPAAEHGIASAAVVVARMIGMLIGIAALGAWGFYRFNQHLATLAARAAGDAGSPMSLAERLTAQAVRYREAYVMMYGDIFLSAAVVCVIGALLGLLISGKHEHAEEFEPAYAPTYGGGGAIDPYDAGDADDAPTEMLDLPTQVLSAPPSDPGDERPGRHRAP

>A9MWE8_MFS

MNENIAEKFRADGVARPNWSAVFAVAFCVACLITVEFLPVSLLTPMAQDLGISEGVAGQSVTVTAFVAMFSSLFITQIIQATDRRYIVILFAVLLTASCLMVSFANSFTLLLLGRACLGLALGGFWAMSASLTMRLVPARTVPKALSVIFGAVSIALVIAAPLGSFLGGIIGWRNVFNAAAVMGVLCVIWVVKSLPSLPGEPSHQKQNMFSLLQRPGVMAGMIAIFMSFAGQFAFFTYIRPVYMNLAGFDVDGLTLVLLSFGIASFVGTSFSSYVLKRSVKLALAGAPLLLALSALTLIVWGSDKTVAAVIAIIWGLAFALVPVGWSTWITRSLADQAEKAGSIQVAVIQLANTCGAAVGGYALDNFGLLSPLALSGGLMLLTALVVAAKVRITPMS

>P45123_MFS

MNQQKSTFIFILTLGILSMLPPFGVDMYLPSFLEIAKDLDVSPEQVQHTLTSFAYGMAFGQLFWGPFGDSFGRKPIILLGVIVGALTALVLTEINSVGNFTALRFVQGFFGAAPVVLSGALLRDLFSKDQLSKVMSTITLVFMLAPLVAPIIGGYIVKFFHWHAIFYVISLVGLLAAALVFFIIPETHKKENRIPLRLNIIARNFLLLWKQKEVLGYMFAASFSFGGLFAFVTAGSIVYIGIYGVPVDQFGYFFMMNIVTMIFASFLNSRFVTKVGAETMLRIALAIQFLSGMWLILTALLDLGFWPMAIGVAFFVGPNPVISSNAMASALERCPQMAGTANSLIGSVRFAVGAIMGSLVASMKMDTAAPMLFTMGACVVISVLAYYFLTSRNLKSRG

>C5BC70_MFS

MQNHLSSTRRLGRRALLFPLCLVLYEFATYIGNDMIQPGMLSVVQTFGVDESWVPTSMTAYLAGGMFLQWLLGPLSDRIGRRPVMLIGTLYFAATCLAILLTNSIEQFTLMRFLQGISLCFIGAVGYAAIQESFEESVCIKITALMANVALIAPLLGPLAGAAWVHLFPWEGMFILFAALSLLAFLGLYKAMPETATRRGEKLSLSALGRDYTLVLKNRRFLCGSLACGFASLPLLAWIAQSPVIIISGEGLSSYDYGMLQVPIFGMLILGNLTLARLSGRRPVRRLIQLGAWPMVGGLAIAAASTLYSAHAYLWMTAGLSLYAFGIGLANAGLYRLTLFSSTMSKGTVSAAMGMISMFIYTLGIEVGKYAWLLGGNGAFNLFNLISGLLWLALIARMLRDQLVGRMAGR

1. **Resistance-nodulation and cell division (RND): 47 protein sequences**

>757424.7.peg.1547_RND

MTNTPNPHPPSPSNASARGSILRRWWFWVLVAALAAGGGYKMWSKKKAEQEQMAAMGGPGGRPGPGAAGARRPGGPGAFGPQTMPVGVAKARLQDVNVFLNGLGAVTPTATATVRARVDGQLMKLHYKEGQVVKAGDLLAEIDPRSLQAALTQAEGQLARDRALLASARLDLKRYQTLLAQDSIASQQVDTQVALVKQYEGTVKADEGNVASARLQLSFTRVTAPISGRLGLRQADVGNNVTTSDTNGLVIITQLQPITAIFSIPEDNIPKVLQQLQSGRKLPAQAWDREQKNKLADGVLLTIDNVVDATTGTVKLKAQFPNTDYALFPSQFVNIRLQLNTEQGATVIPTAAIQRGSKGLFVYVVKDDSSVTVRPVKTGPVQDDLTVITDGVSAGETVVIDGIDRLREGAKVEAVARGGADDPANKLTTENPERRHGKRGQGNPGAQAGAGGDAGQGAQGGMSPEERQKRWAELNKRIDAGEFGEEIKKLPEDQRRQKMMELRRQREAAGNGNGNAAK

>757424.7.peg.2067_RND

MKLAQLRRPKFIFLALLVLLIAAWIIRSVLTPPAPPTYLSATARVADIQDVVLASGTVKAYKQVSVGAQVSGQIKSLKVALGDQVKKGQLVAEIDSLTQANALASAEFSLQNLQAQLRAKEASLKQAQLAYARQKMMLAGDASSRENFESAEATLNTTQADIAALQAQIKDGAIKVDTARLNLGYTRISSPIEGQVVAIVAQEGQTVNANQSTPTIIKVARMDTVTIKAQISEADVVRVKPGQPVFFTILGDPDHRYRTTLRAIEPAPDSILQDDTSSSTTSITSSSSASSTAIYYNGLLDVPNPDGKLRISMTTQVNIVLSEASNALVLPSTALGAKAADGSYTVRVLDDQGQAHERKVRIGINTNALVQIVEGVKAGERVVTGTVLPGAAASSSAHDGPPPHM

>757424.7.peg.2754_RND

MNRINSSAFRLRAIAAVAALAGVLGLSGCASFAGIGSDRQVAQAGDFATQRSLSDPNPGAPNGQWPGSDWVRQFGDAQLVALVEQALTSSPSLQQARARIAAASALAESRGAPLLPSVNAEASVTRNQFSSTTIYPPPYGGNWYNEKKAGLNVGYELDLWNKNQAALAQAISSEKAAQASEQEARLALTASIVTVYSQLAAQYALHDILQSTVDQRTSLEKITAERLRTGLDSQIERDQSRTSSADARAQLAQSEGQIVLLRQQLGALAGKGPDYGLQLAPPALQGLATPGLPAELPLNLMGRRPDIVAARWQVEAASRGVDVAKARFYPDINLSAMIGFDTLLDSNPFTAASKSIAFGPAITLPIFEGGALRAGLKGEYASYELAVATYNKTLNDAYADVARQIAAIHATERQLPIRSEALQAAERAYALARERYRLGLVSQLTLLSAQTGVLAQRQAMVALQAQRRDQQVALYKALGGGFDAQRDGLAYGAQP

>757424.7.peg.3310_RND

MAGCASFSDLGERAQPKSIDRYQSQQSLAASAVQAAWPSDQWWRVYGDAQLNALIDEALQSAPSMAVAKARLMKAEGAAQQQGAALYPQVSANASLDRMKQSYNNGVPPDFVPKDYNNATRATLDFSYEIDFWGKNRAALAAATSELEASRADAAQARITLATSIASAYAELAQLYAQRDTNEAALKVRVESLDLFNQRFTNGLETRGSVKQMEARRAIAQADLKATDESIGLQRNKLAALLGAGPDRGLQLTRPQIDLSRPFALPAQLPVELLGRRPDIVAARLRAEAAGKQIKVARAAFYPNVNLTAYFGFQSLGIDMLTRAGSDIGSIGPAISLPIFNGGRLRGQFRSASASYDEAVANYDQAVTQALQDVADVGVSEKALAGRLADVQAAADAAEEAYRIVSNRYNGGLATYLDVLNAQDTLISNLRQLSDLRSRMFTLDVALVRALGGGYRAADDSSSQNSADAPLHDTQAKG

>1006551.4.peg.3864_RND

MSSAASCPAATNTGIKIMSLQKYWGNFHLTVPGVMLLSALLVGCDEGVAQNAAPQAPAVSAADVVVKSISQWDSFNGRIEAVESVQLRPRVSGYIDKVNYTDGQEVKKGEVLFTIDDRTYRAALEQAQATLARAKTQASLARSEANRTDKLVNTNLVSREEWEQRRAAATQAQADIRAAQAAVDAAQLNLDFTKVTAPIDGRASRALITSGNLVTAGDSASVLTTLVSQKTVYVYFDVDESTYLHYQNLARSGQGASSNHLALPVEIGLVGEEGYPHQGKVDFLDNQLTPSTGTIRMRALLDNAQRQFTPGLFARVRLPGSAEFNATLIDDKAVLTDQDRKYVYVVDKEGKAQRRDITPGRLADGLRIVQQGLKPGDRVIVDGLQKVFMPGMPVNAKTVAMTASTALH

>1069631.3.peg.2211_RND

MLFSLFVTIGLNVYLYAVVPKGLFPQQDTGQLMGFFRVDRGTSFQSMVPKLEYFRSILNQDPDIRSVAVFAGGRSGSTSSFILVELKPMDERKASTTDVVNRLREPLSTTPGARMFMVPQQDIPVGSGGGGRSGSYDYSLLGSDLELLKTWLPKVQQAMAELPELVDVDTGTDDKAGLVQLEIDRDMATRLGIDMSMVAGTLNNSFSQRQVSTIFGRLNQYYVVMEVEPRFAQDLESLKEIEVVAKDGTRVPLSAFTRFTTGTAPRSINHMGLLVAESVSFGLAEGVTLSQATAAIEQAMARIQLPTREIQAGFEGNTAQMLDALAKQPMMFLAAWWLCISCWGCCTRAICTRSPFCPLCLRPGLGHCWL

>1120928.3.peg.3378_RND

MSISRKQLTLSAVIVAIFATGGSFILFQEKADAKATPTASAAPAATVDVANVISQTITDWQEYSGRLEAIDQVDVRPQVSGKLIAVHFKDGSLVNKGDLLFTIDPRPFEAELNRAKAQLASAEAQVTYSSANLGRNQRLIQSNAIAHQELDQAENEARSANANLQAAKAAVETARLNLEYTRITAPVSGRISRAEVTVGNVVSAGNGAQVLTSLVSVSRLYASFDVDEQTYLKYISNQRNSAQVPVYLGLANESGFSREGFISSIDNNLNTTSGTIRVRATFDNPKGVMLPGLYARIRLGGGQPRAAILISPTAIGVDQDKRFVVVVDAKNQTAYREVKLGAQQDGLQIINSGLQVGDRIVVNGLQRIRPGDPVSPHLVSMPNPQIITDNTAQQPQPTEKTPTSAKG

>1154758.3.peg.1457_RND

MIVATVGLFVLSVVMFKFVPQQFFPASGRLELMIDLKLAEGASLTNTAEQVKRLEQMLKDHQGIDNYVAYVGTGSPRFYLPLDQQLPAPSFAQFVVLARSIEDREAIRGWLISSLNEQFPTLRSRVTRLENGPPVGYPVQFRVTGEHIEVVRALARKVQDRVRENPHVANVHLDWEEPSKVVHLNIDQDRARALGVTTADLSAFLRNSLTGSSVSQFRDDDELIDILLRGTRNEREQLGALSSLAIPTQNGTSVALSQVATLDYGFEEGVIWHRNRLPSVTVRADIYGKGQPATLVKQILPTLDSVRAELPDGYLLEVGGTVEDSARGQNSVNAGMPLFIVVVLTLLMIQLRSFSRMLMVFITAPLAADRRHAVPADLQSAVRLCGHAGHHRAVRNDHAQFGDSGRSDRAGHHRRAGPMARDHRCHRAPLPPDRADCSGRSAGDDTAVTQPVFRPDGRGYHGWSDRRHGADPAVPARAVCRVVQGETGG

>1161913.3.peg.4313_RND

MALGTLVLTVVLYIFIPKGFFPVQDTGVIQGISEATQSVSFGAMAERQQALAKVVLEDPAVESLSSFIGVDGINATLNSGRMLINLKPHESRDISASDVIRRLQPRLNEKVPGITLYMQPVQDLTIEDSVSRTQYQFTLEDADAAELSTWVPKIVDRLRQLPELADVATLHVLDVLDDAVALVVHQHDDHVGLFLHGGRQLTQVEDEAAVAGQREGLLARGGHRCADGGADAHRQALADAAAECMHAGQRIENAQIAIAPGAVRHGDVAHPVELAAGGLLYLLNQRAVGTETVDQAGDGGIARLFQVGHEGRIDVDCALAFFEAIRQAFQRQCSIAADEVVAVVAAAFRRWIGVDAIQRTRQLQFVLQGFVAAQARADHDDGVAGLVEVLDRLVQVE

>1194405.4.peg.3028_RND

MIVSQGGAARDRLIERLRQRFRDDYVGVGGXRPWRWPPFSTPTPISVRSSTTGTNRARC

>1208660.3.peg.3555_RND

MTFTDLFVRRPVLALVVSTLILLLGLRATGELPVRQYPLTENTTITIITQYPGASPELMQGFVTQPIAQAVATVENIDYLSSSSTQGRSLITVRMKLNADSNKALTEIMAKVNQVKYRLPQEIYDPVLAKSSGEATSVAYVGFSSKTMPIPALTDYLQRVVLPQLSSIDGVASVDLYGGQTLAMRVWLDPARMAARGISAGEIAQALRDNNVQAAPGQTKGLYVVSNIQVNTDLNSLTDFRDMVVRQVDGAIVRLGDVGTVELGAASYDSSARMDGEKAVYFGLNATPVGNPLTIVERINALLPGIKQNLPPGVEVQVPFELARFINASIDEVRNTLLEAVLIVVAVIFLCLGSLRAVLVPVVTIPLSMLGAAAIMLSLGFSINLLTLLAMVLAIGLVVDDAIVVVENVHRHIEEGKSPVHAALVGAREVAGPVIAMTFTLAAVYAPIGLMGGLTGSLFKEFAFTLAAAVGVSGVIALTLSPVMSSFLLNSRVSEGWMARKAEHFFQRLGDAYGRVLDVSLRHRWVTGLIAVVVLASLPVLYGSAQRELAPVEDQAMILTAVKSPQHANIDYVEKFGQKWDTVMQEIPEQNGRWLINGSDGVANSIGGVNLVTWQARKRSADEIQGDLQNRVNAIEGSNTFAFQLPSLPGSTGGLPVQMVLMSAADYRVVYDAMETLKHAARASGLFMVVDSDLDYNNPVVRVDIDRAKANSLGVTMKAIGDTLAVLVGENYVNRFGMDGRSYDVIPQSPRGMRLTPQSLGQFYVKSASGAQVPLATLVKISMGVEPNRLTQFDQLNSATFQAIPMPGVTMGDAVQFLTEQARLLPPSFSHDWQSDARQYSQEGSALVVTFLFAIIVIYLVLAAQYESLRDPLIILVSVPMSICGALIPLALGMATINIYTQIGLVTLIGLISKHGILMVEFANEMQAHAGLDRRAAMERAARIRLRPILMTTAAMVVGLVPLLFASGAGAHSRFSLGLVIVVGMLVHPVHPVRAAHHVHPAGARPPRRRPIRARPRTGAAGRPGGRRLPRTRDRIMTHPVPTTFARTAGALLAALALAGCAVGPQYQAPTPAPVKLASPEQALFSADLLQREWWRQLQDARLDALIGLALARNLDIRQAQARLREARAALDEKELDRWPTVTAAGGYTRSLSQINPGPDQRNLAQSYRAGFDATWEIDLFGRLQRRAEAAAARDQAAAADLAQTRLVVVAELARNYFEMRGAEQRLAVARANLATQQETLRVTAALVETGRGYAGDLASARAELAGTRALLAPLETQRRLAQYRIAVLAAMRPAELGELRQEQPLAPLAAQLPIGDVAMLLQRRPDVRAAERLLAATNADVGAITAELYPRIDLGGFLGFIALRGGDLGQASSKAFALAPTISWPALHLGSVQAQLRAGQARHDAARARYEQVALQAIEEVEGALTRYGQNQQRLRDLLDSATQSQRAADLAQTRYREGAAPYLTVLDAQRTLLRAQDAVAQSESESYTSLVALYKALGGGWNTDAAAPARSARTAALPASP

>1218169.3.peg.6920_RND

MRRYNFLSAAGEVKGEYVVTSINASTELKSAEAFAALPVKTSGDSRVLLGDVARVEMGAENYDTVSSFDGTPIGVHRHQGHTSRQPAGRHQGSAAHHARAGKPAALGAEGIDRL

>1224163.3.peg.561_RND

MFRTEADVMRAAAGNVDDTNTSVQGELKRLQNVVDTVRGSWAGTAQVSFDNLMIRYNESARDLHEALASIADNIRSNAVGFEDMEATNAQSFDRVGAQGLAL

>1231351.3.peg.1176_RND

MNLSRPFILRPVATTLLTLGLVISGLLGYSQLPVADLPNVDMPVIMVQAQQPGGSPSEIASTIAEPLERHLGAIAGLTEMTSQSMVNQVRILLQFDLARDVNGAARDVEAALQAARQDLPAGSLRSNPTYQKANPNGAPILVLALTSKTRTPQAIYDFTTNVVQQQLSEIRGVGGMEIGGGALPAVRVELNPLKLYKFGIGFEDVRAALVSANAHTPKGFIEQNGQRFTLDTNDQATQAQAYRNLVIAYRDNAAVRLSDVSIVRDSVENLRTSGYFNGERAVIALVFAQAGANVVQTIDQIKQRFDLIRAALPPDIELHLAVDRSQTIRAALDDTKLTLIIAVVLVVLVVLLFLRSLPAIMIPAIVVPTSIIGTFGAMRLLGYQLDNMSLMALTISTGFVVDDAIVVLENVSRYLEQGVAPVPAALRGAGEVAFTVISITVSLIAVFIPILLLGGLPGRLFHEFAITITLTLVISMGLSLSLTPMICALLLKPMPSGETRGRVSHAIERGLSAVTRGYAASLEWSLHHQWLMVLSLPATLVLAGALFVEMPKGFFPTEDTGLLMGHLVGDETSSFGQMSQRAQLGTRIMAHDRDIANVVGFVGGRQANTANLFSSLKPKSERNDTVLQTIVRITRHFRGMVGTQFYLMQPGAVRAGARGGNGAYQYSLQGPDADELYAWTPKVVAAFQRLPELMDVSSDLDEGGAALDVRIERPTSARVQITPQLISNILYDAYGQRAASVIYRSNNQYRVIMEAAPRFWHDPHSLYQTWISVSGGTAAGGTASNNIRARLTTTTSSGSSDTTSSASSQAAQSYQNQMANSLAGGSNASSGAAVTTSAETMVPLTIVSRITPGVTSLSVNHQGQSVATTVSFNLRPGVSLGPAIAAINAALVKMHMPTEIRGGFAGNAAQFQKSVSAEPLIILAALITVYVTLGVLYESLVHPLTILSTLPSAGVGAILALQVFREEFSLIAMIGVILLIGIVKKNAIMLIDFALQAQRAGSSAYDAIHEASLLRFRPIIMTSLAAALGAVPLIVANGYGSELRRPLGIAILGGLVVSQALTLYTTPAIFLMLERAREATHRAVRSFRRPHQQDIPST

>1267562.4.peg.5000_RND

MPADPTFAASPSPAAPVSSLPCRLRATSLVLLTALTVAACGRGEAPAAARTPEVAYVTLQPQPVTLSTELPGRTVAYRVAEVRPQVDGIILKRLFKEGSEVRQGQQLYQIDPSTYQAAHASAAATLESARQTAQRYERLARERAVSQQEYEQARAAWLTAQAAVDRAAIDLRYTRVLAPISGRIGRSFASEGALATNGQANALATVQQLDPIYVDVTQPSSALLGLRRDLAAGRLEAAGENAARVRLILEDGSEYAEPGRLEFTEVGVDTGTGSVTLRAVFPNPRHELLPGMFVRARMQQGVRPAAMLAPQRGVTRDAKGQATALLVNANDEVELRRIDAERVIGDNWLVSGGLQPGERLIVDGLQFVRPGMKVRALPLAGAAPASAPASAVAAPRRAPPSRSVERSAPMSRFFIERPIFAWVIALVIMLAGALSIGALPVSQYPAIAPPTIAIQVNYPGASAQTVQDTVVQVIEQQLNGLDRLRYISSESNGDGNMTITVTFEQGTNPDIAQVQVQNKLQLATPLLPQEVQQQGIRVTKSVRNFLMIVGVVSSDGSMTREDLANYIVSNIQDPLSRTPGVGDFQVFGAQYAMRIWLDPARLTAYQLTPSDVRAAIQAQNVQVASGQLGGLPSVAGQQLNATVVGKTRLQTPEQFREILLKVNGDGSQVRLKDVAEVGLGGQDYNINAQYNGRPASGIAIRLASGANALDTAKAIRATLGELEPFFPPGMQVVYPYDTTPVISASIEGVVRTLLEAVVLVFLVMYLFLQNVRATLIPTIAVPVVLLGTFGVLAAFGYSINTLTMFGMVLAIGLLVDDAIVVVENVERLMAEEGLPPKEAARRSMGQIQGALVGIALVLSAVFLPMAFFGGSAGVIYRQFSITIVSAMVLSVLVALVLTPALCATMLRPLPKHGDGHGHGAPRRGPLGWFNRGFEAATRGYERGVVAVLNRRGRYFAVYLLILALAAWMFTRIPTSFLPDEDQGVLFAQVQTPPGASAQRTQQVLDRLRDYLLQEEGGVVQSLFTVNGFNFAGRGQSSGFAFVLLKPWHERIGEATSVFDLARRAQARFSGMRDAMAFAFVPPAVMELGNATGFDVYLQDRAGVGRGVLMQARDRFLQLAAQRPELQRVRMNGLNDEPQYRLEIDDEKARALGVSLAEINSTVSIAWGSSYVNDFIDQGRVKRVYLQGRPDARMHPDDLAKWFVRNDRGAMVPFTAFASGSWGHGSPKLQRYNGVAAIQILGEPAPGHSSGEAMAAVEAIMAQMPAGVGHSWSGLSYEERLSGAQAPALYALSLLVVFLCLAALYESWTIPFSVMLIVPLGIVGALAATLLRGLPNDVFFQVGLLTTMGLSAKNAILIVEFAKALHDQGKGIVEAAIEASRMRLRPIVMTSLAFVLGVVPLATSVGAGSGSQHAIGTGVIGGVITATVLAIFWVPLFYVAVHRWFGGRRGTPASEISTRTA

>1343158.3.peg.407_RND

MVFCRLFIDRPVATTLLALAIFLSGMIALPFLPISTMPDMTATSIMVIANQPGSDPQQMATSVSTPLERRLATIADIQTLESVTTRGQTSIFLDFSSSRNINGALRDVQAALHAARSDLPTSTLEADPQAFKLDGDKPIYLLHLTSDQLPRAQLYDLATIRVRPILAQIAGVGRVELFGASNPAVRVELNPYPLYRWGLNPEDVRAALASANAFTPKGFITSGNQRIQLQTNDQATEAAHYRDLIVAYRNGKNPIYLKDIATVRDDVQDVYQNSTLNGKTAITIAVIPQPHANAVEIVNDIVRRLPRLQQALPASAELRTGLDLSLTIRASLADAKQTLVISIFLVVLVIALFFRHMASTLIPAITIPVALSGTLTAMAWFNFSLNILSLMALTIAVGFVIDDAIVVLENIARHMENGMNRYQASIVGTSEIAFTIISISLSLIAVFIPLLCIPGTLGSALHEFALTMAATIAISMVLSLTLTPMLCAHFLTIEPAGGTPAIPERPRYSPLADPVSWLLYGAMRTVRAVETGLYHLTSLYDRSMHWSLRHPIIIGLTLPGSFLLMVGIIILMPKTAIPSMDLAILQGSINGEPSLSFKALTRRMHQVESIIQKDPAVQTVVTFNRTSHTGRIFVTLKAKSMRDSIPVILARLRKAIPQQAGAEAFFWALNNGRQGGGDSNTTGNYRYVLQSDSNGPLYATMPPLLAQLRASGKFRNLSTDAEDLSFFANVLIHRDLEARYNITPQLVQNALFDAYGQSIVSTIHLPLTNHRVVMVVAEPFREYSNTLHHLWLSTSAGTAAGGIASNLIRVRTKGTLSTQASLSRDSVTNSLANKLSGNSSNGAAVSSSQETMIPLDNVASIVKTPMPLSITHHNGYYATTLSFDLAEGTSYDDAISLIHRALVNLHASDSIHGEFTGTTGETTDLMLNALLAFLAAITIMYIALGVLYESLLHPITILSTLPSAGVGGVLGLWASGEQFSLVAIIGVILLTGLVKKNAILVIDFALHIHHHHPDMTAEETIRHASVTRFRPILMTTLAAALGGIPLLMSQGYGCELRRPLGVAILGGMAISQLLTFYTTPAVYLLMEKLKHHSLSLMRRVRAAL

>1385369.3.peg.6651_RND

MTGFNLSEWALRHRSFTWYLIISLTLAGGIAYTRLGREEDPAFAIKTMVVQTVWPGATIDDMIDLVTDPIEKKLEEVSYLDYVKSYTRPGFSVVYVNLKDFTPAGEIPDLWYQVRKKIADMKGTLPQGVQGPAFNDEFGDTFGTVYAFTADGFSYRELKDYAETARAELMRVPDVGKIQFVGIQNEKIYLDFSTRQLAALGIDRNQIVAELQAQNAVAPAGVVQAGDEKVTVRVSGEFTSEESLKAINLRAGGKFYRLADLAQVRRGYADPPSPIFRYNGEPAIGMIISMAAGGNVLDFGKDIQERMRQVEANLPVGINTHLVANQSVVVDHSVAGFTKALKEAVVIVLVVSFISLGIRAGIVVACSIPLVLAMTFIGMEYYGIALQRISLGALIIALGLLVDDAMITVEMMITKLEEGFSLDKAATFAYTSTAFPMLTGTLITVAGFIPIGFAQGGAAEYCFSLFAVVAMALLFSWIVAVMFAPLIGVKVLRPPKPGKGHSGHGEPGRMMRAFRASLRLAMRARYIVIVLTVALFGLSVFGLRFVQQQFFPASDRAELLVNLTLPQTSSIKATEEVVNRFEKVLAADPEIESWSFYIGQGAIRFYLPLDVQLANDYFAQAVVVTKGYDVRDGVRARLEKVLNEDFSDLSTRVSPLEMGPPVGWPIQYRVSGPDVGEVRDAAYRLADTIGANPYTLLINYDWNEPSKVVRVDVEQDKARQLGISSKSLSEALNATVSGAVFTQVRDGIYLIDVVAQASNAERSSIETLRNLQVALQDGRTVPLREVAILRYDLEQPLIWRRERLPTITVQADLVPPLQAPTIVNQLAPVVDELRRSLPPGYSIEVGGTVENSAKGMTSIVAVFPIMIFVMLTILMIQLQSFQKLFLVISVAPLGLIGVVAALAPTGTPLGFVAILGVVALIGMIVRNSVIMIAQIDEHLEAGEHPWDAVINATMHRVRPILLTAAAASLGMIPIAPEVFWGPMAYAIIGGLVVATALTLLFLPALYVAWFRIKEPGHEKTIDGKTPVEAESPGHRPIAGPYGPMPPAGVVSGD

>1439940.3.peg.2461_RND

MRFNLSAWALHNRQIVVYLMLLLAVVGALSYSKLGQSEDPPFTFKAMVIQTQWPGLPPRKCRARSPSASRRS

>1469502.3.peg.806_RND

MNLSAPFIARPVATTLITIAIALAGVLGLETIPVSPLPQIDFPTILVQAVLPGASPETMASTVATPLERKLGLIAGVDEMTSVNSLGMTRISLQFDLHRDIDGAARDVQAAINAARAVLPPMPVNPKYWKVNPANAPVMILSLTSRSMTRGQMYDAASTVLAQRIAQVSGVGQVRINGSALPAVRIDVDIEKLARMGISLESVHAAVAAANVDSPKGIIETGGRSWLIGANDQTTTAAAYRRLIVAYRGDRPVRIGDVATVHDSVENIRNAGATNGRPSVLLLIYRQPGANILDTVGRVNALLPRLRASIPSAIDLNVDMDRTSTIRASLHEASRSLLLAVLLVILVVFAFLRSARAIWIPAVAIPVSLVGSFAAMKLLGYSLNNLTLMALAIATGFVVDDVIVVLENIVRHLEEIPGDLAEPGRSGTIPAGNAFALDRRRDAVRLAALRGVRKVGFTVLSMSLSLIAVFIPILAMDGLIGRIFREFAVTLSVSILISLAISLTTTPMLCAVLLRPGAAGADRARRPTSGAGGGSAIRGLRNAWIRVARSASNGTRAIGSRASIAYERSLDAALRHPRITLLILAATVAANISLYIAIPKGFLPAEDIGLIKGKVQGDQSISFQSMTRKLDRFMAIVQSDPAVARVNGFTGGDEANSGFVFAILKPFRERGEISPEAVIDRLRSRLAKVPGATLYLQPARDLHFGGRPSNAEYQYTLESDNLDDLQTWGARIRQALSRLPELVDVNSDAQDRGLGTAITVDRDSLSRLGLTMSQVDTTLDDAFGQRQVSTIFAPRNQYHVVEEADPRFLQDSASLVALNLIGPTGSPIPLQAFARWETRDAPLVVNHQGSFMATTISFNLAPGVSLGTAAAAIDRTMARIGVPATIHGGFQGTAKLFRNSLAAEPLLGLLALFAVYIVLGILYESLTHPITILSTLPSASIGAMLAMMVFRIPMTVIAFIGVILLIGIVMKNAIMMVDVAIDLERRDRLDPREAIRRACLHRLRPIMMTTTAALFGAMPLALGGGDGAELRQPLGIAIVGGLLFSQVLTLYTTPVVYLTLDRLRIRLLRLRHRDSGPSGGQRIPGL

>1500894.3.peg.2187_RND

MNLSKPFVNRPIATVLLTLGLALAGIGAFFVLPVSPLPQVDFPAISVTANLPGGSPDTMASSVATPLERRLAVIAGVNEITSQSGTGQTRINLQFDLNRQIDAAAREVQAAINASRADLPSTLRQNPTYRKANPSDAPVIILALTSKTRSPGQIYDEVSNLVQQKLAQVKGVGDVEIGGGSLPAVRVDLIPYQMNNYGVSAEDIRAAIQATNPNRPKGELEGQGQRLQIYSQVNTPTGGRTAADYKGLVVAWRNGAAVRLQDIAEVSDGVEDIHTLGLFNGRPAIIVLVTSQPGANVIETVDGVRALLPQLQAQLPEDVTMRVASDRTNSIRASLREIEFTLMISIALVVLVVSVFLRSVRATVVPAVATVVSLLGTFGVMYLLGFSLNNLSLMALTVATGFVVDDAIVVLENTSRHVEEGMDKVKAALLGAQEVGFTVLSISLSLIAVFIPLLFMGGQVGRLFREFAVTLSVAVMISLVISLTTTPMLCALLLKGDKEDHKHQLKREQSRIGRFFERGFSVVMKSYEHALDWALDSKPLVMLILLFVVGLNVYLFAAAPKGFFPQQDTGQVAGGMRADQSISFQAMQGKLRQLVNIITSDPAVDTVVGFTGGSRAGGGFMFLNLKPVGERAKGESGQAVIARLRPKLAHVTGVQLFLNPVQDLRMGGRQSNSTYQYTLKSDSSADLKKWATRLADAMKAQKGLTDVDTDQADNGVETYVDIDTATAARLGISARDVDNAMYDAFGQRQVANIYDELNQYHVIMGVAQRYAQSPNALNDVYVPVSSAGAPGTTGTTSGTGSATQGTGGTGTSTASGAGSVTSSVSASVSTSASTSVSSGSAGSAAPATQNAGGAGAGNGSALGTLGAGAASSGGGGSPGSTTNLTAARDPSSGSALSTSAKTMVPLTTMARFSERSTPSSVNHQDGVVATTISFNLAPGVSLSQAQDQVRAAEAQIGMPTNVRGSFEGQAKQAQESNQQQPLLILAAIVVIYIVLGILYESLVHPLTVLSTLPSAGVGAVLALLMFHMEFSIIALIGIFLLIGIVKKNAILIIDFALDAERARGLSATEAVREACLLRFRPILMTTLAAALGALPLAIGFGEGSELRQPLGIAIIGGLIASQLLTLLTTPVVYVYLDKLRTKKPDEHELARQPVEHPSSVPSHS

>1502724.3.peg.3508_RND

MLFAPLIGVAQLPKVMQGHADKKPSRISGWFRQSLAIAMQFRWATIAFTVALFAVALFGLICVVAALLPTGTPLGFVALLGVLALAGIIIRNAAILIGQINDNLRDG

>1736280.3.peg.4105_RND

MSALSPSRPFIERPVATALLMVAIVLAGLLGFRLLPLSALPEVDYPTIQVQTLYPGASPEVMSRTVSAPLERQFGQMPGLARMASTSAAGVSIVTLQFNLGLALDVAEQQVQAAINAGASLLPTDLPAPPVYAKVNPADAPVLTLAISSETLPLTEVQNLVNTRLAQKISQVPGVGLVTLAGGQRPAVRIQADTKALASYGLGLDTLRTAISAANANSAKGSFDGPQRAYNINANDQLVTADDYQRLIVTWKNGAPVRLSDVARVVDAPENNRLGAWAGTTEPPPGRPKAASAPPGGSEPREAGSVGATEPPPGRPKAASAPSGGSEPREAGSVGATEPPPGRPKAASAPLGGSEPREAGSVGATEPPPGRPKAASAPSGGSEPREAGSVGATEPPPGRPKAASARSGGSEPREAGSVGATEPPPGRPKAASAPPGGSEPSVAGSVGATLTPAIILNVQRQPGANVIATVDGIKRQLPELQAQLPASIQVQVLSDRTTGIRASVEHVQMELVLAVLMVVLVIFFFLHSLRATVIASLAVPISLIGTCGVMYLLGYSLNNLSLMALTIATGFVVDDAIVMIENIARYIEEGEPPFQAALKGATQIGFTIISLTVSLIAVLIPLLFMSDVVGRLFREFAVTLALTILISAVVSLTLVPMMSARWLKAEPAHGSQRGWAGAVQRGFDRVIGRYDGWLQWVLRHQRATLVVAVLTMALTALLYVLIPKGLFPTQDTGQLQARLQASQEVSYARMSELQQAAAQAILQDAEVQSLSSFVGVDAANNTMLNAGRMLINLKPGHDAQAEVMQRLRDRVAGVAGVTLFLQPTQDLTIDTETGPTEYRASIGGVEAAQVNGWTQKLVERLKTVPEVRNATTDAGAQGLSAYVDIDRNTASRLSVTASAVDDALYSAFGQRIVSTIFTETNQYRVILEAQQEQLGSLEGLGTLPLRTGSAAPTPLAAVATIREQLAPLQVTRVAQYPAATLGFDTAPGVSLGRAVSAIRAAAQEIGMPAGLSMEFLGAASAYEKSLTSQLWLILAAMVCVYIVLGVLYESYVHPLTILSTLPSAGVGALLALMLTGNDLGVIGIIGIILLIGIVKKNAIMMIDFAIDAERHQGMGPQQAIHQAALLRFRPILMTTLAALFAALPLMLGWGEGAELRRPLGLAIFGGLVLSQLLTLFTTPVIYLAFDRLGRRWTGRGTAAAPVTHAEAGPAAP

>1736456.3.peg.2622_RND

MPHPNTASAVIYSLMLTCRACGVAPLTWLRHVQTGLPQRDEAADIVDLLLFMAYAMMGGIIVGTVVTLLCQFSTWPGSAYRVKTRRRRRRSLGPLEPKTFRISLFSKACCR

>198822.17.peg.3135_RND

MNSDQQQGGLEAMVTFDRSTAARLGIKPAQIDNTLLTPSASARSRPSTTRCRSITW

>199.248.peg.1235_RND

MIKTAINRPITTLMIFLSLVVFGIYSLKTMNVNLYPQVNIPIVKITTYANGDMNYIKTKITQKIEDEISSIEGIKKIYSTSFDNLSVVSIEFELNKDLESATNDVRDKMQKARVGANYEIEKLNGLSSSVFSLFITRLDGNETKLMQEIDDVAKPFLERISGVSKVKTNGFLEPAVKILLDRFKLDKNALSANEVANLIKVENLKAPLGKIENEQIQMAIKSNFSAKSIDEIRNLTIKQGVFLKDIASVDLSYKDANEAAIMDKKSGVLLGLELAPDANALTVIALAKSKLDQFKSLLGSEYDVKIAYDKSEVIQKHIDQTAFDMILGILLTIVIVYLFLRNFSITIISVVAIPTSIVATFFIINALGYDINRLSLIALTLGIGIFIDDAIVVTENIASKLKDEPNALKASFAGIKEIAFSVFAISLVLLCVFVPIAFMSGIVGKYFNSFAMSVAAGIVISFFVSIFLVPTLSARFVNAKQSGFFLKSEPFFEALENFYEKILALALKFKLIFLAITLVVVVCSFTLAKFVGGDFMPSEDNSEFNIYFKLDPSLSLQASKDKLKDKISLINADPQVAYAYFILGYTDAKQPYLVKAYVRLKELKDRVNHERQNAIMQSFRDRLKSDDMSVIVADLPVVEGGDVQPVKLTITSENGKELEKFVPKISKMLKEINDATDVNSPEEDLLKRVQISIDEDKAKRLILDKASVASAVYSAFSQNEVSVFENENGKEYELYMRLDDKFRSDTDDILKTKIRSKEGFFVTLGDVATISFEQKPASISRFNRADEIKFLANTKNNAPLNSVANEISKKLDEILPANFKYKFLGFVELMDDTNASFIFTVSASAVLIYMVLAALYESFLLPFLIMLAMPLAFCGVVIGLFISGNPFSLFVMVGVILLFGMVGKNAILVVDFANHFANNGIEANEAVKMAAKKRLRAVLMTTFAMIFAMLPLALGRGAGFEANSPMAISIIFGLISSTLLSLLVVPVLFAWVYNLDKFIRKFYERERI

>208964.12.peg.1484_RND

MQALRSGGGRVLVGVLAAGLVAFGGWAWLGGDAGAKAAPAPARVPVIVARVERRDVEQQVSGIGTVTSLHNVVIRTQIDGQLTRLLVSEGQMVEAGELLATIDDRAVVAALEQAQASRASNQAQLKSAEQDLQRYRSLYAERAVSRQLLDQQQATVDQLRATLKANDATINAERVRLSYTRITSPVSGKVGIRNVDVGNLVRVGDSLGLFSVTQIAPISVVFSLQQEQLLQLQALLGGEAAVRAYSRDGGSALGEGRLLTIDNQIDSSTGTIRVRASFDNRQARLWPGQFVAVSLHTGVRRDQLVLSSKAVRRGLEGNFVYRVADDRVEAVPVRVLQDIDGLSVVEGLASGDQVVVDGHSRLMPGALVDIQEPRPSLAQATERRP

>244582.5.peg.1519_RND

MNISIPFIRRPIGTTLLAFGLALAGILAFNLMPVSPLPQIEFPTISIQATLPGAAPETMATSVATPLERQLGRIAGITEITSSSRLGTAQITLQFDLSRNINGAARDVQAAINAARSNLPADLPSNPTYKIVNPSDAPIIILALTSDTYSSGQMYDIASTILQQKLSQVNGVGQVIVGGSSLPAVRLELNPTALNKYGISLEQVRTAVAAANNNRPKGQLSDEMHSYIIMTNDQLFKAADYQPLIISYQNSAPIRLSDLGEVIDSVEDLRNAGLSNGKPSVLLIIFKQPGANIIGTVDNVKSALRNLKADIPAAIDLSVVMDRTTTIRASLKDVEFTLILAVCLVIWVIYLFLGNFRAALIPSVVVPLSLLGTFCVMYLCGFSLDNLSLMAMTIATGFVVDDAVVVLENISRHIEAGLKPIQAAILGAKEVGFTVLSMSASLIAVFIPILLMGGIVGRLFREFALTLSIAILMSMVVSLTVTPMMSAYILKPEKKGHHQGRVMNFMMRHYRQSLGWALRRPKFMLTLTAATIASDIFLFVIIPKGFFPQQDVGRIVASIQAQQDISFQALKQKLNDYVKIVKDDPAVETVVGFIGGNSASGNAGTMYISLKPLEERKLPIDDIMGRLRGKLAAIPGASVYMRATQDLVIGGRQSNALYQYTLTSYDLNELNTWAPRVLEKLATLPGIVDVNSDQLSNGKEVFVTIDRDAASRLGVSPQTIDNTLYDAFGQRQIAIMYTALNQYHVVMELAPQYWQRPETLDLIYAPSATNNQIPLSVVTKSKISNTLLLVNHQGQFPAATISFNLLPGYSLGQAVEMINEATTEIGMPKATMHGSFQGTAQAFQDSLSSQPLLILAALIAVYIVLGILYESTIHPITILSTLPSAGIGAMIALLLTGTELSIIAIIGMILLIGIVKKNAIMMIDFALEKERQQHKSAIASIYEACLLRFRPIMMTTMAAILSAVPLAFGSGVGSELRKPLGISIIGGLIFSQMLTLYTTPVIYLSMERVSSWWKRRHKQTSVVVLPLLLLLLNACEVGPDYVRPVIETPAQFKEPPAGWKFATPQDTVDRGTWWDMFNDPLLSNLVAEVELTNQNLALAEAQHRQSQALVDQARAGFFPTINATTSATRQKSFSTGSTNLASAPTNLYNVGLNATWELDVWGSVRRSVESSEAGAEAAAANVALTKLSSEASLTQFYYELRAVDATQKLLDETVGSYQKLLVLTQNRHRMGVSTGLDIAQAESQLKTAEVKAIDNKVTRAQYEHAIAVLVGKAASDFSIPVDSSALPEPPTLPSALPATLMERRPDIAQAERQMAQANATIGVNIAAYFPNLTLNGSGGYESTLWHKLFTAPSQIWSMAGQMAQLVFDGGLVSGKVEAARAAYDQSVANYRQVVLTAFQETEDNLAALRILESEIKSQVEAVKAAKKQLNLTINEYKSGTIYFSDVMTAEINYFTARSNYIAIAARRLTATASLVKSLGGGWCSSDLIREGNWEHKPSPTQQENNR

>318161.16.peg.3236_RND

MFSQFFIKRPIFAAVISLMFFIAGAIAVWKLPITEYPEVVPPTVVVTASYPGANPKVIAQTVASPLEQEINGVEDMLYMSSQATSDGLMTLTITFAIGTDVDRAQTQVQARVDRASPRLPQEVQRLGIVTEKSSPDLTMVVHLTSPDKRYDMLYLSNYAALNVKDELARIEGVGAVRLFGAGEYSLRIWLEPNKMAGLNLSPAQVLAAVREQNQQAAAGSLGAQPSGGADFQLLINVKGRLSTVEEFEDIIINVGPQGELSRLRDVARVELGASTYALRSLLDNQDAIAIPVFQASGSNAIQISDDVRAKMSELSASFPDGLSYDIVYDPTVFVRGSIEAVVKTLFEAILLVVLVVVLFLQTWRASIIPLVAVPVSLVGTFAFMHLLGFSLNALSLFGLVLAIGIVVDDAIVVVENVERNIGDGLSPIAATQKAMREVTGPIIATTLVLAAVFIPTAFMAGLTGQFYKQFALTITISTFISALNSLTLSPALAALLLKGHDAPKDRLTRAMDKLFGTWLFNPFNRMFEKASRGYGFIVKKVIRFGAIVGIIYLALVALTGVMFASTPTGYVPGQDKQYLVAFAQLPDAASLDRTEAVIKQMSEIALAQPGVAHSVAFPGLSINGFTNSPNSGIVFTPLDDFSERTDPSLSAEAIAMQLNQKFAGIEDAYIAIFPPPPVQGLGTIGGFRLQIQDKGNLGYDELYKVTMQVMQKAWGTPELTGVFSSYQVNVPQLDLNIDRTKAKQQGVSLDEVFQTLQTYMGSTYVNDFNQFGRTYQVKMQADEQFRQTPEQISQIKVRNQQGDMVPLGSFINVTQVAGPDRVMHYNAYTTAELNGGPAPGYSSGEAQAAIEKILAETLPNGMTYEWTEITYQQILAGNAGLLVFPLVILLVFMVLAAQYESLSLPMAIILIIPMTLLSALSGVLLYGGDNNIFTQIGLIVLVGLATKNAILIVEFAKELQDEGMNVMDAILEATRLRLRPILMTSIAFIMGVVPMVFSTGAGAEMRQAMGVAVFAGMIGVTIFGLLLTPLFYHFMAKRQKTNVDKNVEPDDSQGQLFAPVVNPAVNTLVTHKGANADA

>343509.12.peg.4097_RND

MTGLDNLLYMASQSTNTGRATTTLTFLAGTDPNEAMQQVQSTAGRTAPSTPGGAKPGHDRKQNRRHQFDDGGFRVYRRVHG

>360107.7.peg.546_RND

MFSKFFIHRPVFACVISIIITLAGLVSLRGLPIEEYPNLTPPQINVFASYPGADAQTIAETVAAPLEDALNGVEDMIYMQSTSSSAGTMRLSIYFKTGTSPQIAQVNVNNRVNLASKLLPDNVTRQGISVFERSDSILEVISFYDPSGQMDIIDLSNYLTINVVDEIKRVNGVGEAFIVGDKKYSMRVWIKPDLLNKYDITTSDVINAISEQNTQYSVGKIGELPENSNSAYVFSIRTEGRLVKVSDFENIIIKSLPNGSALKLKDVANVELGSENYMSNNLINGHYMMPMLVFMQTDGNAIATADAVNKRIEELSKNFPGNLTYNVNYNTTDFVKVSMKEIFQTFIEALVLVLIIMYLFLGNLRSTIIPMIAIPVSIIGTFAGIYAVGFSVNLITLFAMILAIGIVVDDAIIVVENVERNLEENPNISVIEATEKAMEEIMAPIISIVLVLCAVFLPASFIEGFVGIIQRQFALTLVISVCISGIVALTLTPALCAKFLRRDMAKKPKISQWFNKIFDISTNIYAAGVAKILKHIIPSLIVVAILCFCTWRLFTMVPASLVPEEDKGVSIAVSQLPPASTITRTENVIKKQSDELLKNPLIDAVGAMMGYDLFAGGLRENATVIFLKFKDWSERKEKDQSSFAINKKYNILFSQDRNSTTFVLNPPPINGLSLTGGFELFAQNTTGKSFAEIEKDMKVVAAKANARGDLVRVRTTLDTNFPQYKLIVNTQKAKMLNVNIKNLYMTINTMLGQYYVNDFNFLGKTFKVNVKAAGEYRNSVDDLRAIFVKSNDGKSIPVNSLIKLENALGPDTVNRFNGFPAAKIMGDPAEGYTSGQAIDAIAQVFKEEFPNEYTLGWSGTSYQEVQSSGKGATAFIFGLIFVYLILAAQYERWLMPAAVMTAVPFSVFGAILFTYLRGLTNDIYFQIGLILLIGLGAKNAILIVEFAMTEHKKGKNIIEASIAAARLRFRPIVMTSLAFAFGVLPMVISSGAGSASRHSLGTGVIGGMIAASTIAIFFVPLFFYLLETFNNWQAKLSRTKEIKRIRKIRREENA

>401053.4.peg.383_RND

MSHEFQPGDKAPRDGATASDIYRLEHEDTRGGDHAPRDHASPRDEKKQKEDEGPNGGGVHFSAPFIRRPVATFLLSAAIILAGAVAYKLLPVSSLPQVEFPVISVGANLPGADPETMASAVATPLERQFSRIAGINQMTSSSSIGSASITLQFDLTRDINGAARDVQAAINAARSQLPANLPSNPTYRKINPSDAPIMILALTSETLSVPQLYDAADSVLAQKLASVDGVGQTFVGGSSKPAVRIEANPTQLTSYGLGLDALRAAIATINVNQPKGYLNGAGTEGQRWSITTTDQLFGAAAYKPLIVATDRGPVSSAAASNGLQSNVASATTSTTTTNSVSSSGTTGTSSGTATSSASTTSSTASTYSTTATPITTTTATSAAGMASAQVTPSVSNVATPTIGGHGIVRISDVSDVVDSVEDIHNGGLFNLHPAILVIVFKSPGANVIQTVDAINKMLPSLSASISPAIKVQVALDRTATIRASVDDITRTMLITIVLVVLVVFFFLREVRSTLIPAVSVPLSLLGTFGVMYLLGYTLDNLSLMALTISTGFVVDDAIVVIENISRHLEEGLTPYDAAMKGSAEIGFTVVSMSISLIAVFIPILLMGGIVGRLFREFAVTLSVSILVSLCVSLTTTPMLSAKFLQPHSANKHGRIYLLGERFFDWMVGEYTLGLRWVLRHQGLVMLITIGTFLLNIYLFILVPKGFFPQQDTGRLGGRILGQQDVSFDAMKAKAIEMTDLVKQDPGVLNVMTNLGGGGPGGGSSNSANMFIFLKDPAARAKDGDTAEVIINRLRPKLSRMPGVQVYLQSQQELNIGGRQSATQYQYTLQADSVQDLNLWSPKMMAAMQKMPELRDVATDQLENGLESTLVIDRDTASRLGITPLAIDNILSDAFGQRQVSTTYKPLNQYHVVMEVAPQFQKDPDAIRQIYVKNSSGKSIPLTAITHFEMQRIPLQVNHQGLTPAATLSFNLAPGIALSQAAEAIDRARNSISMPASVTGGFQGSAQAFQQSLSSEPVLILLALTTVYIVLGMLYESFIHPLTILSTLPSAGVGAILALLITHTDLSVIAMIGIILLIGLVKKNAILMIDFALVAEREHGKEPVDAIYEACLLRFRPIMMTTMAALFGGLPLAFGTGVGSELRRPLGITIVGGLIVSQCLTLFTTPVVYIYFDKWRQRMESWRGKPVEKKLPRGLRSHPEPVAGD

>436717.3.peg.1877_RND

MQKHLLLPLFLSIGLILQGCGSQETAQAEPAPAKVSVLSIQSQSVNFSENLPARVQAFRTAEIRPQVGGIIERVLFKQGSEVRAGQALYKINSETFEADVNSNRASLNKAEAEVARLKVQLDRYEQLLPSNAISKQEVSNAQAQYRQALADVAQMKALLTRQNLNLQYATVRAPISGRIGQSFVTEGALVGQGDANTMATIQQIDKVYVDVKQSISEYERLQAALKTGELSANSEKTVRISNSHGQEYNVTAKMLFEDINVDPETGDVTFRIEVNNTERKLLPGMYVRVNIDRASIPQALLVPAQAIQRNINGEPQVYVINAKGSAEIRPIEIGQQYEQYYIANKGLKVGDKVVVEGMERIQPNQKLAMTTWKKPASENSASNVETKPSTNQGAQP

>547045.3.peg.622_RND

MASYASKVMRMAAIAAATALALSACNKGSDATQGAKDGKGQQAAAQKEAPPPVVGVVTVHPETVALTTELPGRLESLRTADVRAQVGGIIQKRLFQEGSYVRAGQPLYQIDSSTYQADLESSRAQLAGAQATLAKANADLARYKPLVAADAISKQDYDAAVTAKRSAEASVKAAQAAIKSAGINLNRARITAPISGFIGQSKVSEGTLLNAGDTTVLATIRQTNPMYVNITQSATEVMKLRQQVAEGKLSSVDGAIEVGIKFDNGEVYPHKGRLLFSDPSVNETTGQITLRASVPNDKNILMSGLYVRVLMEQVAADNAFVVPQQAVTRGTKDTVMIVNAKGEMEPREVTVAQQQGTNWVITAGLKDGDKVIVDGIAIASMSGGKKVTPKEWTPPEKAAASAAGAAPKAASEAKKDVQTTSEAKPASAAK

>575.7.peg.976_RND

MKKGEEHGKKGFFGWFNRMFNRNASRYETAVGKILHRSLRWIAIYALLLGGMVFMFLRLPTSFLPQEDRGMFLTSVQLPSGATQQQTLKVVQKVEDYFFNHEQANVASIFATVGSGPGGNGQNVARMFIRLKDWDERDAKTGTSFAIIERATKAFNSINEARVFATNPPAISGLGSSAGLIWSLKTTPATGTRRSWQPAIRCSIWRRKMSG

>648757.4.peg.592_RND

MSGPTAEEPGGVGAQGADAAGEDTYSGISAPFIARPIATSLLAVAILLASLLAYSLLPISSLPQVDFPVVQVTTRLPGANADTMARLVTAPLERQLGQIPSLENMSSTSSEGLSQITLRFMLSRDINAAGQDVQSAISAAGGSLPQNLPYPPVYAKVNPSDPPIVTIALTSQSVSLERLSDFADTLLAPRLSQVAGVGRVTVQGNIRPAIRIQANPLQLASLGIALETVRSAIANANVTGSKGLISGPEKSYIVGANDQLETAGAYEDVVVAYRNKAPVLLRDVATVVAGLENERVAARYNGTPAVVIDVQRQPSANIVGTVDELKKILPKLVDALPAGVKLDIVADRTGTIRASVEEVQFTLVLSVALVIMVVLLFLRTLSATIVAGITLPLSLMAAFGVMYYAGFSLNNLSLMALTIATGFVVDDAIVMIENVMRYIEKGEKPLVAAYKGAGEIGFTIVSLTLSLIAVFIPLLFMEGIVGRLFREFALTLTAAVVTSMIVALTLTPMMAARLLRAPRHGETAPWYSRAFEAPFNALLSVYRVTLDWALNARRFMLLVAAATFVLTVVLYIAIPKGFLPDQDTGFLTAETEAAPGVSFERINALQAEVERIIRRDPDVLGVVSVIGVGTTNATPNAAHLALTLKPKTERKATATEILQRLTEATADFPGLRTTFQIVQDIQIGTARSRTQYQYVIVGLDREGFSGWAQKLEAELSRDRRLIHVASDLQEDGNAVLIKTDRVIAGRLGVTMQALNDTLYDAFGQRQISTIYGQSNQYRVVLEVAPAFQTDTAALGSIYVPGTAISNSTSGNASTGNATASGATNSSITATSASGTGVGSQVPLSSFSVIERATAPLSVNHVQQYPAATISFDVAPGFSLDAAVQAVTDAQSRIALPSSIVGSYTGAAAEFNASLANQPLLILAAVVTIYIILGVLYESFIHPFTILTTLPSAGIGALLALEILGMEFSFIALIGIILLMGIVKKNAIIMIDFALDAERTRGLAPFDAIREACLLRFRPIMMTTVAALLGALPLVIGSGPGSELRMPLGVTIIGGLLLSQLLTLYTTPVIYLAMDGLKRRIERRFGIDEPNYPPPALRPEPGLPDPGPRGGSPRGTGGGGAAGLLPIFGTPSLPMLPPRADWLLLPSPEGNAALPSPNEPLALPPPDKPAS

>679897.3.peg.488_RND

MYKFAIQRPITTLMFAIAVMFFGILGIKKIPVALFPNIDFPIIVISTTYPGGSPEIIESKVTDKVEEAVMGIDGVKKITSNSARNVSIVVVEFYLEKPVEQAMTDVIGKISSIKFDDSNIQQPSIRKFDTSGQAIISLFMSSKQKGPTEIMRHADLIVKPILQSILGVGGVQLNGYRERQIRIYADSTLMNKYGITYDNLFGMLGKENLEANGGRIESATKDFSITVDANSTSIKDIANIRIGKDNVRLSDVAVVEDGLQEETTYAAFNNEPGVIFEVMKVSGANELEVADGVYKALPKIQVASHGYEIVPFLDTTQYIRHSIKDVQFDLMLGGVLAVLIVFLFLRSVTITLVAAISLPISILGTFALIEMLGHTLNMMTMMALTLAIGIIIDDAIVVIENIHKKLELGMSKKQAAYEGVNEIAFAIIAISAMLLSVFVPIANMSGIIGKFFASFGVTVALAIVISYVVVITVIPMVSSLIVSSKQSRFYHFTEPFFNGMENFYLKILRLGLSHKLLFSALTFLIFGFSIYVAKGLGMEFMLKEDKSQFYVWLETSPGISIHEMKVRTLALQEAIAKHEEIEYTTLQVGYGSIQSIFKAKIYAKMKPIEERKISQFDMMKSITDELKKMPQAKGLNVFSSEVPVLGGGDSTPFQVTIYGMTQQAVDKSVAKLKKMLDEDPRFQGKITNYHTSTSDIQPEYKITVLRQNADKYGVRTQEIANVVSAAFSGVNQAAYFKQGGKEYKITMRVPDDERVSVDDIRKLQVMNSSGKLMFLDGLVEITRSQSPSLINRYGRQRSVTVYAAPLKNSGLSLGSMISIVQTNSKDWLEEGVNFAFSGESNNAAESAASFMTAIITAFILIYLILAALYESLLEPFIIMITMPLSFAGVFFSLKLAHQPFSMFSFMGLILLIGIVGKNATLLIDVANEYRKKFKAGVHEAIIFAGKSRLRPILMTTIAMVFGMLPLAVATGSGYAMKSPIGISMIGGLLISMFLSLLMVPILYVIVAPIDDKLKRFYQSEDGEGILQSVVKKIKPGKKEKEKQEEDKDSKKKKKKKDKKKD

>754502.3.peg.2425_RND

MNISRLFILRPVATLLLMIALVLVGLIAMRVLPVSSLPNVDYPTIQVQTFYPGASPTVMATTVTAPLEVQLGEIPGLQQMTSYSSDGASVITLQFDLSLNLDIAEQNVQQAINAANSYLPSGLPAPPTYAKVNPADQPILTLAVTSKSMSLTQLEDVANNRLGTKISEVSGVGVVTTSGGNVPAIRVEADPHKLAAYGLNIDDLRTLLSYVNVSQPKGNFDGPDLDYTINGNDQITDPKDYLDTVIAYQNGSPVFMRDVARVSQAAQDVERGAWYNGSPAIVLNVQRQPGANVIKTVNQIMKELPQLESTLPAGMKVTVVSDSTGVIRASVADAAFELILAIVLVVAVIFVFLRNVPATLIPSISVPVSLIGTLAVMYQLNYSIDNLSLMALIIATGFVVDDSIVMIENIVRYLEEGMSPLEAALEGAGQIGFTILSLTVSLIAVLIPLLFMGGVIGRLFSEFAVTLAVTIVISAVVSLTVVPMLCARMLRAQAERHPSRFERISEGLFDKTLAAYERGLRWVLDHQTLTLMVAIATVVLTGILYVVIPKGLFPVQDVGVIEGISVADNSVSYAAMVQRQSALADAVLKDPDVVSLTSYVGIDGTNATLNNGRFLINLRERDKRSDNAQEIARRLAQEVAHVPGVKLFMQPEQDLTLDTTVSPNQYSFALRGPSQQAFQKYVPELVARLKRIPSLSDVQSDLNSDGLSVNVEVNRQLAARFGITPATIDNALYDALGQRIVSTIFEQSAQYRVILVAKPETMPTLQSIGDLYLPSQTSSTGQVPLSGIAKIEIRKAPLVISHLAQFPAVTVSFNLAKGASLSTAVKEIHQAEQAIDLPPSITSSLQGATAAFEDSLSSEVYLLIAALVAVYIVLGVLYESFIHPVTILSTLPSAGIGALLSLMLAGMDLDVIGIIGIVLLIGIVKKNAIMMVDFALDAERNHGKAPRDAIFEASLLRFRPILMTTLAAMLGALPMLLGTGTGSELRRPLGLAIIGGLTLSQMLTLFTTPVIYLFFDRMAARVNRWRAARAERNGGDEPGGRPPEGGAGGTRVNIPAIFIRRPVATTLLAIAILISGTLAYFRMPVAPLPNIAFPVIVVQANMAGASPSVMASTVAEPLERRLATIADVEELTSISYVGSSMIIVEFGLKRDINGAARDVEAAIQAARADLPTTLRSNPSYRQYNPADAPIMVLSLTSDTLTKAQLYDSADSVIQQQLSQVRGVGQITLGGGALPSVRVELQPGKLNSYGIGMEDVRAAISAANADSAKGHLDVGDQRYVVTSNDQITHAAPYRDLVVAYRDGAPVQLRDVAQVRDSNENIRNAGLFNGKSAILVIVYPMPGSNVVSTVRQIRNVLPSIQATLPSSVHVDVAIDRSQSVTSSVSDTERTLFIAVLLVVGVVFIFLQSPRATLVPAVALPLSIVGTFGPMYLLGYSIDNLSLMALTIGTGFVVDDAVVVLENVVRYIEQGLSPKEAALKGAGEVGFTVISMSLSLIAVFLPIILFPGIVGLMFHEFAITLSIAILISLVISLTVTPAMCAYVLSRDHAGHSRARWAQWIERQFDRFKGVYARSLTAVLDHSLLVILLLFALLVGNVFLLKLVPATFFPEQDTGILIGQIIADQSISFSAMQKKLAQLQSIVQRDPAVQSVAGFTGGRALNTANVFIELKPLSQRHATAAQIVNRLRPKLNQVSGARLFLQAQQDLRIGGRQSAAEYQYTLTSDDSAALFTWTPKLVAALSKERGRLLDVNSDLQQNGLQTYVSINRATAARYGFAPNQVDNVLYDAFGQRTVSTIYNPLNQYFVVMEVAPEYWQYPQTLNQIYLSKSAGNPSGTAATQMPHGTVSALSSTNASTSSTSSTTNSRNSDAQSNATNNSIANSKGGSSTGSADSTAAETMVPLAVMASYASSHTSTQVNHQSGLVAATISFNLPAGGSLSQAGAAINDTIREIGMPASIHGSFAGAAAAYSQSMGVVPLLILAALAVVYIVLGVLYESSIHPLTILSTLPSAGIGATLALLIFGTPFSVIAMIGIILLIGIVKKNGIMMVDVAIQLQRQQQMTARDAIHEAALIRLRPIMMTTFAAVLGAVPLAIGIGQGGSLRQPLGITVMGGLILSQMFTLYTTPVIYLYLDRLRARLVRWSAGLRWNRDAKPGQPDTMA

>887898.3.peg.390_RND

MNLSRPFIRRPIGSTMLALAILLAGWLAWRQLPVAPLPQIDTPMVVVSASLPGASPTSMAATVAGPLERALGAIAGLSSISSSSSTGTTEVRLFFDIDRDLNEASREVQAAINGVIDQLPPGMPGRPTFRKLNSSTSPILALALSSATLPPSQLYDLADNIVLQKISRVQGVGEVSLGGASLPAVRIRFEPSALAALGMSLEDARQVVVAASAEAPEGFLEDEGNRWLVATGHKLKNAADFSDLVLRWKNGQAVRLSDVAEVSDSVENRYSSGFHNHQPAIIALVTRQPDANVVATIDAIKATLPQLQAILPPQASLTVVMDRSLGIRGSLAEAQWTLVFSCLIVAAVVWLFVARLRTALIPVAVIPVSLIGTFAVIWLAGFSLNNLSIMALVVAAGLVVDDAIVVLENITRHTERGLSPYRAAMRGAGEVSFTLLALNVALVVVFVAVLFMGGIIERLFREFSLTLAAAIVISLVVSISLTPALCAHGLPRERRQKAAEHGAAQAAALPGGMPDVTHDAGQGMPGSVSLQDDAEVARAPWHRRLLGLHASYFHHLQAAYEQSLAWMLRYAWYGVVALVGLIAASVWLFANLPRSDLPEQDTGVIGAFIRGDDGFSFQIMQPRIERYRRWILSDPAVQDVAGISGGNGGLTNARLVITLKPLAERKVSARQVIDRLRRNAPQMAGTMFFGRVEQDLQLSPPKFGDDADHVIVLKSGDRDLLRTWNQRLGVALSKRPELENVRYSLGEDTRQIVLDIDRNTASRLGVQLTDISAALSNSFAQRQVATLYQDRNQYRVVMEVSERFTENPLALDRVQIITSEGKSVALAEVARWHFGMVQDRERHVDQFSASTISFSVAADVTDTAALEAVRKVIDAERMPVTVIADIDGDDGRPKSLVKADGQGWLILGVVLAVYLVLGILYENLLHPITVLSTIPSAGVGALLALWASNTPFSLIALLGLFLLIGVVMKNGILMIDVALKKQLHEGLAPQVAILQAAGQRLRPILMTNVAALAGAIPLAMGLGDGGELRRPMGLVIIGGLAVSQLITLYTTPALYLLLERLQQRLRRGRG

>P9WJV1_RND

MIVQRTAAPTGSVPPDRHAARPFIPRMIRTFAVPIILGWLVTIAVLNVTVPQLETVGQIQAVSMSPDAAPSMISMKHIGKVFEEGDSDSAAMIVLEGQRPLGDAAHAFYDQMIGRLQADTTHVQSLQDFWGDPLTATGAQSSDGKAAYVQVKLAGNQGESLANESVEAVKTIVERLAPPPGVKVYVTGSAALVADQQQAGDRSLQVIEAVTFTVIIVMLLLVYRSIITSAIMLTMVVLGLLATRGGVAFLGFHRIIGLSTFATNLLVVLAIAAATDYAIFLIGRYQEARGLGQDRESAYYTMFGGTAHVVLGSGLTIAGATFCLSFTRLPYFQTLGVPLAIGMVIVVAAALTLGPAIIAVTSRFGKLLEPKRMARVRGWRKVGAAIVRWPGPILVGAVALALVGLLTLPGYRTNYNDRNYLPADLPANEGYAAAERHFSQARMNPEVLMVESDHDMRNSADFLVINKIAKAIFAVEGISRVQAITRPDGKPIEHTSIPFLISMQGTSQKLTEKYNQDLTARMLEQVNDIQSNIDQMERMHSLTQQMADVTHEMVIQMTGMVVDVEELRNHIADFDDFFRPIRSYFYWEKHCYDIPVCWSLRSVFDTLDGIDVMTEDINNLLPLMQRLDTLMPQLTAMMPEMIQTMKSMKAQMLSMHSTQEGLQDQMAAMQEDSAAMGEAFDASRNDDSFYLPPEVFDNPDFQRGLEQFLSPDGHAVRFIISHEGDPMSQAGIARIAKIKTAAKEAIKGTPLEGSAIYLGGTAAMFKDLSDGNTYDLMIAGISALCLIFIIMLITTRSVVAAAVIVGTVVLSLGASFGLSVLIWQHILGIELHWLVLAMAVIILLAVGADYNLLLVARLKEEIHAGINTGIIRAMGGSGSVVTAAGLVFAFTMMSFAVSELTVMAQVGTTIGMGLLFDTLIVRSFMTPSIAALLGKWFWWPQVVRQRPIPQPWPSPASARTFALV

>P32714_RND

MINRQLSRLLLCSILGSTTLISGCALVRKDSAPHQQLKPEQIKLADDIHLASSGWPQAQWWKQLNDPQLDALIQRTLSGSHTLAEAKLREEKAQSQADLLDAGSQLQVAALGMLNRQRVSANGFLSPYSMDAPALGMDGPYYTEATVGLFAGLDLDLWGVHRSAVAAAIGAHNAALAETAAVELSLATGVAQLYYSMQASYQMLDLLEQTHDVIDYAVKAHQSKVAHGLEAQVPFHGARAQILAVDKQIVAVKGQITETRESLRALIGAGASDMPEIRPVALPQVQTGIPATLSYELLARRPDLQAMRWYVQASLDQVDSARALFYPSFDIKAFFGLDSIHLHTLFKKTSRQFNFIPGLKLPLFDGGRLNANLEGTRAASNMMIERYNQSVLNAVRDVAVNGTRLQTLNDEREMQAERVEATRFTQRAAEAAYQRGLTSRLQATEARLPVLAEEMSLLMLDSRRVIQSIQLMKSLGGGYQAGPVVEKK

>Q2EHL7_RND

MFTIKKLTLTIVVATTLTGCANIGDSYRASLKNYKQYEEITKQYNIKNDWWKLYKDAQLNRVVEKALLNNKDLAKATISVNRALYSANLAGANLVPAFSGSTRSTAQKNIKTGGNSTISHTGSLNVSYTLDLWFRLADTADAAEWAHKATVQDMESTKLSLINSVVTTYYQIAYLNDAISTTKESIKYYTDISNIMRNRLAQGVADSISVDQAQQAVLTARNNLITYQLNRKTAEQTLRNLLNLKPDETLKITFPHILKVKSVGVNLNVPVSVIANRPDIKGYQARLSSAFKNVKATEKSWFPEITLGGSLNSSGKKLNSATNTLIGGGALGISLPFLNWNTVKWNVKISEADYETARLNYEKSITVALNDVDTNYFSFTQAKKRFTNAQKTYIYNQRITQYYRNRYNAGVSELREWLTAANTEKNSQLSILQAKYNVIQAENAVYSSMAGYYSVKK

>Q83KF5_RND

MNRDSFYPAIACFPLLLMLAGCAPMHETRQALSQQTPAAQVDTALPTALKNGWPDSQWWLEYHDNQLTSLINNALQNAPDMQVAEQRIQLAEAQAKAVATQDGPQIDFSADMERQKMSAEGLMGPFALNDPAAGTTGPWYTNGTFGLTAGWHLDIWGKNRAEVTARLGTVKARAAEREQTRQLLAGSVARLYWEWQTQAALNTVLQQIEKEQNTIIATDRQLYQNGITSSVDGVETDINASKTRQQLNDVAGKMKIIEARLNALTNHQTKSLKLKPVALPKVASQLPDELGYSLLARRADLQAAHWYVESSLSTIDAAKAAFYPDINLMAFLQQDALHLSDLFRHSAQQMGVTAGLTLPIFDSGRLNANLDIAKAESNLSIASYNKAVVEAVNDVARAASQVQTLAEKNQHQAQIERDALRVVGLAQARFNAGIIAGSRVSEARIPALRERANGLLLQGQWLDASIRLTGALGGGYKR

>Q8FWV8_RND

MVAFWTCRNAWFQHLPFAKRGDENAPSGPRRLRPWFLVLALGLAACSEDKSAPQQAAPLPPIPVGVIKITERPTHPQLSFVGRVEATDSVDLIARVDGFLDKRTFTEGQAVKTGDLLFVLQKDALQAALDAAQANLAKAQADADNLKLQTERARSLYKQKTVSQAMLDDRVAAEKQALAVVQQAQASLEQAQINLGYTDIRAPFSGRIGMANFSVGALVGPSSGPLATIVSQDPIYVTFPVSDKTILDLTEGGRTATDRSNVAVSLTLSNGMTYPQTGAIDFTGIKINPNTDTLMVRAQFPNPNNVLIDGQYVQVTAASKHPVEALLVPQKAIMTDQSGNYVLAVGEDNKVIQRQITQGSTFGSNVVVKSGLAVGDQVVVDGLQRIRPGQKVDPQIVDATTPAQKAMSVGN

>Q8FWV9_RND

MLSSVFINRPRLAIVIAIVITLAGLIAVTRIPVAQFPDIVPPQVSVTATYPGASAETVEAAIAQPIEAQVNGVDDMIYMSSTSGNNGTYTLTVTFKVGSDPNLNTVNVQNRVRLAEANLPQEVTRLGVTVKKQSSSFLQIITLLSPDSRYDELFLNNYGVINVVDRLARVPGVGQAQSFGTFNYSMRIWFNTDALTSLNLTPNDIVNAISSQNVQAAVGRLGAPPMTDQQQIQLTLTTQGRLTDAKQFENIIIRANPDGSSVRLKDVARVELAAQSYDTIGRLNGKPASVIAVYQAPGSNAVAAAEGVRNVMEQLKQSFPAGLDYKITYDTTVFVSSTIHEVIKTLLEAFVLVVVVVFIFLGNFRATLIPTLAVPVSLIGTFAVLLVLGFSANTISLFAMILAIGIVVDDAIVVVENVERVMAETGLPPKEAAKQAMQEITAPIIAITLVLLSVFVPVAFIPGITGALYAQFALTVSVAMLISAINALTLSPALCGVFLKPHQGRKKSLYGRTMDKLSSGIEKISDGYAHIVRRLVRMAFLSIVLVAGLGAGAYFLNTIVPTGFLPEEDQGLFFVQVNLPPAASQSRTAAVVSEIEADITKMAGVADVTSVTGFSFIDGLAVSNAGLMIVTLKPLEERLKDNITVFDVIAEVNRRTAAIPSAVAITMNLPPILGLGSSGGFQYQLEDQEGQSPQQLASVAQGLVMAANQNPKLSRVFTTFATDTPQLNLNIDRQKALSLGVSPNNIIQALQSTLGGYFVNNFNTLGRTWQVIIQGEQQDRKTVEDIYRINVRSSHGDMVPLRSLVSVEERLGPLYITRYNNYRSASIQGNAAPGVSSGEALAAMAQVSKTTLPSGYGYEWTGTALQELQAAGQTSMILALAVLFAYLFLVALYESWTIPVGVLLSVTAGLAGAMLALWITGLSNDIYAQIGIVVLIALASKNGILIVEFAKERREEGVPLEQAAIIGARQRFRPVMMTSFAFILGLVPLVIAVGAAAASRRAVGTSVFGGMIAASAVGIFLIPMLYVVLERVREWGHARILRKPLYEEEKQEKADGDASGPTVPPTQPEDRGLS

>Q8G2M7_RND

MTLNRTIRCFAAGAAFIVFAAQPALAQAPGGATPPPPQVFVVDIKPHDVPVTYEYAARINAYRNVQVRARVGGILLHRNFVEGTQVKAGEVLFEIDPAPYQAELEKAQAQVAQAEAQYQQSIRDAERAEQLVQQKVQSAAVRDSAFATRDLNKAAVAAAKAQLRTAELNLSYTKVTAPISGITSQEQVNEGSLIGTDASSSLLTSVTQLDPVYVNFSFTDTEAAEIAKLRAERGATGEDADRLKIKILFGDGKAYDHEGTIDFTSSSLDTETGTLGVRAVVENPNHRLIPGQFVRAEILDIQVKDAITVPKAALMQSAQGQFVYVVNKDNVVEVRPVTGARELKNDWLISQGLNSGDRVITEGVIKAVPGRPVQPVVQGVDDKAQAEAGKEQAADKK

>A0A0P7CXJ9_RND

MGFNLSAWALRNRQIVLFLMILLAAIGAMSYTKLGQSEDPPFTFKAMVIRTLWPGATAEEVSRQVTERIEKKLMETGEYERIVSFSRPGESQVTFMARDSLHSKDIPELWYQIRKKVADIRHTLPPEIQGPFFNDEFGTTFGNIYALTGEGFDYAVLKDYADRIQIQLQRVKDVGKVELIGLQDEKIWIELSNVKLATLGVPLEAVQQALQEQNAVSTAGFFETPSERLQLRVSGRFDSVEQIRQFPIRIAERTFRIGDVAEVHRGFNDPPAPRMRFMGEDAIGLAVSMKDGGDILVLGKALESEFERLARSLPAGMELRKVSDQPAAVKAGVGEFVQVLVEALVIVLLVSFFSLGLRTGLVVALAIPLVLAMTFAAMHYFGIGLHKISLGALVLALGLLVDDAIIAVEMMAIKMEQGYDRLKAASYAWSSTAFPMLTGTLITAAGFLPIATAASSTGEYTRSIFQVVTIALLTSWVAAVVFVPYLGERLLPDLAKLHASRHGKDGHAPDPYATPFYQRVRRVVEWCVRRRKTVILLTIAAFVGSILLFRFVPQQFFPASGRPELMVDLKLAEGASLANTAERVKQLEALLKQQEGIDNYVAYVGTGSPRFYLPLDQQLPAASFAQFVVLAKSMEDRERLRSWLISTMDQQFPDLRARVTRLENGPPVGYPVQFRVTGEHIEKARALAREVADKVRQNPHVVNVHLDWEEPSKAVFLEIDQDRARALGVSTAHLSSFLQSSLTGTTVSQYREDNELIEILLRGTRQERSELGNLGSLALPTDNGQSVALSQVATLEYGFEEGIIWHRNRLPTVTVRADIYDKEQPATLVKQIEPTLRDIRAKLPDGYLLEVGGTVEDSERGQKSVNAGMPLFVVVVLSLLMIQLRSFSRTVMVFLTAPLGLIGVTLFLLVFRQPFGFVAMLGTIALAGMIMRNSVILVDQIEQDIAAGLDRWQAIIEATVRRFRPIVLTALAAVLAMIPLSRSVFYGPMAVAIMGGLIVATVLTLLFLPALYAAWFRVKKA

>W0HU59_RND

MNISRLFIFRPVATLLLTLAILLLGLLGYRLLPVAPLPQVDFPTIMVSASLSGASPETMAATVATPLERSLGQIAGVTEMTSSSSTGSTRIILQFELDRDINGAARDVQAAINAARSLLPSSMPSLPTYRKANPSDAPIVMLALTSNTRASGELYDLASSTIQQKIAQVQGVGQVSLLGSALPAVRIDLQPQMLNHLGISLDTVRSAIANSTTNLPKGMLQGATTSFVVDGNGQLDKARDYRSLIITYINGTAIRLSDVATVTDSVEDKYNIGFYNQTPSVMIGVTRQAGANMLETIDAINAALPALQAELPGDVELHKVVDRSPTIRASLYDTEETLLIAIFLVIAVVFIFLRNLQAVIIPALALPVSLIGTCAVMYLLDYSLDNLSLMALIICTGFVVDDAIVVLENITRYIEEGLGPVRASIKGAQEVGFTVLAMTLSLVAVFIPILLMGSIVGRLFREFAVTLTVSLLISMVVSLSLTPMLCSRLLRRKPPVSKRPNRLYLLIESGLARLLAGYALALGWVMRHQRLTLFSLVLTIMLNLFLYGVVQKGFFPNQDTGLLMGMVRADQNISFQAMKPKVEAIAKLIQQDSAVDGVMSSIGGGAFGSRNSGTFFVRLKDYDKRSDSATVVANRLTNKFRNEAGMQLFLMAAQDLHIGGRSANASYQYSLQADDLNLLRVWTPKVKAALEKLPELTSVDADSENGGQEIMLNIDRDKATRLGVNADMLDAMLNNSFSQRQVATIYKTLNQYHVIMGLNEAYTGDAEVLKKLFVVNDNGESIPLSAFITFSSANAALSVAHQGQSATSTVAFNLADGVSLEQAQAAIKDAMVKIALPSTIQAGFQGTAKAFAALAASMPWLILAALAAVYIVLGVLYESYIHPLTILSTLPSAGLGALLLMLVTGTQLTVIALIGILLLIGIVKKNAIMMIDFALAAERNQGLTPQQAITQACLMRFRPIMMTTLAAFFGALPLALGSGGDADLRSPLGMAIAGGLALSQLLTLFTTPVVYLYLDRLSRNSQRAWHRLRKTGTA

1. **Small Multidrug Resistance (SMR): 4 protein sequences**

>sp|Q65JB2|EBRB_BACLD Multidrug resistance protein EbrB OS=Bacillus licheniformis (strain ATCC 14580 / DSM 13 / JCM 2505 / NBRC 12200 / NCIMB 9375 / NRRL NRS-1264 / Gibson 46) OX=279010 GN=ebrB PE=3 SV=1

MKGMIFLAAAILSEVFGSTMLKLSEGFSAPLPAAGVIIGFAASFTFLSFSLKTLPLSAAY

ATWAGTGTALTAAIGHFIFQEPFNLKTLIGLTLIIGGVFLLNSKRTEAADQKAQLTIEI

>502347.3.peg.3992_SMR

MPFVFSAIVTKVIVEIPLPPGKISVQLPALRDDLQTRLFIGDGPNSSEPDMSWIILVIAGLLEVVWAVGVMTPTY

>56780.15.peg.233_SMR

MLSPFRALAAVACPPAISEQKTGASRIFPEIGLASSSSSSRSLVFTISFRTQIGRYIMKGWLFLVIAIVGEVIATSALKSSEGFTKLAPSAVVIIGYGIAFYFLSLVLKSIPVGVAYAVWSGLGVVIITAIAWLLHGQKLDAWGFVGMGLIIAAFLLARSPSWKSLRRPTPW

>214092.21.peg.591_SMR

MAVFCYLGLAILPLIIEHDNVSRLSLCWLGRPSYASVFRGRPRLSEVVTMAWIILVIAGLLEVIWAIGLKYSHGFSRLTPSIITLVAMAASVFLLAYAMKSLPAGTAYAVWTGIGAVGTAILGIVLLGESASLARILSLGLILAGIIGLKLAS
